# Spatial transcriptomics reveals human cortical layer and area specification

**DOI:** 10.1038/s41586-025-09010-1

**Published:** 2025-05-14

**Authors:** Xuyu Qian, Kyle Coleman, Shunzhou Jiang, Andrea J. Kriz, Jack H. Marciano, Chunyu Luo, Chunhui Cai, Monica Devi Manam, Emre Caglayan, Abbe Lai, David Exposito-Alonso, Aoi Otani, Urmi Ghosh, Diane D. Shao, Rebecca E. Andersen, Jennifer E. Neil, Robert Johnson, Alexandra LeFevre, Jonathan L. Hecht, Nicola Micali, Nenad Sestan, Pasko Rakic, Michael B. Miller, Liang Sun, Carsen Stringer, Mingyao Li, Christopher A. Walsh

**Affiliations:** 1 Division of Genetics and Genomics, Department of Pediatrics, Boston Children’s Hospital, Harvard Medical School, Boston, MA USA; 2 Howard Hughes Medical Institute, Boston Children’s Hospital, Harvard Medical School, Boston, MA USA; 3 Statistical Center for Single-Cell and Spatial Genomics, Department of Biostatistics, Epidemiology and Informatics, Perelman School of Medicine, University of Pennsylvania, Philadelphia, PA USA; 4 Research Computing, Department of Information Technology, Boston Children’s Hospital, Boston, MA USA; 5 Department of Neurology, Boston Children’s Hospital, Boston, MA USA; 6 Broad Institute of MIT and Harvard, Cambridge, MA USA; 7 University of Maryland Brain and Tissue Bank, Department of Pediatrics, University of Maryland School of Medicine, Baltimore, MD USA; 8 Department of Pathology, Beth Israel Deaconess Medical Center, Boston, MA USA; 9 Department of Neuroscience, Yale School of Medicine, New Haven, CT USA; 10 Department of Psychiatry, Yale School of Medicine, New Haven, CT USA; 11 Kavli Institute for Neuroscience, Yale School of Medicine, New Haven, CT USA; 12 Wu Tsai Institute, Yale University, New Haven, CT USA; 13 Division of Neuropathology, Department of Pathology, Brigham and Women’s Hospital, Harvard Medical School, Boston, MA USA; 14 Department of Neurology, Brigham and Women’s Hospital, Boston, MA USA; 15 Janelia Research Campus, Howard Hughes Medical Institute, Ashburn, VA USA; 16 Department of Pathology and Laboratory Medicine, Perelman School of Medicine, University of Pennsylvania, Philadelphia, PA USA; 17 Departments of Pediatrics and Neurology, Harvard Medical School, Boston, MA USA

**Keywords:** Neuronal development, Developmental neurogenesis, Developmental neurogenesis

## Abstract

The human cerebral cortex is composed of six layers and dozens of areas that are molecularly and structurally distinct^1–4^. Although single-cell transcriptomic studies have advanced the molecular characterization of human cortical development, a substantial gap exists owing to the loss of spatial context during cell dissociation^5–8^. Here we used multiplexed error-robust fluorescence in situ hybridization (MERFISH)^9^, augmented with deep-learning-based nucleus segmentation, to examine the molecular, cellular and cytoarchitectural development of the human fetal cortex with spatially resolved single-cell resolution. Our extensive spatial atlas, encompassing more than 18 million single cells, spans eight cortical areas across seven developmental time points. We uncovered the early establishment of the six-layer structure, identifiable by the laminar distribution of excitatory neuron subtypes, 3 months before the emergence of cytoarchitectural layers. Notably, we discovered two distinct modes of cortical areal specification during mid-gestation: (1) a continuous, gradual transition observed across most cortical areas along the anterior–posterior axis and (2) a discrete, abrupt boundary specifically identified between the primary (V1) and secondary (V2) visual cortices as early as gestational week 20. This sharp binary transition in V1–V2 neuronal subtypes challenges the notion that mid-gestation cortical arealization involves only gradient-like transitions^6,10^. Furthermore, integrating single-nucleus RNA sequencing with MERFISH revealed an early upregulation of synaptogenesis in V1-specific layer 4 neurons. Collectively, our findings underscore the crucial role of spatial relationships in determining the molecular specification of cortical layers and areas. This study establishes a spatially resolved single-cell analysis paradigm and paves the way for the construction of a comprehensive developmental atlas of the human brain.

## Abstract (web summary)

Multiplexed error-robust fluorescence in situ hybridization (MERFISH) together with deep-learning-based nucleus segmentation enabled the construction of a highly detailed and informative spatially resolved single-cell atlas of human fetal cortical development.

## Main

Abnormal development of the cerebral cortex is linked to a wide range of neurological disorders, including autism spectrum disorder, epilepsy, intellectual disability and various neuropsychiatric conditions^1^. Different areas of the cortex, such as the frontal lobe and occipital lobe, display considerable variations in neuronal subtypes and layer cytoarchitecture, which provides the cellular and structural basis for area-specific circuitry^3,5^. Two models have been proposed to explain how distinct cortical areas are formed during development: the protomap hypothesis and the protocortex hypothesis^10–12^. The protomap hypothesis posits that morphogen gradients along the anatomical axis pattern neural progenitor cells, which in turn transmit map information to the neurons that they generate^13–15^. By contrast, the protocortex hypothesis suggests that cortical neurons initially form a homogeneous sheet, with area-specific identities induced later by extrinsic factors, most notably interactions with thalamocortical axons^16–18^. Evidence supporting both models have emerged from various animal studies. However, the process of cortical arealization in humans remains poorly characterized owing to the limited accessibility of human fetal tissues for experimental studies.

**Fig. 1:**
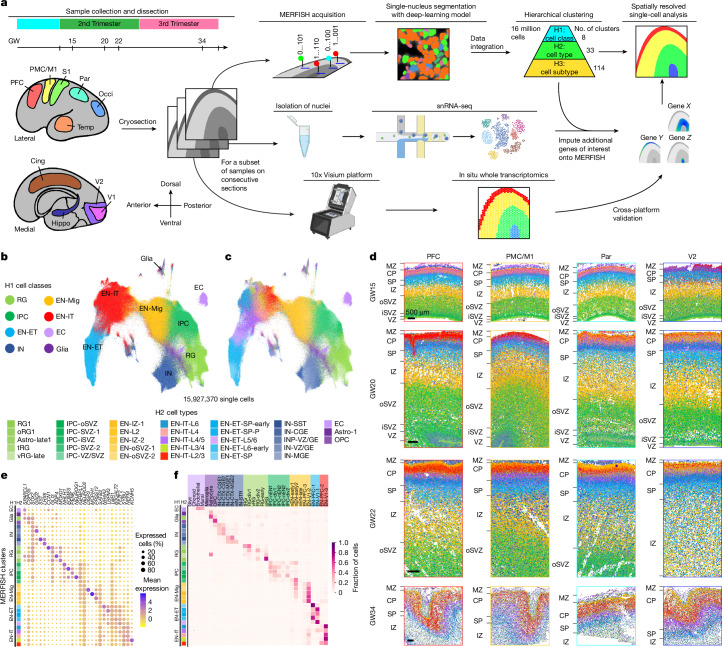
A spatially resolved single-cell atlas of human fetal cortical development. **a**, Schematic of sampling and workflow. **b**,**c**, UMAP of single cells analysed by MERFISH, coloured by H1 cell classes (**b**) and H2 cell types (**c**). **d**, Spatial maps of H2 cell types from major cortical areas across GW15–GW34. Scale bars, 500 µm. **e**, Dot plot showing the expression of top marker genes for H2 cell types. **f**, Cell-type correspondence heatmap shows the fraction of cells from MERFISH H2 clusters that associate to clusters from a published mid-gestation scRNA-seq dataset^7^. Cing, cingulate cortex; Hippo, hippocampus; Occi, occipital cortex; OPC, oligodendrocyte precursor cells; oRG, outer radial glia; S1, primary somatosensory cortex; tRC, truncated radial glia; vRG, ventricular radial glia. A full glossary of acronyms and abbreviations used throughout the main text and figures is provided in Supplementary Table 1. The schematic in **a** was created using BioRender (https://www.biorender.com).

Achieving a comprehensive understanding of human cortical development requires methods that simultaneously analyse both molecular and structural features in intact tissue architectures. Conventional single-cell transcriptomics is limited by the loss of spatial context during cell dissociation, which makes it impossible to determine whether cell types in neighbouring layers and areas with indistinguishable morphology are spatially segregated or intermingled^5–8^. In situ whole-transcriptome sequencing methods, although capable of detecting mRNA in a spatial grid, typically lack single-cell resolution because each capture spot encompasses multiple cells—a challenge further exacerbated by the high cell density of the fetal brain^19,20^. By contrast, the microscopy-based MERFISH method offers a solution, enabling precise transcript detection in a targeted gene panel at true single-cell resolution^9,21,22^. In this study, we used MERFISH to analyse human fetal cortex samples across major cortical areas, focusing on the molecular and cellular specification of cortical layers and areas during the second and third trimesters (Fig. 1a). We complemented MERFISH with single-nucleus RNA sequencing (snRNA-seq) and in situ whole-transcriptomics (10x Visium) on consecutive tissue sections for a subset of samples (Fig. 1a and Methods). Collectively, our integrated approach enables spatially resolved single-cell analysis of the developing human cortex on an exceptional scale to reveal crucial insights into the development of cortical layers and areas that were previously not attainable with traditional methodologies.

## A spatial atlas of the human fetal cortex

We analysed fetal human tissues from a total of ten individuals, with six included in the initial analysis and four subsequently added. The initial dataset encompassed samples from gestational week 15 (GW15), GW20, GW22 and GW34, spanning eight major cortical areas along the anterior–posterior (AP) axis (Fig. 1a and Supplementary Table 2). For MERFISH analysis, we curated a panel of 300 genes, including canonical marker genes for major cell types, alongside genes selected for their cluster-specific enrichment in a published single-cell RNA sequencing (scRNA-seq) dataset of a mid-gestation human fetal cortex^7^ (Methods and Supplementary Tables 3, 4).

**Extended Data Fig. 1:**
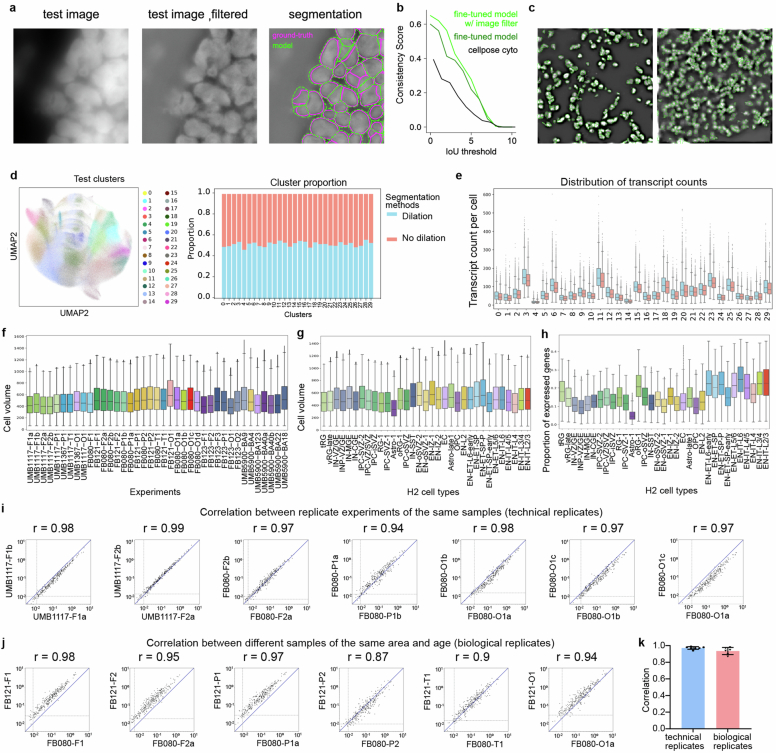
Single-nucleus segmentation and reproducibility of MERFISH. **a**, Example test image (left), test image filtered with difference of Gaussians filter (mid), and filtered test image overlaid with ground-truth manual segmentation (magenta) and segmentation from the fine-tuned CellPose 2.0 model trained using filtered images (green)(right). **b**, Consistency of the segmentation algorithm comparing with human manual labeling as the ground truth. The consistency is quantified using the average precision (AP) metric, at varying intersection-over-union (IoU) thresholds. The AP is defined as the number of true positives divided by the total number of true positives, false negatives, and false positives. The fine-tuned model trained with filtered images outperformed the fine-tuned model trained on the raw images and the built-in “CellPose cyto” model. **c**, Example segmentations of fields of view from two experiments (repeated 44 times). **d**, UMAP projection of unsupervised clustering of cells processed with and without the 10-pixel dilation shows unbiased representation across all clusters. To test if the dilation affected the accuracy of cell type identification, one experiment (FB080-O1c) was separately processed with the 10-pixel dilation disabled, and the resulting cells were co-clustered with the dilated cells. **e**, Dilated cells show modest increase in transcript counts over non-dilated cells across all clusters (total n = 1,001,363 cells distributed across 30 clusters). **f, g**, Box plots show that the distribution of volumes of individual cells after CellPose segmentation were consistent across experiments (**f**) and across cell types (**g**) (total n = 15,927,370 cells distributed across experiments and cell types). Box plots show media value as center; lower and upper bounds of box correspond to the first and third quartiles; whiskers extend up to 1.5 times the interquartile range from the lower and upper bounds of the box. **h**, Box plot showing the distribution of proportion of expressed genes among the 300 panel genes in cells from different H2 cell types (total n = 15,927,370 cells distributed across experiments and cell types). Our MERFISH panel design placed an emphasis on excitatory neurons, leading to more genes detected in EN cell types than others. **i, j, k**, Scatterplots and summary histogram (mean ± standard deviation) show the average count per cell of individual genes is consistent between two replicate experiments from the same sample (n = 7 pairs of technical replicates) (**i**), and between two independent donors with matching gestational age and cortical area (n = 6 pairs of biological replicates) (**j**). The blue solid line indicates equality. The grey dashed lines indicate the average counts per cell of the blank barcodes, which provides an estimate of the false-positive rate.

The extraordinarily high cell density in the mid-gestation human fetal cortex posed a challenge for achieving precise single-cell resolution. To address this issue, we developed a custom deep-learning model based on the CellPose 2.0 framework^23,24^ to automate single-nucleus segmentation from nucleus-stained images captured during MERFISH imaging. The model achieved strong agreement with manual labelling (Extended Data Fig. 1a–c). A modest dilation of nuclei-based cell masks was applied, which enriched transcript counts for each cell without compromising the precision of cell identity (Extended Data Fig. 1d,e). The distribution of cell volume remained consistent across samples, experiments and clusters (Extended Data Fig. 1f–h), and gene-expression profiles from technical and biological replicates demonstrated high reproducibility (Extended Data Fig. 1i–k).

**Extended Data Fig. 2:**
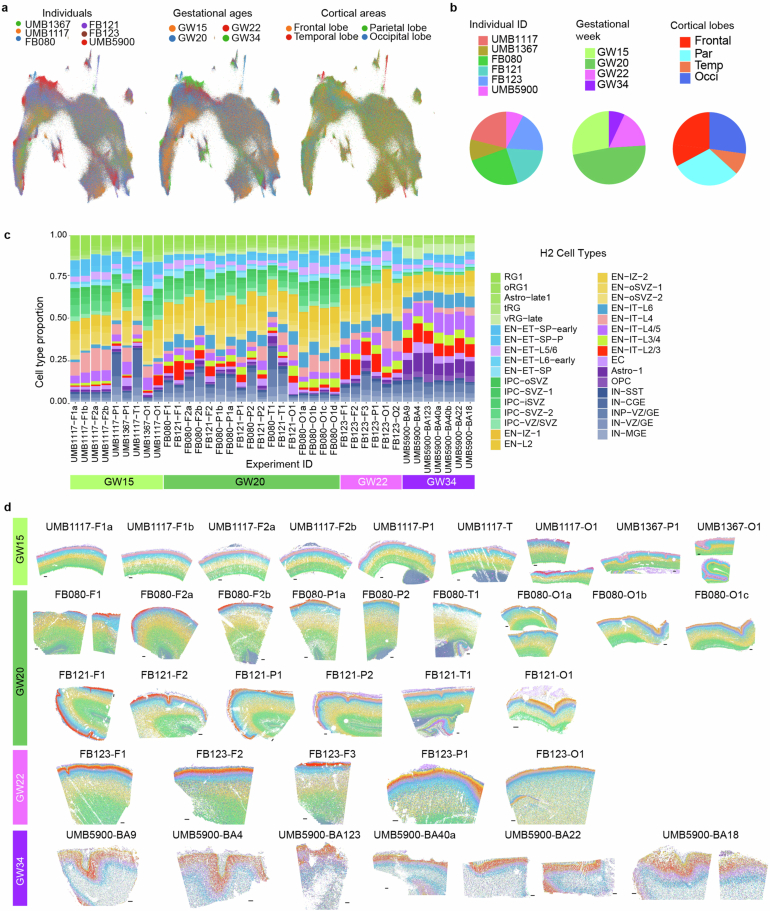
Robust integration of single cell data from various donors, ages, and areas. **a**, UMAP plots showing cells from different individuals (left), gestational ages (mid), and cortical lobes (right) integrated evenly into a combined dataset. **b**, Pie charts showing the proportion contributions of cells from different individuals (left), gestational ages (mid) and cortical lobes (right). **c**, Histogram showing the compositions of H2 cell types across MERFISH experiments. **d**, MERFISH spatial maps colored by H2 cell types. The experiment names are shown above each graph, which includes the sample ID, and a suffix indicating the cortical lobe. F, frontal; P, parietal; O, occipital; T, temporal; BA, Brodmann’s Area (only identifiable at GW34). Scale bars, 500 µm.

Initially, we analysed approximately 16 million single cells that met quality-control criteria with this gene panel, and we integrated these experiments to cluster the cells on the basis of their gene expression following a hierarchical strategy (Fig. 1a). An additional 2.6 million cells were analysed separately in a subsequent set of experiments and were not integrated with the initial analysis (Supplementary Table 2). At the first hierarchy, referred to as H1, we identified eight cell classes: radial glia (RG), intermediate progenitor cells (IPCs), migrating excitatory neurons (EN-Mig), intratelencephalic excitatory neurons (EN-ITs), extratelencephalic excitatory neurons (EN-ETs, including both corticothalamic and pyramidal tract neurons), inhibitory neurons (INs), other glia and endothelial cells (ECs) (Fig. 1b). At the second hierarchy (H2), these eight H1 cell classes were divided into 33 cell types, which were then subdivided into 114 subtypes at the third hierarchy (H3), 58 of which were excitatory neuron (EN) subtypes (Fig. 1c and Supplementary Table 5). Our sampling strategy ensured comprehensive representations of different cortical areas and gestational ages (Extended Data Fig. 2a–c).

**Extended Data Fig. 3:**
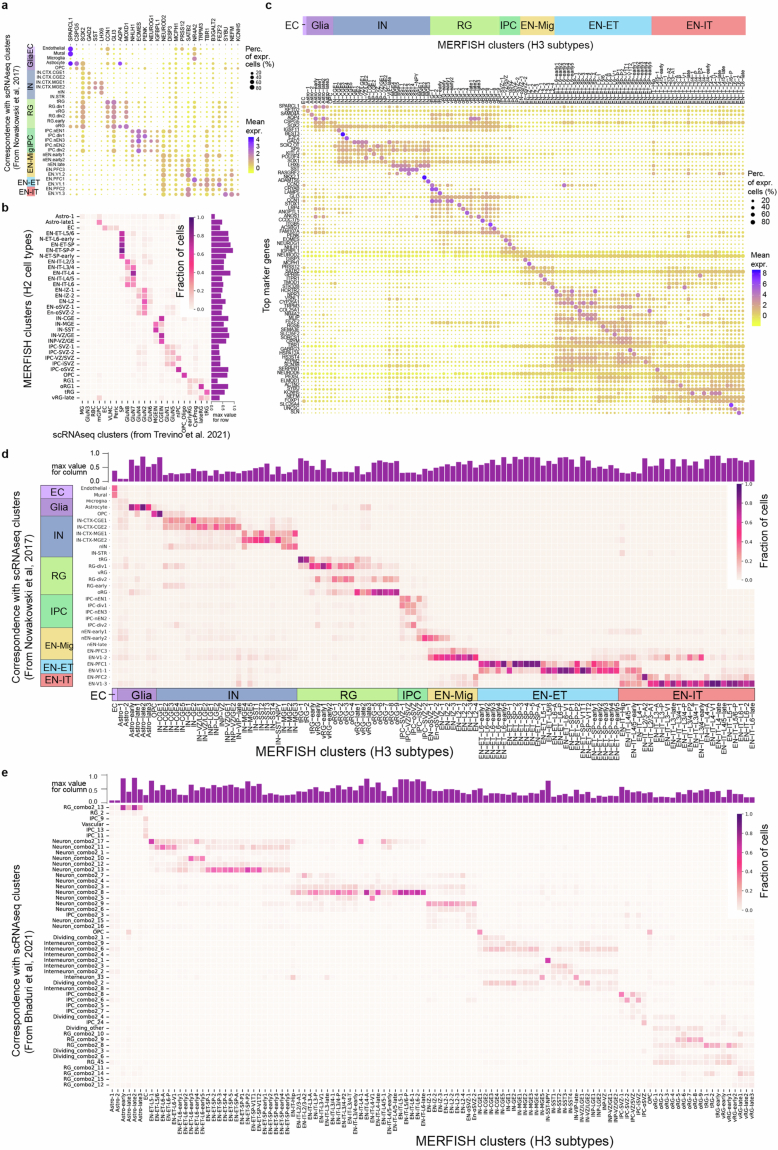
Gene expression profiles of MERFISH clusters and correspondence with scRNAseq datasets. **a**, Top markers genes for MERFISH cell types identified in Fig. 1e show similar expression patterns in the corresponding scRNAseq clusters identified in Fig. 1f. **b**, Cell type correspondence heatmap shows the fraction of cells from MERFISH H2 clusters that associate to clusters from another published mid-gestation scRNA-seq dataset^8^. The highest correspondence fraction for each MERFISH H2 cell type (largest value of each row) is shown in the histogram (right). **c**, Dot plot showing the expression of top marker genes for H3 subtypes. **d, e**, Cell type correspondence heatmaps showing the fraction of cells from MERFISH H3 clusters associated to clusters from published fetal scRNA-seq dataset from Nowakowski et al. 2017 (**d**)^7^ and from Bhaduri et al. 2021 (**e**)^6^. The highest correspondence fraction for each MERFISH H3 subtype (largest value of each column) is shown in the histogram (top).

**Extended Data Fig. 4:**
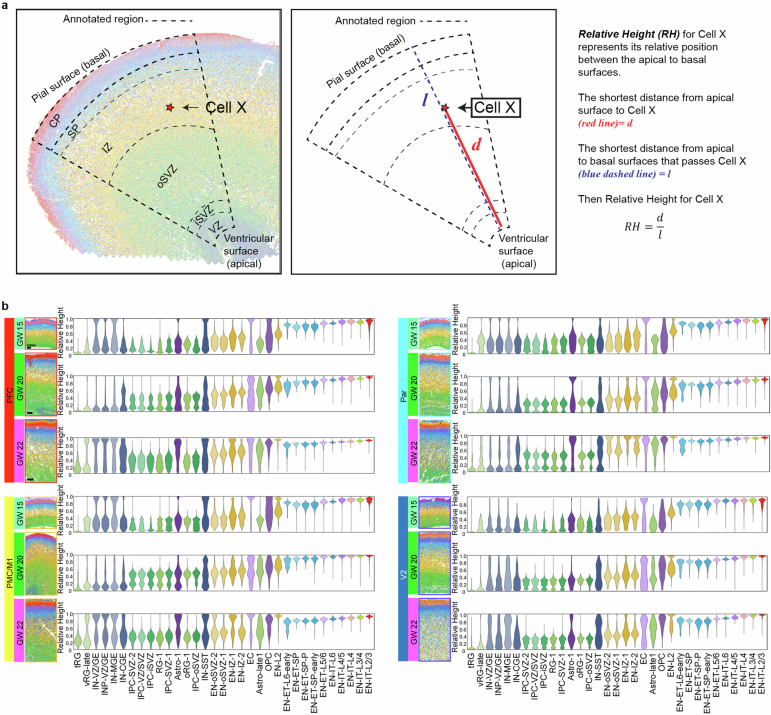
A framework for quantitative analysis of cells’ laminar localization. **a**, Schematics illustrating the annotated fan-shaped cortical area and the calculation of relative height (RH). **b**, Spatial maps and violin plots showing the laminar distribution of H2 cell types from the apical to basal surface quantified by the RH across major cortical areas in the second trimester. The width of the violin for each cell type is normalized to the maximum value. Image scales are the same for different areas from the same GW. Scale bars, 500 µm.

Clusters across all three hierarchical levels displayed distinct spatial distribution patterns. The localization of H2 cell types delineated the apical–basal laminar structures, including the ventricular zone (VZ), the inner subventricular zone (iSVZ), the outer subventricular zone (oSVZ), the intermediate zone (IZ), the subplate (SP), the cortical plate (CP) and the marginal zone (MZ) (Fig. 1d and Extended Data Fig. 2d). Clusters exhibited dynamic marker expression, and cell-label-transfer analysis showed close alignment to multiple fetal human cortex scRNA-seq datasets^6–8^ (Fig. 1e,f and Extended Data Fig. 3). To enable quantitative analysis of cell localization, we manually annotated the cytoarchitecture of each sample to create a framework in a fan-shaped region, which was then divided into major laminar structures based on the localization of H2 cell types (Methods). In this framework, each cell was assigned to a specific structure and we calculated its relative height (RH), which represented its normalized laminar position between the apical and basal surfaces (Extended Data Fig. 4a,b).

**Extended Data Fig. 5:**
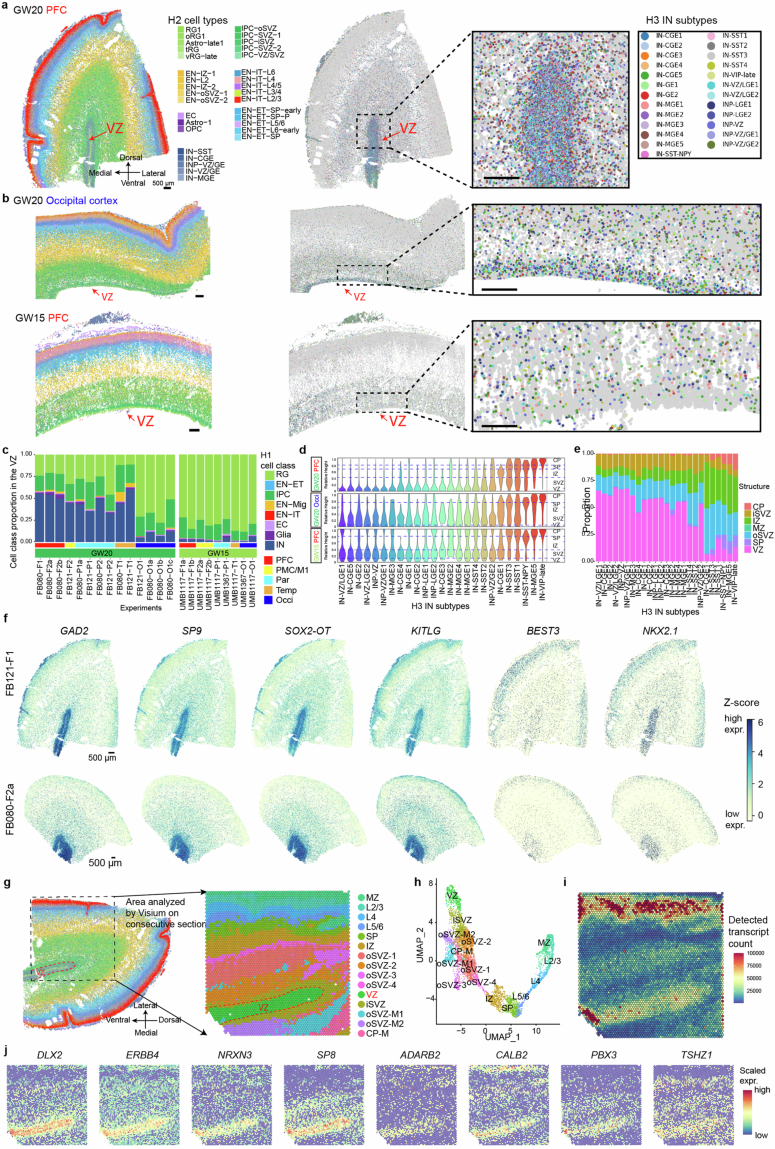
High concentration of INs in the dorsal VZ at mid-gestation. **a**, Spatial map shows inhibitory neurons (IN) are highly concentrated in the ventricular zone (VZ) of the prefrontal cortex (PFC) at GW20. **b**, INs are not enriched in the VZ of the occipital cortex at GW20, and are mostly absent in the VZ of all cortical areas at GW15. **c**, Histograms quantifying the cell class proportions within the VZ areas across experiments show INs outnumber RG and IPCs in all cortical areas except occipital cortex at GW20, but are largely absent at GW15. Due to the size restriction of MERFISH imaging area, the VZ was not systematically analyzed for GW22 and GW34 samples. **d, e**, Relative height (RH) violin plots and histograms for the distribution of H3 IN subtypes show the VZ localization is subtype dependent. **f**, Z-score spatial plots show IN markers are highly expressed in the VZ, but MGE progenitor marker *NKX2.1* and CGE progenitor marker *BEST3* were expressed sparsely. **g, h**, Spatial graph and UMAP of 10x Visium analysis of the PFC at GW20, performed on consecutive tissue section analyzed by MERFISH. Clusters for Visium spots were annotated based on tissue structure and gene expression. “M” in the cluster annotation means medial, as opposed to lateral. Capture area: 6.5 mm×6.5 mm. **i**, Spatial heatmap for detected transcript count in each spot. Because each spot has a diameter of 55 µm and contains variable number of cells, the variation in transcript count is largely due to difference in cell density in different structures. **j**, Scaled expression spatial heatmaps showing additional marker genes for various IN subtypes are highly expressed in the VZ, including olfactory bulb-like IN markers *CALB2*, *PBX3*, and *TSHZ1*. Scale bars, 500 µm.

We discovered a substantial concentration of INs and immature IN precursors (INPs) in the VZ of the dorsal forebrain during GW20–GW22, a phenomenon absent at GW15 (Extended Data Figs. 4b, 5a,b). By GW20, these IN populations outnumbered both RG and IPCs in the VZ across most cortical areas, except in the VZ of the occipital cortex, where INs were notably sparse (Extended Data Fig. 5c). The degree of VZ localization varied between IN subtypes, but VZ-enriched subtypes included those with transcriptional signatures resembling each of the three ganglionic eminence (GE) structures: the lateral ganglionic eminence (LGE), the caudal ganglionic eminence (CGE) and the medial ganglionic eminence (MGE) (Extended Data Fig. 5d,e). MERFISH and Visium analyses showed that marker genes of INs were expressed at high levels in the VZ, including those traditionally associated with olfactory bulb interneurons such as *CALB2*, *PBX3* and *TSHZ1* (ref. ^25^) (Extended Data Fig. 5f–j). The unexpected localization of these INs in the dorsal VZ raises the possibility that, contrary to being ventrally born and migrating into the cortex, they could include some dorsal-born INs reported in several recent studies^26–29^. Lineage-tracing analysis will be required to conclusively determine their origin.

## Progressive formation of cortical layers

**Extended Data Fig. 6:**
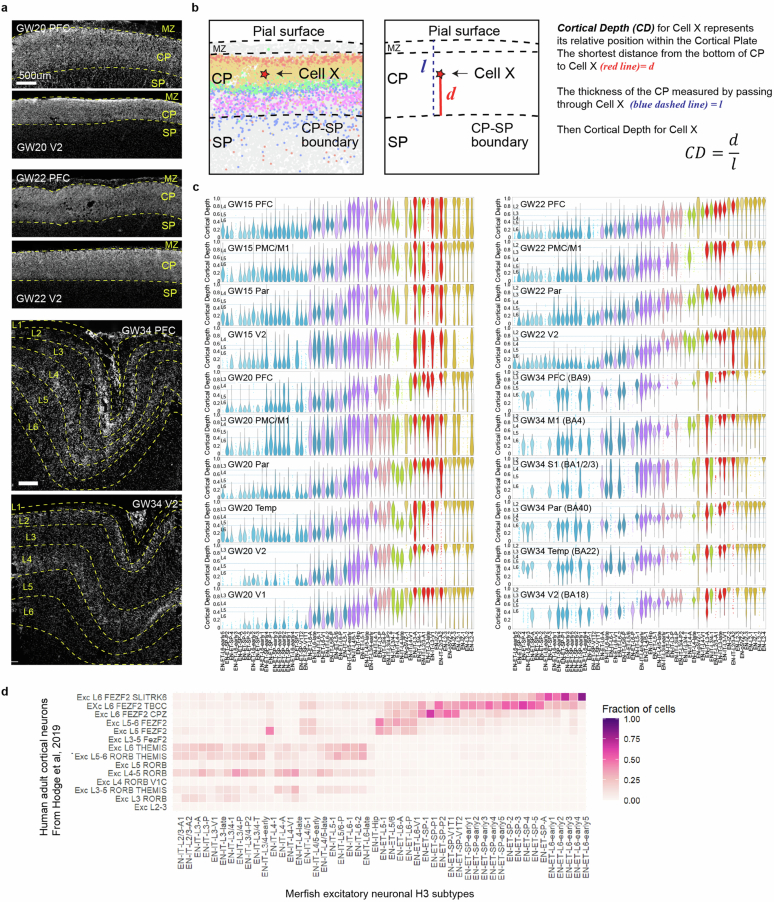
Cortical layer morphology and cortical depth analysis of neuronal subtypes. **a**, Microscopy images of nuclei staining (DAPI) for the CP of PFC and V2 at GW20, 22 and 34, demonstrating that cytoarchitectural difference between cortical layers is not visible until GW34. These images were co-captured during MERFISH imaging. Scale bars, 500 µm. **b**, Schematics illustrating the calculation of cortical depth (CD) within the CP. **c**, Violin plots showing the CD distribution of H3 EN subtypes across major cortical areas at GW15 to 34. **d**, Cell type correspondence heatmap shows the fraction of cells from H3 EN subtypes associated to neuronal cell types from published adult human cortex scRNA-seq dataset from Hodge et al.^35^.

The establishment of cortical layer structures during human fetal development has long been elusive owing to the lack of cytoarchitectural features and validated molecular markers^30–33^. Adult-like cytoarchitectural distinctions between layers were not present between GW15 and GW22, and became discernible only at GW34 (Extended Data Fig. 6a), a result consistent with previous reports^34^.

**Fig. 2:**
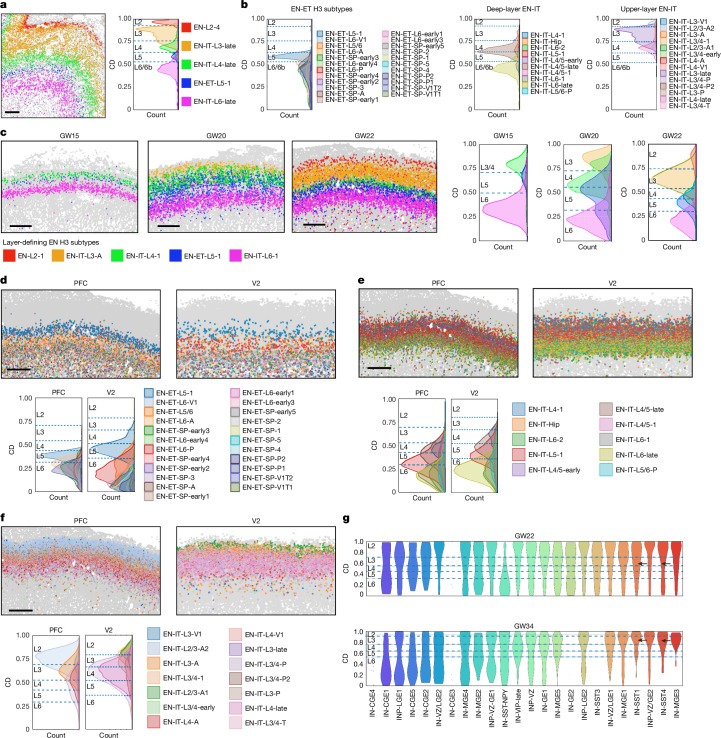
Progressive formation and specification of cortical layers. **a**, Left, spatial map showing that layers 2–6 (L2–L6) are defined by the organization of selected EN H3 subtypes in the PFC at GW34. Right, ridgeline plot showing the CD distribution of layer-defining EN subtypes. The height of the ridgeline represents the cell density. Dashed lines represent the border between layers, calculated on the basis of the CD distribution of layer-defining EN subtypes. **b**, Ridgeline plots reveal further spatial complexity among EN-ETs, deep-layer (layers 5 and 6) EN-ITs and upper-layer (layers 2–4) EN-ITs in the PFC at GW34. **c**, Spatial maps and ridgeline plots show the progressive formation of cortical layers from GW15 to GW22. The six-layer structure can be visualized and quantitatively defined by the distribution of layer-defining EN subtypes at GW22 despite the lack of cytoarchitectural differences between layers. **d**–**f**, Spatial maps (top) and ridgeline plots (bottom) showing the distribution of EN-ETs (**d**), deep-layer EN-ITs (**e**) and upper-layer EN-ITs (**f**) in the PFC and the V2 at GW22. Although many clusters exhibit area-dependent abundance, their laminar localization is highly consistent between the PFC and the V2. **g**, Violin plots showing the laminar distribution of H3 IN subtypes in the CP of the PFC at GW22 and GW34. IN-SST clusters indicated by the arrows exhibit specific localization in layers 2 and 3 at GW34. Scale bars, 500 µm (**a**,**c**,**d**,**f**).

MERFISH analyses revealed that most EN subtypes exhibited layer-dependent distribution in the CP and the SP. To precisely analyse the cortical layer distribution of H3 EN subtypes, we measured their relative laminar position in the CP as cortical depth (CD) (Methods and Extended Data Fig. 6b). For individual H3 EN subtypes, laminar distribution was highly stable across cortical areas and gestational ages, with gradual refinement in layer specificity over time, which was particularly evident when comparing the GW34 distribution to earlier time points (Extended Data Fig. 6c). We identified a subset of narrowly dispersed subtypes that exhibited distribution patterns that aligned with one of the six morphologically defined cortical layers at GW34 (Fig. 2a). Using their CD distribution, we quantitatively defined the boundaries between layers for each area, which enabled us to assign individual cells to specific layers (Methods). EN clusters were annotated for their predominant layer localization at GW34, a classification supported by a comparison with human adult cortex data^35^ (Extended Data Fig. 6d).

**Extended Data Fig. 7:**
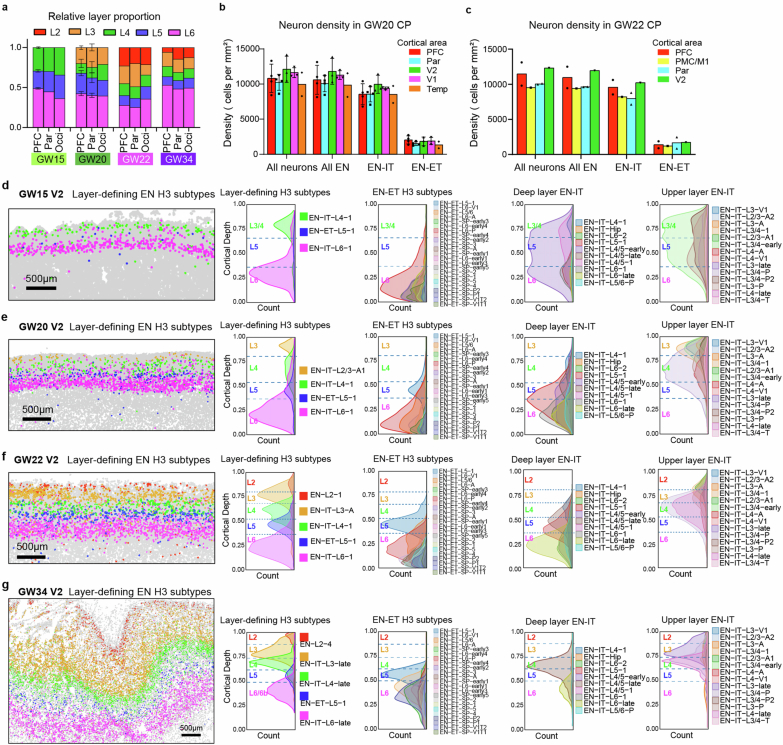
Additional characterizations of cortical layers and EN laminar distribution. **a**, Histogram showing the relative proportion of each cortical layer at GW15 to 34 in the PFC (n = 10 independent experiments), parietal cortex (Par) (n = 8 independent experiments), and occipital cortex (Occi) (n = 5 independent experiments). Average is taken for replicate experiments; histograms represent mean ± standard deviation when applicable. **b, c**, Histograms for cell and neuron density across major cortical areas at GW20 (**b**, n = 17 independent experiments) and GW22 (**c**, n = 6 independent experiments). Replicate data points are shown when applicable; histograms represent mean ± standard deviation. **d-g**, Spatial maps of layer-defining EN subtypes, and ridgeline plots showing the laminar distribution of H3 EN subtypes in the V2 at GW15 (**d**), 20 (**e**), 22 (**f**) and 34 (**g**). Scale bars, 500 µm.

In marked contrast to conventional characterizations, the distribution of layer-defining H3 EN subtypes clearly delineated all six cortical layers at GW22, despite the absence of morphological distinctions (Fig. 2c and Extended Data Fig. 7a). Cortical layers evolved progressively over time, with layers 4–6 visible at GW15 and layers 3–6 at GW20, a result consistent with the inside–out sequence of cortical layer formation and proposed corticogenesis timeline^2,10,36,37^ (Fig. 2c). This observation demonstrates that human cortical layers are molecularly defined by neuronal subtypes nearly simultaneously with the construction of the layers, months before the emergence of cytoarchitectural differences between the layers. Unlike EN-ETs, EN-IT subtypes often spanned multiple layers and exhibited distinct bell-shaped distribution curves irrespective of layer boundaries, which resulted in extensive laminar intermixing (Fig. 2e,f and Extended Data Fig. 7). This complexity persisted at GW34, despite the emergence of distinct cytoarchitectures (Fig. 2b), which mirrored observations in the adult cortex^35^.

We extended our analysis to quantify the CD distribution of IN subtypes. Cortical layer specificity was not evident during GW15–GW22, which suggested that layer-dependent IN organization had not been established at mid-gestation (Fig. 2g). Nevertheless, some layer specificity began to emerge at GW34, particularly among somatostatin (SST) INs, which primarily localized in layers 2 and 3, consistent with observations in mouse and human adult cortices^35^.

## Dynamic laminar gene expression

**Fig. 3:**
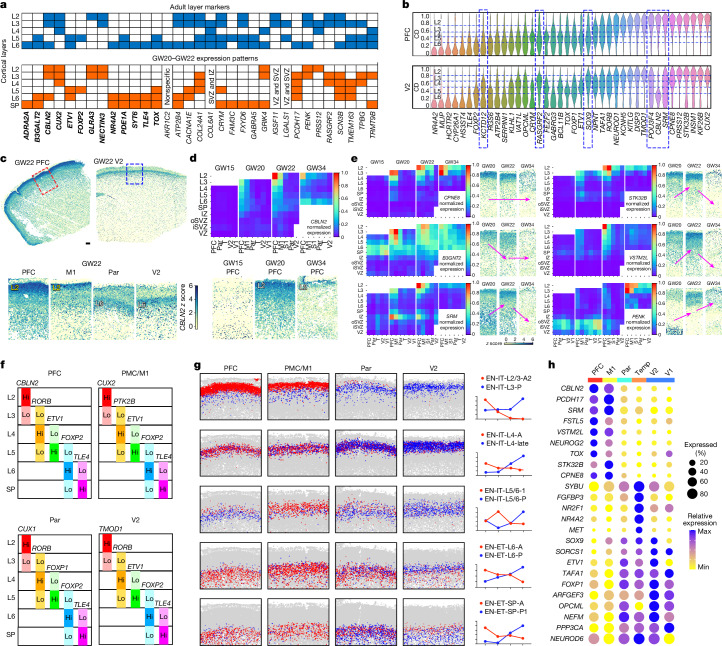
Areally dynamic gene expression and continuous neuron specification along the AP axis. **a**, Table comparing the laminar expression patterns of classical cortical layer markers between the adult human PFC (data from ref. ^38^) and mid-gestation PFC (this study). Genes that show a conserved laminar expression pattern are highlighted in bold. **b**, Violin plots show the laminar expression patterns of layer-dependent genes in the CP of the PFC and the V2 at GW22. The width of the violins represent the cumulative normalized expression in the cells located at a CD. Genes that are enriched in different layers between the PFC and the V2 are highlighted by the dashed outline boxes. **c**,**d**, *z* score spatial maps (**c**) and summary expression heatmap (**d**) showing the spatiotemporal expression pattern of *CBLN2*. The summary heatmap shows different cortical layers and laminar structures from top to bottom as rows, and cortical areas and gestational ages as columns, organized from anterior to posterior and from young to old. The average is taken for replicate samples from the same area and gestational week. T, temporal cortex. Scale bar, 500 µm. **e**, Summary expression heatmaps and *z* score spatial maps of genes identified with layers 2 and 3 enrichments in the PFC. Arrows indicate the direction of upregulation or downregulation over time. **f**, Schematic summary of the combinations of five marker genes that enable the approximation of cortical layers of different cortical areas at GW22. **g**, Spatial graphs and normalized curve plots showing the localization and abundance of pairs of anterior-enriched and posterior-enriched EN subtypes at GW22 across major cortical areas. **h**, Dot plot showing the expression of top areally enriched genes at GW20 and GW22 in all post-migratory ENs (EN-IT and EN-ET cell classes).

**Extended Data Fig. 8:**
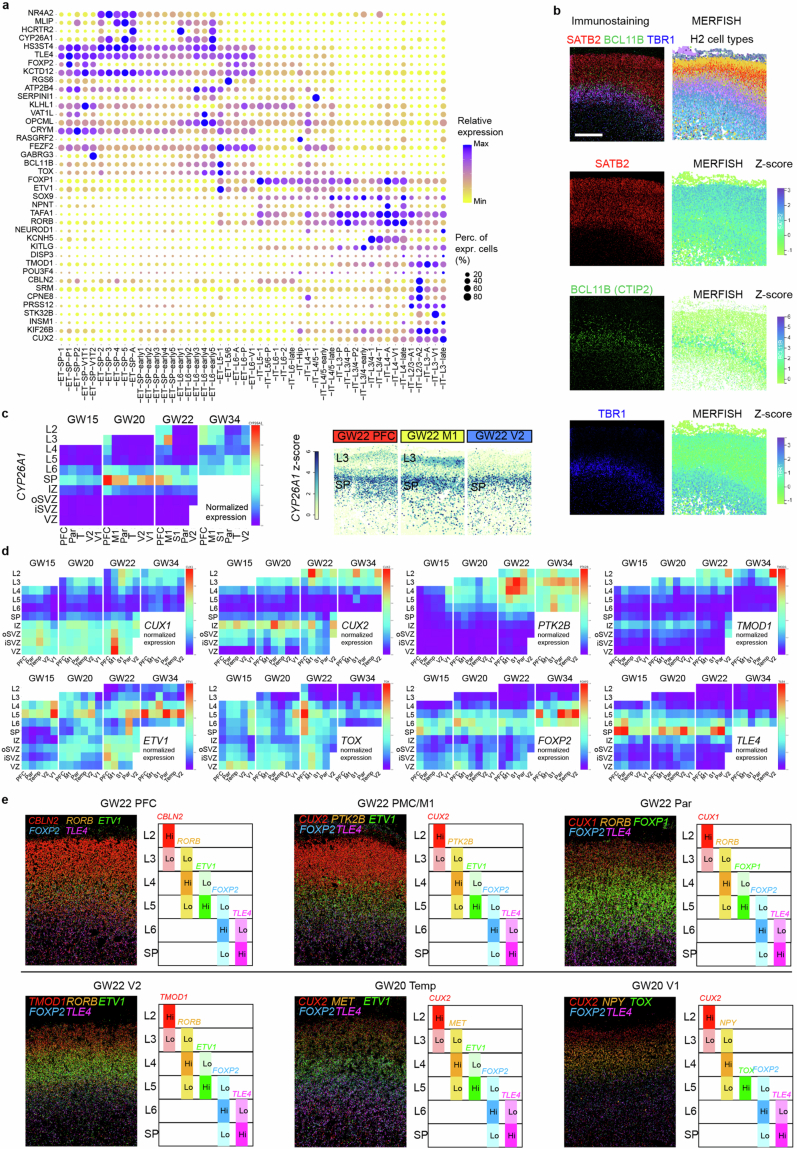
Laminar expression patterns of genes are dynamic across ages and areas. **a**, Dot plots showing the expression of genes exhibiting cortical layer-dependent distribution from Fig. 3b across H3 EN subtypes. **b**, Z-score spatial maps (right) from MERFISH experiments on the parietal cortex at GW22, and matching confocal microscopy images (left) of immunostaining for SATB2, CTIP2 (gene name *BCL11B*), and TBR1. The immunostaining was performed on a tissue section adjacent to the one analyzed by MERFISH, and the same area is shown, demonstrating high spatial correspondence between mRNA detection by MERFISH and protein detection by immunohistology (not repeated). Scale bar, 500 µm. **c**, Summary expression heatmap and z-score spatial maps for *CYP26A1*. **d**, Summary expression heatmaps showing the spatial temporal expression patterns of area-specific cortical layer markers listed in Fig. 3j. **e**, Pseudo-colored images of detected transcript for area-specific cortical layer marker combinations identified by MERFISH.

Because canonical marker genes identified in the adult human cortex and in mice exhibit reduced laminar specificity in the fetal cortex, we sought to identify genes that could label cortical layers during mid-gestation^7,31^. Building on our CD framework, we assessed the laminar expression patterns of marker genes identified in the adult human cortex^38^ and observed substantial divergence (Fig. 3a). Notably, genes associated with EN-ETs showed greater conservation of expression patterns than those for EN-ITs. Next, we identified genes with layer-dependent enrichment and quantified their laminar expression in the CP (Fig. 3b and Extended Data Fig. 8a). Immunostaining for selected markers using validated antibodies showed concordance of mRNA localization detected by MERFISH with protein expression (Extended Data Fig. 8b).

Notably, some genes displayed laminar expression patterns that varied significantly with gestational age and cortical area. For example, *CBLN2*, a synaptic organizer with a hominin-specific variant in a retinoid-acid-responsive enhancer^39^, displayed strong frontal enrichment in layers 2 and 3 at GW22–GW34. Moreover, low-level expression in layer 6 was maintained across all cortical areas throughout GW15–GW34 (Fig. 3c,d). Similarly, *CYP26A1*, a retinoic-acid-metabolizing enzyme^13^, manifested frontal-specific expression in layers 2 and 3 and remained highly expressed in EN-ETs in layer 6 and the SP across all areas (Extended Data Fig. 8c). Moreover, a group of genes demonstrated substantial frontal enrichment in layers 2 and 3, but their peak expression timing varied (Fig. 3e), which suggested that the frontal specification of layers 2 and 3 had undergone temporally dynamic transcriptional changes. For visualization, we compiled the expression pattern for each of the 300 genes in our MERFISH panel into a structured heatmap (Supplementary Data).

Because most genes spanned across multiple layers at mid-gestation, pinpointing a singular gene as a definitive marker for any specific cortical layer is improbable. Instead, we curated context-aware marker sets to facilitate the approximate visualization of mid-gestation cortical layers using conventional methods, such as multichannel in situ hybridization (Fig. 3f). During GW20–GW22, *FOXP2* and *TLE4* labelled layer 6 and the SP, respectively, in all cortical areas. *ETV1* marked layer 5 in the prefrontal cortex (PFC), the premotor cortex (PMC), the primary motor cortex (M1), the V2 and the temporal cortex (Temp), whereas *FOXP1* and *TOX* marked layer 5 in the parietal cortex (Par) and V1, respectively. Markers for layers 2–4 showed increasing areal differences, including *CBLN2*, *CUX2*, *CUX1*, *TMOD1*, *RORB*, *PTK2B*, *MET* and *NPY* (Extended Data Fig. 8d,e).

## Areal specification of neuronal subtypes

**Extended Data Fig. 9:**
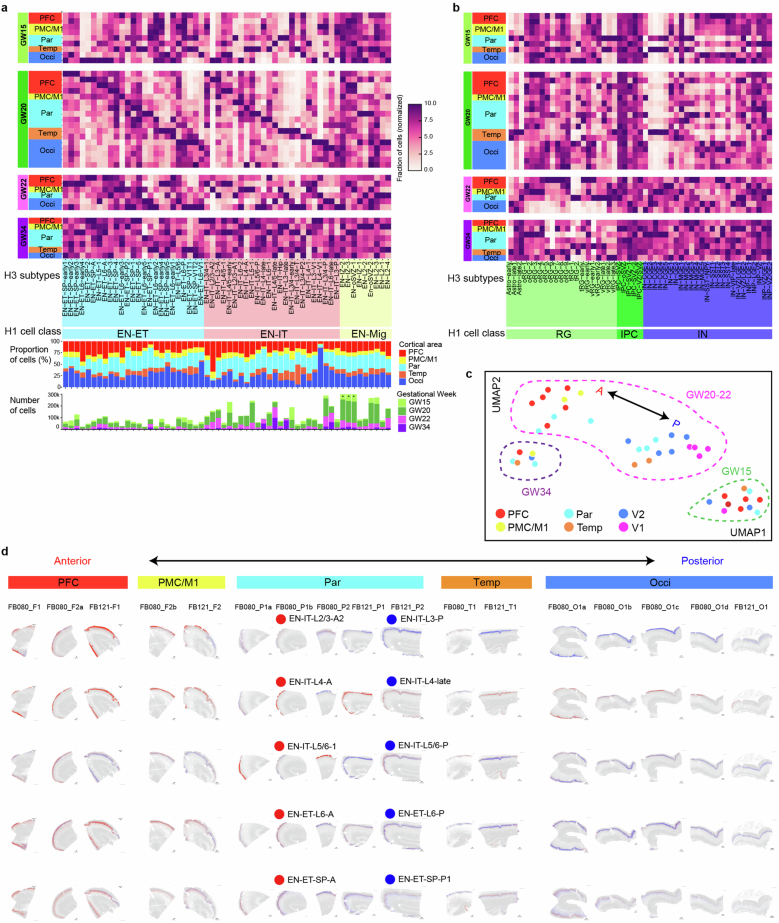
Anteriorly and posteriorly enriched neuronal subtypes showcase continuous cortical arealization in the mid-gestation. **a**, Top, enrichment heatmap showing the normalized fraction of cells from each H3 EN subtype that came from a given age and cortical area. Each row represents an independent MERFISH experiment, and each column represents an EN subtype under EN-IT, EN-ET, and EN-Mig. For each column the number of cells from each experiment was normalized to the total number of the cluster, and then normalized to the maximum value of the column within the same gestational week to show relative enrichment (Methods). Bottom row 1, histogram showing the contribution of major cortical areas towards each H3 EN subtype. Bottom row 2, histogram showing the number of cells and contribution from each gestational age for H3 EN subtypes. Three columns that have values exceeding 300k cells are indicated with an asterisk. **b**, Heatmap for areal enrichment of RG, IPC, and IN H3 subtypes, similar to **a**. Parts of the CGE were included in the temporal lobe samples for GW15 and 20, leading to strong enrichment of several IN-CGE subtypes. **c**, UMAP plot of EN subtype composition for individual experiments shows a continuous anterior-posterior spectrum for GW20-22. **d**, Arrays of spatial maps of GW20 MERFISH experiments, showing the pairs of EN clusters in Fig. 3g exhibit similar gradient-like enrichment patterns in the A-P axis.

Next, we analysed the area-dependent specification of neuronal subtypes in each cortical layer. Although the distribution of H2 EN cell types remained largely uniform along the AP axis, many H3 EN subtypes (7 out of 25 EN-ET H3 subtypes and 15 out of 24 EN-IT subtypes) showed gradual enrichment in either the anterior or the posterior regions and formed continuous gradient-like distribution patterns (Fig. 3g and Extended Data Fig. 9a). By contrast, subtypes of EN-Mig cells, RG and IPCs did not display discernible areal preference (Extended Data Fig. 9b). EN-IT and EN-ET subtype compositions revealed clear distinctions between major cortical areas at GW20 and GW22, forming a continuous AP spectrum on uniform manifold approximation and projection (UMAP) plots (Extended Data Fig. 9c).

**Extended Data Fig. 10:**
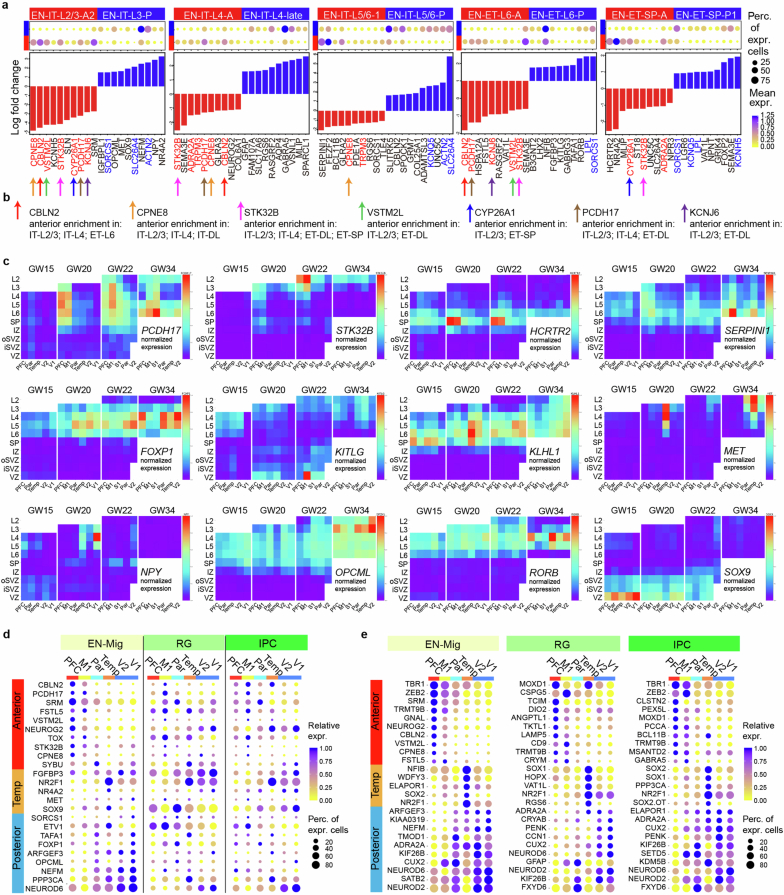
Identification of genes driving neuronal areal specification along the A-P axis. **a, b**, Dot plots and histograms of fold change for top differentially expressed genes (DEGs) between pairs of anterior- and posterior-enriched EN subtypes for each cortical layer at GW22. Genes that appear repeatedly are highlighted in red (for anterior-enriched) or in blue (for posterior-enriched). **b** summarizes several prominent anteriorly enriched genes seen across different pairs. **c**, Summary expression heatmaps of genes that are anteriorly or posteriorly enriched consistently across all layer categories in Fig. 3h. **d**, Dot plots for EN-Mig, RG, and IPC at GW20 showing the expression of areally enriched genes identified in EN-IT and EN-ETs in Fig. 3h. EN-Mig, but not RG and IPC, exhibited similar areal enrichment patterns as EN-IT and EN-ET. **e**, Dot plot showing the expression of top areally-enriched genes at GW20 identified separately among EN-Mig, RG, and IPC.

We selected five pairs of subtypes with opposing AP distributions from five categories of neurons: layers 2 and 3, layer 4, deep-layer EN-ITs, deep-layer EN-ETs and SP EN-ETs (Fig. 3g and Extended Data Fig. 9d). The two clusters in each pair were transcriptionally similar; therefore, their differentially expressed genes (DEGs) were likely to have crucial roles in areal specification. Although the lists of top anteriorly and posteriorly enriched DEGs were distinct across the five categories (Extended Data Fig. 10a), several genes with strong anterior enrichment, such as *CBLN2*, *CPNE8*, *VSTML2*, *STK32B*, *PCDH17* and *CYP26A1*, frequently emerged as top DEGs in multiple categories (Extended Data Fig. 10a,b and Supplementary Table 6). This result suggests that although area-dependent neuronal specification is regulated in a layer-specific and subtype-specific manner, the genes that orchestrate this process are versatile and often shared.

When we analysed all significant DEGs (adjusted *P* < 0.05, log[fold change] > 0.5), we identified four genes enriched in anterior regions and eight in posterior regions across all five neuronal categories. These genes also showed strong anterior or posterior enrichment in EN populations as a whole, which indicated their role as markers of cortical areal identity (Fig. 3h and Extended Data Fig. 10c). Their areal enrichment patterns varied in other cell classes, limited among RG and IPCs, but were notably consistent in EN-Mig cells (Extended Data Fig. 10d,e), which indicated that areal identities are dynamically shaped during and after radial migration.

## Neuronal subtype diversification over time

To investigate EN subtype specification over time, we implemented an alternative approach by clustering cells from each gestational age independently rather than combining them. Using the single-cell significant hierarchical clustering (scSHC) pipeline^40^, we determined the number of EN subclusters that achieved transcriptional differences above a fixed significance threshold between clusters (Methods). In the EN-ET and EN-IT cell classes, the number of distinct scSHC clusters increased over gestational time, as the analysis produced 16, 39, 40 and 62 subclusters at GW15, GW20, GW22 and GW34, respectively (Supplementary Table 7). To differentiate these clusters from those identified through integrated analysis, an apostrophe (’) precedes the names of scSHC clusters.

**Extended Data Fig. 11:**
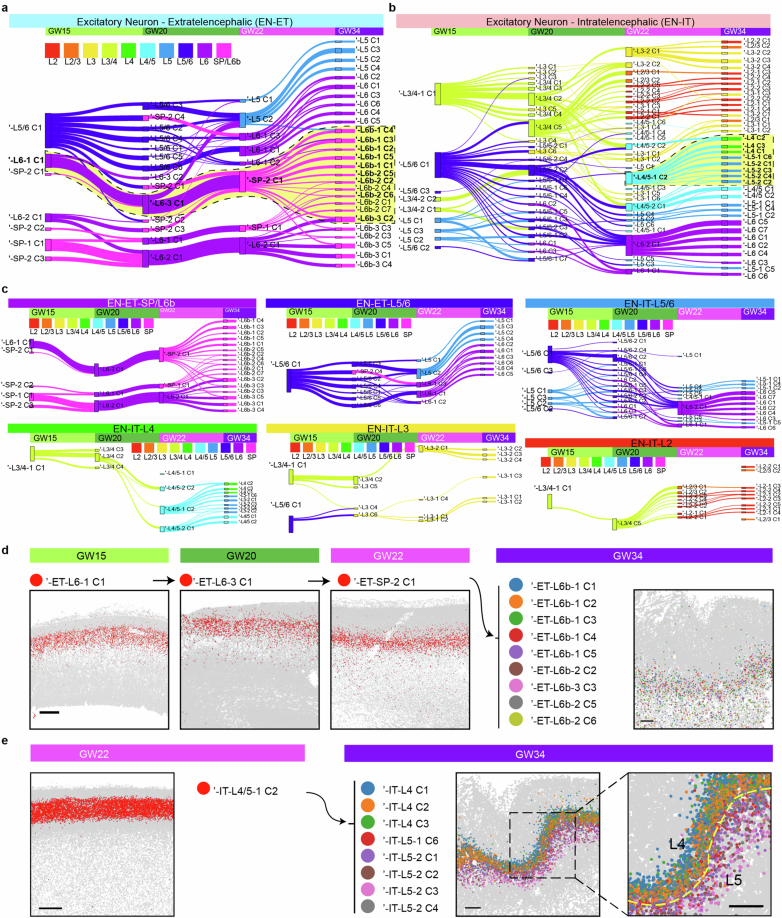
Diversification of EN subtypes over time shows continued post-mitotic fate specification. **a, b**, Sankey diagrams showing the correspondence between EN-ET (**a**) and EN-IT (**b**) subtypes from different gestational time points. Clustering was performed separately for each gestational age and the number of subclusters was determined by single cell significant hierarchical clustering (scSHC)^40^. To differentiate these clusters from those identified through integrated analysis, an apostrophe (’) precedes the names of scSHC clusters. Nodes represent EN subtypes from this alternative clustering strategy. Thickness of edges represents the fraction of cells showing correspondence, and edges with <0.1 fraction were hidden. Nodes and edges are colored based on the most enriched layer identity of the EN subtype. Two inferred lineages shown in **c** and **d** are highlighted with yellow background. **c**, Sankey diagrams similar to **a** and **b**, with layer-based groups shown separately from each other. **d**, Spatial graphs for an inferred lineage (highlighted in **a**) of Layer 6/SP EN-ETs across GW15 to GW34 show a conserved stream from GW15 to 22 before dramatic diversification at GW34. Scale bars, 500 µm. **e**, Spatial graphs showing a GW22 cluster EN-ITs spamming both Layers 4 and 5 specifying into seven GW34 clusters with refined layer specificity in either Layer 4 or 5 (highlighted in **b**). Dashed line shows the border between Layers 4 and 5. Scale bars, 500 µm.

To infer EN lineage relationships, we applied a deep-learning cell-type classifier to predict the probable origin of clusters at the earlier time^41^ (Methods). The resulting correspondence flows uncovered continued diversification that was particularly notable between GW22 and GW34. This result aligns with the anticipated role of the third trimester in neuronal-subtype specification^42^ (Extended Data Fig. 11a–c). For instance, ’EN-ET-L6-2-Cluster1 at GW15 corresponded strongly with a single cluster at GW20 and GW22, before branching into nine layer 6b EN-ET clusters by GW34 (Extended Data Fig. 11d). Third-trimester specification also led to the refinement of layer specificity, as exemplified by the ’EN-IT-L4/5-1-Cluster2 from GW22 diverging into five layer 5 subtypes and three layer 4 subtypes by GW34 (Extended Data Fig. 11e).

**Extended Data Fig. 12:**
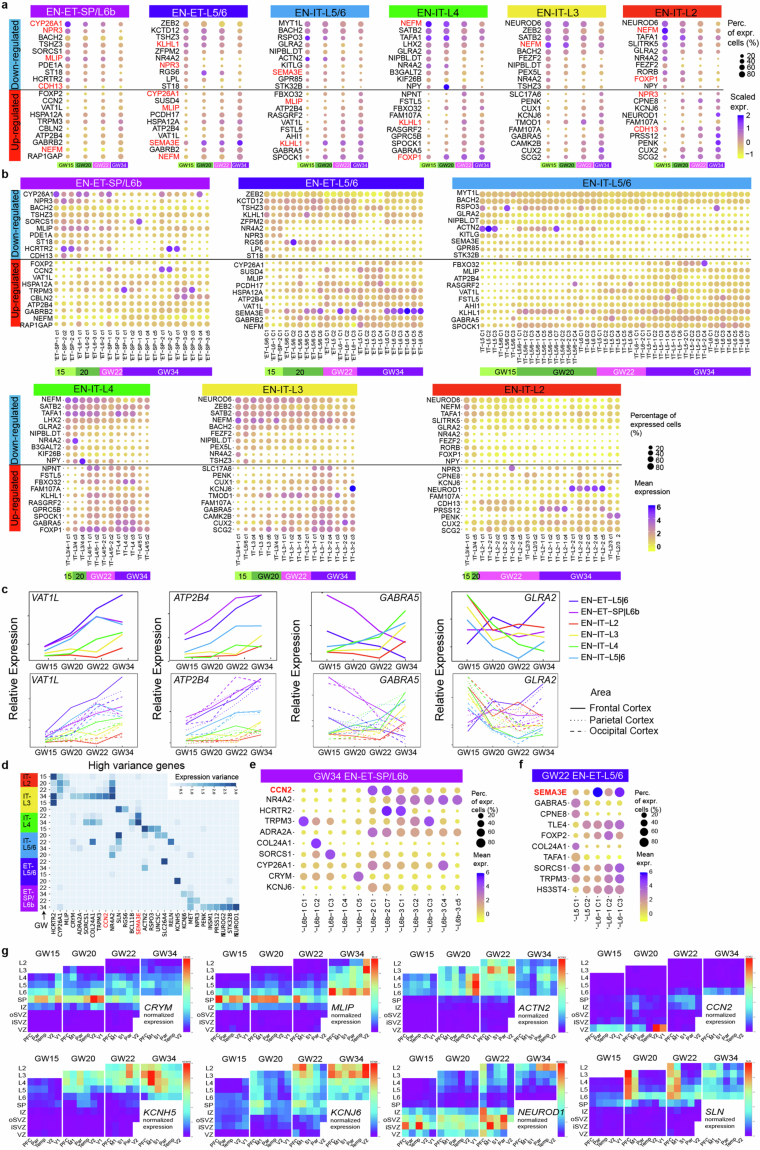
Dynamic gene expression underlies diversification of EN subtypes. **a**, Dot plots showing the top genes that are down- or up-regulated with gestational age among EN subtypes from each layer-based group. The genes that are downregulated over time in some categories but upregulated in others are highlighted in red. **b**, Dot plots similar to **a**, but showing the expression of these genes in individual EN subtypes. **c**, Curve plots showing the change of scaled expression over time in different EN groups and cortical lobes for selected genes. Top, across all cortical areas; bottom, showing each cortical lobe separately: data from PFC, PMC and M1 are averaged for frontal cortex; V1 and V2 are averaged for occipital cortex. **d**, Heatmap showing the genes with high expression variance among EN subtypes within the same group and gestational age. **e, f**, Dot plots showing the expression of high variance genes among EN subtypes in EN-ET-SP/L6b group at GW34 (**g**) and EN-ET-L5/6 group at GW22 (**h**). **g**, Summary expression heatmaps for high variance genes among EN subtypes display distinct laminar and areal enrichment.

We next identified genes upregulated or downregulated across gestational ages in EN subtypes grouped by correspondence flows (Extended Data Fig. 12a,b). Among the top DEGs, those associated with the general functional maturation of neurons, such as *VAT1L* (vesicle amine transport protein 1 homolog) and *ATP2B4* (ATPase plasma membrane Ca2+ transporting 4), showed synchronous regulation across all layers. By contrast, genes that exhibited upregulation in specific groups but downregulated in others often encoded specific neurotransmitter receptors such as *GABRA5* (GABA receptor subunit α5) and *GLRA2* (glycine receptor α2). This result suggests that transcriptional changes stem from differential functional specification (Extended Data Fig. 12c).

To identify genes that potentially drive subtype specification, we focused on those with the highest variance between EN subtypes in the same group and gestational age, many of which demonstrated specific laminar and areal enrichment (Extended Data Fig. 12d,g). For example, *CCN2* (also known as *CTGF*) emerged as one of the genes with the highest variance among EN-ETs in the SP and layer 6b specifically at GW34 (Extended Data Fig. 12e). Given the known role of *CCN2* in differentiating between upright pyramidal neurons (PNs) and other PNs in layer 6b, its upregulation in specific EN subtypes could indicate the commencement of PN subtype specification^43^. Conversely, *SEMA3E*, which is crucial for specifying a subset of layer 5 PNs projecting to higher-order thalamic and pontine nuclei^44^, showed high variance among EN-ET layer 5 and 6 subtypes as early as GW22 (Extended Data Fig. 12f).

## A sharp transcriptional border between V1 and V2

**Fig. 4:**
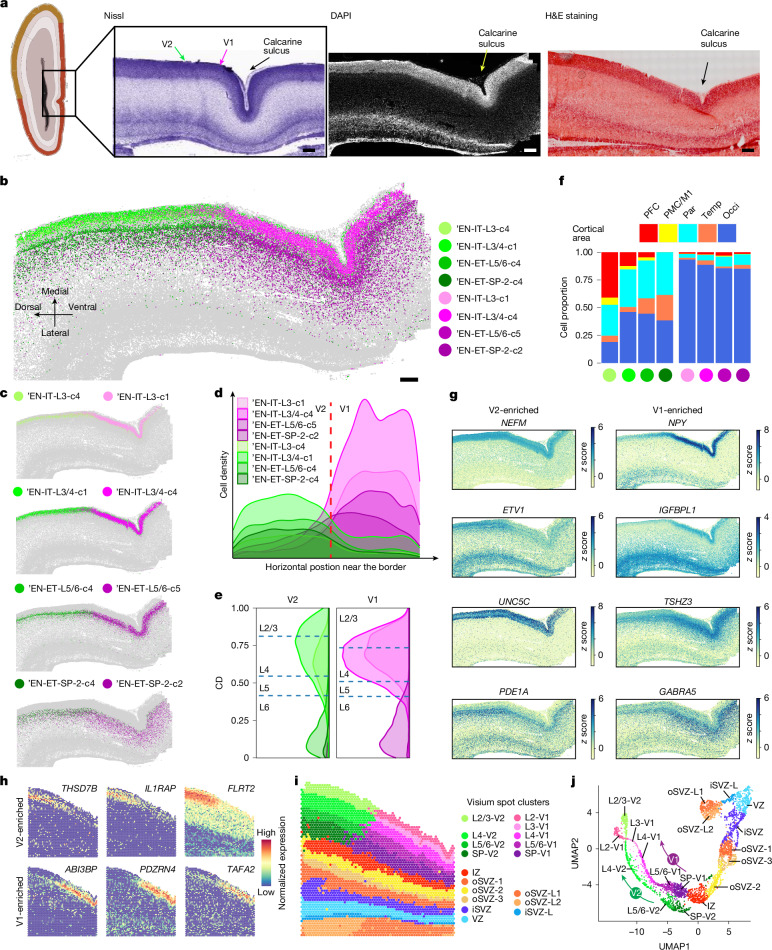
Sharp molecular border between the V1 and V2 reveals discrete arealization as early as GW20. **a**, The V1 and V2 do not exhibit morphological differences at GW20–GW21. Schematics and Nissl staining are taken from the Allen Reference Atlas for GW21 human fetal brain^63^. DAPI staining image of a GW20 occipital cortex was co-captured with MERFISH (repeated independently for four times). Haematoxylin and eosin (H&E) staining image of a GW20 occipital cortex (consecutive section) was co-captured with Visium (not repeated). Scale bars, 500 µm. **b**,**c**, Spatial maps for selected EN subtypes in the occipital cortex show a distinct border between the V1 and V2 marked by a sharp transition of EN subtypes at GW20. Scale bar, 500 µm. **d**, Ridgeline plots of the horizontal cell density profile for V1-enriched and V2-enriched EN subtypes in the CP near the border. **e**, Ridgeline plots of the laminar distribution for V1-enriched and V2-enriched EN subtypes. Dashed lines represent the borders between cortical layers. **f**, Histograms for areal distribution show V2-enriched subtypes (green) distributed broadly in other cortical areas, whereas V1-enriched subtypes (purple) are exclusive to the occipital cortex. **g**. *z* score spatial maps of representative V1-enriched and V2-enriched genes. **h**, Spatial graphs showing additional genes identified by Visium exhibiting a clear transition at the border. **i**, Spatial graph from Visium analyses show a clear V1–V2 border across all cortical layers. Capture area, 6.5 × 6.5 mm. **j**, UMAP of Visium spots shows that the trajectory remains coherent from the VZ to the IZ, but bifurcates after the SP to form two trajectories representing the V1 and the V2.

**Extended Data Fig. 13:**
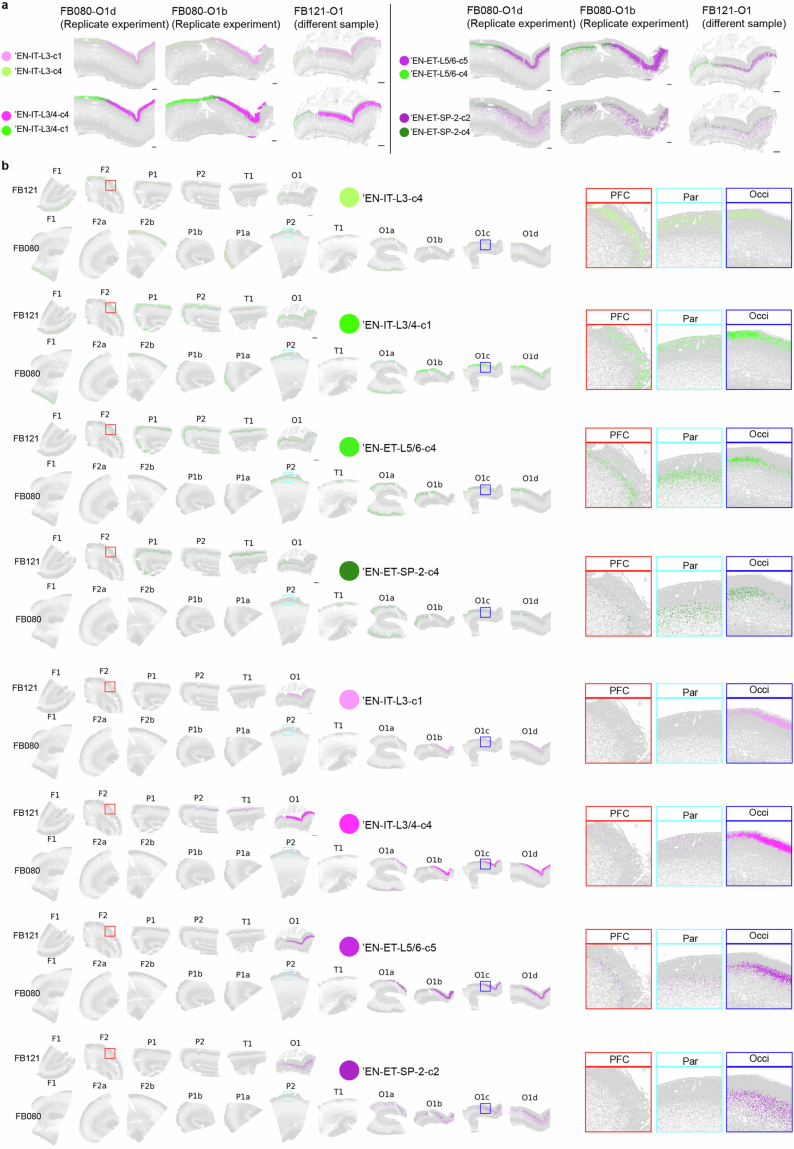
V1-specific subtypes are exclusive, in contrast to more continuous arealization patterns elsewhere. **a**, The delineation of sharp V1-V2 border by neuronal subtypes at GW20 is consistent across technical and biological replicates. Scale bars, 500 µm. **b**, Arrays of spatial maps of GW20 MERFISH experiments, showing V2-enriched clusters (in green) are broadly distributed in other cortical areas, whereas V1-enriched clusters (in purple) are found exclusively in the V1. Right, magnified views of the indicated regions.

To investigate the precise differentiation of adjacent cortical areas, we focused on the V1 and V2. Although the V1 and V2 in the adult cortex are distinguished by a prominent morphological border^3,45^, they appeared completely homogeneous in mid-gestation (Fig. 4a). Notably, MERFISH analyses of GW20 samples revealed a clear border between the V1 and V2 that was defined by four opposing pairs of EN subtypes (Fig. 4b,c and Extended Data Fig. 13a). A swift transition was seen across all cortical layers and the SP, with the most abrupt shift occurring in layers 3 and 4 (Fig. 4d). The two subtypes in each pair originated from the same H2 cell type and shared identical laminar distributions (Fig. 4c,e). V2-enriched subtypes were broadly distributed in other cortical areas, with some presence even in anterior areas. This result aligns with the continuous gradient-like pattern along the AP axis described above. By contrast, V1-specific EN subtypes were exclusively found in the V1 and absent elsewhere. This result suggests that a highly localized program, distinct from continuous AP patterning, may underlie V1 specification, thereby setting the V1 apart as an exception from other areas analysed (Fig. 4f and Extended Data Fig. 13b).

**Extended Data Fig. 14:**
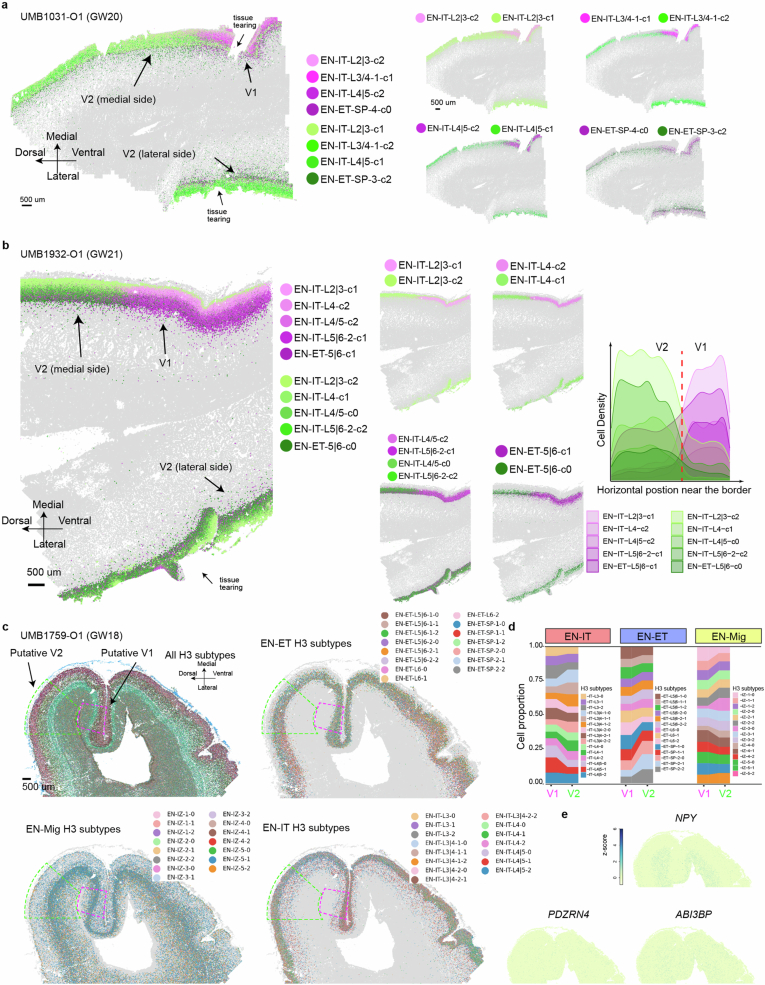
Sharp V1-V2 border is seen in additional samples from GW20-21, but not GW18. **a, b**, Spatial maps for selected EN subtypes show distinct border between V1 and V2 marked by sharp transition of EN subtypes at GW20 (**a**) and GW21 (**b**). Scale bars 500 µm. Right, ridgeline plots showing the horizontal cell density profile for V1- and V2- enriched EN subtypes in the CP near the border at GW21. **c**, Spatial graphs for GW18 occipital cortex showing all H3 subtypes and EN-ET, EN-IT, EN-Mig subtypes, respectively. No V1- or V2-specific clusters are identified at GW18, despite variation in cluster proportions within the putative V1 and V2 areas shown in **d**. **d**, Quantitative comparison of neuronal subtype composition in the annotated regions of V1 and V2 (magenta and green boxes in **c**), shown separately among EN-ET, ET-IT and EN-Mig. **e**, Z-score spatial plots showing that the genes that mark a sharp V1-V2 border at GW20 and 21, including *NPY*, *PDZRN4*, and *ABI3BP* were all expressed at very low levels at GW18. The MERFISH experiments were conducted using an expanded 960 gene panel (Supplementary Table 8), and thus not integrated with other experiments analyzed with the initial 300 gene panel. Clustering and annotation were conducted individually for each new sample (Supplementary Table 9). Scale bars, 500 µm.

To expand our analysis, we designed a new 960 gene panel that expands on the original 300 gene panel (Supplementary Table 8). We conducted MERFISH on additional GW18, GW20 and GW21 samples, and clustered the cells independently for each new experiment (Supplementary Table 9). Sharp borders splitting the V1 and V2 were consistently delineated by neuronal subtypes across all cortical layers at GW20 and GW21, but not at GW18 (Extended Data Fig. 14a–c). At GW18, we did not find V1-specific or V2-specific clusters, but a modest difference in their neuronal subtype compositions was observed (Extended Data Fig. 14d). Therefore, we pinpointed that the sharp V1–V2 border is established around GW20 at the earliest.

**Extended Data Fig. 15:**
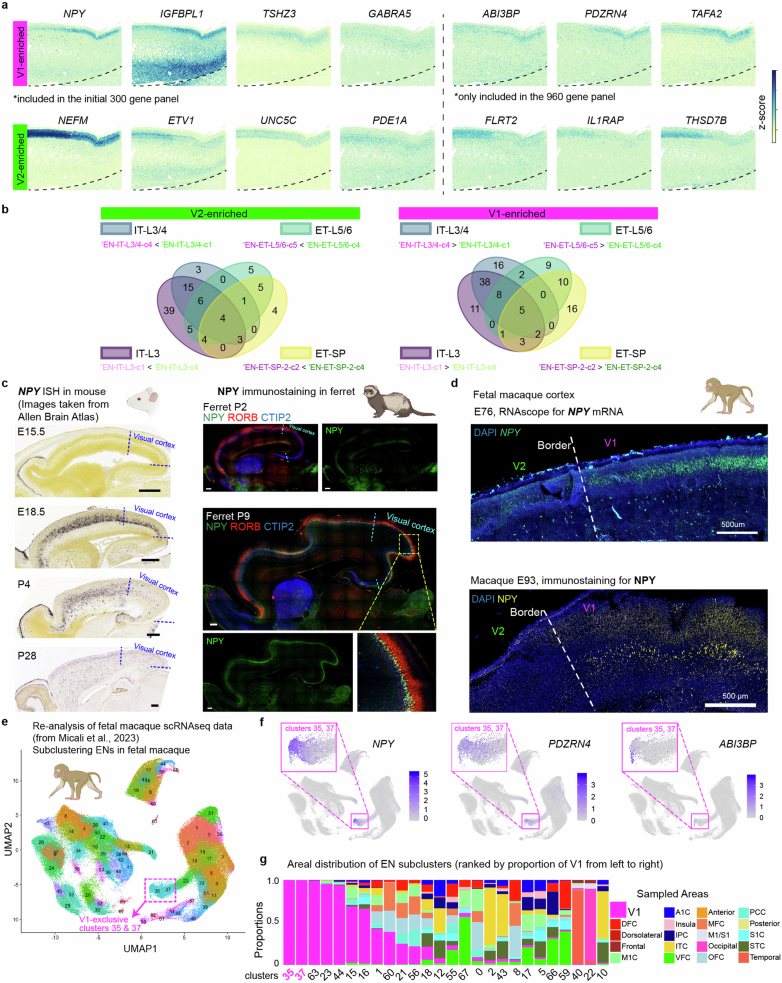
Expression patterns of V1-V2 border markers in human, mouse, and macaque. **a**, Z-score spatial maps of V1- and V2- enriched genes in GW21. To the left of the dashed lines shows the genes in Fig. 5g, while to the right of the dashed lines are genes only included in the expanded 960 gene panel. **b**, Venn diagrams for the overlap between V2-enriched (left) and V1- (right) DEGs across the four pairs of subtypes in Fig. 4c. Only strong DEGs with Log fold change >0.5 and adjusted p-value < 0.0001 were considered. See Supplementary Table 10. **c**, NPY expression in mouse and ferret cortex at various developmental stages does not resemble the pattern observed in human fetal cortex. Left, in situ hybridization images are taken from Allen Brain Atlas (https://developingmouse.brain-map.org/). Right, immunostaining images for P2 and P9 ferret cortex (repeated independently for 3 times) show similar areal and laminar expression pattern with mouse: In P9 ferret, NPY is expressed in the upper part of Layer 5, overlapping with Layer 5 marker CTIP2, but not Layer 4 marker RORB. This pattern differs from the V1-specific expression in Layers 3 and 4 observed in human GW20. Magnified view is shown to highlight the laminar pattern. The region of putative visual cortex (containing both V1 and V2) is labeled. **d**, NPY shows a clear V1-V2 border in mid-gestation fetal macaque (repeated independently for 2 times), as shown in E76 by single-molecule in situ hybridization (RNAscope) (top) and in E93 by immunostaining (bottom). **e**, Re-analysis of a published fetal macaque scRNAseq dataset^46^ identified 58 unsupervised clusters for EN, as shown by UMAP. Notably, clusters 35 and 37, highlighted in box, are exclusively found in V1 at E76 and E93. **f**, V1-specific marker genes identified in human mid-gestation show exclusive expression in clusters 35 and 37, but not in any other clusters. **g**, Histograms showing areal distribution of macaque EN clusters. Clusters are ranked from left to right based on the proportion contribution from V1. Not all clusters are shown due to space restrictions. Sample area annotations were taken directly from original dataset based on microdissection^46^. Scale bars, 500 µm. The schematic in **d**,**e** was created using BioRender (https://www.biorender.com).

A spatial transition of gene expression underlies the neuronal subtype border (Fig. 4g). Although most DEGs showed changes within 1–2 mm of the border, *NPY* stood out by displaying a definitive binary switch, with a strong enrichment observed in layers 3 and 4 of the V1 (Fig. 4g and Extended Data Fig. 15a). This distinct pattern only emerged at GW20 and GW21, but not GW18 (Extended Data Fig. 14e). Differential gene expression analysis (DEA) of each pair of V1 and V2 clusters revealed that few DEGs were universally shared among all four pairs, but more DEGs were shared in EN-ITs or EN-ETs (Extended Data Fig. 15b and Supplementary Table 10).

Notably, similar V1–V2 gene expression patterns were not present in the developing cortex of mice and ferrets. However, we found a clear V1–V2 border marked by *NPY* in mid-gestation (embryonic day 76 (E76) and E93) macaques (Extended Data Fig. 15c,d). Subclustering of fetal macaque scRNA-seq data^46^ confirmed the presence of V1-exclusive EN subtypes that specifically expressed *NPY*, *PDZRN4* and *ABI3BP*, which indicated that this molecular border between mid-gestation V1 and V2 is a developmental feature shared between humans and macaques and is potentially conserved among primates (Extended Data Fig. 15e–g).

**Extended Data Fig. 16:**
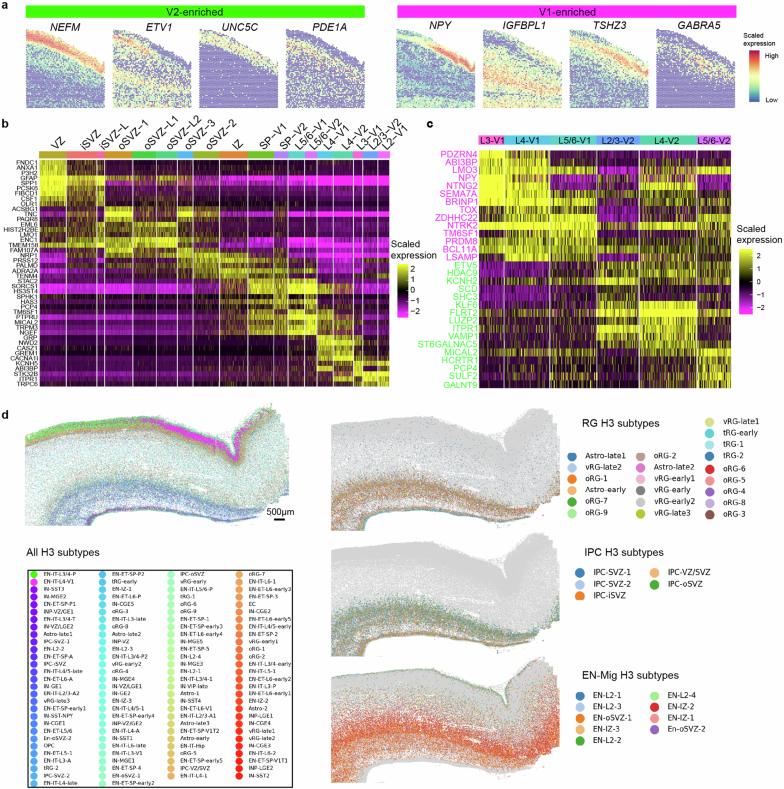
Supplementary analysis for the V1-V2 border at GW20. **a**, Visium spatial graphs for V1- and V2-enriched genes show high consistency with MERFISH measurements in Fig. 4g. **b**, Heatmap showing the expression of top enriched marker genes for each Visium cluster. Columns represent individual Visium detection spots. **c**, Heatmap showing the expression of top DEGs between V1 and V2 Visium spot clusters identified separately for each cortical layer. **d**, Left, spatial graph for the medial occipital cortex at GW20, colored by all H3 subtypes from integrated clustering, show the V1-V2 border is distinctly visible in the CP. Scale bar, 500 µm. Right, spatial graphs for EN-Mig, IPC, and RG subtypes show that a border between V1 and V2 is not seen in the IZ, SVZ, or VZ.

We conducted Visium profiling on consecutive sections analysed by MERFISH to expand our spatial analysis to the whole transcriptome. The results validated the presence of sharp borders and the gene-expression patterns identified by MERFISH and revealed additional DEGs (Fig. 4h and Extended Data Fig. 16a–c). The border divided the V1 and V2 in a straight line across all cortical layers and the SP, but the IZ, the oSVZ, the iSVZ and the VZ appeared homogeneous (Fig. 4i). Such distinctions were supported by the UMAP plots of Visium spots, which demonstrated two parallel trajectories for the V1 and V2 that bifurcated at the SP and beyond (Fig. 4j). This finding was consistent with the MERFISH results, in which no cell-subtype difference was observed in the IZ, the oSVZ, the iSVZ or the VZ (Extended Data Fig. 16d).

## Transcriptional signature of V1 neuron specification

**Fig. 5:**
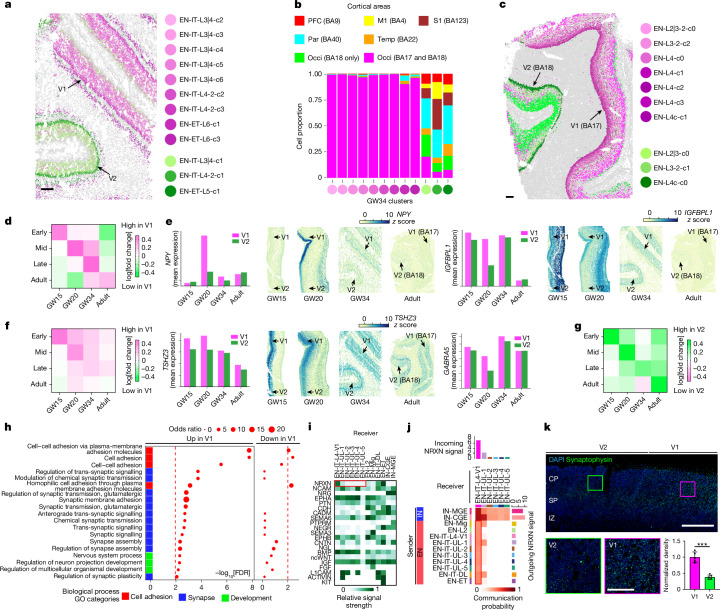
Temporally dynamic gene expression underlies post-migratory neuron specification in the V1. **a**, MERFISH spatial map shows that the V1 and the V2 contain distinct neuronal subtypes at GW34. **b**, Histograms for areal distribution show that V1-enriched subtypes (purple) are exclusively found in the V1 (BA17). **c**, The V1 and V2 also contain distinct neuronal subtypes in adulthood. **d**, Summary heatmap shows that V1-enriched gene sets for EN-ITs are distinct at each time point. Each row corresponds to the set of V1–V2 DEGs identified at different time points: early, GW15; mid, GW20; late, GW34. The cumulative mean expression levels of the gene sets were compared between the V1 and the V2, and the log[fold change] values of V1–V2 are plotted as heatmap colours. The following experiments were analysed: UMB1117-O1, FB080-O1c, UMB5900-BA17 and UMB5958-BA17. See Supplementary Table 11 for the full list of DEGs. **e**, *NPY* and *IGFBPL1* show the most prominent V1 enrichment specifically at GW20. **f**,**g**, Summary heatmaps, similar to **d**, show that V1-enriched gene sets for EN-ETs are more stable over time than EN-ITs (**f**), and V2-enriched gene sets for EN-ITs are more stable over time than those enriched in the V1 (**g**). **h**, GO analysis reveals synapse-associated and cell-adhesion-associated biological processes are upregulated in V1-specific layer 4 neurons. **i**, Heatmap for incoming signalling pathways shows NRXN signalling is specifically enriched in V1-specific layer 4 neurons (EN-IT-L4-V1 cluster; highlighted by the red outline) compared with other upper-layer (UL) EN-IT clusters. **j**, Network heatmap shows that EN-IT-L4-V1 is unique among upper-layer EN-ITs to receive NRXN signalling from both IN and EN sources. Bar graphs on the top and right show the sum of interaction strength as incoming and outgoing signals, respectively. **k**, Immunostaining for synaptophysin validates upregulation of synaptogenesis in layer 4 of the V1 at GW20. Quantification shows mean ± s.d., *n* = 5 images from 3 samples. ***P = 0.00037, Student’s two-sided *t*-test. Scale bars, 500 µm (**a**,**c**,**k** (top)) or 100 µm (**k**, bottom).

**Extended Data Fig. 17:**
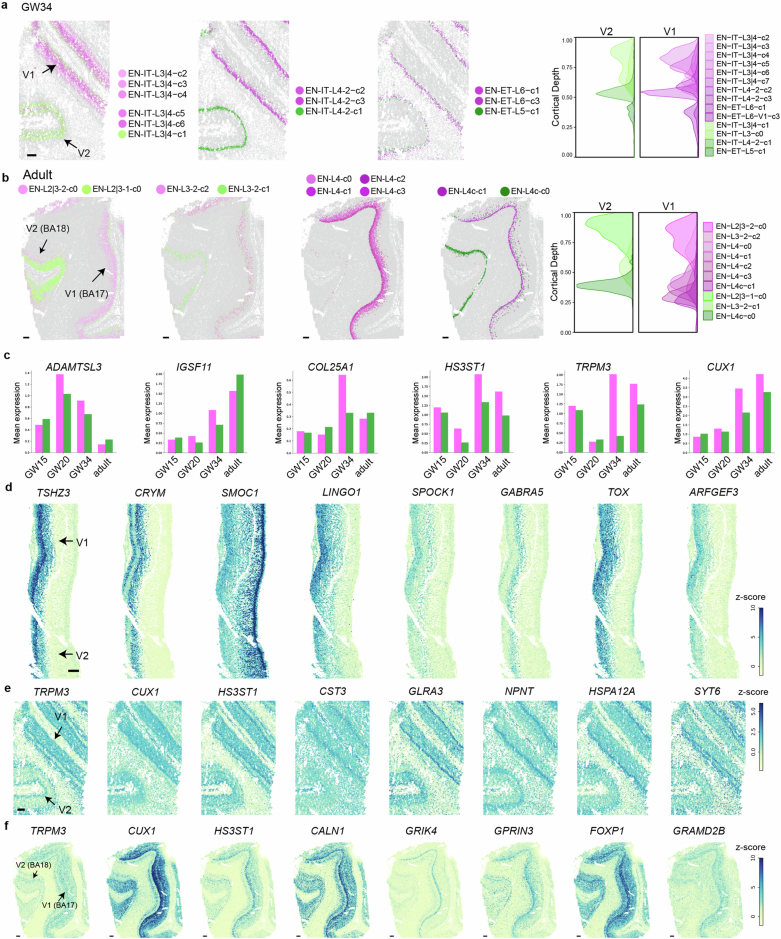
Additional characterization on V1-V2 gene expression dynamics. **a, b**, Spatial maps for EN subtypes showing V1- and V2-specific localization at GW34 (**a**) and adult (**b**), and ridgeline plots (right) showing the laminar distribution of V1- and V2- enriched EN subtypes within the cortical plate/grey matter. **c**, Examples of genes exhibiting dynamic V1-enrichment patterns among EN-ITs. **d-f**, Z-score spatial maps showing the expression pattern of genes exhibiting strong V1- enrichment at GW15 (**d**), GW34 (**e**), and adult (**f**). Scale bars, 500 µm.

After identifying that V1-specific neuron specification takes place at GW20, we next analysed its progression over time. EN subtypes that displayed mutually exclusive distributions between the V1 and V2 were observed at GW34 and adult stages, which suggested that the distinction was probably maintained continuously from GW20 (Fig. 5a,c and Extended Data Fig. 17a,b). Consistent with the GW20 pattern, V1-specific subtypes at GW34 were exclusively localized in the V1, whereas V2 subtypes exhibited a broader distribution along the AP axis (Fig. 5b).

We performed DEA to identify sets of V1-enriched genes at early, mid, late and adult stages separately in EN-ITs and EN-ETs (Supplementary Table 11). The collective expression of these gene sets was compared between the V1 and V2 at GW15, GW20, GW34 and adult stages. Among EN-IT clusters, V1-enriched genes exhibited substantial divergence over time, with distinct—and at times contradictory—gene sets identified across stages, particularly between prenatal and adult periods (Fig. 5d and Extended Data Fig. 17c). For instance, *NPY* and *IGFBPL1* were strongly enriched in the V1 at GW20, but showed reduced enrichment at GW34 and were absent in adulthood (Fig. 5e). Conversely, genes such as *TRPM3* and *CUX1*, strongly enriched at both GW34 and adulthood, did not show significant V1–V2 differences at GW15 or GW20 (Extended Data Fig. 17c–f). Unlike the transient patterns observed for V1-specific EN-ITs, V1-enriched gene sets in EN-ETs and V2-enriched gene sets remained more stable over time, with genes such as *TSHZ3* and *GABRA5* consistently showing moderate V1 enrichment (Fig. 5f,g). These findings indicate that V1 specification involves at least two distinct transcriptional programs: one transiently activated at mid-gestation for EN-ITs and another maintained throughout development for EN-ETs.

**Extended Data Fig. 18:**
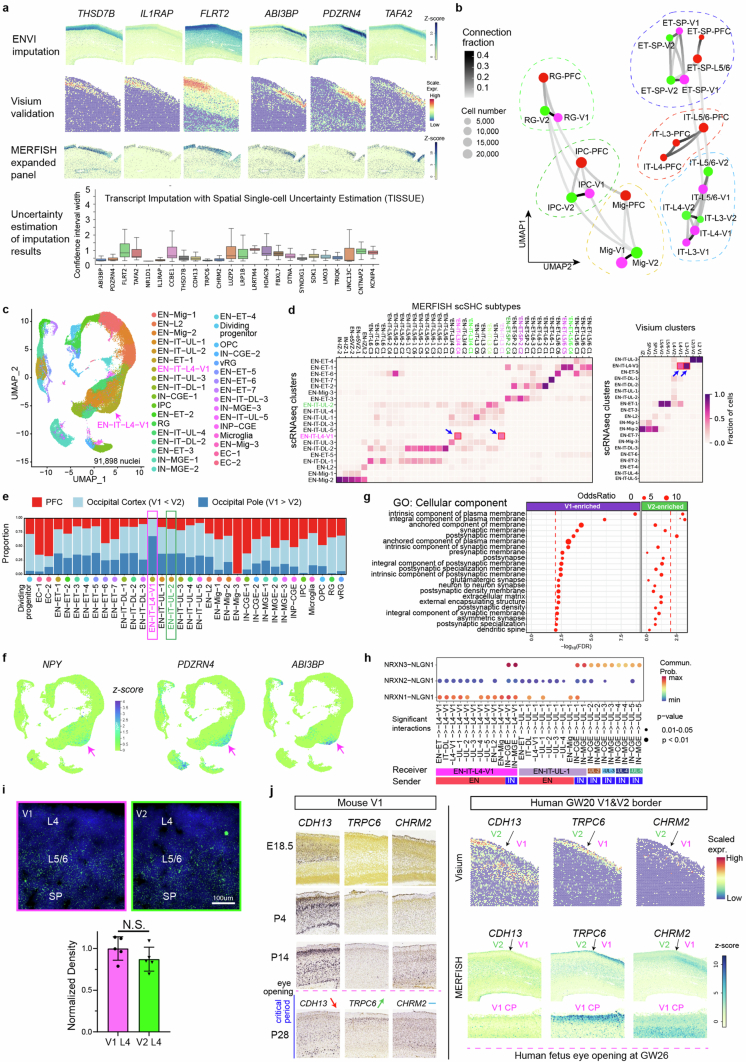
Integrated snRNAseq and MERFISH analyses at the V1-V2 border. **a**, ENVI Imputation results for V1-V2 border markers (row 1) show consistent expression patterns with direct measurements using Visium (row 2) and expand MERFISH gene panel (row 3), validating the accuracy of imputation. Row 4, box plots showing the distribution of uncertainty for imputation results of V1-V2 border genes (n = 467082 cells analyzed), estimated using Transcript Imputation with Spatial Single-cell Uncertainty Estimation (TISSUE) pipeline^48^. Uncertainty values are represented as the logged width of the 95% confidence interval for imputed expression (in reads per cell), smaller values indicate higher confidence of prediction accuracy. Box plots show media value as center; lower and upper bounds of box correspond to the first and third quartiles; whiskers extend up to 1.5 times the interquartile range from the lower and upper bounds of the box. **b**, Constellation plots of cell types in different cortical areas show V1 and V2 share a developmental lineage that is distinct with PFC. Matching nodes between V1 and V2 have connection fraction > 0.2 except for EN-IT-L3 and EN-IT-L4. See Supplementary Table 12. **c** UMAP for 91,898 cells analyzed by snRNAseq, colored by unsupervised clustering. Adjacent tissue sections used for MERFISH experiments were dissociated for snRNAseq. FB121_F1 is GW20 PFC sample, FB080_O1 and FB080_O2 are both GW20 occipital cortex sample, containing both V1 and V2. FB080_O2 is closer to the occipital pole, and therefore contains a higher proportion of V1 than FB080_O1. **d**, Heatmaps showing cell type correspondence of snRNAseq clusters with MERFISH scSHC EN subtypes (left), and with Visium spot clusters (right). EN-IT-L4-V1 cluster from snRNAseq corresponds strongly to V1-specific Layer 3&4 EN-ITs (arrows), whereas EN-IT-UL-2 corresponds to V2-enriched Layer 3&4 EN-ITs. **e**, Histogram for the cell number contribution from each sample towards each cluster, highlighting the V1-exclusive presence of EN-IT-L4-V1. **f**, Z-score plots for the expression of V1- and V2- enriched genes demonstrate high consistency between snRNAseq, MERFISH, and Visium analysis. **g**, Gene ontology (GO) analysis reveals genes associated with synapse-related membrane components are upregulated in EN-IT-L4-V1, comparing to EN-IT-UL-2. **h**, Dot plot for specific signaling interactions between neuronal clusters highlight EN-IT-L4-V1 is unique among upper layer (UL) EN-ITs in receiving strong NRXN1-NLGN1 signaling from EN sources. Only significant interactions with p-value < 0.05 (one-sided permutation test) are shown. **i**, Immunostaining for synaptophysin shows no significant V1-V2 difference in puncta density in Layer 4. Quantification shows mean ± standard deviation, n = 5 images from 1 sample (UMB1759-O1). N.S., not significant, p-value = 0.192, student’s two-sided t-test. **j**, Left, in situ hybridization images from Allen Developmental Mouse Brain Atlas (https://developingmouse.brain-map.org/) showing the expression of *CDH13*, *TRPC6* and *CHRM2* in the mouse V1 at different developmental time points. Right, Visium spatial heatmaps and MERFISH z-score spatial graphs for the expression of *CDH13*, *TRPC6* and *CHRM2* at the human V1-V2 border at GW20. *TRPC6* and *CHRM2* expressions on MERFISH data are imputed based on snRNAseq data.

We integrated snRNA-seq data from 91,898 cells from the same samples with MERFISH data using environmental variational inference (ENVI) to impute the expression of genes not included in the MERFISH panel^47^ (Extended Data Fig. 18c). The imputed spatial expression patterns of the V1–V2 border markers closely matched direct measurements from MERFISH and Visium analyses. Moreover, a computational analysis^48^ showed high confidence for the predicted gene-expression values (Extended Data Fig. 18a). We used the imputation results to expand our MERFISH analysis to include the top 1,000 highly variable genes from snRNA-seq. We also constructed a constellation plot to analyse the nearest neighbour connection between cell-type nodes of the PFC, the V1 and the V2 (Extended Data Fig. 18b). Nodes representing RG, IPCs and EN-Mig cells between the V1 and V2 were strongly connected (connection fraction > 0.2) but not with the PFC (Extended Data Fig. 18b and Supplementary Table 13). This finding indicates that V1-specific EN subtypes share a developmental lineage with V2-enriched EN subtypes but are induced to diverge after migration.

DEA between the snRNA-seq clusters corresponding to the V1-specific and V2-specific layer 3 and 4 EN subtypes revealed sets of genes enriched in the V1 and V2 (Extended Data Fig. 18d and Supplementary Table 13). Gene ontology (GO) analysis of V1-enriched genes, but not V2-enriched genes, revealed cell adhesion and synapse assembly as the most associated biological processes. Moreover, most associated cellular components were synapse-related membrane components, which suggests that there is V1-specific early upregulation of synaptogenesis (Fig. 5h and Extended Data Fig. 18g). This finding was corroborated by a ligand–receptor cell–cell communication analysis using CellChat^49^, which highlighted neurexin–neuroligin (NRXN–NLGN) signalling, which is fundamental to synapse formation^50^, as the most specifically enriched signal received by V1-specific layer 4 neurons (Fig. 5i). Although other upper-layer EN-IT clusters received NRXN signals from IN sources, V1-specific layer 4 neurons were distinct in also receiving strong NRXN signals from EN sources (Fig. 5j and Extended Data Fig. 18h). This finding was validated by immunostaining, which revealed higher synaptic puncta density in the V1 than the V2 at GW20 but not at GW18 time points (Fig. 5k and Extended Data Fig. 18i).

Previous studies of the V1 in postnatal mice identified crucial roles of visual inputs during the postnatal ‘critical period’ (postnatal day 21 (P21)–P38) in establishing V1-specific neuronal identities, which are marked by *Cdh13* downregulation and *Trpc6* upregulation in layers 2 and 3 (ref. ^51^). Our GW20 V1 data exhibited expression patterns of *CDH13* and *TRPC6* that aligned with mouse P28 but not with earlier times (Extended Data Fig. 18j). Notably, the lower *CDH13* and higher *TRPC6* levels in the V1 than in the V2 at GW20 suggest that these changes might accompany the V1–V2 fate divergence. This parallel implies that thalamic afferents may contribute to human V1–V2 differentiation in a fashion similar to their postnatal role in mice near the time of eye opening, but at a much earlier, prenatal stage in humans that is close to the time of eye opening of human fetuses, which occurs at about GW26 (refs. ^4,10^).

## Discussion

In this study, we used MERFISH to create an extensive spatially resolved single-cell atlas of the developing human cerebral cortex. To enhance accessibility, we developed a web-based browser for spatial visualization of our data (https://walshlab.org/research/cortexdevelopment). Recent advances in spatial transcriptomics have transformed our understanding of human brain development^52,53^, and our study differs from previous studies by its focus on the cerebral cortex during mid-gestation and late-gestation periods. By enhancing MERFISH with a deep-learning segmentation pipeline, we achieved spatially resolved single-cell analysis that integrates molecular and spatial information. Notably, we found that distinct cortical layers and areal borders emerge months before visible morphological differences, thereby revising the established timeline of human cortical development. Although some experiments conducted later in the study were not included in the integrated analyses presented in Figs. 1–3, their inclusion would probably support the same conclusion.

To address the gene-panel limitations of MERFISH, we complemented it with whole-transcriptomic methods such as 10x Visium and snRNA-seq. Although across-modal imputation of gene expression between spatial and single-cell data remains a challenge in the field, our use of the ENVI imputation method produced robust results that were validated by direct measurements. Two factors contributed to this success: the large number of cells analysed by MERFISH and our use of consecutive tissue sections for MERFISH and snRNA-seq, which ensured that identical cell contents (both in cell types and their proportions) were analysed between these methods, thereby enhancing the performance of the imputation.

Our study revealed two distinct modes of human cortical arealization in mid-gestation: a continuous mode observed along anatomical axes and a discrete mode specific to the V1 among the areas we analysed. Echoing previous findings^6^, we observed gradients of cell-subtype variation along the AP axis that is probably the result of progenitor patterning. However, the identification of a distinct boundary between the V1 and V2 by GW20—present in post-migratory ENs but undetectable in progenitors or migrating neurons—indicates that some areal specification occurs in a post-migratory manner. The distinct V1–V2 border, coupled with the exclusivity of V1-specific subtypes, is in marked contrast to the more continuous AP gradients of cell types observed elsewhere. This finding indicates that additional mechanisms may be involved in shaping discrete areal boundaries. Therefore, our study provides direct evidence for an integrated perspective on the protomap hypothesis and the protocortex hypothesis.

The distinct V1–V2 demarcation implicates a potential role for afferents, presumably from the dorsal lateral geniculate nucleus (dLGN) of the thalamus. These afferents reach the cortex as early as GW12–GW14 in humans—a remarkably earlier developmental stage than in rodents^54^. After arrival, these axons initiate synapse formation and transmit spontaneous thalamic activity to the cortex, which potentially influences V1 neuronal identities through activity-induced mechanisms^10,54–57^. The early innervation of layer 4 of the V1 by dLGN axons aligns with the upregulation of synaptogenesis observed in our study. Damage to these afferents in macaques disrupts normal formation of the V1 cytoarchitecture^58,59^. Moreover, studies of rodents have demonstrated that disruptionsof visual inputs lead to failure in fate specification of V1 neurons and result in a shift towards V2-like ‘default’ molecular identities and blurring of the V1–V2 border^51,55,60,61^. The V1–V2 boundary observed in the SP may also result from the initial interaction of thalamic axons with SP neurons before targeting into layer 4 (ref. ^62^). Although we could not precisely define the primary somatosensory and auditory cortices in our samples, additional analyses with broader areal coverage may reveal border formation mechanisms similar to those observed in the V1.

## Methods

### Human sample preparation and acquisition

Research performed on samples of human origin was conducted with approval of the institutional review board of Boston Children’s Hospital. De-identified human fetal tissue samples were collected after written informed consent was acquired from two sources. (1) Samples were received from the Beth Israel Deaconess Medical Center Department of Pathology, with a maximum post-mortem interval of 12 h. Tissue was transported in Hibernate-E medium on ice to the laboratory for research processing. Cortical tissue was then dissected into coronal pieces of 1–3 cm^2^ cross-section area and 0.5–1 cm thickness. The dissection was performed under the supervision of a neuropathologist to provide annotation of cortical areas based on anatomical location and features. Tissue pieces were then directly frozen in liquid nitrogen and stored at −80 °C. (2) Banked fresh-frozen tissues were obtained from the University of Maryland Brain and Tissue Bank through the NIH NeuroBioBank. Only samples with postnatal intervals of <12 h were used. Cortical area annotation was provided by NeuroBioBank. Tissue was shipped overnight in dry ice and stored at −80 °C. For both sources, only samples with no neurological anomalies were analysed. Samples were screened for RNA quality by collecting 50-µm-thick cryosections, isolating total RNA and measuring the RNA integrity number using an Agilent 4200 TapeStation system, and RNA samples with integrity numbers of ≥6.5 were used in the study. Details of the samples used are summarized in Supplementary Table 2.

### Annotation of cortical areas

The cortical areas of tissue sections used in MERFISH analyses were annotated on the basis of the reference atlas for human fetal brain developed by the Allen Institute for Brain Science (https://atlas.brain-map.org)^30^. For GW20 and GW22 samples, dissection of the tissue was performed under the supervision of a neuropathologist to distinguish the frontal lobe, the parietal lobe, the occipital lobe and the temporal lobe. The relative AP, dorsal–ventral and medial–lateral location of each piece of dissected tissue was recorded by notes and photographs. For GW15, GW18 and GW21 samples obtained from the NIH NeuroBioBank, annotation of cortical lobes was provided by NeuroBioBank staff. For more specific annotation of cortical areas, the GW15 samples were compared with the GW15 reference atlas (https://atlas.brain-map.org/atlas?atlas=138322603), and the GW18, GW20, GW21 and GW22 samples were compared with the GW21 reference atlas (https://atlas.brain-map.org/atlas?atlas=3). The relative AP locations of tissue sections were matched with the reference atlas, and cytoarchitectural features such as early sulci were used as reference landmarks. The transition between the PMC and the M1 lacks clear definition in mid-gestation; therefore, some tissues analysed were annotated as PMC/M1. By contrast, the calcarine sulcus was morphologically well defined in GW15–GW22 samples, which enabled the identification of the V1 and the V2. For GW34 and adult samples from NeuroBioBank, the specific Brodmann’s area corresponding to the postnatal human cortex was determined by NeuroBioBank staff and was provided when tissues were requested. Definition of the specific Brodmann’s area is possible only for GW34 and adult samples because by this age, the major sulci and gyri structures have formed, which is not the case for GW15–GW22 periods. Annotation of the cortical area for all samples is summarized in Supplementary Table 2.

### Immunohistochemistry and microscopy

Cryosections (10 µm thick) of human fetal tissue were placed on Superfrost slides (Fisher) for immunohistochemistry. Preparation of fetal macaque tissue samples are described separately, but immunostaining for NPY followed the same procedure. The samples were first fixed in 4% paraformaldehyde (PFA) for 15 min at room temperature, followed by permeabilization with 0.5% Triton-X in PBS for 1 h and blocked with blocking solution of 10% donkey serum in PBS and 0.05% Triton-X (PBST) for 30 min. Primary antibodies diluted 1:500 in blocking solution were applied to the sections overnight at 4 °C. Primary antibodies used were rat anti-CTIP2 (Abcam, ab18465), mouse anti-SATB2 (Abcam, ab9244), rabbit anti-TBR1 (Abcam, ab31940), rabbit anti-NPY (Abcam, ab30914) and goat anti-synaptophysin (R&D Systems, AF5555-SP). After washing with PBST for a minimum of 5 times, secondary antibodies and DAPI (1:2,000) diluted in blocking solution were applied to the sections for 1–4 h at room temperature or overnight at 4 °C. Secondary antibodies used were AlexaFluor 488-conjugated, 555-conjugated or 647-conjugated donkey antibodies (Invitrogen) used at 1:500 dilution. Finally, sections were washed with PBST for a minimum of 5 times before mounting with Vectashield Vibrance Antifade mounting medium (Vector Labs, H-1700-10). Images were captured using a Zeiss LSM 980 confocal microscope. The *z* stack function was used to image at 10 µm thickness, and the tile-stitching function was used. Sample images were prepared in ImageJ software (Fiji 2.15.1) and Adobe Photoshop (m.2506).

### MERFISH gene-panel selection and probe construction

We initially designed a panel of 300 genes (Supplementary Table 3) that was used for most of the experiments. The panel contained 39 canonical markers for major cell types in the human fetal cortex, including markers for ENs and INs, IPCs and neural progenitor cells; 32 were validated cortical-layer markers in the adult human cerebral cortex from a previous study^38^. Thirty genes associated with autism spectrum disorders were selected from the SFARI Gene Portal (https://gene.sfari.org/database/human-gene/)^64^, and one putative regulator long noncoding RNA for each SFARI gene was included (30 in total). The remaining genes on the panel were obtained from the top enriched cluster markers in published single-cell RNA SMART-seq data of mid-gestation human fetal cerebral cortex^7^. Single-cell clustering and marker identification based on differential expression were performed in the original study^7^. The top 20 marker genes based on the fold change for EN clusters and the top 5–10 marker genes for other clusters were manually curated. The genes that did not overlap with previous selected categories were included in the gene panel, which resulted in a total of 300 genes. All genes in the panel were included for subsequent analyses.

We also designed a new 960 gene panel that contained all 300 genes from the first gene panel and expanded. This panel was only used for the UMB1031-O1, UMB1759-O1 and UMB1932-O1 experiments. The following criteria were used for including additional genes: (1) genes clinically associated with malformation of the cerebral cortex; (2) genes exhibiting high expression variance from our snRNA-seq data of GW20 human fetal cortex; (3) DEGs between the V1 and V2 at GW20 identified by our Visium data; (4) genes involved in postnatal V1 specification in mice, taken from a published scRNA-seq analysis^51^; (5) new markers for interneuron subtypes identified in developing macaque brain, taken from a scRNA-seq analysis^65^; (6) additional classical interneuron-subtype markers; (7) marker genes for cell types found in the human fetal GE, taken from a scRNA-seq analysis^66^; and (8) marker genes for cell types found in the human fetal thalamus, taken from a scRNA-seq analysis^67^.

Merscope-encoding probes for the 300 and 960 genes were constructed by Vizgen using a commercial pipeline. Each of the 300 and 960 genes was assigned a unique binary barcode drawn from a 22-bit, Hamming-Distance-4, Hamming-Weight-4 encoding scheme^9^ (Supplementary Table 4). Fifteen extra barcodes (for the 300 gene panel) and 40 extra barcodes (for the 960 gene panel) were included as blank barcodes, which were not assigned to any genes to provide a measure of the false-positive rate in MERFISH, as previously described^9^.

### MERFISH imaging

MERFISH experiments was performed using a Vizgen Merscope system. Sample preparation was performed according to manufacturer’s instructions (‘Merscope Fresh and Fixed Frozen Tissue Sample Preparation User Guide’, number 91600002). In brief, fresh-frozen tissues were embedded in OCT and sectioned into 10-µm-thick sections using a cryostat (Leica) and adhered to Merscope slides (Vizgen, 1050001) placed in a 6-cm Petri dish. For GW15–GW22 samples, slides were kept inside the cryostat maintained at −15 °C for 30 min to allow the section to dry and firmly adhere to the glass slides before fixation. For GW34 samples, slides were kept inside the cryostat for 5 min and then transferred to room temperature for 15 min before fixation. Slides were then fixed in 4% PFA and permeabilized by 70% ethanol overnight, with Parafilm sealing the Petri dish to prevent evaporation. Slides were then treated in a Merscope photobleacher instrument for autofluorescence quenching for 3 h. Slides were stored overnight or up to 1 week before proceeding to the next step. The encoding probe mix was added directly on top of the tissue section for hybridization at 37 °C for 36 h in a humidified incubator. After probe hybridization, sections were fixed again using formamide and embedded in gel. After gel embedding, tissue samples were cleared using a clearing mix solution supplemented with proteinase K for 24–48 h at 37 °C until no visible tissue was evident in the gel. After clearing, sections were stained for DAPI and PolyT and fixed with formamide before imaging. No additional cell boundary staining was used. Reagents used for these steps were included in Merscope sample preparation kit (Vizgen 10400012).

The MERFISH imaging process was performed according to the Merscope Instrument Preparation guide (number 91500001). In brief, an imaging kit was thawed in a 37 °C water bath for 45 min, activated and loaded into the Merscope instrument. The flow chamber was then assembled, fluidics were primed and the flow chamber filled with liquid. A low-resolution image for the DAPI and PolyT stains was taken at ×10 magnification, and a region of interest (ROI) was manually drawn in the Merscope software, followed by automated image acquisition and fluidic control in the Merscope instrument. For each section, a ROI of up to 1 cm^2^ area was imaged as 2,000–2,500 tiles at ×40 magnification, and images were collected in the 750 nm, 650 nm and 560 nm channels for the readout probes and in the 488 nm and 405 nm channels for PolyT and DAPI staining, respectively. Seven *z* stacks were captured over a thickness of 10 µm. After imaging, image processing and transcript decoding were performed using Merscope proprietary software. The transcript matrix with spatial coordinates and the stitched tiled DAPI images acquired were transferred for subsequent processing and single-nucleus segmentation. Vizgen Merscope Visualizer (v.2.1.2595.1) software was used to visualize each experiment to determine whether the MERFISH run was successful before systematic analysis was performed.

### Nucleus segmentation and MERFISH data processing

Automated segmentation was performed on the DAPI channel using a custom CellPose model^23,24^. The model was initialized with the CellPose (2.0) ‘cyto’ weights, then trained for 300 epochs with a learning rate of 0.1 and a weight decay of 1 × 10^–4^ using 145 manually segmented images for training and 3 for testing. All images, before training and running segmentation, were filtered using a difference of Gaussians filter, which consisted of a positive Gaussian having zero standard deviation (all weight at zero) and a negative Gaussian with a standard deviation of 20 pixels. When running the CellPose segmentation, for testing and in the Vizgen pipeline, the cell diameter was set to 55 pixels (average from training data), the flow threshold was set to 0.5, the cell probability threshold was set to –3 and the minimum mask size was 500 pixels.

The Vizgen Merscope output consisted of 7 planes evenly spaced across 10 µm. Given the wide point-spread function and the small spacing, we used the maximum projection image over the first six planes as input to the CellPose segmentation algorithm, using the CellPose parameters and filtering as specified above. Because nucleus staining (DAPI) was used, we dilated the masks by 10 pixels to approximate the cytoplasmic area of the cells. These processing steps were added to the Vizgen Post-processing Tool (v.1.2.0); the modified code is available from GitHub (https://github.com/carsen-stringer/vizgen-postprocessing). The Vizgen processing pipeline uses the CellPose masks to define ROIs and then assigns the RNA transcripts to each ROI to return a cell-by-gene matrix. In Extended Data Fig. 1b, we quantified the segmentation accuracy using the average precision metric, which is the number of true positives divided by the total number of true positives, false negatives and false positives^24^. The true positives were defined as segmented ROIs that matched ground-truth ROIs at or above a defined intersection-over-union threshold. The false negatives were the ground-truth ROIs that were missed, and the false positives were the predicted ROIs that did not match any ground-truth ROIs.

To compare the transcriptomic profiles between MERFISH cells segmented with and without dilation, we performed a test (Extended Data Fig. 1d,e). The same experiment (FB080-O1c) was segmented twice using our CellPose and Vizgen Post-processing Tool pipeline, with the dilation function disabled or enabled, respectively. The cells segmented with and without dilation were combined, preprocessed and clustered into 30 unsupervised clusters using sklearn.cluster.KMeans(). The resulting clusters were visualized using UMAP in Extended Data Fig. 1d. To further compare the source distribution of cells in each cluster, we calculated and visualized the proportions of dilated and non-dilated cells in each cluster as a bar plot in Extended Data Fig. 1d. We also compared the distribution of transcript count in each cluster (Extended Data Fig. 1e).

### MERFISH data quality control and integrated hierarchical clustering

After performing nucleus segmentation, for each experiment, we filtered out all cells with a total transcript count below the tenth percentile specific to that experiment. We then normalized each cell by its total transcript count using scanpy.pp.normlize_total(). Transcript counts were then log-transformed and *z* score-normalized using scanpy.pp.log1p() and scanpy.pp.scale(), respectively. Once preprocessed, we integrated the gene-expression data from all samples. Subsequently, hierarchical clustering was performed on the integrated dataset to identify groups of cells with similar gene-expression profiles. Initially, all cells were grouped into eight H1 clusters at the first hierarchy (cell class). After this step, each H1 cluster was subclustered into 5 preliminary H2 clusters, which resulted in 40 preliminary H2 clusters (cell type). Each H2 cluster was further subdivided into 5 preliminary H3 clusters to form a total of 200 preliminary H3 clusters (cell subtypes). The clustering results at each hierarchy were obtained using sklearn.cluster.KMeans(). Marker genes were then identified using scanpy.tl.rank_gene_groups() and scanpy.get.rank_genes_group_df(). For a given H1 cluster, genes with a log_2_[fold change] of at least 0.25 compared with the cells in all other H1 clusters were denoted as marker genes for that cluster. Marker genes for each H2 or H3 cluster were identified using the log_2_[fold change] between that cluster and the other four clusters belonging to the same H1 or H2 group, respectively.

Cluster annotations were conducted sequentially from H1 to H3 on the basis of the marker gene list and the spatial distribution of each cluster. The eight H1 cell classes were annotated on the basis of expression of canonical markers. Some preliminary H2 and H3 clusters were manually merged. Because ECs were not of particular interest in our study and our gene panel has limited relevant genes for ECs, all EC clusters were merged into one cluster. Similarly, astrocytes and oligodendrocyte (OPC) clusters were merged into three H2 clusters, and subsequently five H3 clusters. H3 clusters for IPCs derived from each H2 IPC cluster were highly similar in spatial distribution and gene expression; therefore, these were also merged. Finally, we noted that a few clusters exhibited aberrant spatial distribution that reflected technical artefacts during the imaging process. These clusters, easily recognizable because of their exclusive localization at the edge of certain tissue sections or surrounding bubbles in the tissue section, were the accidental result of tissue hydrogel detaching from the surface during imaging. We removed cells from these artefact clusters from all subsequent analyses.

After transcript count filtering, artefact cluster removal and supervised merging, we analysed 15,927,370 cells from the initial set of experiments, which were clustered into 8 H1 clusters, 33 H2 clusters and 114 H3 clusters (Supplementary Table 5). H2 cell types were annotated to reflect their spatial distribution. For EN-IT, EN-ET and EN-Mig clusters, their approximated layer enrichment at GW34 were denoted. For RG and IPCs, their enriched laminar structures (such as the VZ, the SVZ and the IZ) were denoted in cluster annotation. In addition, if a cluster was predominantly present only in GW15 samples, it was denoted with ‘early’, whereas a cluster predominantly present only in GW34 was denoted with ‘late’. H3 EN clusters were similarly annotated to reflect their layer enrichment and their areal distribution enrichment (A for anterior, P for posterior, T for temporal lobe, and so on). In subsequent set of experiments that were separately analysed, 2,635,057 cells were analysed using the same quality-control procedures.

### Correlation between technical and biological replicates

To verify the reproducibility of our MERFISH experiments, we computed the mean expression for each gene and visualized the correlation between the mean expression levels from two replicates as scatterplots (Extended Data Fig. 1i,j). Pearson correlation coefficients were calculated to assess the agreement. Horizontal and vertical dashed lines marked the mean expressions of blank genes for each replicate. Technical replicates analysed included UMB1117-F1a and UMB1117-F1b, UMB1117-F2a and UMB1117-F2b, FB080-F2a and FB080-F2b, FB080-P1a and FB080-P1b, FB080-O1a and FB080-O1b, FB080-O1b and FB080-O1c, and FB080-O1a and FB080-O1c. Biological replicates included FB080-F1 and FB121-F1, FB080-F2a and FB121-F2, FB080-P1a and FB121-P1, FB080-P2 and FB121-P2, FB080-T1 and FB121-T1, and FB080-O1a and FB121-O1. The mean and standard deviation of correlation coefficients were computed and visualized in Extended Data Fig. 1k.

### Cell-label-transfer analysis

Reference-based cell-type annotation was conducted using SingleR (v.1.8.1)^68^ to transfer cell-type labels from the reference data to the testing data with the following parameters: “genes”: “de”; “de.method”: “classic”; “fine.tune”: TRUE; “prune”: TRUE. The five separate analyses are summarized below. (1) For Fig. 1f and Extended Data Fig. 3d, previous fetal human cortex scRNA-seq data^7^ were used as a reference to transfer labels to our MERFISH data. In the reference, we excluded ‘MGE-’ (cells from dorsal cortical specimens) and ‘unknown’ clusters and aggregated all ‘nIN’ subclusters into one and denoted it as ‘IN’. (2) For Extended Data Fig. 3b, previous scRNA-seq data^8^ were used as a reference to transfer cell-type labels to the MERFISH data. (3) For Extended Data Fig. 3e, previous scRNA-seq data^6^ were used to transfer cell-type labels, whereby ‘CombinedCluster-Final’ in the metadata was used to define reference cell types. Because the reference dataset from ref. ^6^ covered more gestational ages and cortical areas and non-cortical structures, it contains cell types for which counterparts are not expected to be found in our MERFISH dataset. Thus, we added a filter to hide rows with maximal correspondence fractions of <0.05 to save space on the graph. (4) For Extended Data Fig. 6d, cells from excitatory neuron (Exc) clusters in a published adult human cortex scRNA-seq dataset^35^ were extracted and served as a reference to transfer cell-type labels to EN-ET and EN-IT cells in our MERFISH data. For simplicity, the 56 original EN clusters in the adult human cortex dataset were merged into 14 groups on the basis of their shared layer and molecular marker identities. (5) For Extended Data Fig. 18d, cells from the EN clusters in our snRNA-seq data were used as a reference to transfer labels to EN cells or spots in our MERFISH and Visium data separately. The pruned labels returned from SingleR were used to annotate the target cells. Correspondences between raw and transferred cell types in the target data were visualized using heatmaps.

### Quantitative spatial analysis of MERFISH data

For the majority of tissues analysed by MERFISH, one to three fan-shaped regions were manually drawn for quantitative spatial analysis, referred to as ‘annotated area A, B or C’ in Supplementary Table 2. The fan-shaped regions were selected on the basis of relative geometrical uniformity, avoiding anatomical structures such as sulci, which may complicate location quantification. Areas with tissue section tears and bubbles were also avoided. For each tissue section analysed by MERFISH, spatial graphs of H1 cell-class distribution were generated. On the basis of these graphs, each fan-shaped region was made of hand-traced vector lines marking the apical surface and basal surface and were connected by two straight lines using the pen tool in Adobe Photoshop software. The basal borders were defined by the pial surface of the cortex. For GW15 and GW20 samples, the apical borders were drawn at the ventricular surface. For GW22 samples, the apical borders were drawn in the oSVZ as the VZ could not fit in the imaged area owing to the larger tissue size. For GW34 samples, the apical surfaces were drawn at the approximated transition between layer 6b and the white matter.

In each fan-shaped region, laminar structures of the developing cerebral cortex were further manually defined with respect to the distribution and morphology of cell classes. The CP was defined as the condensed layer of excitatory neurons (EN-ITs and EN-ETs) near the pial surface. The thin layer above the CP to the pial surface was defined as the MZ. The SP was defined as the layer containing only EN-ETs, below the CP, and with lower cell density than the CP. The IZ was defined as the relatively cell-sparse layer predominantly composed of EN-Mig cells below the SP. The oSVZ was defined as the region composed of a mixture of EN-Mig cells, IPCs and RG below the IZ. The VZ was defined as the condensed layer of RG showing clear ventricular morphology at the apical surface. The thin layer above the VZ composed of a high density of IPCs was defined as the iSVZ. The boundaries between each neighbouring laminar structure were manually traced using the pen tool in Photoshop, which established an annotated vector mask for each fan-shaped region.

The vector mask was then imported into the computational analysis object by overlapping the spatial coordinates of cells with the vectors. Therefore, each cell in the fan-shaped region was also assigned with its laminar structure (VZ, iSVZ, oSVZ, IZ, SP, CP or MZ). For each annotated region, three images were exported from Photoshop: (1) a mask containing the boundary of the fan-shaped region along with the lines delineating the boundaries of the annotated structures; (2) a mask containing only the boundary of the fan-shaped region; and (3) a mask containing the boundary of the fan-shaped region, where the lines marking the apical surface and basal surface were replaced by straight lines. The initial objective was to determine the coordinates of the apical and basal surfaces. Using image (3), we applied cv2.goodFeaturesToTrack() to identify the four corners of the fan shape. Using the orientation of the annotated region (up, down, left and right), corner coordinates were assigned to their respective ends of the apical and basal surfaces. We calculated the equations of the lines connecting the corners of the apical and basal surfaces, and the point at which the lines intersected (*O*) was determined. To identify all points comprising the apical and basal surfaces, we first set all pixels equal to 0 in the mask of image (1) that were in a 3-pixel radius of each of the corners. We then used cv2.connectedComponents() to categorize each of the four lines in the mask into different components. The component identifiers for the apical and basal surfaces were then determined on the basis of the previously calculated corner locations. To extract the cells in a specific layer, we first subtracted image (2) from image (1) to eliminate the boundaries of the fan-shaped region, including the apical and basal surfaces. With only the layer-defining boundaries remaining, we applied cv2.connectedComponents() to assign each boundary to a different component. The component identifiers corresponding to each of the layer boundaries were determined using the orientation of the fan-shaped region. To calculate the mask boundary for a given layer, we used cv2.line() to draw lines between the corresponding end points on the top and bottom boundaries for that layer. We then created the mask by applying cv2.binary_fill_holes() to the boundary. Using the coordinates of the cells located in the annotated region, we then extracted all cells with coordinates contained in the layer-specific mask.

For each cell in the annotated fan-shaped region, its RH was calculated to represent its laminar location from the apical to basal surfaces. The RH calculation normalizes for the tilting of the fan-shape created by the asymmetrical morphology of the human cerebral cortex. Similarly, for a cell in the CP of an annotated fan-shaped region, its CD was calculated to quantify its relative laminar location in the CP. To calculate the RH for a given cell, we used cv2.line() to draw a line from point *O* to the cell’s location at point *C*. Using the mask containing this line, we identified intersection points with the apical and basal surfaces (designated as *C*_3_ and *C*_1_, respectively). The Euclidean distances between *C*_3_ and *C*_1_, and between *C* and *C*_1_ were then computed. The RH of the cell was then calculated as the ratio of these distances. To calculate the CD, we first extracted all cells located in the CP of the fan-shaped region. Then, for each cell located in the CP, we used cv2.line() to draw a line connecting *O* and the location of the cell, *C*. Using the mask containing this line, we identified the points where the line intersected with the top and bottom boundaries of the cortical plate (denoted by *C*_3_ and *C*_2_, respectively). We then calculated the Euclidean distances between *C*_3_ and *C*, and between *C* and *C*_2_. The ratio of these distances defined the CD of the cell. For a given annotated region, the RH violin plot was constructed using the H2 cell-type annotations for all cells in that region. The CD violin plot was created using the H3 cell-type annotations for all EN-IT, EN-ET and EN-Mig cells in the CP of that region. For any clusters with fewer than 50 cells in that region, we represented the RH or CD distribution with individual cell dots instead of a violin plot.

Cortical layers in the CP of an annotated region were computationally defined by the distribution of CD values for specific H3 EN subtypes. Although the laminar location of most H3 EN subtypes were consistent across gestational ages, some clusters showed varying abundance with gestational age, which prevented us from using the exact same set of H3 EN subtypes for the definition of cortical layers in samples from all gestational weeks analysed. For GW15, EN-IT-L4-1, EN-ET-L5-1 and EN-IT-L6-1 were used to defined layers 3/4, layer 5 and layer 6, respectively. For GW20, EN-IT-L2/3-A1, EN-IT-L4-1, EN-ET-L5-1 and EN-IT-L6-1 were used to defined layers 2/3, layer 4, layer 5 and layer 6 respectively. For GW22, EN-L2-1, EN-IT-L3-A, EN-IT-L4-1, EN-ET-L5-1 and EN-IT-L6-1 were used to define layer 2, layer 3, layer 4, layer 5 and layer 6, respectively. For GW34, EN-L2-4, EN-IT-L3-late, EN-IT-L4-late, EN-ET-L5-1 and EN-IT-L6-late were used to define layer 2, layer 3, layer 4, layer 5 and layer 6, respectively. The borders between adjacent layers, for example, between layer 5 and layer 6, were calculated as the mean CD value between the lower 25% of layer 5 cells and the upper 75% of layer 6 cells. Therefore, the CD value for each cell in the CP was used to assign a cortical layer identity for that cell.

### Spatiotemporal expression patterns of genes

For each gestational week and cortical area, we isolated all cells in each annotated layer using their coordinates and the corresponding layer-defining masks. Cells in the CP were assigned to specific cortical layers on the basis of their CD values. Subsequently, for each gene, we calculated the mean expression across all cells in each annotated layer for a given gestational age. We then standardize the expression for that gene to the [0, 1] range across all layers and gestational weeks. The results are visualized in the summary expression heatmaps (Supplementary Data). To construct *z* score expression spatial graphs for a given cortical area, the normalized expression values for all cells in the area were extracted, and gene-expression values were *z* score-normalized using scanpy.pp.scale(). Gene-expression heatmaps were then plotted using scanpy.pl.embedding().

### Cell number proportion and enrichment quantifications

The two panels at the bottom of Extended Data Fig. 9a illustrate (1) the proportion of cells in a H3 cell type for each cortical area (the PFC, the PMC/M1, the Par, the Temp and the Occi) and (2) the cell number of a H3 cell type for each gestational week (GW15, GW20, GW22 and GW34). For the cortical area proportion plot in (1), the proportion (*r*) of cells for each cortical area (CA) was calculated as the total number of cells for each CA for the H3 cell type divided by the total number of cells for that H3 cell type across all the CAs: ${r}_{{\rm{CA}},{\rm{H}}3}=\frac{\,{N}_{{\rm{CA}},{\rm{H}}3}}{{N}_{{\rm{H3}}}}$ where *N*_CA,H3_ is the number of cells for a CA at H3 and *N*_H3_ is the total number of cells at H3. For the number of cells plot in (2), the number of cells for each gestational week at each H3 subcluster is shown.

The results of spatiotemporal enrichment analysis of EN subtypes (EN-ETs, EN-ITs and EN-Mig cells) in Extended Data Fig. 9a and RG, IPC and IN subtypes in Extended Data Fig. 9b were done using the normalization process described below. The total number of cells for each sample–region (SR) pair at H1 and H3 were counted separately for each gestational week. The number of cells of SR pairs at each H3 cell type was then divided by the number of cells of SR pairs at the corresponding H1 cell type. ${r}_{{\rm{SR}},{\rm{H}}3}=\frac{\,{N}_{{\rm{SR}},{\rm{H}}3}}{{N}_{{\rm{SR}},{\rm{H}}1}}$ where *N*_SR,H1_ is the number of cells of a SR pair at H1 and *N*_SR,H3_ is the number of cells of a SR pair at H3. The ratio above was then divided by the maximum number of cells of SR pairs at H3. ${\rm{Normalized}}({r}_{{\rm{SR}},{\rm{H}}3})=\frac{{r}_{{\rm{SR}},{\rm{H}}3}}{{R}_{{\rm{H3}}}}{R}_{{\rm{H3}}}$ is the maximum of *r*_SR,H3_ among all the SR pairs at that H3 cell type.

For Extended Data Fig. 2c, the proportions of all H2 cell types in each SR pair was calculated. The number of cells of SR pairs at each H2 cell type was divided by the total number of cells in that SR. ${r}_{{\rm{H2}},{\rm{SR}}}=\frac{\,{N}_{{\rm{H2}},{\rm{SR}}}}{{N}_{{\rm{SR}}}}$, where *N*_H2,SR_ is the number of cells of a SR pair at H2 and *N*_SR_ is the total number of cells of an SR pair. Figures 4f and 5b were calculated in a similar manner.

For UMAP of EN subtype compositions (Extended Data Fig. 9c), all EN-ET and EN-IT cells from each annotated cortical region were extracted. The relative proportion of the EN-ET and EN-IT H3 subtypes was calculated for each cortical area. We computed the UMAP of the matrix of relative EN H3 cell-type proportions, providing a two-dimensional representation for each cortical area based on the EN subtype composition.

### Identification of DEGs in anterior and posterior clusters

In Extended Data Fig. 10a, five pairs of anteriorly enriched and posteriorly enriched neuronal subtype H3 clusters were compared on the basis of their DEGs: EN-IT-L2/3-A2 versus EN-IT-L3-P, EN-IT-L4-A versus EN-IT-L4-late, EN-IT-L4/5-1 versus EN-IT-L5/6-P, EN-ET-L6-A versus EN-ET-L6-P, and EN-ET-SP-A versus EN-ET-SP-P1. The DEGs were detected using the scanpy.tl.rank_genes_groups() function with the default *t*-test from the Scanpy_1.8.2 in Python (v.3.10). The top ten and bottom ten genes were selected separately on the basis of their log[fold change], and the log_10_[*P*] was used to break any ties. The top ten genes represent the most upregulated genes in the DEG analysis, whereas the bottom ten genes represent the most downregulated genes. In the bubble plots, the mean expressions for each gene across all the cells were calculated and represented by the spot colour. The percentage of expressed cells was calculated by dividing the number of cells expressed for each gene by the total number of cells expressed across all the genes, represented by the spot size.

### Identification of area-enriched genes

To identify area-enriched genes (Fig. 3h), we analysed EN-ET and EN-IT cells from GW20 and GW22 samples in the annotated fan-shaped regions. Cells in the PFC and the PMC/M1 were grouped as anterior regions and cells in the Par and the V2 were grouped as posterior regions. We identified anteriorly and posteriorly enriched genes by *t*-tests using the scanpy.tl.rank_gene_groups function. The top ten genes with the highest anterior enrichment and posterior enrichment were selected separately on the basis of their *P* values. Moreover, the top five genes with the most enrichment exclusively in the Temp were detected using the same methods. EN-Mig cells, RG and IPCs from GW20 and GW22 samples were analysed using a similar method in Extended Data Fig. 10d,e.

### Clustering by individual gestational age and single-cell significant hierarchical clustering

To compare neuronal subtypes at different gestational ages, we used an alternative clustering strategy in Figs. 4, 5 and Extended Data Fig. 11, for which only cells from the same gestational age were clustered together. Using the preprocessed data, all cells belonging to samples from the same gestational week were combined. Subsequently, hierarchical clustering was conducted for each gestational week independently. Analogous to the method applied for the integrated analysis, for each time point, we first clustered the cells into 8 H1 and 40 preliminary H2 clusters using sklearn.cluster.KMeans(). H1 and H2 clusters were annotated using the same strategy as for integrated clustering described above.

The single-cell significant hierarchical clustering (scSHC) pipeline (v.0.1.0) was used to determine in an unbiased manner the number of significant H3 subclusters^40^. scSHC adopts a hypothesis-testing approach, recursively dividing the cells into two groups and testing the significance of each split with an adjusted threshold to control the family-wise error rate. It defines Ward linkage as test statistics and uses parametric bootstrapping to estimate its null distribution. If the *P* value at a given node is larger than the adjusted threshold, the two subsequent branches are merged. For each gestational age, we randomly sampled 500,000 cells and ran scSHC in each H2 cluster. To ensure model robustness, we increased the number of cells used for null distribution estimation from 1,000 in the original method to one-third of the cluster size. Furthermore, to avoid overly small subclusters, if one subcluster comprised fewer than 10% of the cluster size, we stopped further splitting. For each run of scSHC, the following parameters were used: alpha, the family-wised error rate, was fixed at 5 × 10^–4^; num_features, the number of genes used as features, was set to 300 (all MERFISH panel genes); num_PCs, the number of top principal components retained for gene expression dimension reduction, was set to 30. The H3 subclusters generated by scSHC are summarized in Supplementary Table 7. Experiments that were subsequently added, including those using the expanded 960 gene panel, were individually clustered using the same method, as illustrated in Supplementary Table 9.

### scSHC subcluster correspondence analysis across gestational ages

To evaluate the transcriptomic correspondence of EN-ET and EN-IT scSHC subclusters across different ages, we applied XGBoost (v.2.0.3)^41^, a distributed gradient-boosted decision-tree-based classification method. For each adjacent pair of gestational stages, we trained a XGBoost classifier to learn cluster labels from gene-expression data in the earlier gestational stage. Consequently, we used the classifier to classify cells from the later gestational stage. The correspondence between the original clusters and the classifier-assigned labels in the later gestational age dataset were used to map clusters between ages. The general classification workflow is described below and was applied to each adjacent gestational age pair.

Let *X*_E_ represent the earlier gestational stage dataset grouped into *N*_E_ clusters, and *X*_L_ denote the later stage dataset grouped into *N*_L_ clusters. Two gene-expression matrices are normalized and log-transformed. The main steps are as follows:

1. We trained a multiclass XGBoost classifier on *X*_E_ using all 300 genes as features. For clusters with fewer than 15,000 cells, we upsampled by bootstrapping to 15,000 cells to make the dataset more balanced. XGBoost classifier parameters were set to the following values: “objective”: “multi:softmax”; “eval_metric”: “mlogloss”; “num_class”: ${N}_{{\rm{E}}}$; “eta”: 0.2; “max_depth”: 20; “subsample”: 0.6; “num_boost_round”: 1000.
2. For validation, we randomly sampled 80% of cells in each cluster of *X*_E_ to train the classifier and predicted the cluster labels for the remaining 20% of cells. For each testing cell *k*, the classifier returns a cluster *a*.
3. Assignment probability vector $p\in {{\mathbb{R}}}^{{N}_{{\rm{E}}}}$ and the final cluster label is assigned as cluster (*k*) = argmax_i_ *p*_i_. For EN-ET cells, the classifiers achieved over 90% accuracy (96.17% between GW15 and GW20, 90.40% between GW20 and GW22, and 92.98% between GW22 and GW34), whereas for EN-IT cells, the accuracy was more than 83% (93.65% between GW15 and GW20, 88.96% between GW20 and GW22, and 83.09% between GW22 and GW34).
4. For prediction, we re-trained the classifier on 100% of cells in *X*_E_ and applied it to *X*_L_ to obtain the predicted label for each cell. The overall correspondence flows between clusters across gestational ages were visualized using Sankey diagrams (Extended Data Fig. 11).

To identify genes exhibiting upregulation or downregulation across gestational ages in one layer-based EN group, we calculated the expressed cell fraction and mean expression for each gene at each time point. We then fit gene-specific linear-regression models for each of these two metrics, using time points as input, for which the time points were encoded as 1, 2, 3 and 4 for GW15, GW20, GW22 and GW34, respectively. Genes were ranked according to the regression coefficient, with the top ten positive ones indicating upregulated genes and the top ten negative ones indicating downregulated genes. The analyses were repeated for each EN group, and the expression patterns of identified genes were visualized in dot plots (Extended Data Fig. 12a). Cluster-specific expression levels of identified genes are shown in Extended Data Fig. 12b.

To pinpoint genes that drive subcluster specification, we first created a pseudobulk for each cluster in the same EN group and gestational age by averaging cell expression. Subsequently, if there was more than one cluster in the same EN group and gestational age pair, we computed the pseudobulk expression variance for each gene. Next, we selected genes with expression variances greater than 1 in at least one EN group and gestational age pair and showed their expression variances using a heatmap (Extended Data Fig. 12d). We also picked the top ten genes with the highest expression variance in each EN group and gestational age pair and visualized their expression patterns as dot plots (Extended Data Fig. 12e,f).

### Visium spatial transcriptomic analysis

Visium spatial gene expression analysis (10x Genomics) was performed according to the manufacturer’s user guide and demonstrated protocol (‘Visium CytAssist Spatial Gene Expression for Fresh Frozen–Methanol Fixation, H&E Staining, Imaging & Destaining, CG000614, Rev A; Visium CytAssist Spatial Gene Expression Reagent Kits User Guide, CG000495, Rev D’). In brief, 10-µm-thick cryosections adjacent to the sections used for MERFISH analyses were adhered to Superfrost slides. Two samples were used for Visium analysis: FB080-O1 (a GW20 occipital cortex sample containing the V1 and V2) and FB121-F1 (a GW20 prefrontal cortex sample). FB080-O1 was coded as A1 and FB121-F1 was coded as D1 in the data-processing and analyses steps.

The tissues were fixed in chilled methanol at −20 °C for 30 min and stained with H&E. The slides were mounted with 85% glycerol in water. H&E staining was imaged at ×20 magnification using a Zeiss Axioscan 7 microscope. After imaging, the slides were placed in Visium tissue slide cassettes with a 6.5 mm gasket and destained with 0.1 N HCL for 15 min at 42 °C. Probe hybridization was performed overnight at 50 °C on a thermal cycler using the provided slide adaptor, followed by post-hybridization buffing. A probe ligation mix was then added into the slide gasket and incubated for 1 h at 37 °C, followed by a post-ligation wash. RNA digestion and tissue removal were performed using a CytAssist instrument (10x Genomics) before probe extension and probe elution. Pre-amplification, SPRIselect and library construction were performed according to the user’s guide. GEX post-library construction quality control was performed using an Agilent 4200 TapeStation System.

Visium libraries were sequenced on a NovaSeq6000 according to recommended parameters by 10x Genomics to the depth of 214–235 million PE150 (paired-end reads with a read length of 150 base pairs) reads. Demultiplexed reads were processed using Space Ranger (v.2.1.0) and Spaceranger-count with default parameters and manual alignment. Visium data analysis was conducted separately on two slices: A1 (FB080-O1) and D1 (FB121-F1). For visualization and clustering, Seurat_5.0.1 and R (v.4.3.1, 2023-06-16) were used on the BCH compute nodes. SCTransform normalization was performed with the ‘spatial’ assay parameter. Optimal clusters were generated, and clusters with negligible cells were removed. Marker identification was performed using FindAllMarkers analysis from the Seurat package using Wilcoxon rank-sum test with the following parameters: (1) only return positive markers; and (2) only test genes that are detected in a minimum fraction of 1% of cells in either of the two populations. This was the default. After these results, we filtered for markers grouped by clusters with an average log_2_[fold change] greater than 1. We generated different visualizations using top genes from this analysis. R (v.4.1.2) along with Seurat_4.1.1 were used for both exploratory analyses and final visualization on a local machine. Spatial feature plots were generated to visualize the expression of genes of interest in the tissue sections.

### Ferret tissue preparation and immunohistochemistry

All procedures on ferrets were performed under protocols approved by the Institutional Animal Care and Use Committee at Boston Children’s Hospital. Ferrets (*Mustela putorius furo*) were obtained from Marshall BioResources and were housed in a vivarium under a 12-h light–dark cycle. Food and water were available ad libitum.

Ferrets were deeply anaesthetized with ketamine (50 mg ml^–1^) and xylazine (20 mg ml^–1^) by intraperitoneal injection and transcardially perfused with 0.9% NaCl solution (w/v) followed by 4% PFA in PBS. Brains were dissected out, post-fixed overnight in 4% PFA in PBS at 4 °C, cryoprotected by serial overnight incubations with 15% and 30% sucrose in PBS and sectioned frozen at 50 µm on a sliding microtome (Leica SM2010 R). Free-floating brain slices were permeabilized with 0.25% Triton X-100 in PBS for 4 times for 15 min each and then blocked in a solution containing 5% BSA (Sigma-Aldrich, 05470), 0.3% Triton X-100, and 10% normal donkey serum (Abcam, ab7475) for 2 h. Brain slices were then incubated overnight at 4 °C with primary antibodies on an orbital shaker.

The next day, slices were washed for 4 times for 15 min each in PBS and then incubated with secondary antibodies for 2 h at room temperature on an orbital shaker. All primary and secondary antibodies were diluted in a solution containing 1% BSA, 0.3% Triton X-100 and 5% normal donkey serum. The following antibodies were used: mouse anti-RORB (1:500, R&D Systems, PP-N7927-00), rabbit anti-NPY (1:500, Abcam, ab30914), rat anti-CTIP2 (1:500, Abcam, ab18465), Alexa Fluor 488 donkey anti-mouse (1:500, Invitrogen), Alexa Fluor 555 donkey anti-rabbit and Alexa Fluor 647 donkey anti-rat. Slices were counterstained with DAPI solution. Slices were mounted on microscope glass slides and coverslipped with Vectashield Vibrance antifade mounting medium. Imaging of ferret brain slices was performed using a LSM980 confocal microscope at 8-bit depth with a ×10 objective and 0.6 digital zoom. Tile scan images were acquired and stitched.

### Fetal macaque tissue preparation and single-molecule fluorescent in situ hybridization

Preserved cryosections of fetal rhesus macaque monkeys at E93 and E76 were shared from a previous study^46^. Macaque fetal brains were dissected and immersed in 4% PFA overnight at 4 °C. Fixed brain blocks were immersed in step-gradients of sucrose–PBS up to 30% for 2–3 days at 4 °C, then embedded in OCT and frozen at −80 °C. Sagittal sections (for E76) and coronal sections (for E93) were cut at 25 μm using a Leica CM3050S cryostat and stored at −80 °C until use. Immunostaining of fetal macaque samples was performed as described above, but with an extra step of antigen retrieval before permeabilization. Antigen retrieval was performed by heating slides in a steam cooker for 30 min in Retrievagen A (pH 6.0) solution (BD Biosciences), followed by two washes of PBS wash. Single-molecule fluorescent in situ hybridization was performed on macaque sections using a RNAscope Multiplex Fluorescent V2 assay (ACD). A custom RNAscope probe for macaque *NPY* was designed and made by ACD (RNAscope Probe, Mmu-NPY). The manufacturer’s standard protocol for multiplex fluorescent in situ hybridization using a HybEZ Hybridization system was followed. Whole tissue mRNA in situ hybridization imaging was performed on a Zeiss LSM 980 confocal microscope with image tiling and stitching, *z* stack of 10 µm and 2× averaging at ×20 magnification.

### Re-analysis of a macaque scRNA-seq dataset

scRNA-seq data (Macaque.dev.rds Seurat object) for macaque fetal cortex^46^ were downloaded from http://resources.sestanlab.org/devmacaquebrain/. The standard workflow for visualization and clustering was executed using Seurat_5.0.1 and R (v.4.3.1). To refine the analysis of ENs, we used the cell_subtype annotations from the pre-processed dataset. Specifically, we subclustered cells labelled as ‘enIPC’ and ‘Excitatory neurons’. This iterative subclustering process continued until we identified clusters exclusive to the V1. In these V1-specific clusters, we assessed the expression patterns of *NPY*, *PDZRN4* and *ABI3BP*. Dimensionality reduction plots were generated to visualize the subclusters, and DGE was performed using the FindAllMarkers function to identify key markers defining each subcluster. To evaluate the representation of the V1 among the identified subclusters, contribution plots were generated, quantifying the proportion of cells from the V1 in each cluster.

### V1-enriched and V2-enriched gene set analysis

To identify sets of genes that are enriched in the V1 or V2 at different ages (GW15, GW20, GW34 and adult), we conducted DEA between V1-specific and V2-specific clusters in EN-ITs and EN-ETs separately at GW15, GW20, GW34 and adult stages (Fig. 5d–g and Supplementary Table 11). Lists of V1-specific and V2-specific clusters are provided below:

- V1-specific clusters in EN-ITs. GW20: ’EN-IT-L3-c1 and ’EN-IT-L3/4-c4. GW34: EN-IT-L3/4-c2, EN-IT-L3/4-c3, EN-IT-L3/4-c4, EN-IT-L3/4-c5, EN-IT-L3/4-c6, EN-IT-L3/4-c7, EN-IT-L4-2-c2 and EN-IT-L4-2-c3. Adult: EN-L4c-c1, EN-L4-c0, EN-L4-c1, EN-L4-c2, EN-L4-c3 and EN-L3-2-c2.
- V2-specific clusters in EN-ITs. GW20: ’EN-IT-L3-c4 and ’EN-IT-L3/4-c1. GW34: EN-IT-L3/4-c1, EN-IT-L3-c0 and EN-IT-L4-2-c1. Adult: EN-L4c-c0 and EN-L3-2-c1.
- V1-specific clusters in EN-ETs. GW20: ’EN-ET-L5/6-c5 and ’EN-ET-SP-2-c2. GW34: EN-ET-L6-c1 and EN-ET-L6-V1-c3. Adult: EN-L2/3-2-c0.
- V2-specific clusters in EN-ETs. GW20: ’EN-ET-L5/6-c4 and ’EN-ET-SP-2-c4. GW34: EN-ET-L5-c1. Adult: EN-L2 | 3-1-c0.

For GW15, no V1-specific or V2-specific clusters were found, and DEA was conducted across the annotated V1–V2 regions. The DEGs were detected using the scanpy.tl.rank_genes_groups() function with *t*-tests. Genes with adjusted *P* < 0.05 and log[fold change] > 0.5 were selected as significant DEG sets. For each annotated V1 or V2 region at every time point, the log[fold change] for the combined expression of the DEG set was calculated, and the mean log[fold changes] were visualized as heatmaps.

### Nucleus isolation and snRNA-seq

Crysoections (100 µm thick) consecutive to the ones collected for MERFISH and Visium analyses were collected in a 1.5 ml tube. Nuclei were isolated from the cryosection as previously described but with minor modifications^69^. In brief, one cryosection per sample was resuspended in 1 ml homogenization buffer with additives (10 mM Tris buffer pH 8.0, 250 mM sucrose, 25 mM KCl, 5 mM MgCl_2_, 0.1% Triton X-100, 0.1 mM DTT, 1× complete, mini, EDTA-free protease inhibitor cocktail (Roche 11836170001) and 25 µl Protector RNAse inhibitor (Roche 3335399001, 0.2 U µl^–1^)) and transferred to a 7 ml douncer and dounced 10 times with a ‘tight’ pestle. Homogenized nuclei were spun for 10 min at 900*g* at 4 °C, then washed once with blocking buffer (1× PBS pH 7.4 and 1% BSA), centrifuging for 5 min at 400*g* at 4 °C. All centrifugations were done in a bucket centrifuge. Nuclei were resuspended in 300 µl blocking buffer with Protector RNAse inhibitor (Roche 3335399001, 1 U µl^–1^) and DAPI (final concentration 1 µg ml^–1^) and passed through a 40 µm filter. DAPI-positive nuclei (13,500 nuclei per 10x reaction) were fluorescence-activated nuclei sorted directly into Chromium Next GEM Single Cell 3′ Reagent Kit v3.1 GEM master mix (Step 1.1) minus RT Enzyme C. RT Enzyme C was then added and reactions loaded into a Chromium Next GEM Chip and snRNA-seq libraries were generated according to the Chromium Next GEM Single Cell 3′ Reagent Kit v.3.1 manual with 11 PCR cycles for cDNA amplification and 11–12 PCR cycles used for final amplification of gene expression libraries. Five reactions were performed for FB080-O1 and three reactions were performed for FB121-F1 samples. Three reactions were performed on FB080-O2, a consecutive tissue block posterior to FB080-O1, near the occipital pole of the brain. snRNA-seq libraries were sequenced on a NovaSeq6000 according to the recommended parameters of 10x Genomics to the depth of 909 million PE150reads for FB080-O1, 541 million PE150 reads for FB080-O2 and 484 million PE150 reads for FB121-F1.

### snRNA-seq processing, clustering and analysis

Demultiplexed reads were processed in Cell Ranger (v.7.2.0) with cellranger-count and default parameters, which resulted in 41,740 estimated cells for FB080-O1, 23,001 estimated cells for FB080-O2 and 26,161 estimated cells for FB121-F1. For visualization and clustering, Seurat_5.0.1 and R (v.4.3.1) were used. Clustering optimization was achieved through iterative application of the FindClusters algorithm to attain optimal cluster resolution. Feature plots were constructed for genes of interest, and integration into the cell-by-gene tool was verified. Contribution plots were generated to observe the contribution of cell types to the overall dataset. To find DEGs between V1-enriched (EN-IT-L4-V1) and V2-enriched (EN-IT-UL-2) clusters, we performed Wilcoxon rank-sum tests at log_2_[fold change] > 0.5 and Bonferroni-corrected *P* < 0.05. Only test genes that were detected in a minimum fraction of 25% of cells in either of the two populations were considered, filtering the genes that were infrequently expressed and only returning positive markers. To verify that we captured DEGs between the V1 and V2, we also identified DEGs between V1 and V2 regions in Merscope, which revealed overlap only between V1-upregulated genes (50%) or V2-upregulated genes (73%) but not vice versa (0%). For GO analysis, we used Bioconductor (v.3.19) to perform GO analyses in R (https://bioconductor.org/packages/release/bioc/html/motifmatchr). Specifically, DEGs upregulated in the V1 or V2 were both tested for GO enrichments using a background of all genes tested for differential expression (odds ratio > 2 and FDR < 0.01).

### ENVI imputation from scRNA-seq to Merscope

We used ENVI (v.0.1.0)^47^ on our MERFISH and snRNA-seq data to expand the MERFISH gene panel. ENVI leverages a conditional variational autoencoder to integrate spatial and snRNA-seq data, which can simultaneously impute missing gene expression for genes that are not included in the spatial transcriptomic data and transfer spatial information onto the snRNA-seq data. We selected the top 1,000 highly variable genes from snRNA-seq, along with the following genes of interest for imputation: *ABI3BP*, *PDZRN4*, *FLRT2*, *TAFA2*, *NR1D1*, *IL1RAP*, *CCBE1*, *THSD7B*, *TRPC6*, *CHRM2*, *LUZP2*, *LRP1B*, *LRRTM4*, *HDAC9*, *FBXL7*, *DTNA*, *SYNDIG1*, *SDK1*, *LMO3*, *TRIQK*, *UNC13C*, *CNTNAP2*, *KCNIP4*, *PDZRN3*, *DLX6*, *DLX6-AS1*, *ADARB2*, *ERBB4*, *NRXN3*, *DLX2*, *ZNF536*, *PRKCA*, *THRB*, *TSHZ1*, *PBX3*, *MEIS2*, *CALB2*, *CDCA7L*, *SYNPR*, *SP8*, *CASZ1* and *FOXP4*. We ran ENVI with the following key parameters: “k_nearest”: 100; “num_cov_genes”: 50; “num_layers”: 3; “num_neurons”: 1024; “latent_dim”: 512; “spatial_dist”: “pois”; “sc_dist”: “nb”; “cov_dist”: “OT”; “prior_dist”: “norm”; “spatial_coeff”: 1; “sc_coeff”: 1; “cov_coeff”: 1; “kl_coeff”: 0.3.

### Confidence interval analysis for imputed gene expression

To quantify the imputation uncertainty, we used transcript imputation with spatial single-cell uncertainty estimation (TISSUE) (v.1.0.1)^48^ to generate the 95% confidence intervals for imputation values. TISSUE assumes that neighbouring cells of the same cell type share similar gene-expression profiles. It defines the cell-centric variability measure and adopts a conformal inference framework to calibrate uncertainties for new expression predictions. We ran TISSUE with the following key parameters: “grouping_method”: “kmeans_gene_cell”, “k”: 4, “k2”: 2, “alpha_level”: 0.05. After obtaining the confidence intervals, we truncated the lower bounds to 0 and computed interval widths. These widths were then visualized for each gene using box plots (Extended Data Fig. 18a).

### Constellation plot analysis

The constellation plot was generated using the ENVI imputation results. We selected all RG, IPC, EN-Mig, EN-ET and EN-IT cells from the V1 and V2 of FB080-O1c and the PFC of FB121-F1. On the basis of the H2 annotations, we combined all EN-ET cells from layers 5 and 6 into a single node (EN-ET L5/6) and did the same for layers 5 and 6 EN-IT cells (EN-IT L5/6). Other EN-ET and EN-IT cells were categorized into nodes according to their H2 annotations, whereas RG and IPC cells were classified by their H1 annotations. Cells were further subclassified on the basis of the sample and annotated region in which they were located. The positions of the cell types on the constellation plot were determined using the median UMAP coordinates of all cells of each type. Node sizes were scaled according to the number of cells of each type. To establish edges between pairs of cell types, we first performed principal component analysis on the ENVI gene expression matrix. Using the top 50 principal components, we then identified the 15 nearest neighbours for each cell by applying the Ball Tree algorithm. For any given pair of cell types, A and B, we added a connection from A to B if a cell in A had a nearest neighbour in B. We then calculated the total number of connections from *A* to *B* (*n*_A→B_) and the total number of connections from A to all types (*n*_A_). An edge was drawn between A and B on the constellation plot if both *n*_A→B_/*n*_A_ and *n*_B→A_/*n*_u_ exceeded 0.02. The edge from A to B was coloured according to the proportion of connections from A to B relative to the total connections from A (*n*_A→B_/*n*_A_).

### Cell–cell communication analysis

We used the CellChat package (v.1.6.1) to decipher cell–cell communication networks using its extensive database of known human ligand–receptor interactions. To assess the communication probability, we followed the methodologies outlined in the original publication^49^. This method applies the law of mass action to the average expression levels of ligands and receptors across annotated cell groups and evaluates their significance using the random permutation statistical test. Interactions with *P* *<* 0.05 were considered significant and those identified in fewer than ten cells in each cell group were discarded to enhance the robustness of our findings. For this study, we analysed all cell types available in our dataset but specifically focused on EN-IT-L4-V1, EN-IT-UL-1, EN-IT-UL-2, EN-IT-UL-3, EN-IT-UL-4 and EN-IT-UL-5 as receivers in the NRXN pathway. Visualization of the communication networks and pathways was performed using the native plotting functions in CellChat, which facilitated a comprehensive representation of the cellular interactions in our dataset.

### Interactive web-based browser for MERFISH data

To create a web browser of our MERFISH data, we used the WebAtlas pipeline, which leverages Vitessce (v.1.0.15) to visualize spatial transcriptomic data, in the cloud-optimized Zarr format and the data are uploaded to Amazon Web Service (AWS) S3 buckets for easy access and retrieval^70^. WebAtlas retrieves the data directly from S3 using its web portal hosted by the Wellcome Sanger Institute, which enables seamless integration and efficient visualization by leveraging cloud storage. WebAtlas provides interactive visualizations including UMAP plots, cell-type annotation and gene-expression distributions, all powered by AWS S3 cloud storage. Examples of visualization can be found at the links below:

FB121-F1 (GW20 PFC experiment): https://webatlas.cog.sanger.ac.uk/dev/index.html?theme=dark&config=https://f1-fb121.s3.us-east-1.amazonaws.com/Merfish_F1_FB121/0.5.2/F1_FB121-scRNAseq-config.json.

FB123-F1 (GW22 PFC experiments): https://webatlas.cog.sanger.ac.uk/dev/index.html?theme=dark&config=https://f1-fb123.s3.us-east-1.amazonaws.com/Merfish_F1_FB123/0.5.2/F1_FB123-scRNAseq-config.json.

### Statistics and reproducibility

Replicates and statistical tests are described in the figure legends and Methods. Sample sizes were not predetermined using statistical methods. Tissue samples were not randomized, nor were the investigators blinded during collection as no subjective measurements were taken. Data for snRNA-seq, Visium and MERFISH were collected from all available samples and no randomization was necessary. To identify DEGs between clusters, Wilcoxon rank-sum tests were performed.

### Reporting summary

Further information on research design is available in the Nature Portfolio Reporting Summary linked to this article.

## Online content

Any methods, additional references, Nature Portfolio reporting summaries, source data, extended data, supplementary information, acknowledgements, peer review information; details of author contributions and competing interests; and statements of data and code availability are available at 10.1038/s41586-025-09010-1.

## Supplementary information

Supplementary Information This file contains Supplementary Fig. 1 (sorting strategies used to isolate single nuclei for snRNA-seq) and the legends for Supplementary Tables 1–13.

Reporting Summary

Supplementary Tables This file contains Supplementary Tables 1–13.

Supplementary Data Source Data Fig. 3: Expression pattern heatmap for all 300 genes in the MERFISH.

## Data Availability

Processed MERFISH objects, snRNA-seq data and Visium data used for performing the analysis shown in the figures in the manuscript are available from Zenodo (10.5281/zenodo.14422018)^71^. MERFISH data are available from Zenodo (10.5281/zenodo.15127709)^72^. An interactive web browser for MERFISH data is available at: https://walshlab.org/research/cortexdevelopment. Raw sequencing data from Visium were deposited in the Sequence Read Archive database with BioProject accession number PRJNA1231045. Three previously published human fetal brain scRNA-seq datasets are available from the DbGaP (phs000989.v3.p1)^7^, the Gene Expression Omnibus (GSE162170)^8^ and the NeMO Archive (RRID: SCR_002001)^6^. Fetal macaque scRNA-seq data are available from the Gene Expression Omnibus (GSE226451). Other raw data that support the findings of this study are available from the lead contact C.A.W. upon reasonable request.

## Code availability

Codes for Merscope processing and CellPose nucleus-segmentation pipelines are available from GitHub (https://github.com/carsen-stringer/vizgen-postprocessing). Codes used for data analysis in this article is available at from GitHub (https://github.com/ShunzhouJiang/Spatial-Single-cell-Analysis-of-Human-Cortical-Layer-and-Area-Specification).
