# Supplementary material for: Spatial transcriptomics reveals human cortical layer and area specification
Source: Nature. 2025 May 14;644(8075):153–63. doi: 10.1038/s41586-025-09010-1 (PMC12328223; doi:10.1038/s41586-025-09010-1)
Supplement: Supplementary file 1 — This file contains Supplementary Fig. 1 (sorting strategies used to isolate single nuclei for snRNA-seq) and the legends for Supplementary Tables 1–13. [file 41586_2025_9010_MOESM1_ESM.pdf]

nature portfolio

https://doi.org/10.1038/s41586-025-09010-1

# Supplementary information

# Spatial transcriptomics reveals human cortical layer and area specification

In the format provided by the authors and unedited

## Suppelmentary Figure

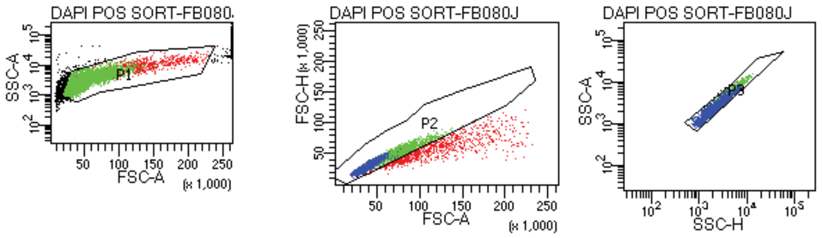


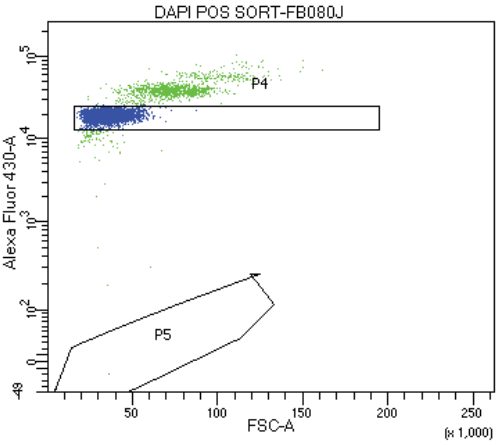


Tube: FB080J

| Population | #Events | %Parent | %Total |
|---|---|---|---|
| All Events | 8,883 | #### | 100.0 |
| P1 | 8,222 | 92.6 | 92.6 |
| P2 | 7,319 | 89.0 | 82.4 |
| P3 | 7,313 | 99.9 | 82.3 |
| P4 | 6,015 | 82.3 | 67.7 |
| P5 | 1 | 0.0 | 0.0 |

**Suppelmentary Figure 1. Sorting strategies used for single nucleus used in snRNAseq.**

Representative fluorescence-activated nuclear sorting (FANS) using Dapi to label 2N nuclei for separation from debris and multiplet nuclei. Gate P4 shows Dapi-positive 2N nuclei sequenced for this study.

**Supplementary Table 1:**
Glossary of acronyms.

**Supplementary Table 2:**
Meta information table for samples used in the study.

**Supplementary Table 3:**
MERFISH 300 gene panel list with annotation for selection criterion.

**Supplementary Table 4:**
Merscope encoding probe design for the 300 and 960 gene panels.

**Supplementary Table 5:**
Cluster annotations for integrated clustering.

**Supplementary Table 6:**
Differentially expressed genes between anteriorly and posteriorly enriched EN subtypes.

**Supplementary Table 7:**
Annotation for clustering by individual gestational age.

**Supplementary Table 8:**
MERFISH 960 gene panel used for additional experiments included in revision.

**Supplementary Table 9:**
Annotations for additional experiments clustered individually.

**Supplementary Table 10:**
Differentially expressed genes between V1 and V2 EN subtypes and their overlaps from MERFISH analysis.

**Supplementary Table 11:**
List of differentially expressed genes between V1 and V2 at different gestational ages.

**Supplementary Table 12:**
Connection fraction values in constellation plot.

**Supplementary Table 13:**
Differentially expressed genes between EN-IT-L4-V1 and EN-IT-UL-2 clusters from snRNAseq, used for GO analysis.
