# Supplementary material for: Spatial transcriptomics reveals human cortical layer and area specification
Source: Nature. 2025 May 14;644(8075):153–63. doi: 10.1038/s41586-025-09010-1 (PMC12328223; doi:10.1038/s41586-025-09010-1)
Supplement: Supplementary file 2 — Reporting Summary [file 41586_2025_9010_MOESM2_ESM.pdf]

nature portfolio

Corresponding author(s): Christopher A. Walsh

Last updated by author(s): Mar 5, 2025

Reporting Summary

Nature Portfolio wishes to improve the reproducibility of the work that we publish. This form provides structure for consistency and transparency in reporting. For further information on Nature Portfolio policies, see our Editorial Policies and the Editorial Policy Checklist.

Please do not complete any field with "not applicable" or n/a. Refer to the help text for what text to use if an item is not relevant to your study.
For final submission: please carefully check your responses for accuracy; you will not be able to make changes later.

Statistics

For all statistical analyses, confirm that the following items are present in the figure legend, table legend, main text, or Methods section.

n/a	Confirmed	
☐	☒	The exact sample size (n) for each experimental group/condition, given as a discrete number and unit of measurement
☐	☒	A statement on whether measurements were taken from distinct samples or whether the same sample was measured repeatedly
☐	☒	The statistical test(s) used AND whether they are one- or two-sided<br>*Only common tests should be described solely by name; describe more complex techniques in the Methods section.*
☐	☒	A description of all covariates tested
☐	☒	A description of any assumptions or corrections, such as tests of normality and adjustment for multiple comparisons
☐	☒	A full description of the statistical parameters including central tendency (e.g. means) or other basic estimates (e.g. regression coefficient) AND variation (e.g. standard deviation) or associated estimates of uncertainty (e.g. confidence intervals)
☐	☒	For null hypothesis testing, the test statistic (e.g. F, t, r) with confidence intervals, effect sizes, degrees of freedom and P value noted<br>*Give P values as exact values whenever suitable.*
☒	☐	For Bayesian analysis, information on the choice of priors and Markov chain Monte Carlo settings
☒	☐	For hierarchical and complex designs, identification of the appropriate level for tests and full reporting of outcomes
☒	☐	Estimates of effect sizes (e.g. Cohen's d, Pearson's r), indicating how they were calculated

Our web collection on statistics for biologists contains articles on many of the points above.

Software and code

Policy information about availability of computer code

Data collection	The following packages and software were used in data collection: Vizgen Merscope software (release version 232), Cell Ranger (7.2.0), Space Ranger (2.1.0)
Data analysis	The following packages and software were used in data analysis: Fiji (2.15.1), Adobe Photoshop (m.2506), BioGrids (2.9.9), Merscope Visualizer (2.1.2595.1), Vizgen Post-processing Tool (1.2.0), CellPose (2.0), SingleR (v.1.8.1), Scanpy (1.8.2), single-cell significant hierarchical clustering (scSHC v.0.1.0), TISSUE (v.1.0.1), XGBoost (2.0.3), Seurat (5.0.1&4.1.1), R (4.3.1&4.1.2), ENVI (v.0.1.0), CellChat (1.6.1), Bioconductor (3.19), Vitessce (1.0.15).<br>Codes for Merscope Processing and Cellpose cell segmentation pipelines are available at: https://github.com/carsen-stringer/vizgen-postprocessing. Code used for data analysis in this manuscript is available at GitHub: https://github.com/ShunzhouJiang/Spatial-Single-cell-Analysis-of-Human-Cortical-Layer-and-Area-Specification

For manuscripts utilizing custom algorithms or software that are central to the research but not yet described in published literature, software must be made available to editors and reviewers. We strongly encourage code deposition in a community repository (e.g. GitHub). See the Nature Portfolio guidelines for submitting code & software for further information.

Data

Policy information about availability of data

All manuscripts must include a data availability statement. This statement should provide the following information, where applicable:

- Accession codes, unique identifiers, or web links for publicly available datasets
- A description of any restrictions on data availability
- For clinical datasets or third party data, please ensure that the statement adheres to our policy

Processed MERFISH objects, snRNAseq data and Visium data used for performing the analysis shown in the figures in the manuscript are available on Zenodo: https://zenodo.org/records/14422018. MERFISH data are available on Zenodo: https://zenodo.org/records/14941391. An interactive web browser for MERFISH data is available at: https://walshlab.org/research/cortexdevelopment. Raw sequencing data from Visium were deposited in the SRA databased with BioProject accession number PRJNA1231045. Three previously published human fetal brain scRNA-seq datasets are available at: DbGaP: phs000989.v3.p1 (Nowakowski et al.); GEO: GSE162170 (Trevino et al.); and NeMO Archive: RRID:SCR_002001 (Bhaduri et al). Fetal macaque scRNA-seq data is available at GEO: GSE226451. Other raw data that support the findings of this study are available from the lead contact Dr. Christopher A. Walsh (Christopher.Walsh@childrens.harvard.edu) upon reasonable request.

Research involving human participants, their data, or biological material

Policy information about studies with human participants or human data. See also policy information about sex, gender (identity/presentation), and sexual orientation and race, ethnicity and racism.

Reporting on sex and gender	The human brain tissue samples were from 5 male and 5 female individuals. Sex-based analysis was not performed due to the scarcity of rare samples, and due the the general consensus that prenatal brain development is not significantly different between sex.
Reporting on race, ethnicity, or other socially relevant groupings	No socially relevant categorization variables were involved in the study.
Population characteristics	Human brain tissue samples from 10 deidentified individuals from gestational weeks 15, 18, 20, 21, 22, 34 and adult were used. No demographic information was collected, including gender, genotype or other treatment history and thus was not analyzed in any co-variate manner.
Recruitment	De-identified tissue samples were collected with previous patient consent in strict observance of the legal and institutional ethical regulations. This was performed by the clinic and no recruitment criteria were used. Because we have no demographic information about either our samples or the patient population, we cannot comment on how any bias may or may not be present.
Ethics oversight	Research performed on samples of human origin was conducted according to protocols approved by the institutional review boards (IRB) of Boston Children's Hospital and Beth Israel Deaconess Medical Center.

Note that full information on the approval of the study protocol must also be provided in the manuscript.

Field-specific reporting

Please select the one below that is the best fit for your research. If you are not sure, read the appropriate sections before making your selection.

☒ Life sciences ☐ Behavioural & social sciences ☐ Ecological, evolutionary & environmental sciences

Life sciences study design

All studies must disclose on these points even when the disclosure is negative.

Sample size	Due to the scarcity of intact human fetal samples, we included 10 individuals with a representative coverage of developmental time points and brain regions. Sample size was determined by availability of rare samples, cost and labor of experiments, scale of analysis. No sample calculation analysis was performed. These samples were sufficient because all expected cell types were identified.
Data exclusions	For each MERFISH experiments, we filtered out all cells with a total transcript count below the tenth percentile to enrich for high-quality cells. In addition, we noticed that a few clusters exhibited aberrant spatial distribution reflecting technical artifact during imaging process. These clusters, easily recognizable with exclusive localization at the edge of certain tissue sections or surrounding bubbles within the tissue section, were the accidental result of tissue hydrogel detaching from the surface during imaging. We removed cells from these artifact clusters from all subsequent analysis. However, these excluded cells are contained in the raw dataset. Exclusion criteria were pre-determined based upon published metrics from similar studies.
Replication	For biological replicates, two independent samples were analyzed for GW15; three independent samples were analyzed for GW20. For technical replicates, 6 out of the 47 MERFISH experiments were replicated on consecutive tissue section (see Supplementary Table 2). Replicated experiments were performed independently.

	Whether biological replicate is necessary was determined by importance of validating the biological findings. For example, to support our finding on the V1-V2 border at GW20, 3 technical replicates were performed on consecutive sections from one sample, and 2 independent experiment was performed on two other samples of the same age and area. All attempts at replication were successful.<br>Single-nucleus RNA-seq analysis include cells from at least three tissue sections from each sample processed in two separate experiments. All attempts at replication were successful.<br>Experiments other than those mentioned in the reporting summary were not replicated
Randomization	No randomization was used. Covariates were not controlled and are not relevant to this study because no covariates were statistically analyzed.
Blinding	No blinding was used. Blinding was not used because all analysis was performed on all relevant samples in reproducible ways so no bias could have been removed by blinding.

Reporting for specific materials, systems and methods

We require information from authors about some types of materials, experimental systems and methods used in many studies. Here, indicate whether each material, system or method listed is relevant to your study. If you are not sure if a list item applies to your research, read the appropriate section before selecting a response.

Materials & experimental systems

n/a	Involved in the study	
☐	☒	Antibodies
☒	☐	Eukaryotic cell lines
☒	☐	Palaeontology and archaeology
☐	☒	Animals and other organisms
☒	☐	Clinical data
☒	☐	Dual use research of concern
☒	☐	Plants

Methods

n/a	Involved in the study	
☒	☐	ChIP-seq
☐	☒	Flow cytometry
☒	☐	MRI-based neuroimaging

Antibodies

Antibodies used	Primary antibodies used were rat anti-CTIP2 (Abcam, ab18465), mouse anti-SATB2 (Abcam, ab9244), and rabbit anti-TBR1 (Abcam, ab31940), mouse anti-RORB (1:500, R&D Systems, PP-N7927-00), rabbit anti-NPY (1:500, Abcam, ab30914), goat anti-synaptophysin (R&D Systems, AF5555-SP)
Validation	All antibodies are validated by manufacture for immunohistochemistry on human cells.

Animals and other research organisms

Policy information about studies involving animals; ARRIVE guidelines recommended for reporting animal research, and Sex and Gender in Research

Laboratory animals	Ferrets (Mustela putorius furo) were obtained from Marshall BioResources. Ferret kits at postnatal days 2 and 9 were analyzed in this study at postnatal.
Wild animals	No wild animals were used in the study
Reporting on sex	Male ferret kits were analyzed in this study. Sex was not considered in study design.
Field-collected samples	No field collected samples were used in the study
Ethics oversight	All ferret procedures were performed under protocols approved by the Institutional Animal Care and Use Committee at Boston Children's Hospital.

Note that full information on the approval of the study protocol must also be provided in the manuscript.

Plants

Seed stocks	*Report on the source of all seed stocks or other plant material used. If applicable, state the seed stock centre and catalogue number. If plant specimens were collected from the field, describe the collection location, date and sampling procedures.*
Novel plant genotypes	*Describe the methods by which all novel plant genotypes were produced. This includes those generated by transgenic approaches, gene editing, chemical/radiation-based mutagenesis and hybridization. For transgenic lines, describe the transformation method, the number of independent lines analyzed and the generation upon which experiments were performed. For gene-edited lines, describe the editor used, the endogenous sequence targeted for editing, the targeting guide RNA sequence (if applicable) and how the editor was applied.*
Authentication	*Describe any authentication procedures for each seed stock used or novel genotype generated. Describe any experiments used to assess the effect of a mutation and, where applicable, how potential secondary effects (e.g. second site T-DNA insertions, mosiacism, off-target gene editing) were examined.*

Flow Cytometry

Plots

Confirm that:

☒ The axis labels state the marker and fluorochrome used (e.g. CD4-FITC).

☒ The axis scales are clearly visible. Include numbers along axes only for bottom left plot of group (a 'group' is an analysis of identical markers).

☒ All plots are contour plots with outliers or pseudocolor plots.

☒ A numerical value for number of cells or percentage (with statistics) is provided.

Methodology

Sample preparation	Nuclei were isolated from frozen post-mortem human brain tissue as follows. 100µm-thick cryosections of tissue were resuspended in 5mL nuclei isolation media with additives (10mM Tris Buffer pH 8.0, 250mM Sucrose, 25mM KCl, 5mM MgCl2, 0.1% Triton X-100, 0.1mM DTT, 1X cOmplete™, Mini, EDTA-free Protease Inhibitor Cocktail (Roche 11836170001)) in a 7mL douncer and dounced 10-15 times with a 'loose' pestle and 10-15 times with a 'tight' pestle. Dounced nuclei were passed through a 40um filter and spun for 10min at 900g, then resuspended in 2mL Immunostaining Buffer (1X PBS pH 7.4, 1% BSA, 1:1000 dilution NeuN-488). Nuclei were stained for 20min and then centrifuged for 5min at 400g. All spins were done in a bucket centrifuge. Nuclei were resuspended in 1mL Blocking Buffer with Dapi (final concentration 1ug/ml) and passed through a 40um filter before downstream processing.
Instrument	BD FACSAria
Software	BD FACSAria
Cell population abundance	We used flow cytometry for single nuclei isolation, no particular cell types were targeted.
Gating strategy	We used flow cytometry for single nuclei isolation, no particular cell types were gated

☐ Tick this box to confirm that a figure exemplifying the gating strategy is provided in the Supplementary Information.

This checklist template is licensed under a Creative Commons Attribution 4.0 International License, which permits use, sharing, adaptation, distribution and reproduction in any medium or format, as long as you give appropriate credit to the original author(s) and the source, provide a link to the Creative Commons license, and indicate if changes were made. The images or other third party material in this article are included in the article's Creative Commons license, unless indicated otherwise in a credit line to the material. If material is not included in the article's Creative Commons license and your intended use is not permitted by statutory regulation or exceeds the permitted use, you will need to obtain permission directly from the copyright holder. To view a copy of this license, visit http://creativecommons.org/licenses/by/4.0/
