# Supplementary figures and images for: Spatial transcriptomics reveals human cortical layer and area specification (part 2 of 2)
Source: Nature. 2025 May 14;644(8075):153–63. doi: 10.1038/s41586-025-09010-1 (PMC12328223; doi:10.1038/s41586-025-09010-1)

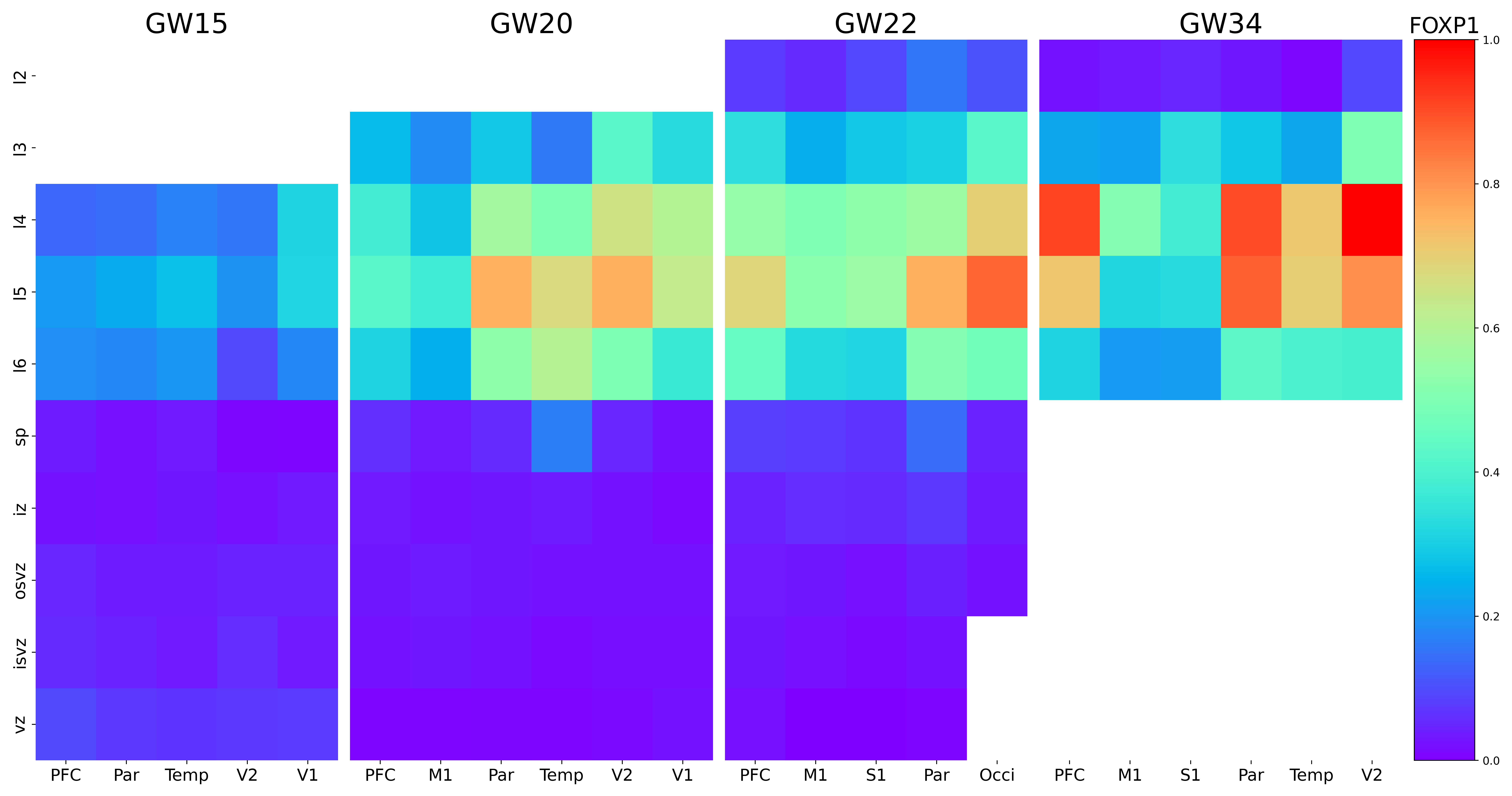

Supplement: Supplementary file 4 — Source Data Fig. 3: Expression pattern heatmap for all 300 genes in the MERFISH. [file 41586_2025_9010_MOESM4_ESM.zip › FOXP1.png]

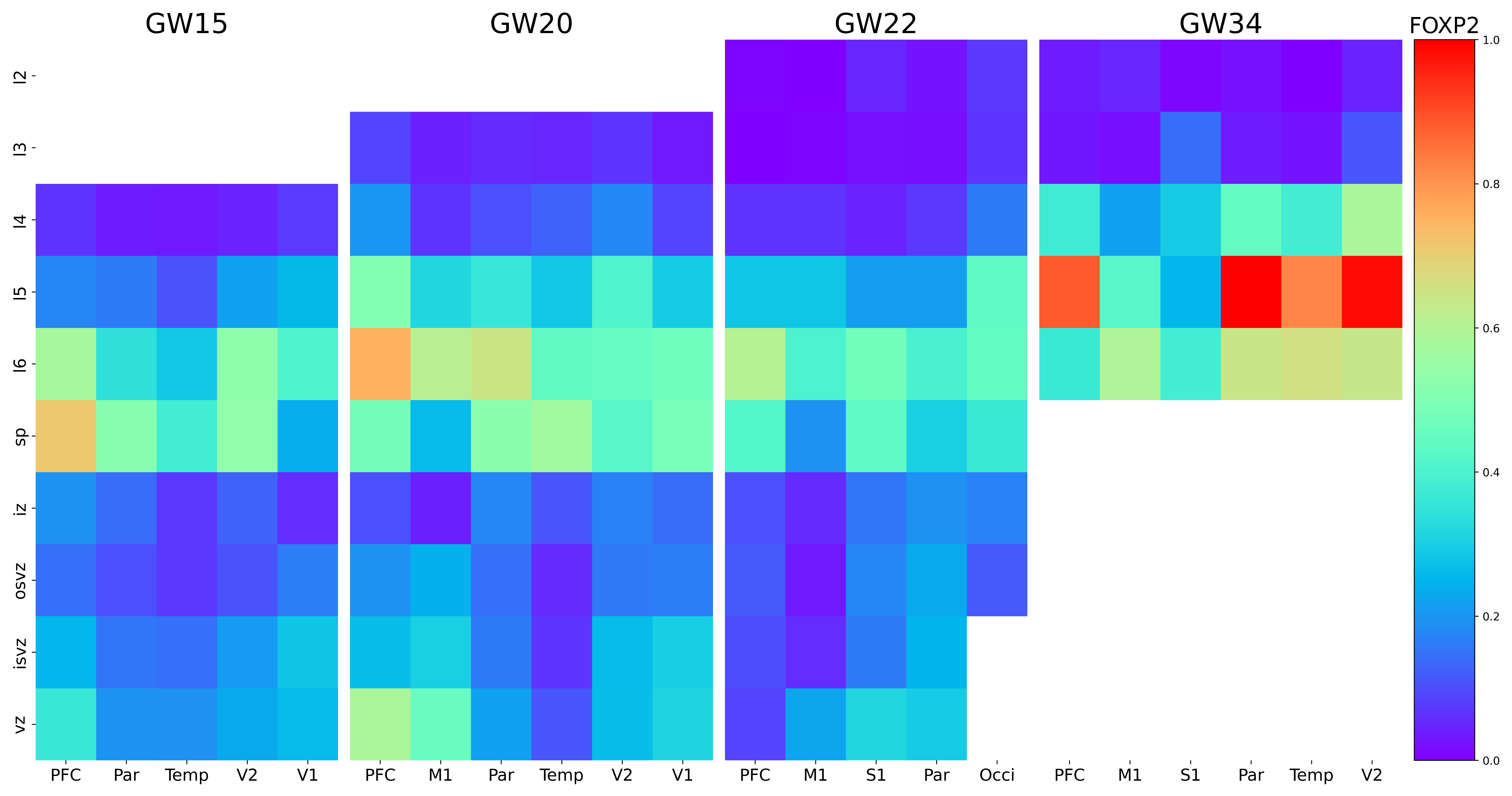

Supplement: Supplementary file 4 — Source Data Fig. 3: Expression pattern heatmap for all 300 genes in the MERFISH. [file 41586_2025_9010_MOESM4_ESM.zip › FOXP2.png]

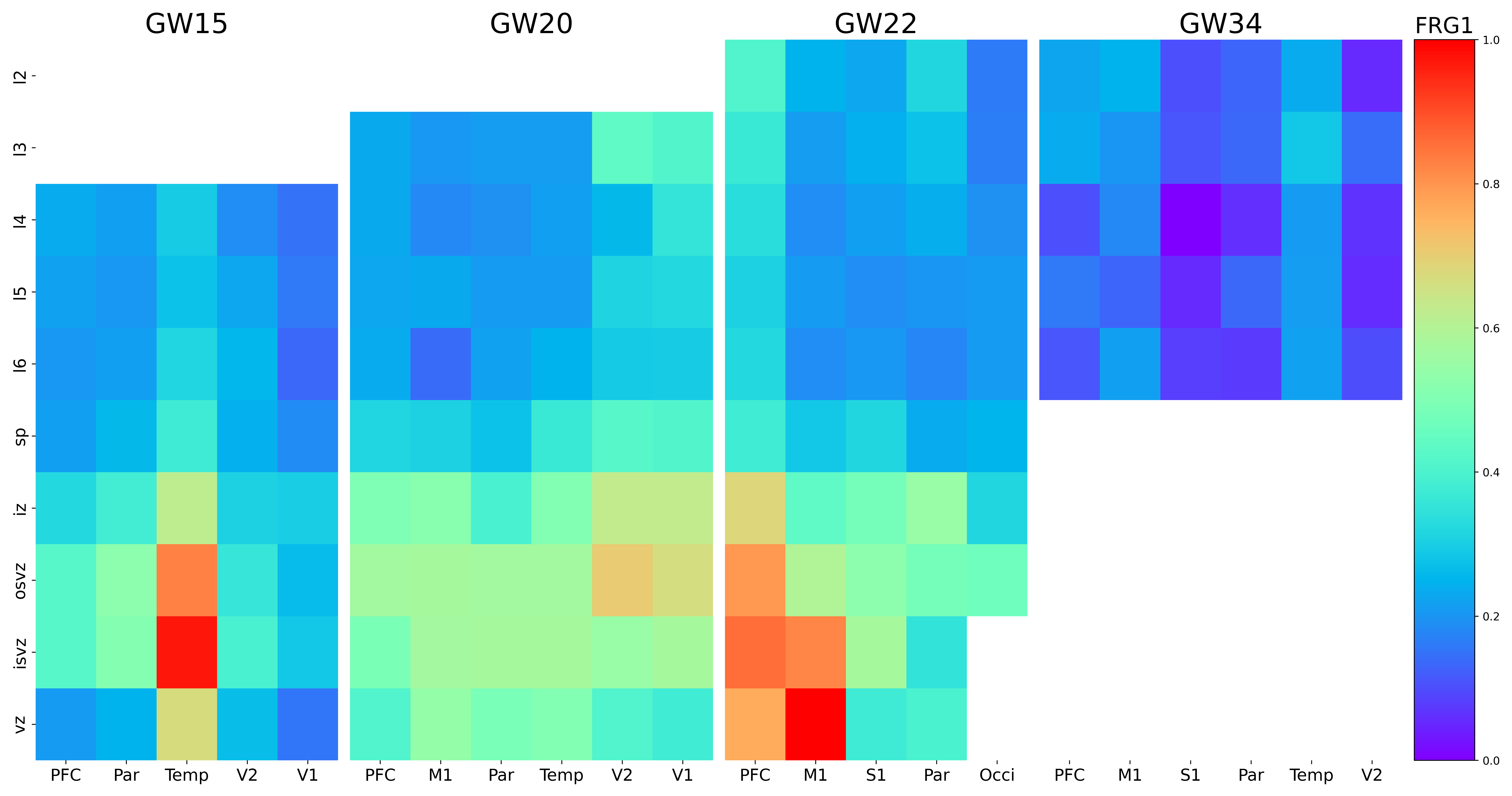

Supplement: Supplementary file 4 — Source Data Fig. 3: Expression pattern heatmap for all 300 genes in the MERFISH. [file 41586_2025_9010_MOESM4_ESM.zip › FRG1.png]

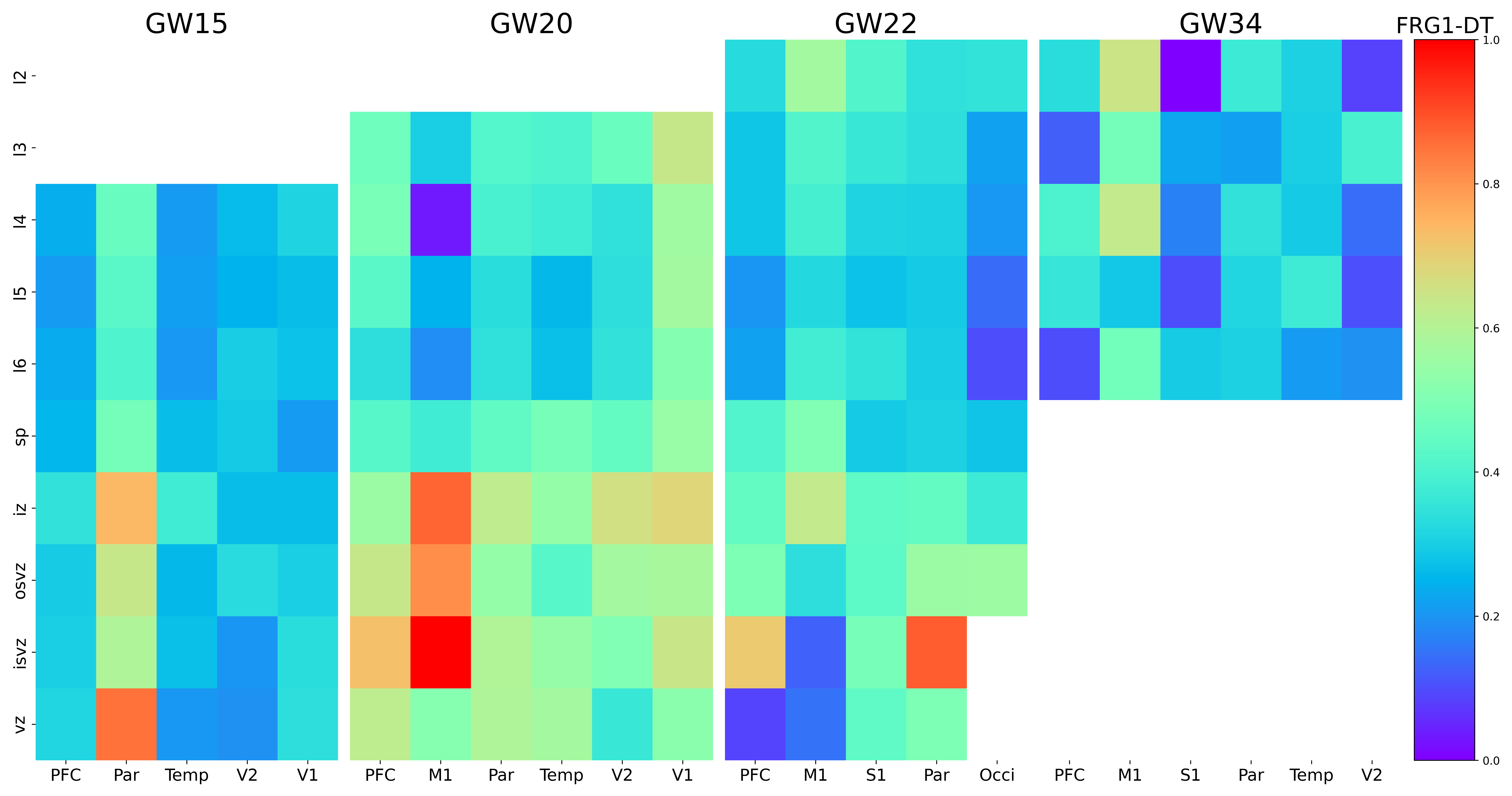

Supplement: Supplementary file 4 — Source Data Fig. 3: Expression pattern heatmap for all 300 genes in the MERFISH. [file 41586_2025_9010_MOESM4_ESM.zip › FRG1-DT.png]

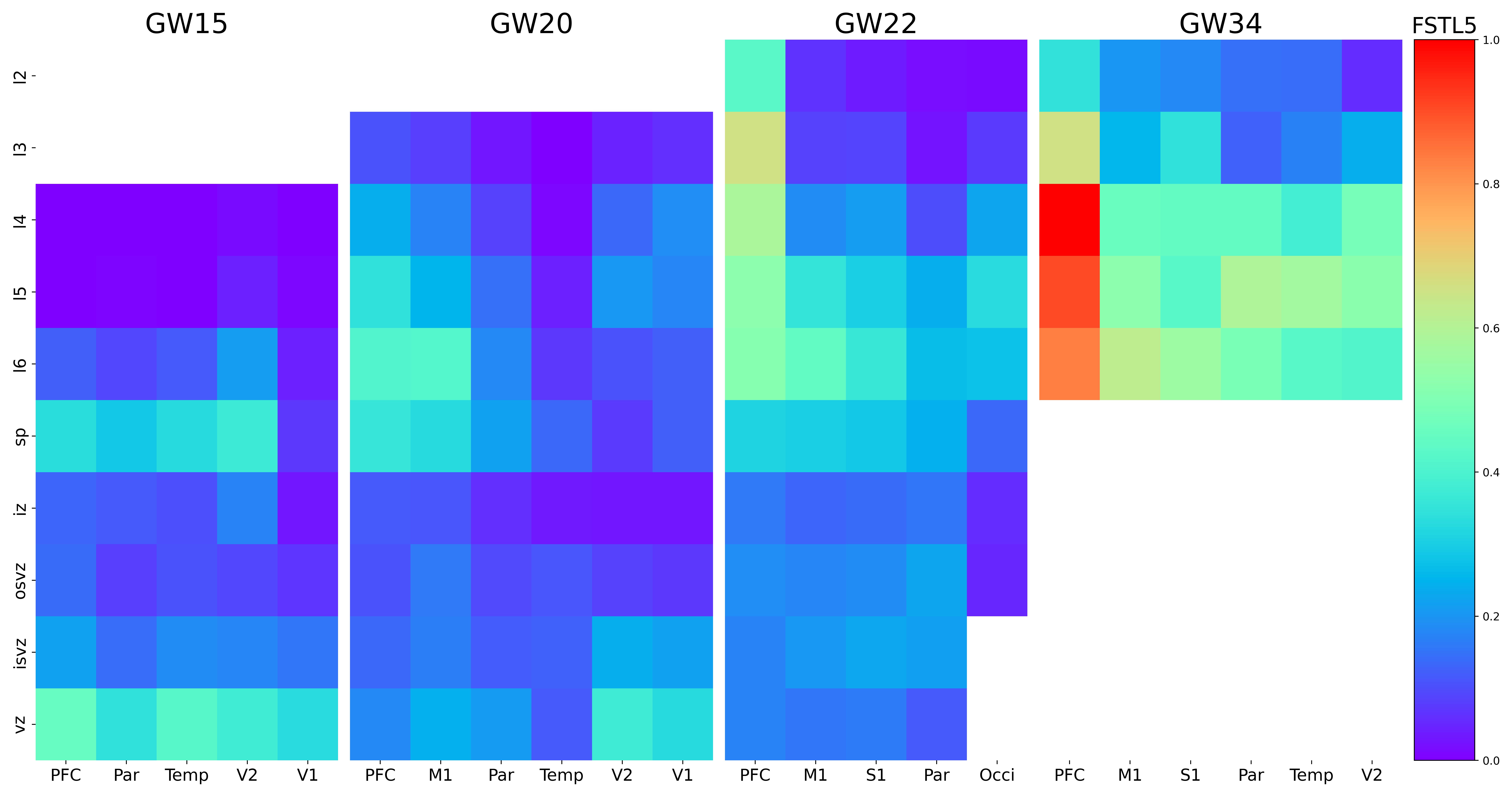

Supplement: Supplementary file 4 — Source Data Fig. 3: Expression pattern heatmap for all 300 genes in the MERFISH. [file 41586_2025_9010_MOESM4_ESM.zip › FSTL5.png]

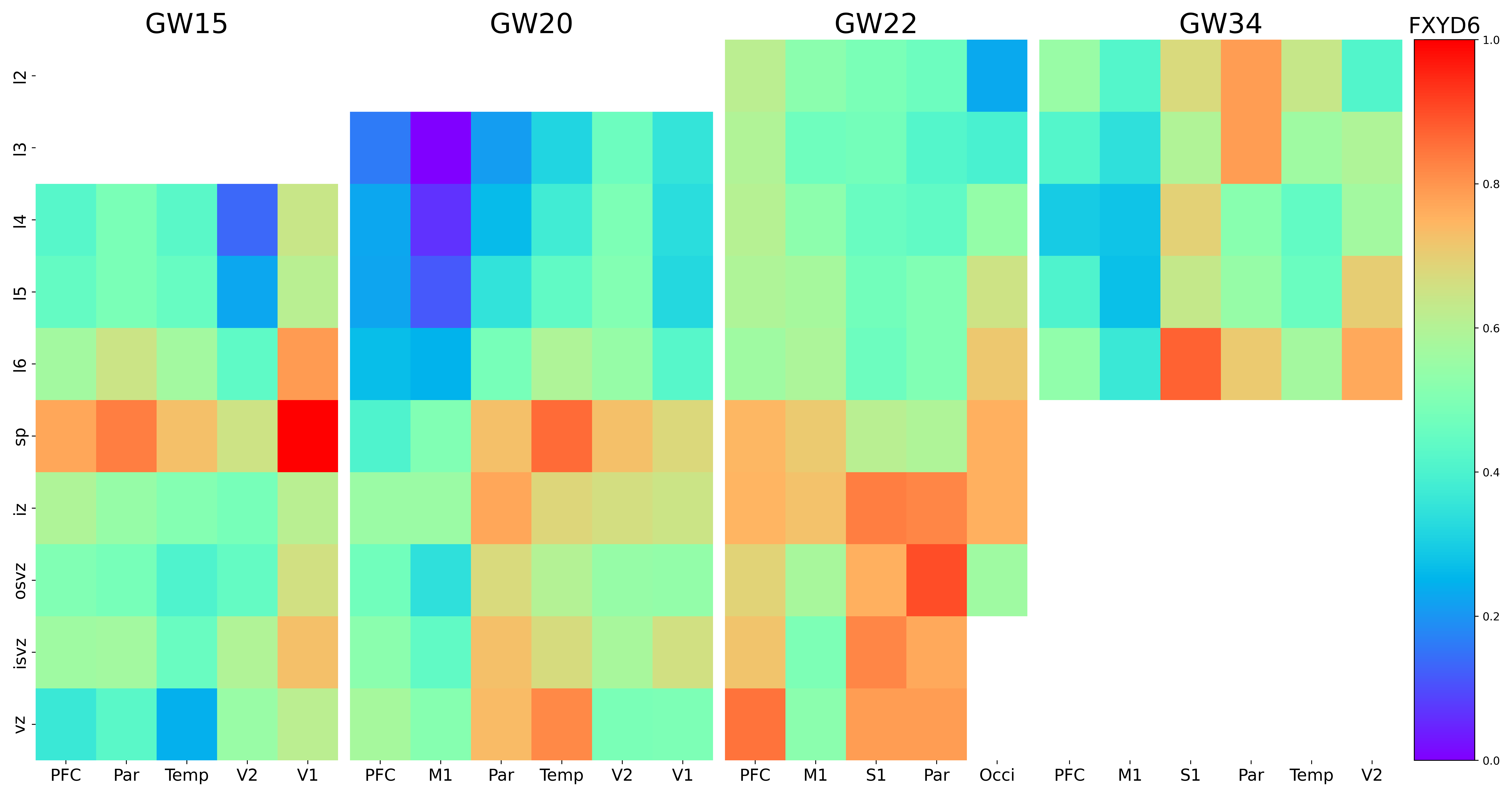

Supplement: Supplementary file 4 — Source Data Fig. 3: Expression pattern heatmap for all 300 genes in the MERFISH. [file 41586_2025_9010_MOESM4_ESM.zip › FXYD6.png]

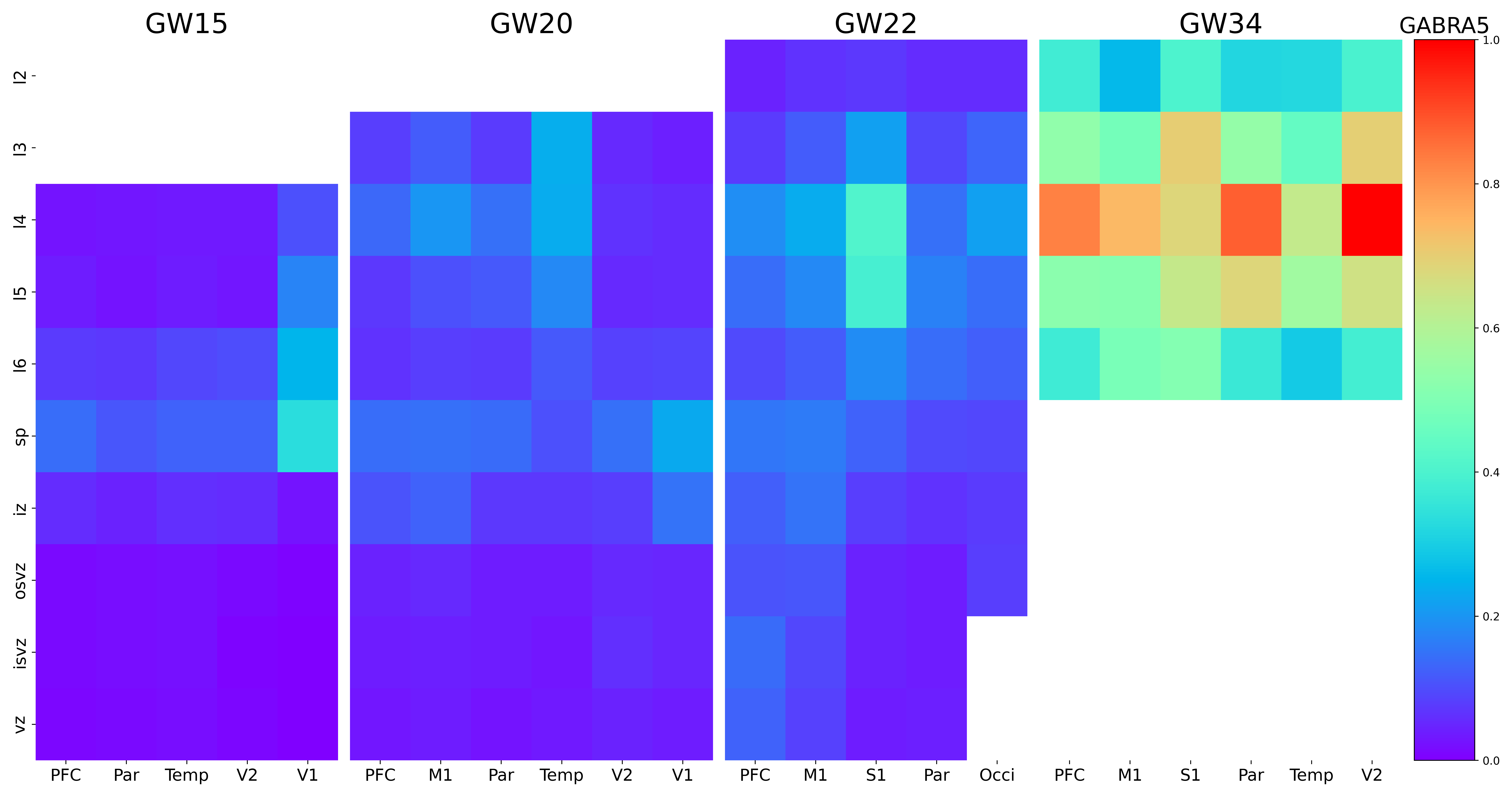

Supplement: Supplementary file 4 — Source Data Fig. 3: Expression pattern heatmap for all 300 genes in the MERFISH. [file 41586_2025_9010_MOESM4_ESM.zip › GABRA5.png]

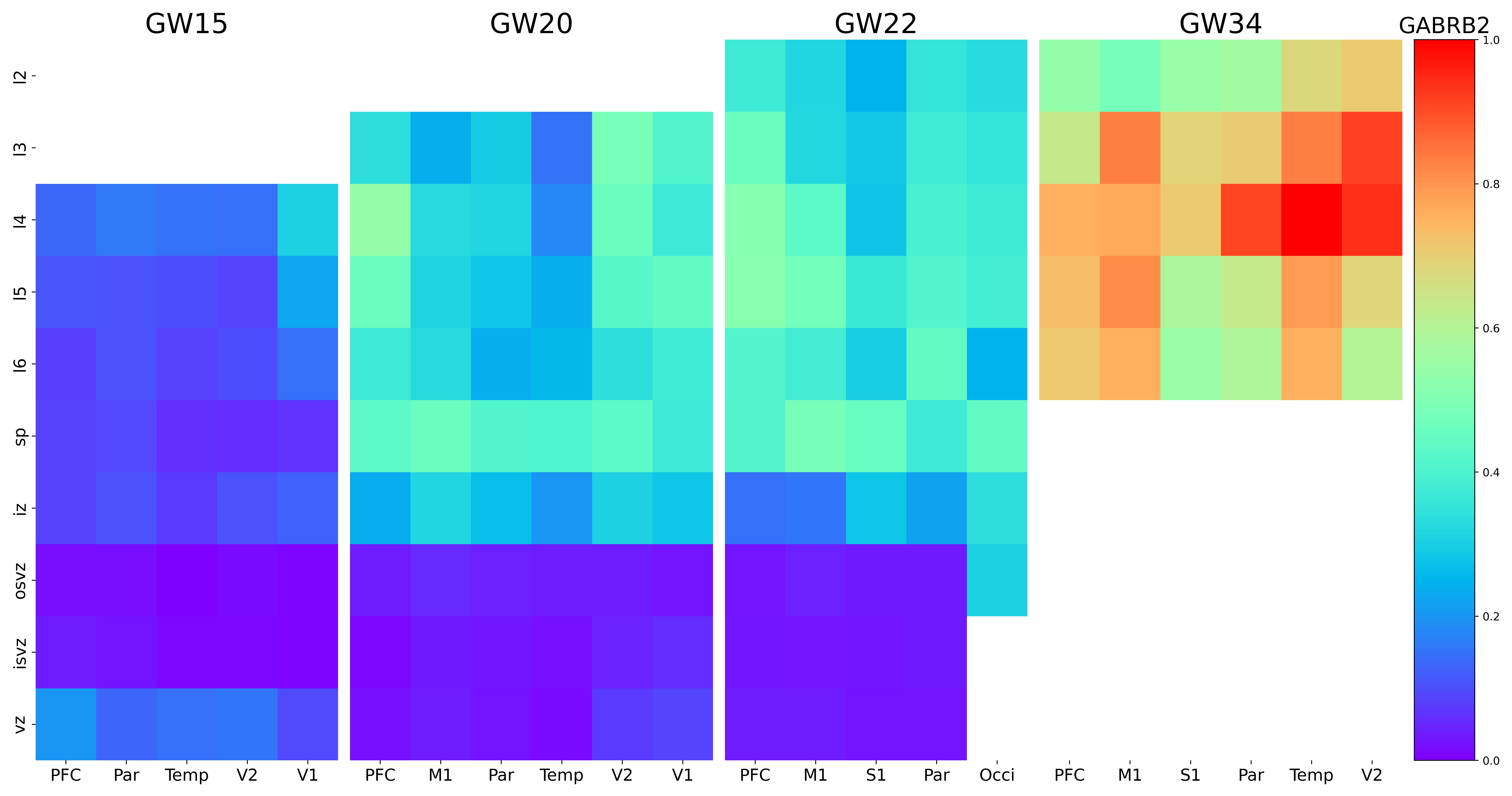

Supplement: Supplementary file 4 — Source Data Fig. 3: Expression pattern heatmap for all 300 genes in the MERFISH. [file 41586_2025_9010_MOESM4_ESM.zip › GABRB2.png]

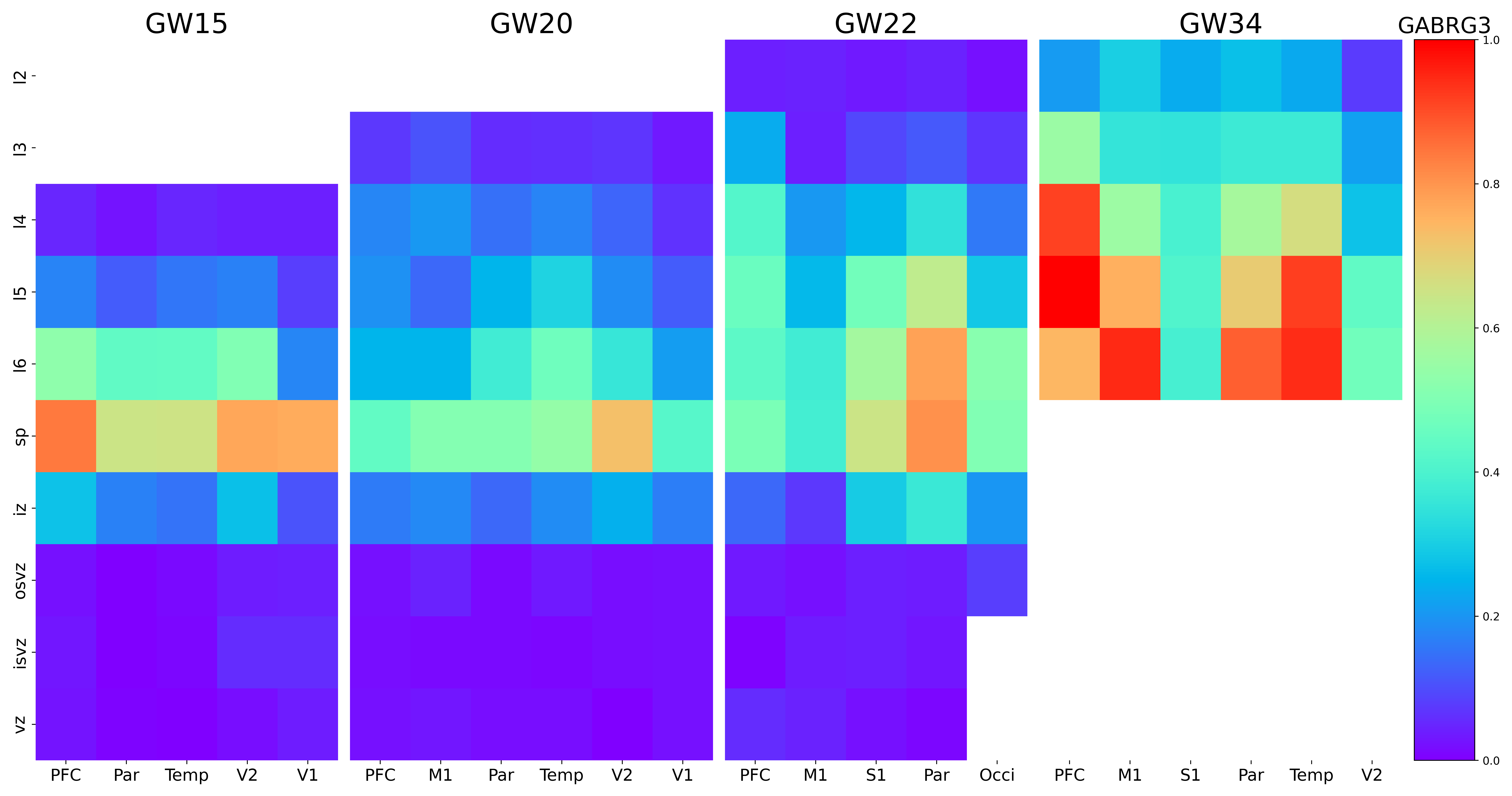

Supplement: Supplementary file 4 — Source Data Fig. 3: Expression pattern heatmap for all 300 genes in the MERFISH. [file 41586_2025_9010_MOESM4_ESM.zip › GABRG3.png]

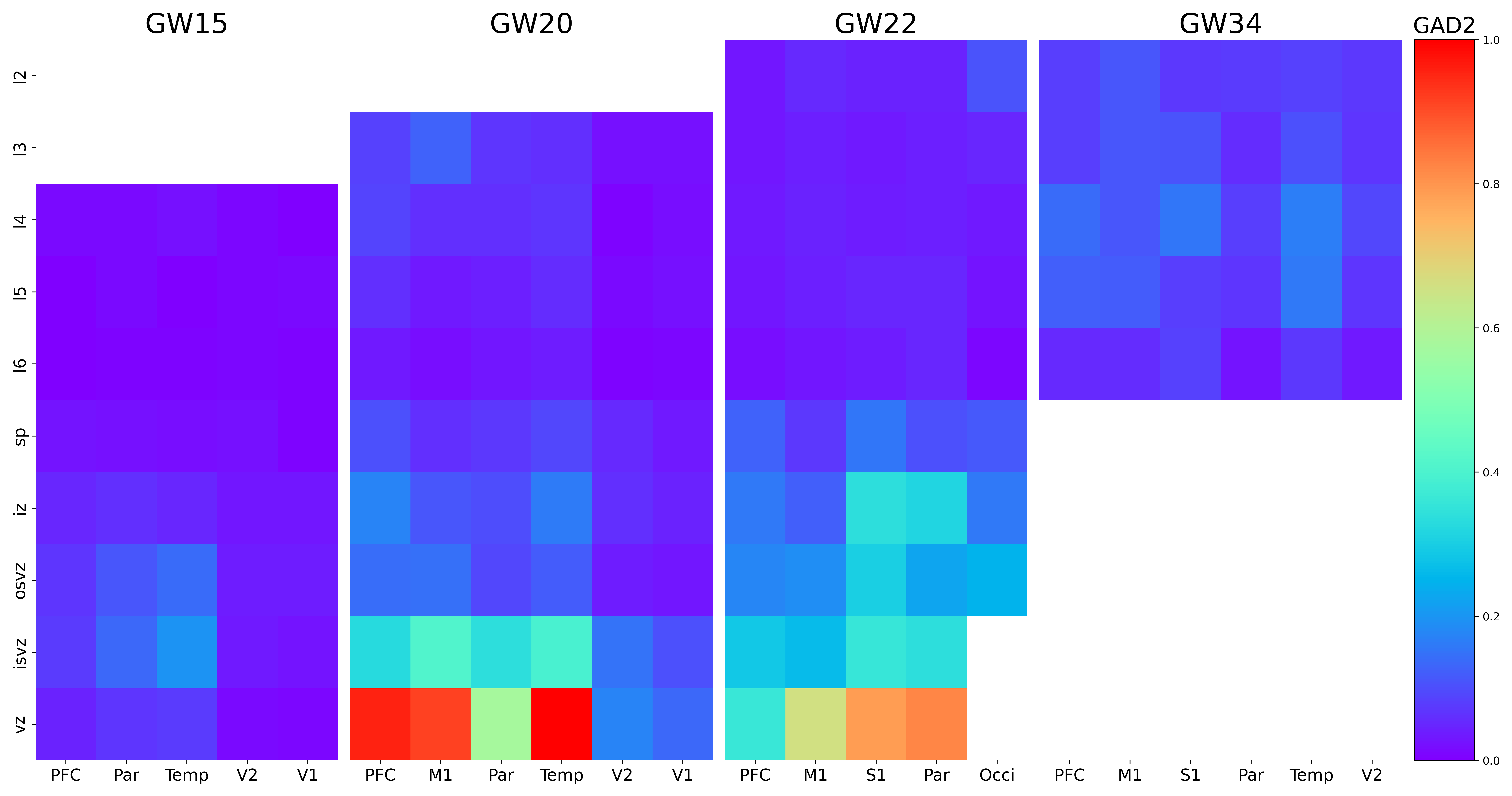

Supplement: Supplementary file 4 — Source Data Fig. 3: Expression pattern heatmap for all 300 genes in the MERFISH. [file 41586_2025_9010_MOESM4_ESM.zip › GAD2.png]

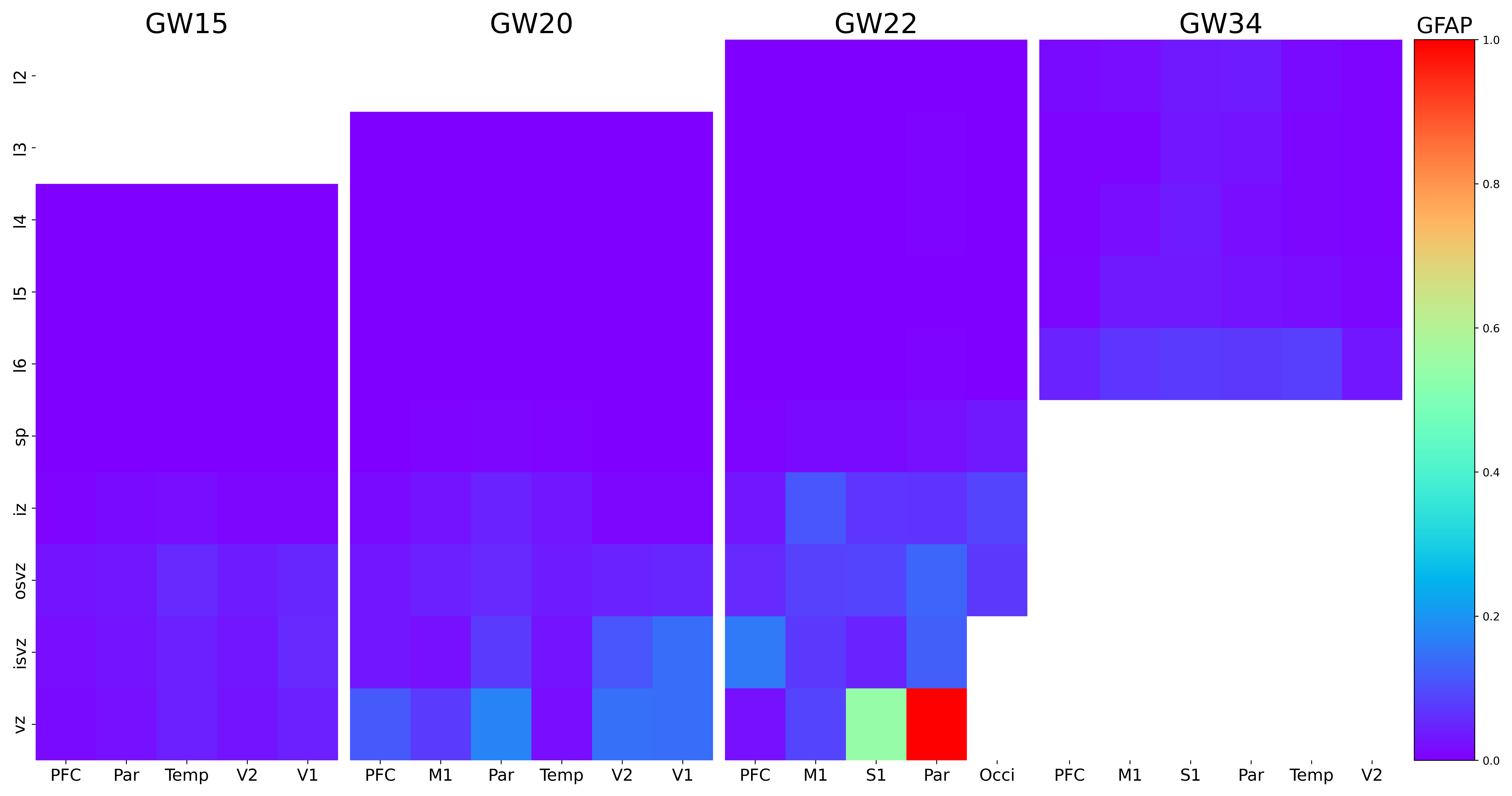

Supplement: Supplementary file 4 — Source Data Fig. 3: Expression pattern heatmap for all 300 genes in the MERFISH. [file 41586_2025_9010_MOESM4_ESM.zip › GFAP.png]

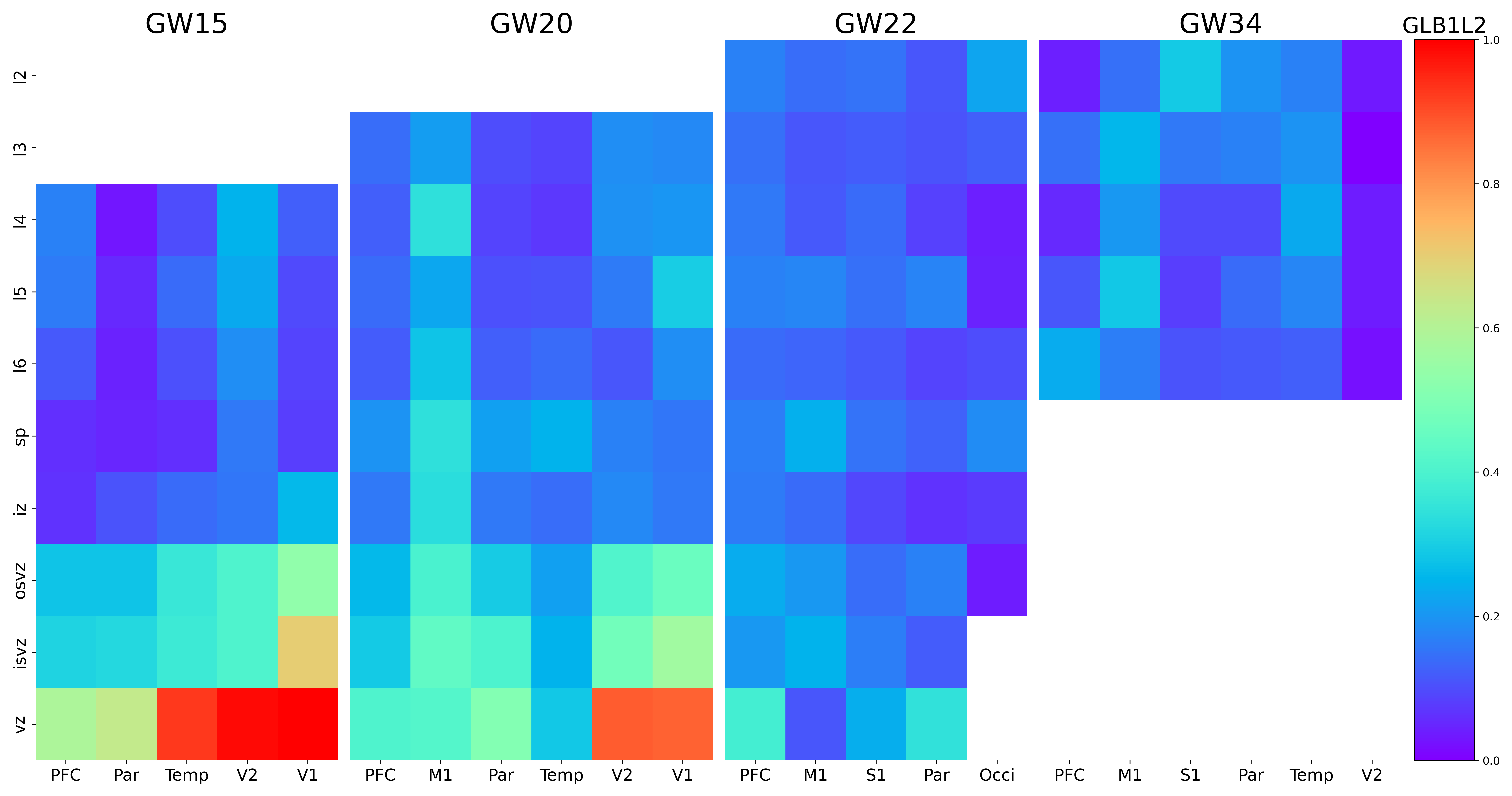

Supplement: Supplementary file 4 — Source Data Fig. 3: Expression pattern heatmap for all 300 genes in the MERFISH. [file 41586_2025_9010_MOESM4_ESM.zip › GLB1L2.png]

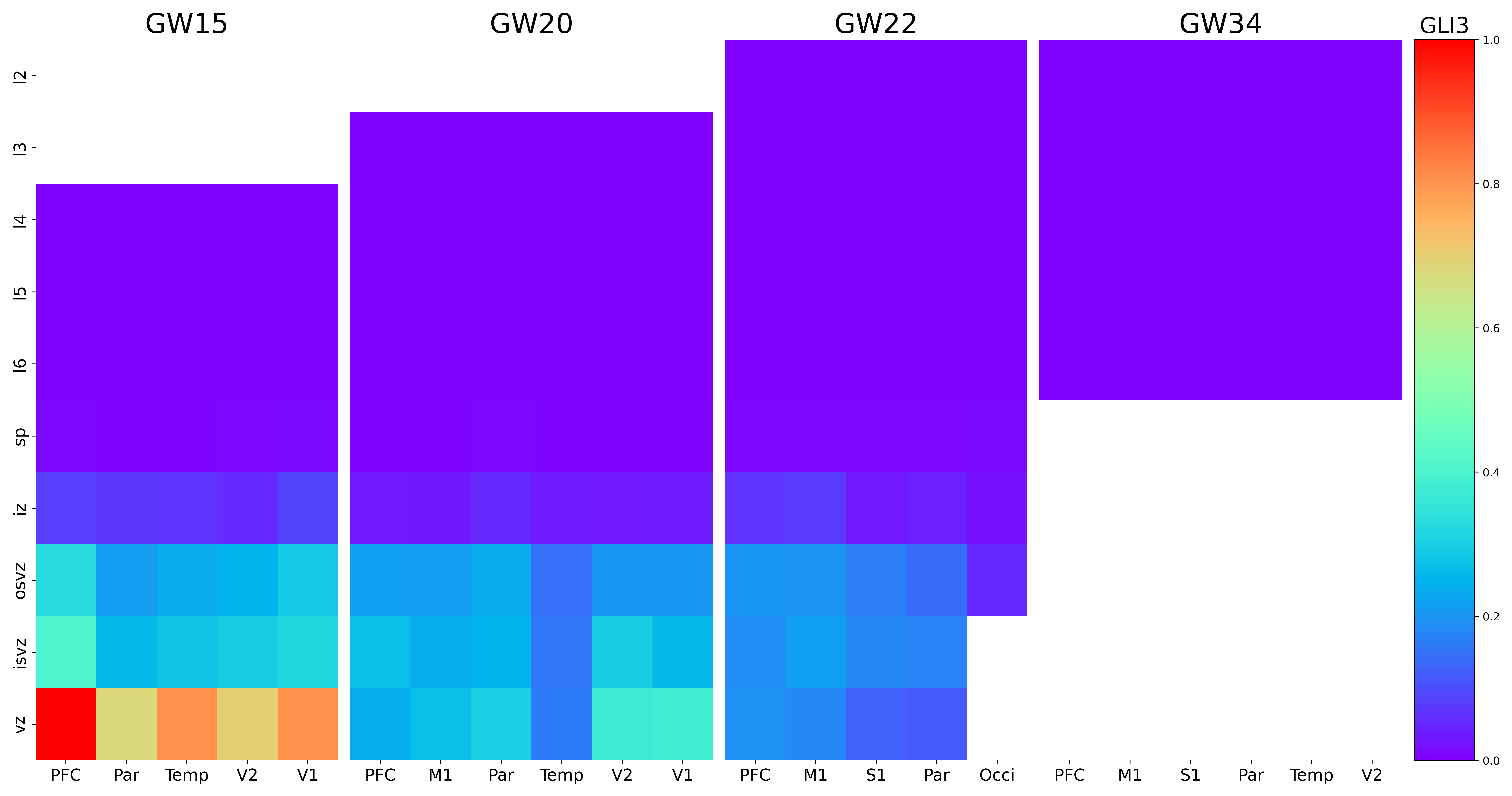

Supplement: Supplementary file 4 — Source Data Fig. 3: Expression pattern heatmap for all 300 genes in the MERFISH. [file 41586_2025_9010_MOESM4_ESM.zip › GLI3.png]

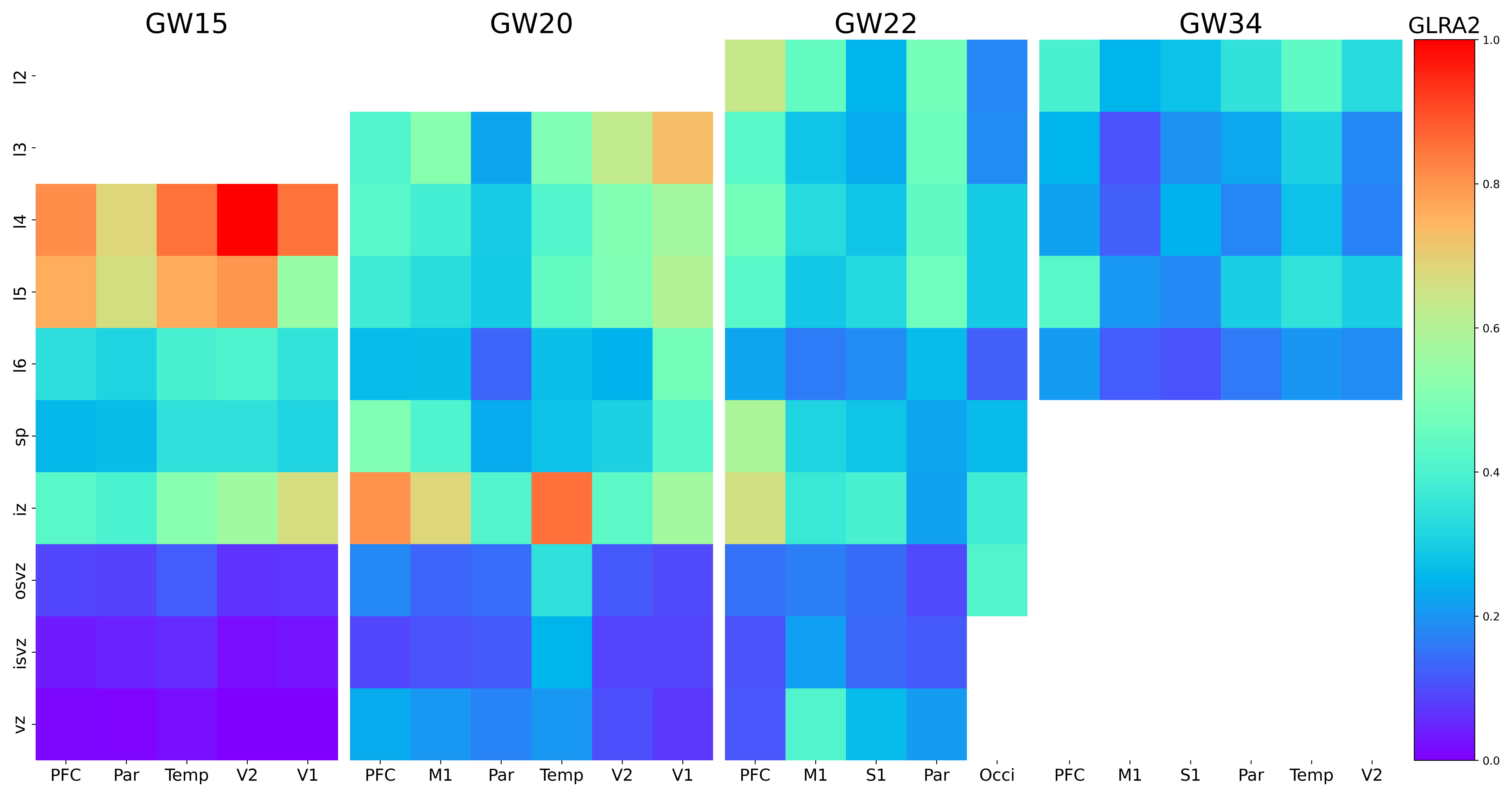

Supplement: Supplementary file 4 — Source Data Fig. 3: Expression pattern heatmap for all 300 genes in the MERFISH. [file 41586_2025_9010_MOESM4_ESM.zip › GLRA2.png]

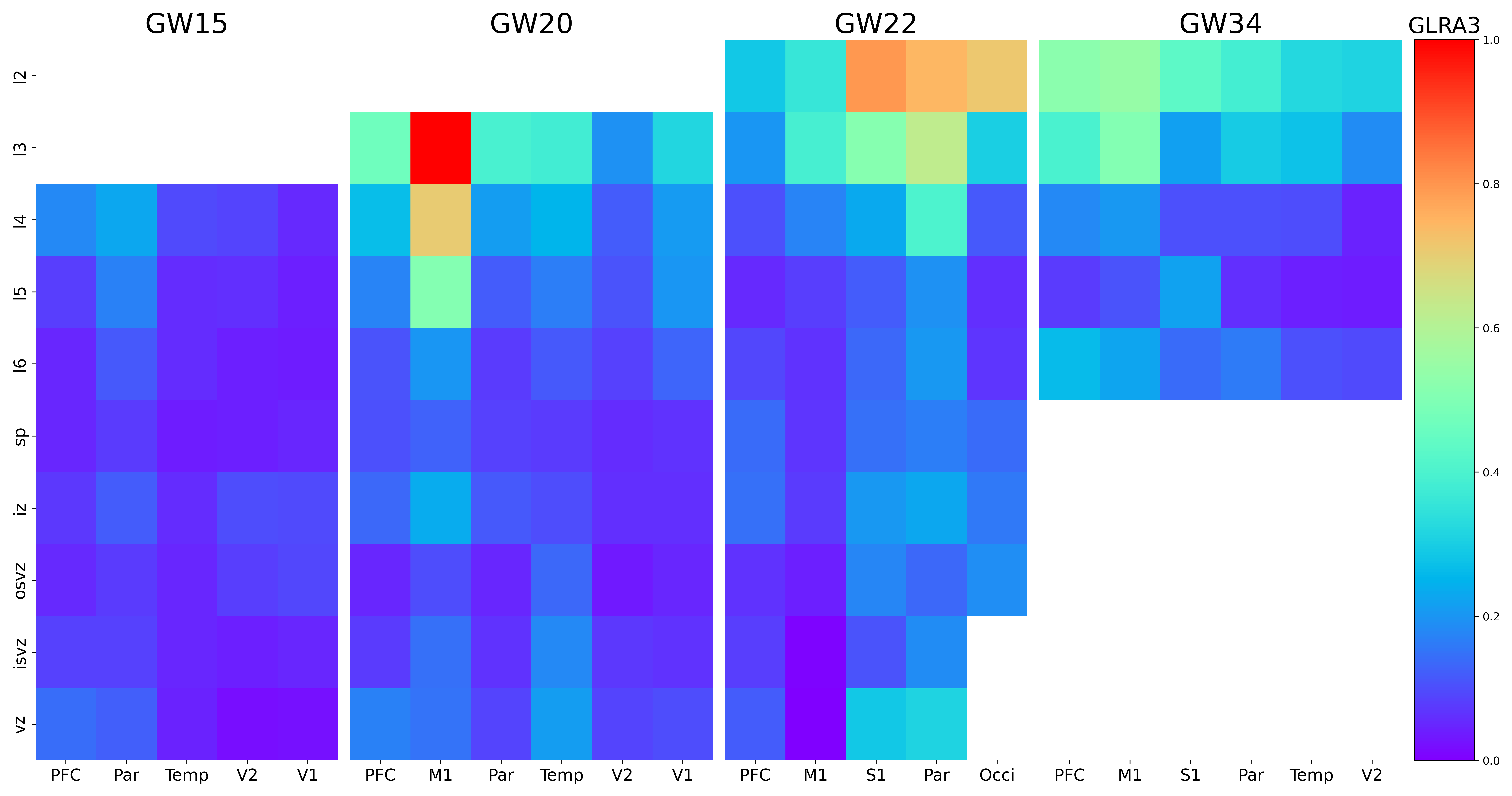

Supplement: Supplementary file 4 — Source Data Fig. 3: Expression pattern heatmap for all 300 genes in the MERFISH. [file 41586_2025_9010_MOESM4_ESM.zip › GLRA3.png]

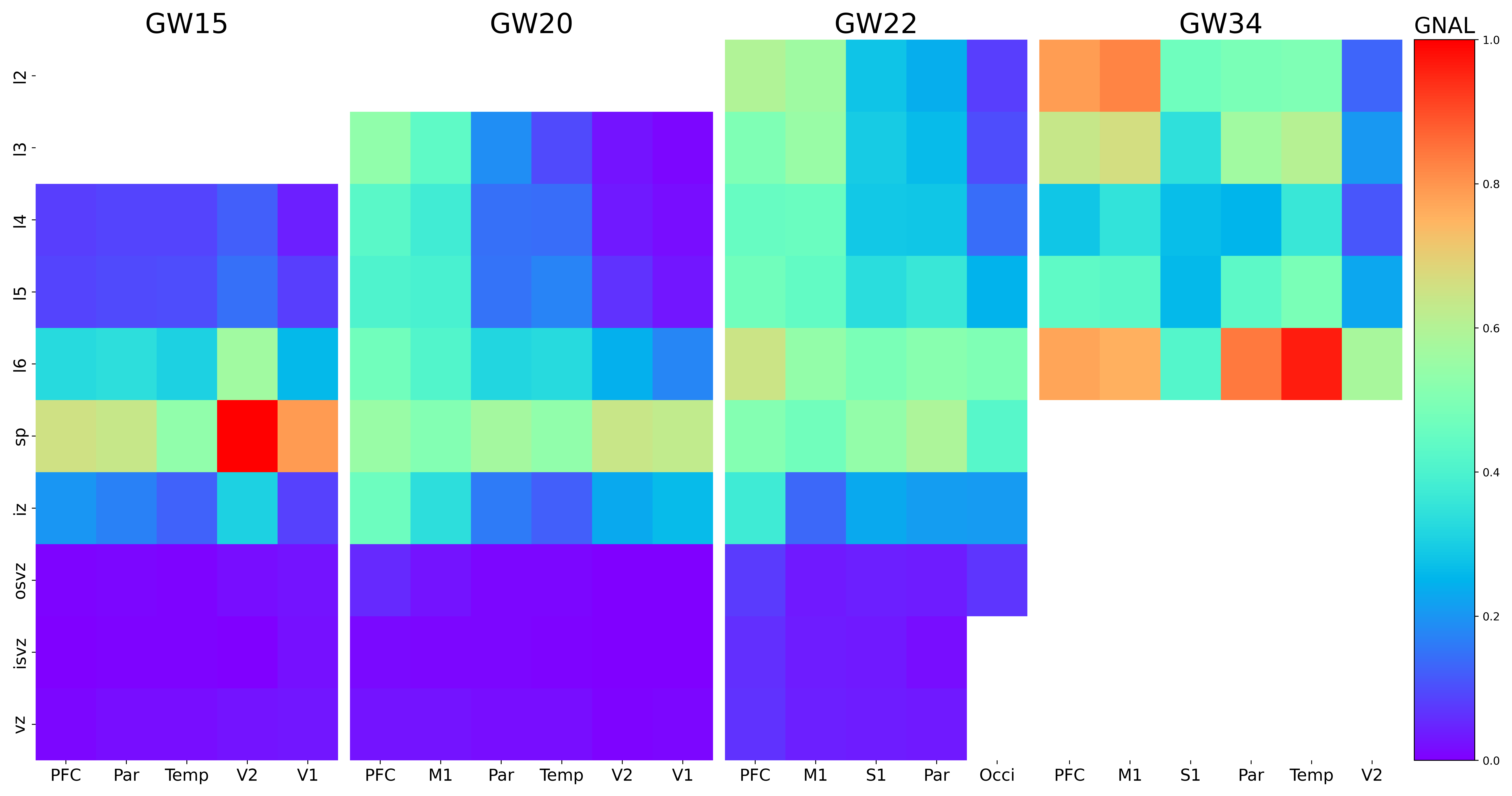

Supplement: Supplementary file 4 — Source Data Fig. 3: Expression pattern heatmap for all 300 genes in the MERFISH. [file 41586_2025_9010_MOESM4_ESM.zip › GNAL.png]

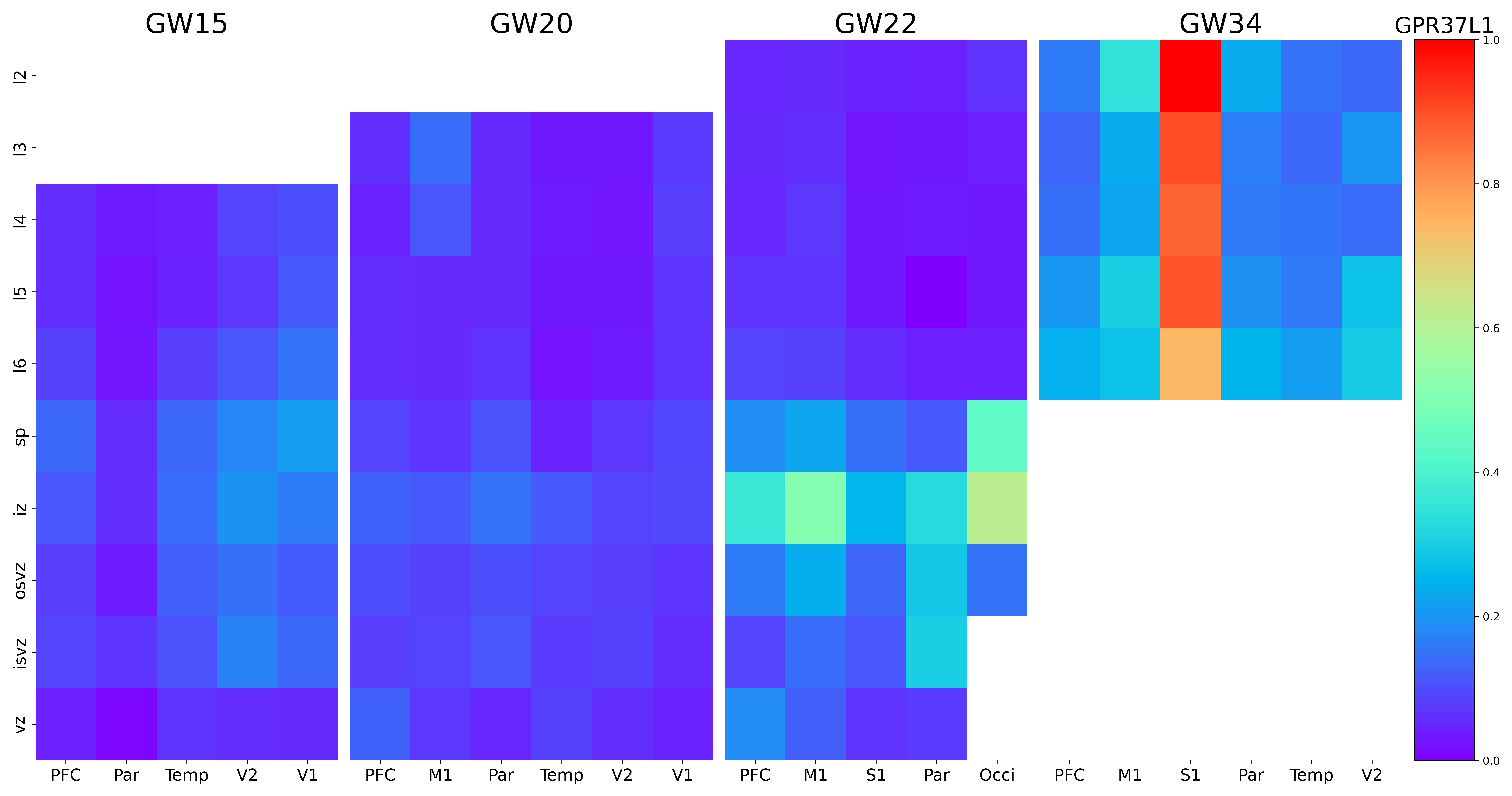

Supplement: Supplementary file 4 — Source Data Fig. 3: Expression pattern heatmap for all 300 genes in the MERFISH. [file 41586_2025_9010_MOESM4_ESM.zip › GPR37L1.png]

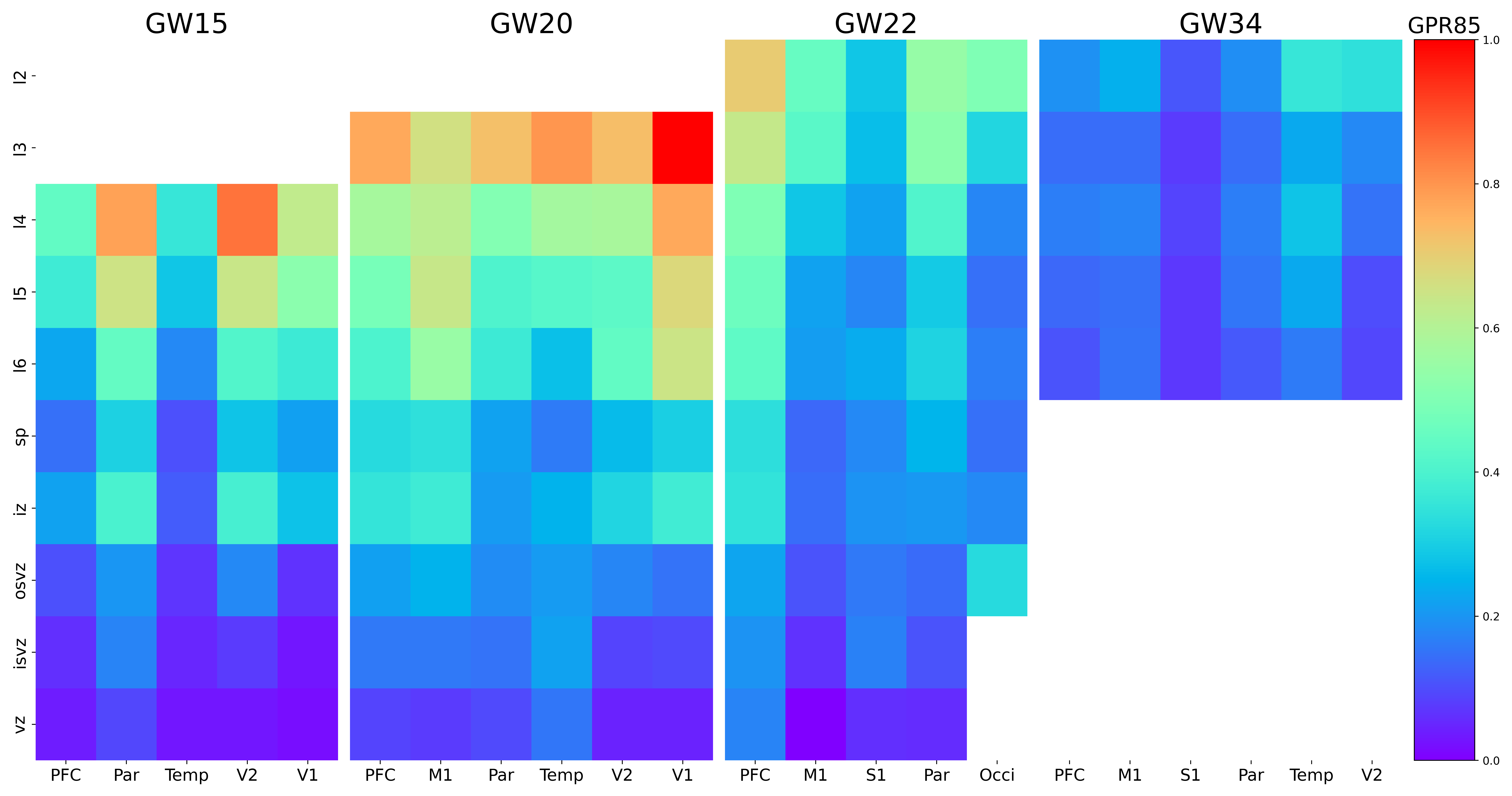

Supplement: Supplementary file 4 — Source Data Fig. 3: Expression pattern heatmap for all 300 genes in the MERFISH. [file 41586_2025_9010_MOESM4_ESM.zip › GPR85.png]

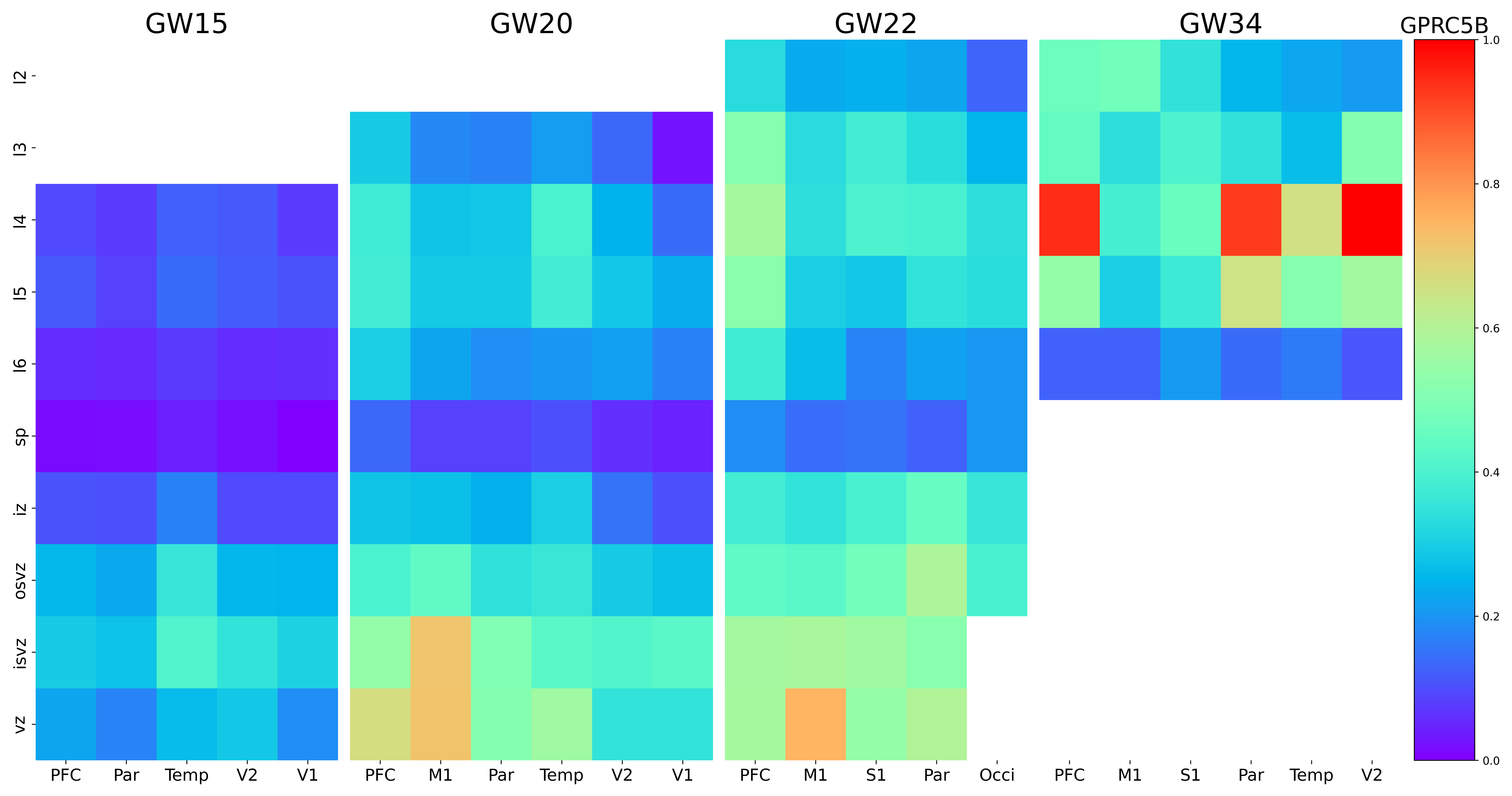

Supplement: Supplementary file 4 — Source Data Fig. 3: Expression pattern heatmap for all 300 genes in the MERFISH. [file 41586_2025_9010_MOESM4_ESM.zip › GPRC5B.png]

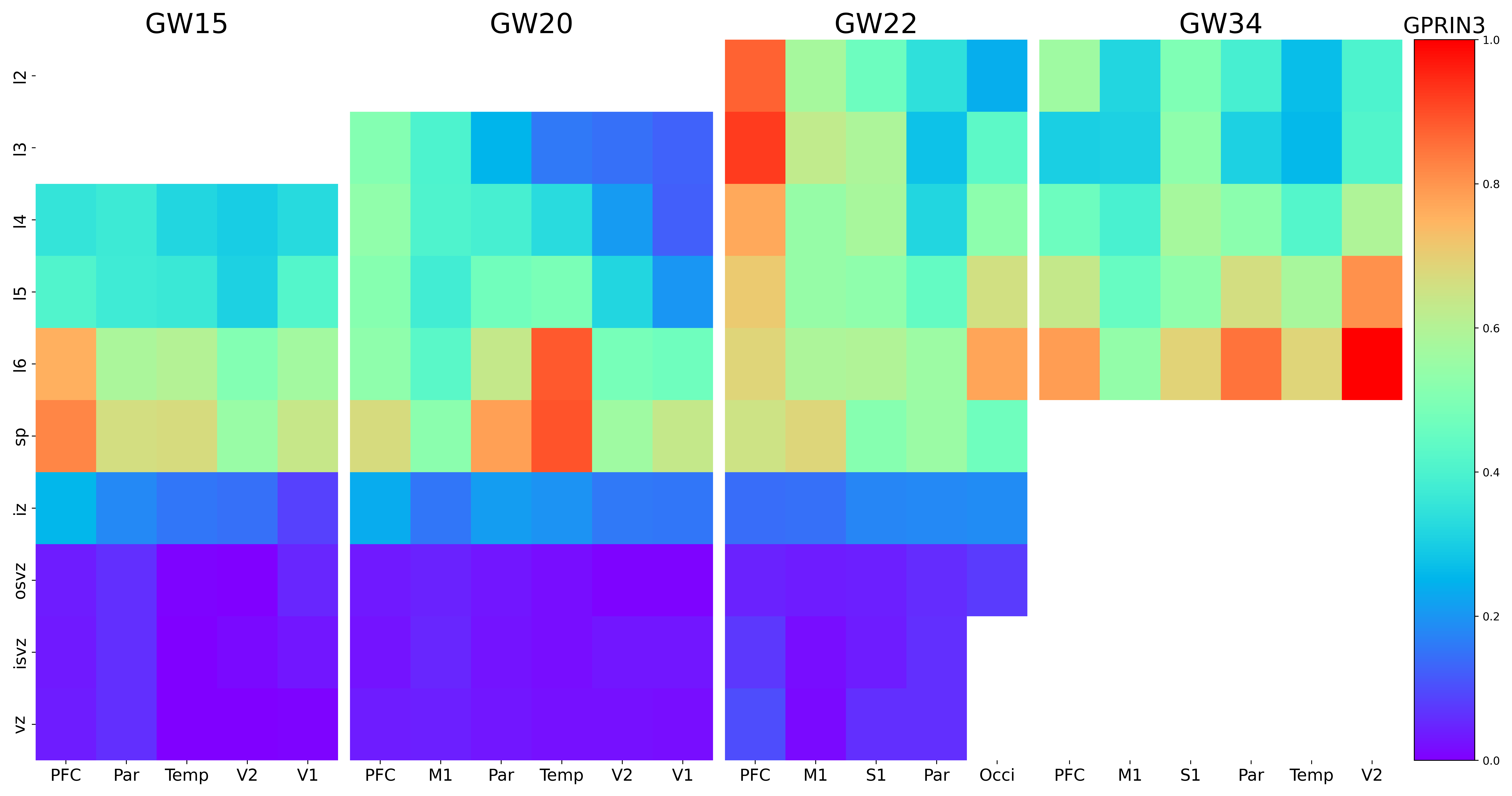

Supplement: Supplementary file 4 — Source Data Fig. 3: Expression pattern heatmap for all 300 genes in the MERFISH. [file 41586_2025_9010_MOESM4_ESM.zip › GPRIN3.png]

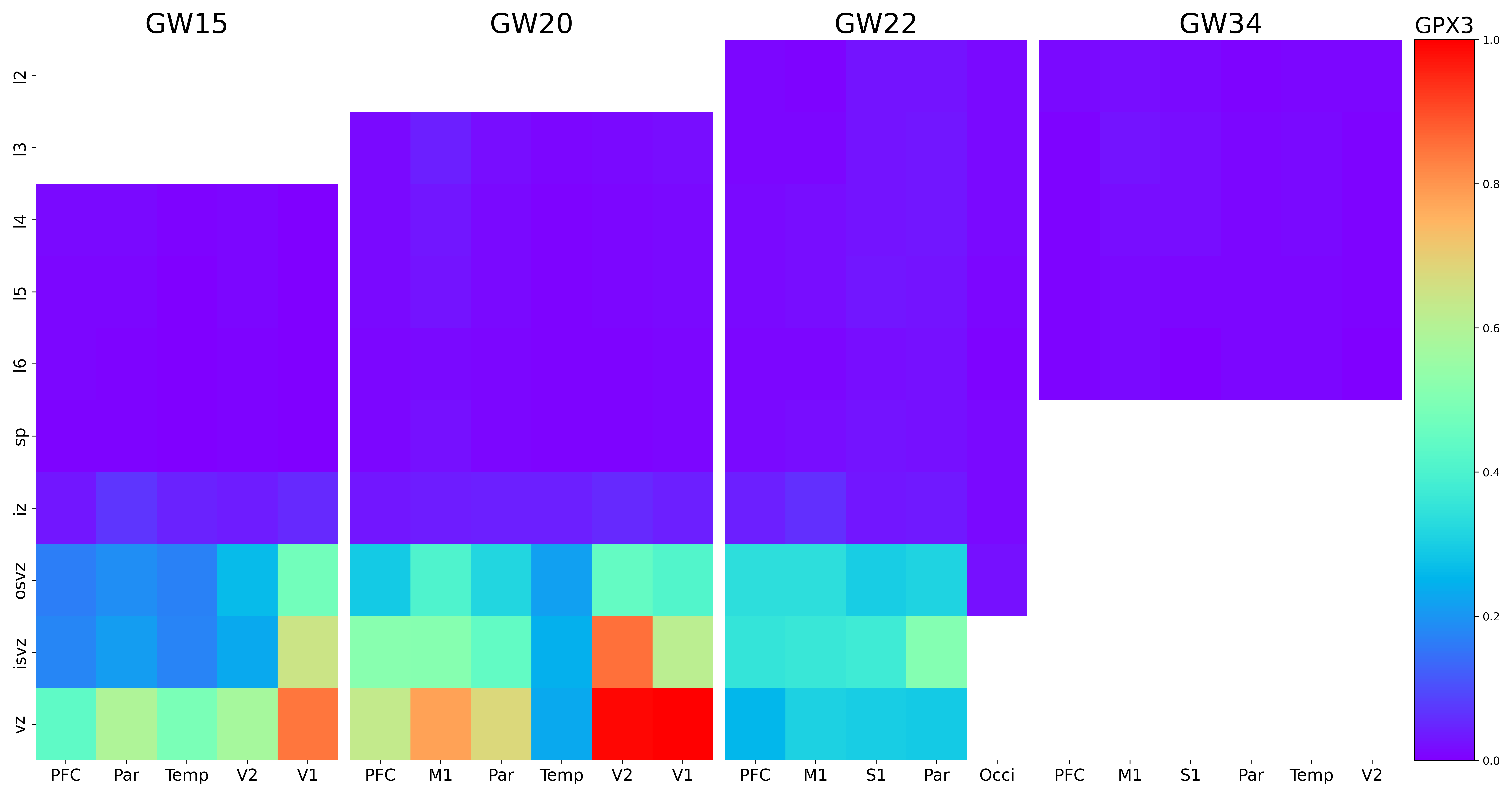

Supplement: Supplementary file 4 — Source Data Fig. 3: Expression pattern heatmap for all 300 genes in the MERFISH. [file 41586_2025_9010_MOESM4_ESM.zip › GPX3.png]

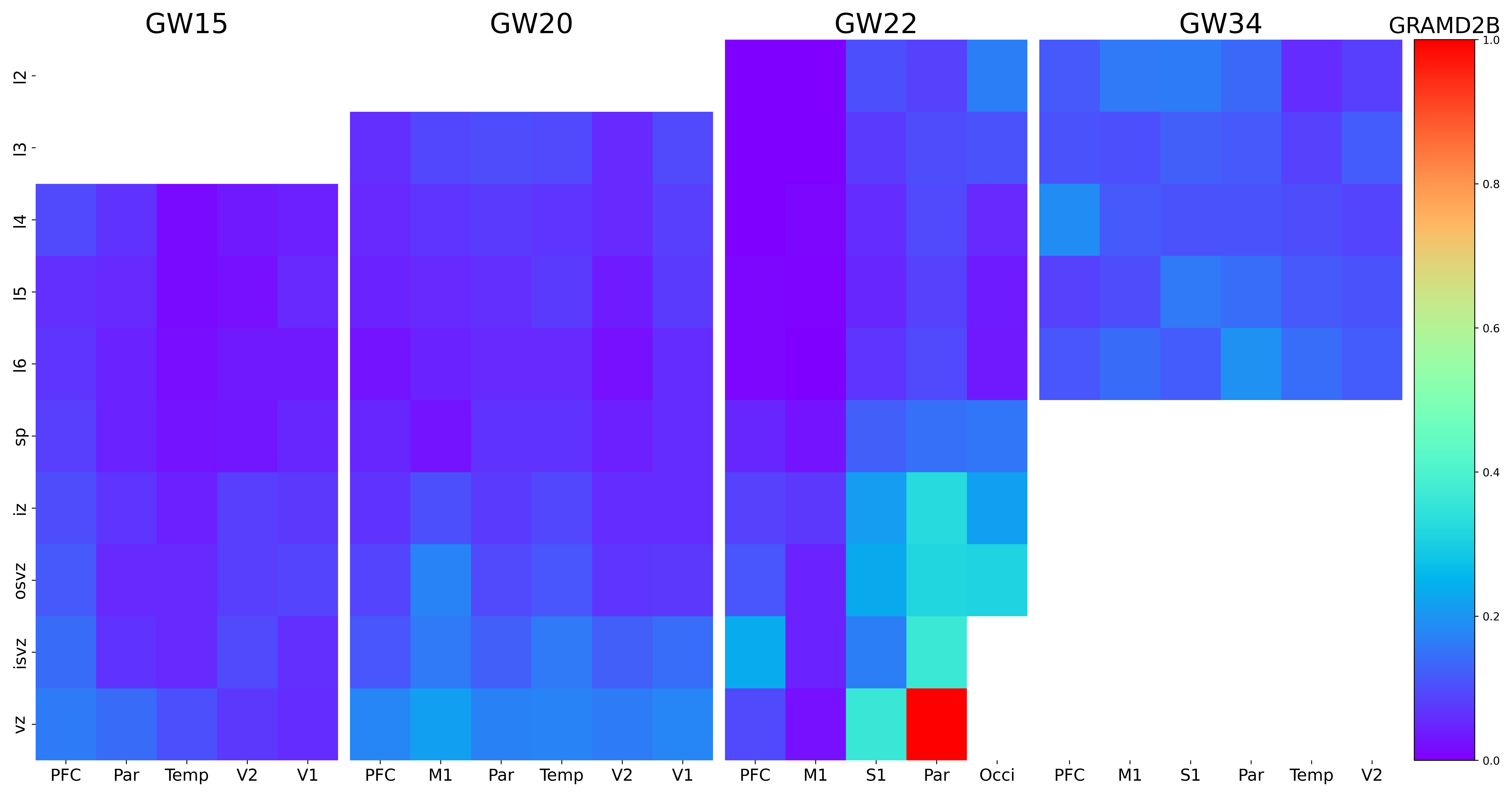

Supplement: Supplementary file 4 — Source Data Fig. 3: Expression pattern heatmap for all 300 genes in the MERFISH. [file 41586_2025_9010_MOESM4_ESM.zip › GRAMD2B.png]

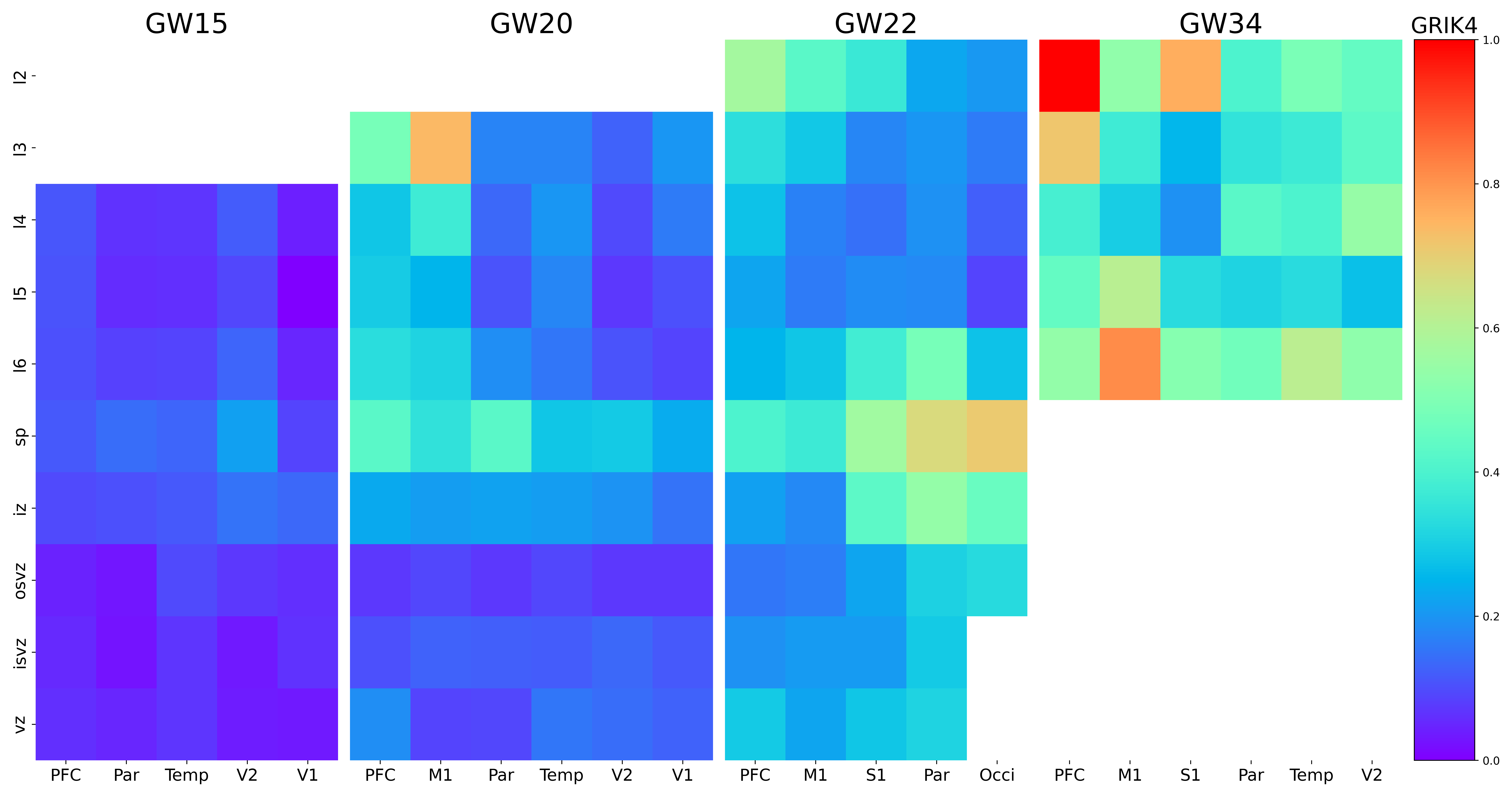

Supplement: Supplementary file 4 — Source Data Fig. 3: Expression pattern heatmap for all 300 genes in the MERFISH. [file 41586_2025_9010_MOESM4_ESM.zip › GRIK4.png]

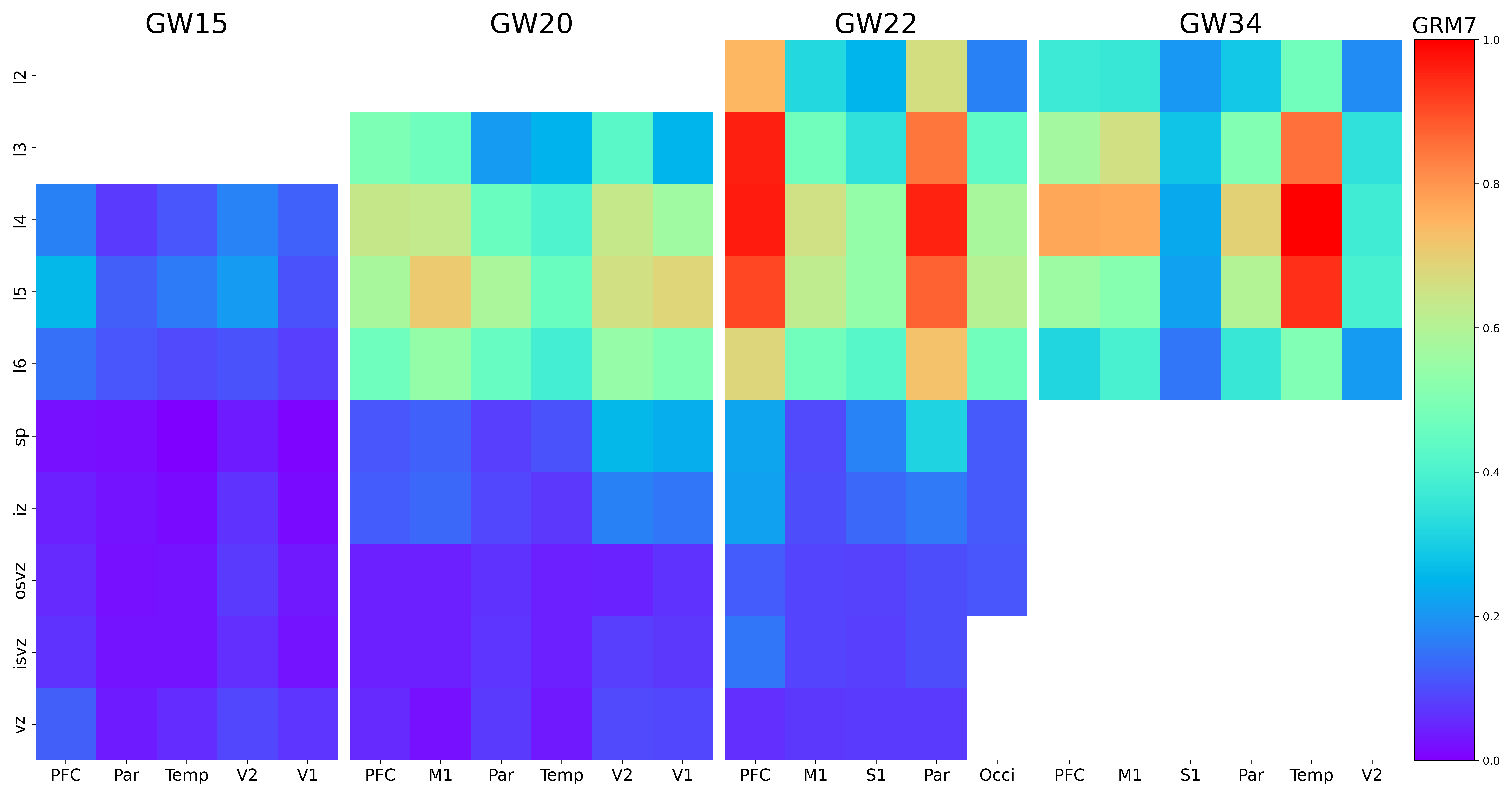

Supplement: Supplementary file 4 — Source Data Fig. 3: Expression pattern heatmap for all 300 genes in the MERFISH. [file 41586_2025_9010_MOESM4_ESM.zip › GRM7.png]

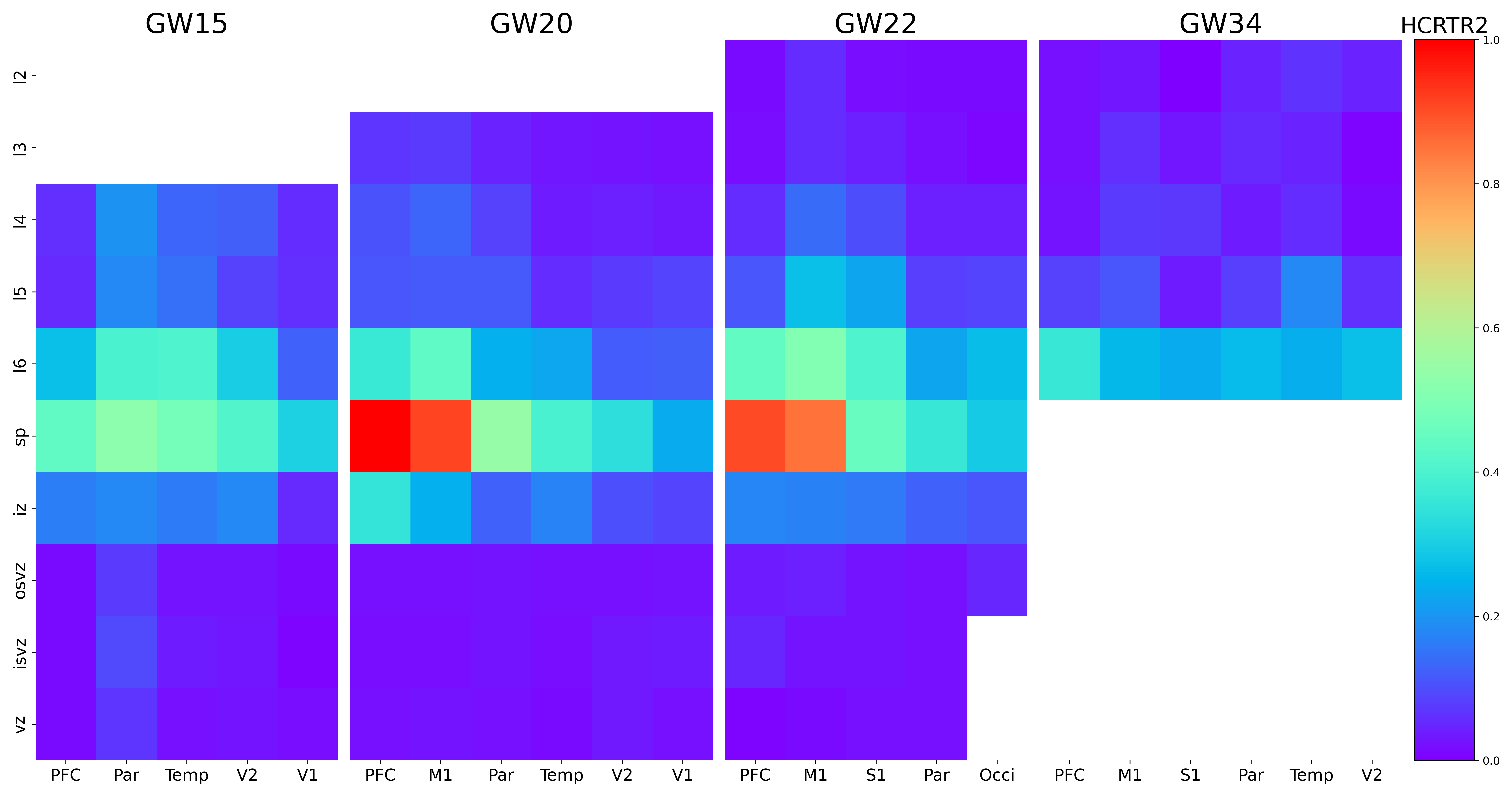

Supplement: Supplementary file 4 — Source Data Fig. 3: Expression pattern heatmap for all 300 genes in the MERFISH. [file 41586_2025_9010_MOESM4_ESM.zip › HCRTR2.png]

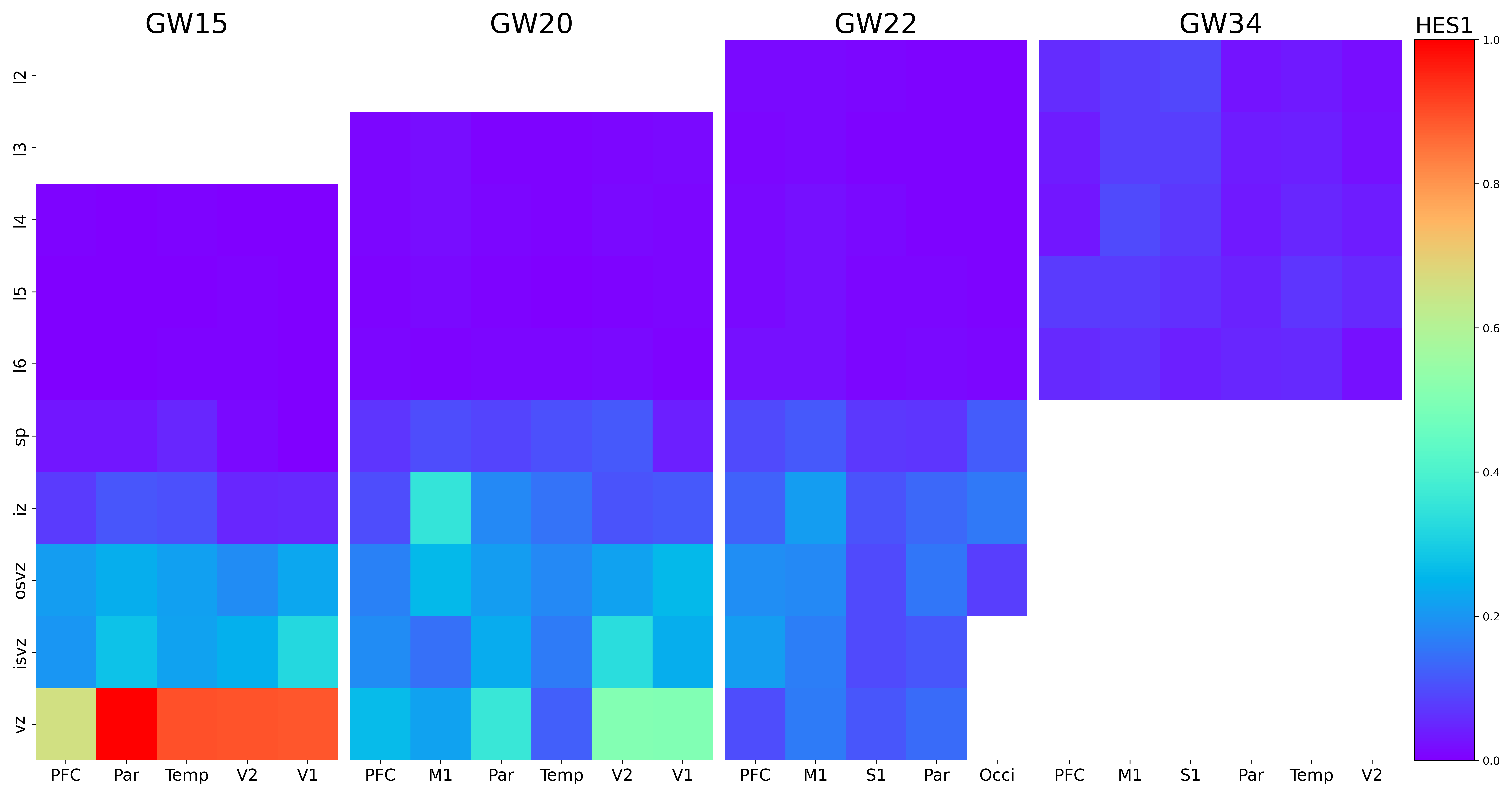

Supplement: Supplementary file 4 — Source Data Fig. 3: Expression pattern heatmap for all 300 genes in the MERFISH. [file 41586_2025_9010_MOESM4_ESM.zip › HES1.png]

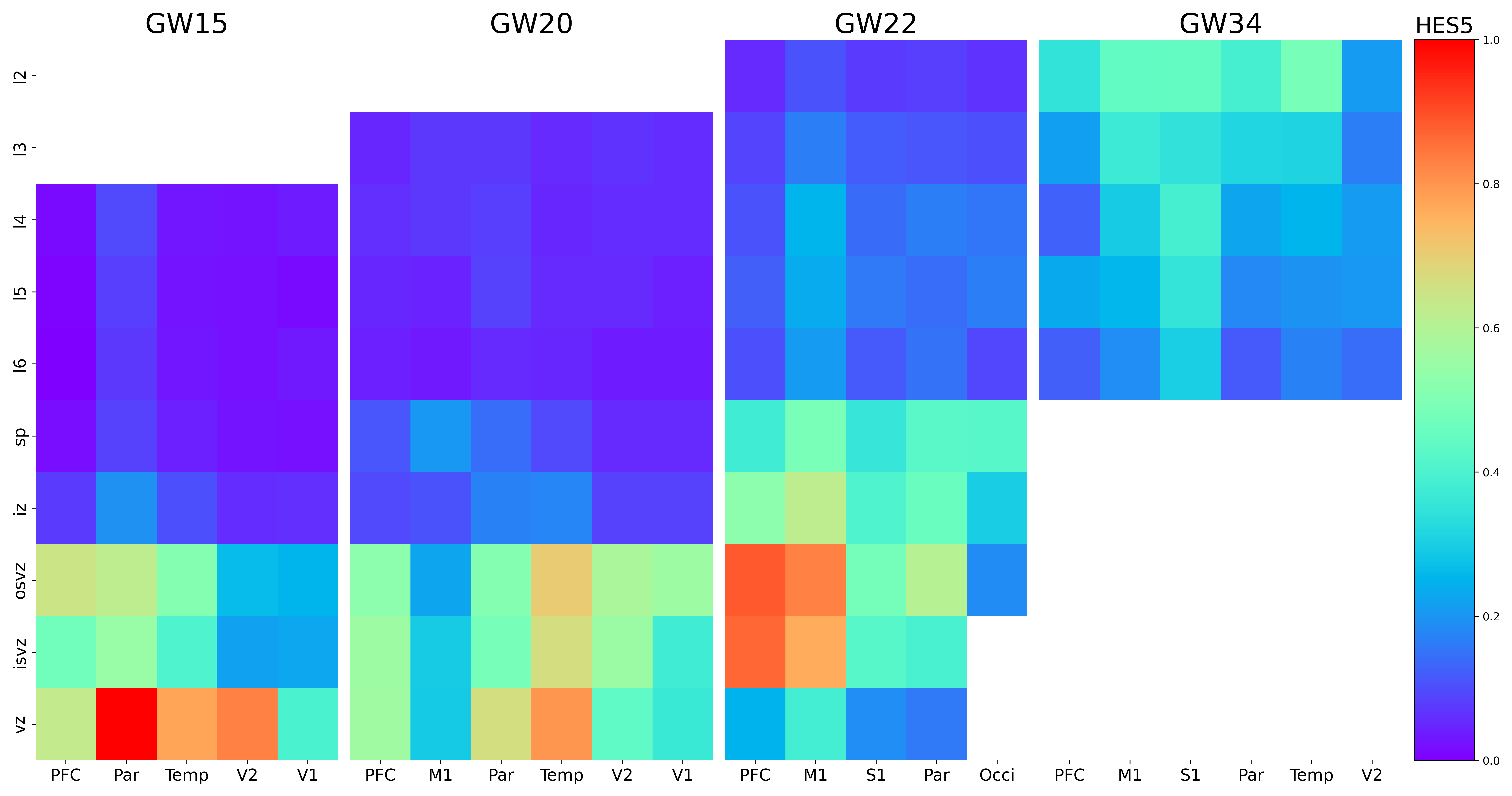

Supplement: Supplementary file 4 — Source Data Fig. 3: Expression pattern heatmap for all 300 genes in the MERFISH. [file 41586_2025_9010_MOESM4_ESM.zip › HES5.png]

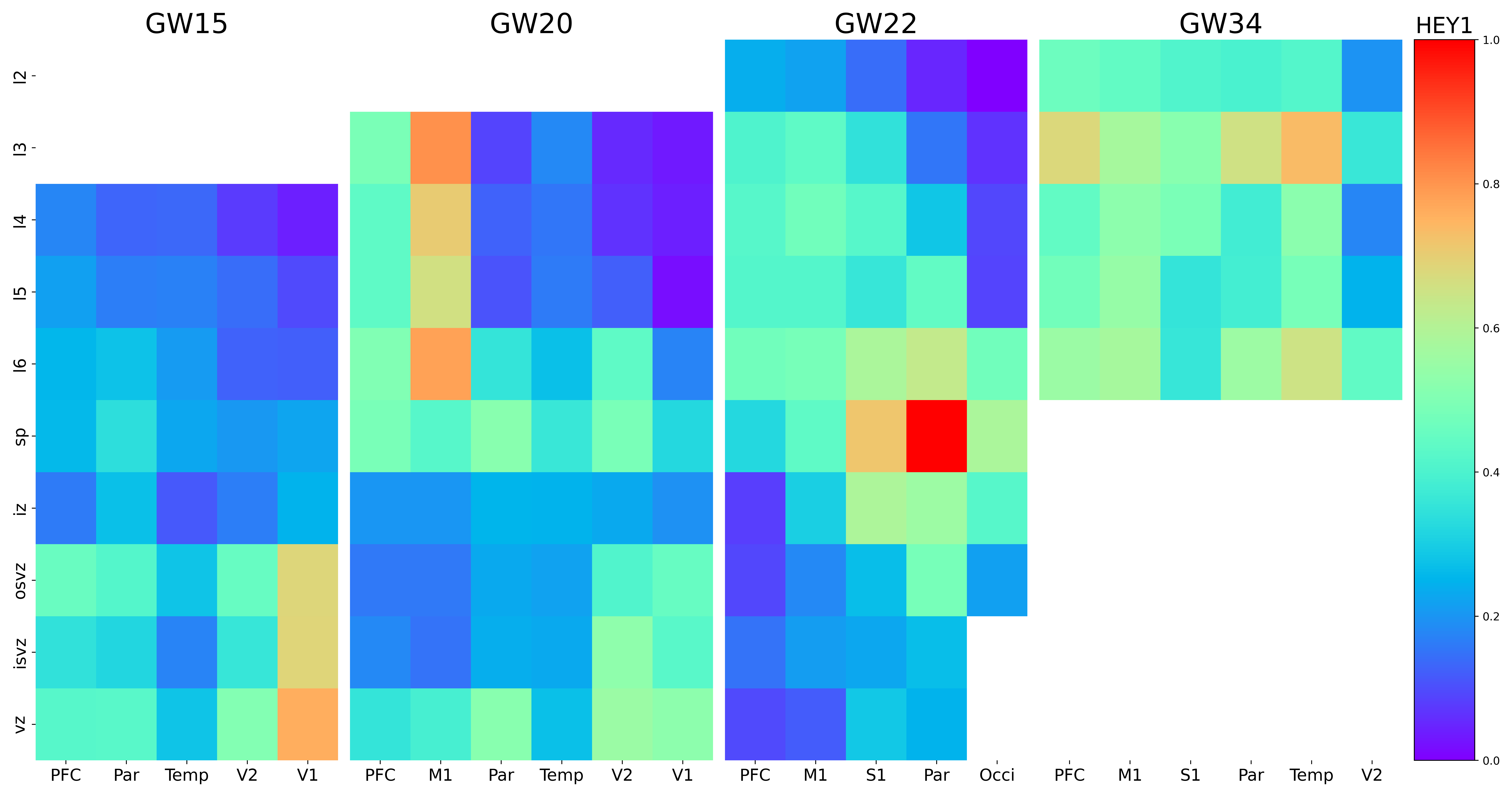

Supplement: Supplementary file 4 — Source Data Fig. 3: Expression pattern heatmap for all 300 genes in the MERFISH. [file 41586_2025_9010_MOESM4_ESM.zip › HEY1.png]

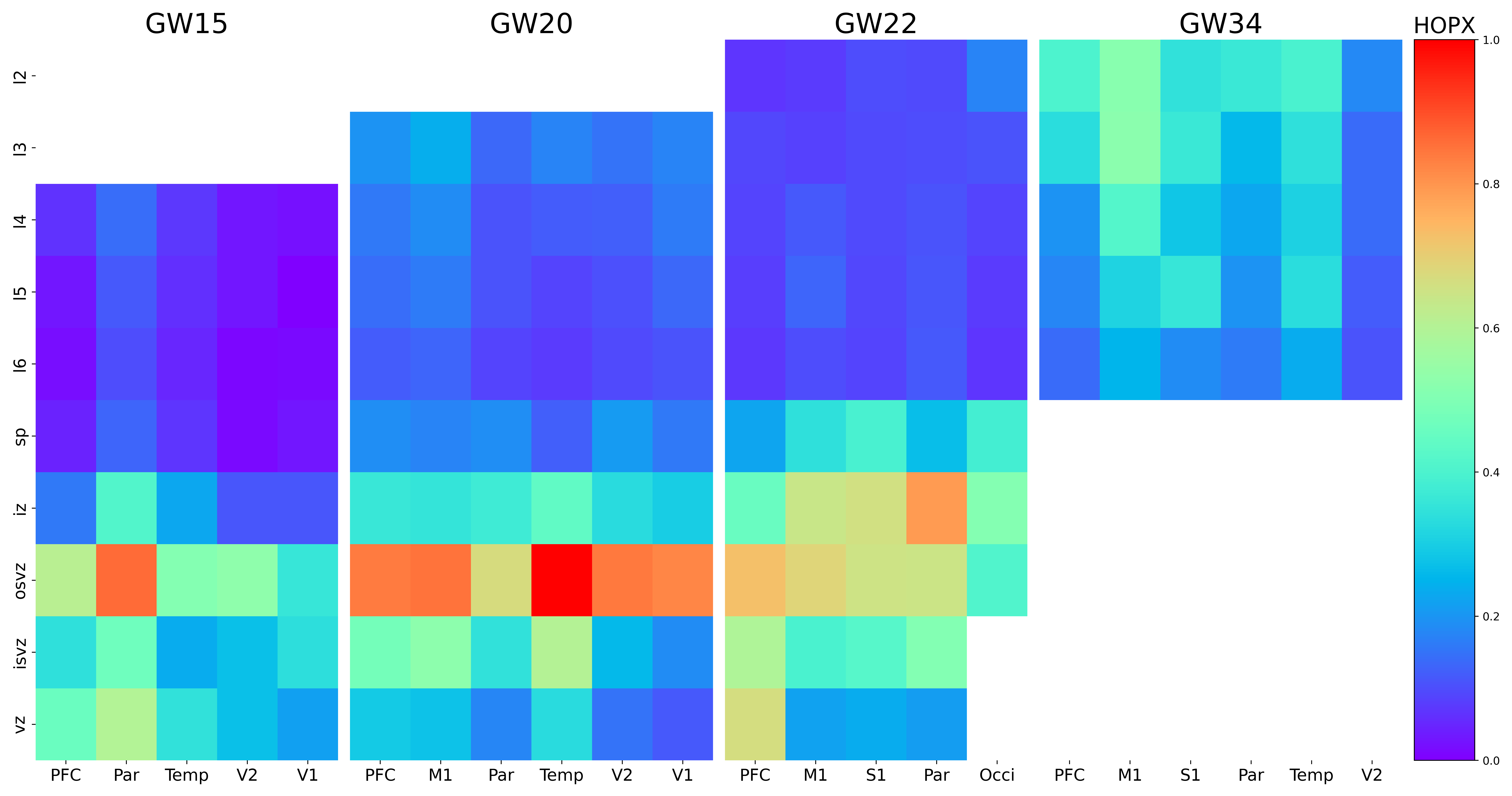

Supplement: Supplementary file 4 — Source Data Fig. 3: Expression pattern heatmap for all 300 genes in the MERFISH. [file 41586_2025_9010_MOESM4_ESM.zip › HOPX.png]

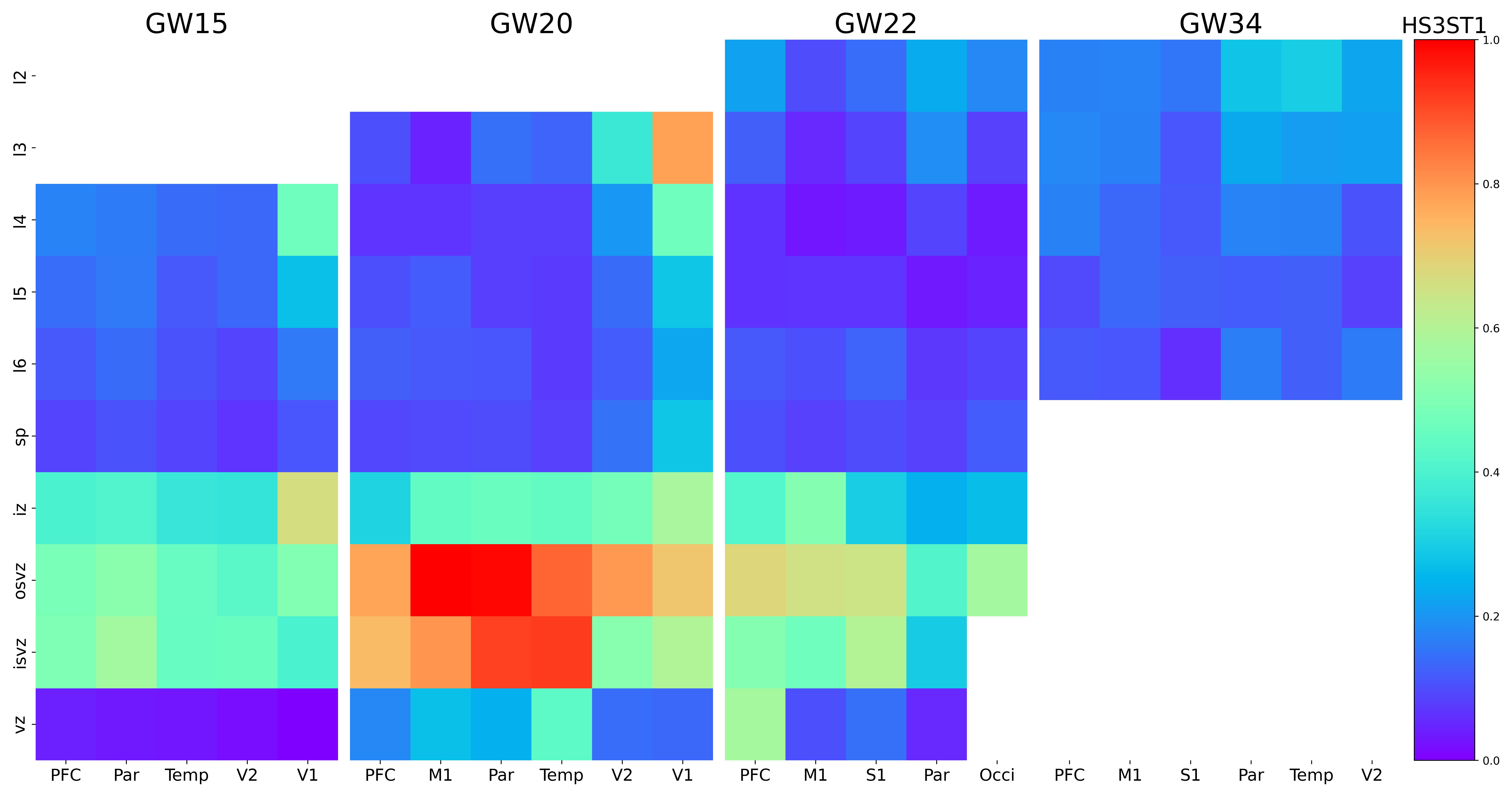

Supplement: Supplementary file 4 — Source Data Fig. 3: Expression pattern heatmap for all 300 genes in the MERFISH. [file 41586_2025_9010_MOESM4_ESM.zip › HS3ST1.png]

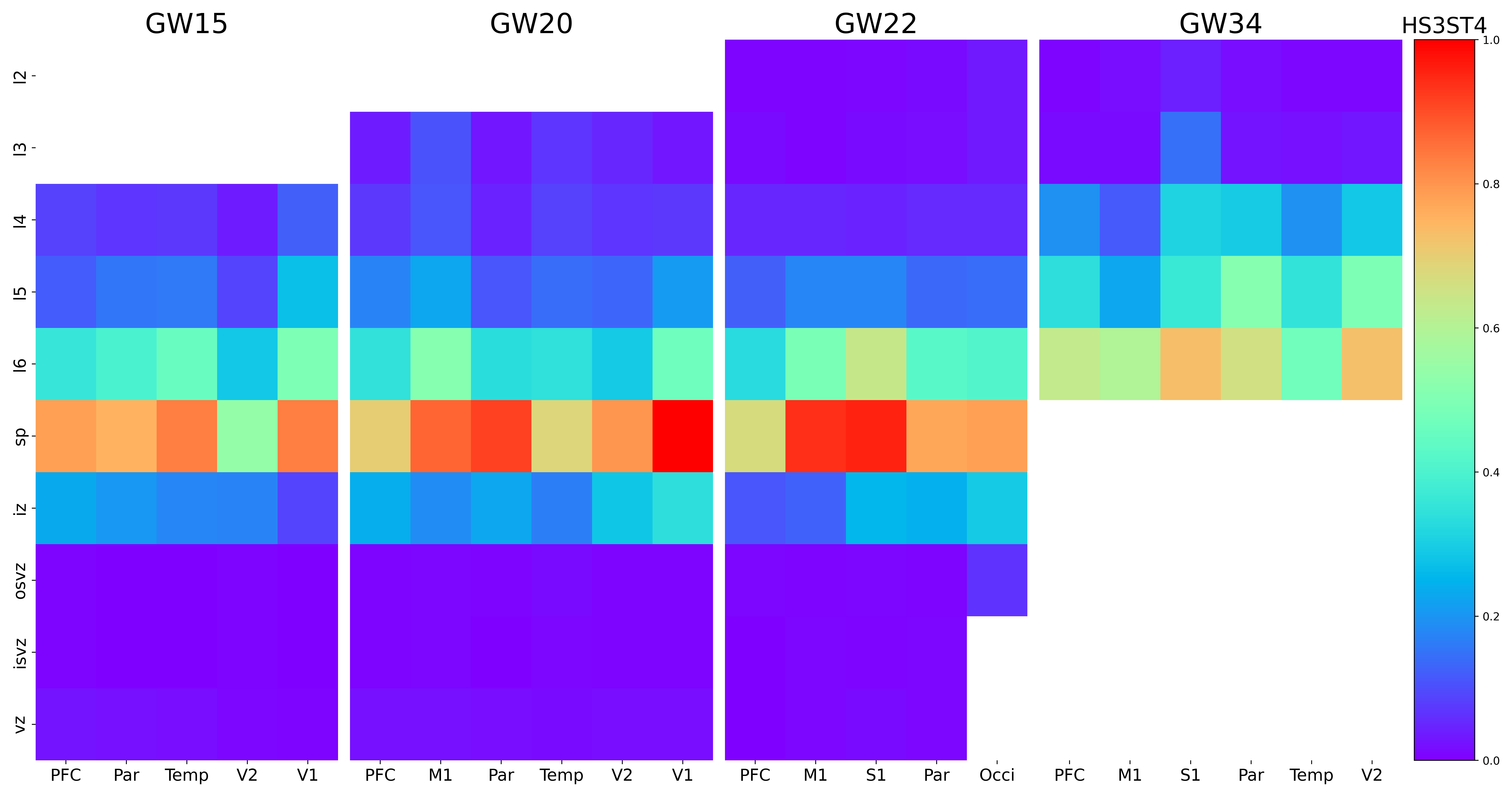

Supplement: Supplementary file 4 — Source Data Fig. 3: Expression pattern heatmap for all 300 genes in the MERFISH. [file 41586_2025_9010_MOESM4_ESM.zip › HS3ST4.png]

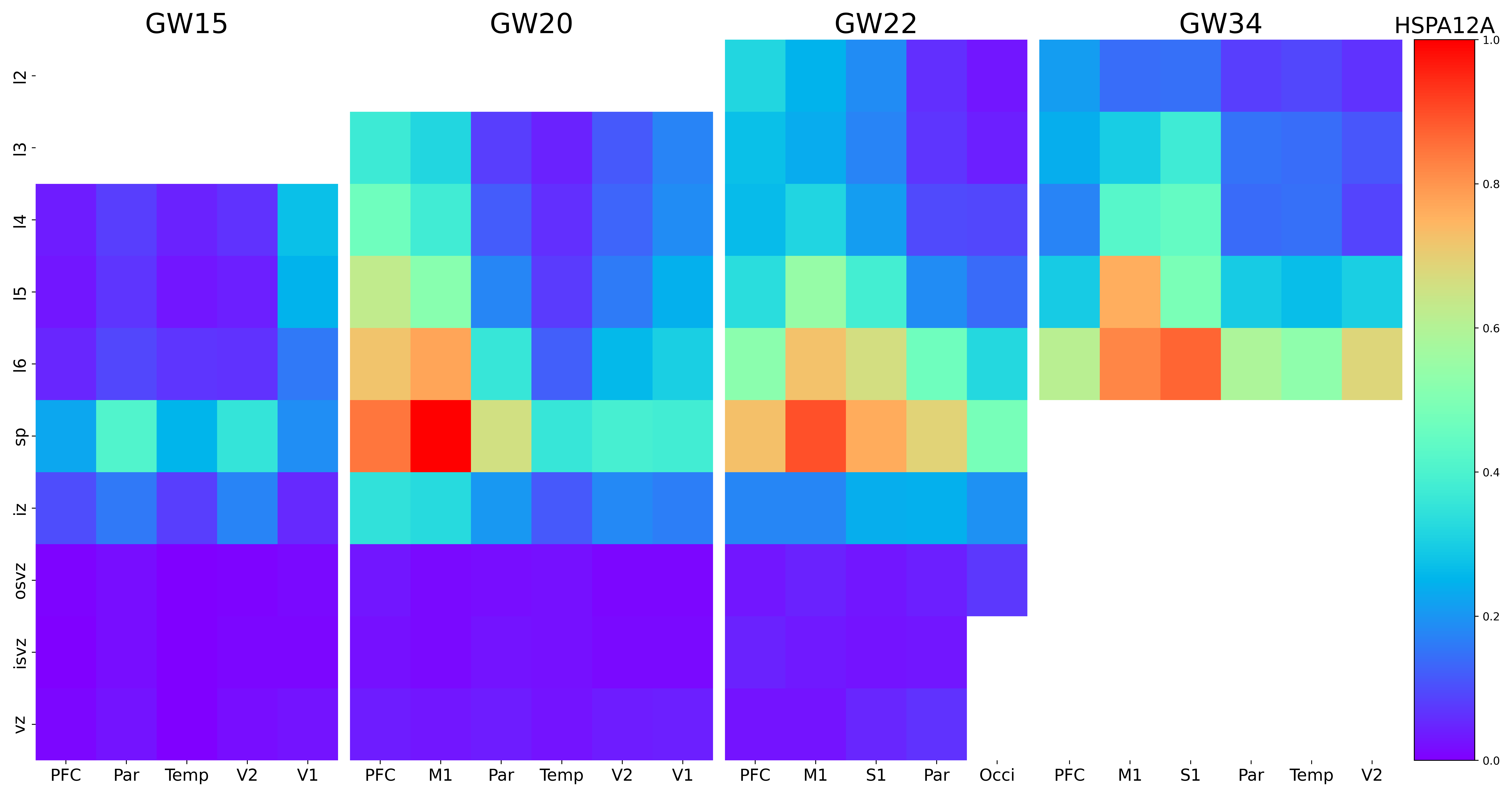

Supplement: Supplementary file 4 — Source Data Fig. 3: Expression pattern heatmap for all 300 genes in the MERFISH. [file 41586_2025_9010_MOESM4_ESM.zip › HSPA12A.png]

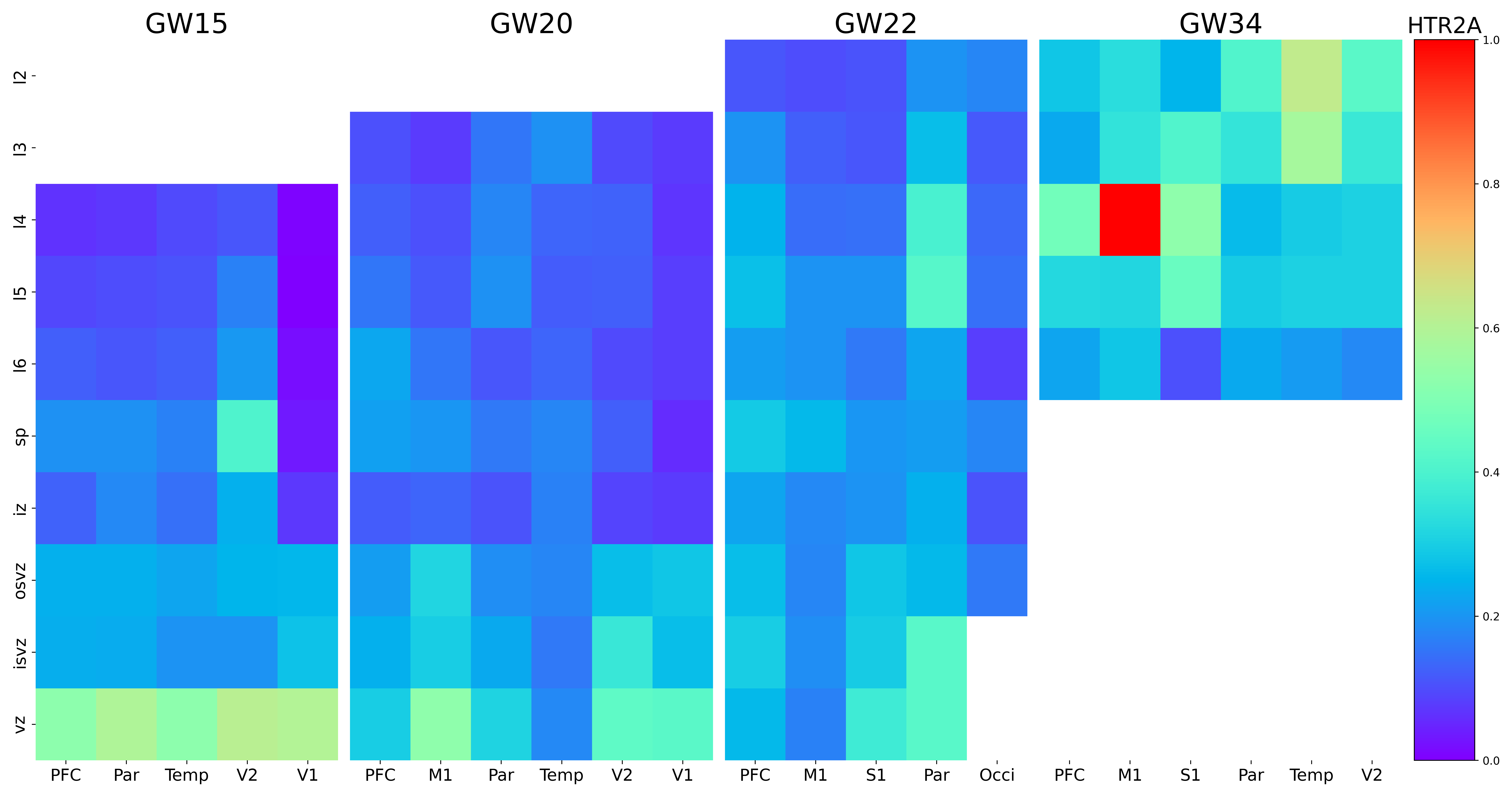

Supplement: Supplementary file 4 — Source Data Fig. 3: Expression pattern heatmap for all 300 genes in the MERFISH. [file 41586_2025_9010_MOESM4_ESM.zip › HTR2A.png]

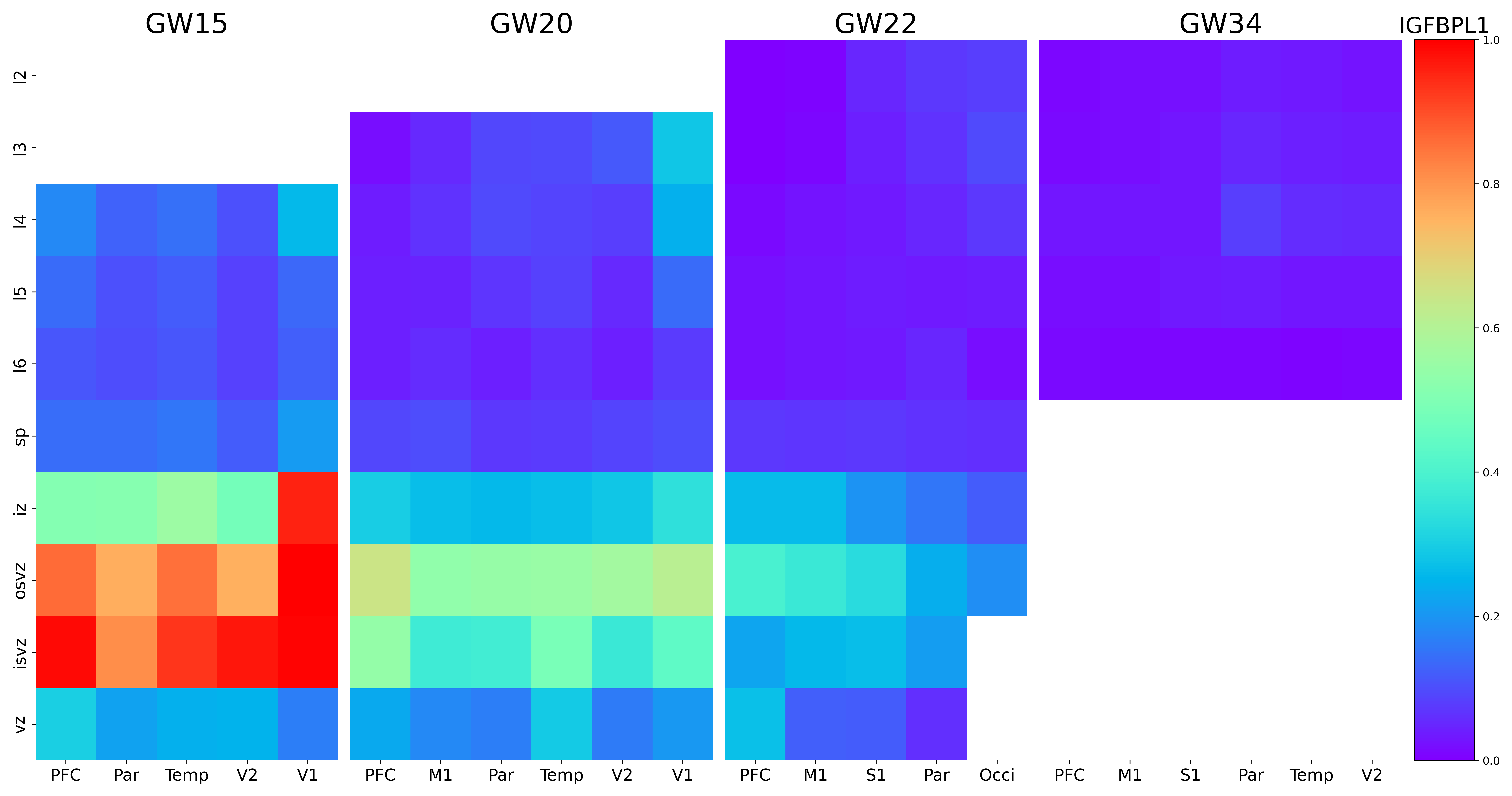

Supplement: Supplementary file 4 — Source Data Fig. 3: Expression pattern heatmap for all 300 genes in the MERFISH. [file 41586_2025_9010_MOESM4_ESM.zip › IGFBPL1.png]

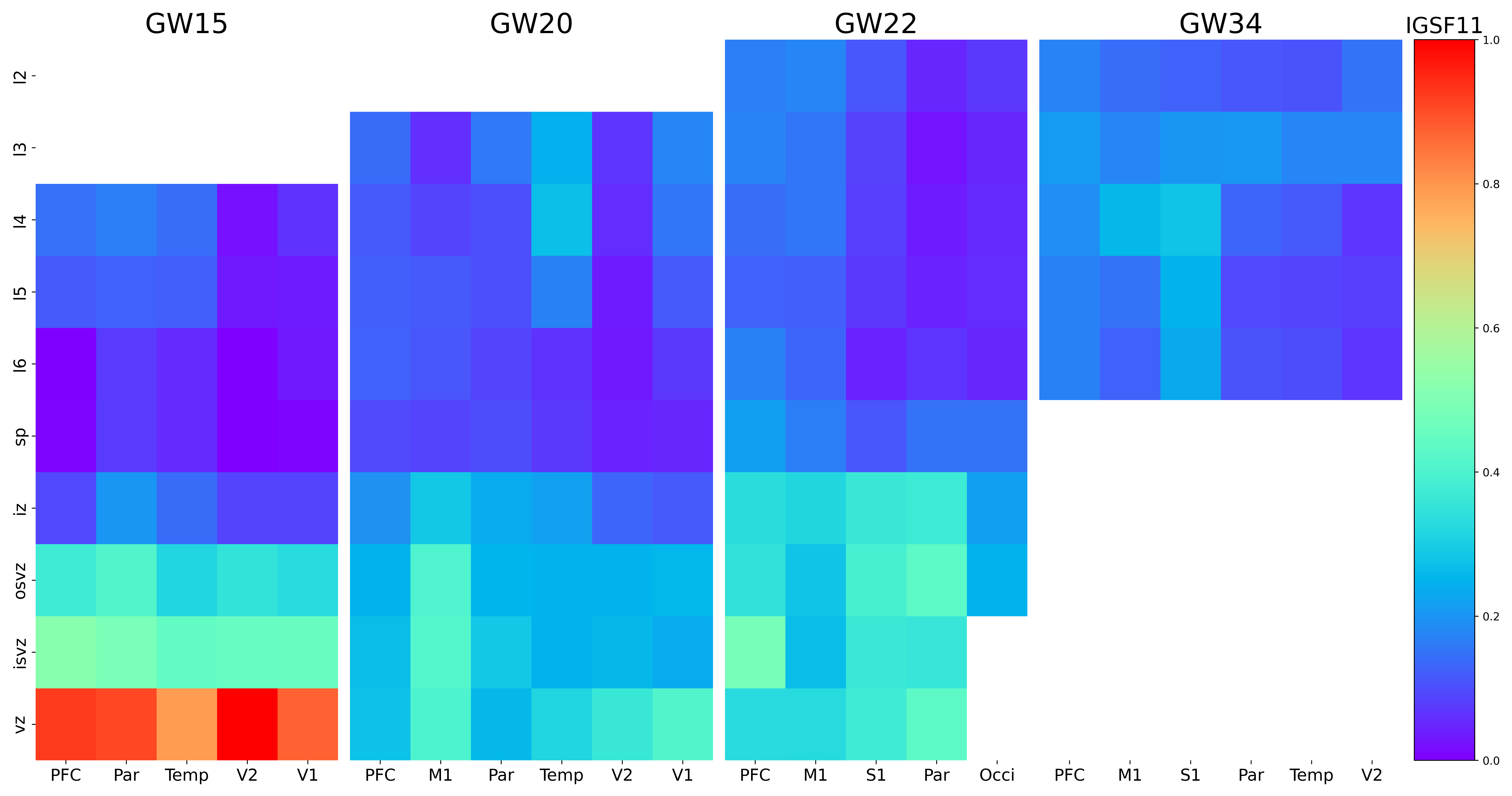

Supplement: Supplementary file 4 — Source Data Fig. 3: Expression pattern heatmap for all 300 genes in the MERFISH. [file 41586_2025_9010_MOESM4_ESM.zip › IGSF11.png]

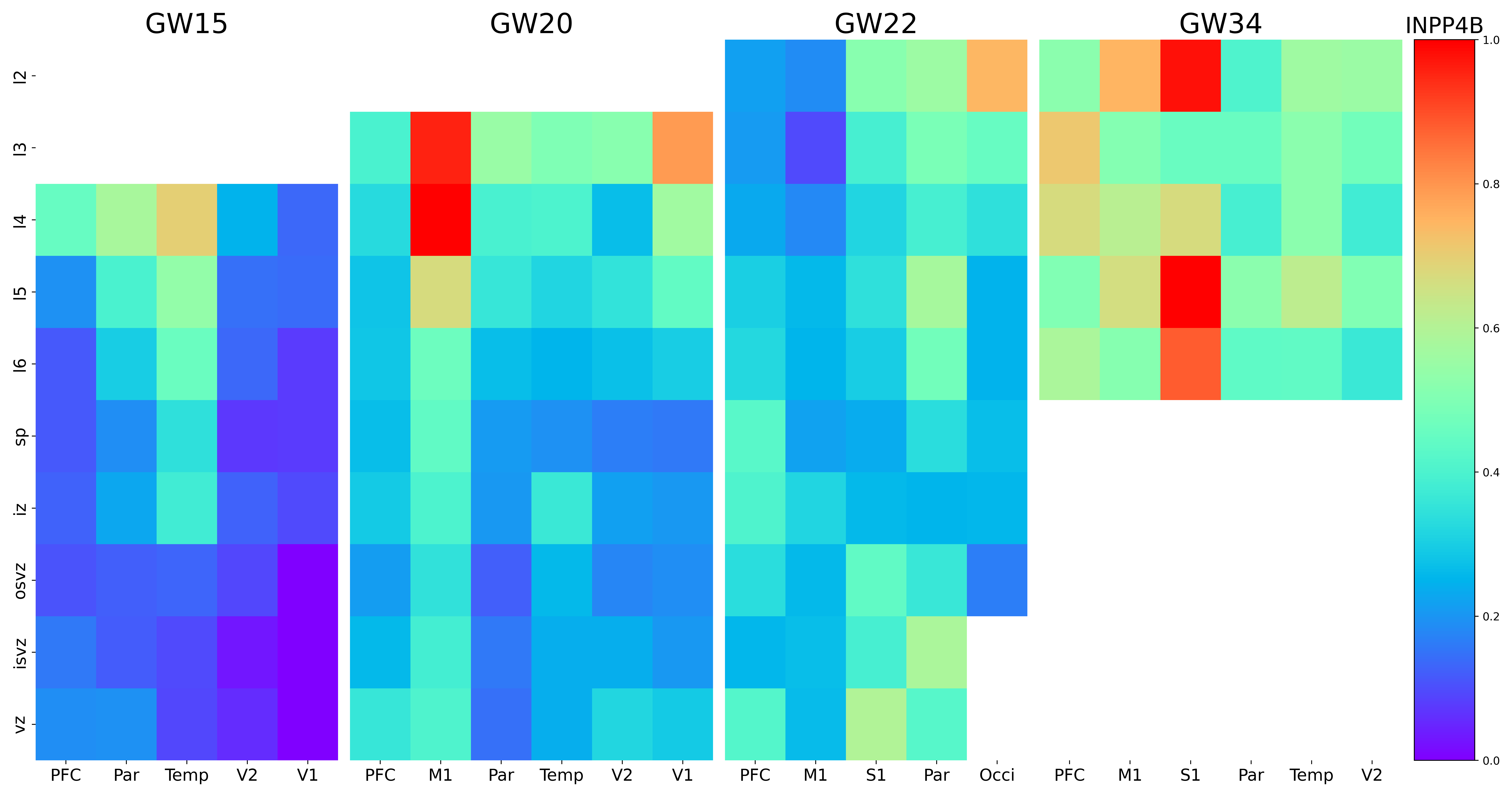

Supplement: Supplementary file 4 — Source Data Fig. 3: Expression pattern heatmap for all 300 genes in the MERFISH. [file 41586_2025_9010_MOESM4_ESM.zip › INPP4B.png]

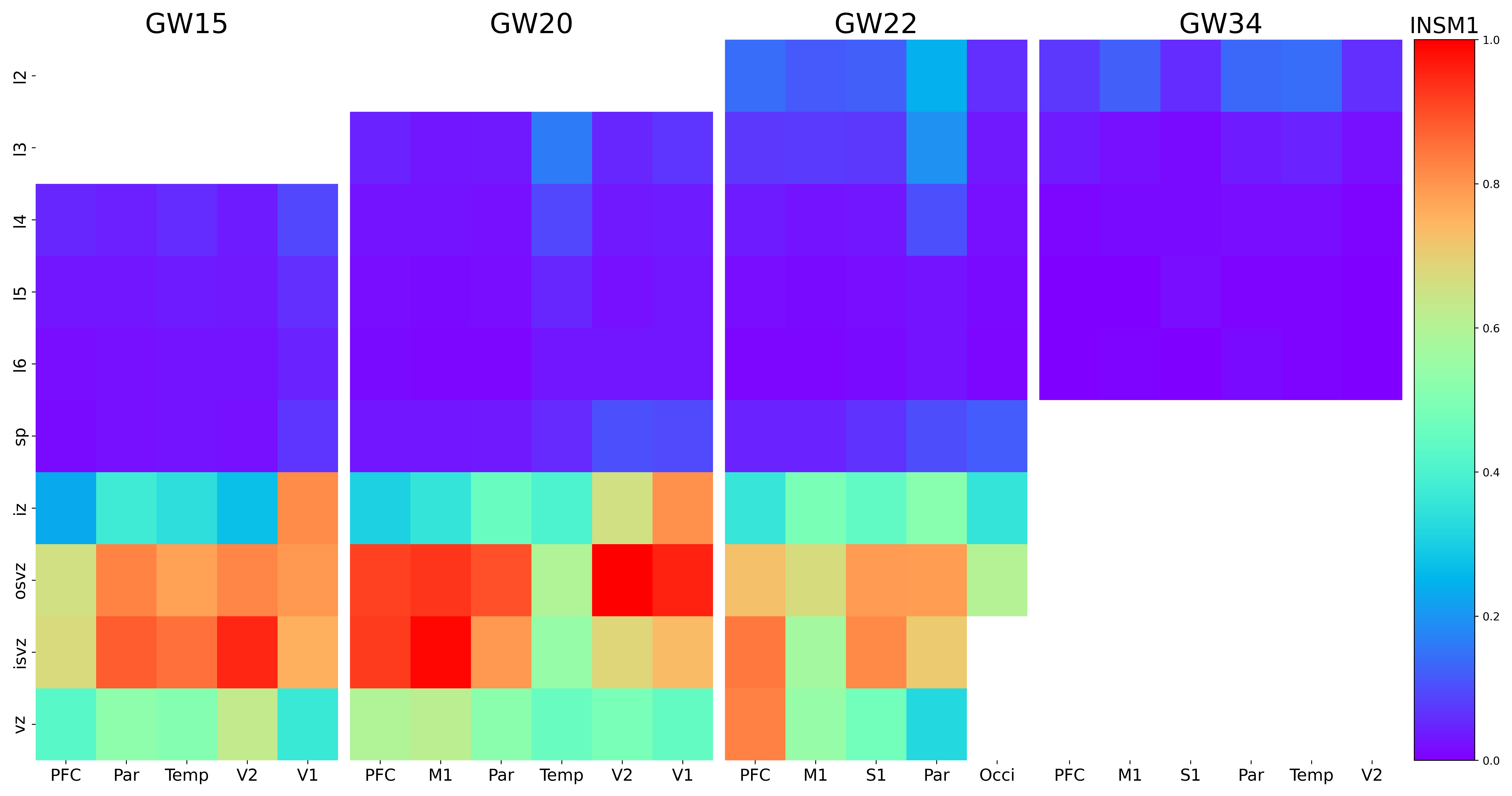

Supplement: Supplementary file 4 — Source Data Fig. 3: Expression pattern heatmap for all 300 genes in the MERFISH. [file 41586_2025_9010_MOESM4_ESM.zip › INSM1.png]

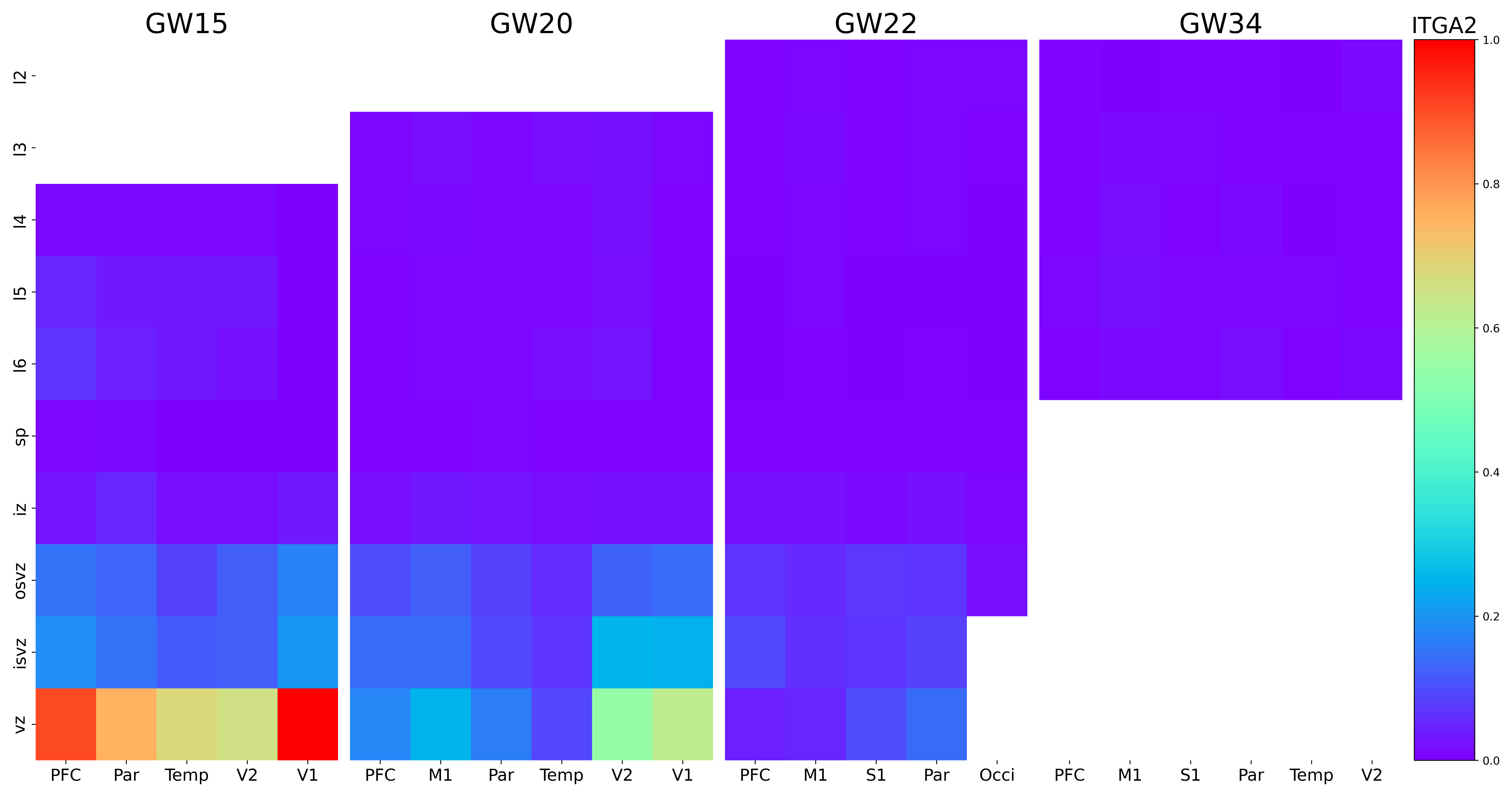

Supplement: Supplementary file 4 — Source Data Fig. 3: Expression pattern heatmap for all 300 genes in the MERFISH. [file 41586_2025_9010_MOESM4_ESM.zip › ITGA2.png]

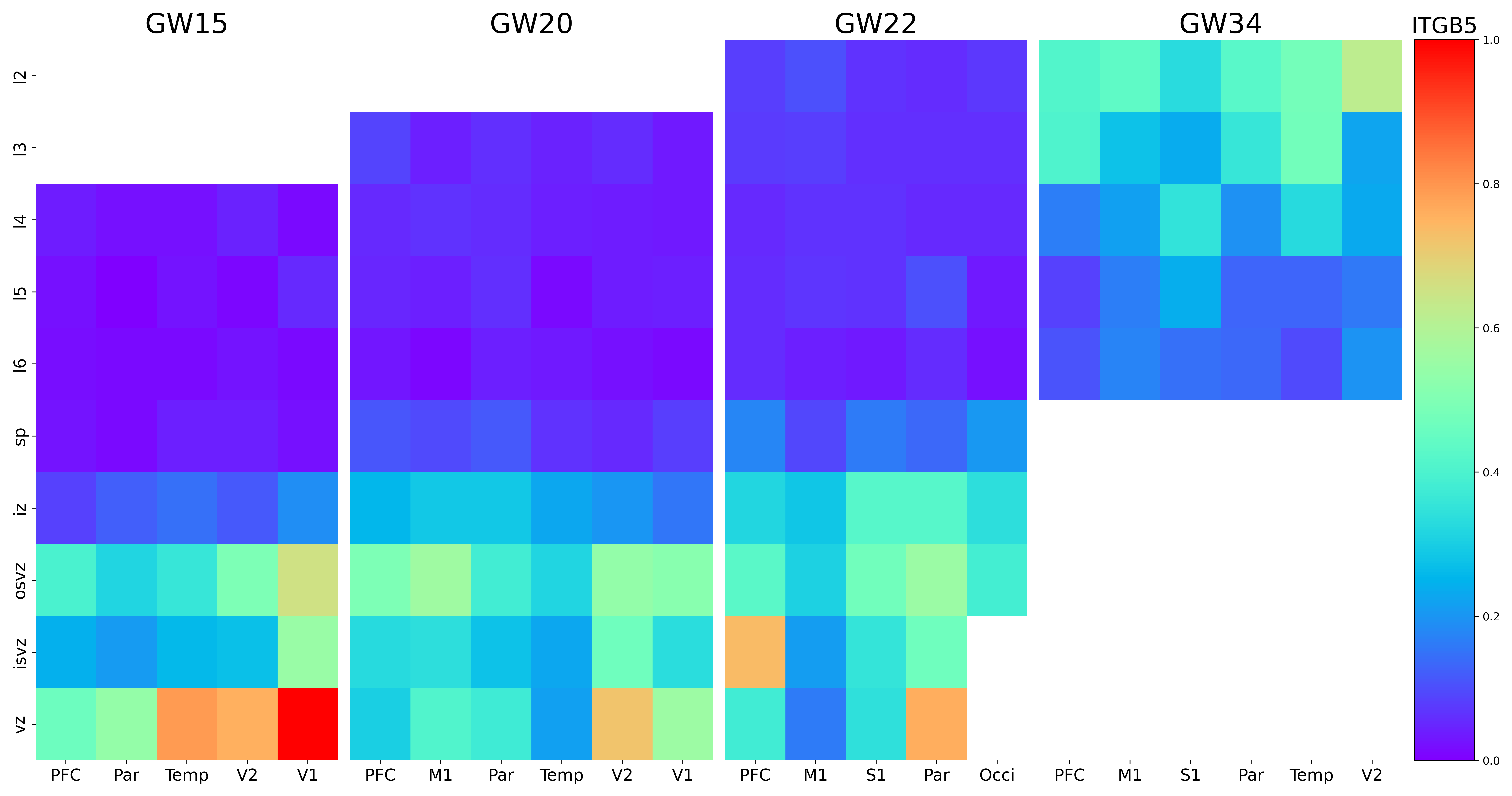

Supplement: Supplementary file 4 — Source Data Fig. 3: Expression pattern heatmap for all 300 genes in the MERFISH. [file 41586_2025_9010_MOESM4_ESM.zip › ITGB5.png]

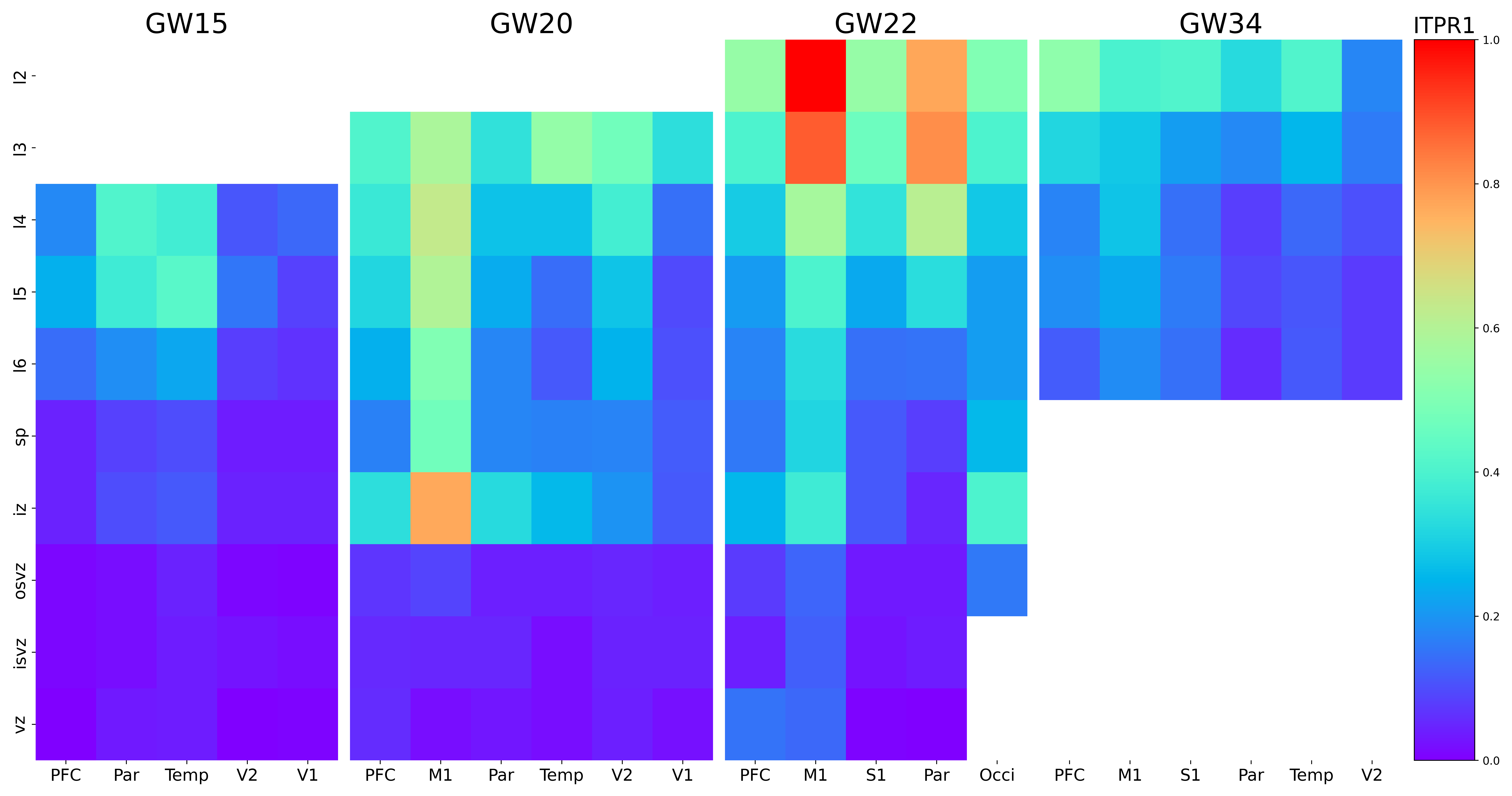

Supplement: Supplementary file 4 — Source Data Fig. 3: Expression pattern heatmap for all 300 genes in the MERFISH. [file 41586_2025_9010_MOESM4_ESM.zip › ITPR1.png]

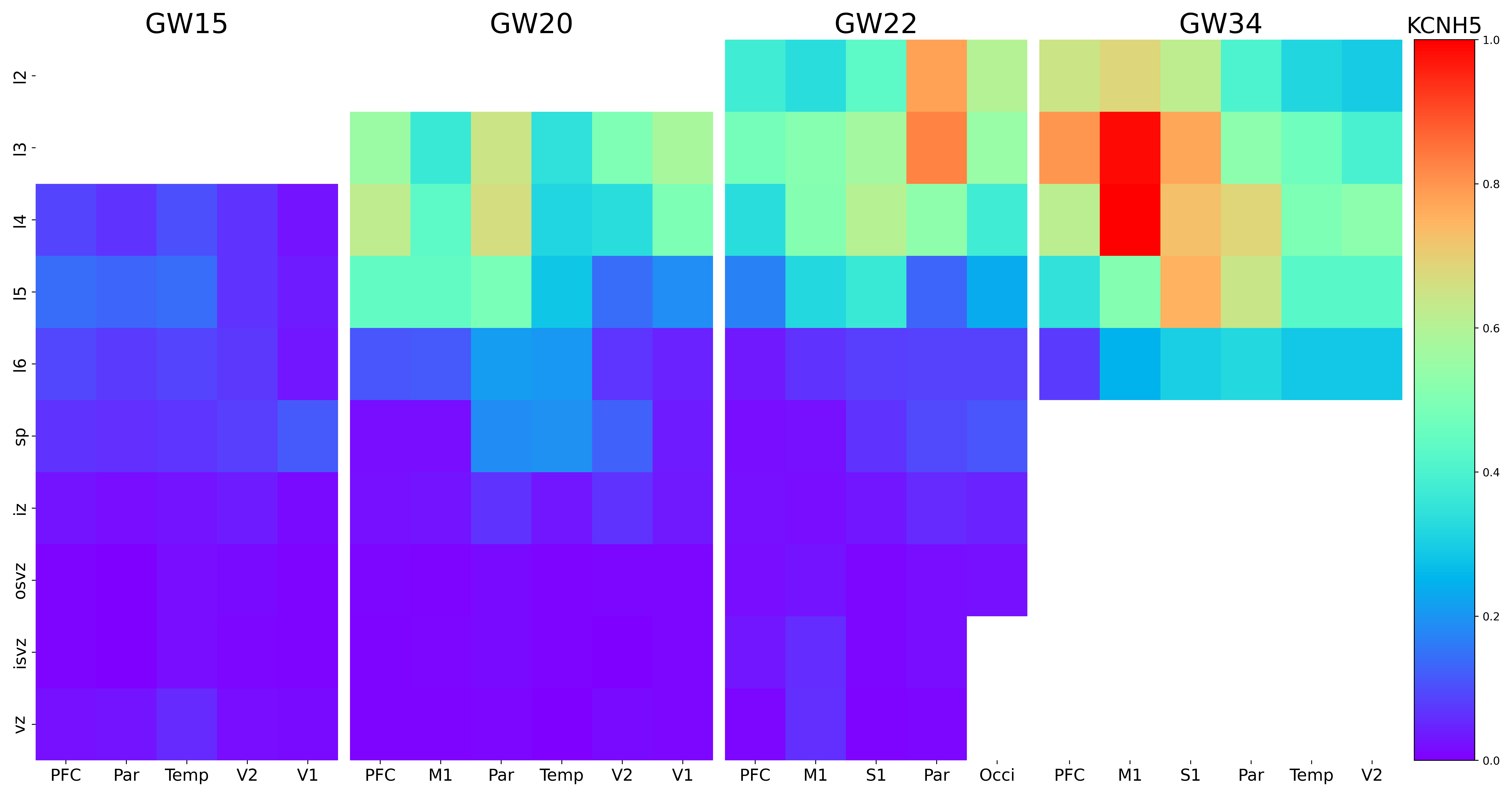

Supplement: Supplementary file 4 — Source Data Fig. 3: Expression pattern heatmap for all 300 genes in the MERFISH. [file 41586_2025_9010_MOESM4_ESM.zip › KCNH5.png]

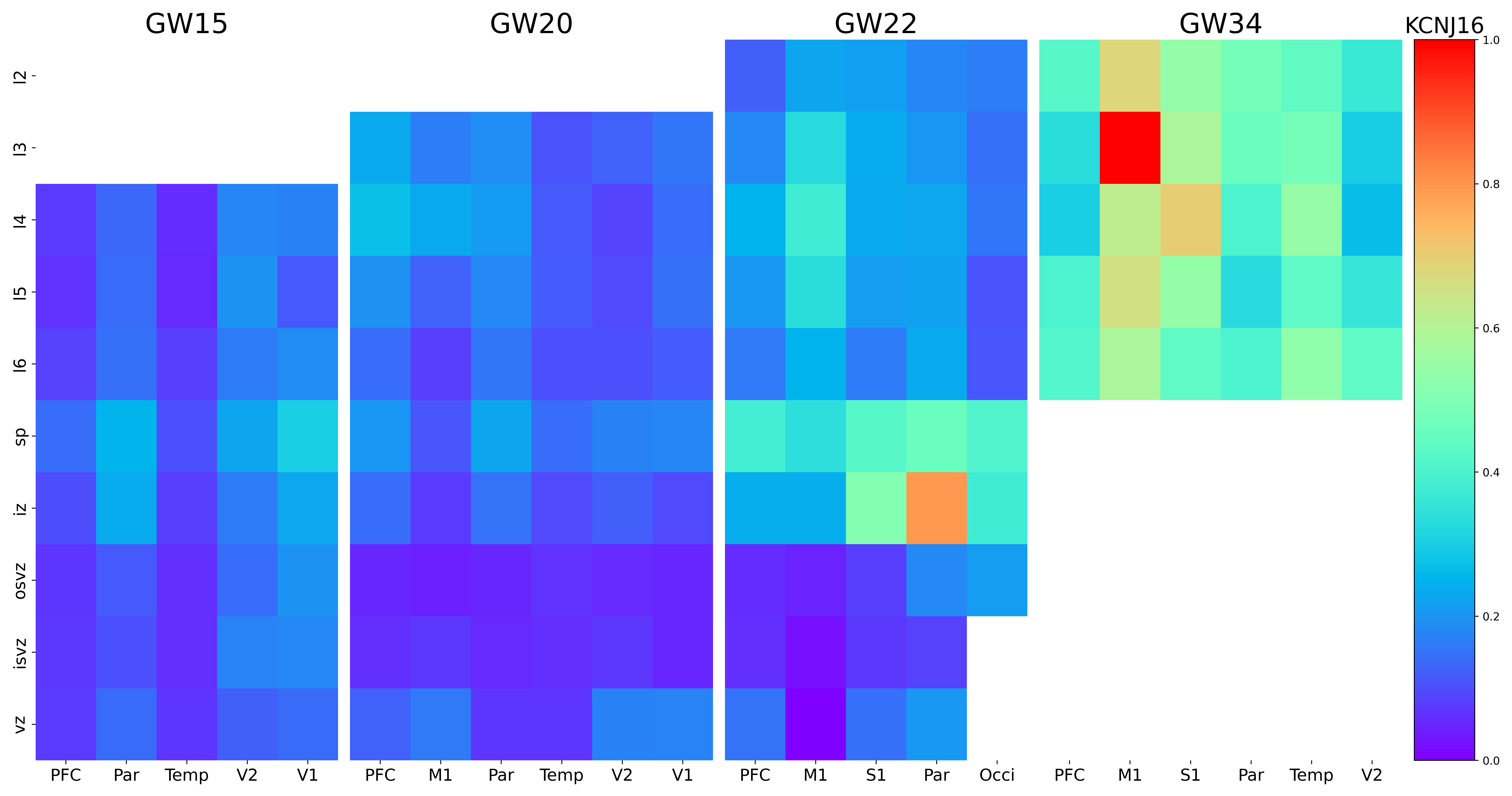

Supplement: Supplementary file 4 — Source Data Fig. 3: Expression pattern heatmap for all 300 genes in the MERFISH. [file 41586_2025_9010_MOESM4_ESM.zip › KCNJ16.png]

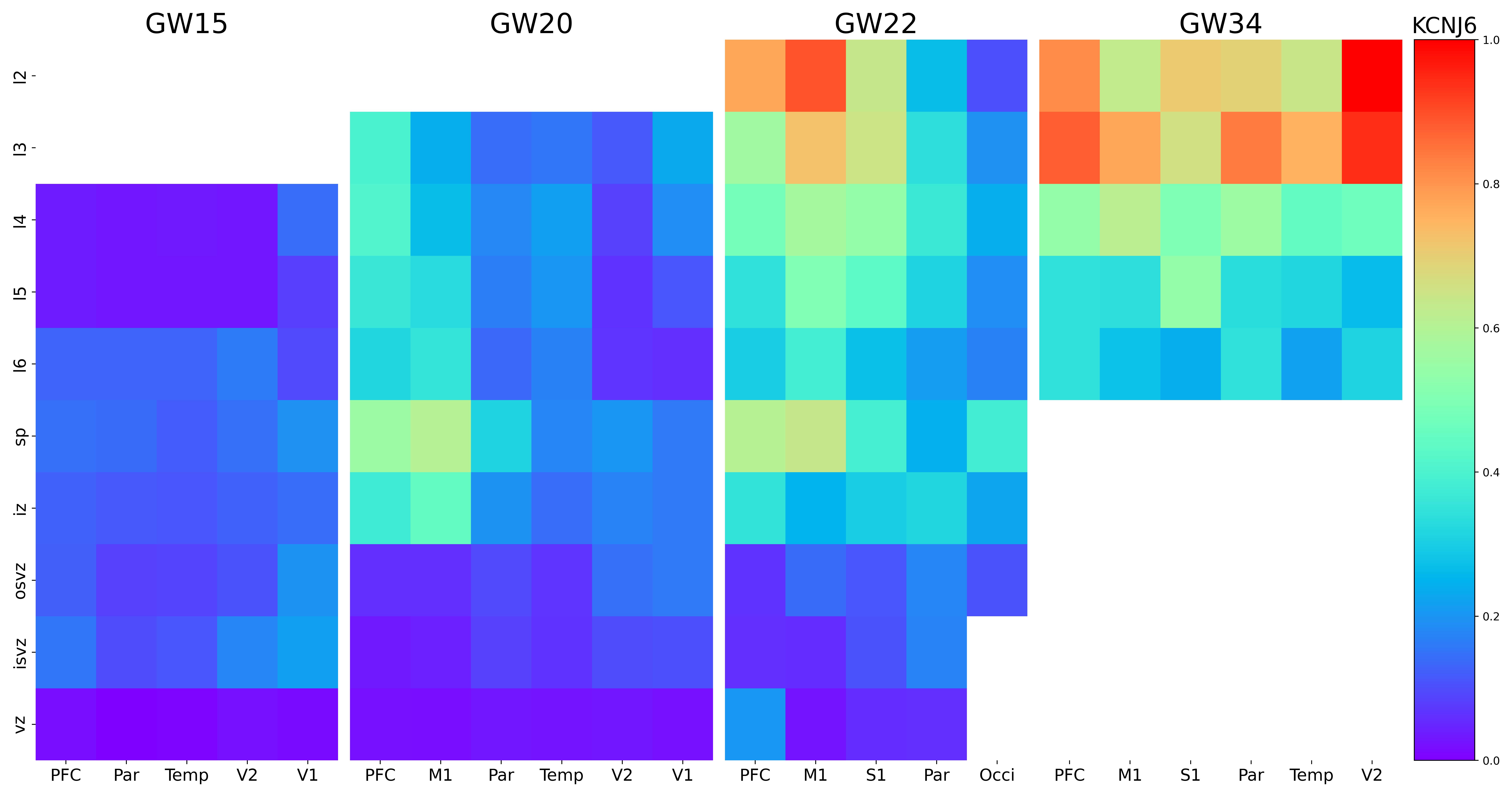

Supplement: Supplementary file 4 — Source Data Fig. 3: Expression pattern heatmap for all 300 genes in the MERFISH. [file 41586_2025_9010_MOESM4_ESM.zip › KCNJ6.png]

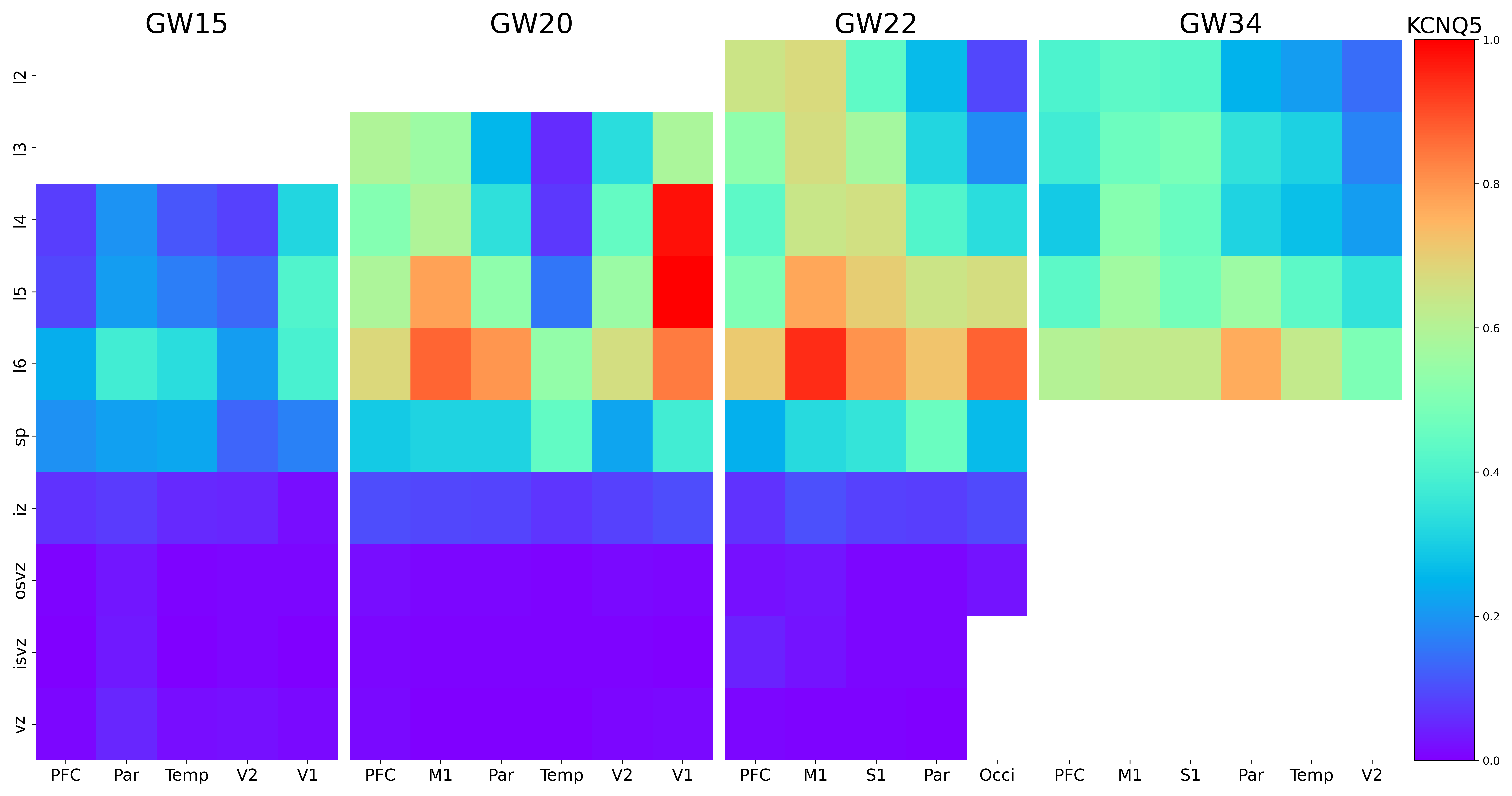

Supplement: Supplementary file 4 — Source Data Fig. 3: Expression pattern heatmap for all 300 genes in the MERFISH. [file 41586_2025_9010_MOESM4_ESM.zip › KCNQ5.png]

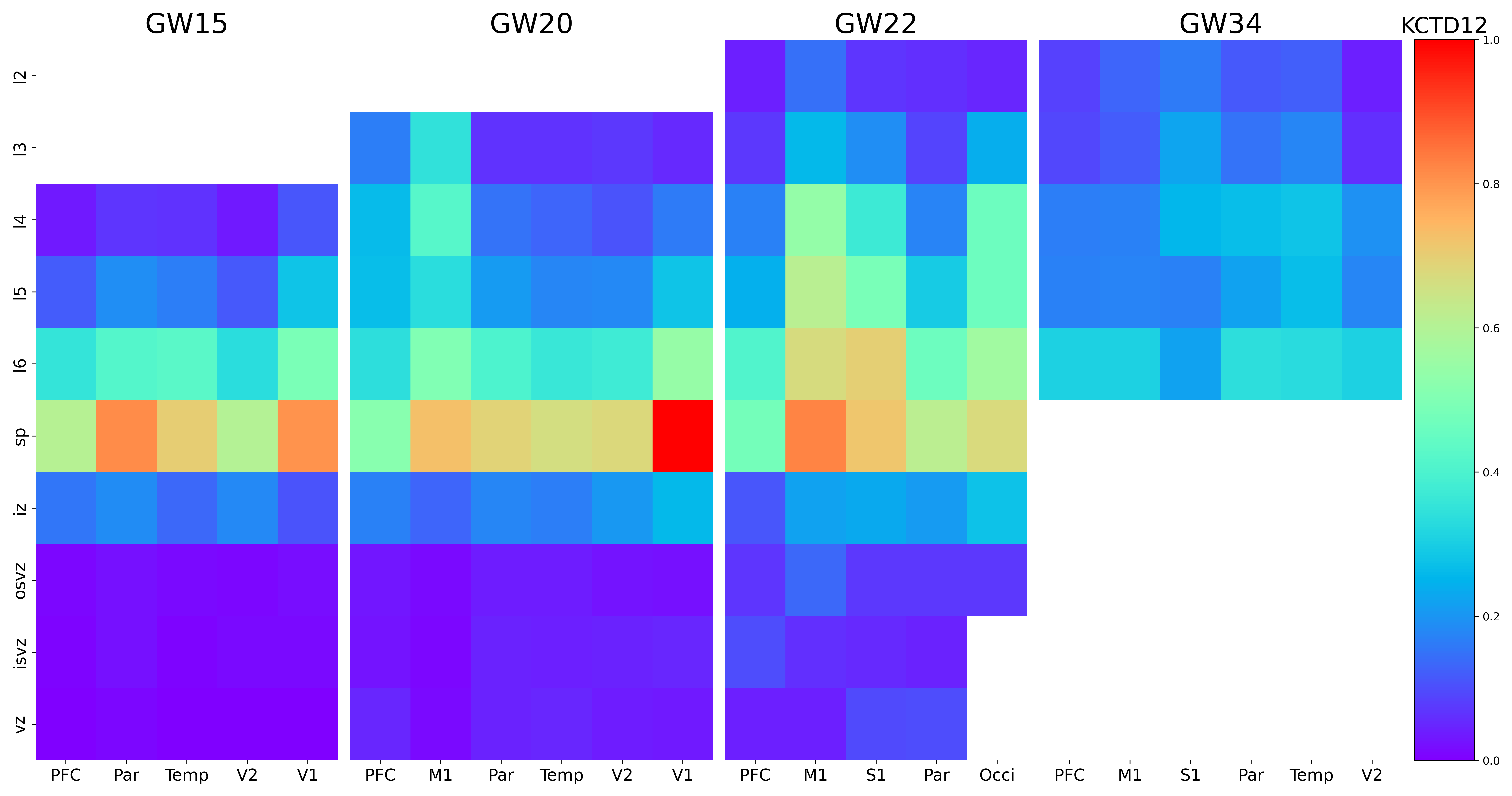

Supplement: Supplementary file 4 — Source Data Fig. 3: Expression pattern heatmap for all 300 genes in the MERFISH. [file 41586_2025_9010_MOESM4_ESM.zip › KCTD12.png]

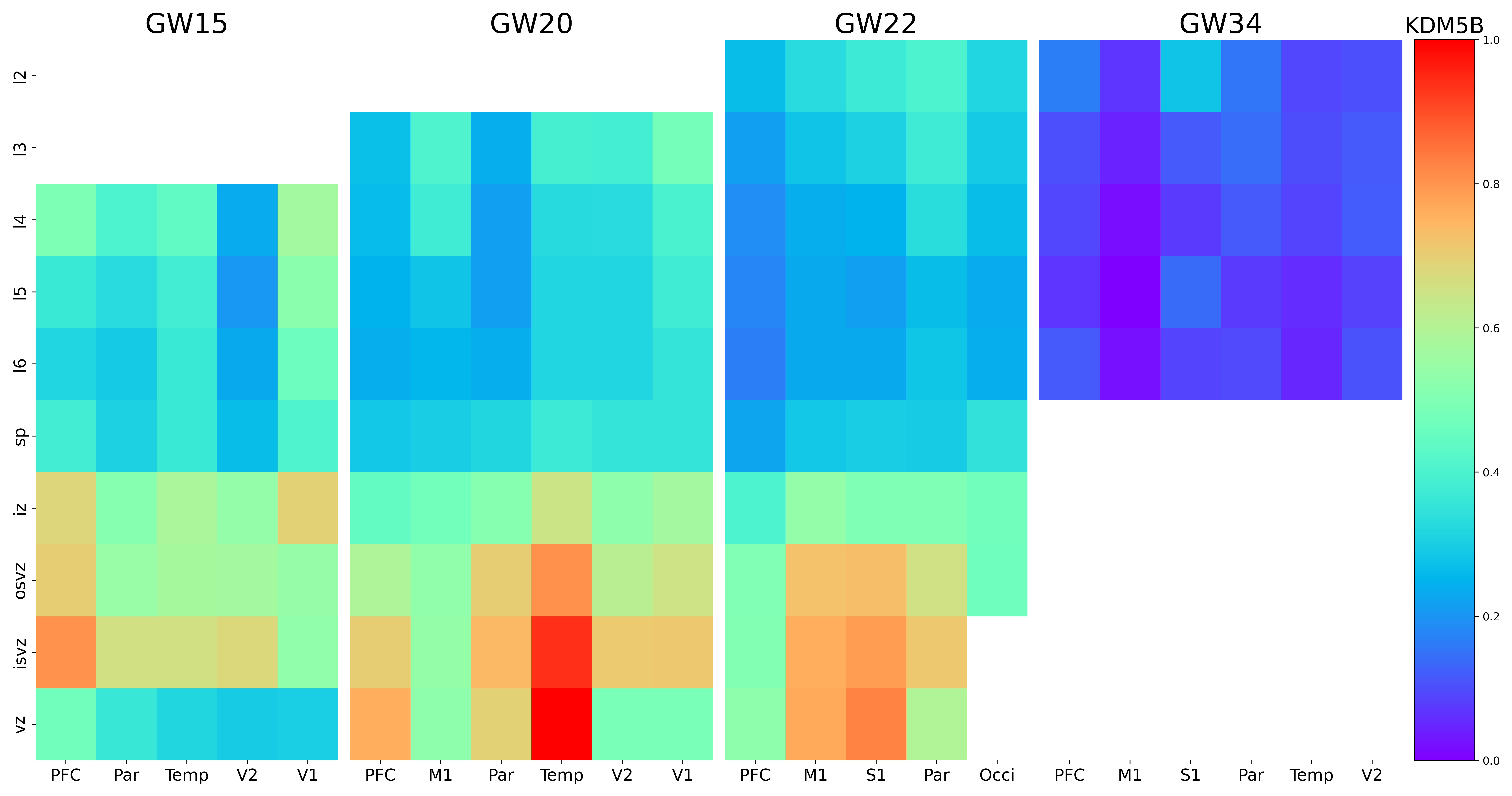

Supplement: Supplementary file 4 — Source Data Fig. 3: Expression pattern heatmap for all 300 genes in the MERFISH. [file 41586_2025_9010_MOESM4_ESM.zip › KDM5B.png]

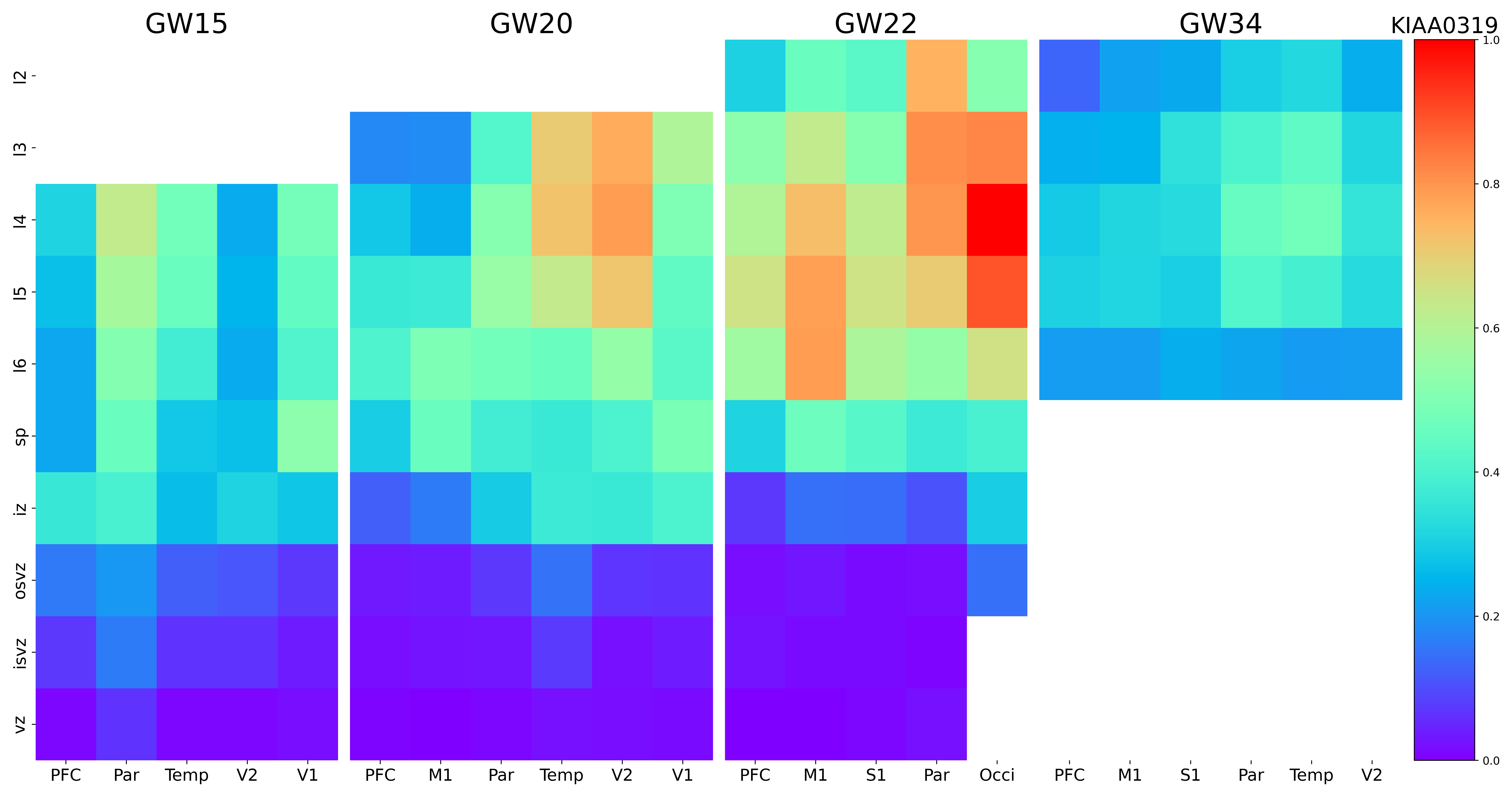

Supplement: Supplementary file 4 — Source Data Fig. 3: Expression pattern heatmap for all 300 genes in the MERFISH. [file 41586_2025_9010_MOESM4_ESM.zip › KIAA0319.png]

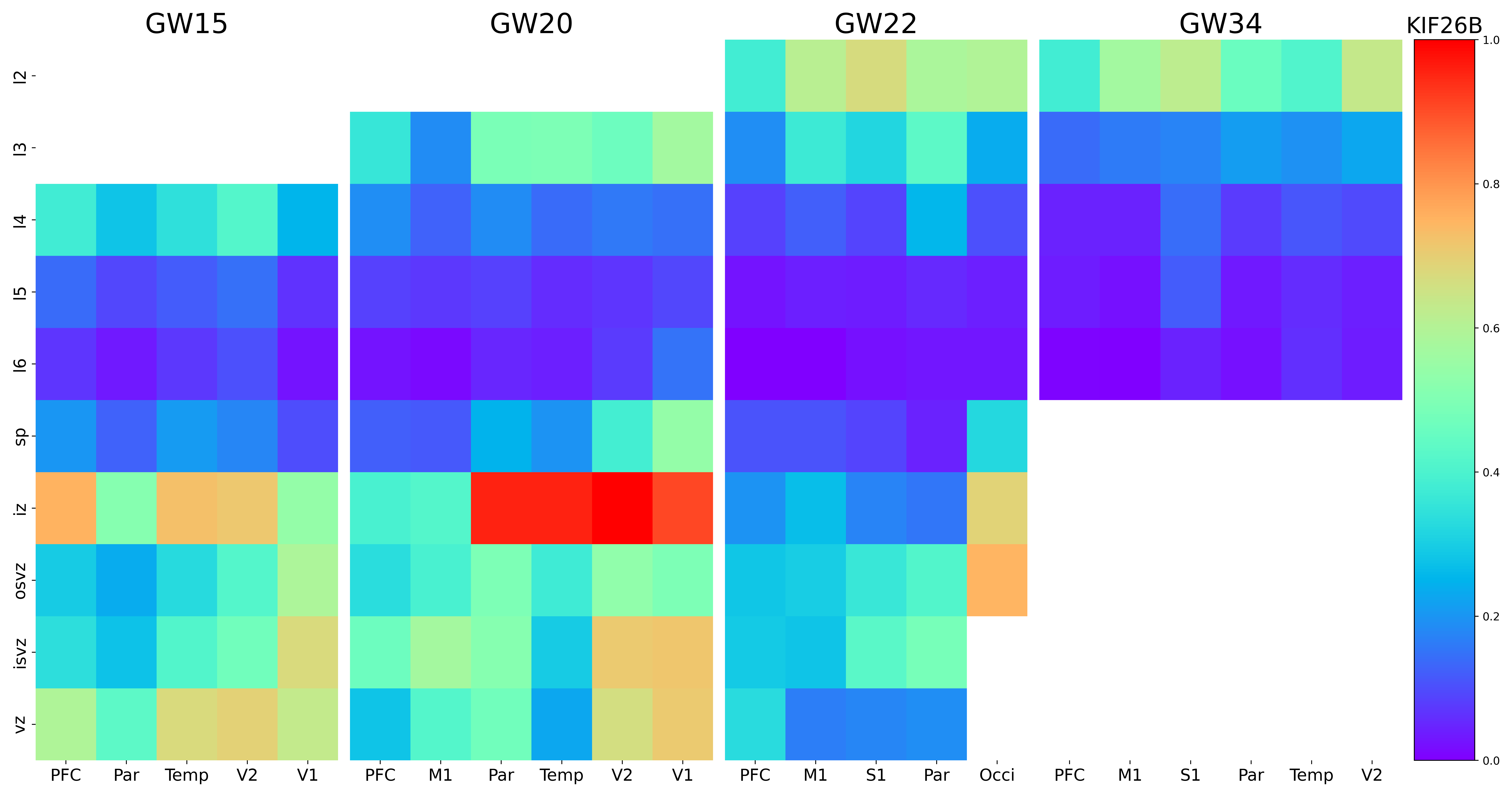

Supplement: Supplementary file 4 — Source Data Fig. 3: Expression pattern heatmap for all 300 genes in the MERFISH. [file 41586_2025_9010_MOESM4_ESM.zip › KIF26B.png]

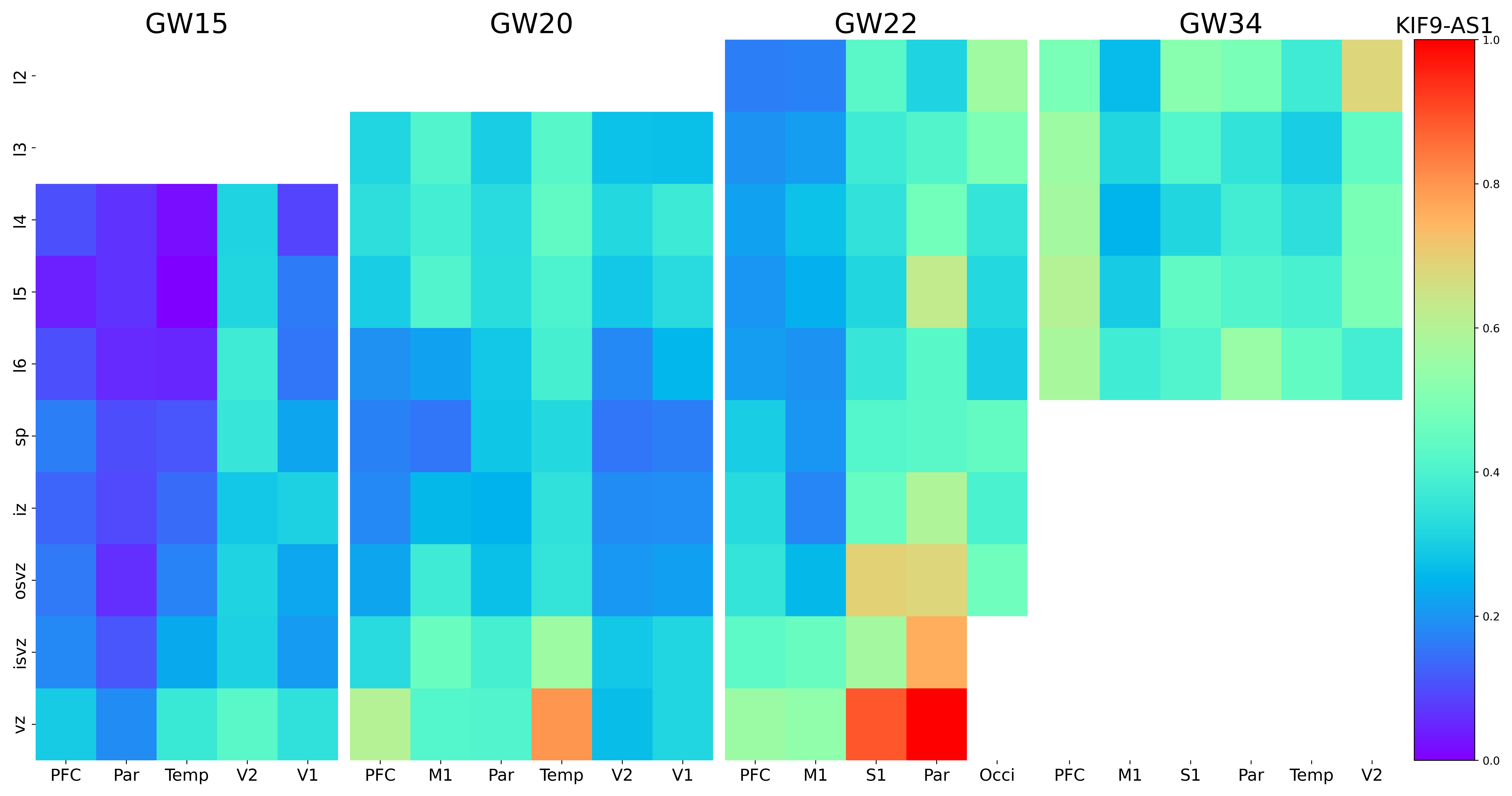

Supplement: Supplementary file 4 — Source Data Fig. 3: Expression pattern heatmap for all 300 genes in the MERFISH. [file 41586_2025_9010_MOESM4_ESM.zip › KIF9-AS1.png]

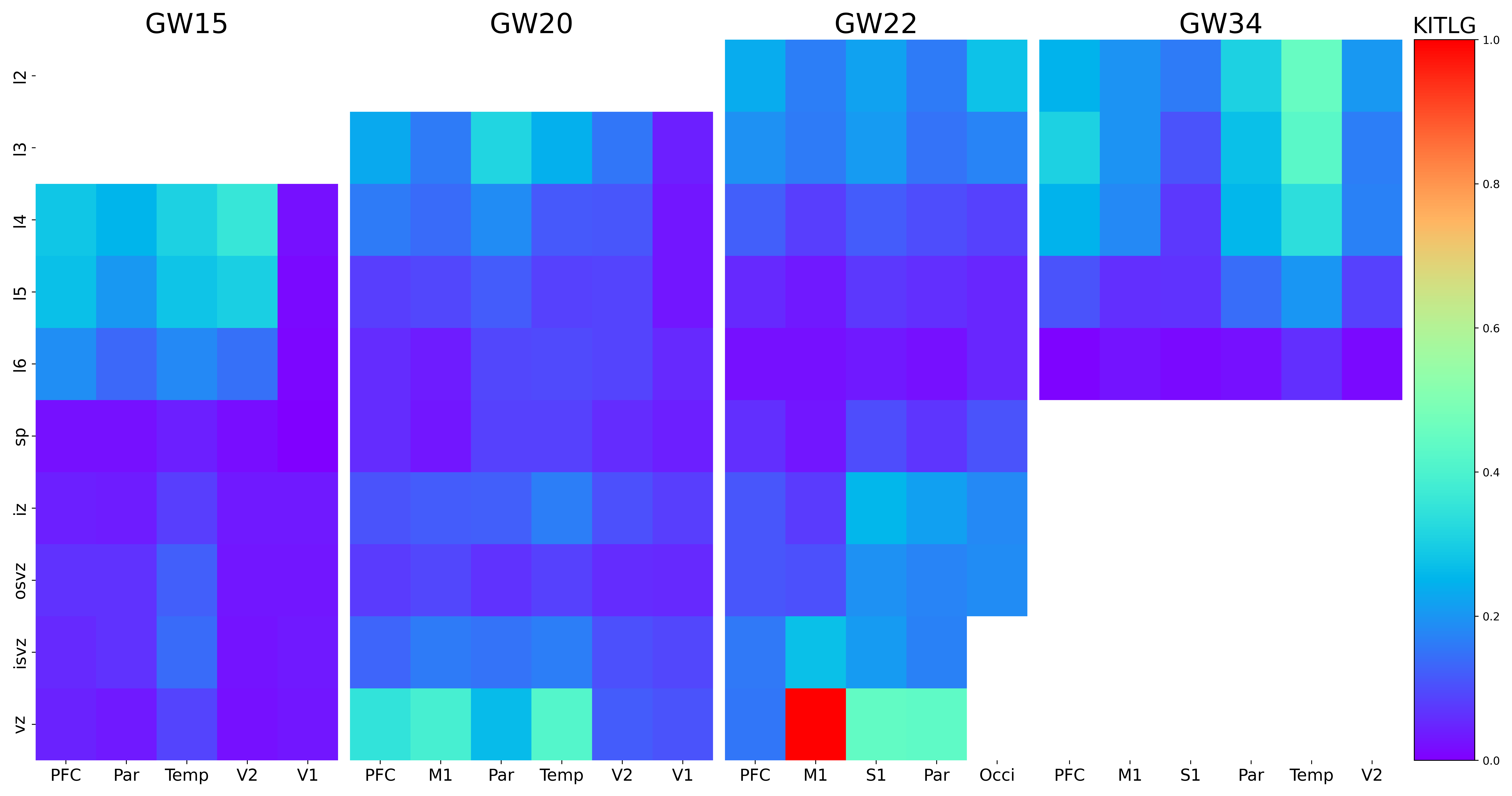

Supplement: Supplementary file 4 — Source Data Fig. 3: Expression pattern heatmap for all 300 genes in the MERFISH. [file 41586_2025_9010_MOESM4_ESM.zip › KITLG.png]

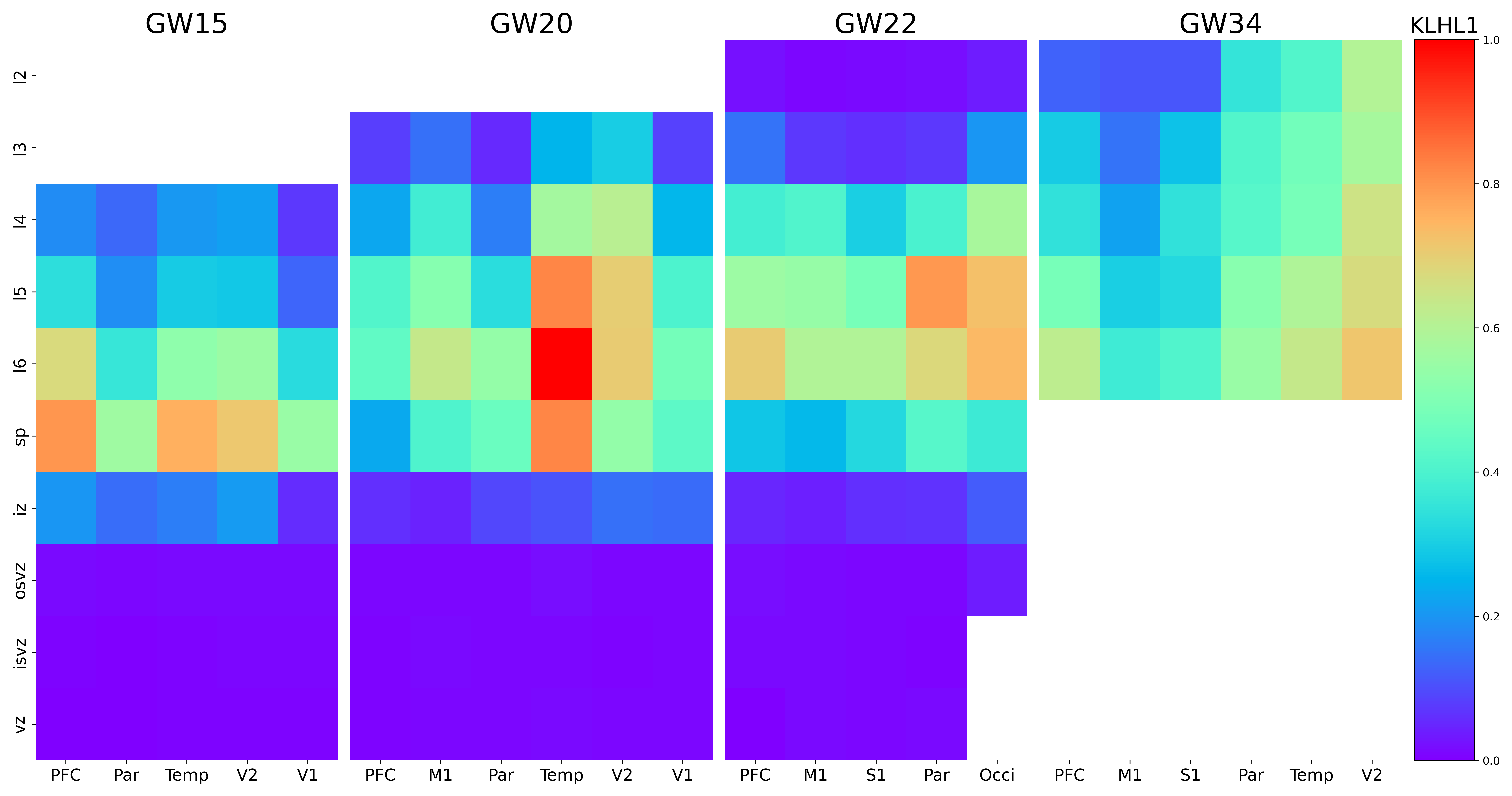

Supplement: Supplementary file 4 — Source Data Fig. 3: Expression pattern heatmap for all 300 genes in the MERFISH. [file 41586_2025_9010_MOESM4_ESM.zip › KLHL1.png]

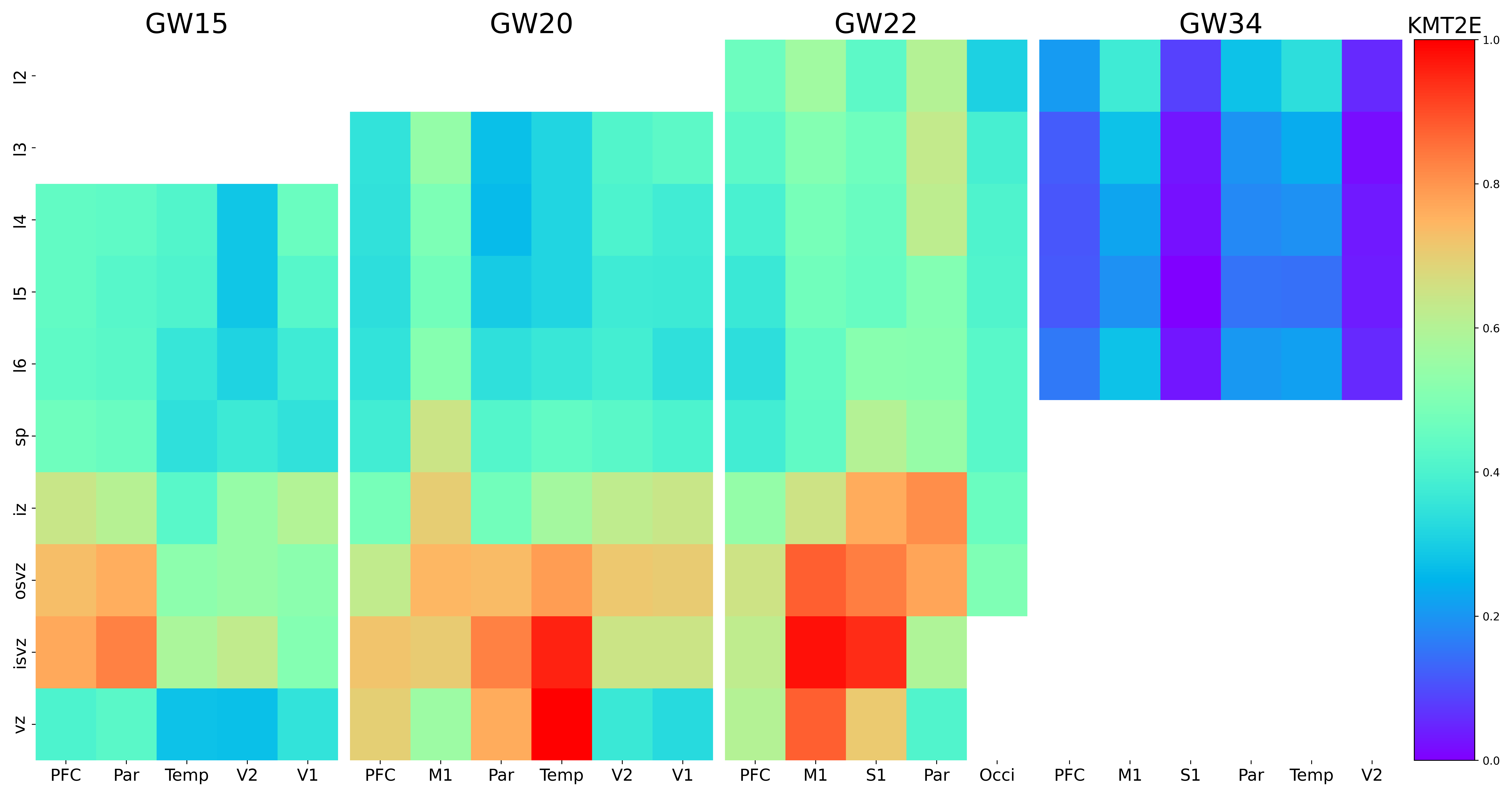

Supplement: Supplementary file 4 — Source Data Fig. 3: Expression pattern heatmap for all 300 genes in the MERFISH. [file 41586_2025_9010_MOESM4_ESM.zip › KMT2E.png]

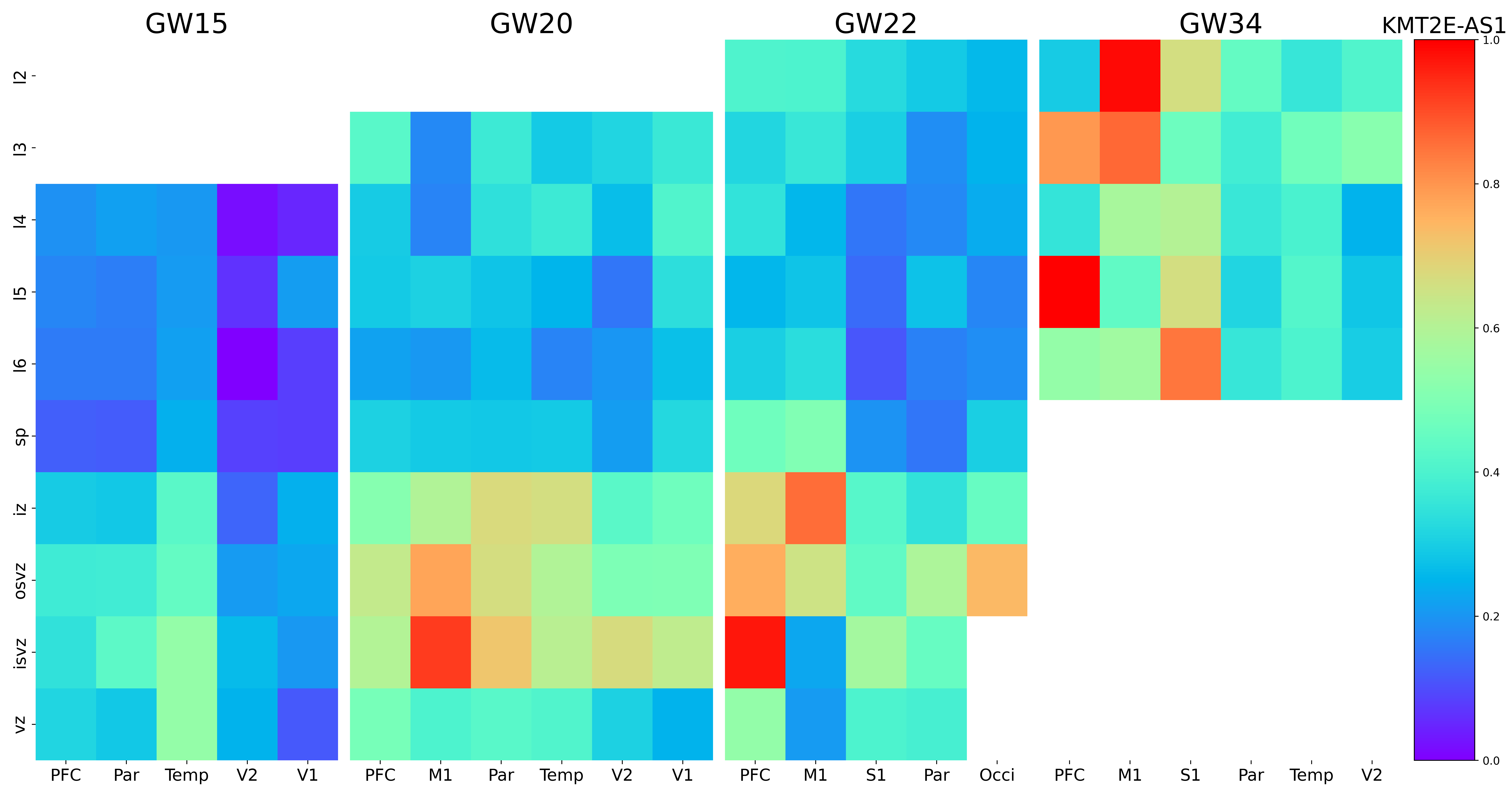

Supplement: Supplementary file 4 — Source Data Fig. 3: Expression pattern heatmap for all 300 genes in the MERFISH. [file 41586_2025_9010_MOESM4_ESM.zip › KMT2E-AS1.png]

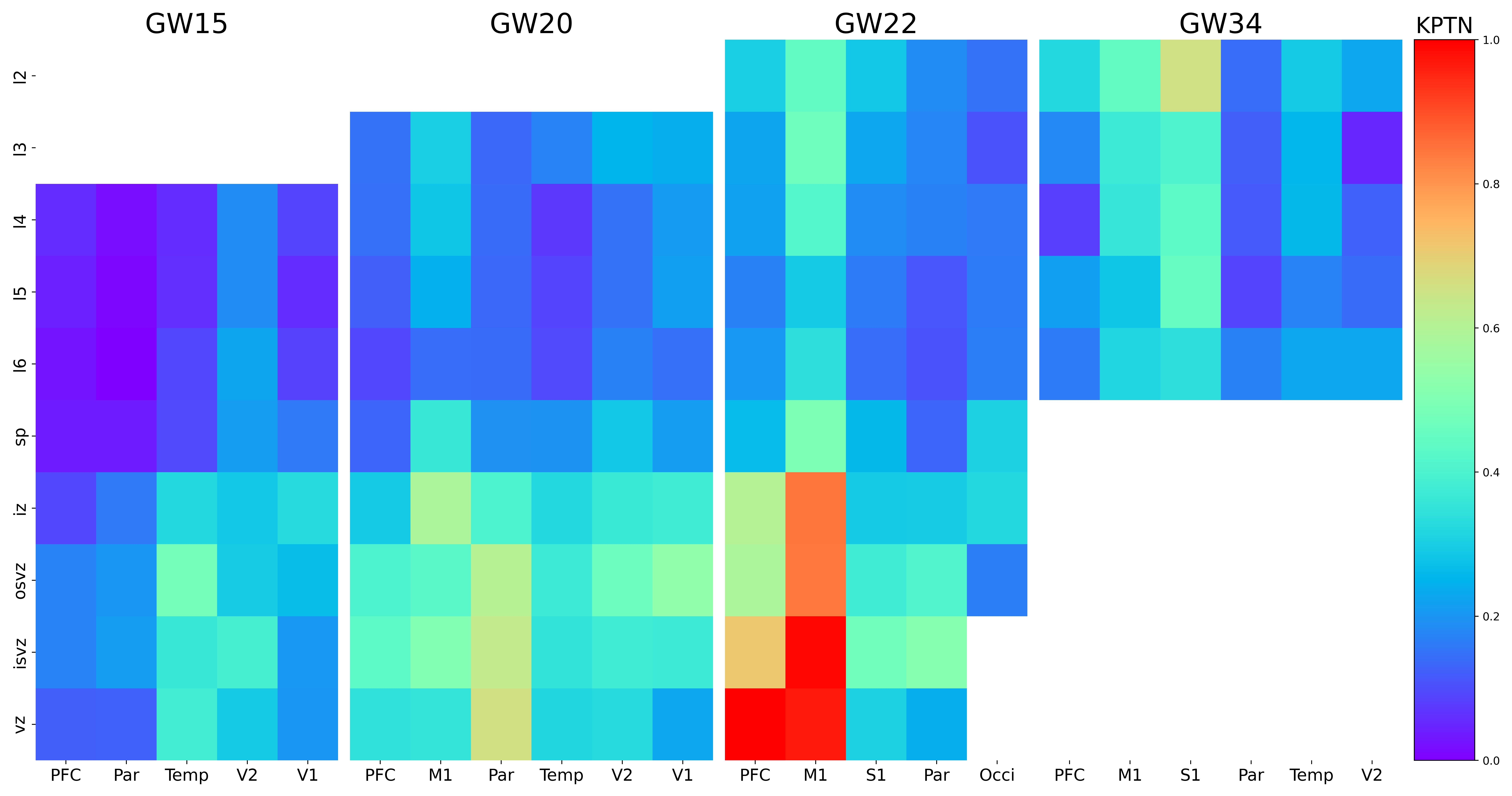

Supplement: Supplementary file 4 — Source Data Fig. 3: Expression pattern heatmap for all 300 genes in the MERFISH. [file 41586_2025_9010_MOESM4_ESM.zip › KPTN.png]

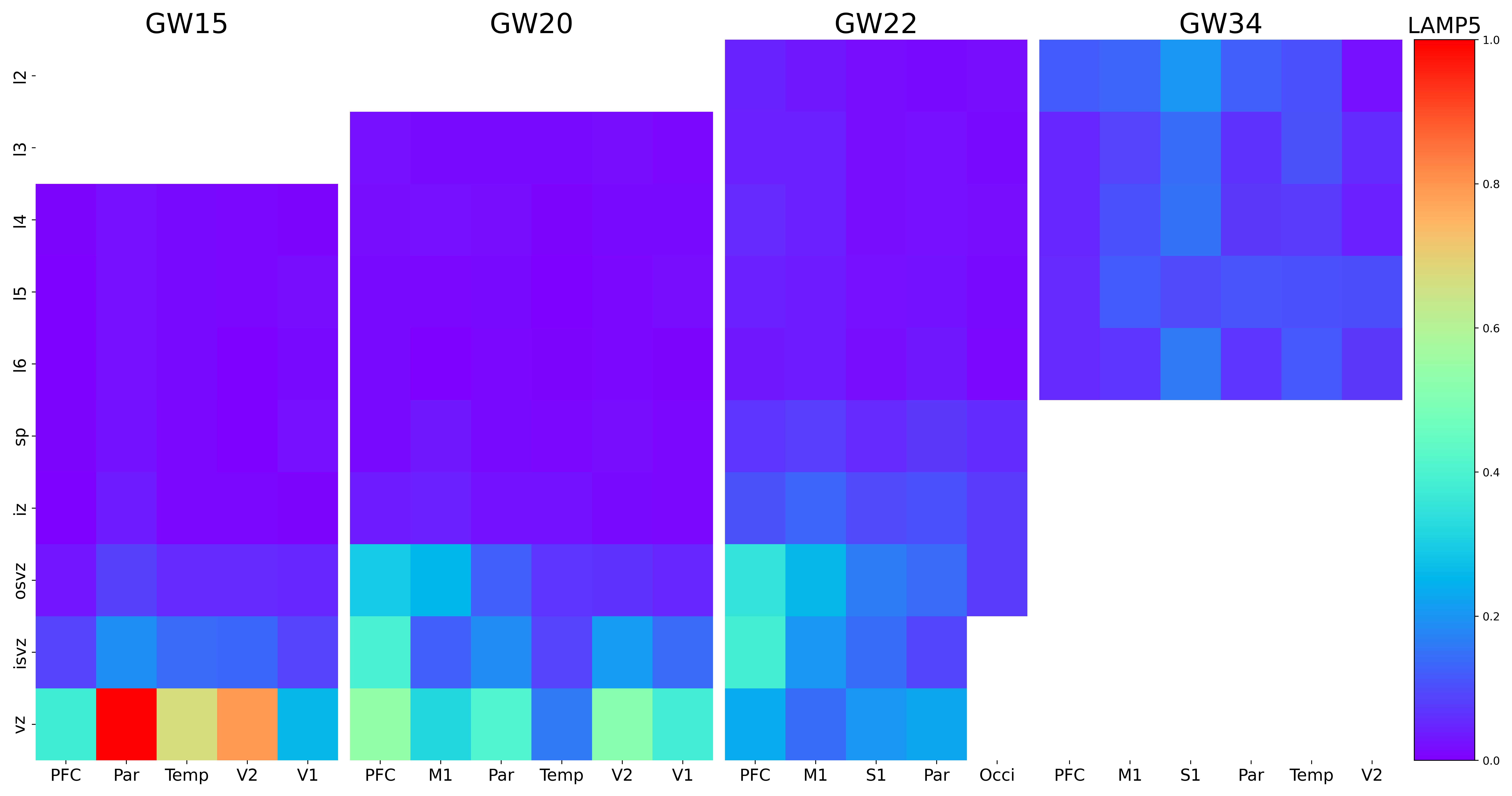

Supplement: Supplementary file 4 — Source Data Fig. 3: Expression pattern heatmap for all 300 genes in the MERFISH. [file 41586_2025_9010_MOESM4_ESM.zip › LAMP5.png]

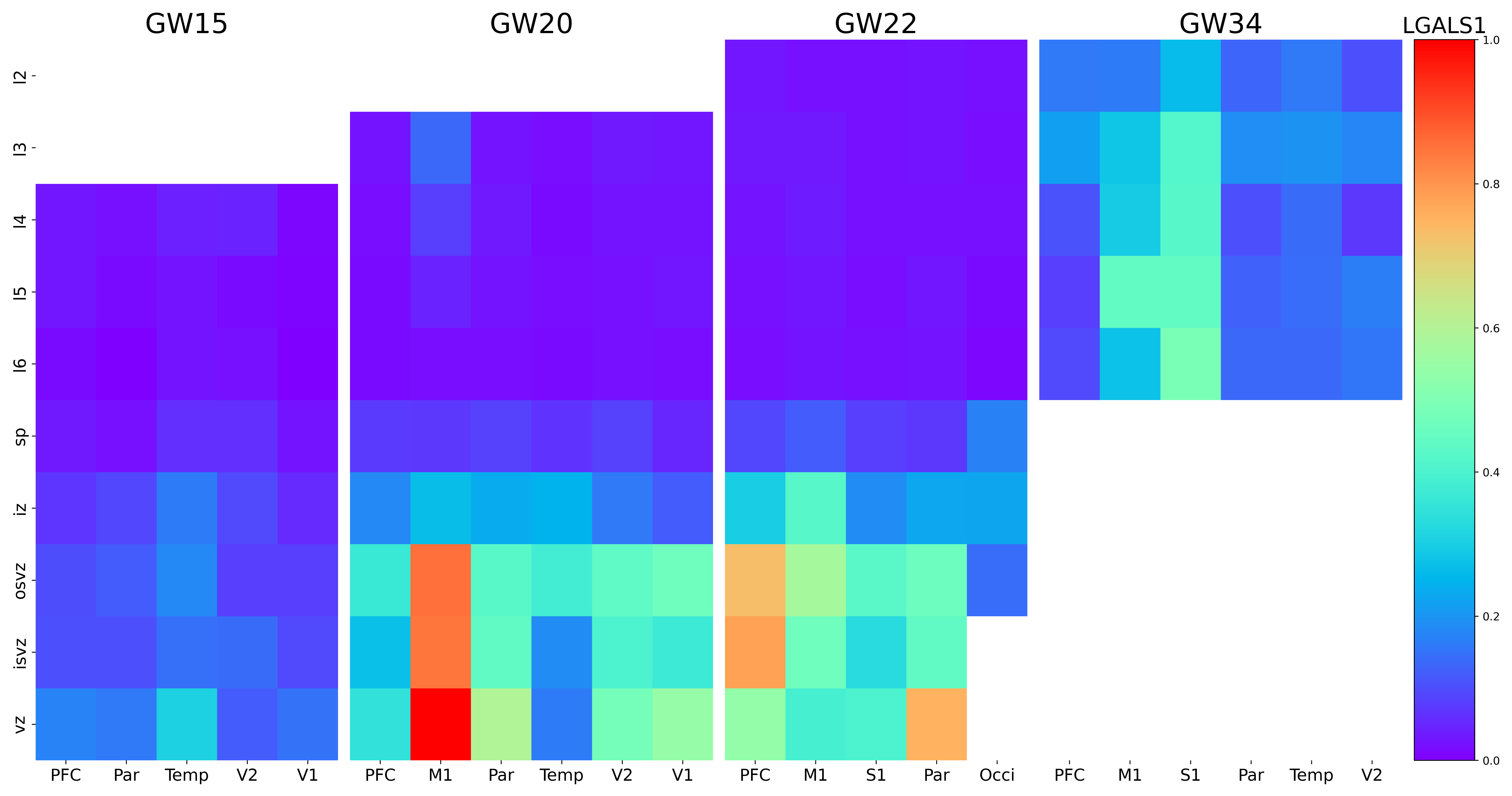

Supplement: Supplementary file 4 — Source Data Fig. 3: Expression pattern heatmap for all 300 genes in the MERFISH. [file 41586_2025_9010_MOESM4_ESM.zip › LGALS1.png]

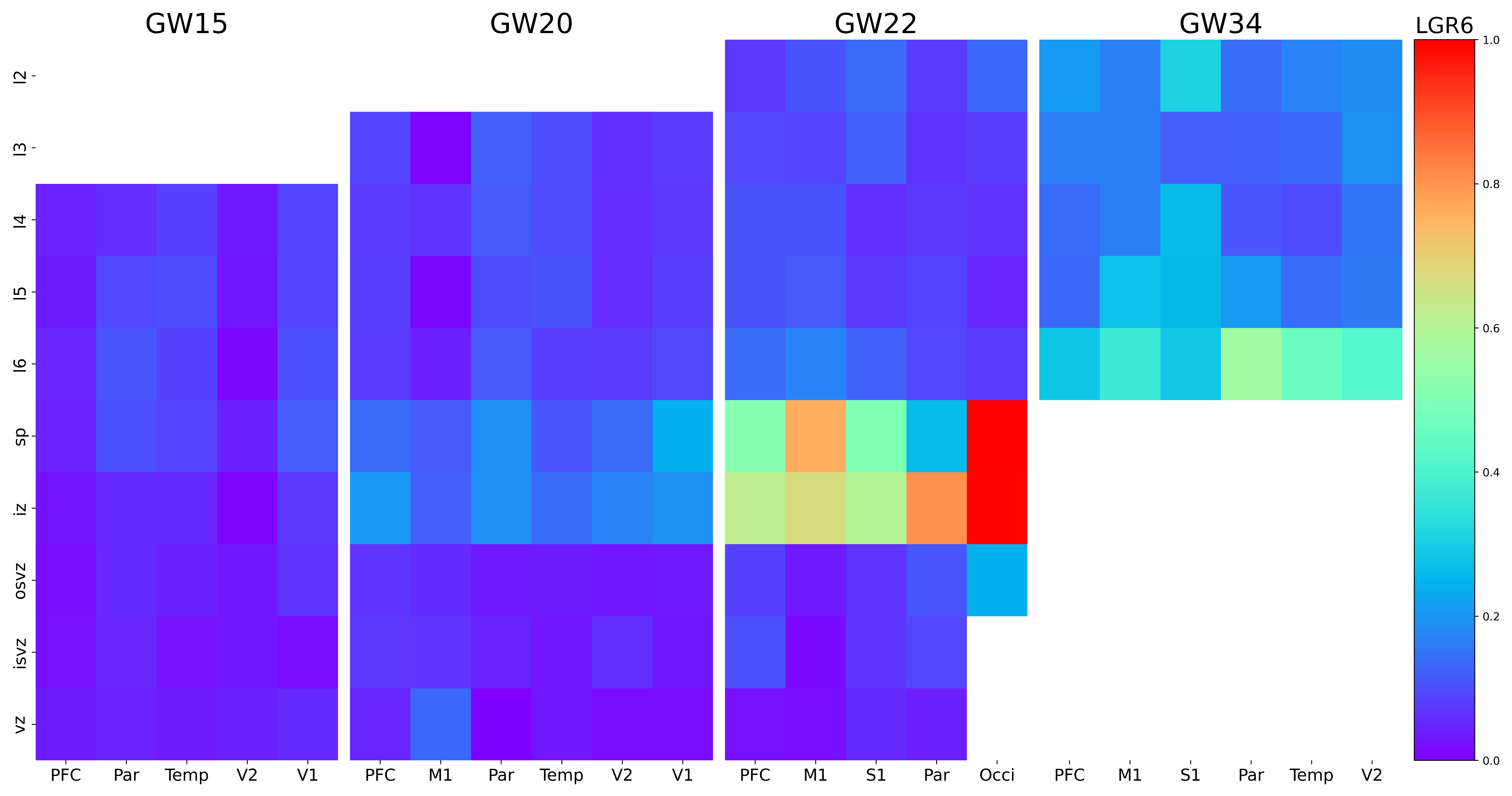

Supplement: Supplementary file 4 — Source Data Fig. 3: Expression pattern heatmap for all 300 genes in the MERFISH. [file 41586_2025_9010_MOESM4_ESM.zip › LGR6.png]

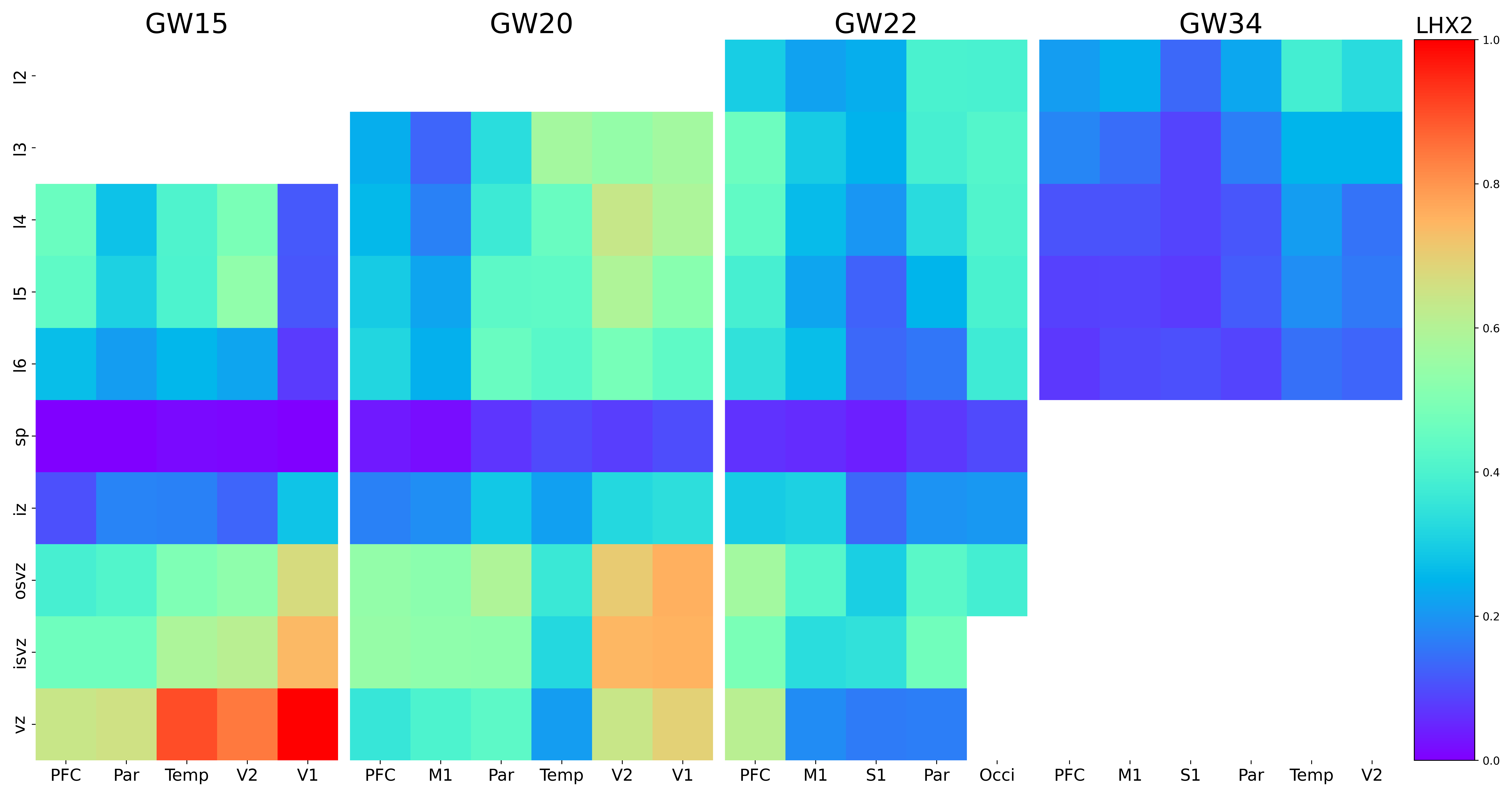

Supplement: Supplementary file 4 — Source Data Fig. 3: Expression pattern heatmap for all 300 genes in the MERFISH. [file 41586_2025_9010_MOESM4_ESM.zip › LHX2.png]

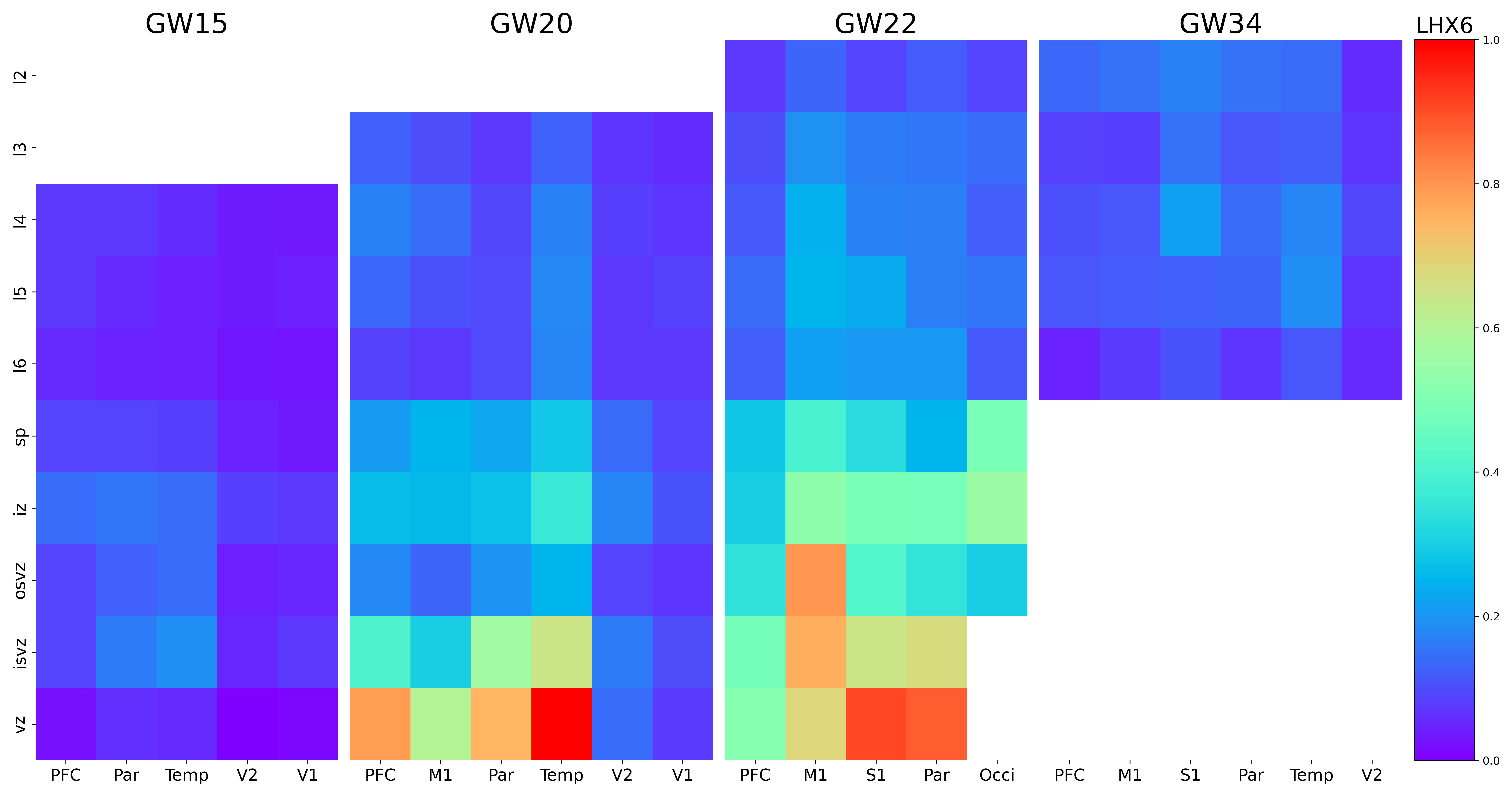

Supplement: Supplementary file 4 — Source Data Fig. 3: Expression pattern heatmap for all 300 genes in the MERFISH. [file 41586_2025_9010_MOESM4_ESM.zip › LHX6.png]

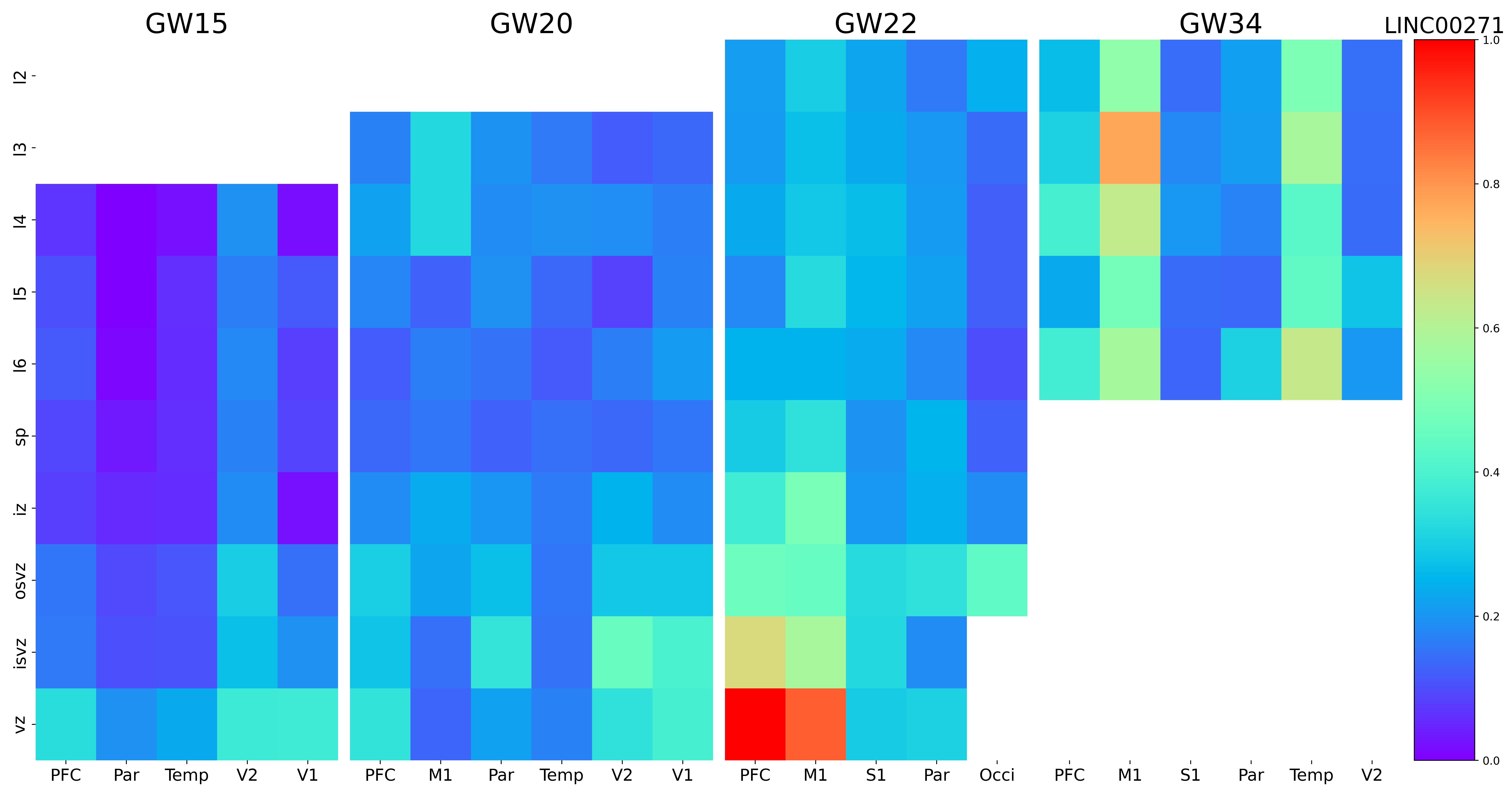

Supplement: Supplementary file 4 — Source Data Fig. 3: Expression pattern heatmap for all 300 genes in the MERFISH. [file 41586_2025_9010_MOESM4_ESM.zip › LINC00271.png]

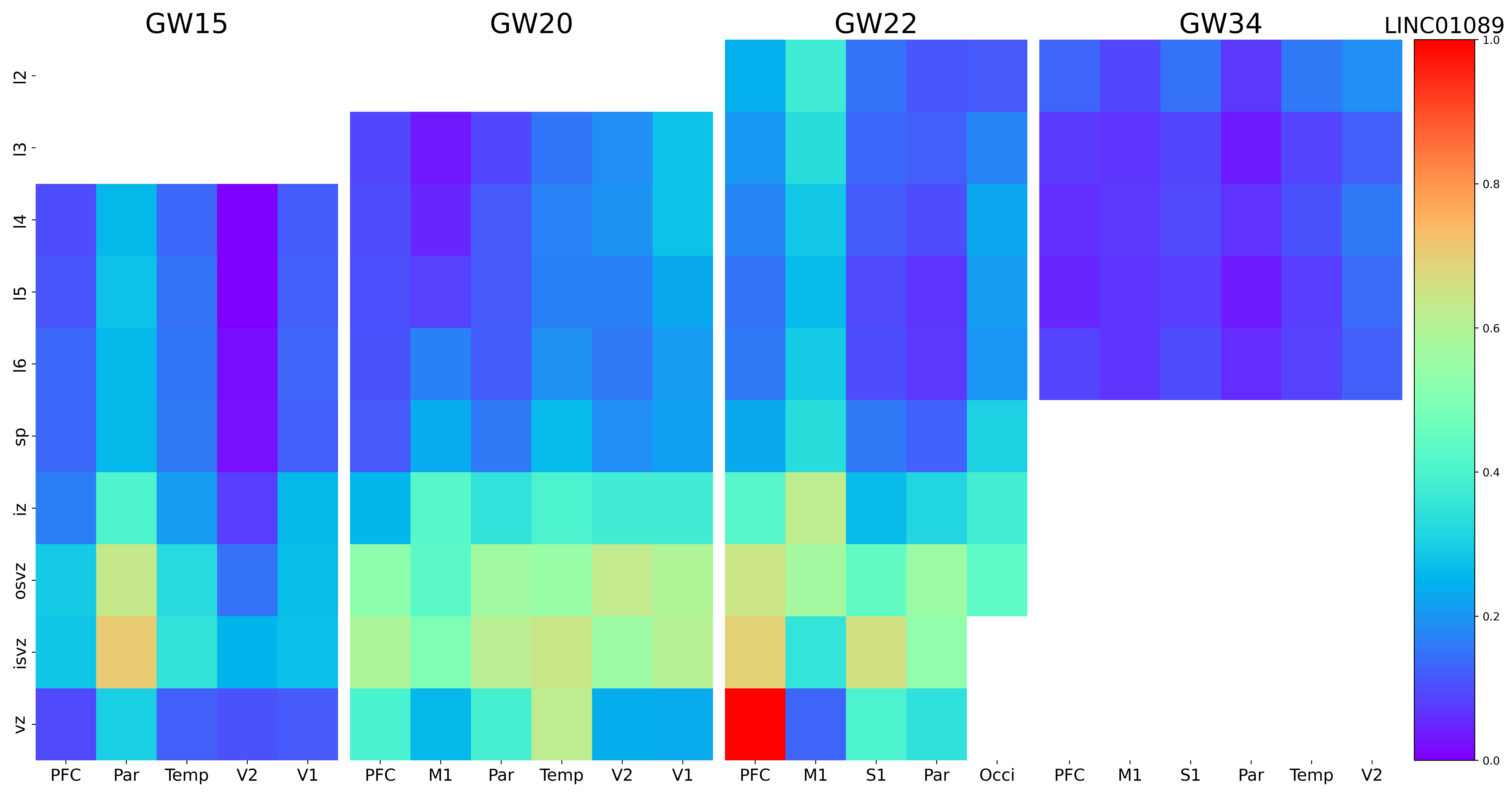

Supplement: Supplementary file 4 — Source Data Fig. 3: Expression pattern heatmap for all 300 genes in the MERFISH. [file 41586_2025_9010_MOESM4_ESM.zip › LINC01089.png]

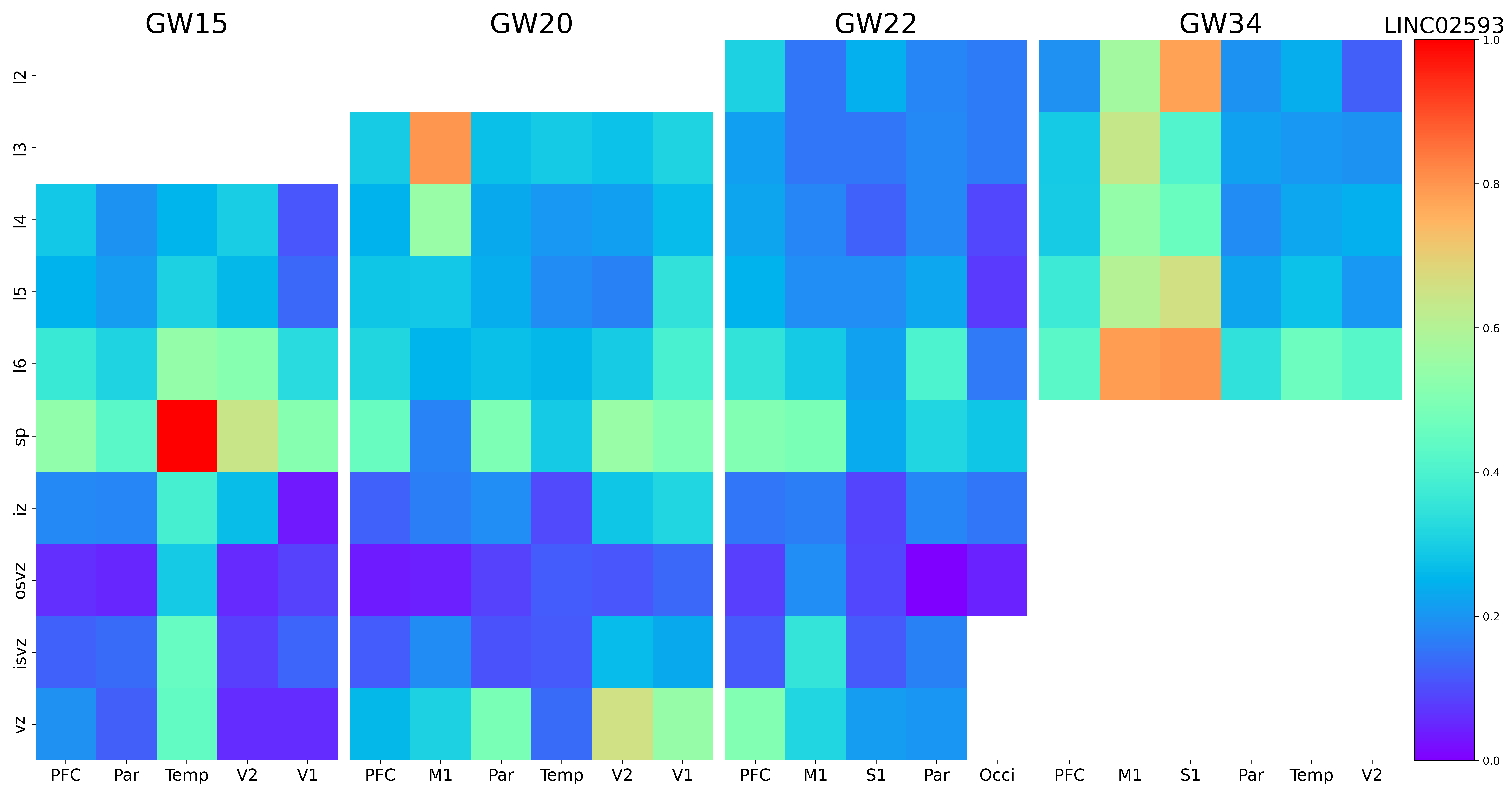

Supplement: Supplementary file 4 — Source Data Fig. 3: Expression pattern heatmap for all 300 genes in the MERFISH. [file 41586_2025_9010_MOESM4_ESM.zip › LINC02593.png]

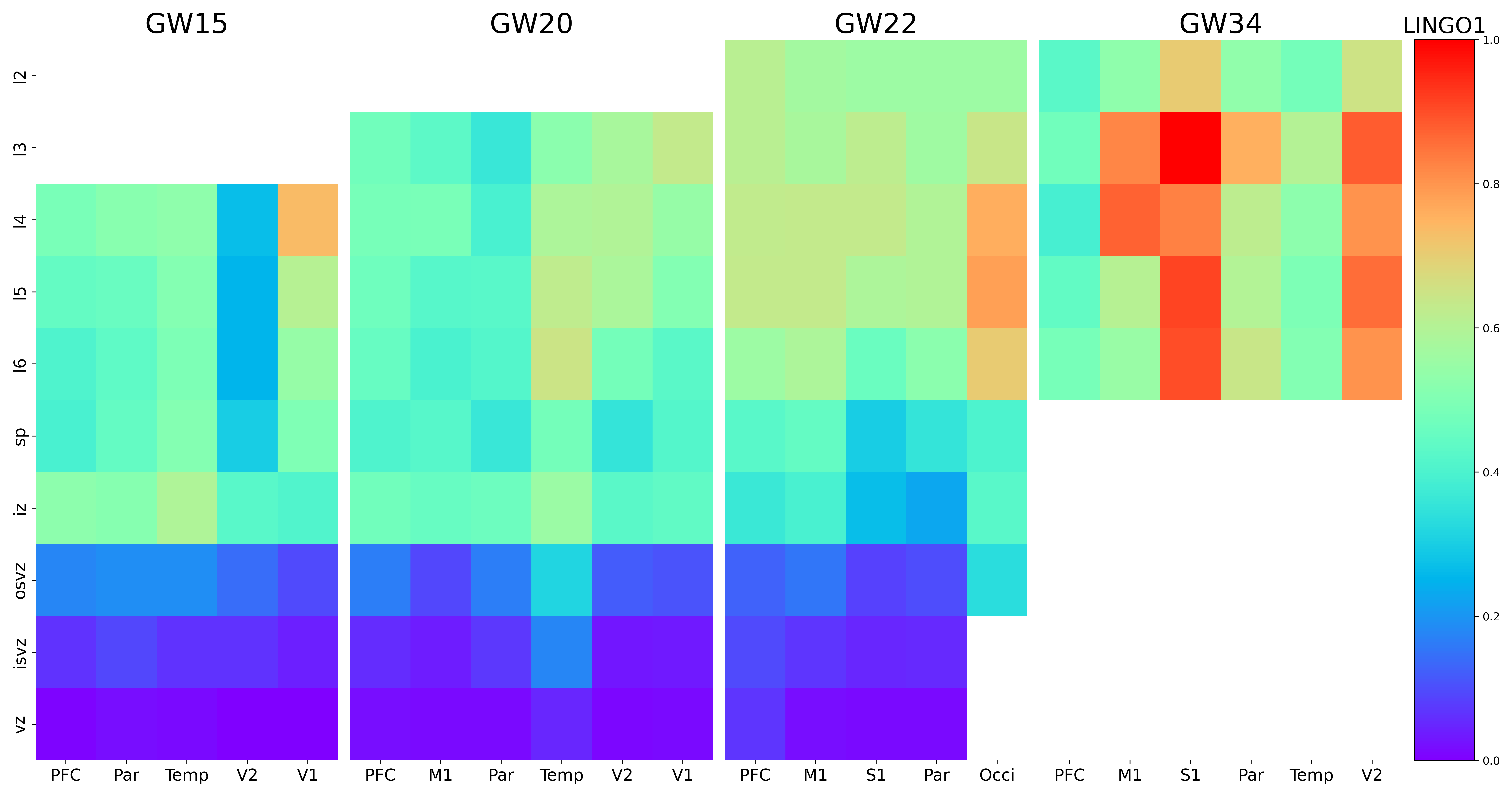

Supplement: Supplementary file 4 — Source Data Fig. 3: Expression pattern heatmap for all 300 genes in the MERFISH. [file 41586_2025_9010_MOESM4_ESM.zip › LINGO1.png]

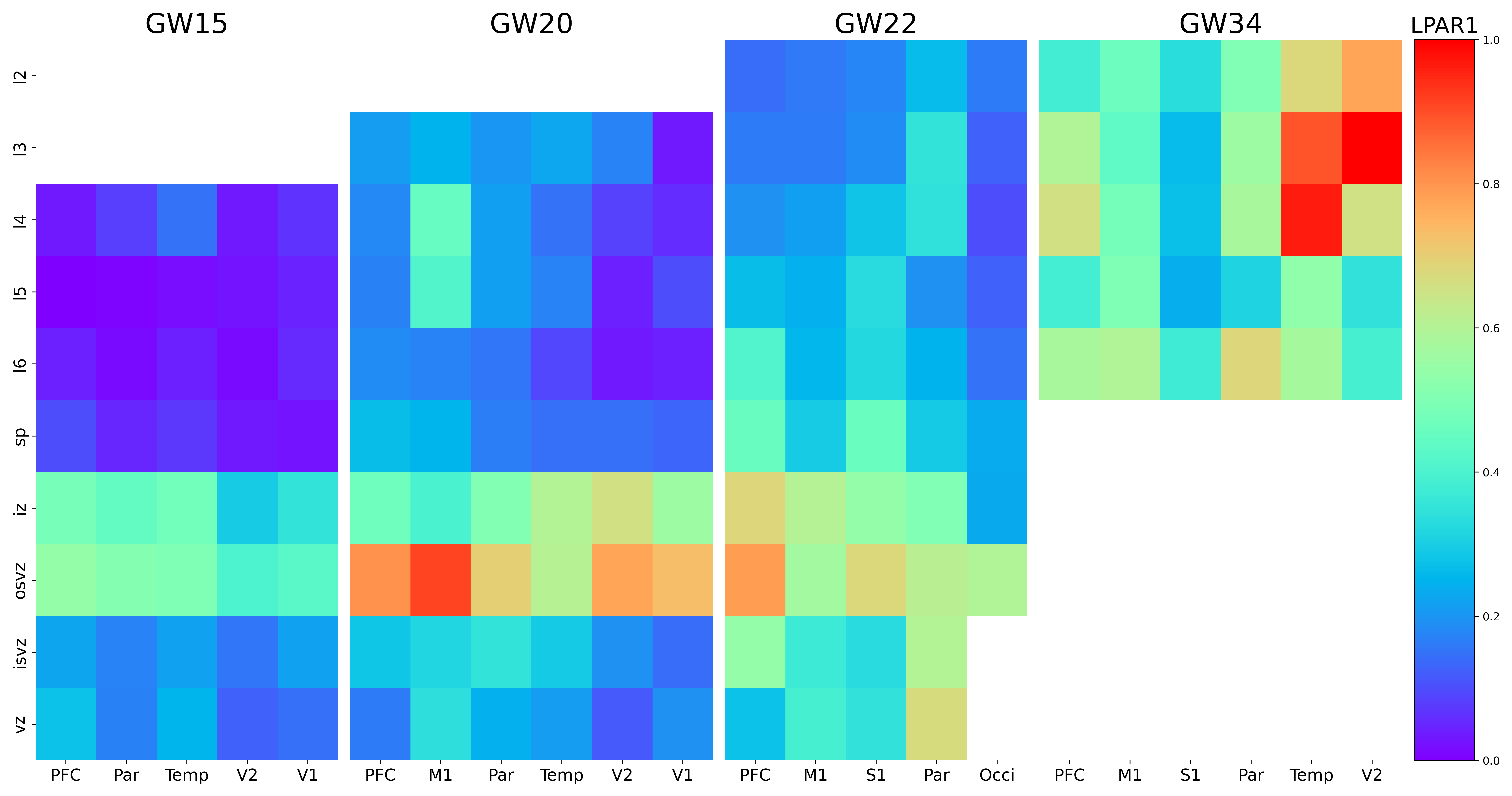

Supplement: Supplementary file 4 — Source Data Fig. 3: Expression pattern heatmap for all 300 genes in the MERFISH. [file 41586_2025_9010_MOESM4_ESM.zip › LPAR1.png]

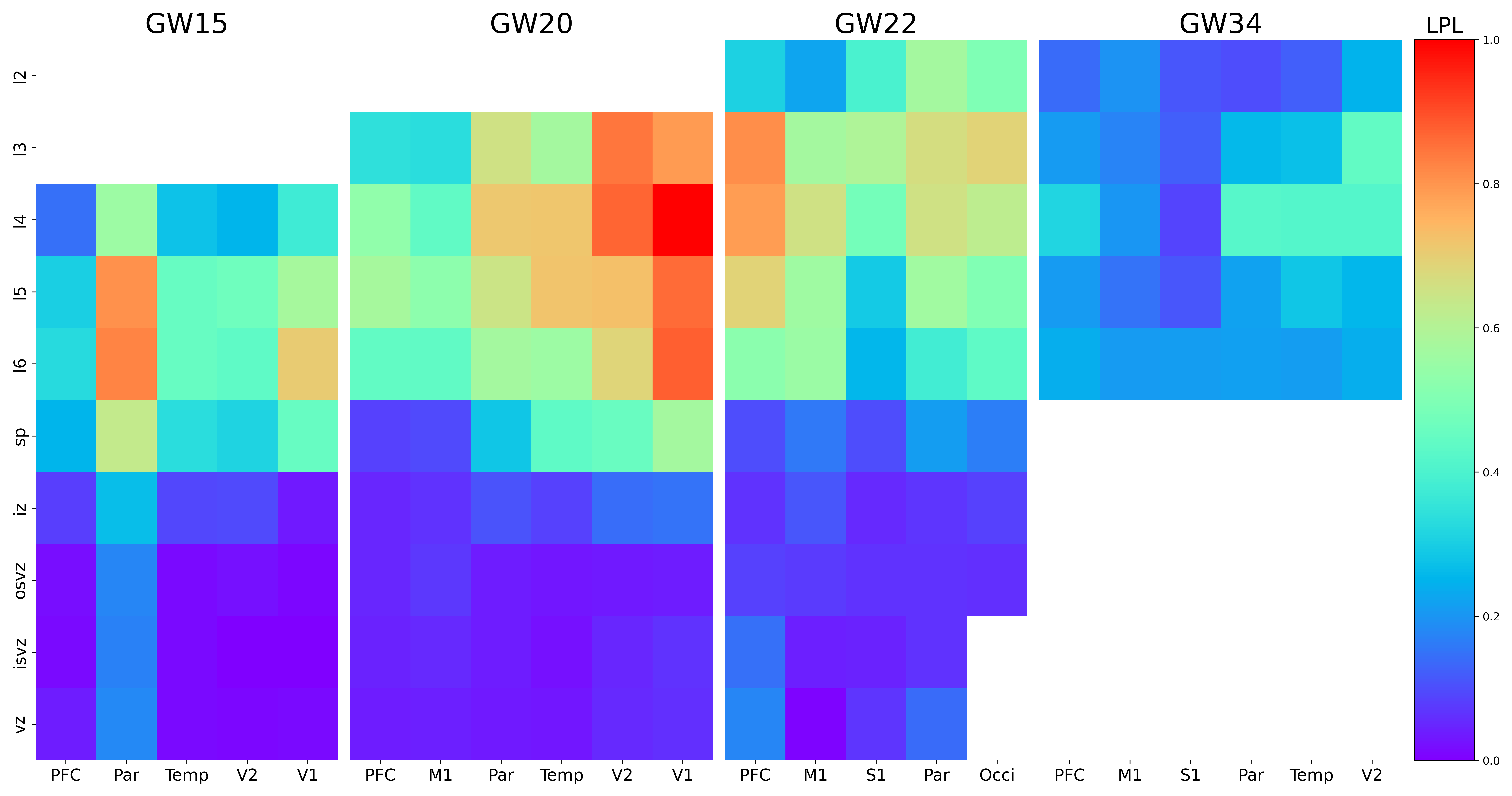

Supplement: Supplementary file 4 — Source Data Fig. 3: Expression pattern heatmap for all 300 genes in the MERFISH. [file 41586_2025_9010_MOESM4_ESM.zip › LPL.png]

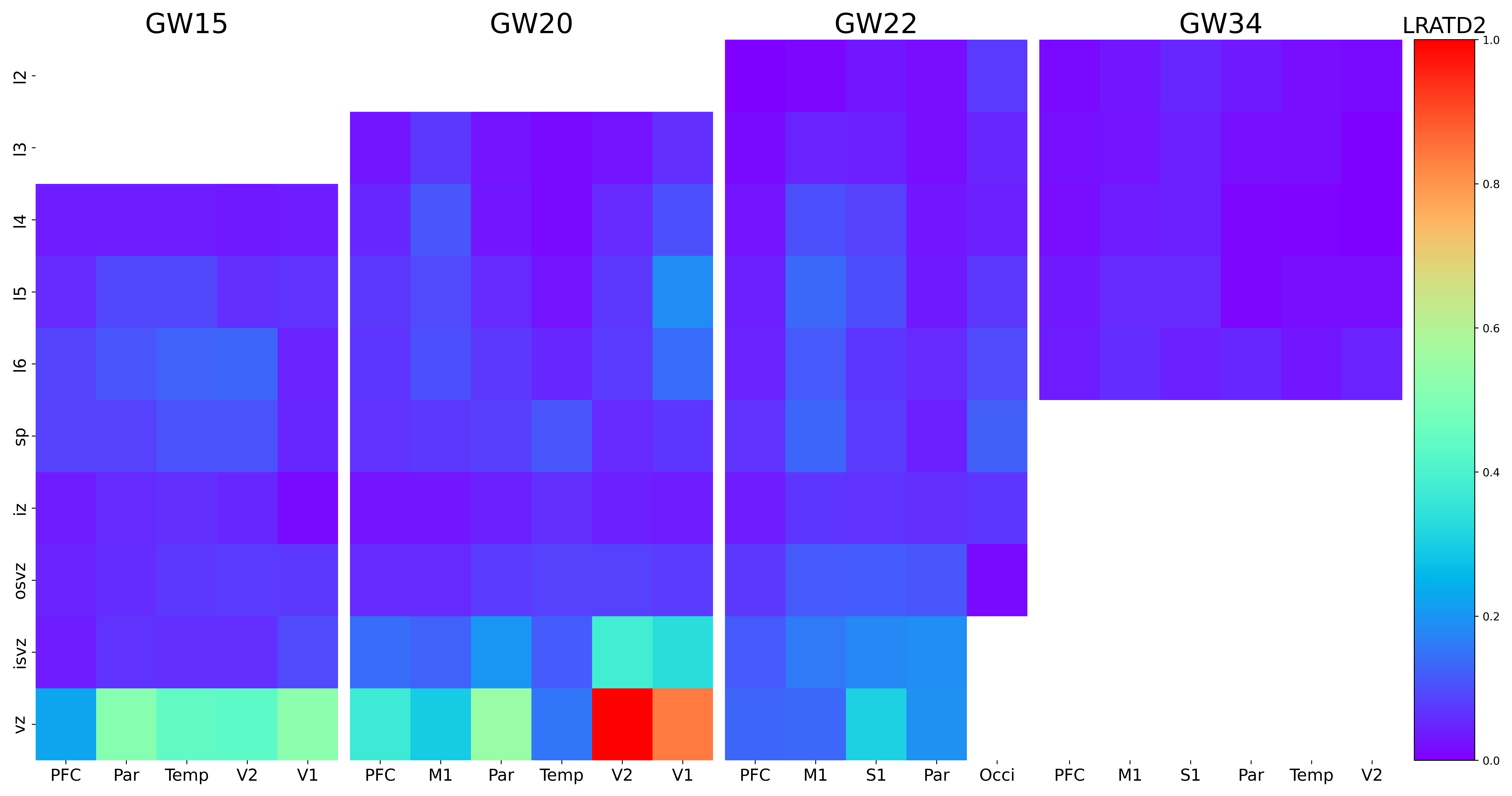

Supplement: Supplementary file 4 — Source Data Fig. 3: Expression pattern heatmap for all 300 genes in the MERFISH. [file 41586_2025_9010_MOESM4_ESM.zip › LRATD2.png]

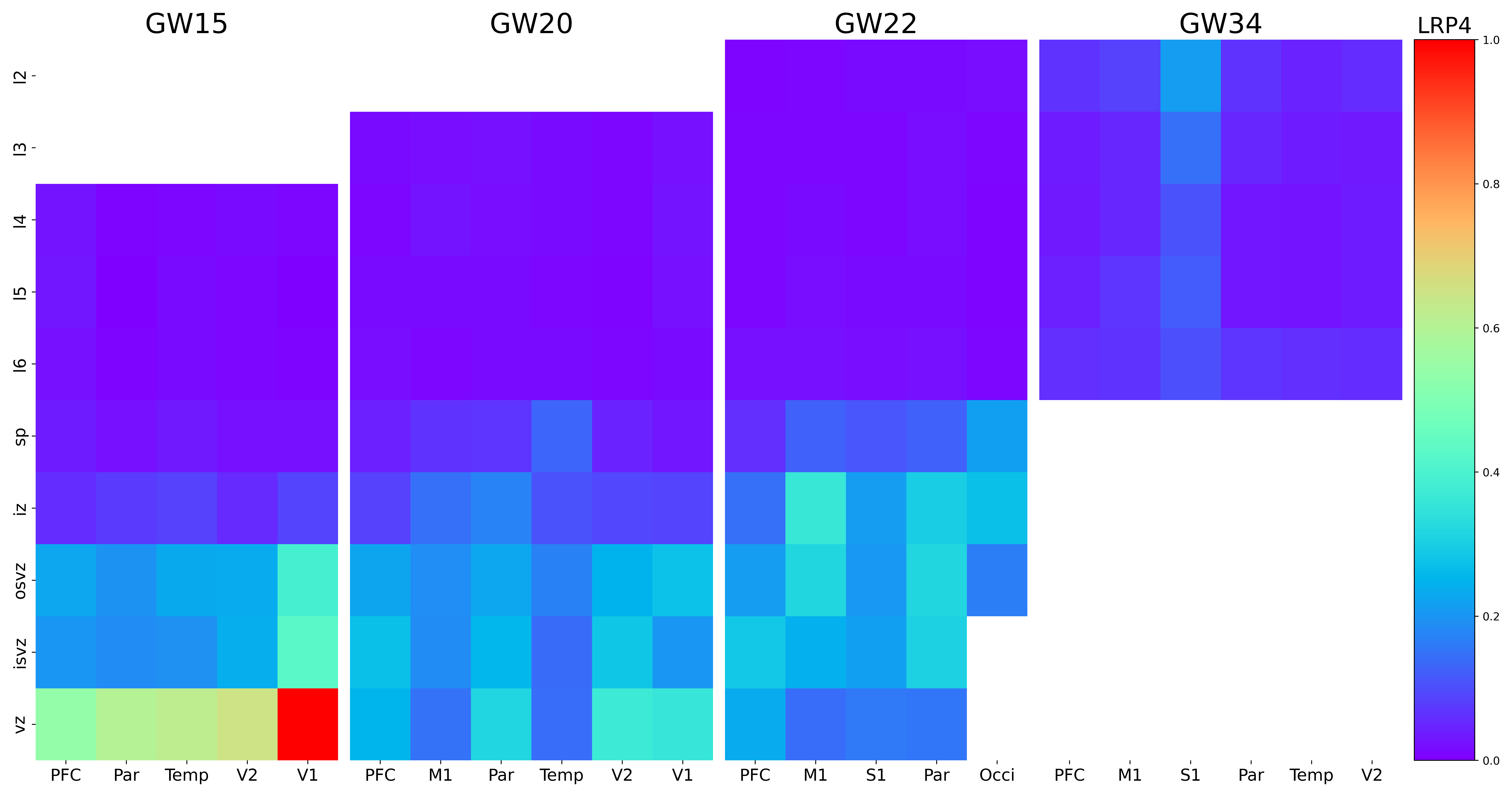

Supplement: Supplementary file 4 — Source Data Fig. 3: Expression pattern heatmap for all 300 genes in the MERFISH. [file 41586_2025_9010_MOESM4_ESM.zip › LRP4.png]

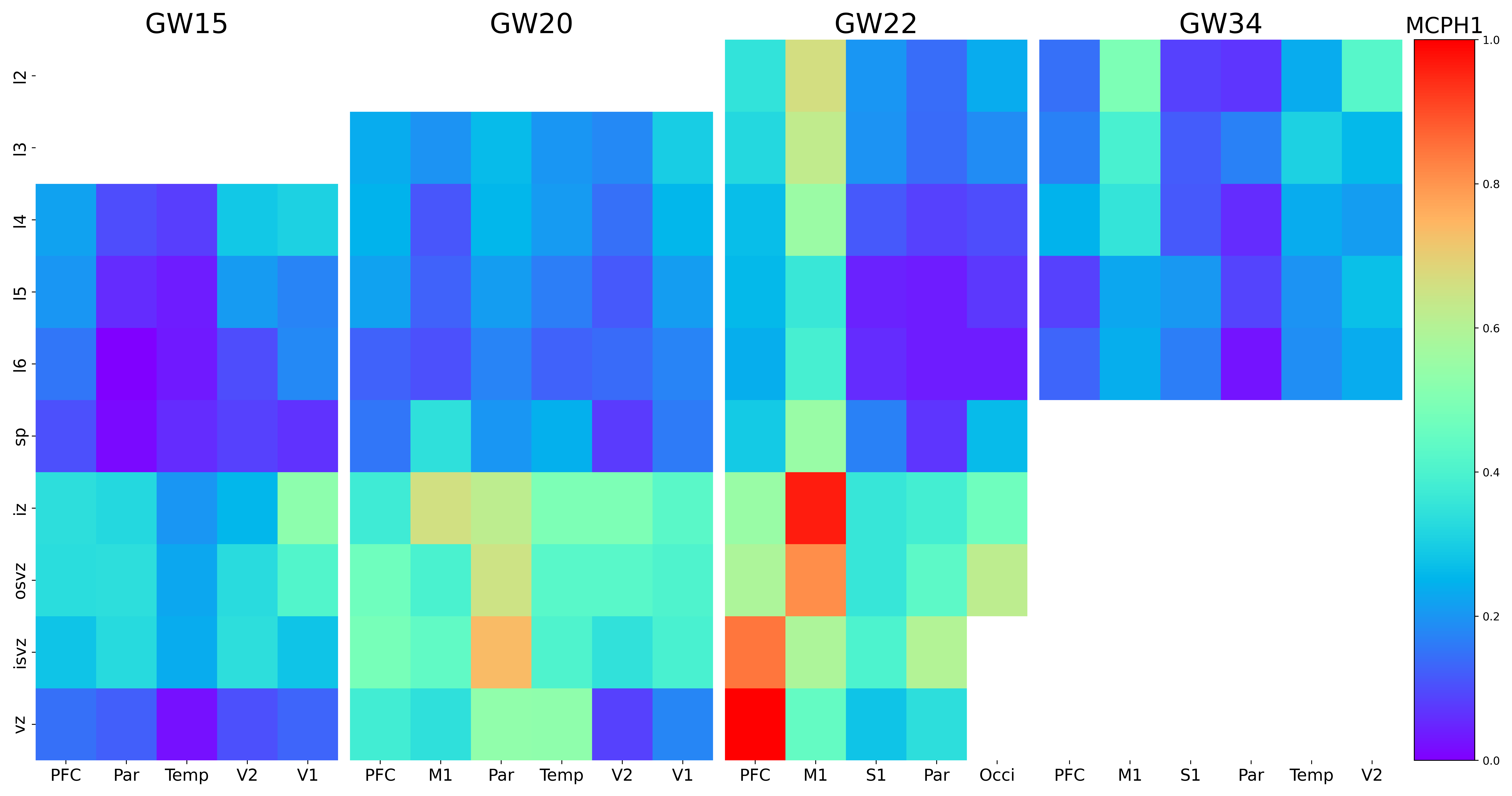

Supplement: Supplementary file 4 — Source Data Fig. 3: Expression pattern heatmap for all 300 genes in the MERFISH. [file 41586_2025_9010_MOESM4_ESM.zip › MCPH1.png]

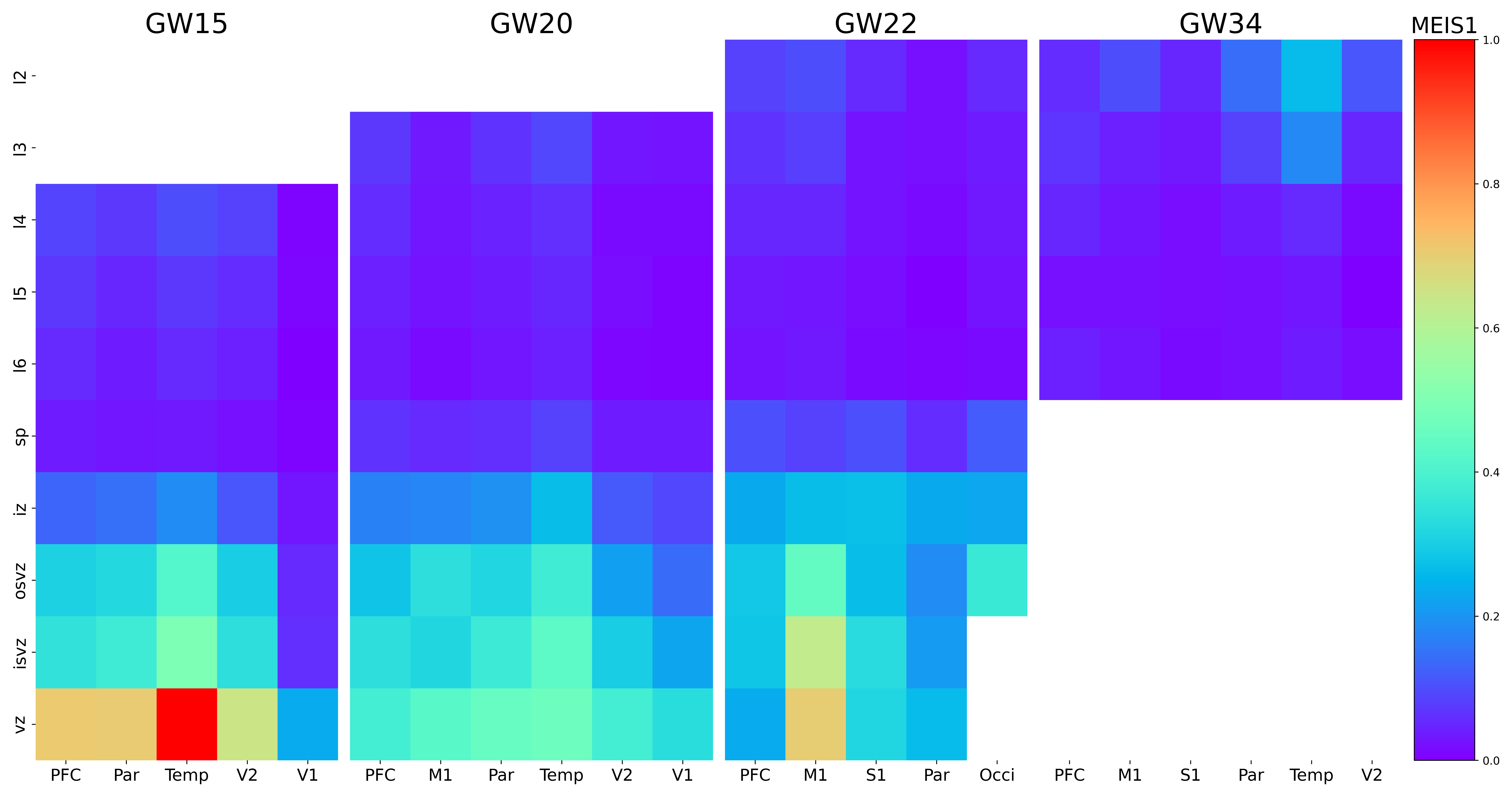

Supplement: Supplementary file 4 — Source Data Fig. 3: Expression pattern heatmap for all 300 genes in the MERFISH. [file 41586_2025_9010_MOESM4_ESM.zip › MEIS1.png]

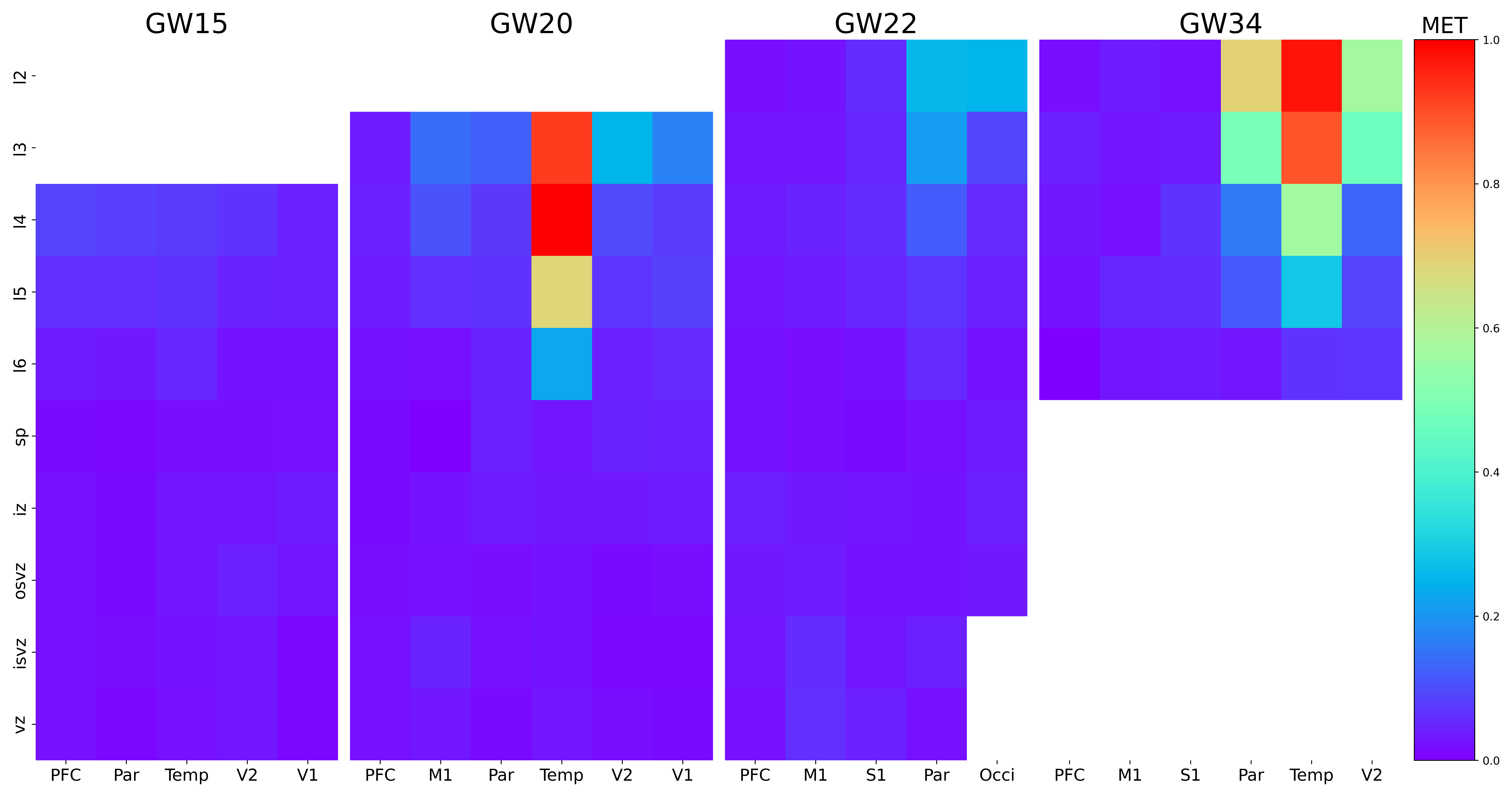

Supplement: Supplementary file 4 — Source Data Fig. 3: Expression pattern heatmap for all 300 genes in the MERFISH. [file 41586_2025_9010_MOESM4_ESM.zip › MET.png]

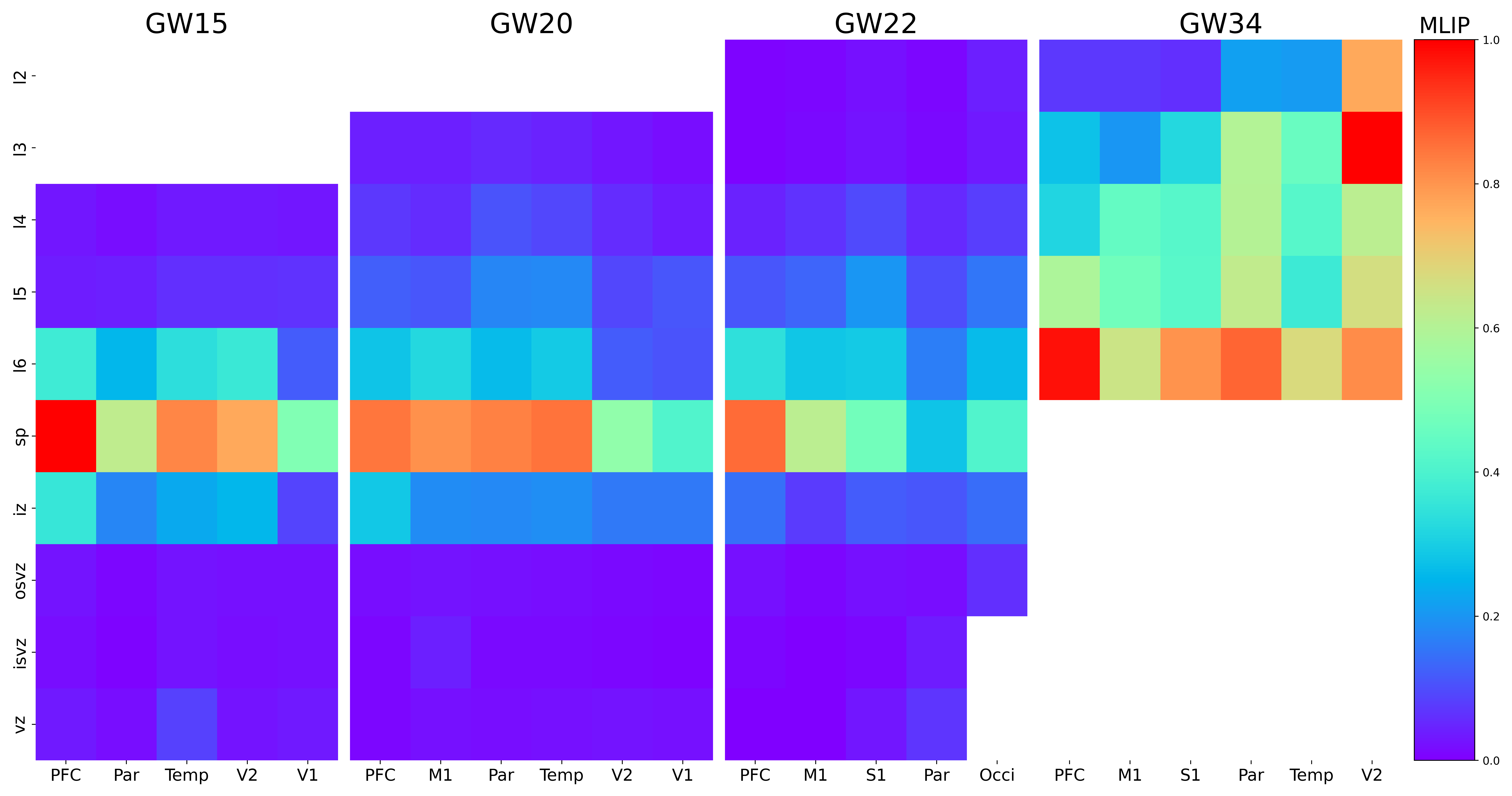

Supplement: Supplementary file 4 — Source Data Fig. 3: Expression pattern heatmap for all 300 genes in the MERFISH. [file 41586_2025_9010_MOESM4_ESM.zip › MLIP.png]

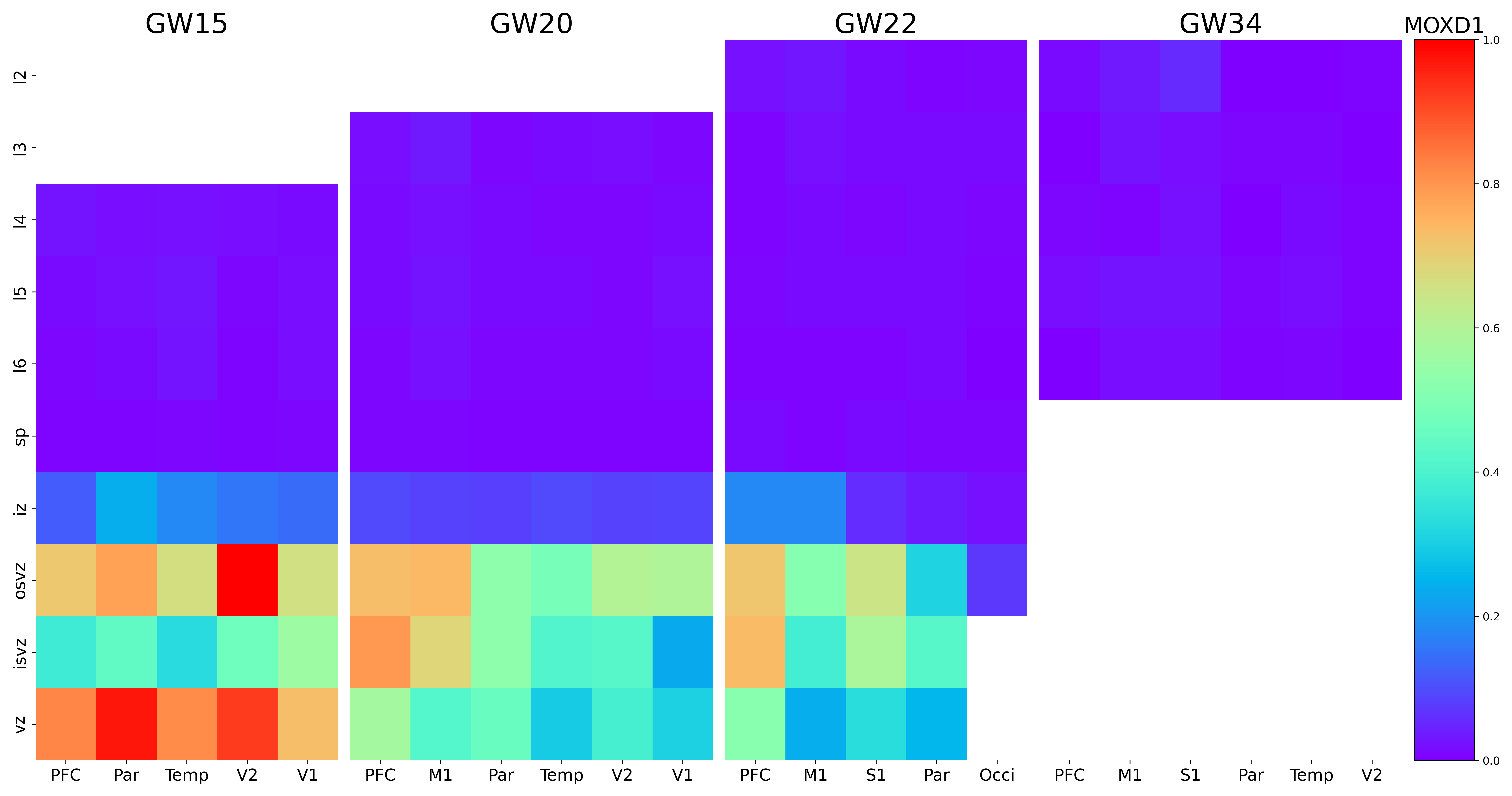

Supplement: Supplementary file 4 — Source Data Fig. 3: Expression pattern heatmap for all 300 genes in the MERFISH. [file 41586_2025_9010_MOESM4_ESM.zip › MOXD1.png]

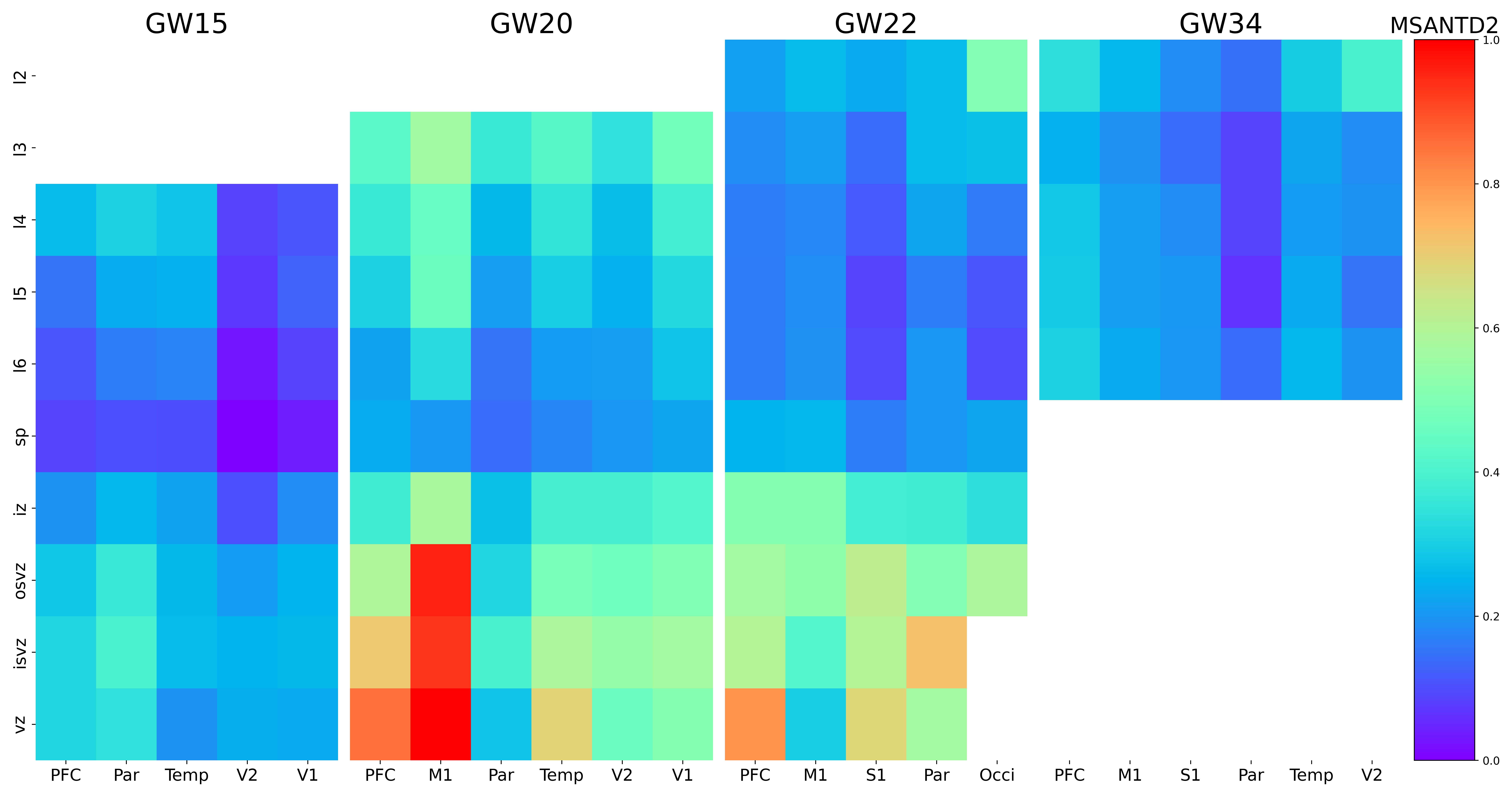

Supplement: Supplementary file 4 — Source Data Fig. 3: Expression pattern heatmap for all 300 genes in the MERFISH. [file 41586_2025_9010_MOESM4_ESM.zip › MSANTD2.png]

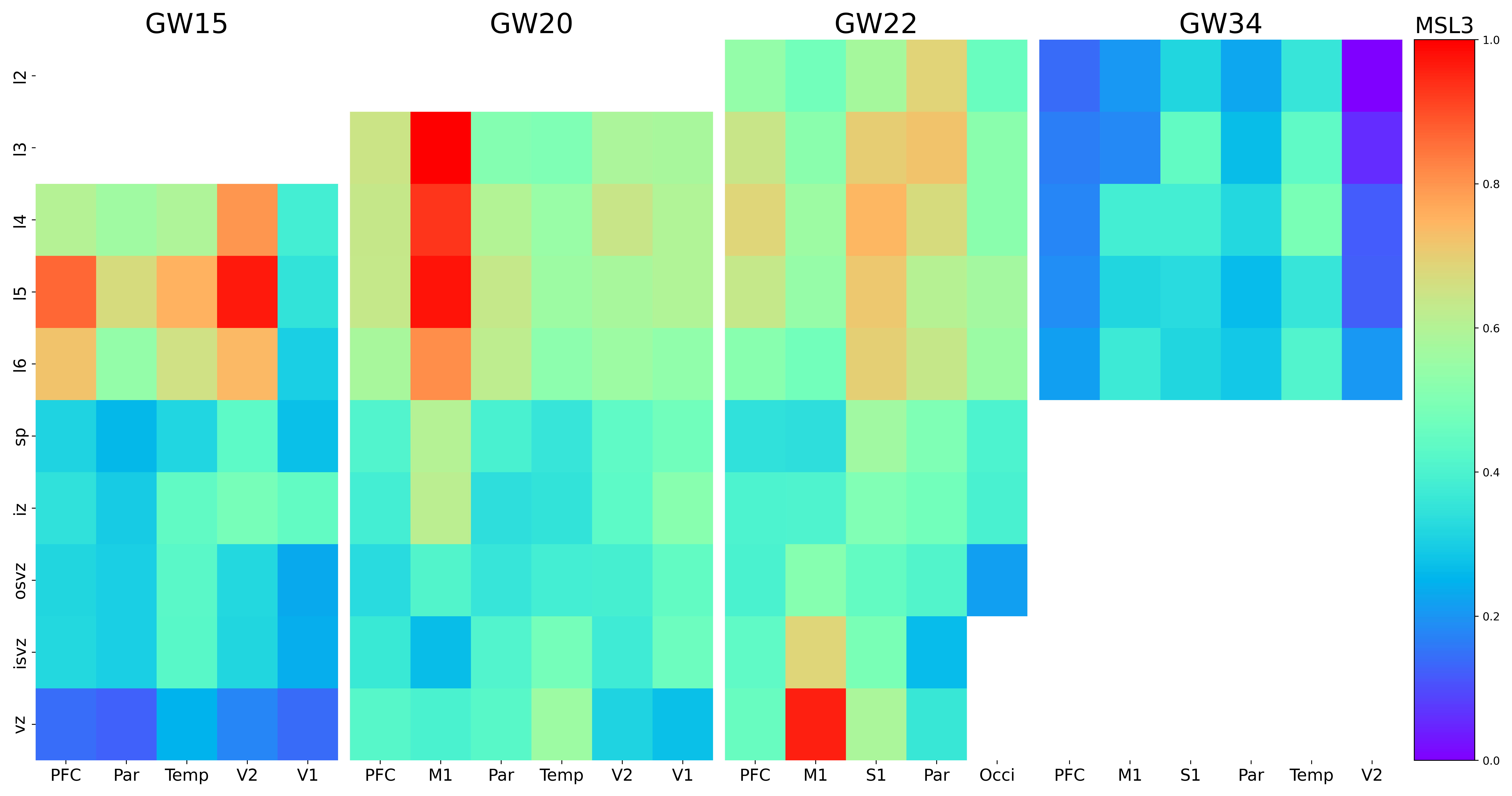

Supplement: Supplementary file 4 — Source Data Fig. 3: Expression pattern heatmap for all 300 genes in the MERFISH. [file 41586_2025_9010_MOESM4_ESM.zip › MSL3.png]

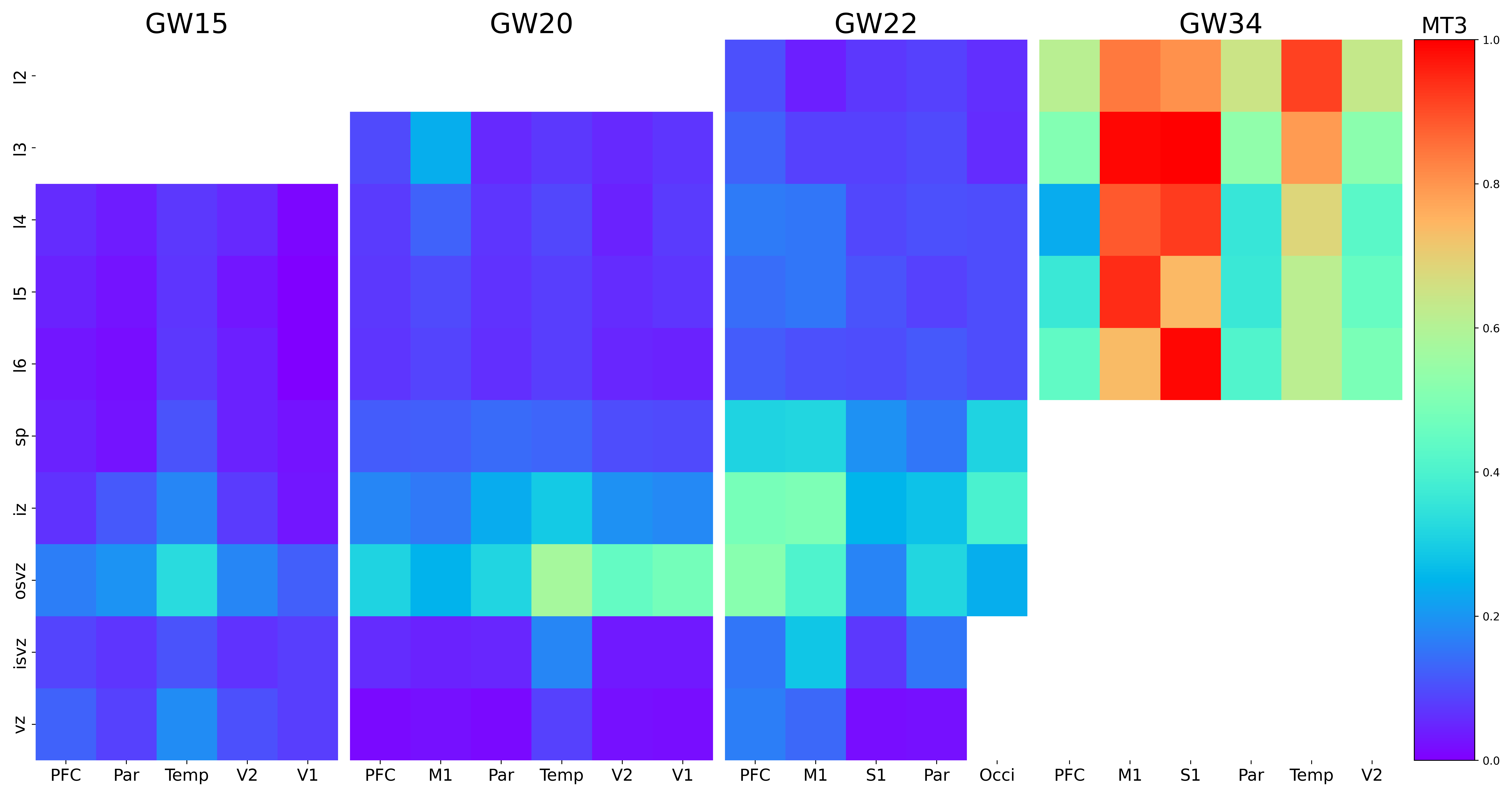

Supplement: Supplementary file 4 — Source Data Fig. 3: Expression pattern heatmap for all 300 genes in the MERFISH. [file 41586_2025_9010_MOESM4_ESM.zip › MT3.png]

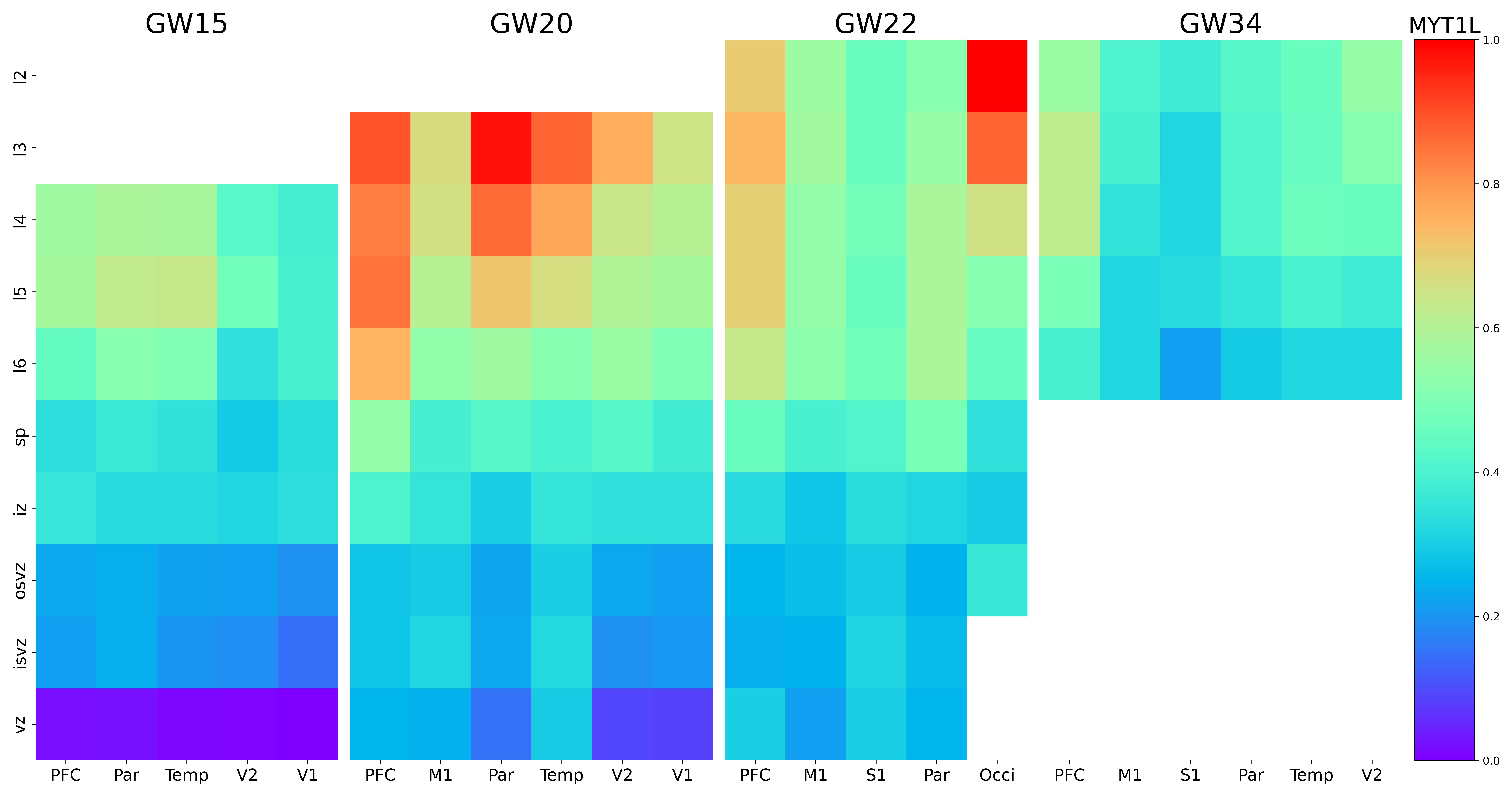

Supplement: Supplementary file 4 — Source Data Fig. 3: Expression pattern heatmap for all 300 genes in the MERFISH. [file 41586_2025_9010_MOESM4_ESM.zip › MYT1L.png]

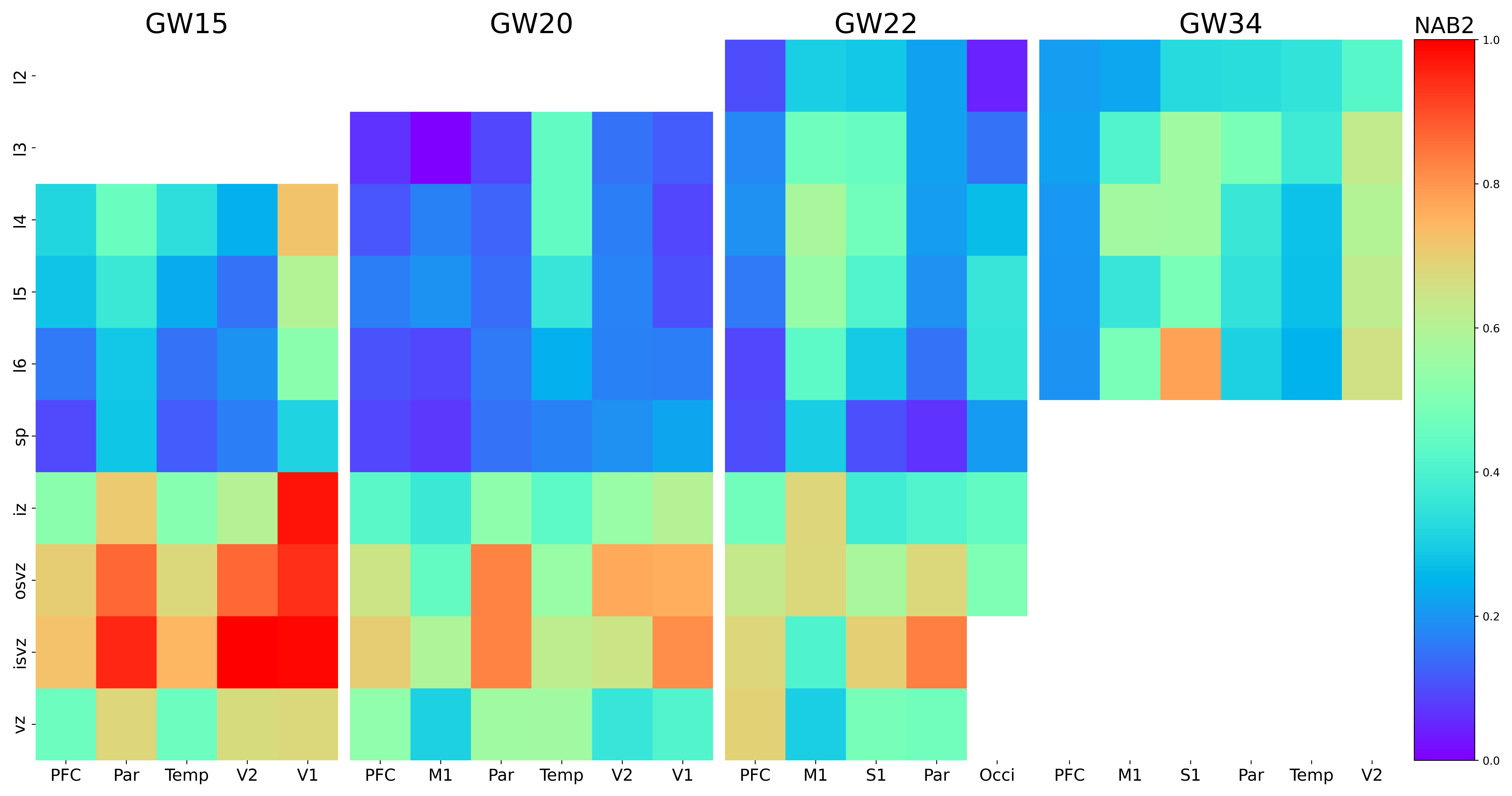

Supplement: Supplementary file 4 — Source Data Fig. 3: Expression pattern heatmap for all 300 genes in the MERFISH. [file 41586_2025_9010_MOESM4_ESM.zip › NAB2.png]

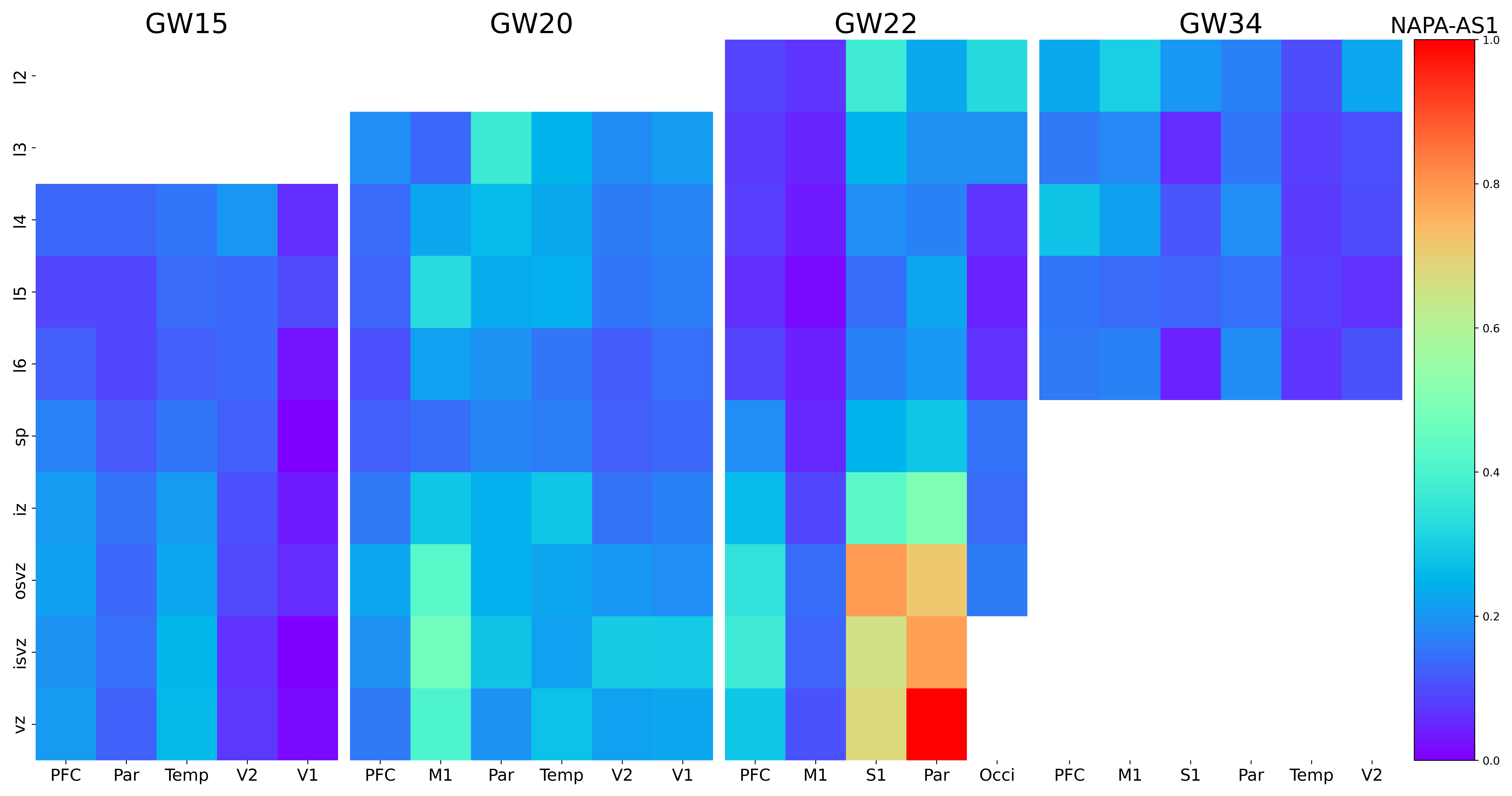

Supplement: Supplementary file 4 — Source Data Fig. 3: Expression pattern heatmap for all 300 genes in the MERFISH. [file 41586_2025_9010_MOESM4_ESM.zip › NAPA-AS1.png]

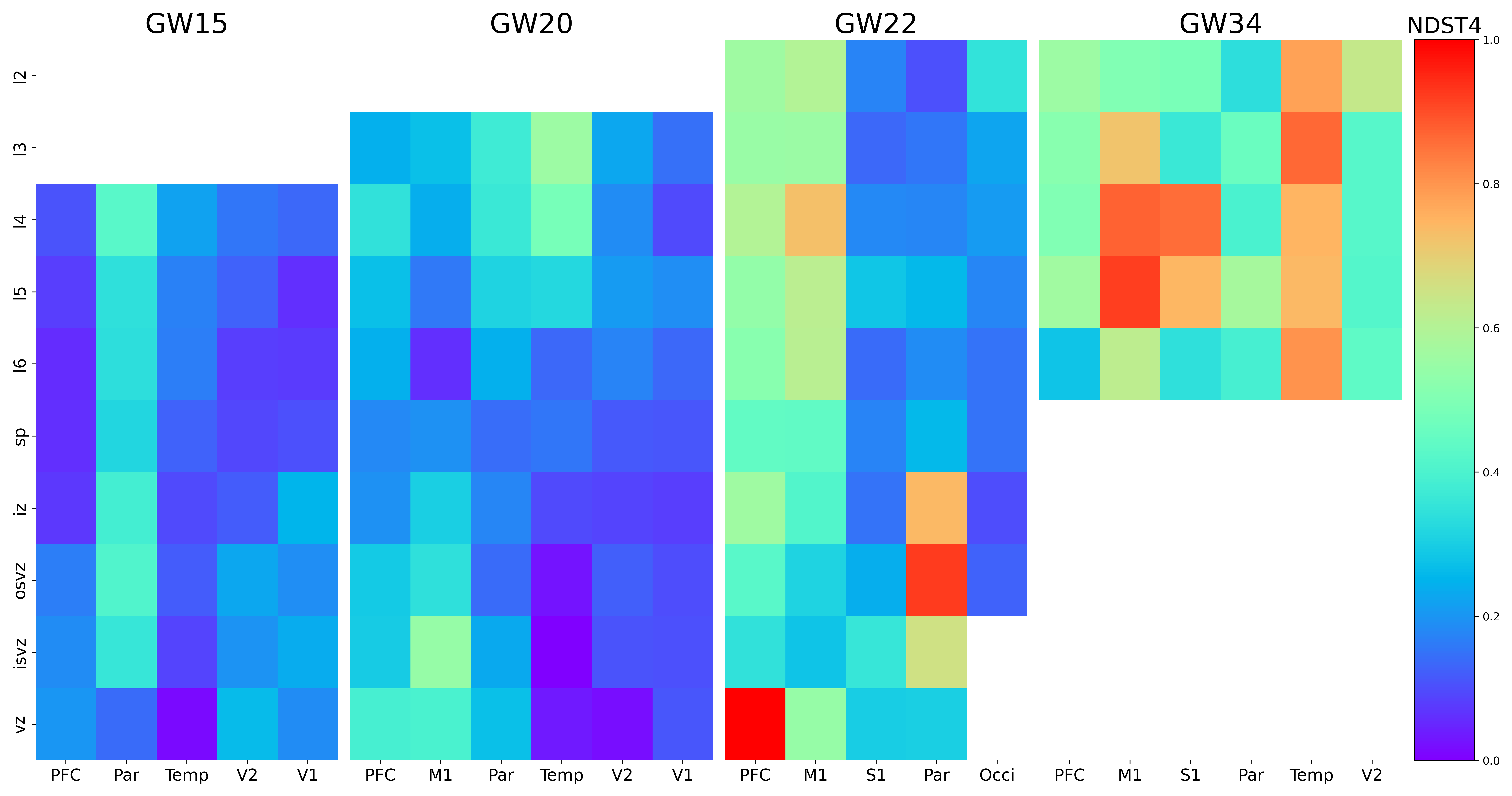

Supplement: Supplementary file 4 — Source Data Fig. 3: Expression pattern heatmap for all 300 genes in the MERFISH. [file 41586_2025_9010_MOESM4_ESM.zip › NDST4.png]

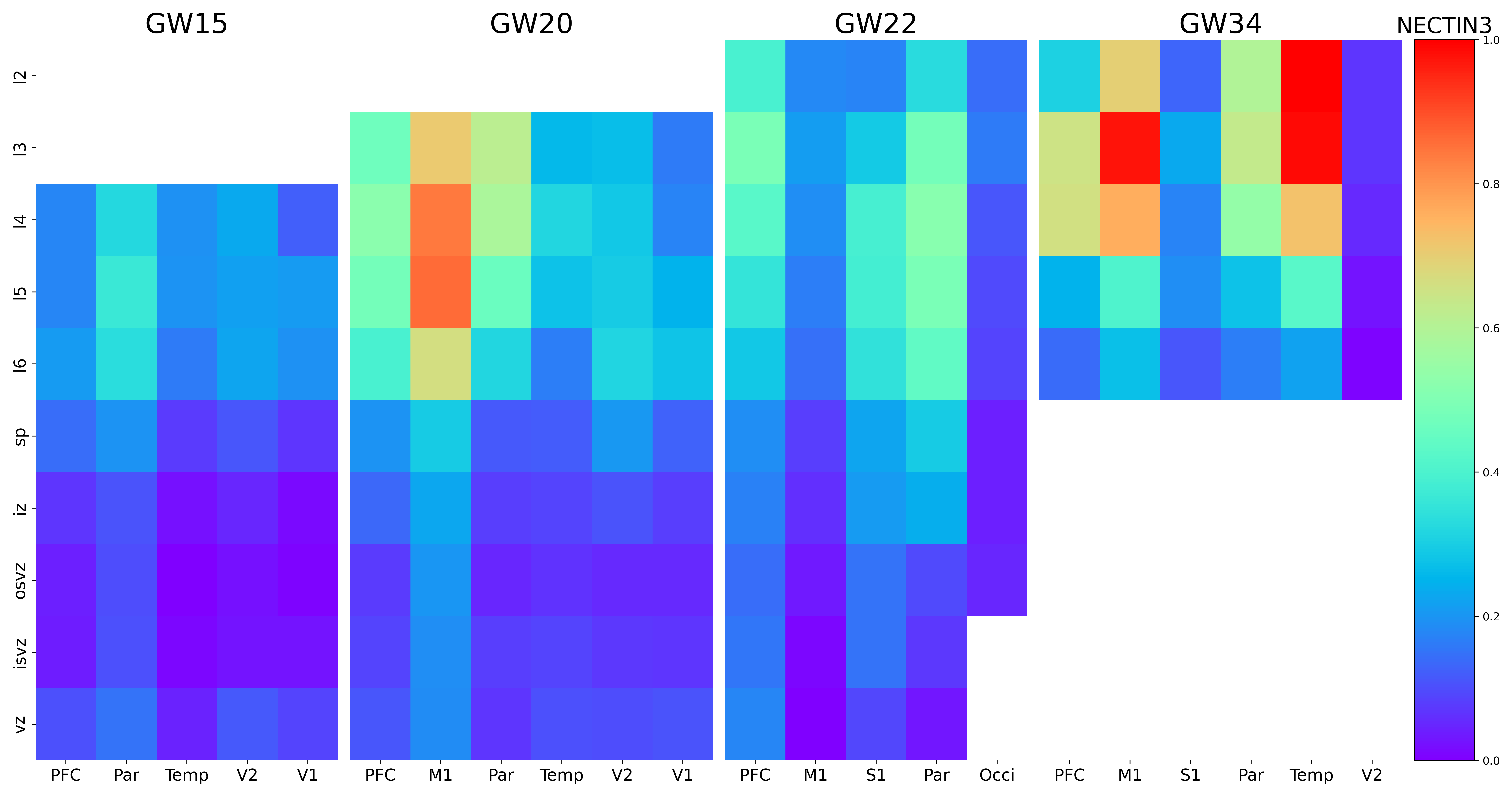

Supplement: Supplementary file 4 — Source Data Fig. 3: Expression pattern heatmap for all 300 genes in the MERFISH. [file 41586_2025_9010_MOESM4_ESM.zip › NECTIN3.png]

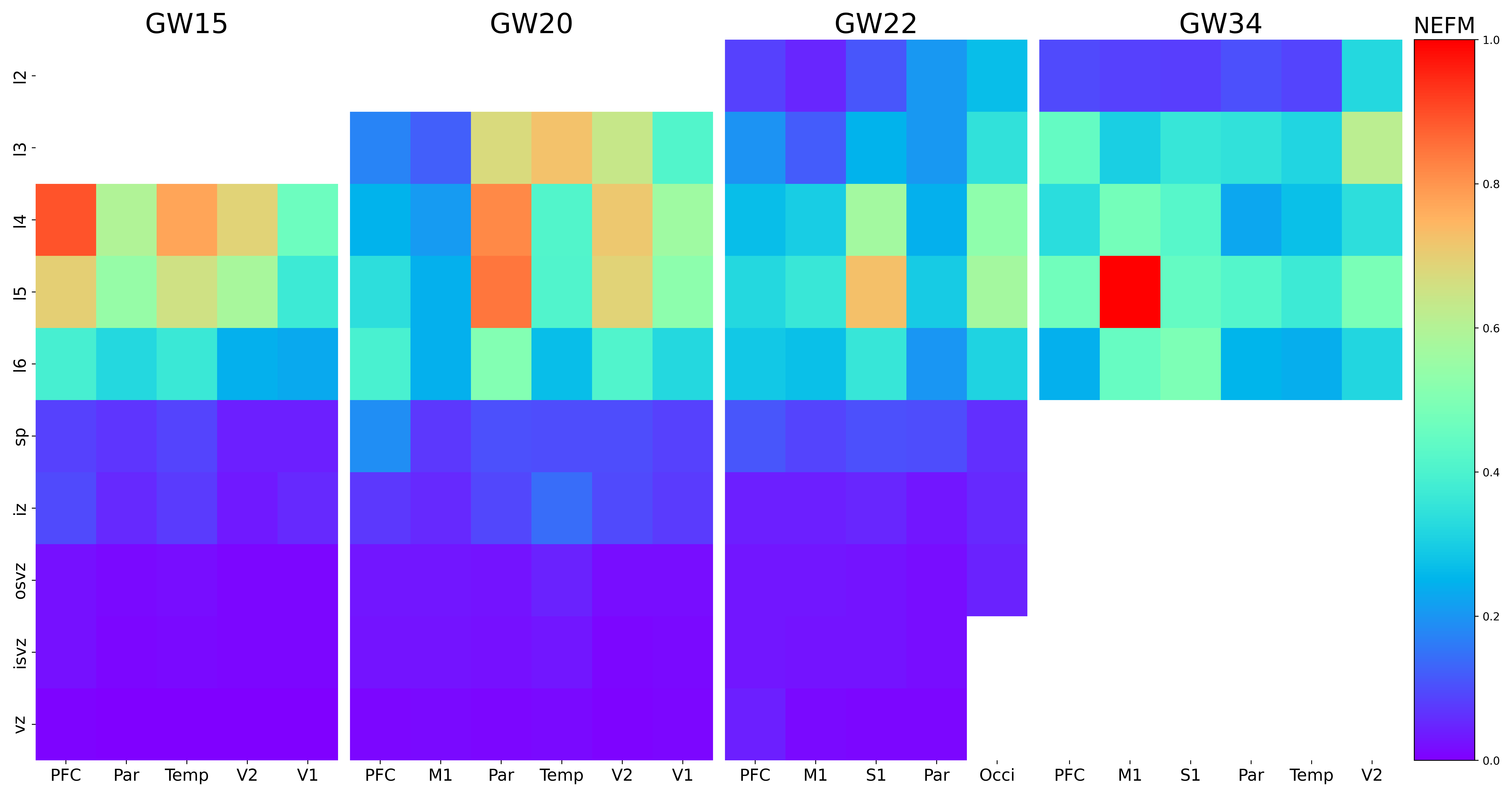

Supplement: Supplementary file 4 — Source Data Fig. 3: Expression pattern heatmap for all 300 genes in the MERFISH. [file 41586_2025_9010_MOESM4_ESM.zip › NEFM.png]

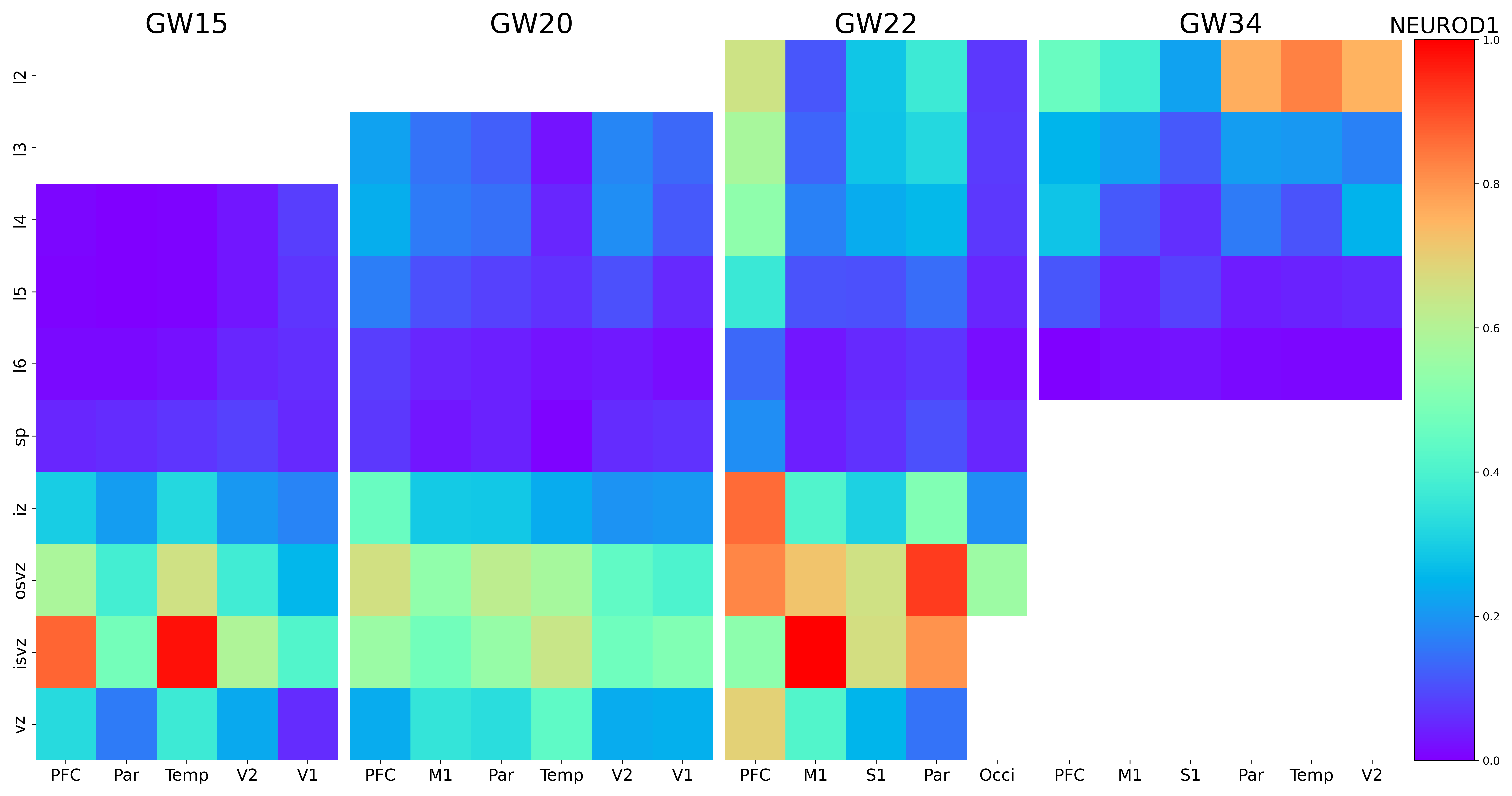

Supplement: Supplementary file 4 — Source Data Fig. 3: Expression pattern heatmap for all 300 genes in the MERFISH. [file 41586_2025_9010_MOESM4_ESM.zip › NEUROD1.png]

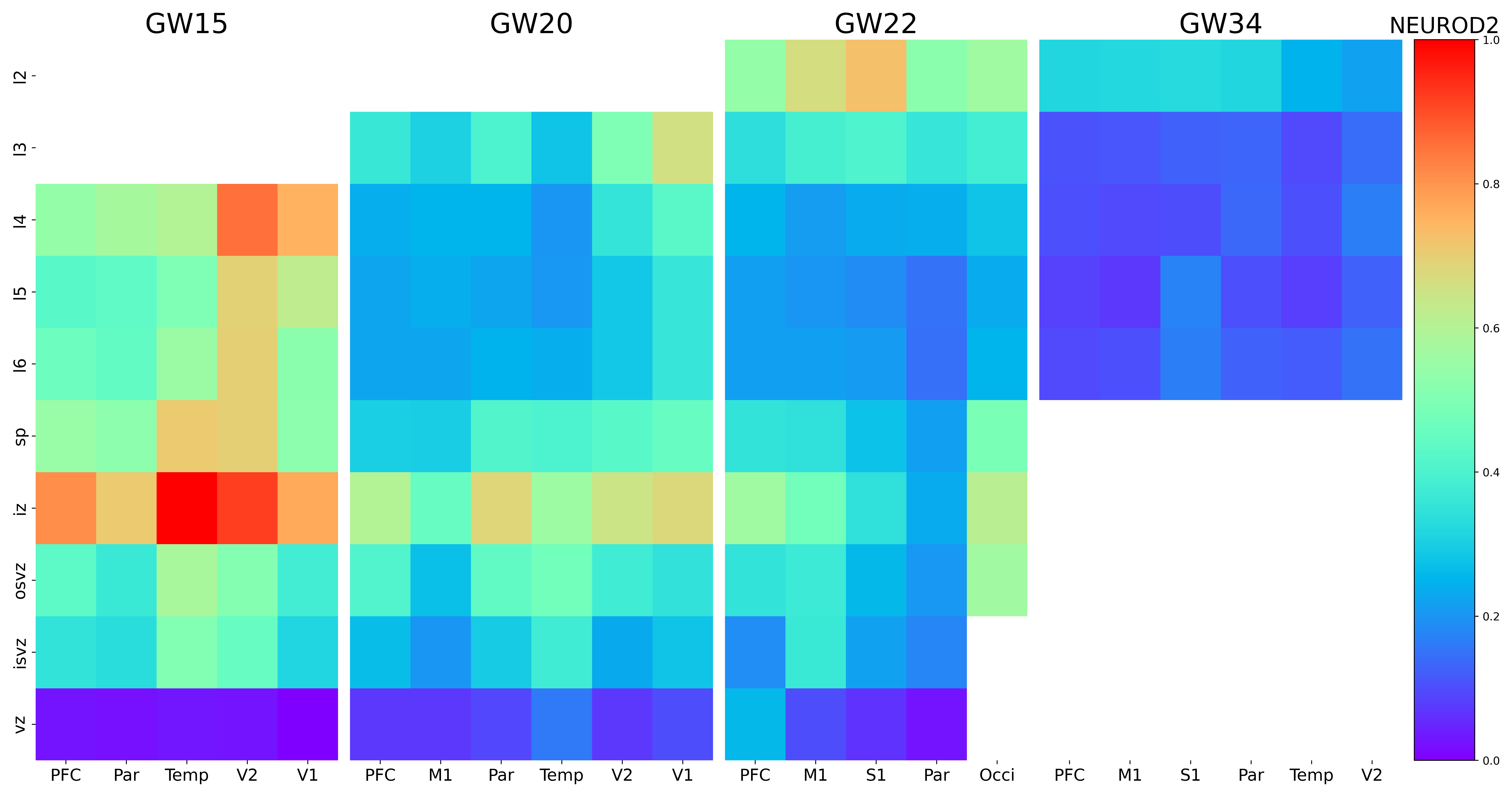

Supplement: Supplementary file 4 — Source Data Fig. 3: Expression pattern heatmap for all 300 genes in the MERFISH. [file 41586_2025_9010_MOESM4_ESM.zip › NEUROD2.png]

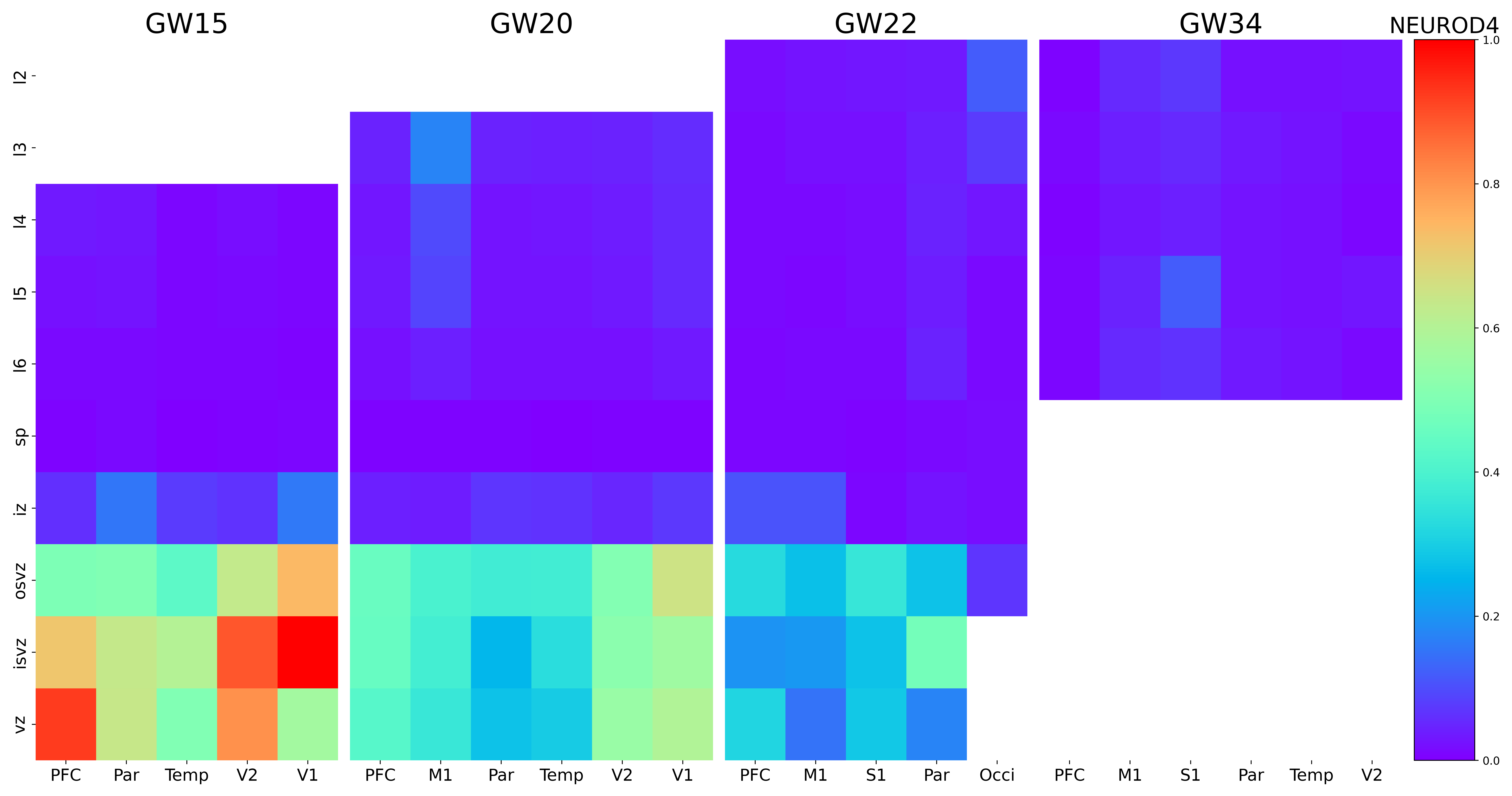

Supplement: Supplementary file 4 — Source Data Fig. 3: Expression pattern heatmap for all 300 genes in the MERFISH. [file 41586_2025_9010_MOESM4_ESM.zip › NEUROD4.png]

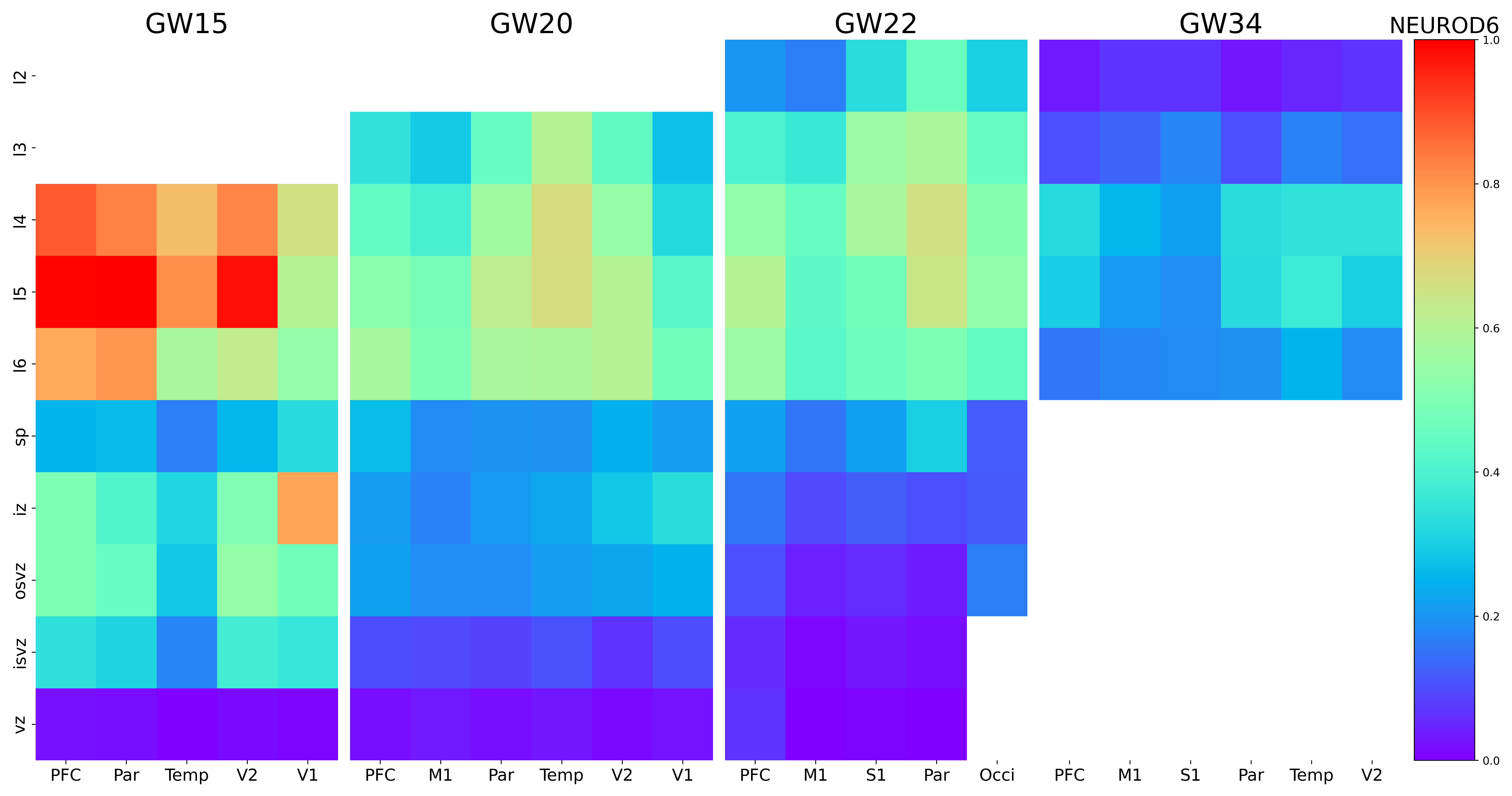

Supplement: Supplementary file 4 — Source Data Fig. 3: Expression pattern heatmap for all 300 genes in the MERFISH. [file 41586_2025_9010_MOESM4_ESM.zip › NEUROD6.png]

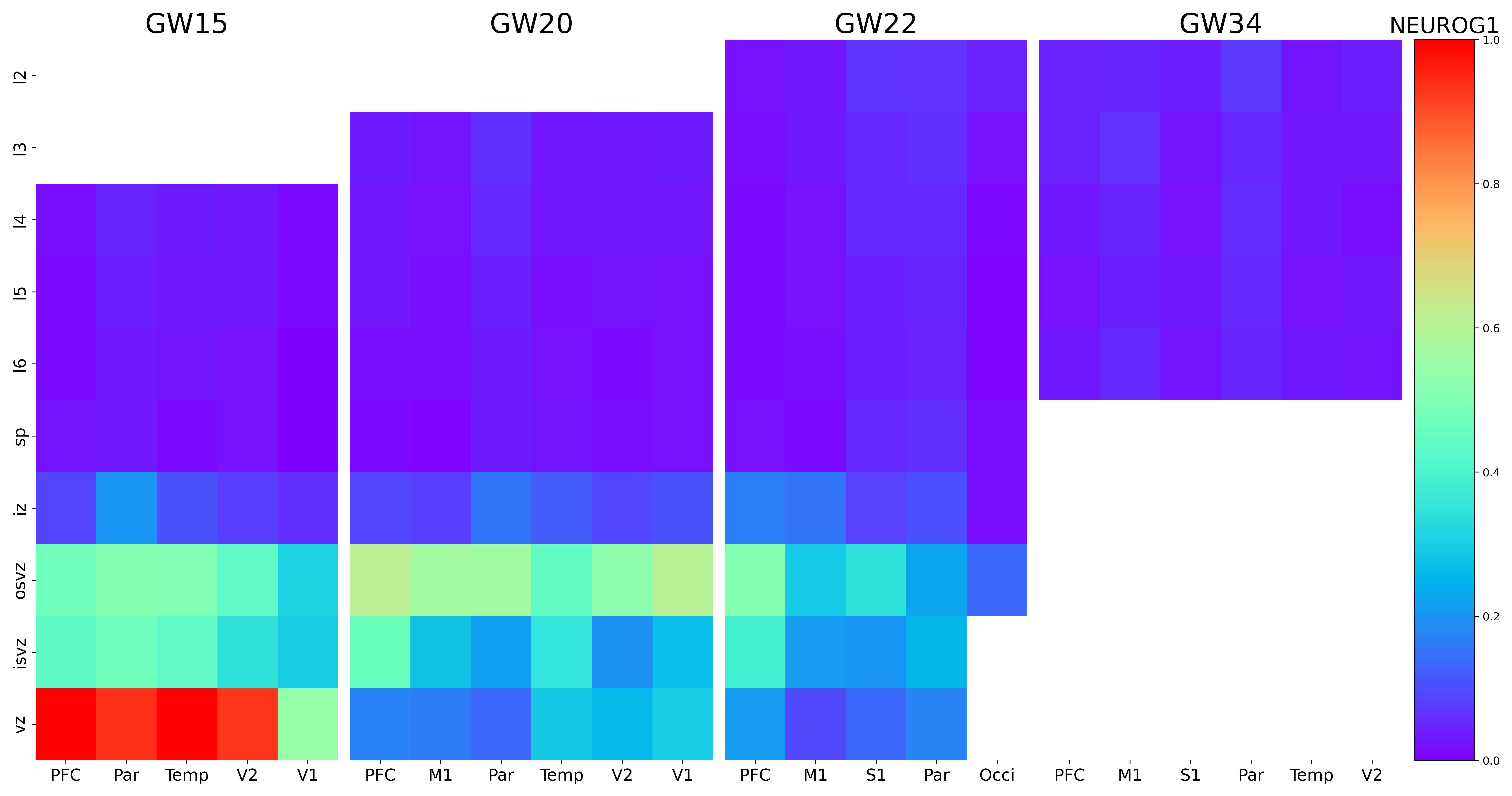

Supplement: Supplementary file 4 — Source Data Fig. 3: Expression pattern heatmap for all 300 genes in the MERFISH. [file 41586_2025_9010_MOESM4_ESM.zip › NEUROG1.png]

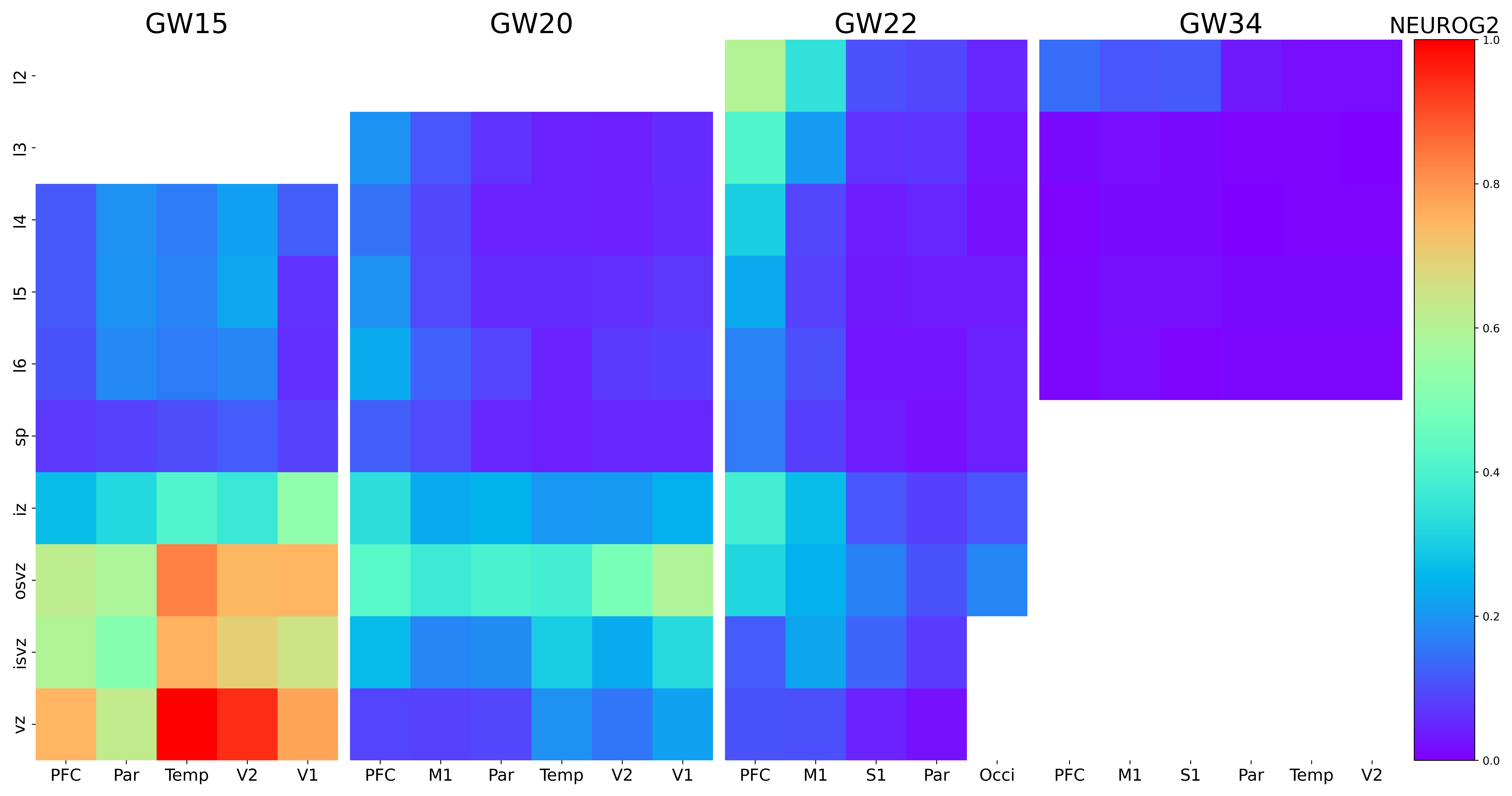

Supplement: Supplementary file 4 — Source Data Fig. 3: Expression pattern heatmap for all 300 genes in the MERFISH. [file 41586_2025_9010_MOESM4_ESM.zip › NEUROG2.png]

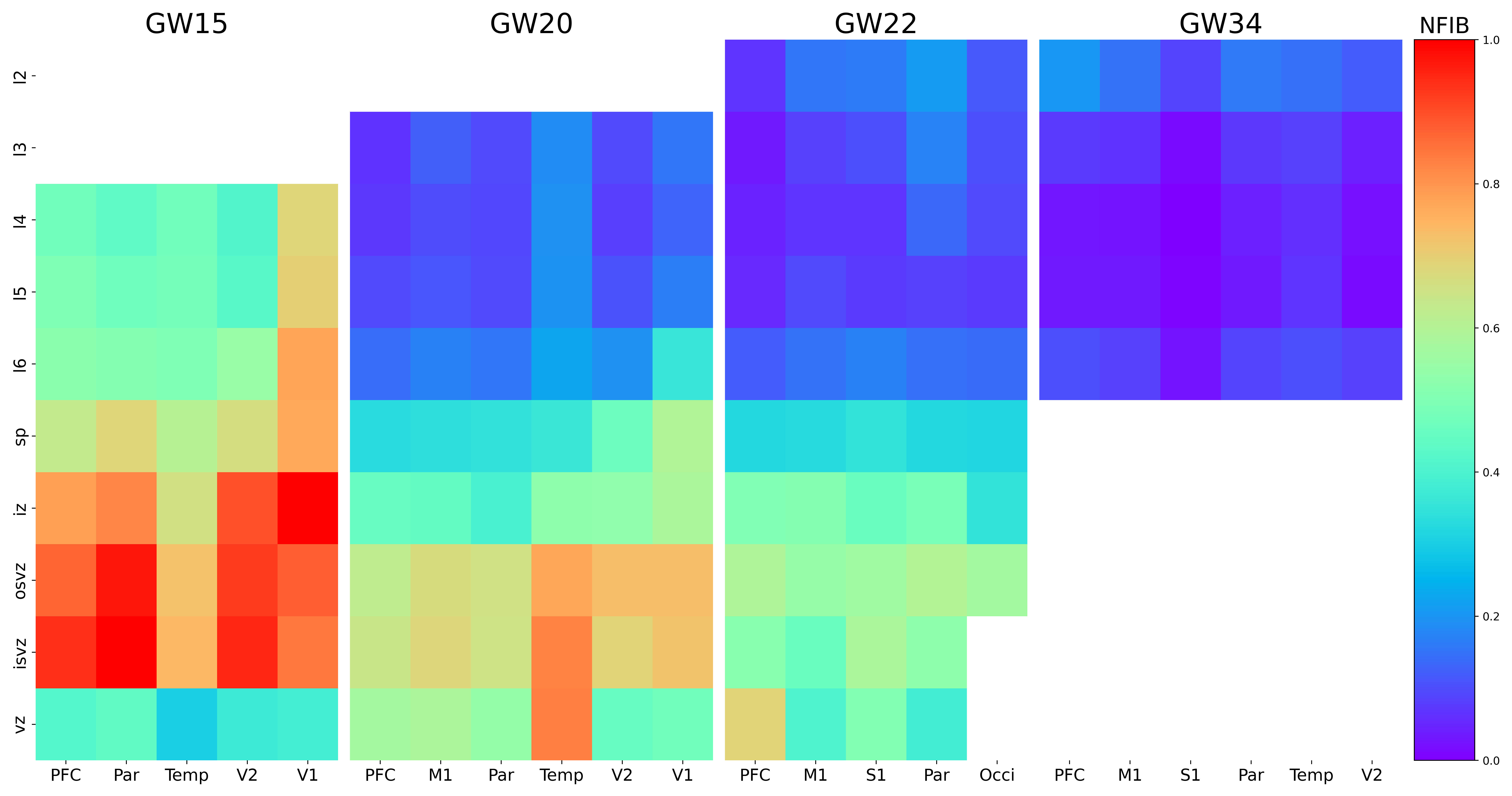

Supplement: Supplementary file 4 — Source Data Fig. 3: Expression pattern heatmap for all 300 genes in the MERFISH. [file 41586_2025_9010_MOESM4_ESM.zip › NFIB.png]

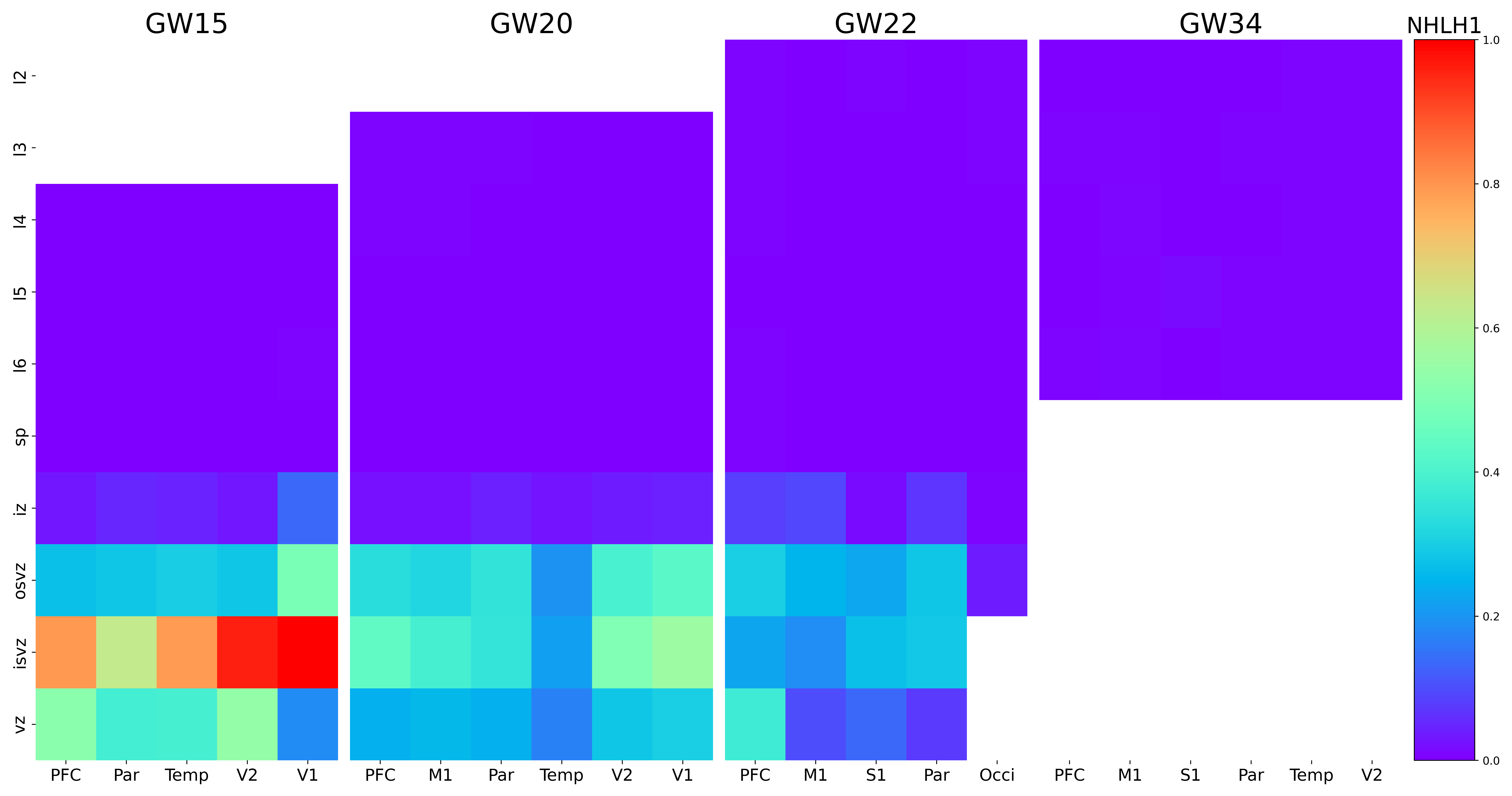

Supplement: Supplementary file 4 — Source Data Fig. 3: Expression pattern heatmap for all 300 genes in the MERFISH. [file 41586_2025_9010_MOESM4_ESM.zip › NHLH1.png]

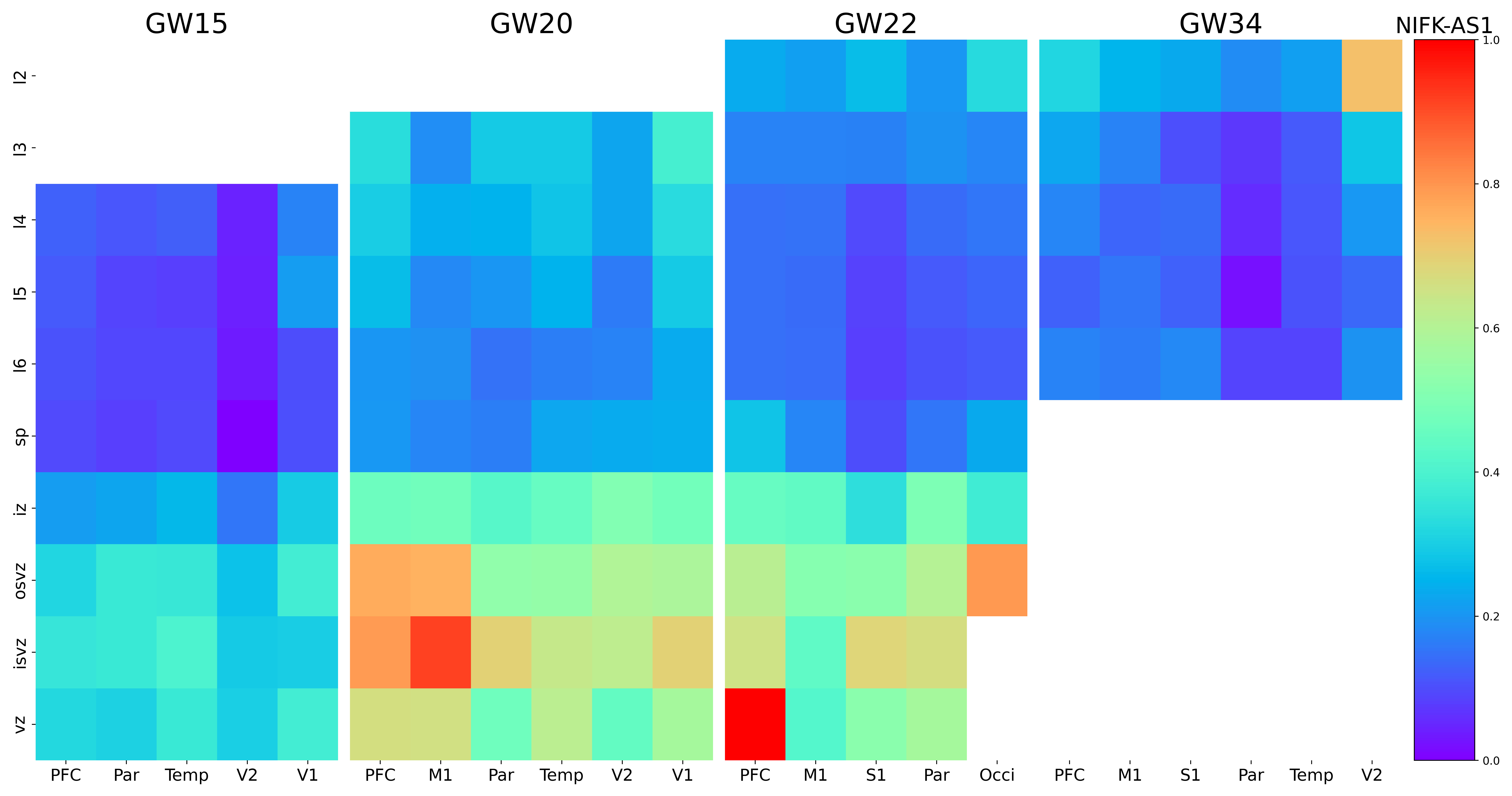

Supplement: Supplementary file 4 — Source Data Fig. 3: Expression pattern heatmap for all 300 genes in the MERFISH. [file 41586_2025_9010_MOESM4_ESM.zip › NIFK-AS1.png]

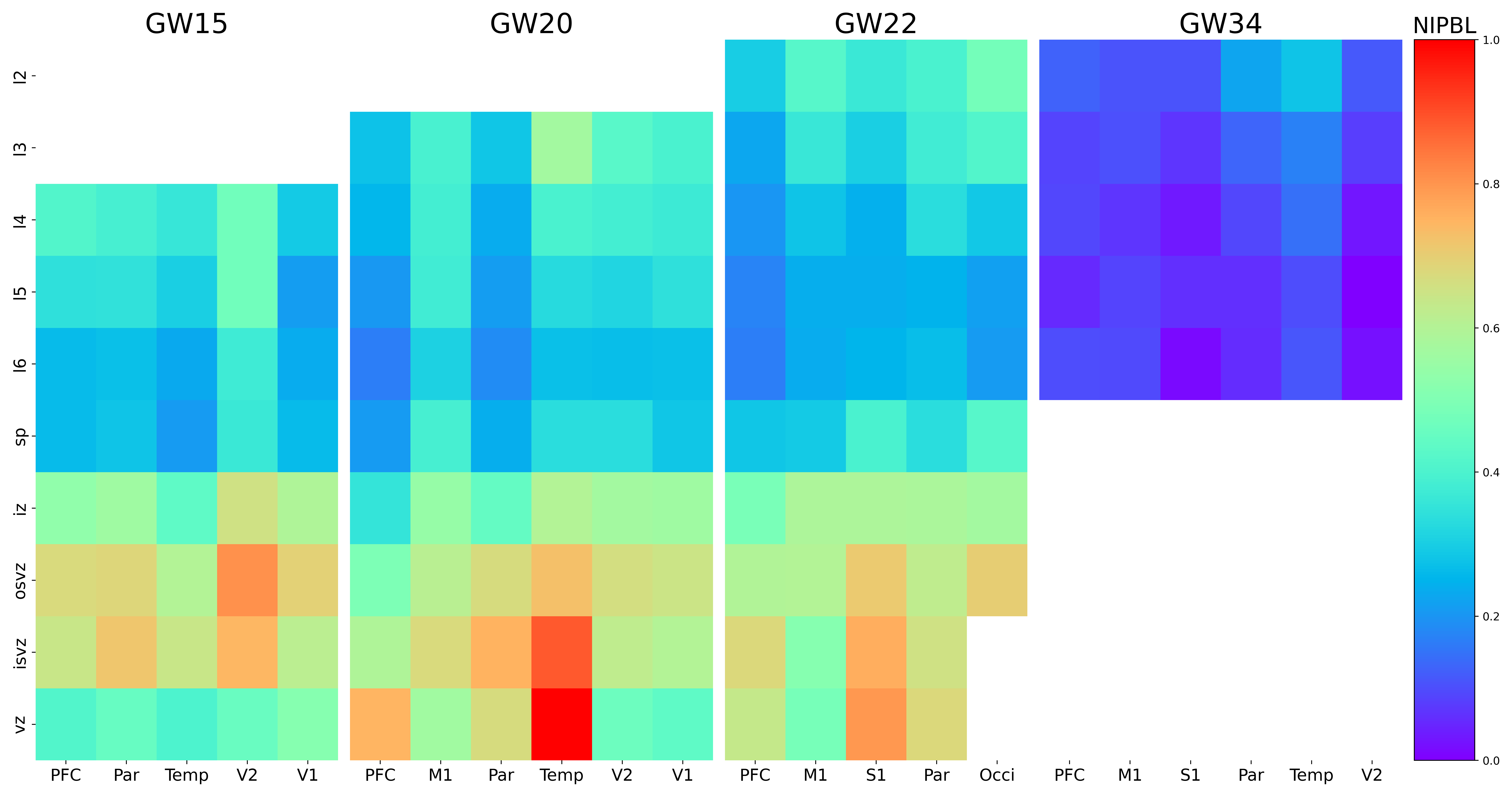

Supplement: Supplementary file 4 — Source Data Fig. 3: Expression pattern heatmap for all 300 genes in the MERFISH. [file 41586_2025_9010_MOESM4_ESM.zip › NIPBL.png]

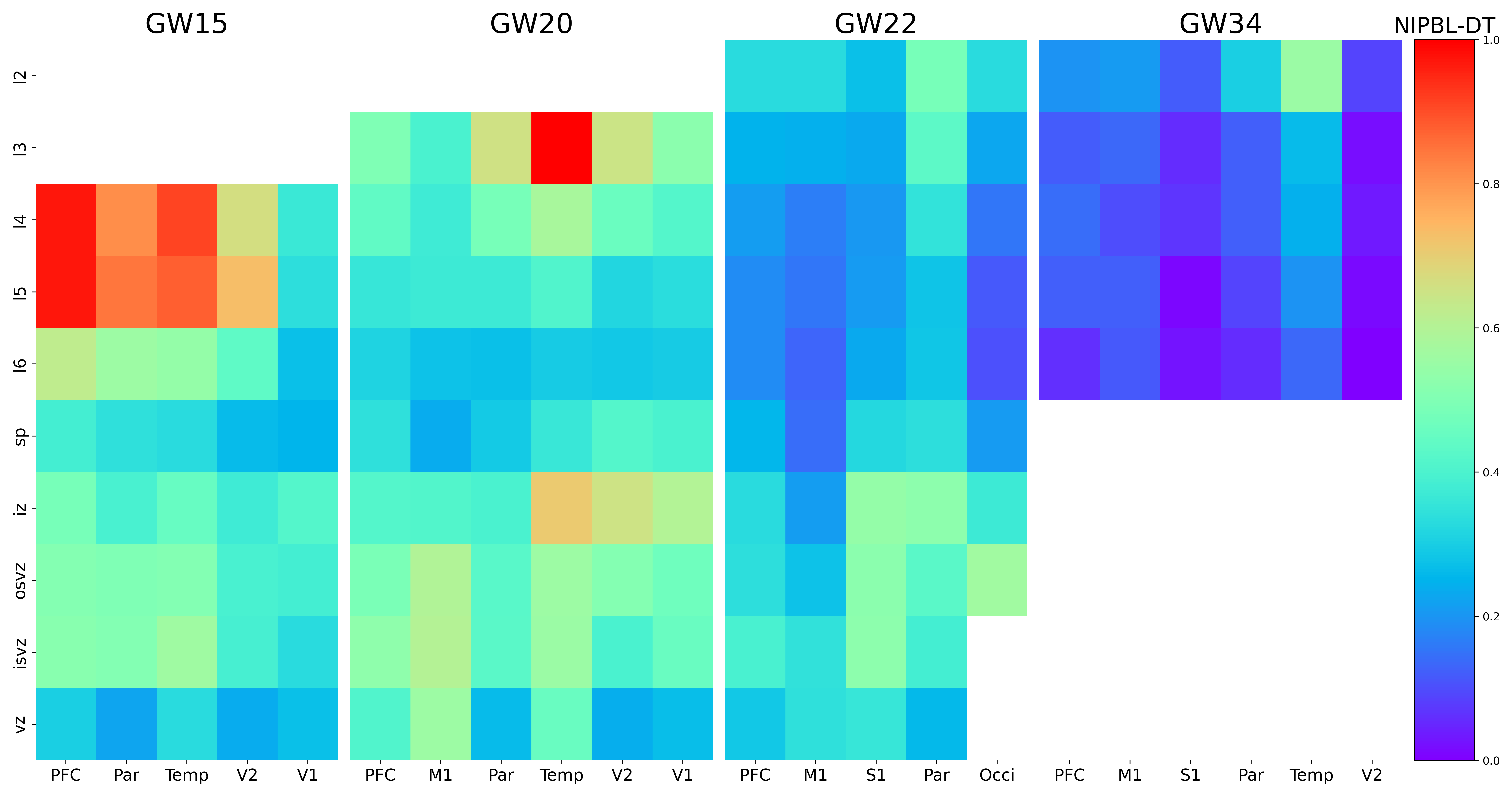

Supplement: Supplementary file 4 — Source Data Fig. 3: Expression pattern heatmap for all 300 genes in the MERFISH. [file 41586_2025_9010_MOESM4_ESM.zip › NIPBL-DT.png]

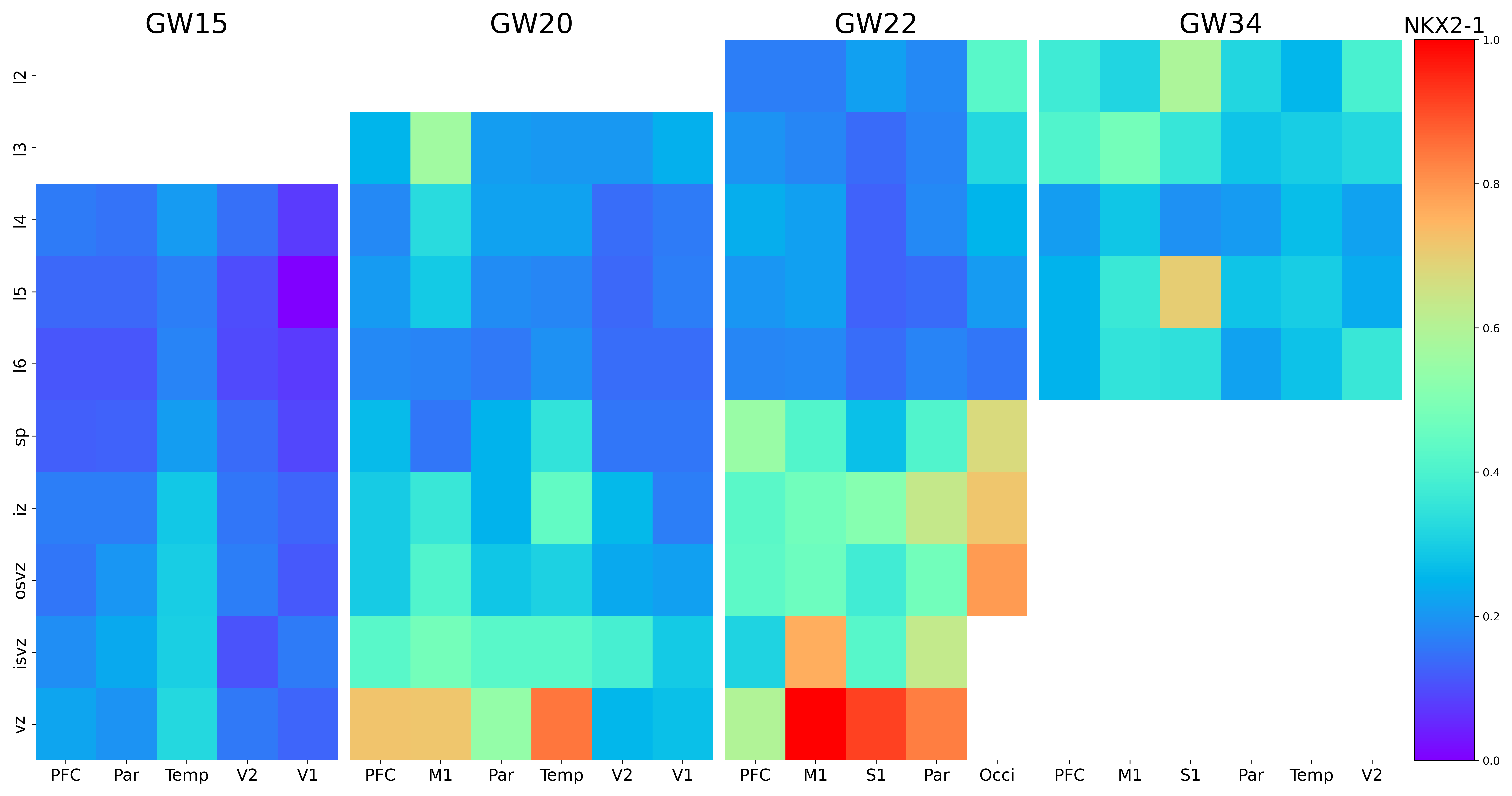

Supplement: Supplementary file 4 — Source Data Fig. 3: Expression pattern heatmap for all 300 genes in the MERFISH. [file 41586_2025_9010_MOESM4_ESM.zip › NKX2-1.png]

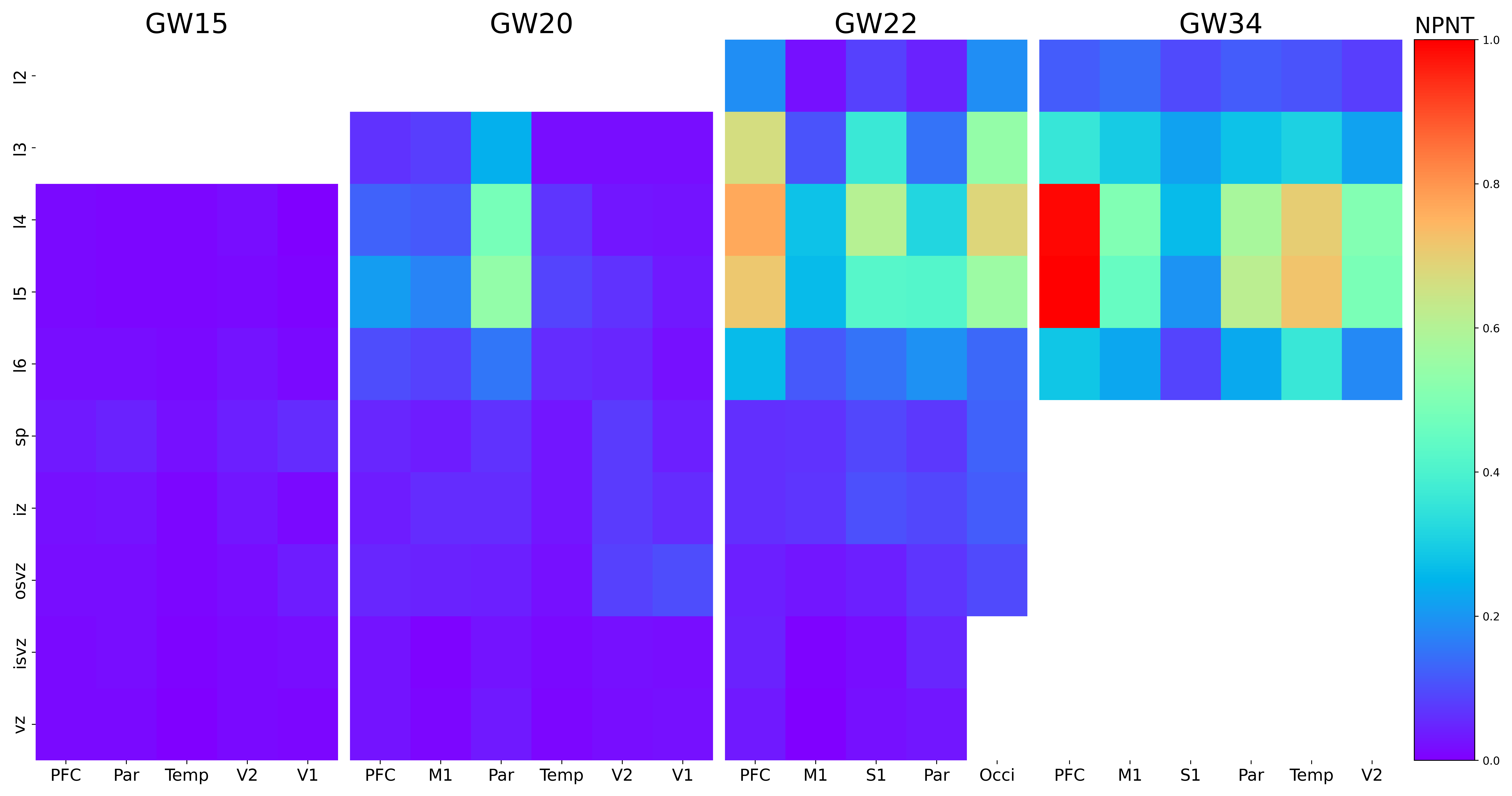

Supplement: Supplementary file 4 — Source Data Fig. 3: Expression pattern heatmap for all 300 genes in the MERFISH. [file 41586_2025_9010_MOESM4_ESM.zip › NPNT.png]

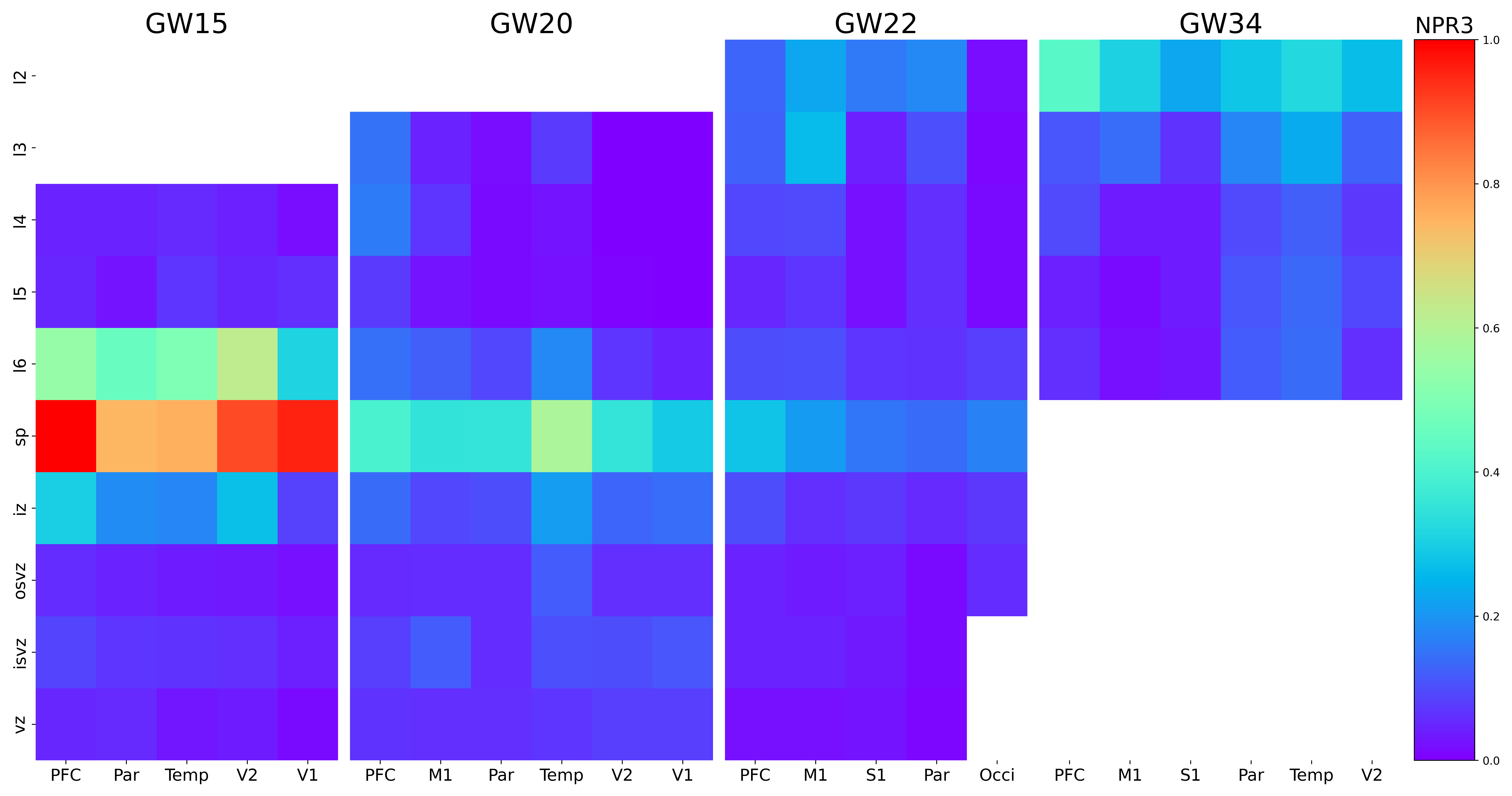

Supplement: Supplementary file 4 — Source Data Fig. 3: Expression pattern heatmap for all 300 genes in the MERFISH. [file 41586_2025_9010_MOESM4_ESM.zip › NPR3.png]

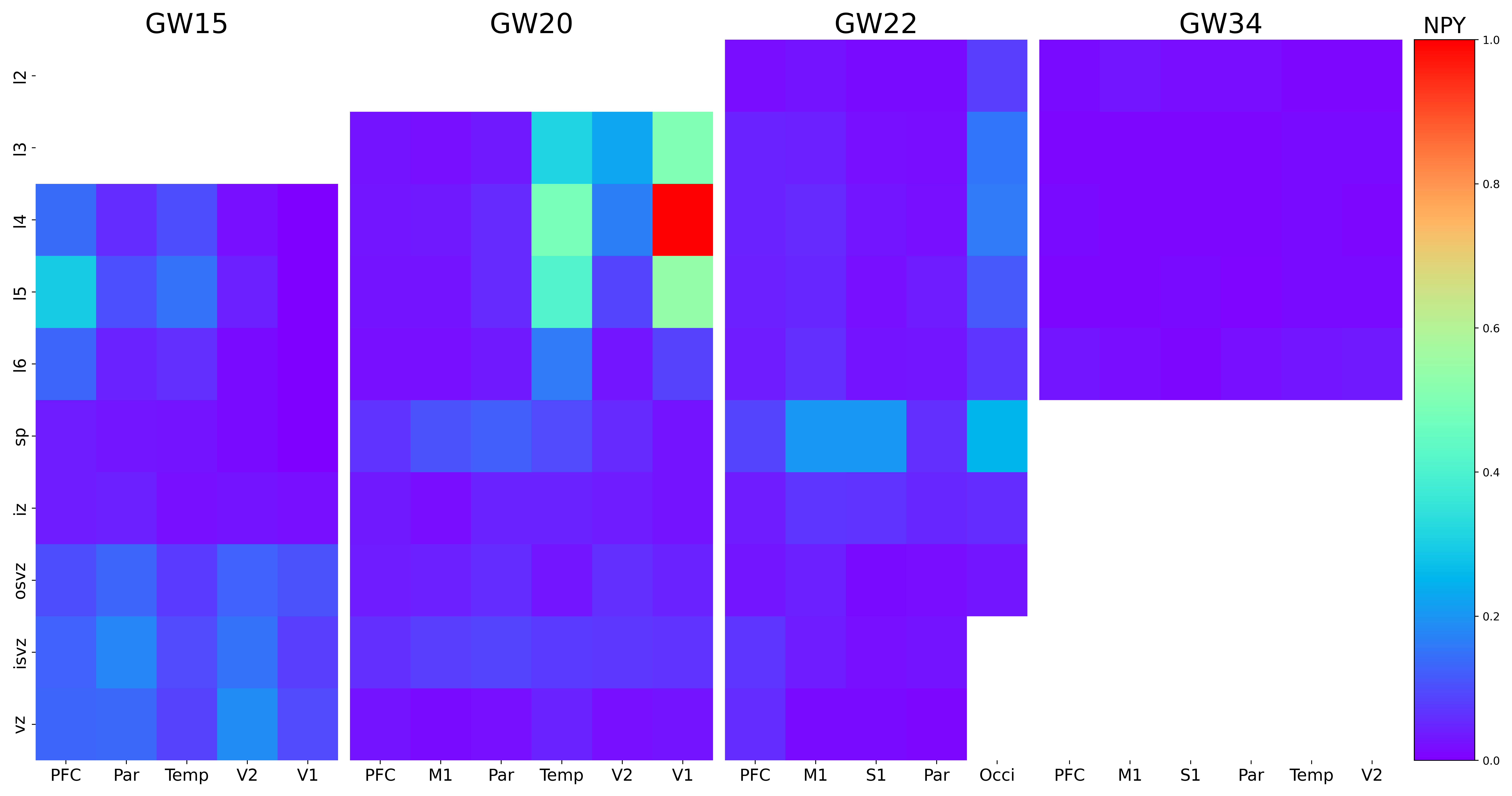

Supplement: Supplementary file 4 — Source Data Fig. 3: Expression pattern heatmap for all 300 genes in the MERFISH. [file 41586_2025_9010_MOESM4_ESM.zip › NPY.png]

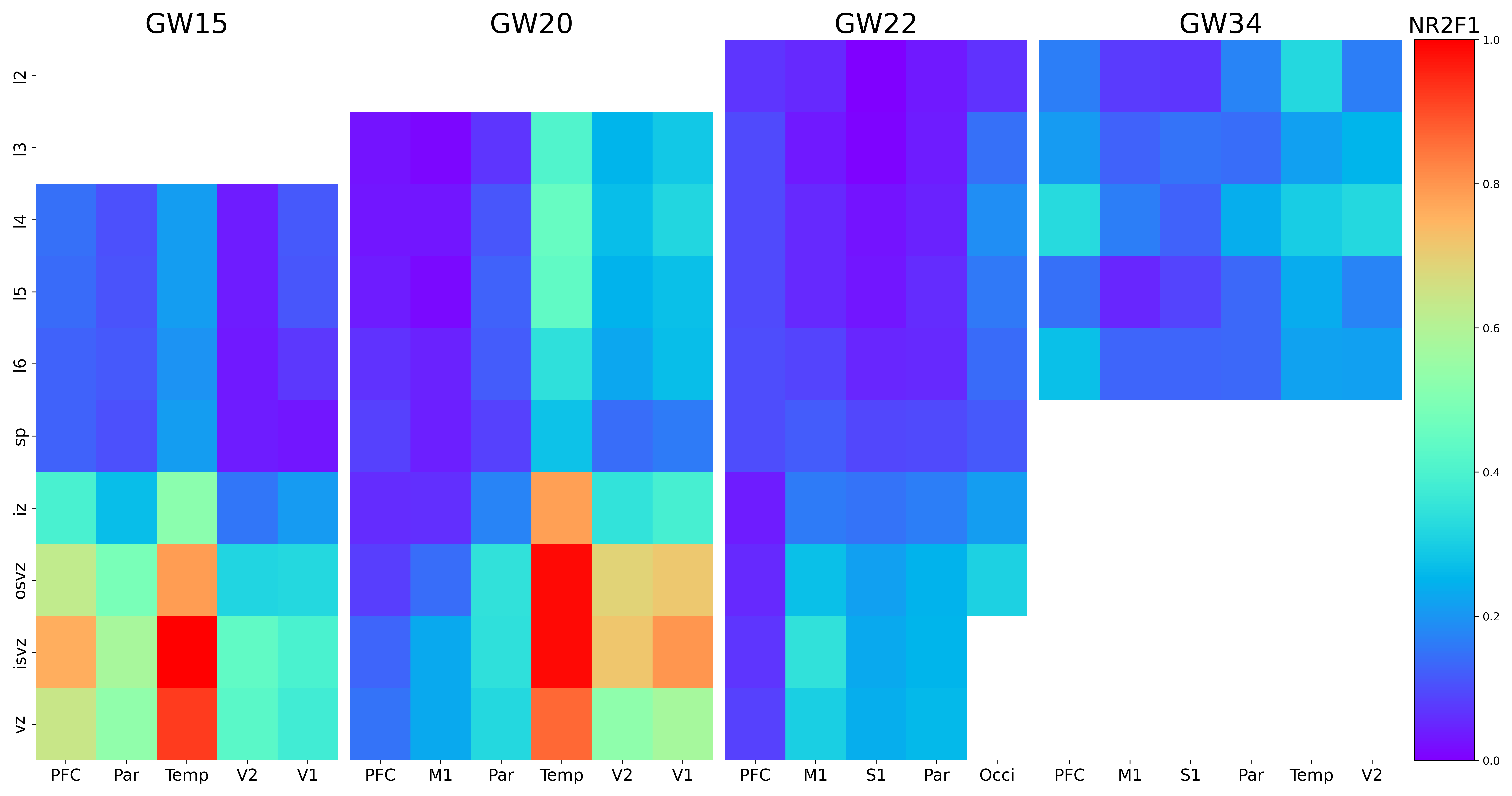

Supplement: Supplementary file 4 — Source Data Fig. 3: Expression pattern heatmap for all 300 genes in the MERFISH. [file 41586_2025_9010_MOESM4_ESM.zip › NR2F1.png]

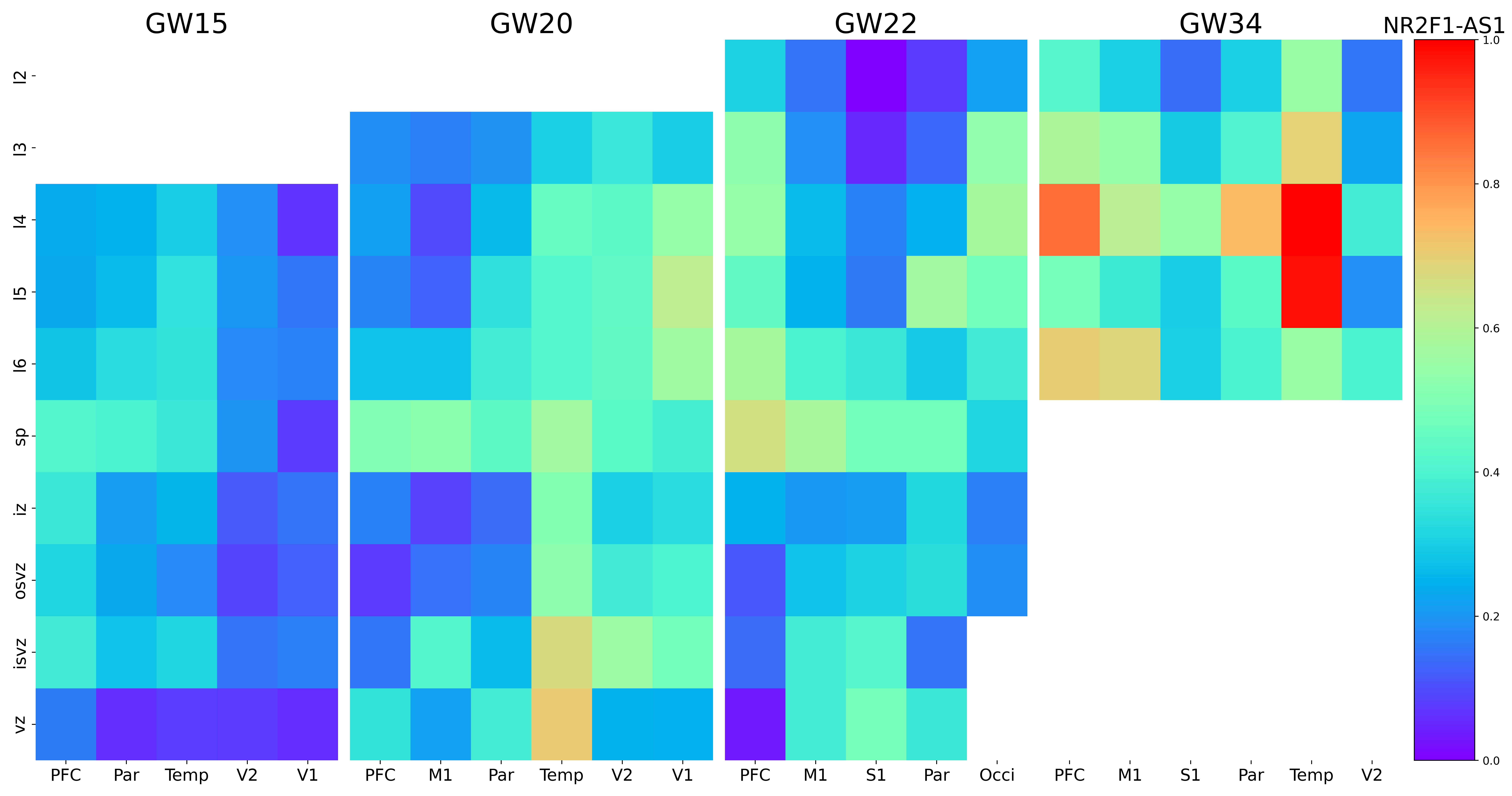

Supplement: Supplementary file 4 — Source Data Fig. 3: Expression pattern heatmap for all 300 genes in the MERFISH. [file 41586_2025_9010_MOESM4_ESM.zip › NR2F1-AS1.png]

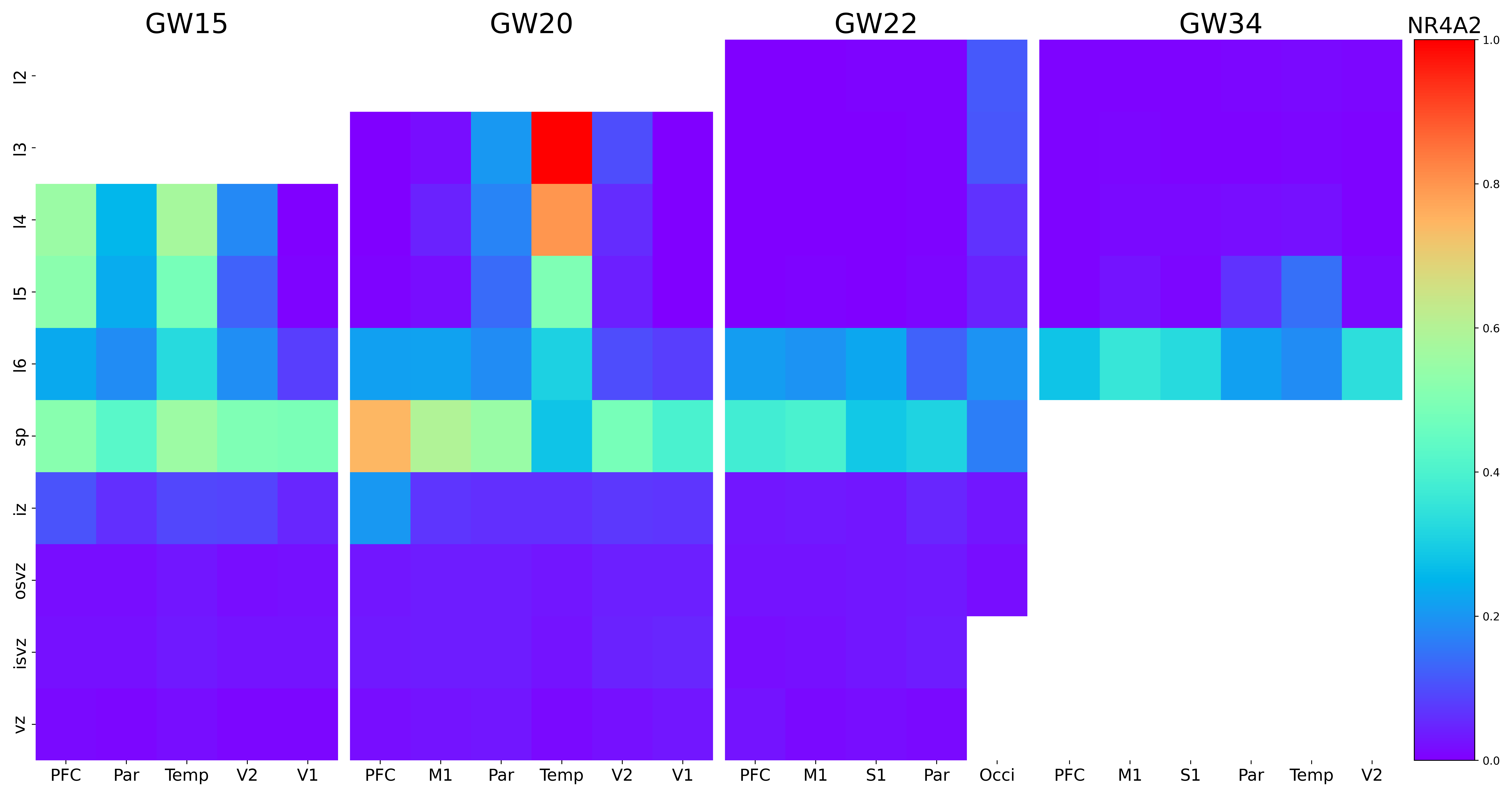

Supplement: Supplementary file 4 — Source Data Fig. 3: Expression pattern heatmap for all 300 genes in the MERFISH. [file 41586_2025_9010_MOESM4_ESM.zip › NR4A2.png]

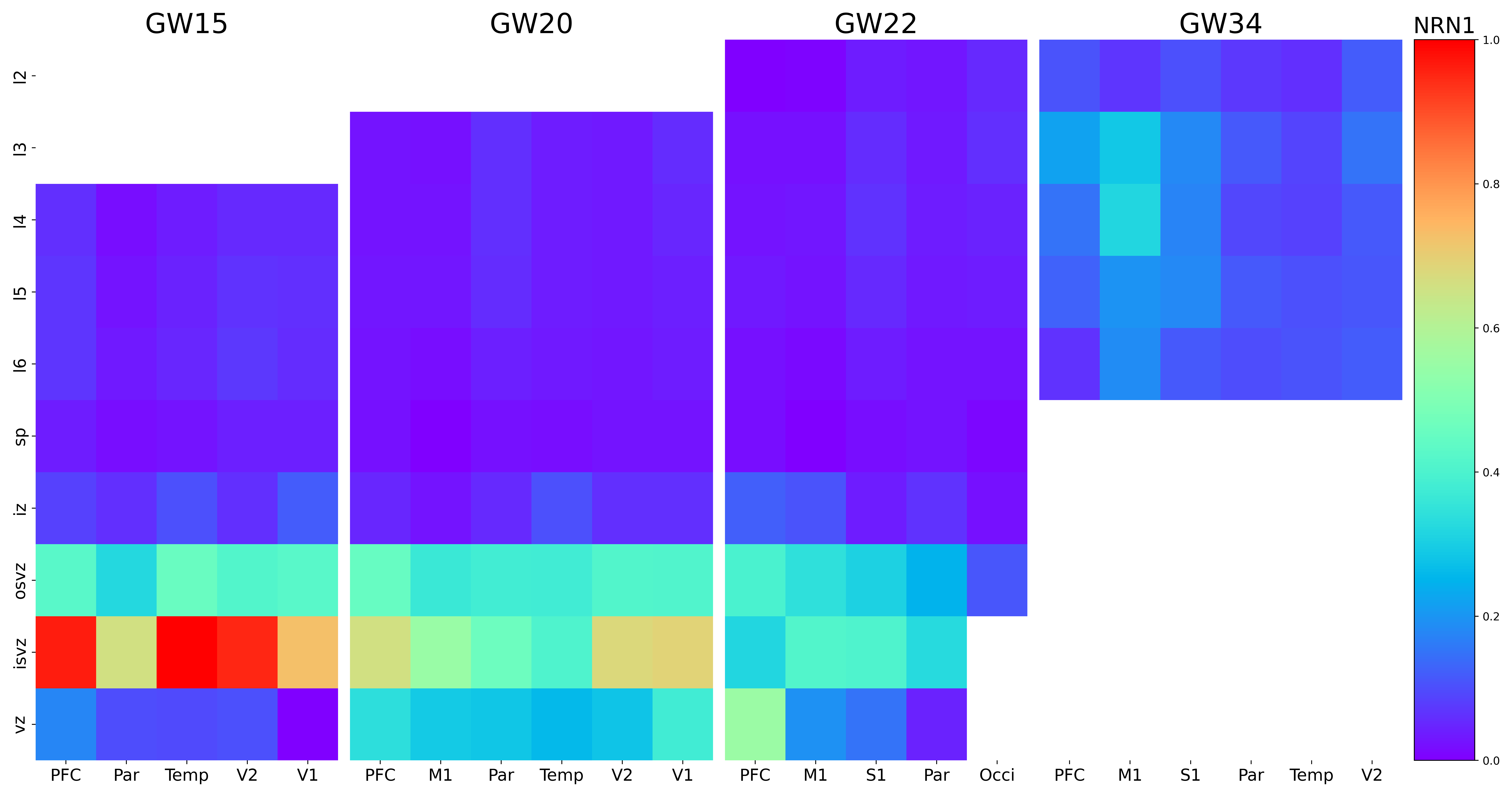

Supplement: Supplementary file 4 — Source Data Fig. 3: Expression pattern heatmap for all 300 genes in the MERFISH. [file 41586_2025_9010_MOESM4_ESM.zip › NRN1.png]
