# Supplementary figures and images for: Spatial transcriptomics reveals human cortical layer and area specification (part 1 of 2)
Source: Nature. 2025 May 14;644(8075):153–63. doi: 10.1038/s41586-025-09010-1 (PMC12328223; doi:10.1038/s41586-025-09010-1)

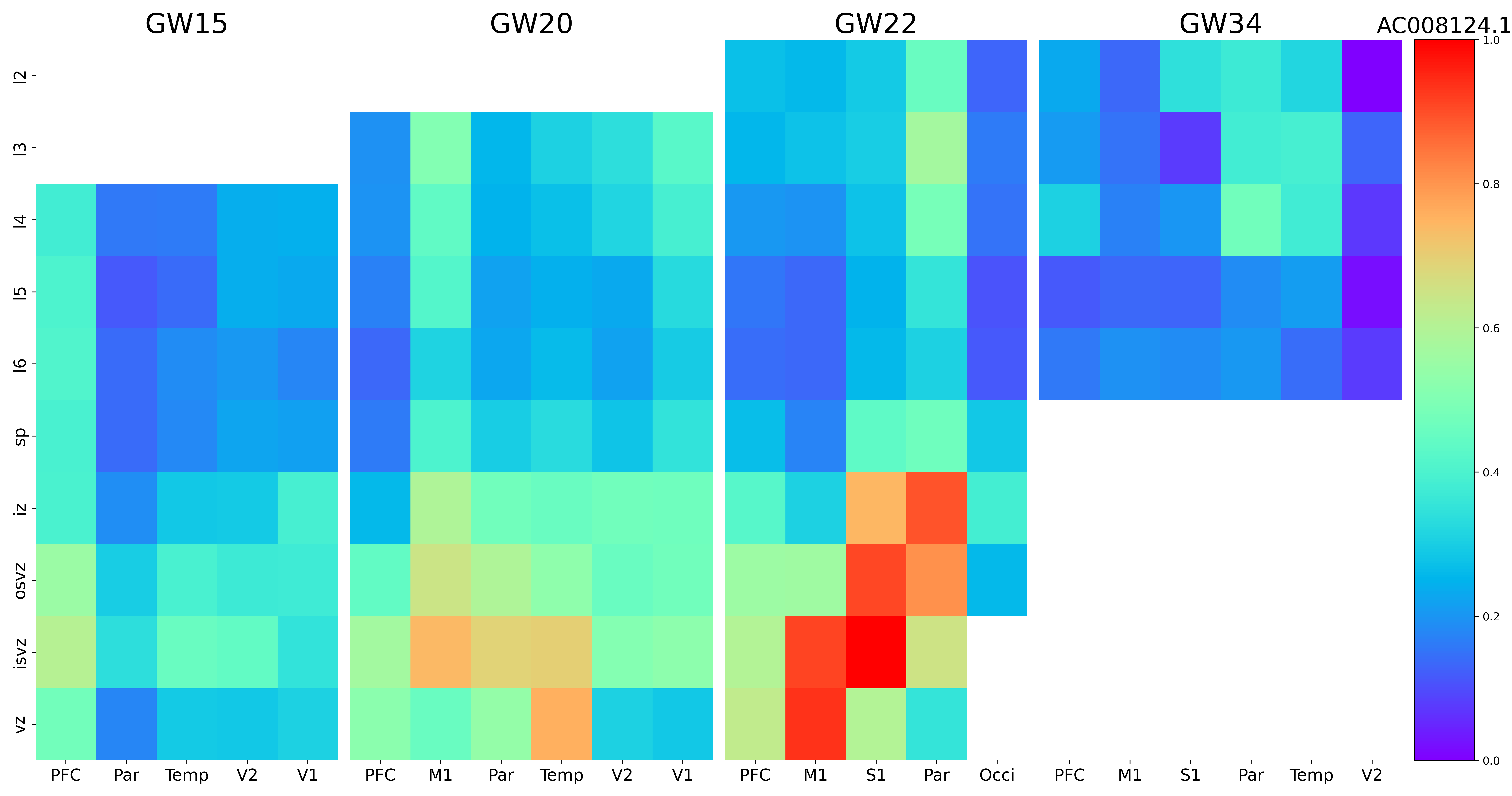

Supplement: Supplementary file 4 — Source Data Fig. 3: Expression pattern heatmap for all 300 genes in the MERFISH. [file 41586_2025_9010_MOESM4_ESM.zip › AC008124.1.png]

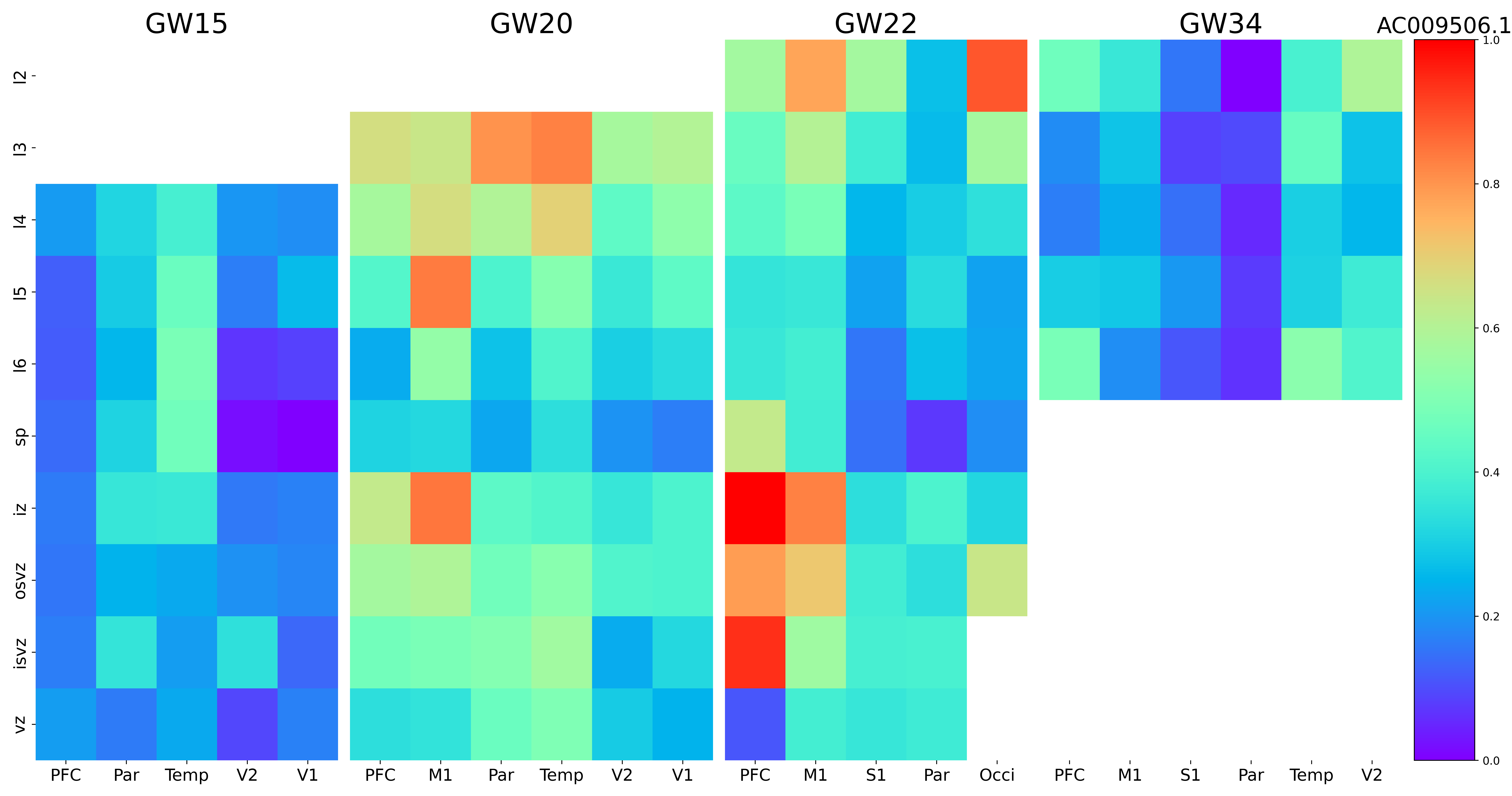

Supplement: Supplementary file 4 — Source Data Fig. 3: Expression pattern heatmap for all 300 genes in the MERFISH. [file 41586_2025_9010_MOESM4_ESM.zip › AC009506.1.png]

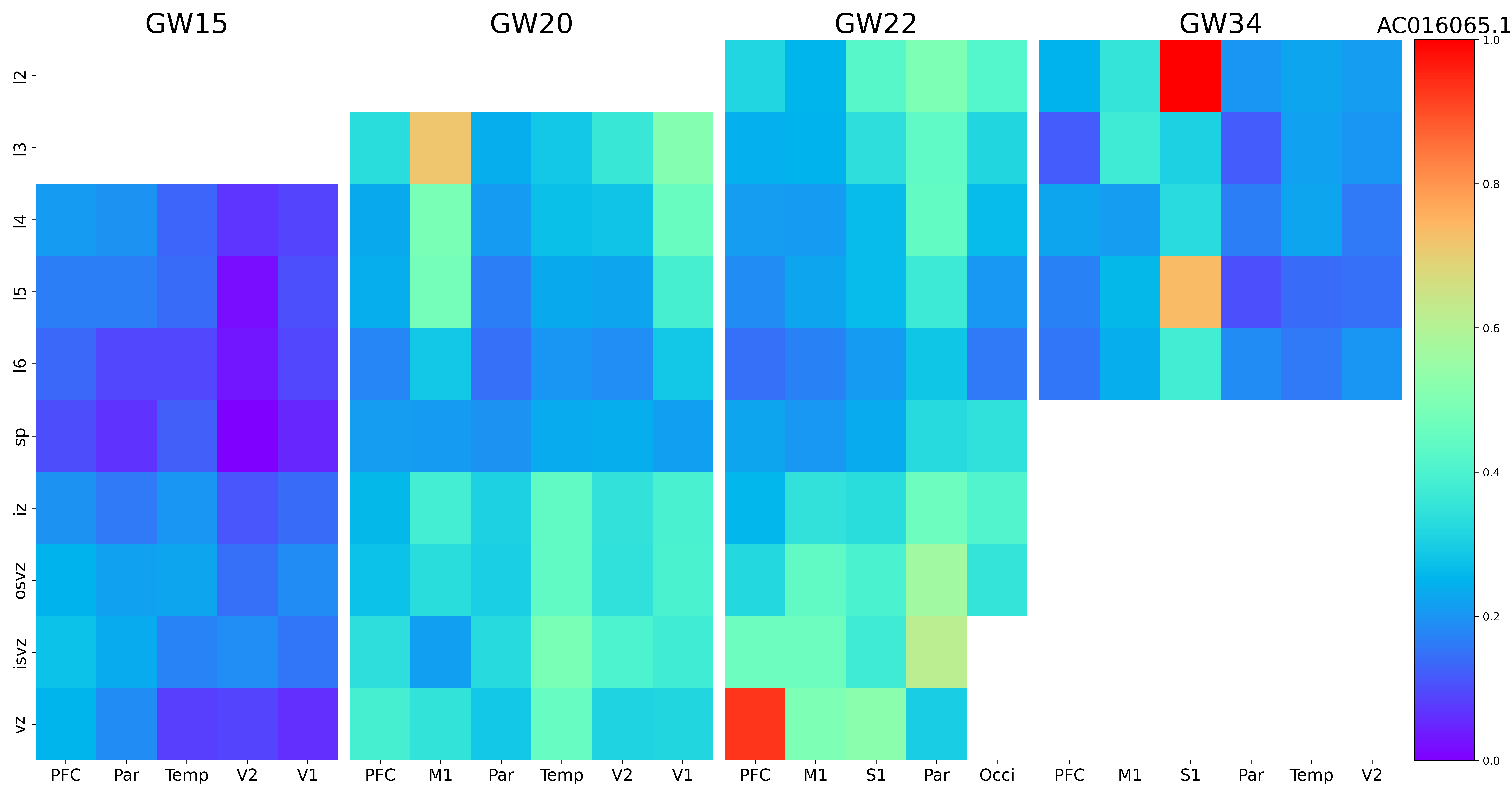

Supplement: Supplementary file 4 — Source Data Fig. 3: Expression pattern heatmap for all 300 genes in the MERFISH. [file 41586_2025_9010_MOESM4_ESM.zip › AC016065.1.png]

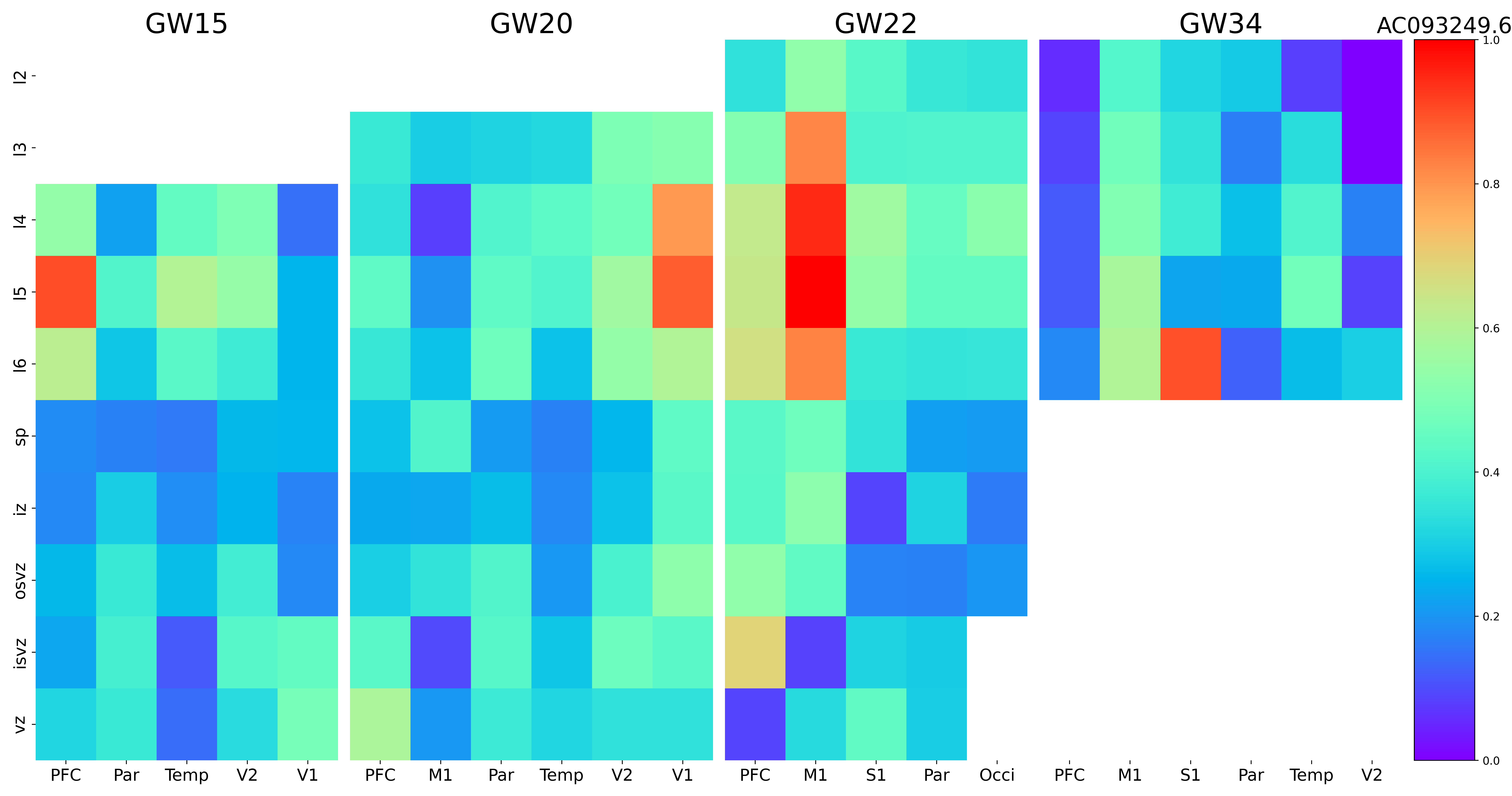

Supplement: Supplementary file 4 — Source Data Fig. 3: Expression pattern heatmap for all 300 genes in the MERFISH. [file 41586_2025_9010_MOESM4_ESM.zip › AC093249.6.png]

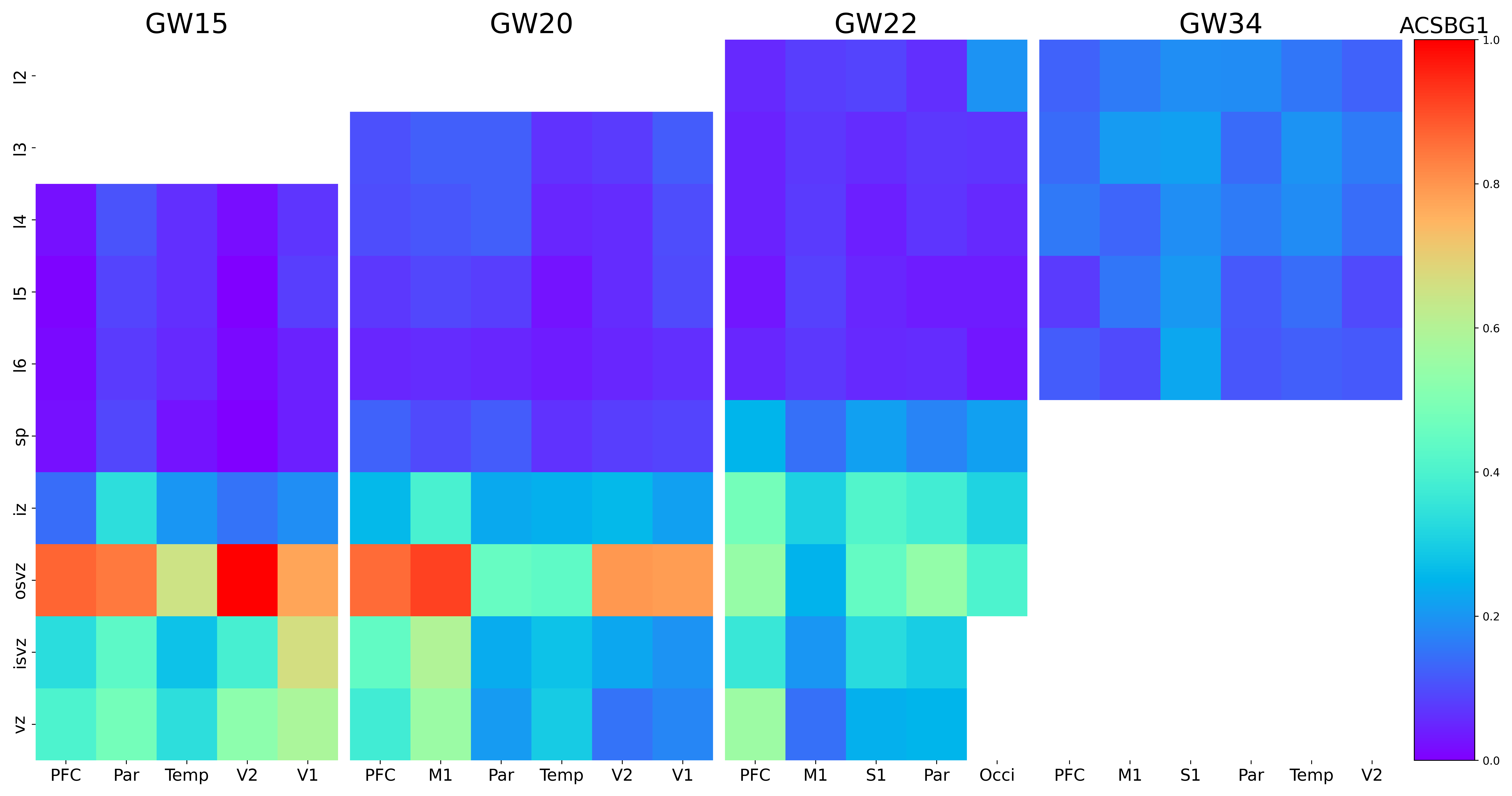

Supplement: Supplementary file 4 — Source Data Fig. 3: Expression pattern heatmap for all 300 genes in the MERFISH. [file 41586_2025_9010_MOESM4_ESM.zip › ACSBG1.png]

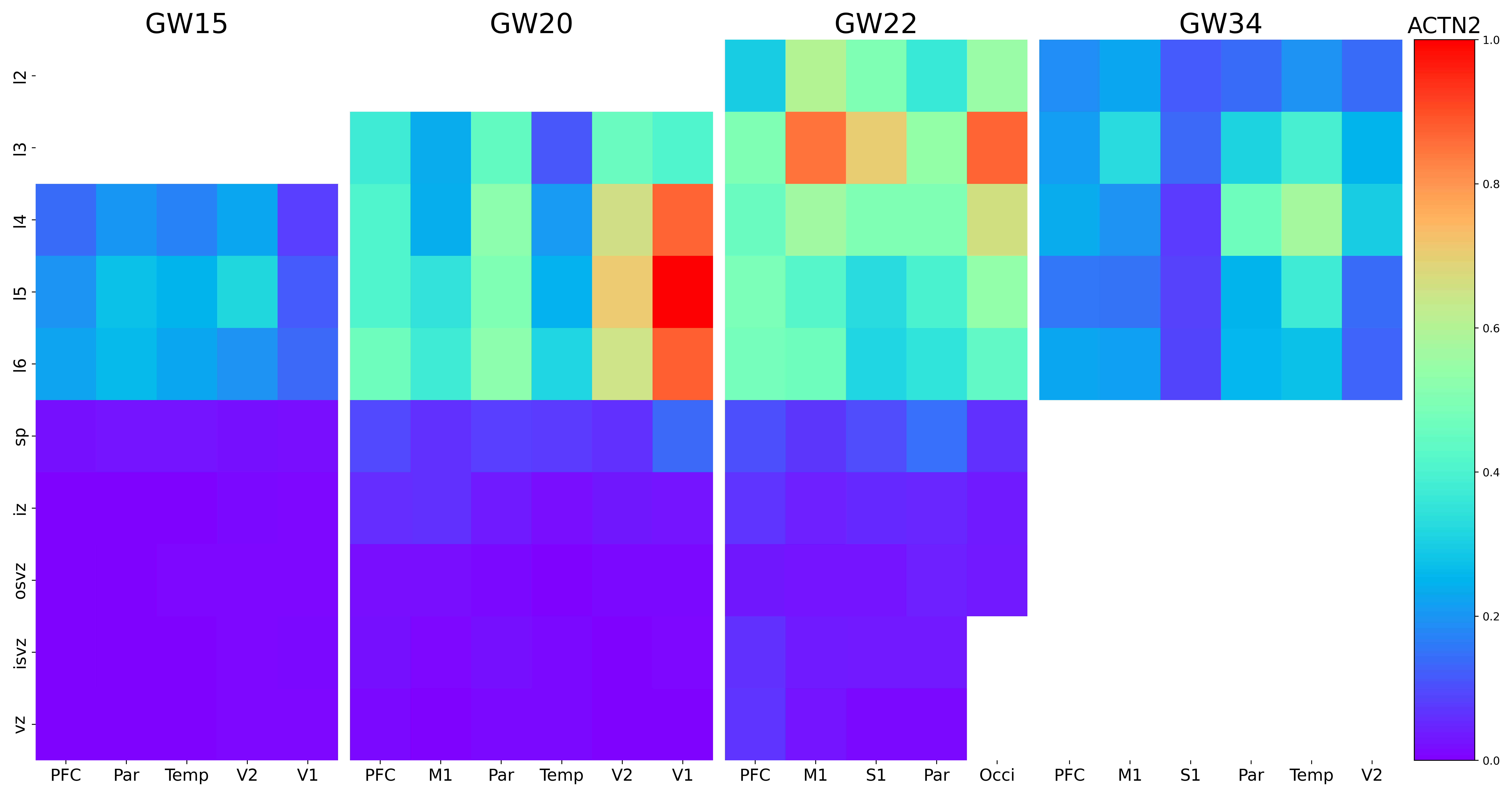

Supplement: Supplementary file 4 — Source Data Fig. 3: Expression pattern heatmap for all 300 genes in the MERFISH. [file 41586_2025_9010_MOESM4_ESM.zip › ACTN2.png]

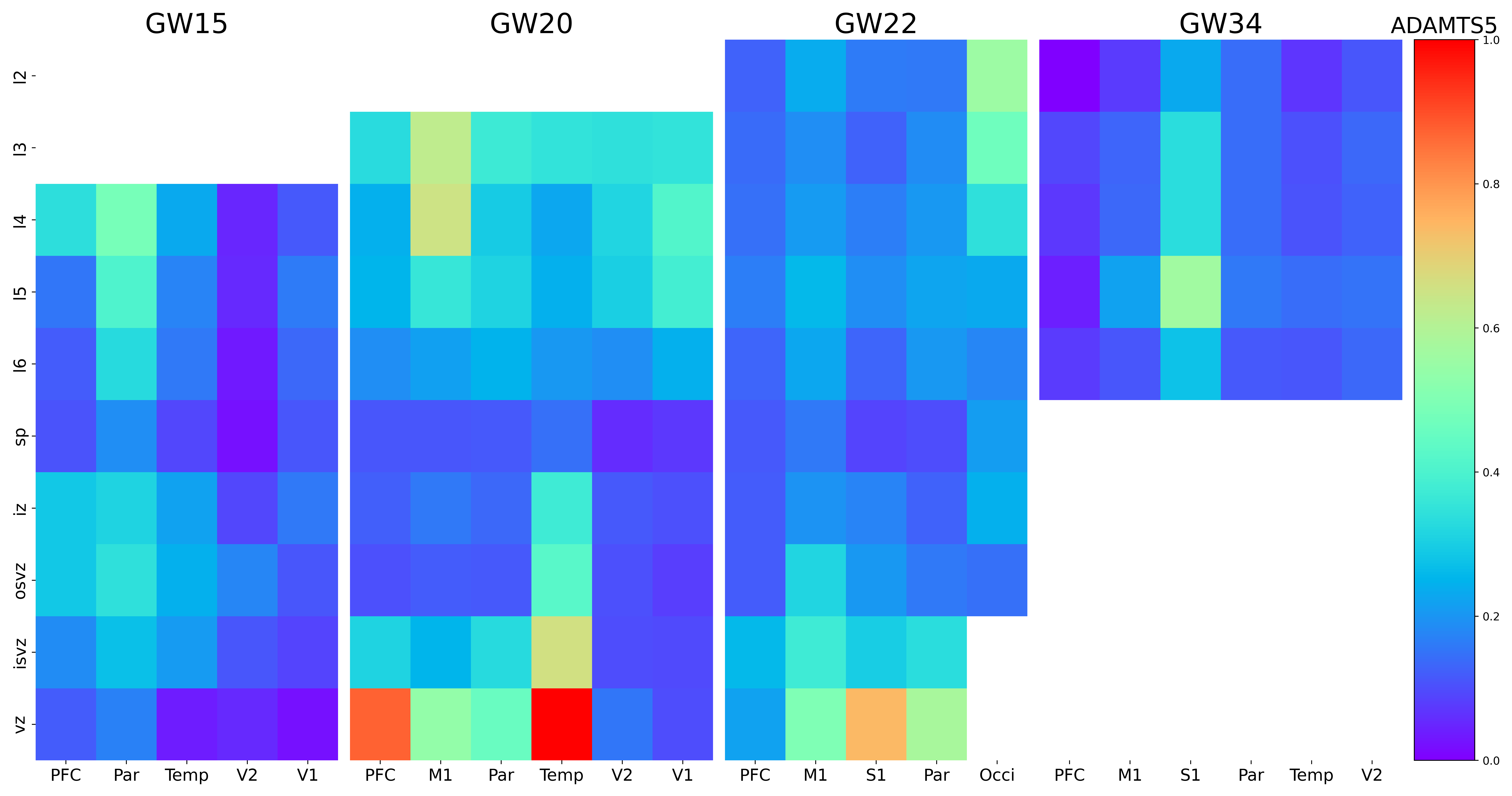

Supplement: Supplementary file 4 — Source Data Fig. 3: Expression pattern heatmap for all 300 genes in the MERFISH. [file 41586_2025_9010_MOESM4_ESM.zip › ADAMTS5.png]

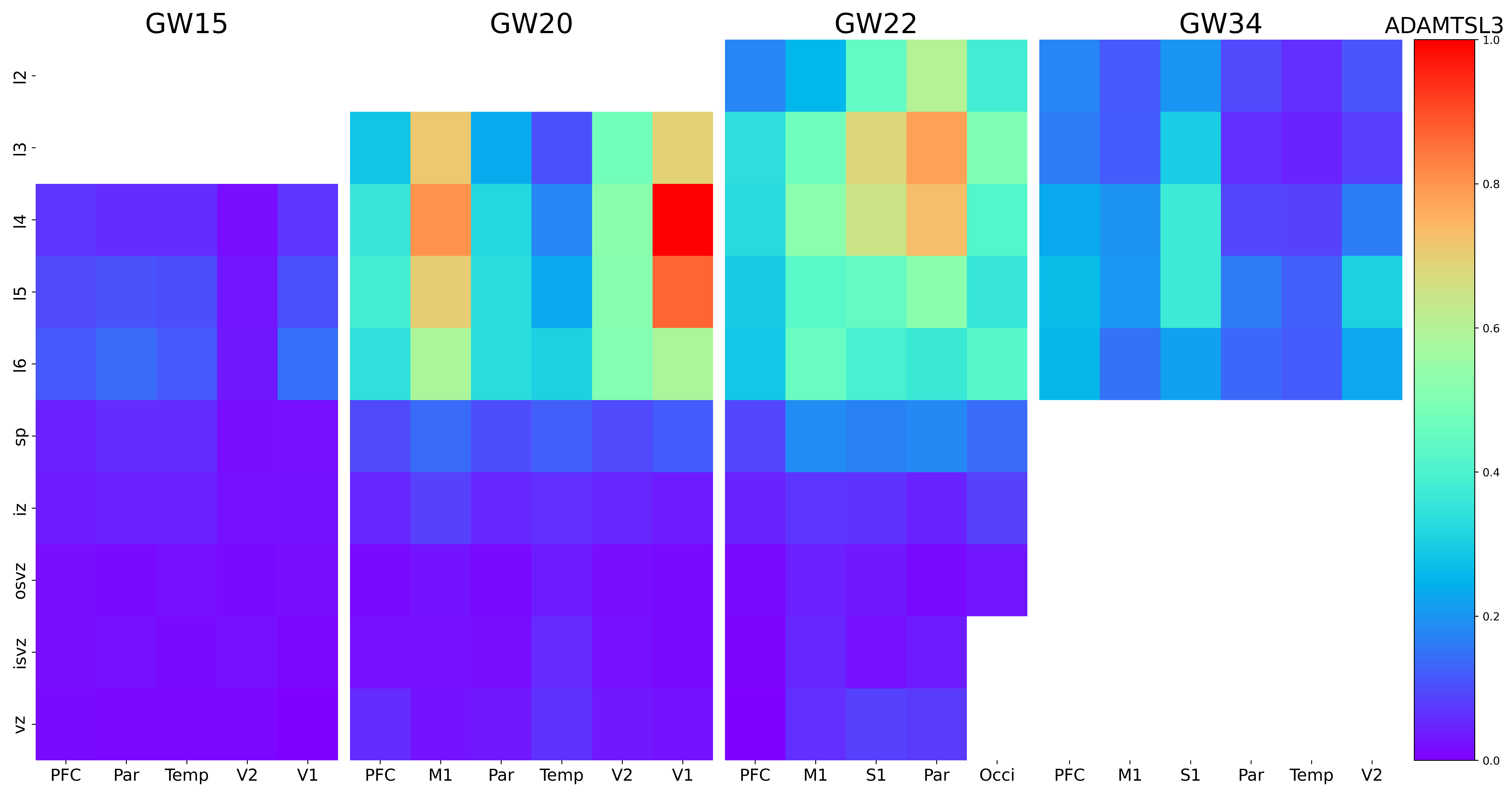

Supplement: Supplementary file 4 — Source Data Fig. 3: Expression pattern heatmap for all 300 genes in the MERFISH. [file 41586_2025_9010_MOESM4_ESM.zip › ADAMTSL3.png]

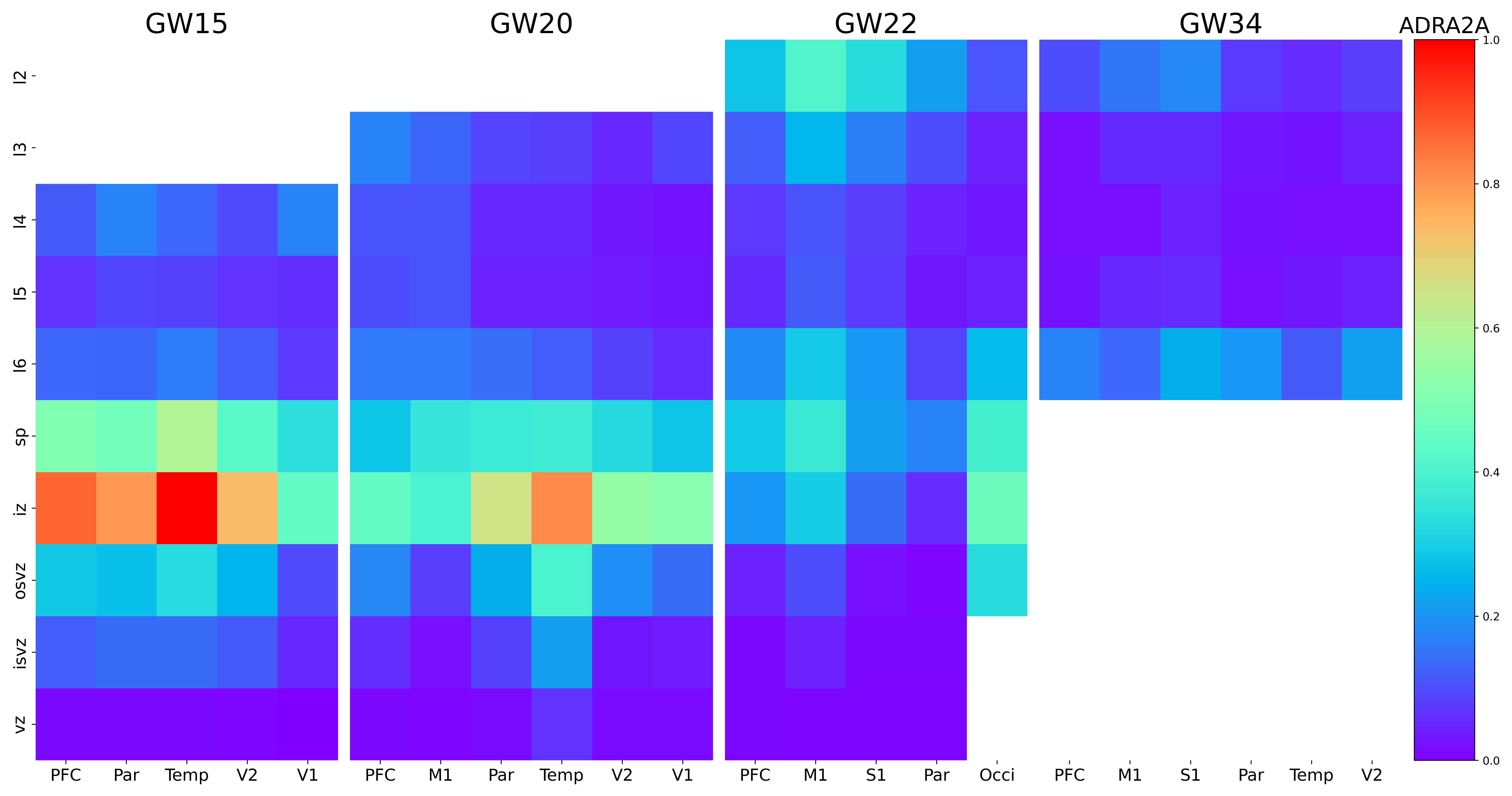

Supplement: Supplementary file 4 — Source Data Fig. 3: Expression pattern heatmap for all 300 genes in the MERFISH. [file 41586_2025_9010_MOESM4_ESM.zip › ADRA2A.png]

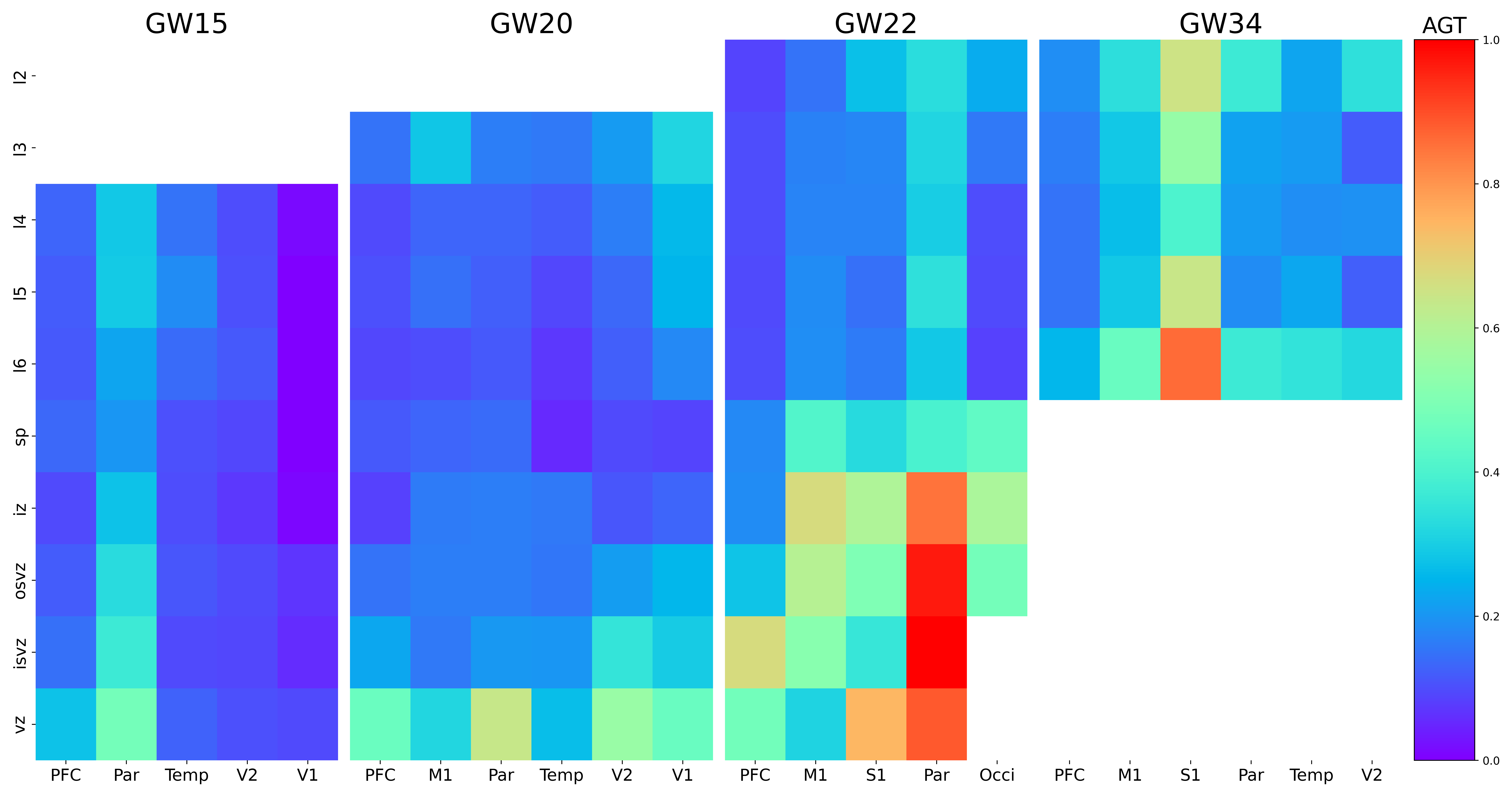

Supplement: Supplementary file 4 — Source Data Fig. 3: Expression pattern heatmap for all 300 genes in the MERFISH. [file 41586_2025_9010_MOESM4_ESM.zip › AGT.png]

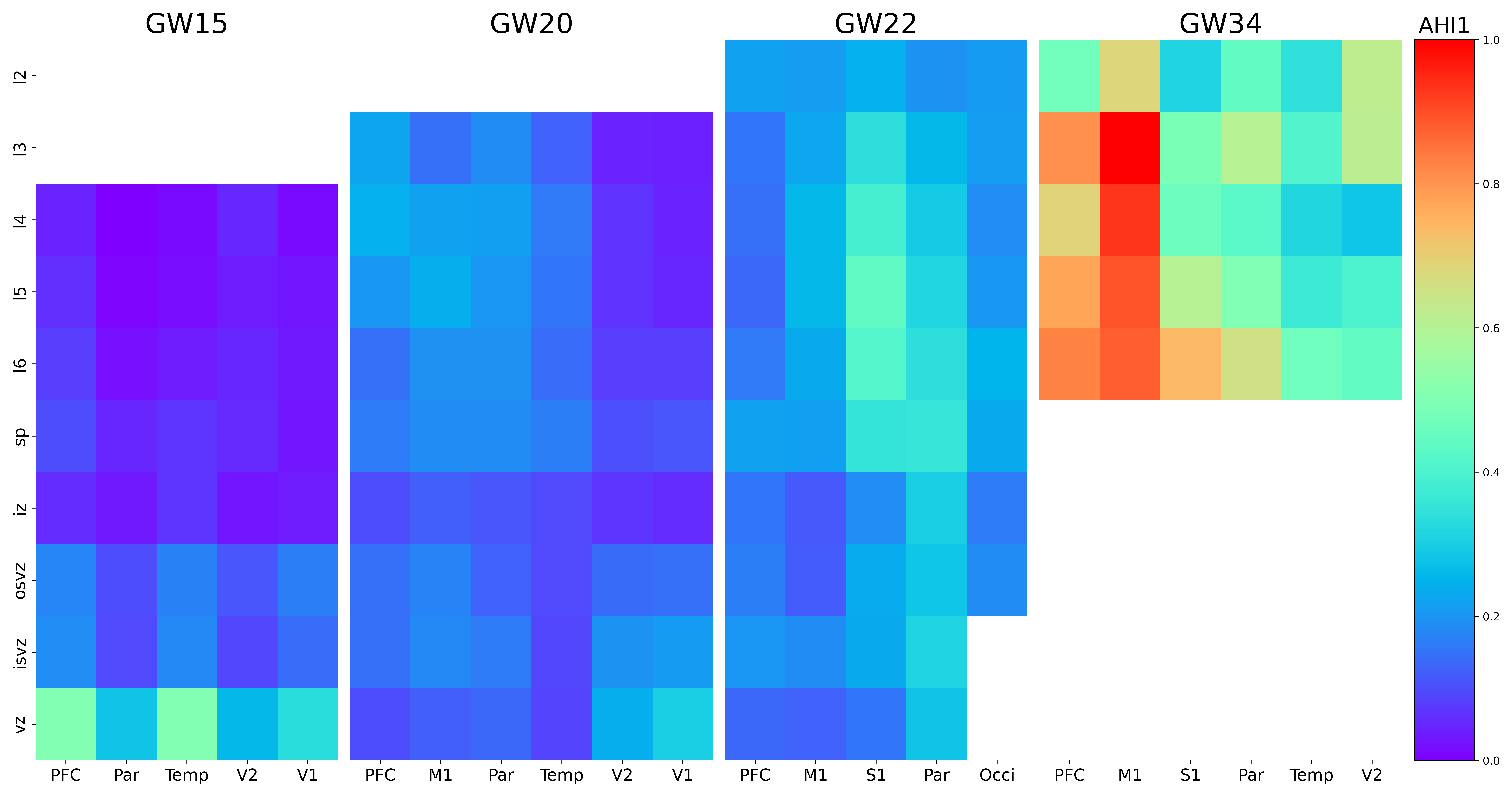

Supplement: Supplementary file 4 — Source Data Fig. 3: Expression pattern heatmap for all 300 genes in the MERFISH. [file 41586_2025_9010_MOESM4_ESM.zip › AHI1.png]

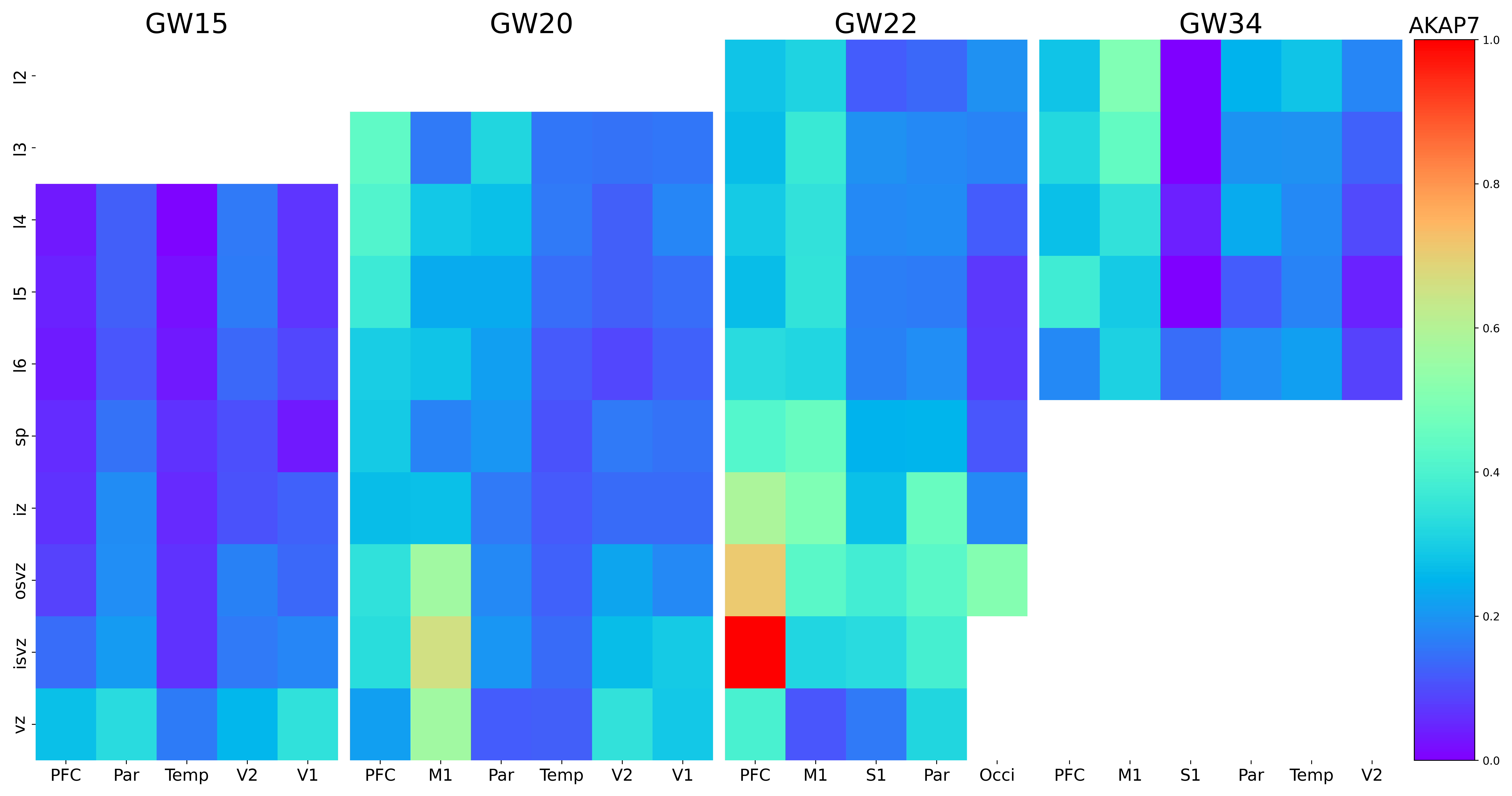

Supplement: Supplementary file 4 — Source Data Fig. 3: Expression pattern heatmap for all 300 genes in the MERFISH. [file 41586_2025_9010_MOESM4_ESM.zip › AKAP7.png]

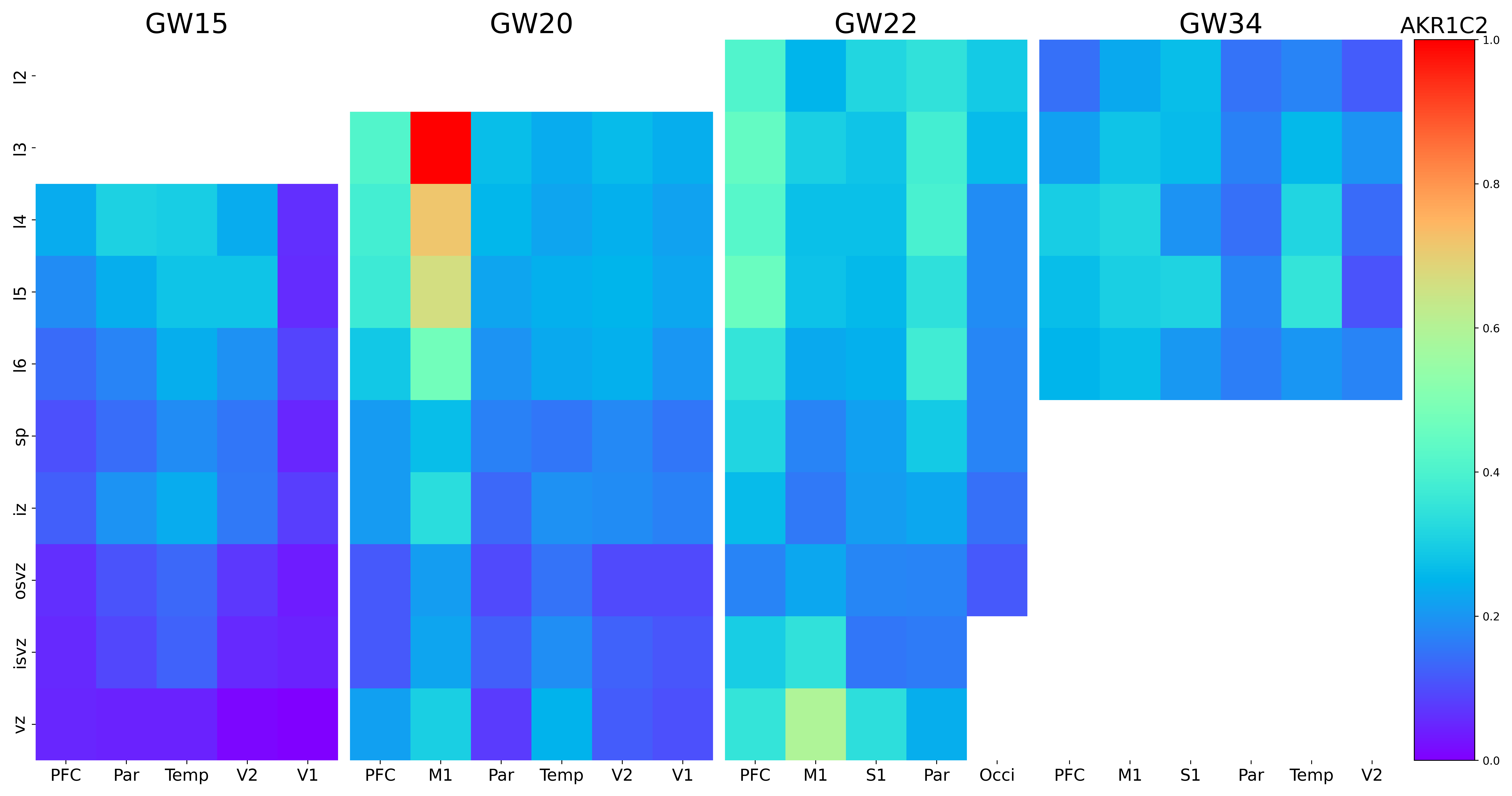

Supplement: Supplementary file 4 — Source Data Fig. 3: Expression pattern heatmap for all 300 genes in the MERFISH. [file 41586_2025_9010_MOESM4_ESM.zip › AKR1C2.png]

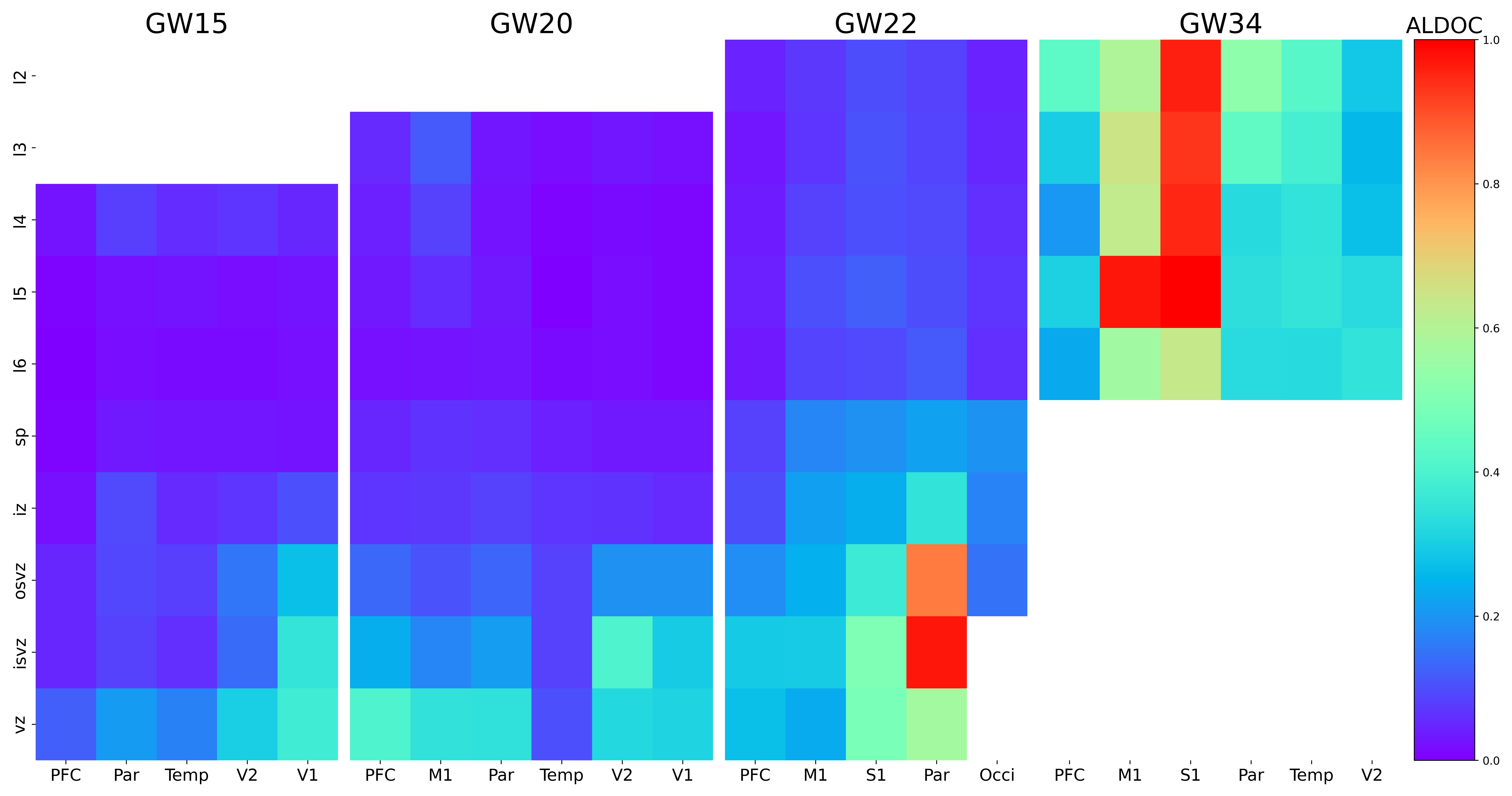

Supplement: Supplementary file 4 — Source Data Fig. 3: Expression pattern heatmap for all 300 genes in the MERFISH. [file 41586_2025_9010_MOESM4_ESM.zip › ALDOC.png]

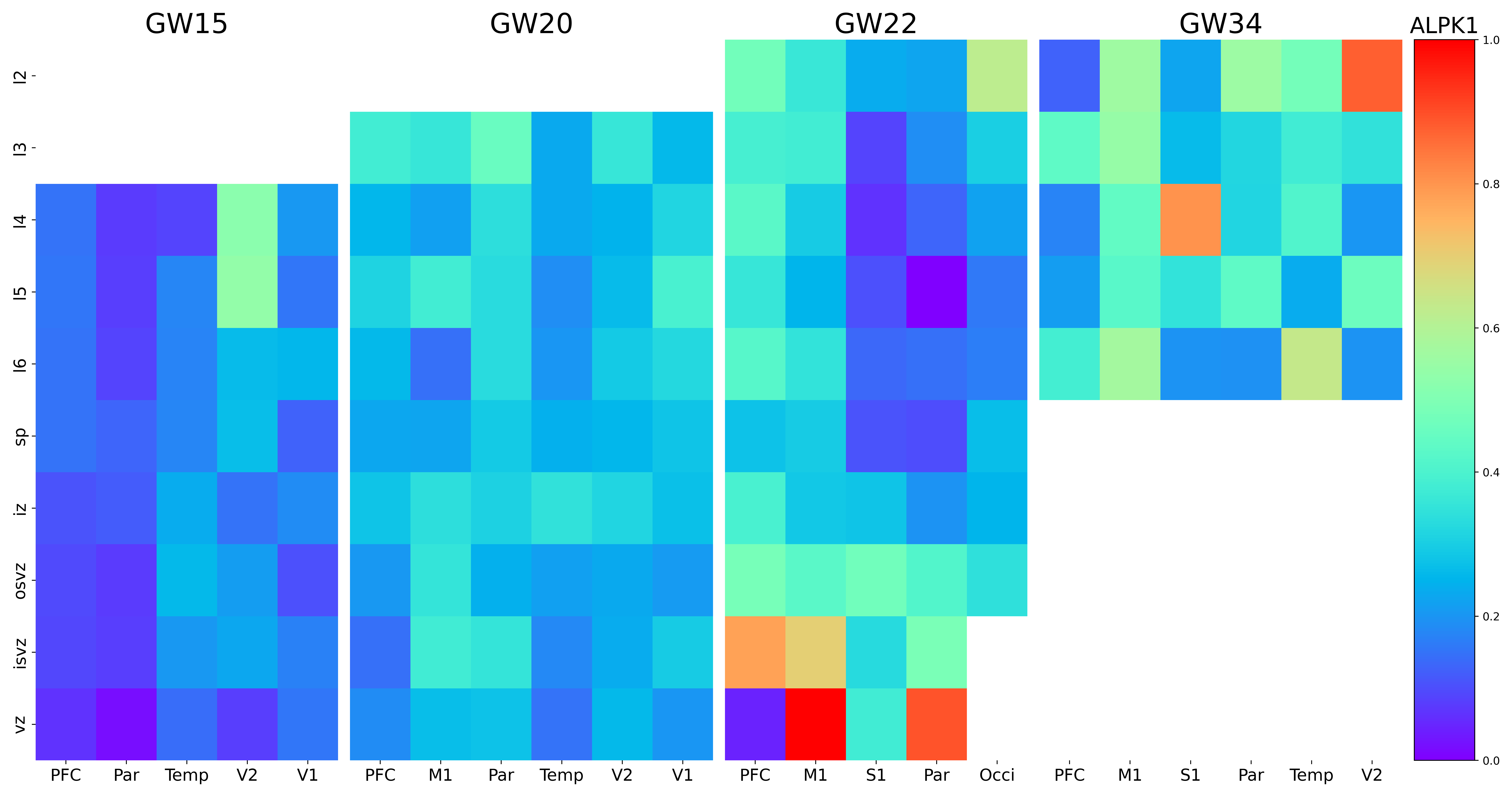

Supplement: Supplementary file 4 — Source Data Fig. 3: Expression pattern heatmap for all 300 genes in the MERFISH. [file 41586_2025_9010_MOESM4_ESM.zip › ALPK1.png]

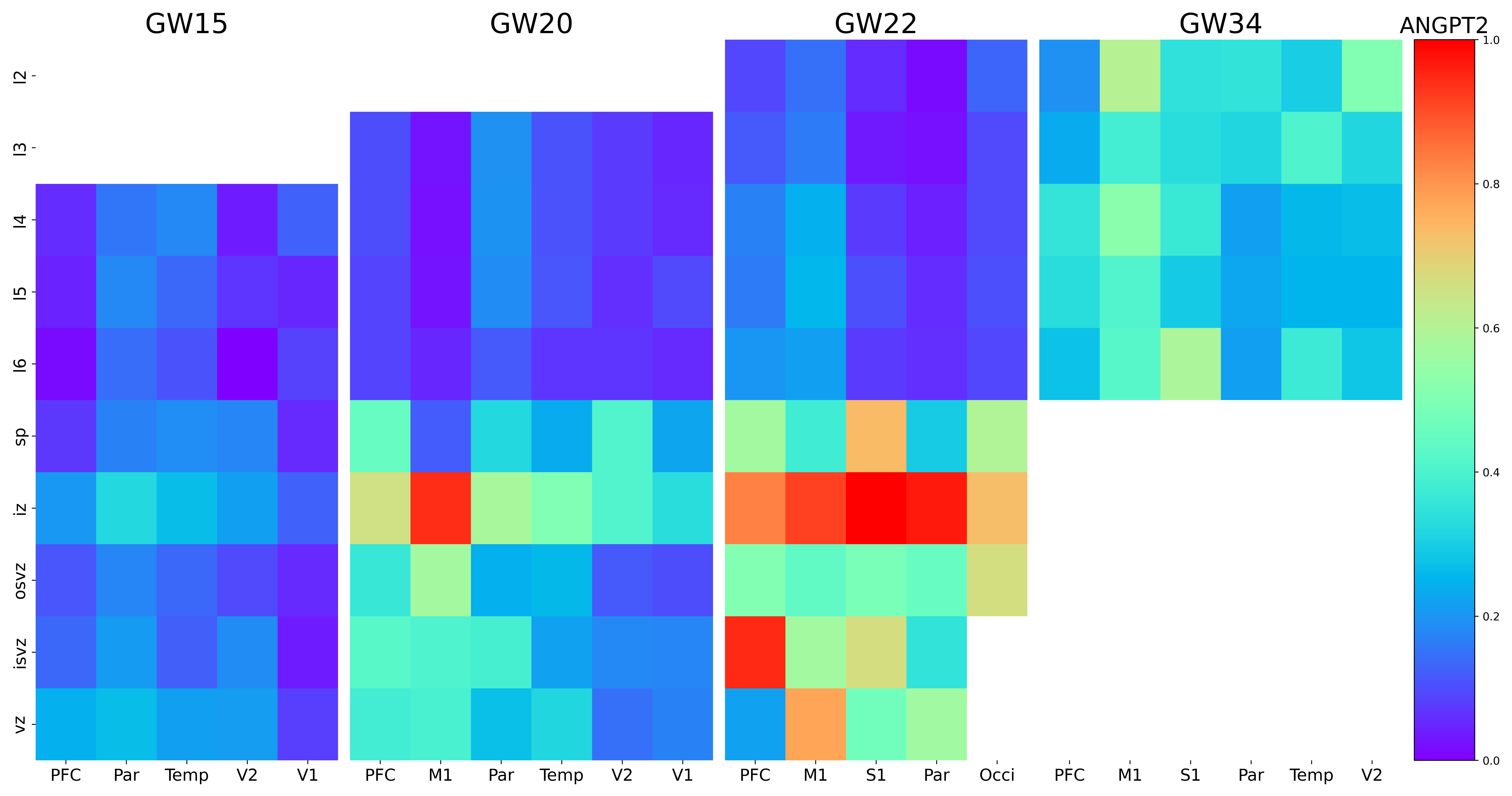

Supplement: Supplementary file 4 — Source Data Fig. 3: Expression pattern heatmap for all 300 genes in the MERFISH. [file 41586_2025_9010_MOESM4_ESM.zip › ANGPT2.png]

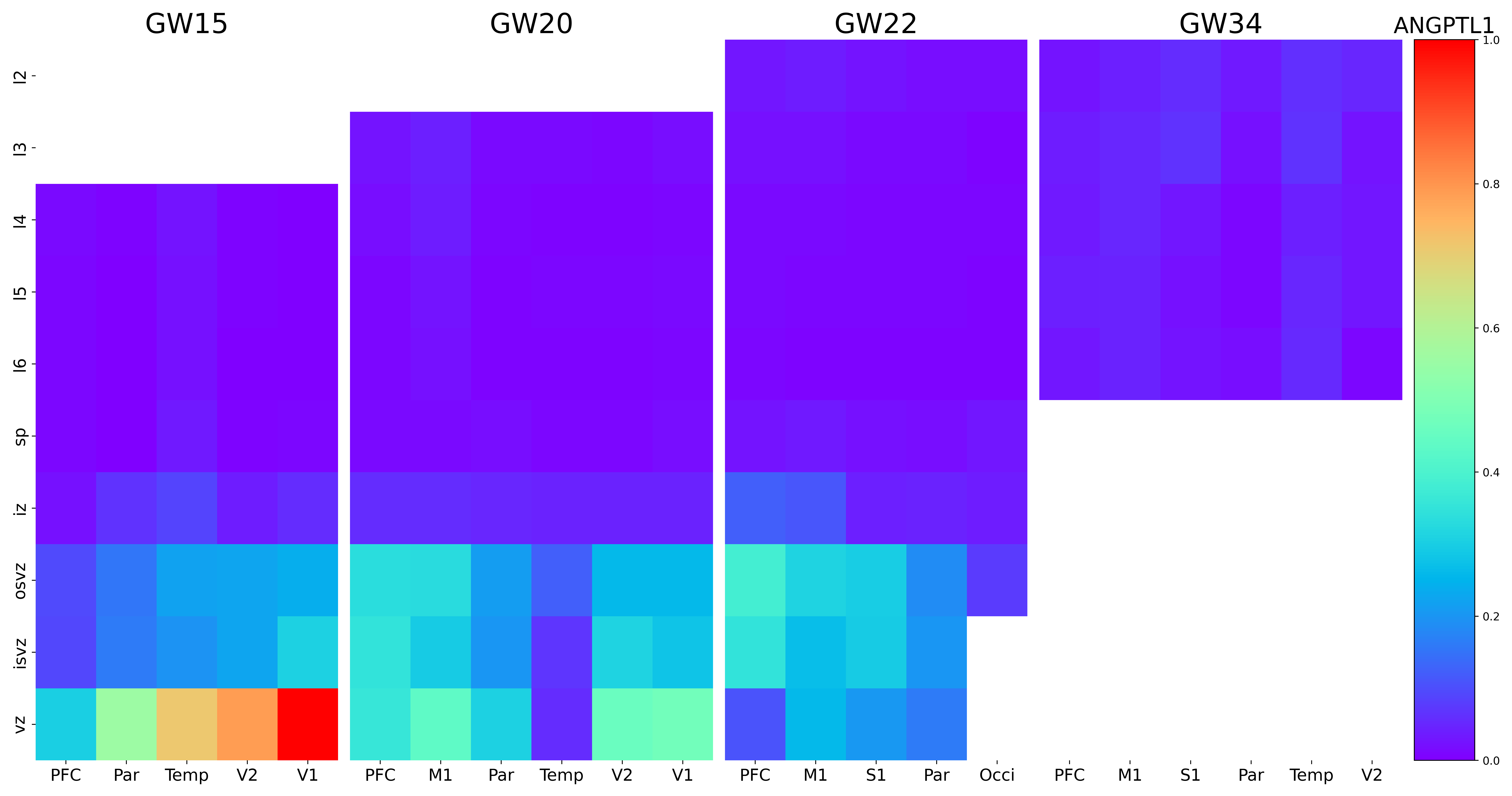

Supplement: Supplementary file 4 — Source Data Fig. 3: Expression pattern heatmap for all 300 genes in the MERFISH. [file 41586_2025_9010_MOESM4_ESM.zip › ANGPTL1.png]

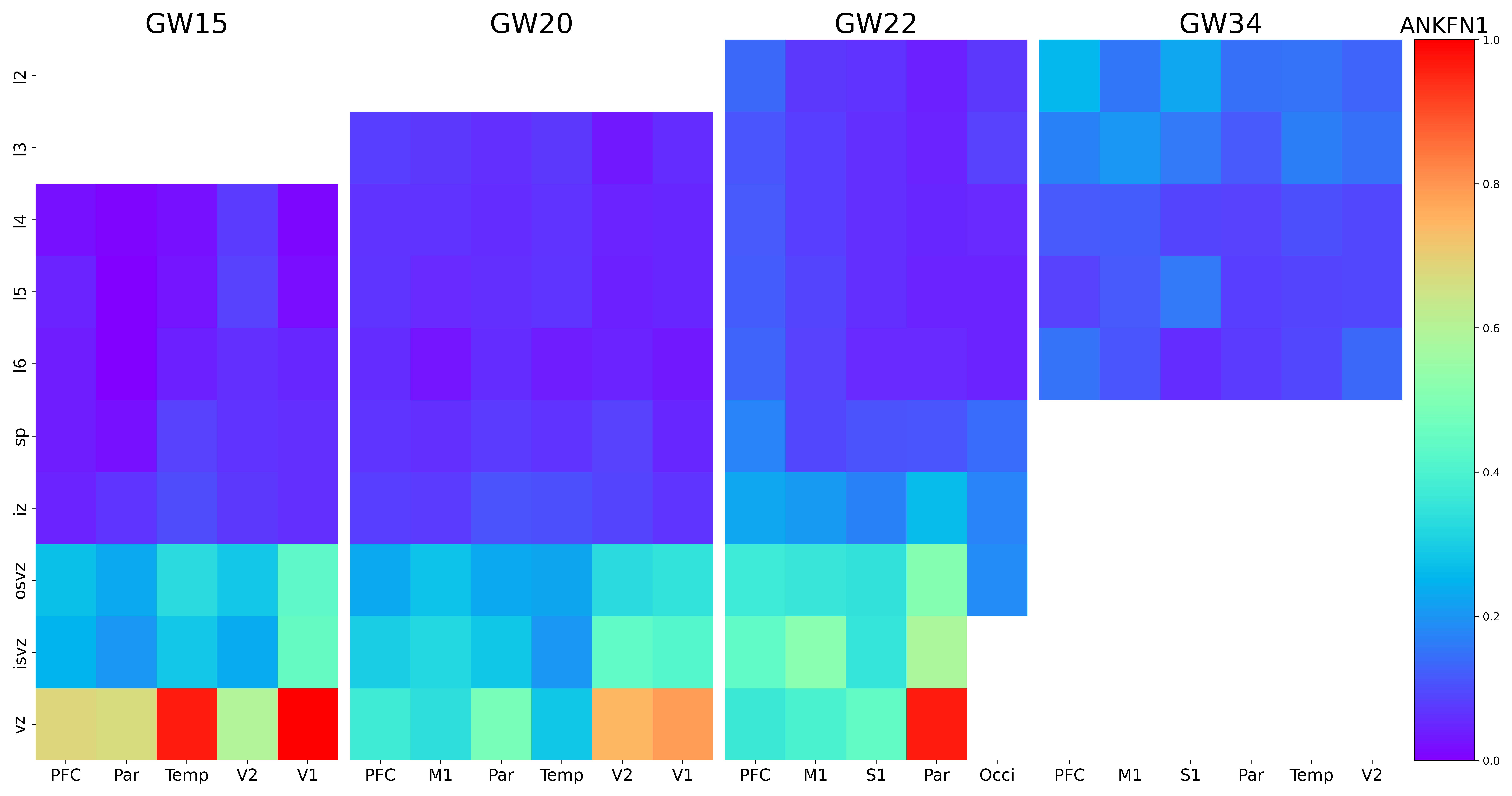

Supplement: Supplementary file 4 — Source Data Fig. 3: Expression pattern heatmap for all 300 genes in the MERFISH. [file 41586_2025_9010_MOESM4_ESM.zip › ANKFN1.png]

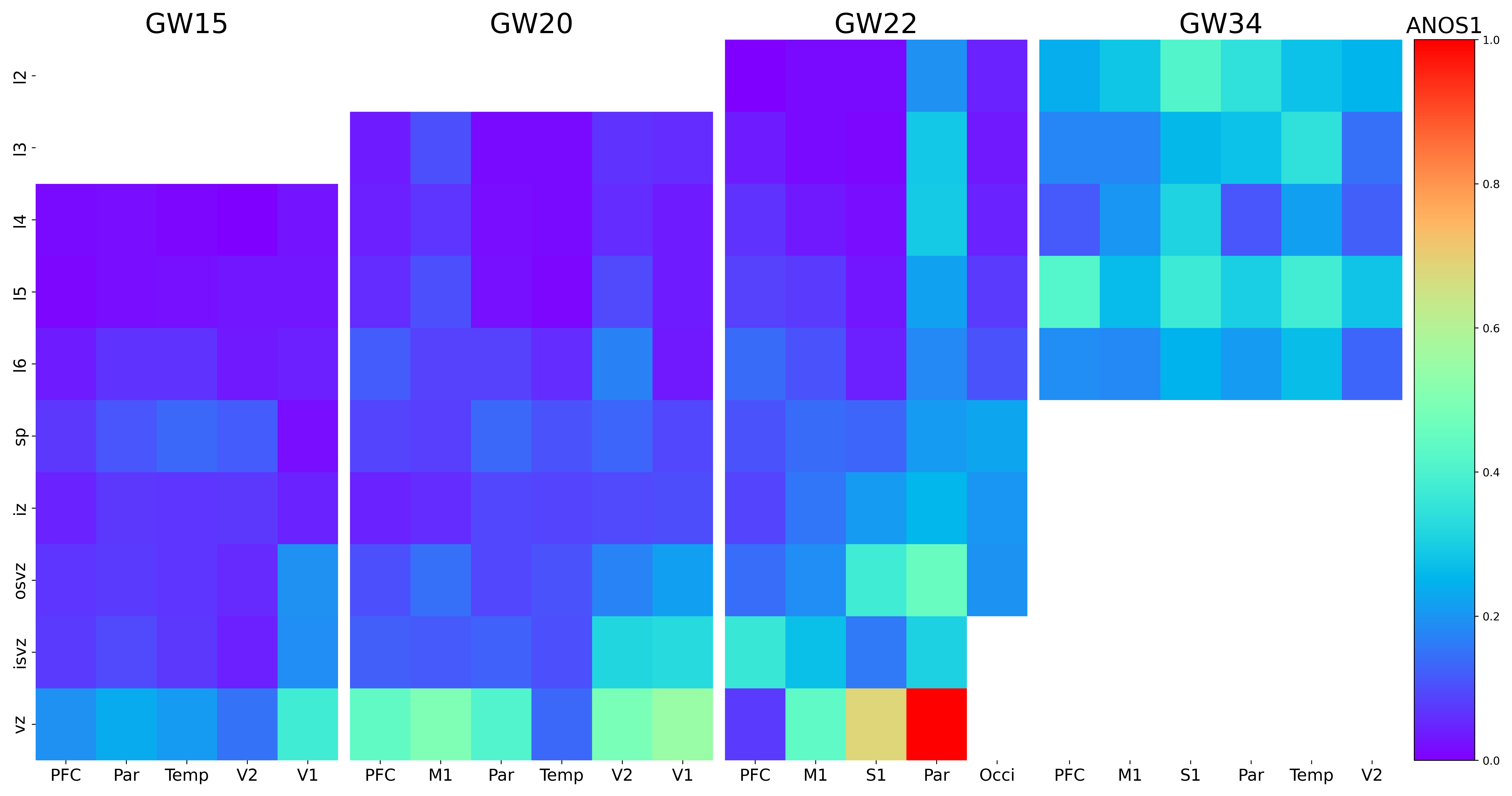

Supplement: Supplementary file 4 — Source Data Fig. 3: Expression pattern heatmap for all 300 genes in the MERFISH. [file 41586_2025_9010_MOESM4_ESM.zip › ANOS1.png]

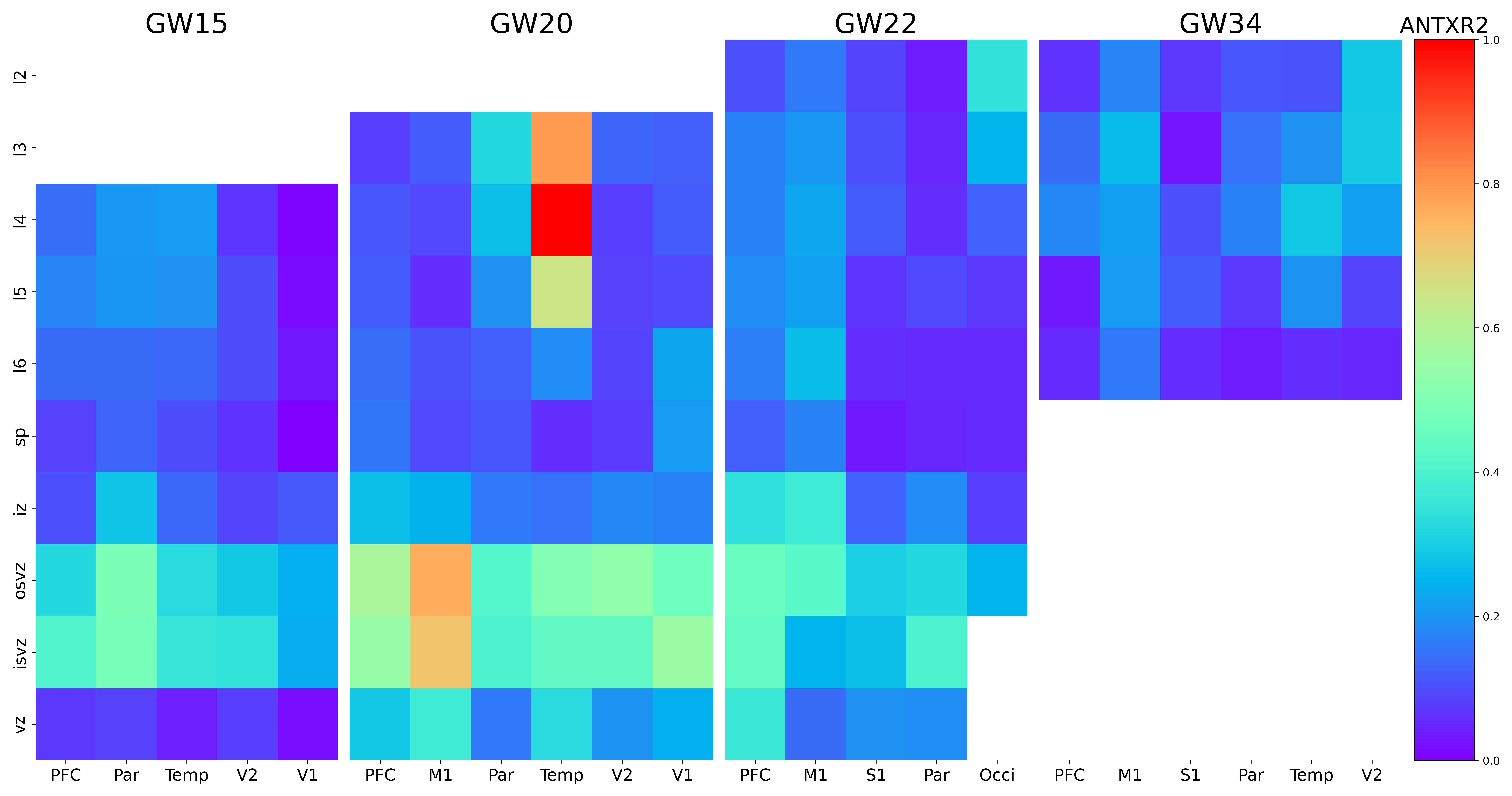

Supplement: Supplementary file 4 — Source Data Fig. 3: Expression pattern heatmap for all 300 genes in the MERFISH. [file 41586_2025_9010_MOESM4_ESM.zip › ANTXR2.png]

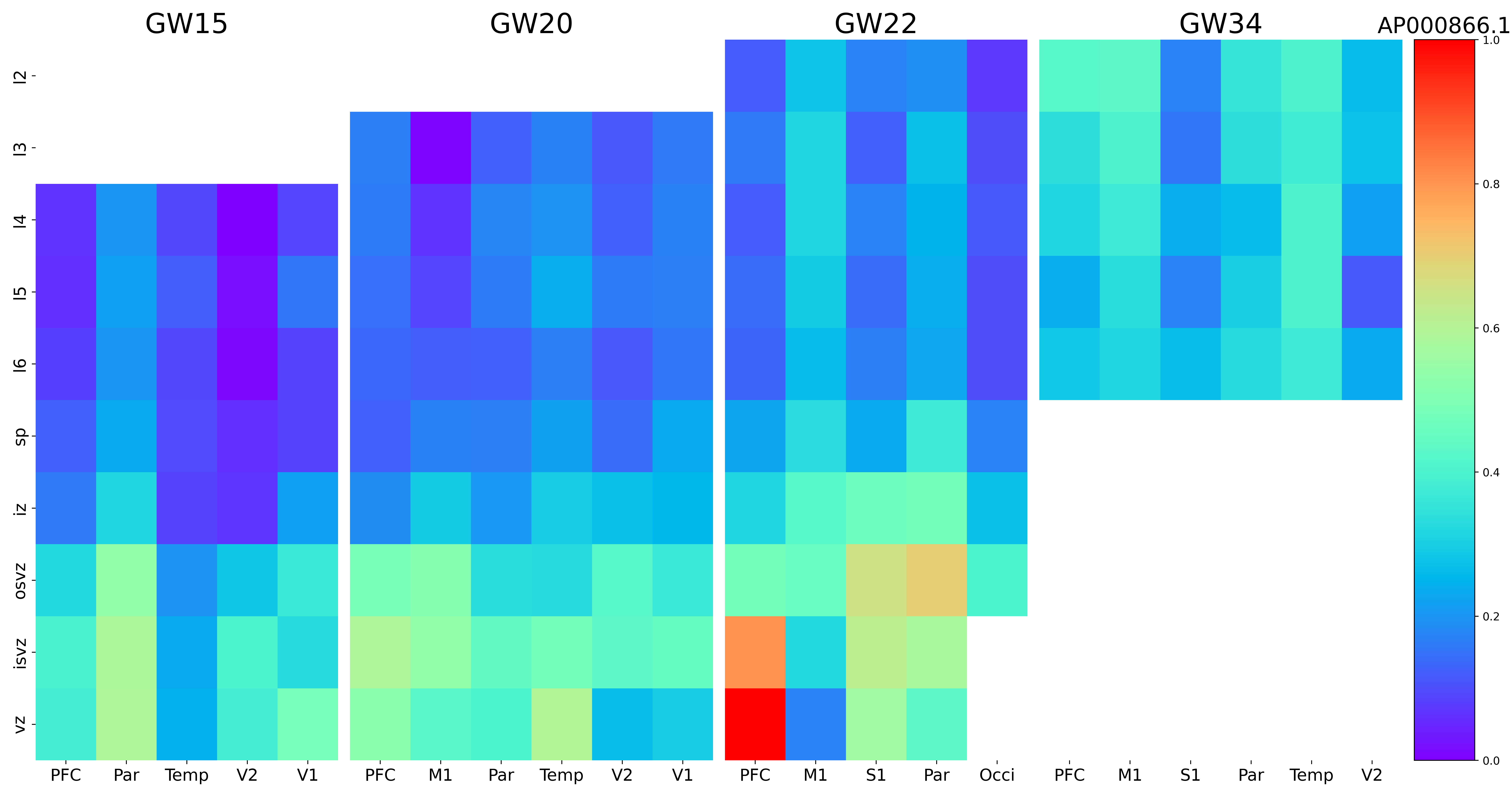

Supplement: Supplementary file 4 — Source Data Fig. 3: Expression pattern heatmap for all 300 genes in the MERFISH. [file 41586_2025_9010_MOESM4_ESM.zip › AP000866.1.png]

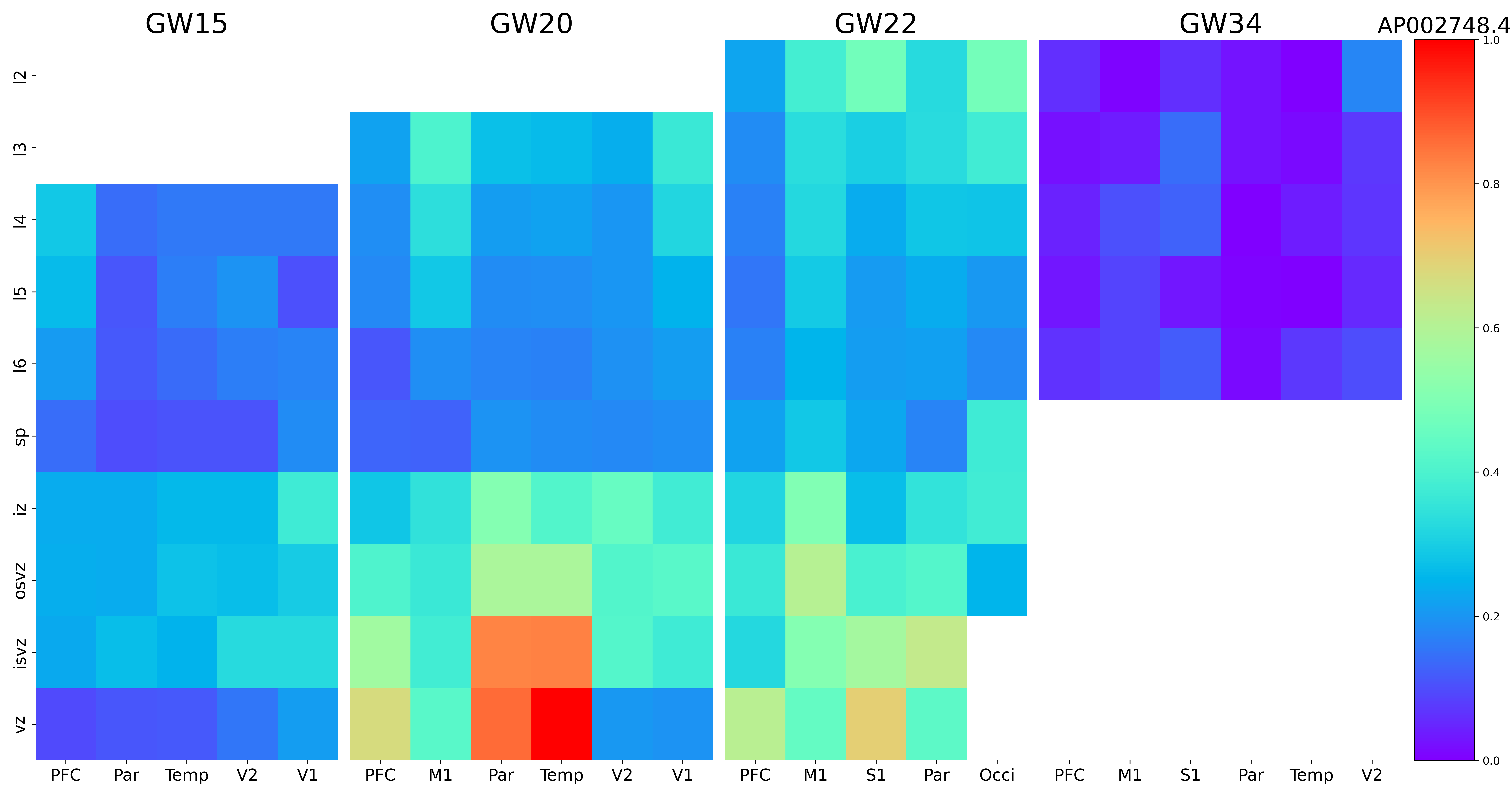

Supplement: Supplementary file 4 — Source Data Fig. 3: Expression pattern heatmap for all 300 genes in the MERFISH. [file 41586_2025_9010_MOESM4_ESM.zip › AP002748.4.png]

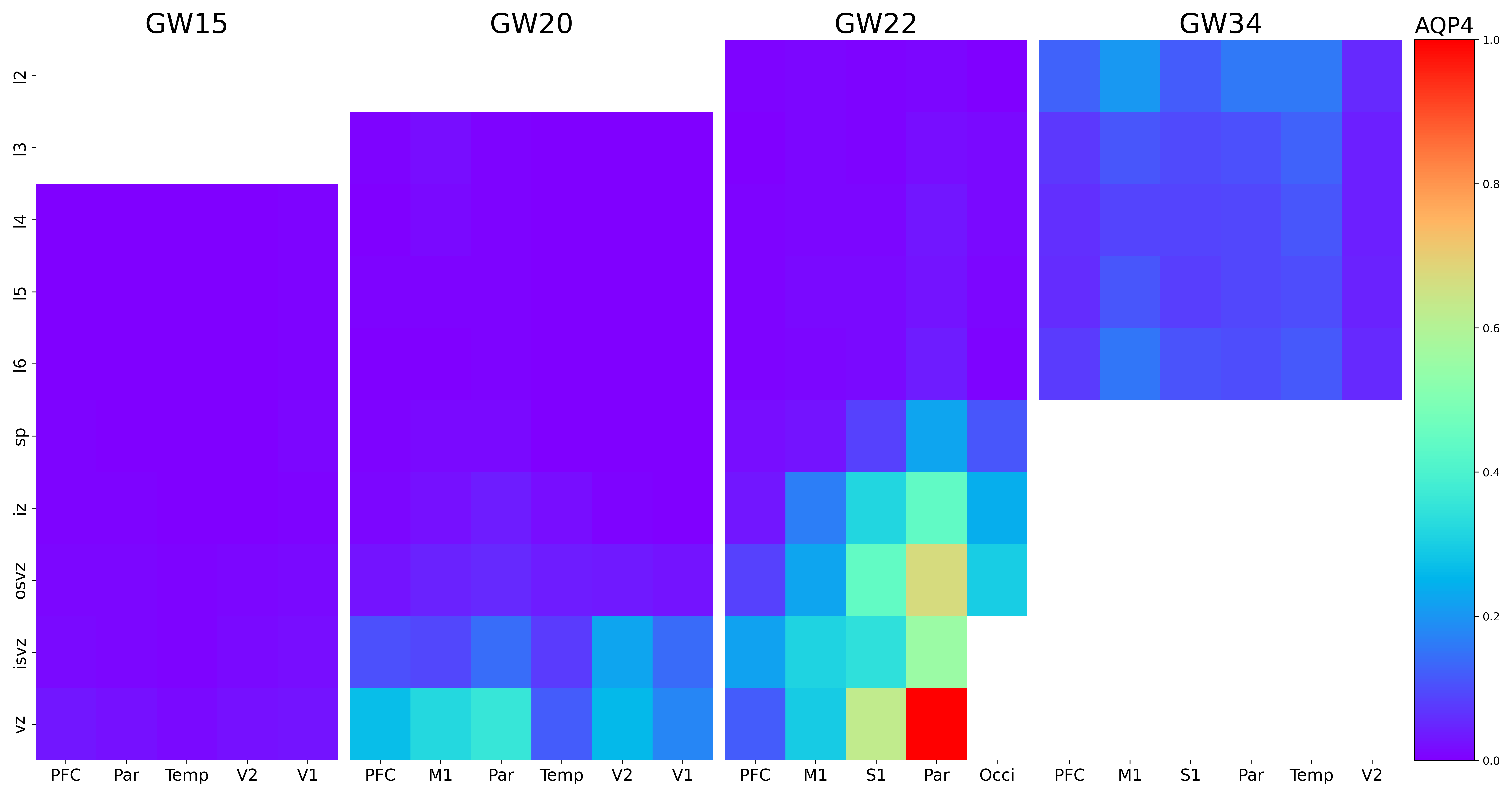

Supplement: Supplementary file 4 — Source Data Fig. 3: Expression pattern heatmap for all 300 genes in the MERFISH. [file 41586_2025_9010_MOESM4_ESM.zip › AQP4.png]

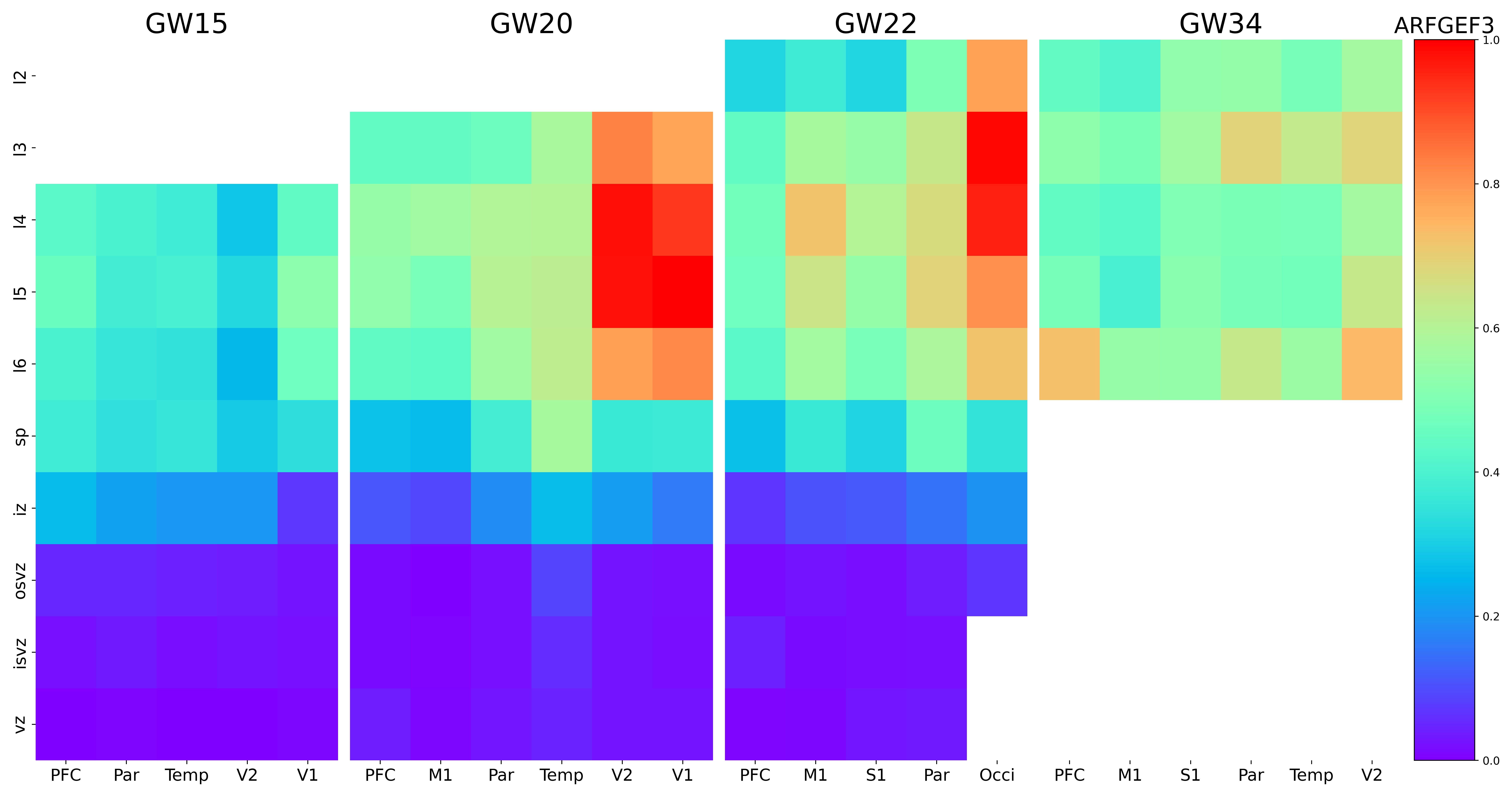

Supplement: Supplementary file 4 — Source Data Fig. 3: Expression pattern heatmap for all 300 genes in the MERFISH. [file 41586_2025_9010_MOESM4_ESM.zip › ARFGEF3.png]

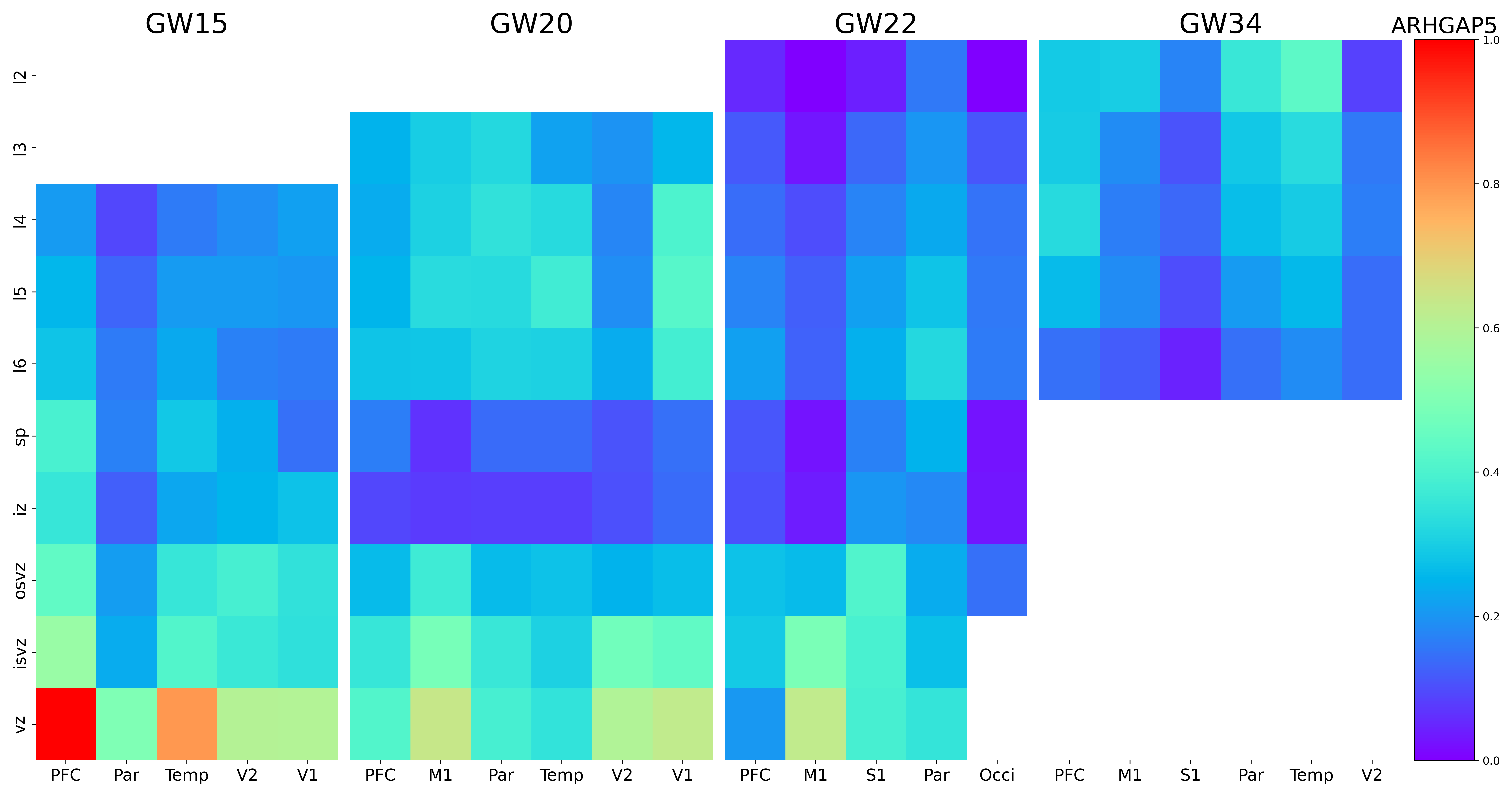

Supplement: Supplementary file 4 — Source Data Fig. 3: Expression pattern heatmap for all 300 genes in the MERFISH. [file 41586_2025_9010_MOESM4_ESM.zip › ARHGAP5.png]

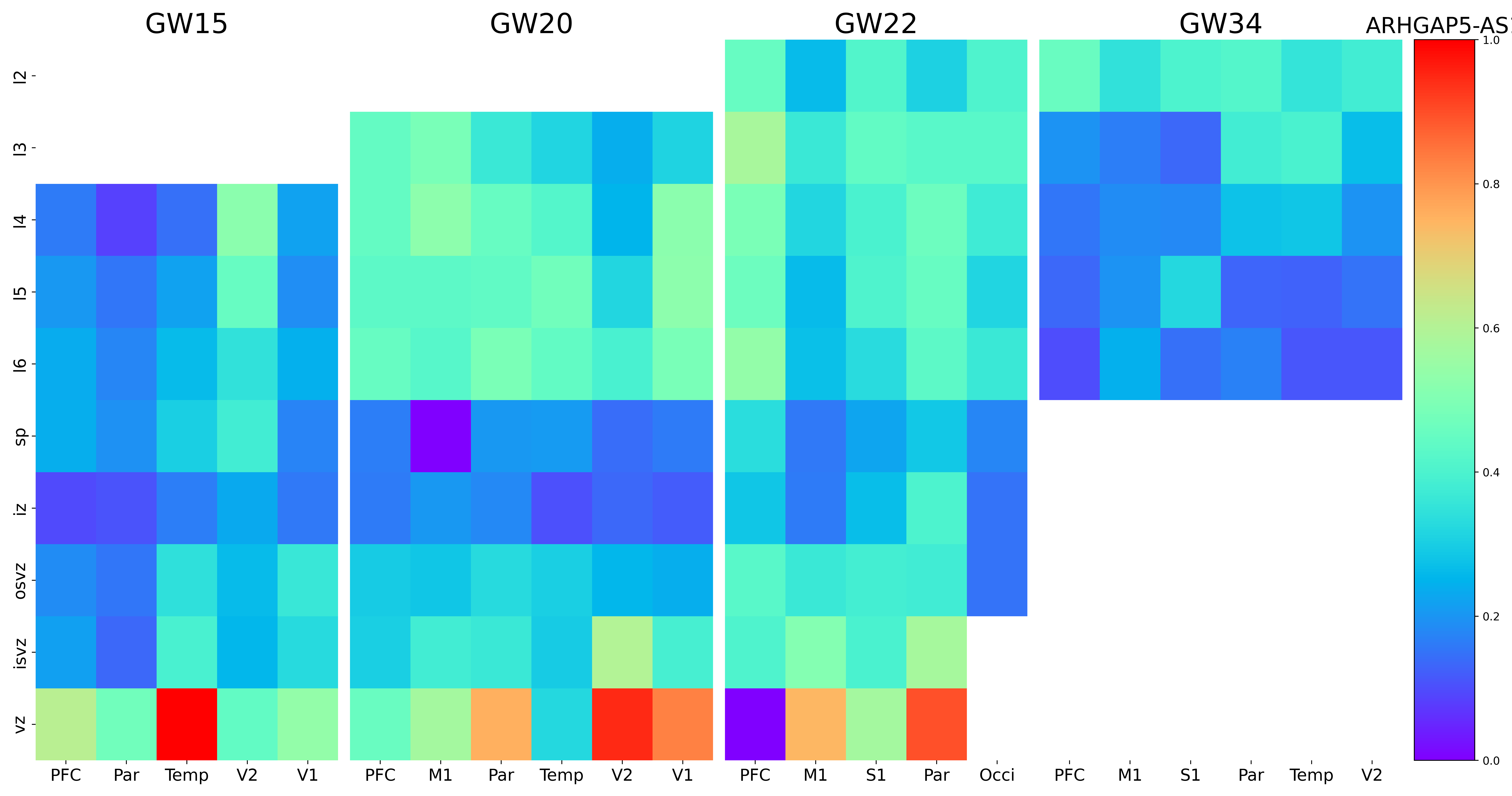

Supplement: Supplementary file 4 — Source Data Fig. 3: Expression pattern heatmap for all 300 genes in the MERFISH. [file 41586_2025_9010_MOESM4_ESM.zip › ARHGAP5-AS1.png]

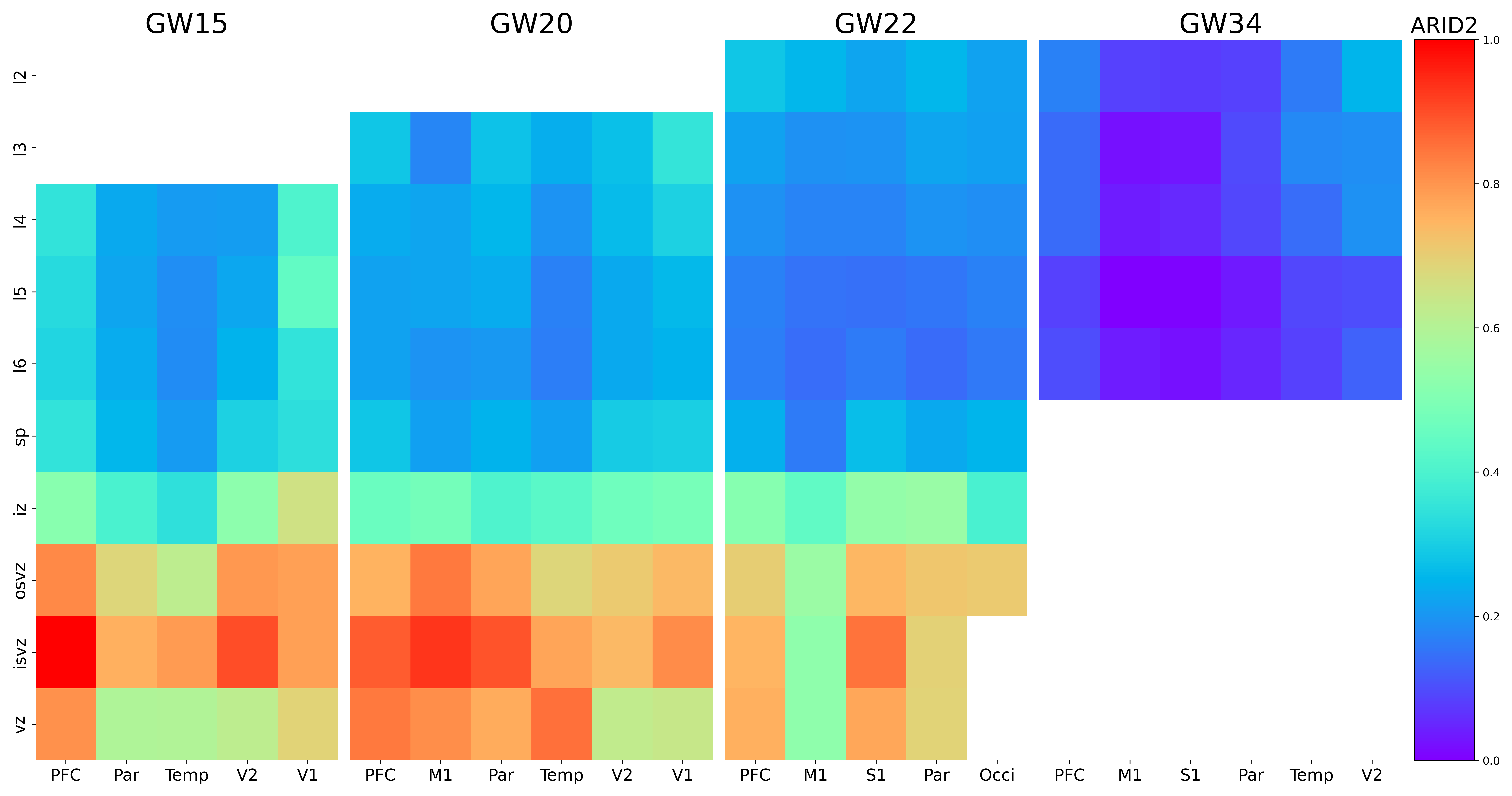

Supplement: Supplementary file 4 — Source Data Fig. 3: Expression pattern heatmap for all 300 genes in the MERFISH. [file 41586_2025_9010_MOESM4_ESM.zip › ARID2.png]

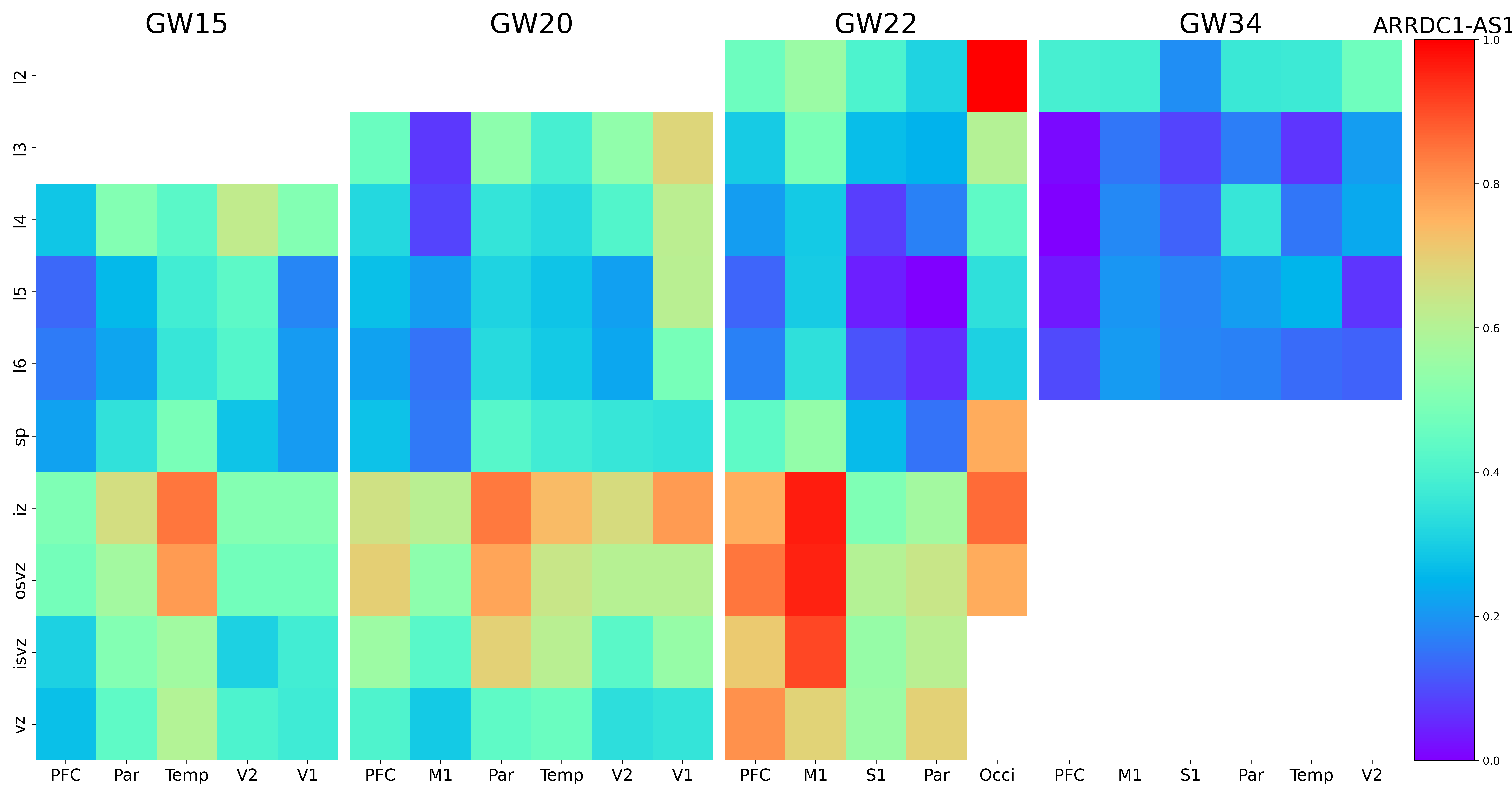

Supplement: Supplementary file 4 — Source Data Fig. 3: Expression pattern heatmap for all 300 genes in the MERFISH. [file 41586_2025_9010_MOESM4_ESM.zip › ARRDC1-AS1.png]

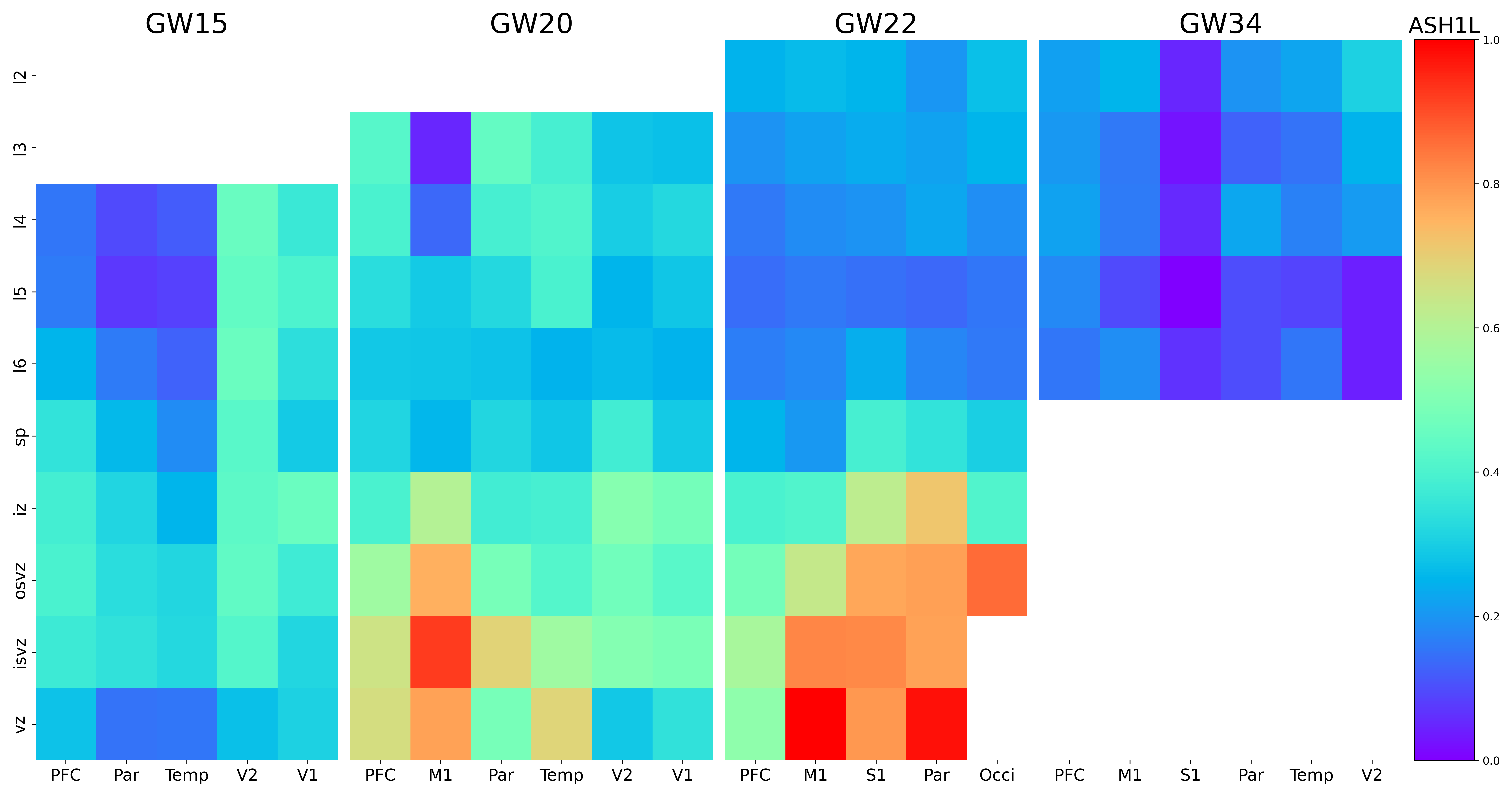

Supplement: Supplementary file 4 — Source Data Fig. 3: Expression pattern heatmap for all 300 genes in the MERFISH. [file 41586_2025_9010_MOESM4_ESM.zip › ASH1L.png]

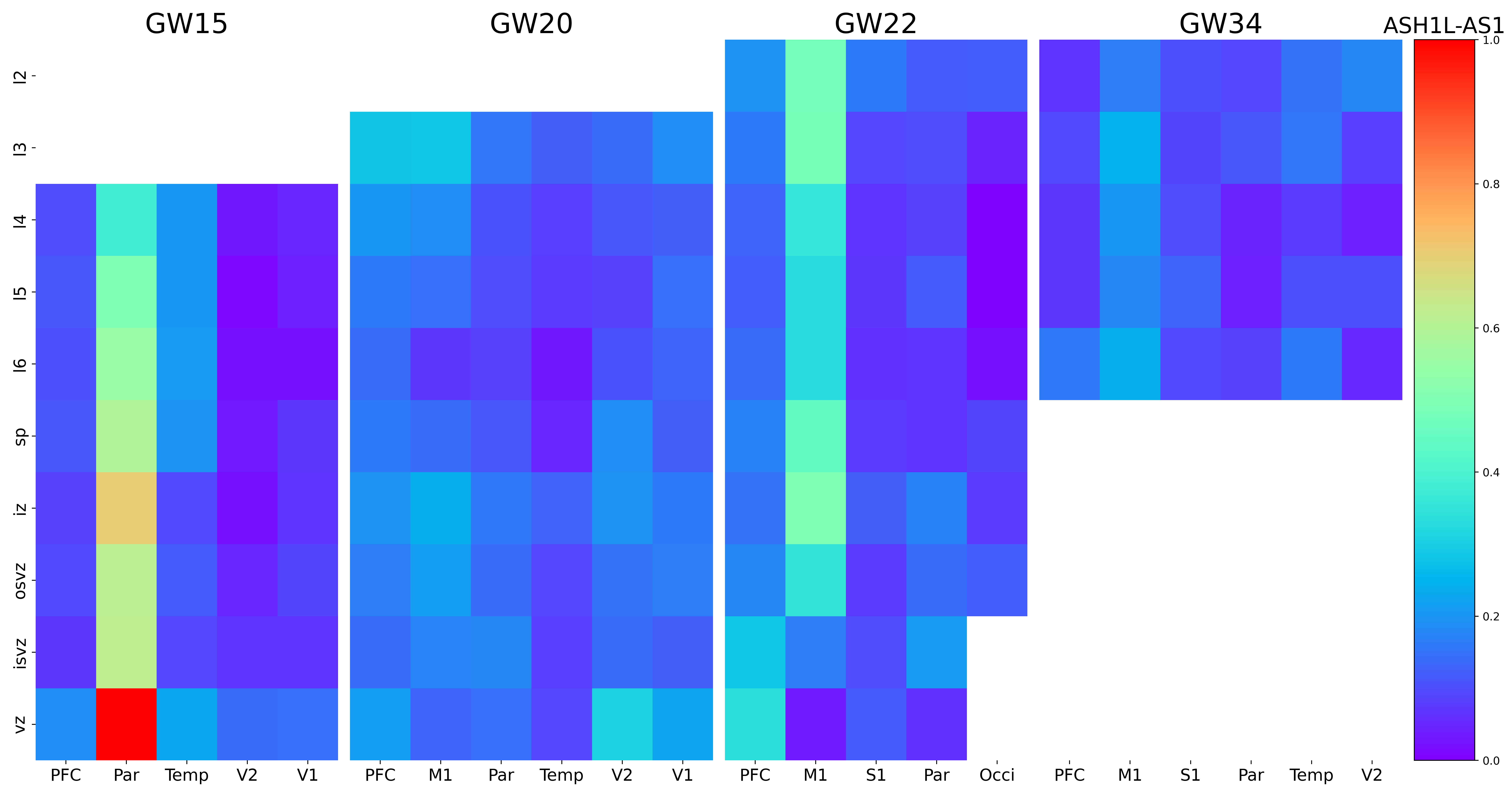

Supplement: Supplementary file 4 — Source Data Fig. 3: Expression pattern heatmap for all 300 genes in the MERFISH. [file 41586_2025_9010_MOESM4_ESM.zip › ASH1L-AS1.png]

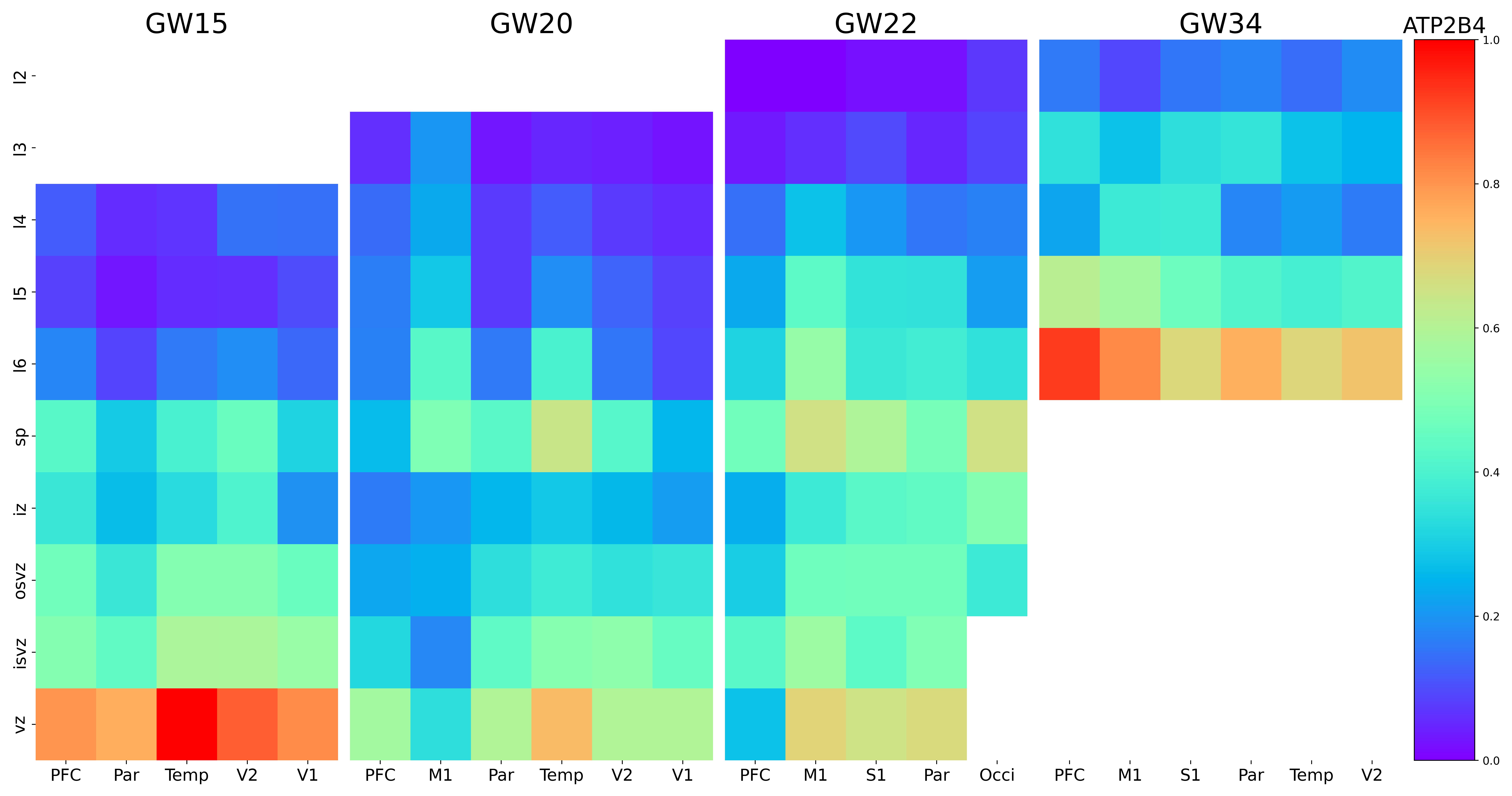

Supplement: Supplementary file 4 — Source Data Fig. 3: Expression pattern heatmap for all 300 genes in the MERFISH. [file 41586_2025_9010_MOESM4_ESM.zip › ATP2B4.png]

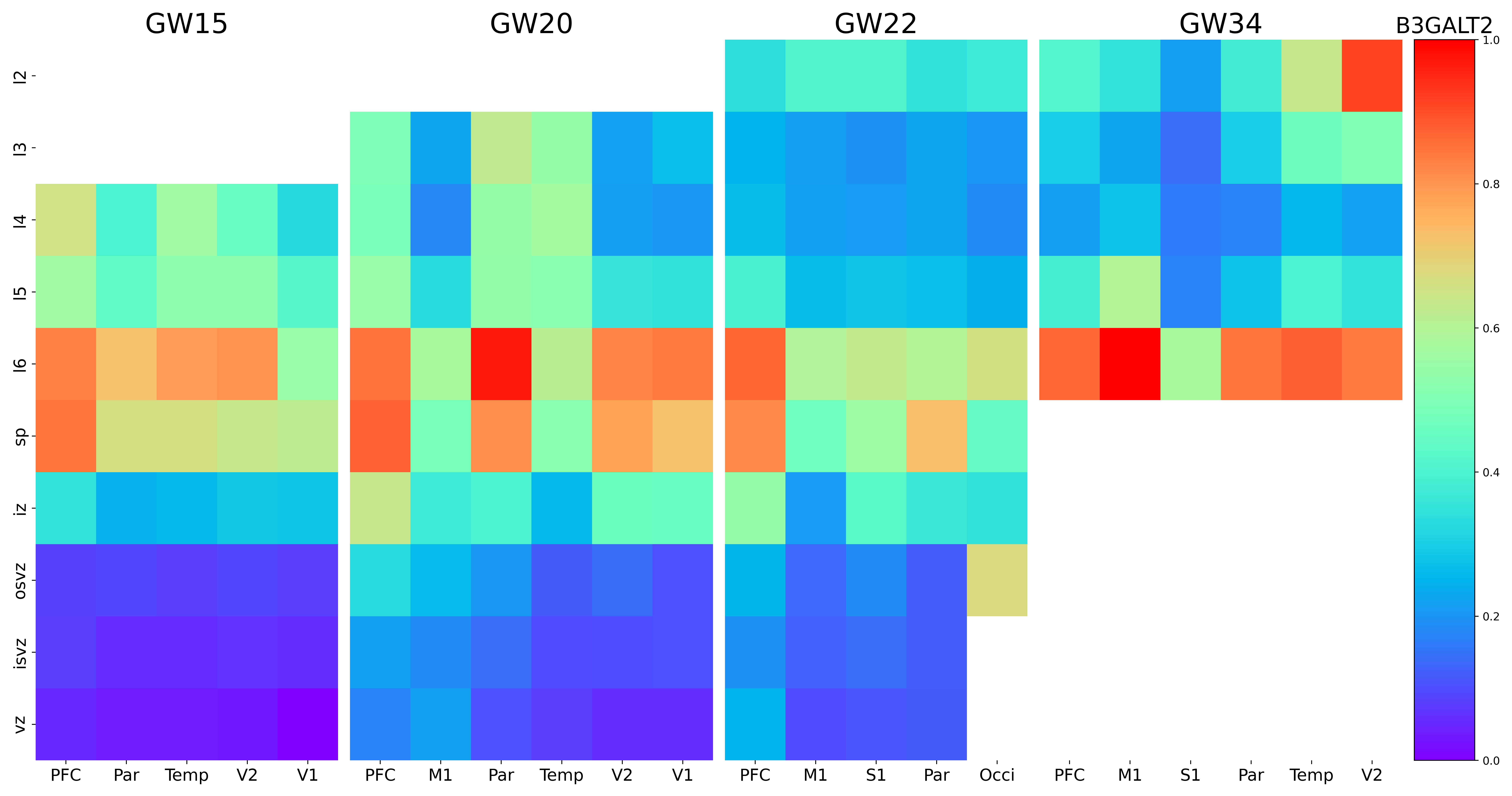

Supplement: Supplementary file 4 — Source Data Fig. 3: Expression pattern heatmap for all 300 genes in the MERFISH. [file 41586_2025_9010_MOESM4_ESM.zip › B3GALT2.png]

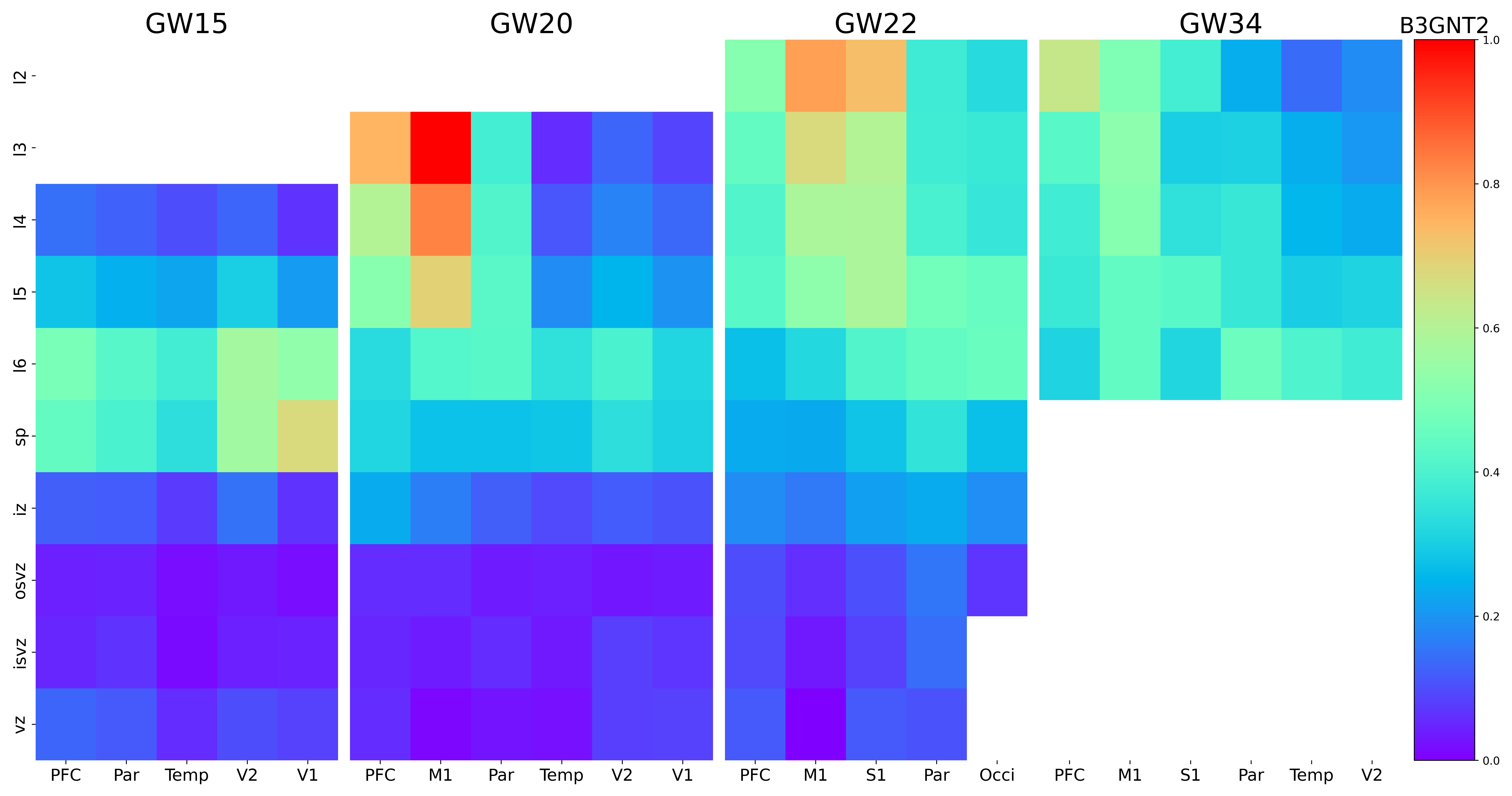

Supplement: Supplementary file 4 — Source Data Fig. 3: Expression pattern heatmap for all 300 genes in the MERFISH. [file 41586_2025_9010_MOESM4_ESM.zip › B3GNT2.png]

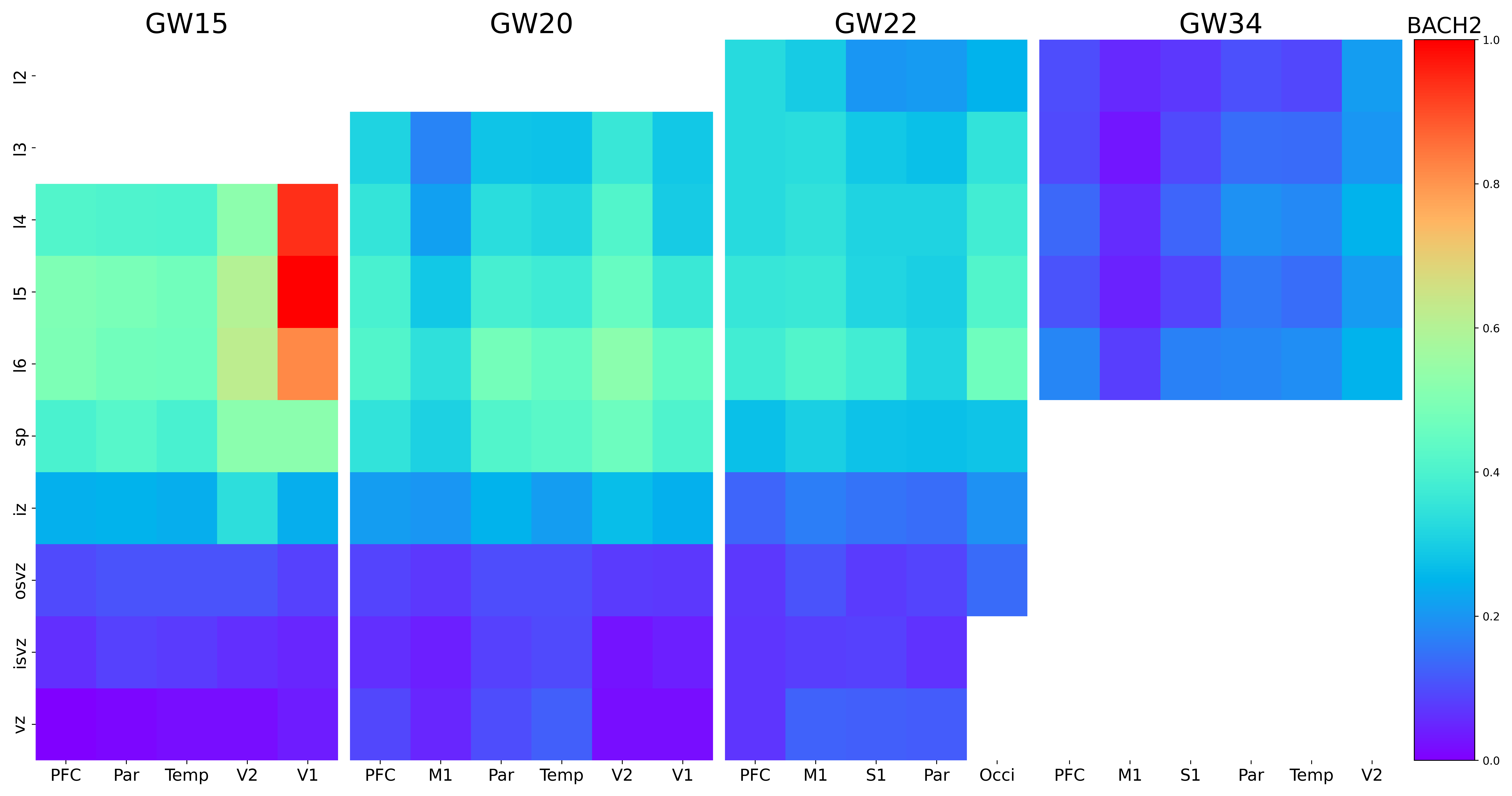

Supplement: Supplementary file 4 — Source Data Fig. 3: Expression pattern heatmap for all 300 genes in the MERFISH. [file 41586_2025_9010_MOESM4_ESM.zip › BACH2.png]

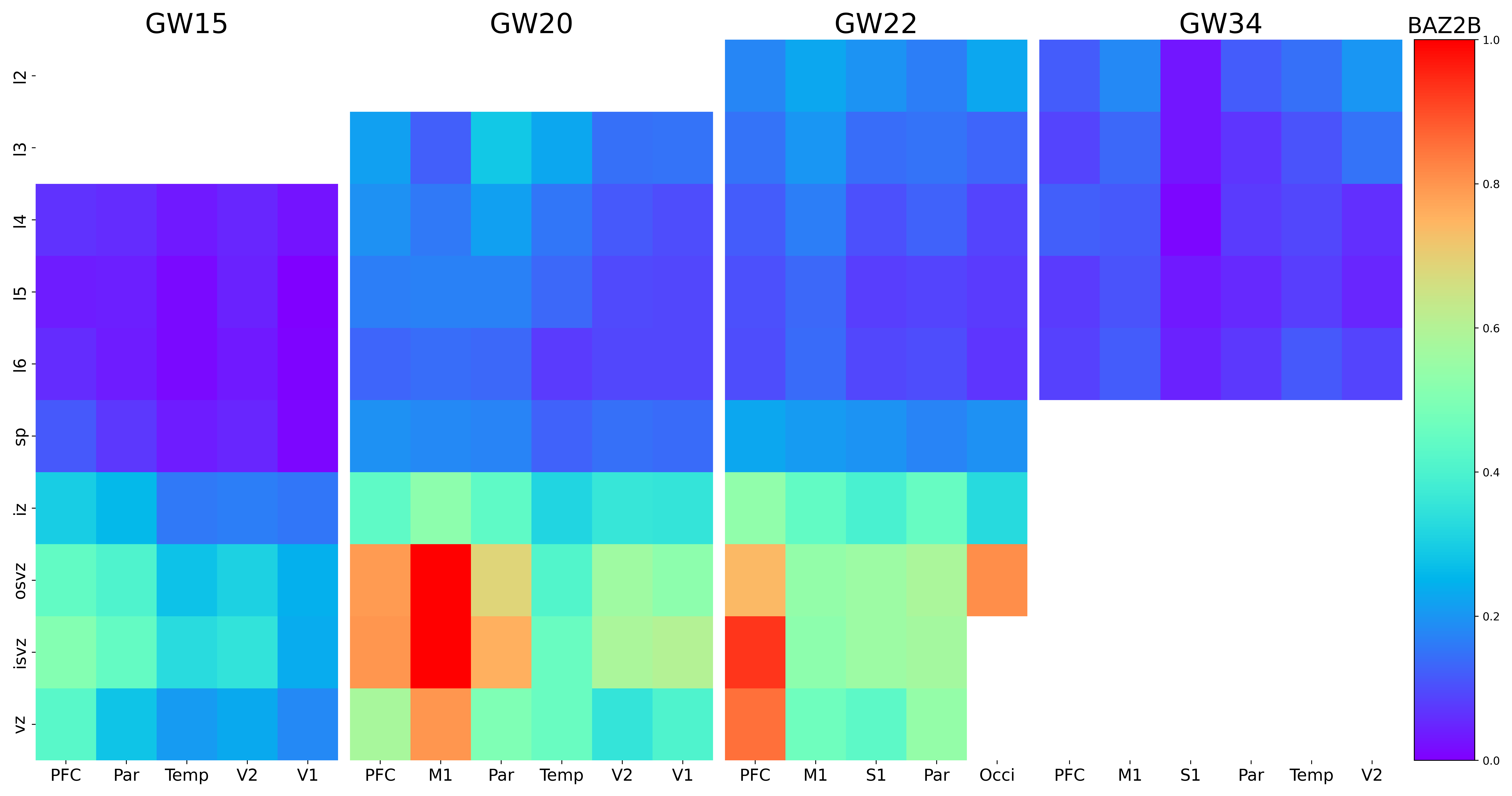

Supplement: Supplementary file 4 — Source Data Fig. 3: Expression pattern heatmap for all 300 genes in the MERFISH. [file 41586_2025_9010_MOESM4_ESM.zip › BAZ2B.png]

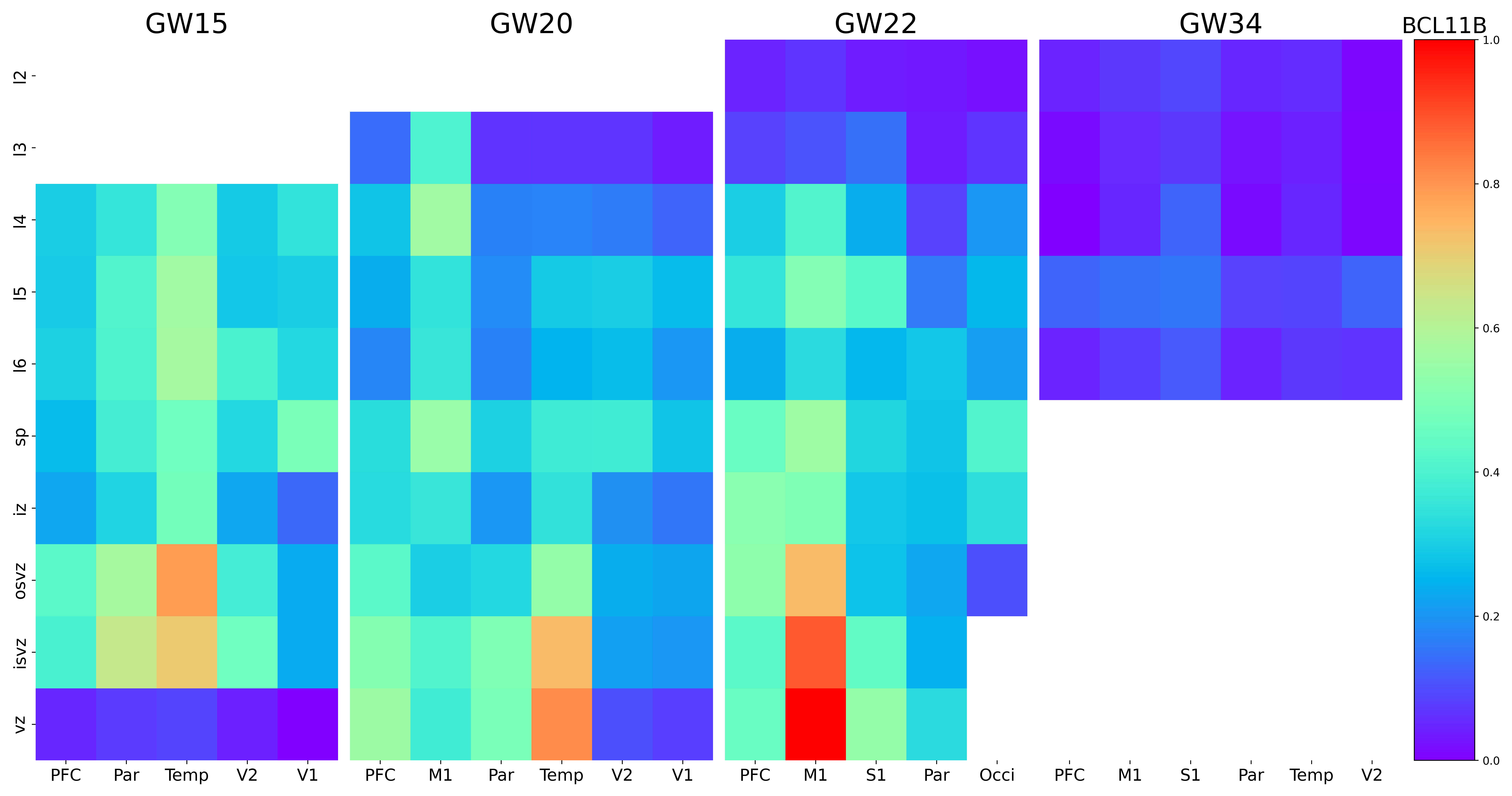

Supplement: Supplementary file 4 — Source Data Fig. 3: Expression pattern heatmap for all 300 genes in the MERFISH. [file 41586_2025_9010_MOESM4_ESM.zip › BCL11B.png]

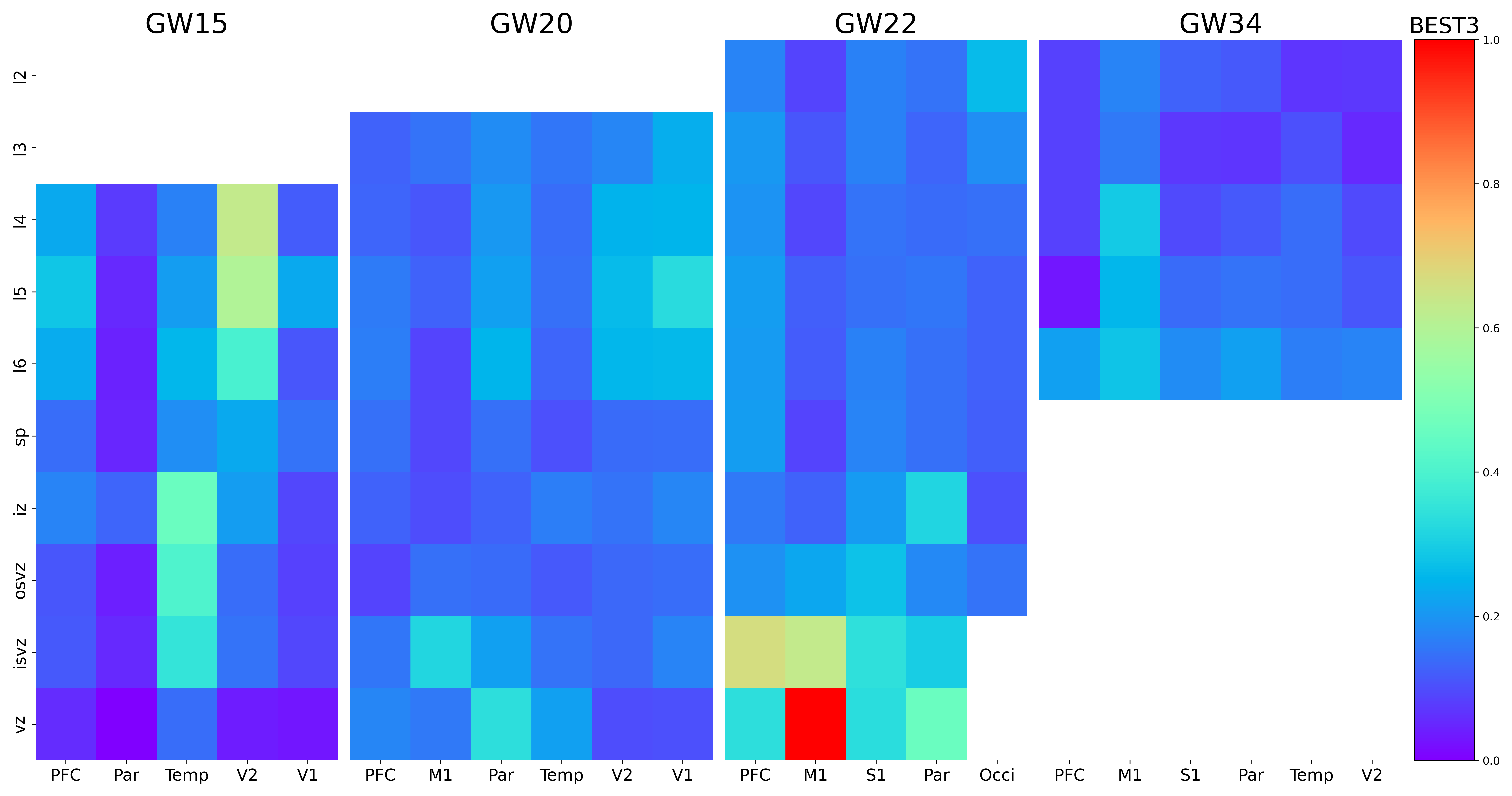

Supplement: Supplementary file 4 — Source Data Fig. 3: Expression pattern heatmap for all 300 genes in the MERFISH. [file 41586_2025_9010_MOESM4_ESM.zip › BEST3.png]

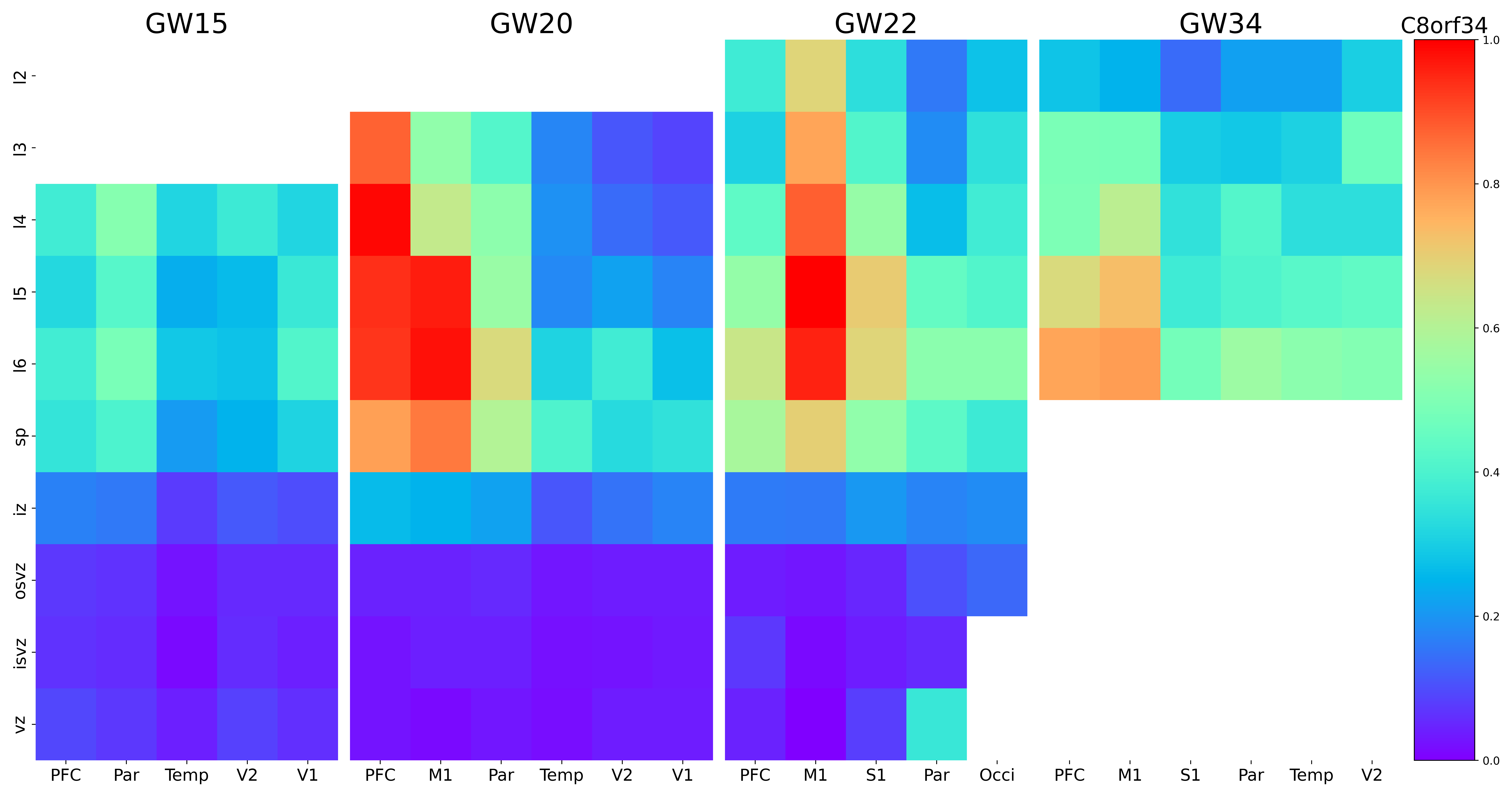

Supplement: Supplementary file 4 — Source Data Fig. 3: Expression pattern heatmap for all 300 genes in the MERFISH. [file 41586_2025_9010_MOESM4_ESM.zip › C8orf34.png]

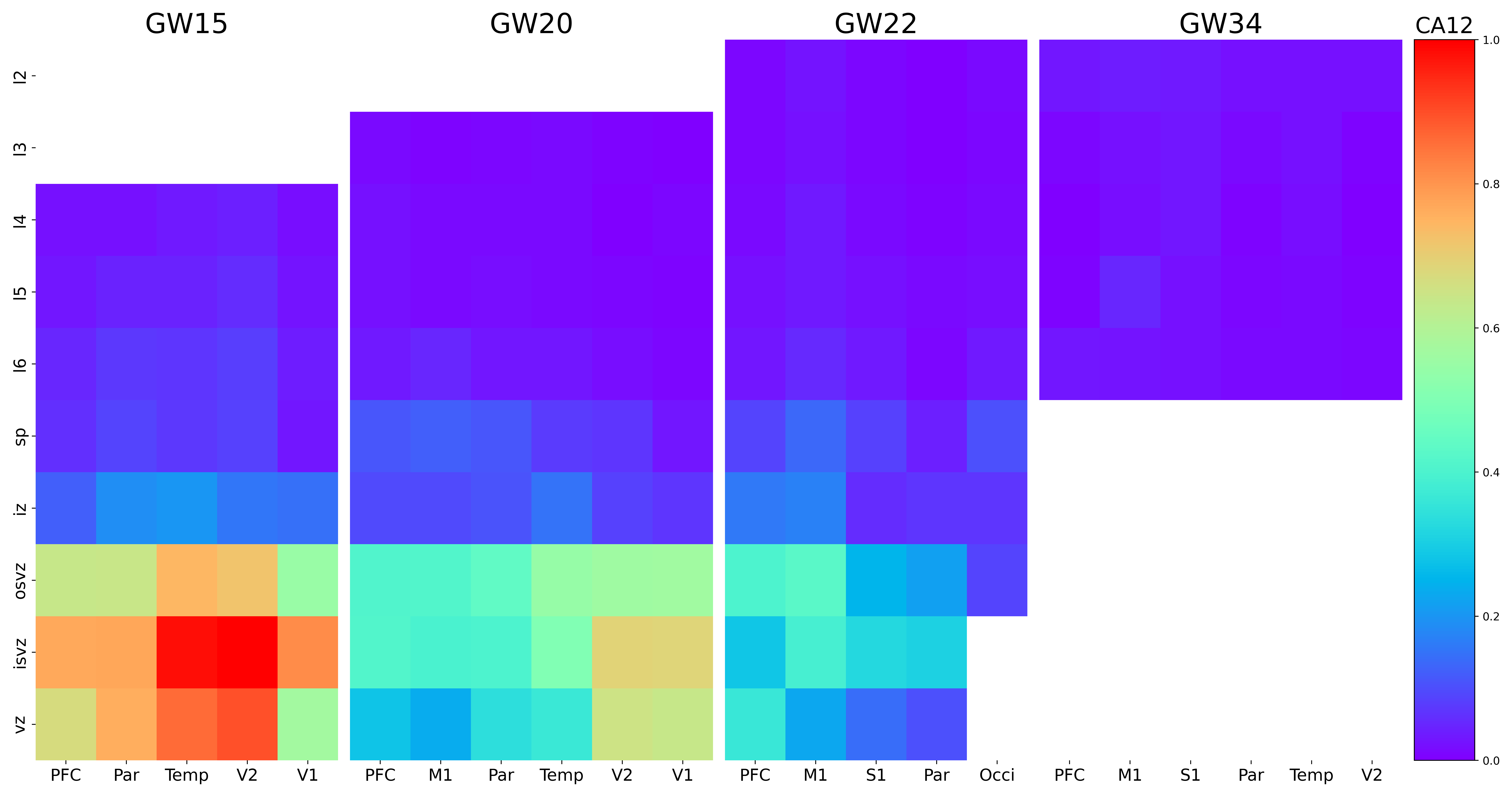

Supplement: Supplementary file 4 — Source Data Fig. 3: Expression pattern heatmap for all 300 genes in the MERFISH. [file 41586_2025_9010_MOESM4_ESM.zip › CA12.png]

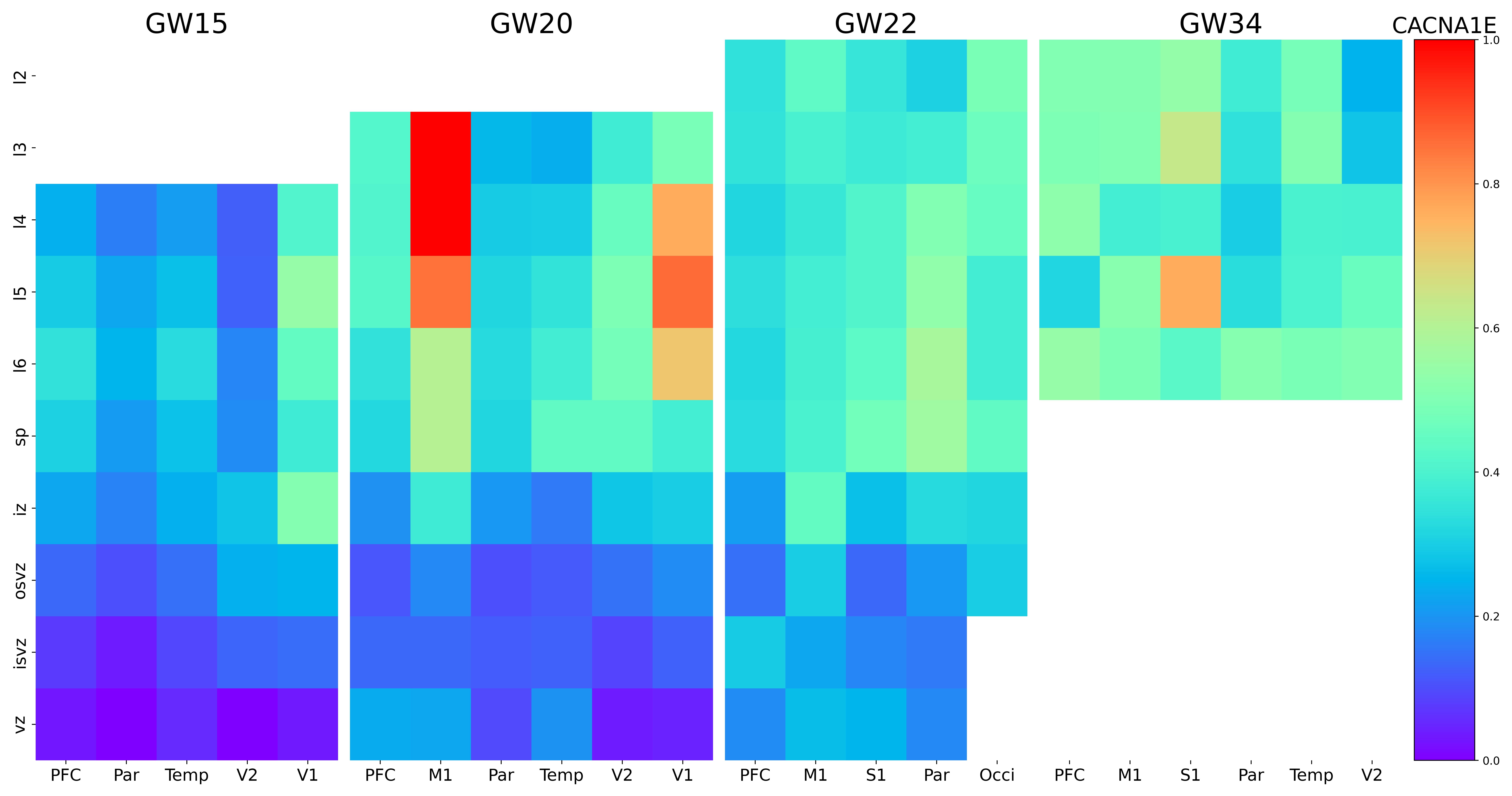

Supplement: Supplementary file 4 — Source Data Fig. 3: Expression pattern heatmap for all 300 genes in the MERFISH. [file 41586_2025_9010_MOESM4_ESM.zip › CACNA1E.png]

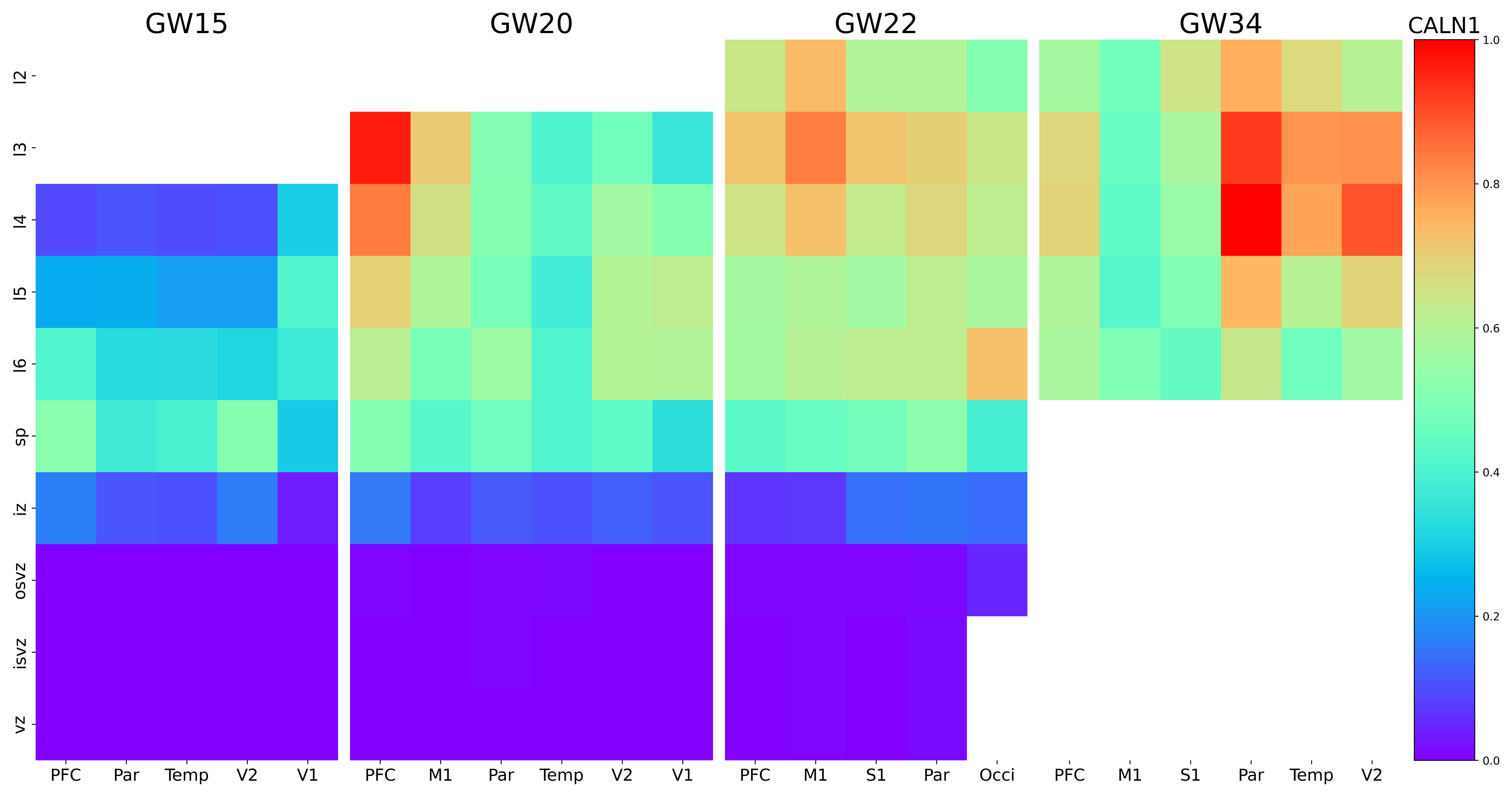

Supplement: Supplementary file 4 — Source Data Fig. 3: Expression pattern heatmap for all 300 genes in the MERFISH. [file 41586_2025_9010_MOESM4_ESM.zip › CALN1.png]

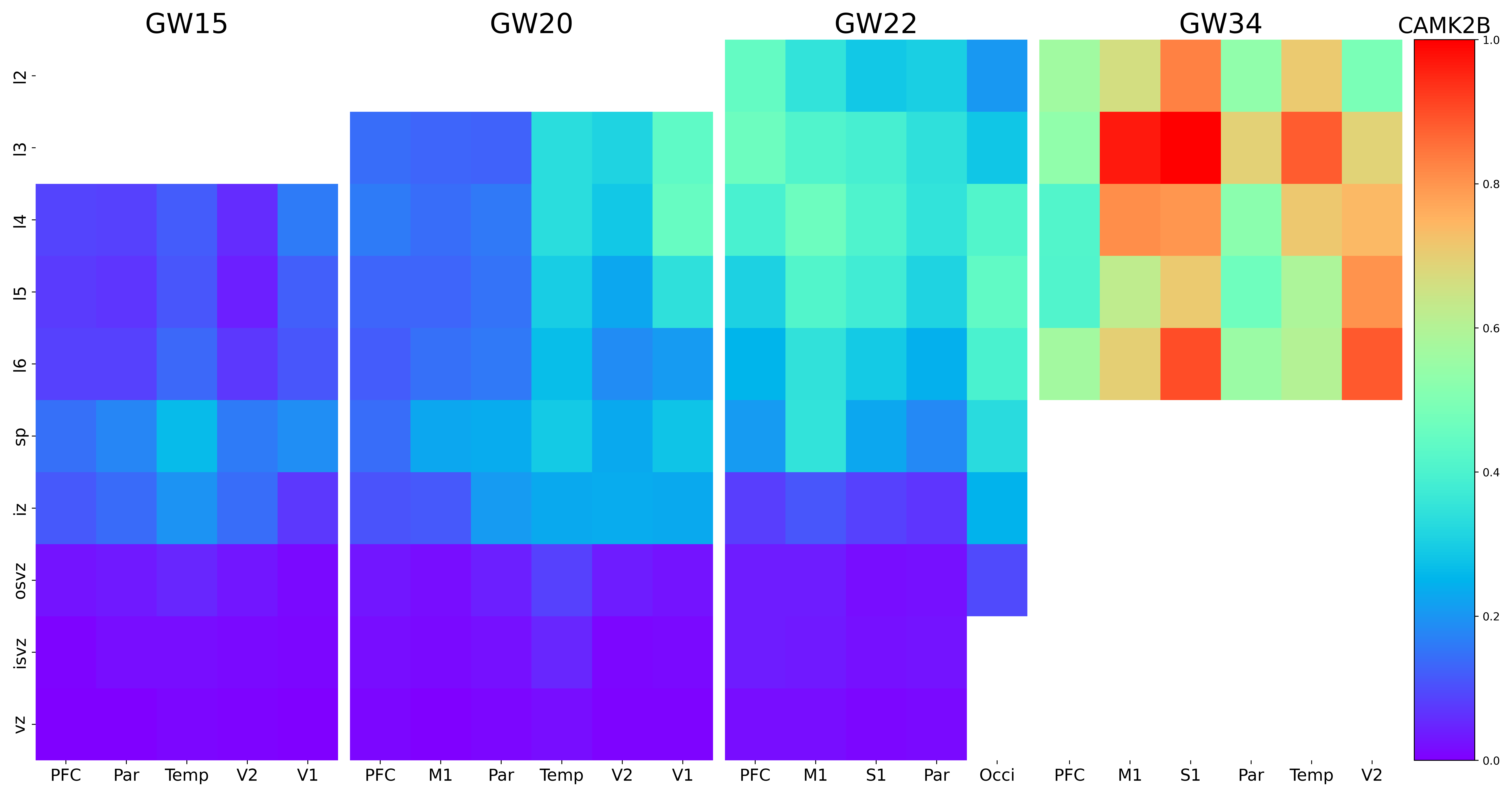

Supplement: Supplementary file 4 — Source Data Fig. 3: Expression pattern heatmap for all 300 genes in the MERFISH. [file 41586_2025_9010_MOESM4_ESM.zip › CAMK2B.png]

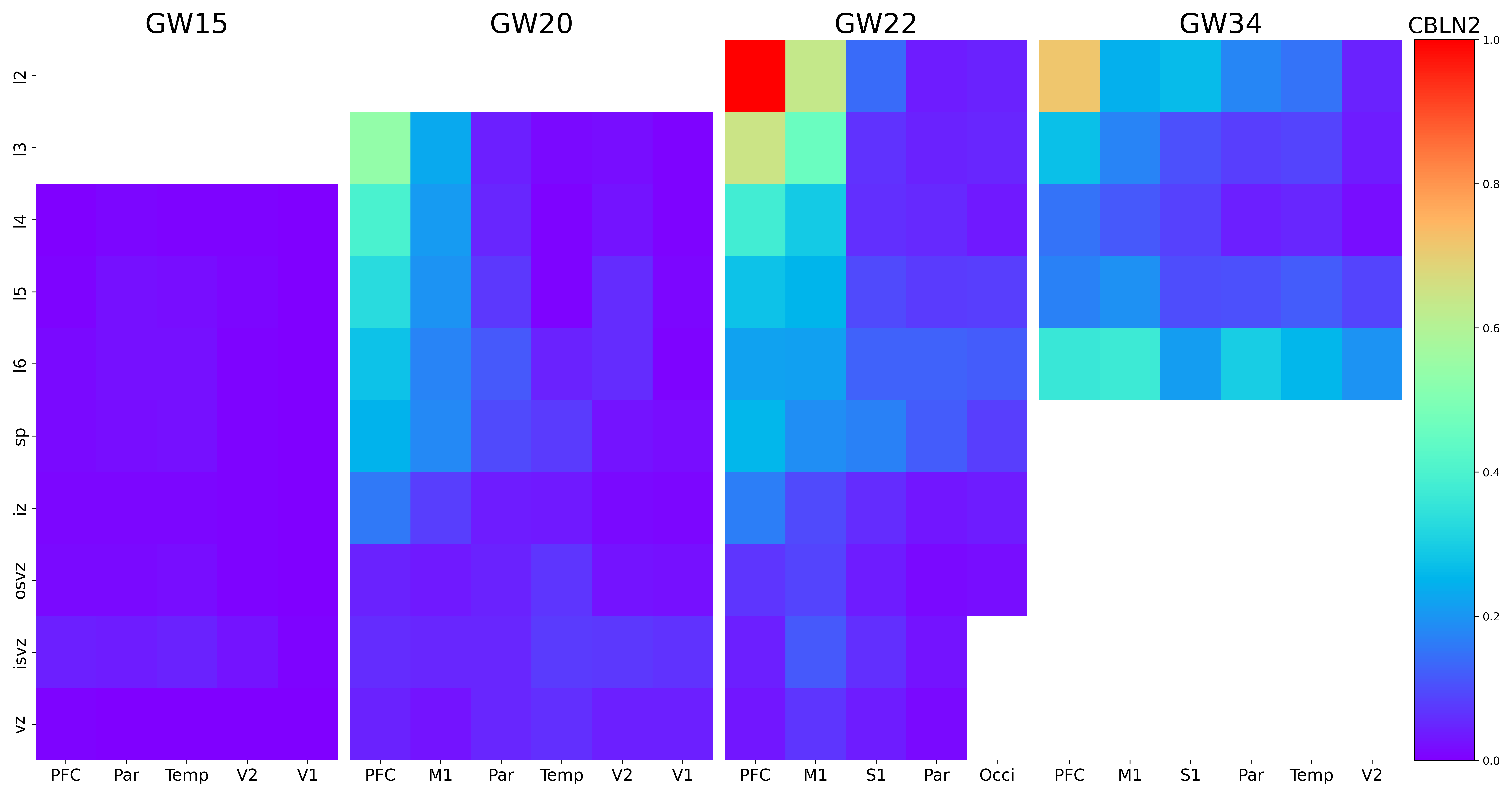

Supplement: Supplementary file 4 — Source Data Fig. 3: Expression pattern heatmap for all 300 genes in the MERFISH. [file 41586_2025_9010_MOESM4_ESM.zip › CBLN2.png]

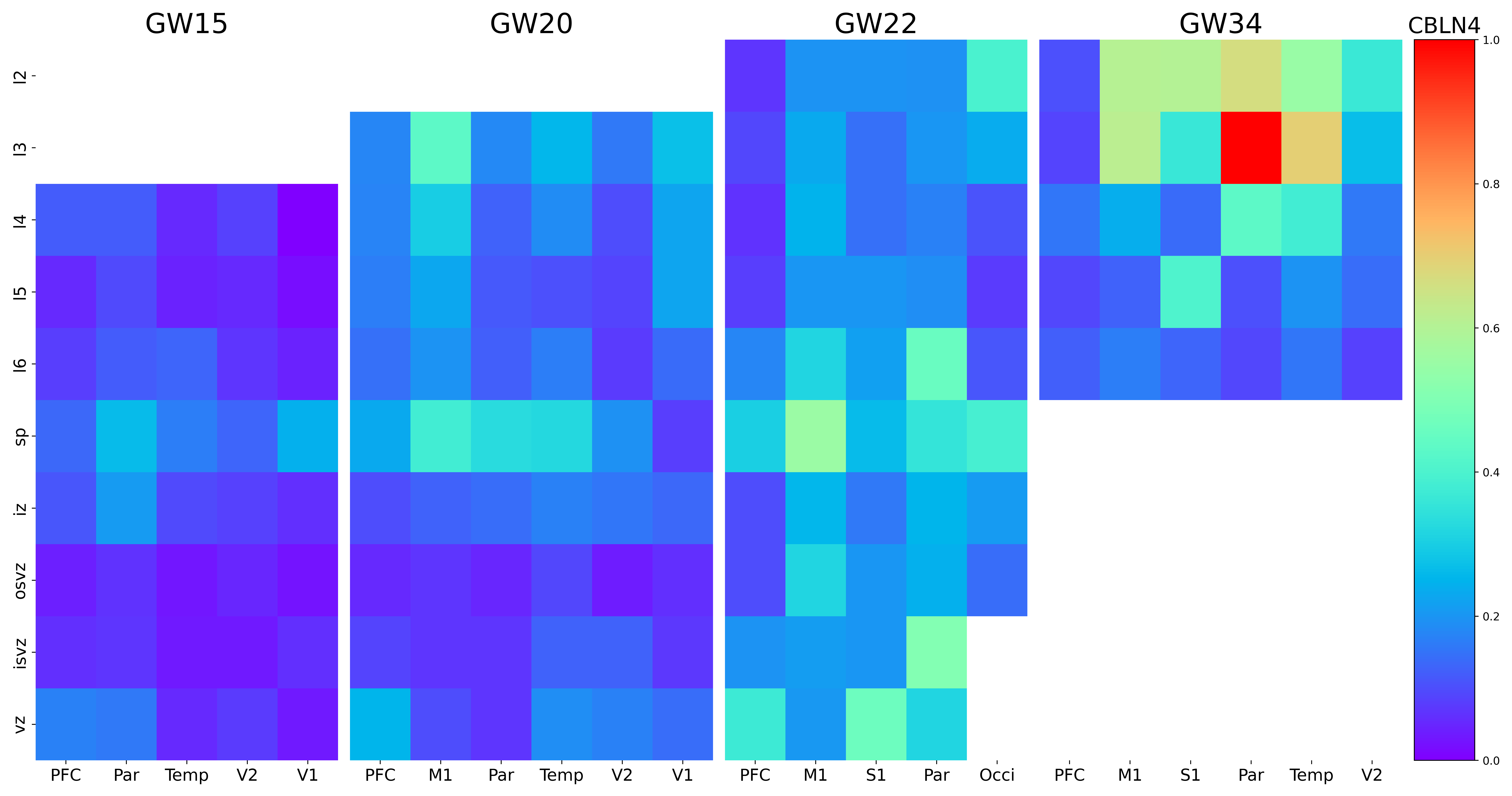

Supplement: Supplementary file 4 — Source Data Fig. 3: Expression pattern heatmap for all 300 genes in the MERFISH. [file 41586_2025_9010_MOESM4_ESM.zip › CBLN4.png]

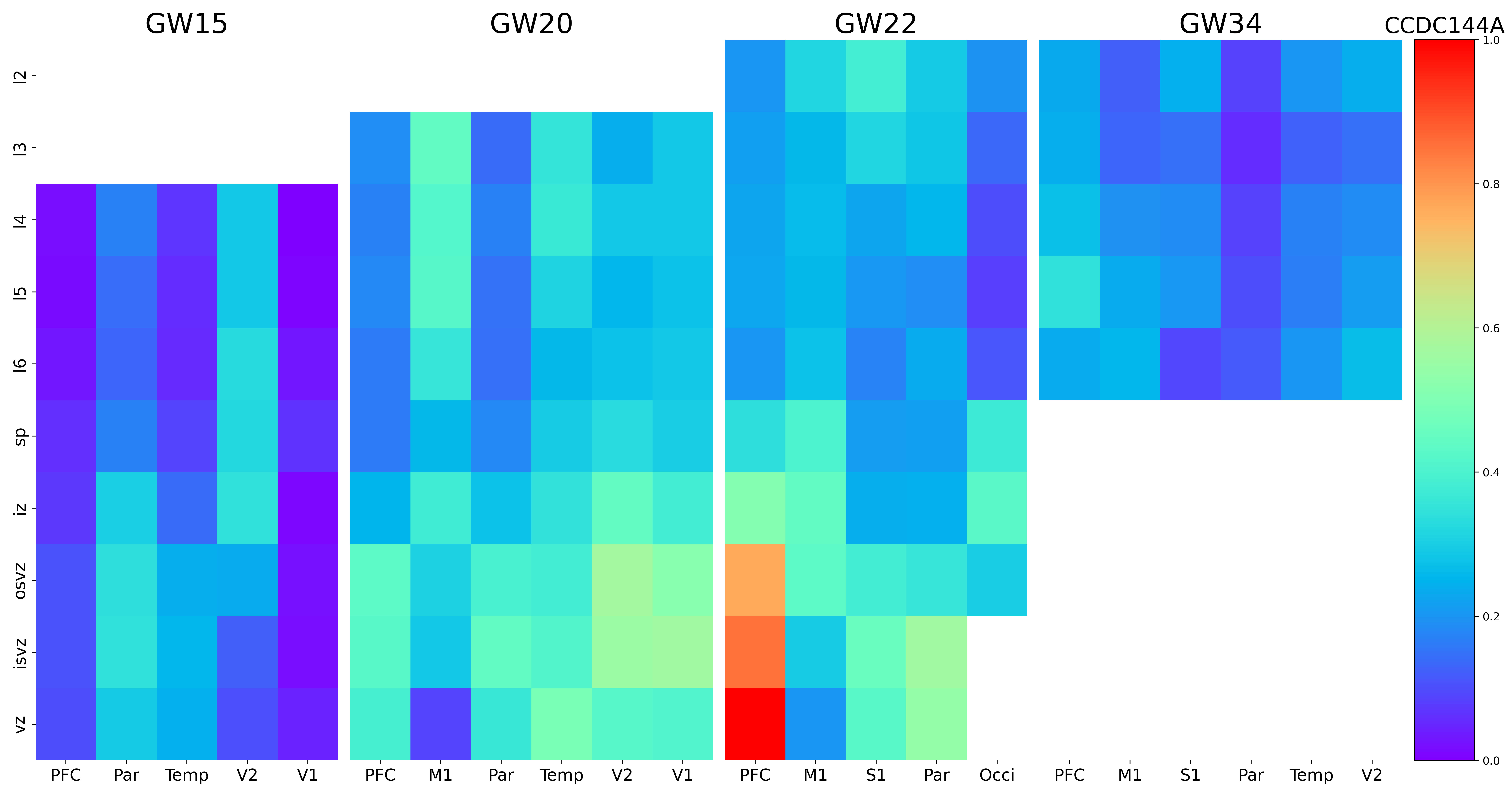

Supplement: Supplementary file 4 — Source Data Fig. 3: Expression pattern heatmap for all 300 genes in the MERFISH. [file 41586_2025_9010_MOESM4_ESM.zip › CCDC144A.png]

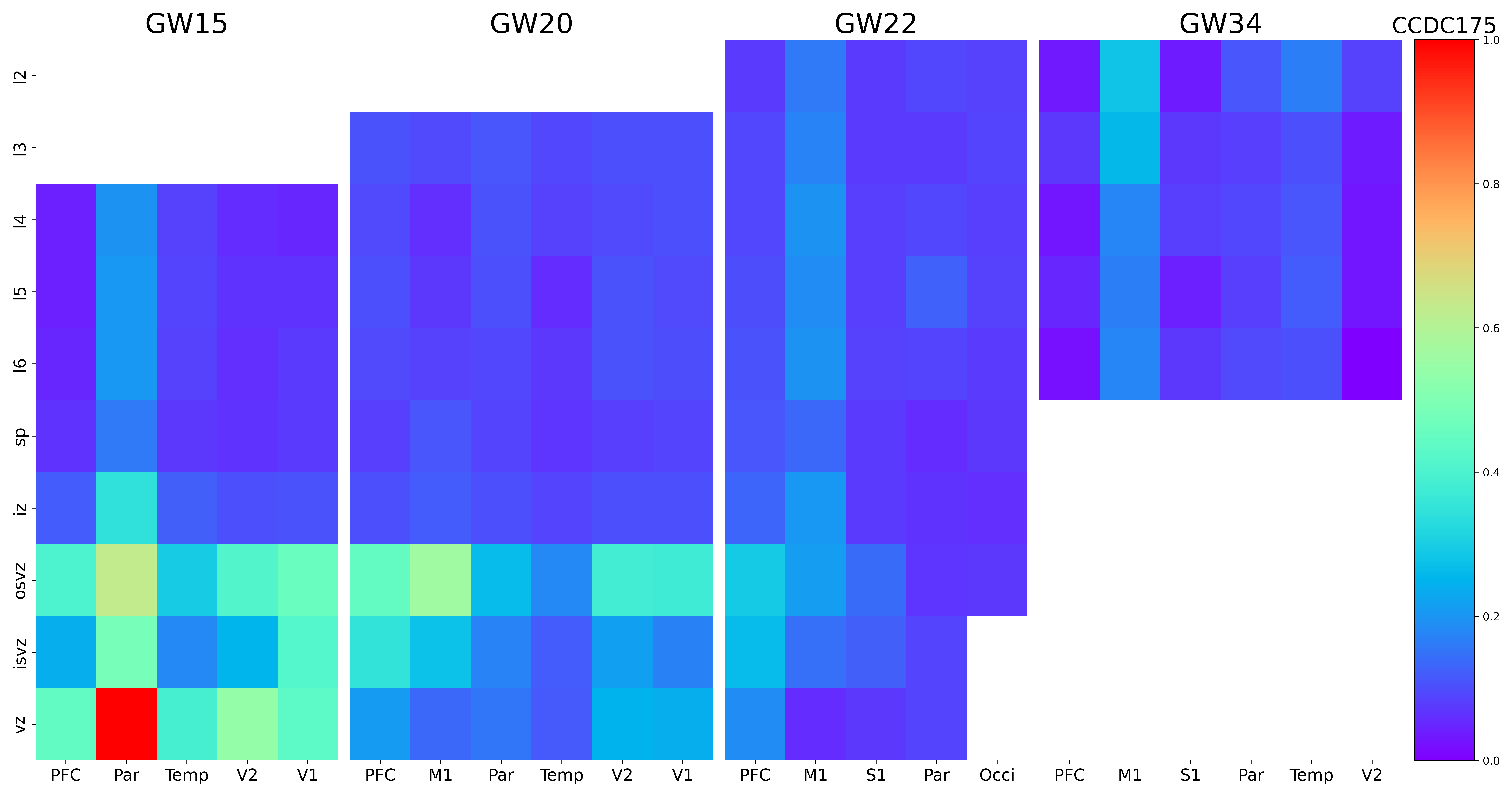

Supplement: Supplementary file 4 — Source Data Fig. 3: Expression pattern heatmap for all 300 genes in the MERFISH. [file 41586_2025_9010_MOESM4_ESM.zip › CCDC175.png]

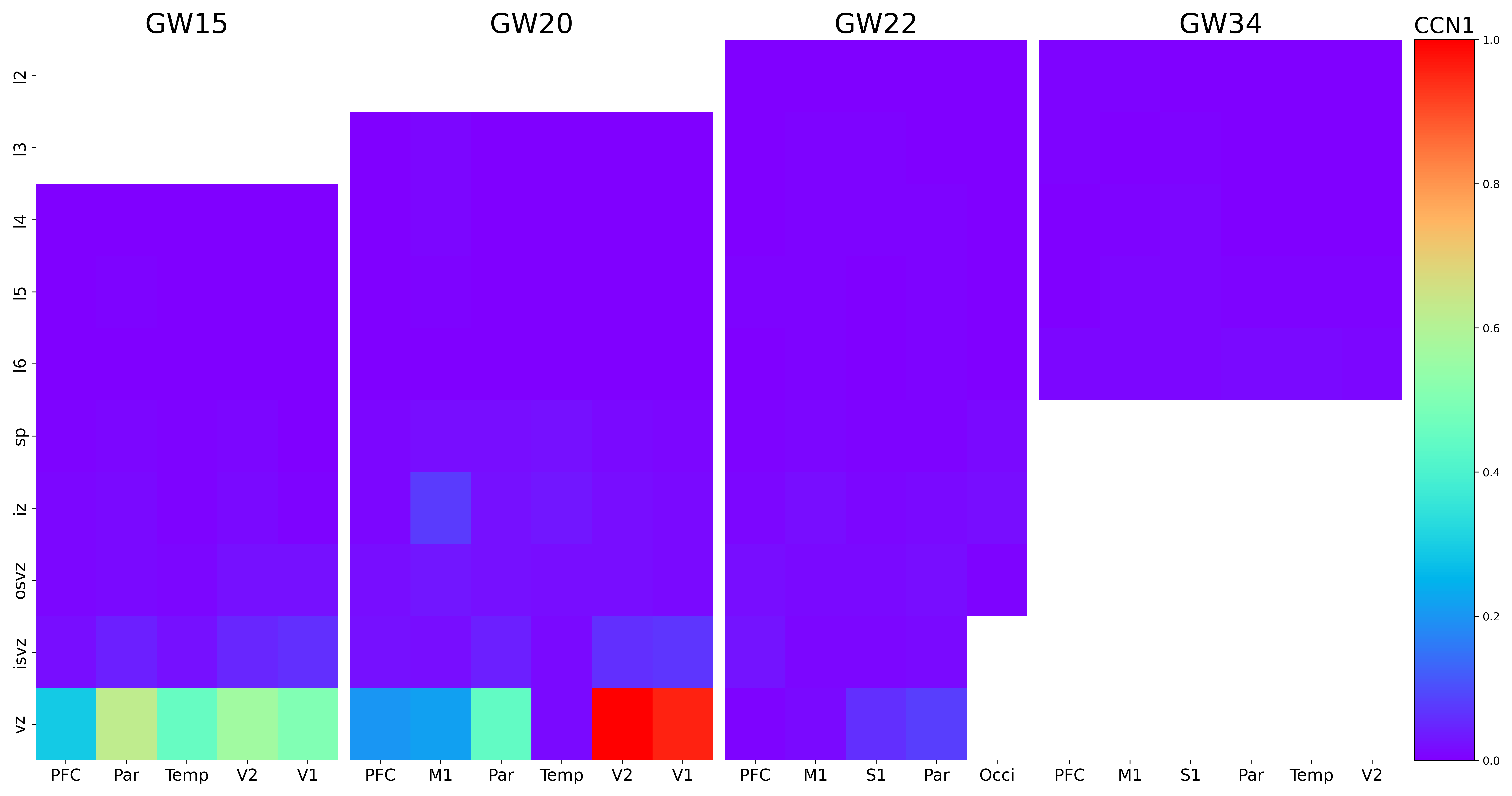

Supplement: Supplementary file 4 — Source Data Fig. 3: Expression pattern heatmap for all 300 genes in the MERFISH. [file 41586_2025_9010_MOESM4_ESM.zip › CCN1.png]

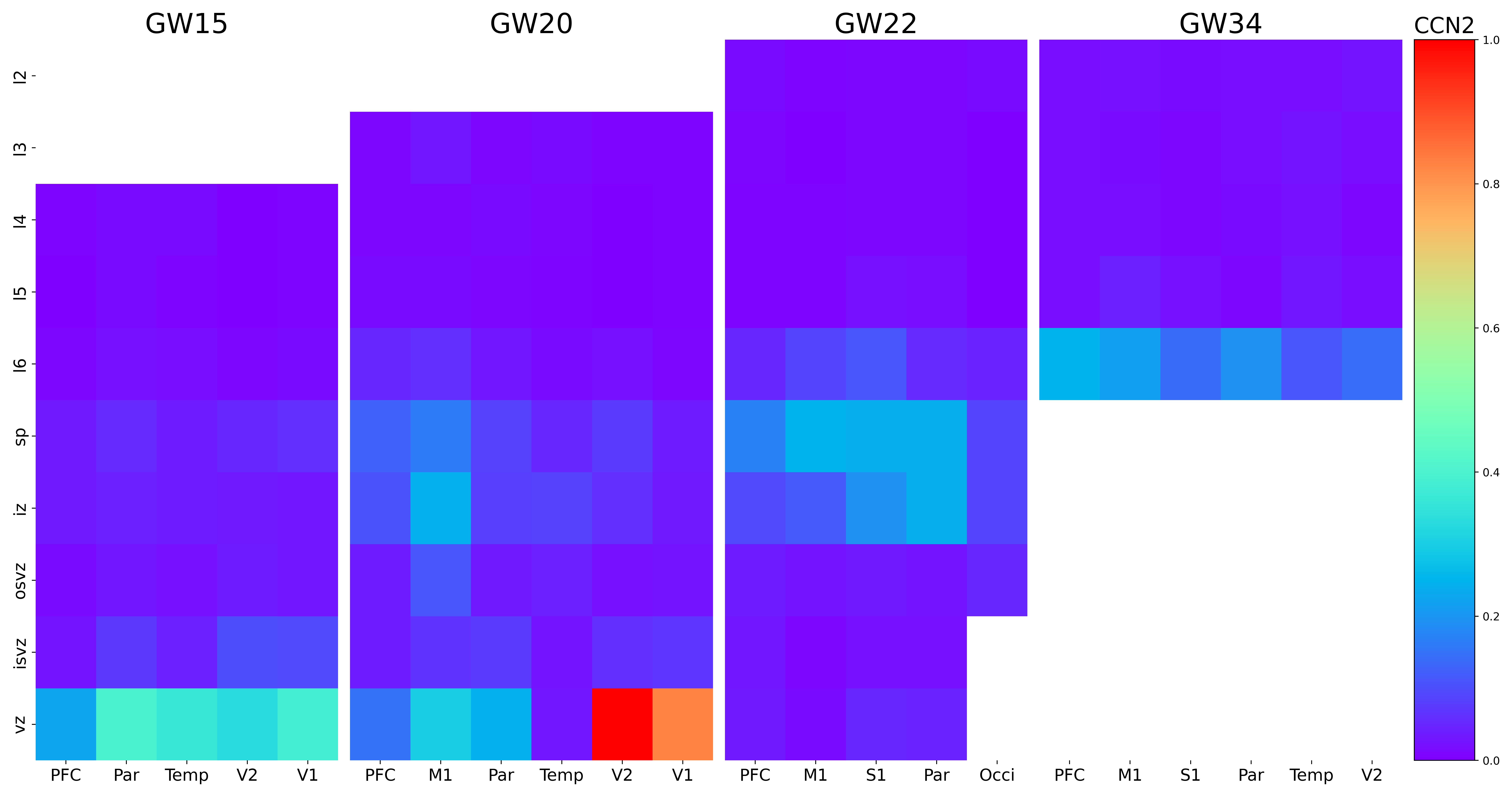

Supplement: Supplementary file 4 — Source Data Fig. 3: Expression pattern heatmap for all 300 genes in the MERFISH. [file 41586_2025_9010_MOESM4_ESM.zip › CCN2.png]

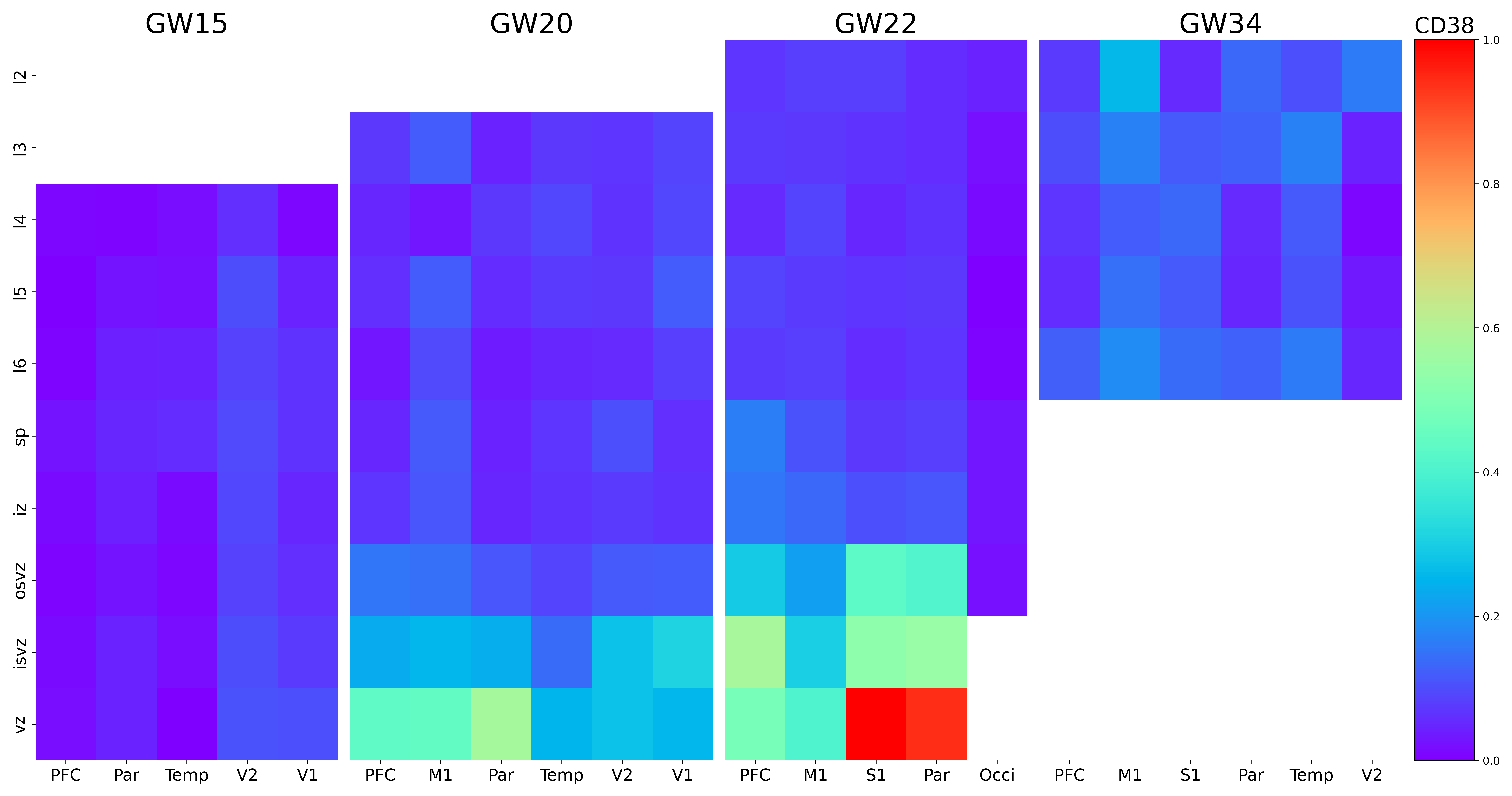

Supplement: Supplementary file 4 — Source Data Fig. 3: Expression pattern heatmap for all 300 genes in the MERFISH. [file 41586_2025_9010_MOESM4_ESM.zip › CD38.png]

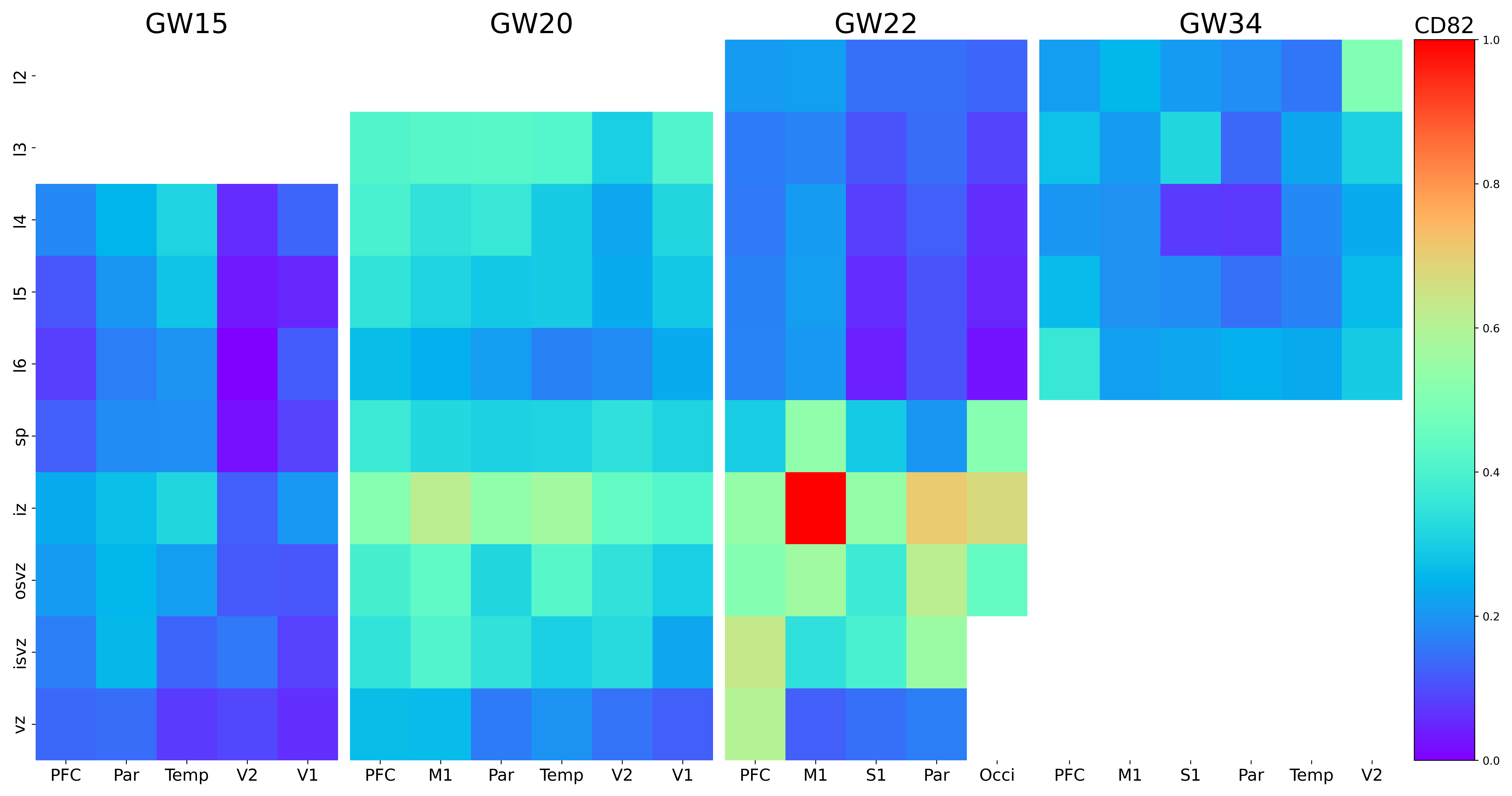

Supplement: Supplementary file 4 — Source Data Fig. 3: Expression pattern heatmap for all 300 genes in the MERFISH. [file 41586_2025_9010_MOESM4_ESM.zip › CD82.png]

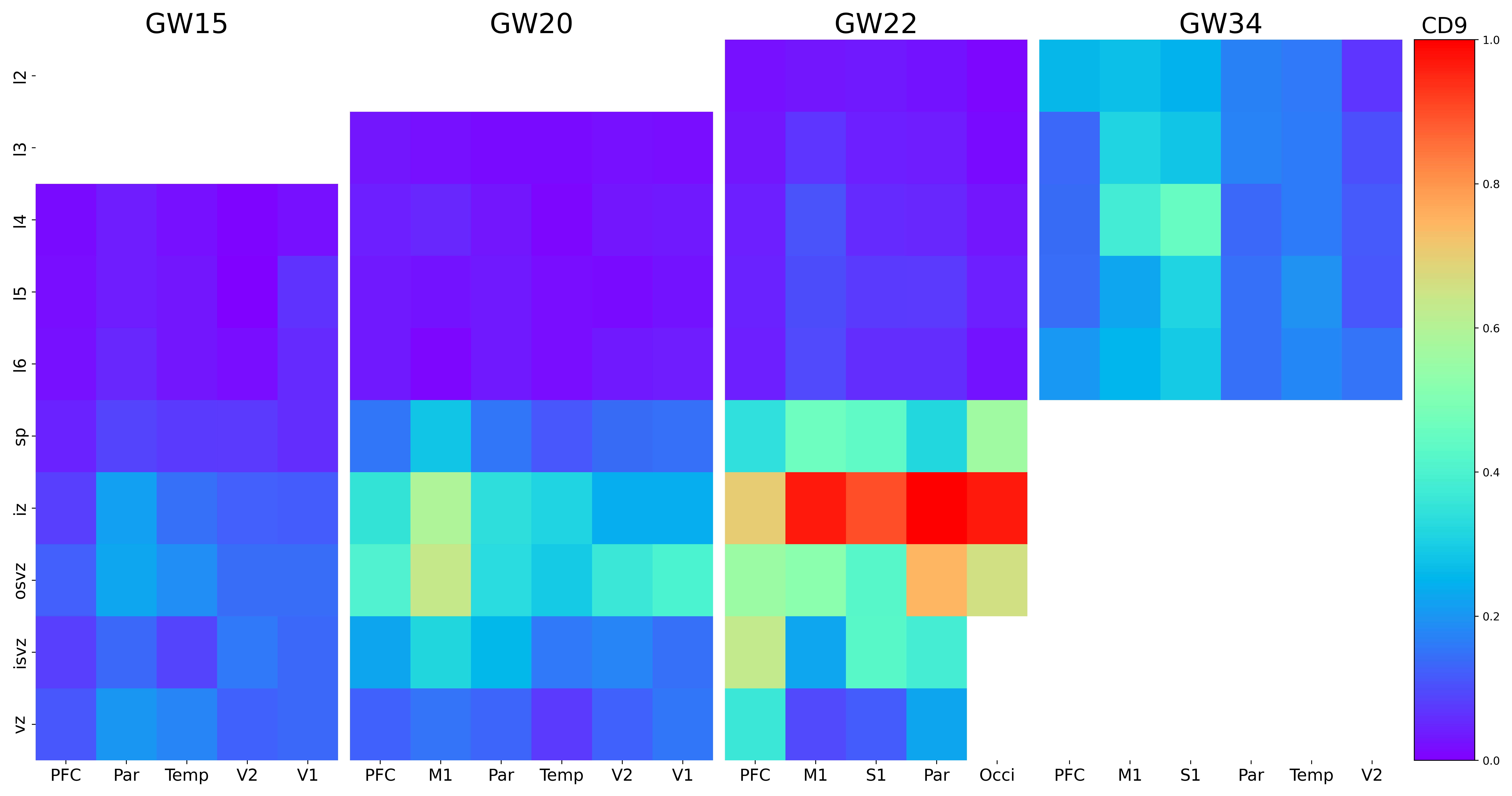

Supplement: Supplementary file 4 — Source Data Fig. 3: Expression pattern heatmap for all 300 genes in the MERFISH. [file 41586_2025_9010_MOESM4_ESM.zip › CD9.png]

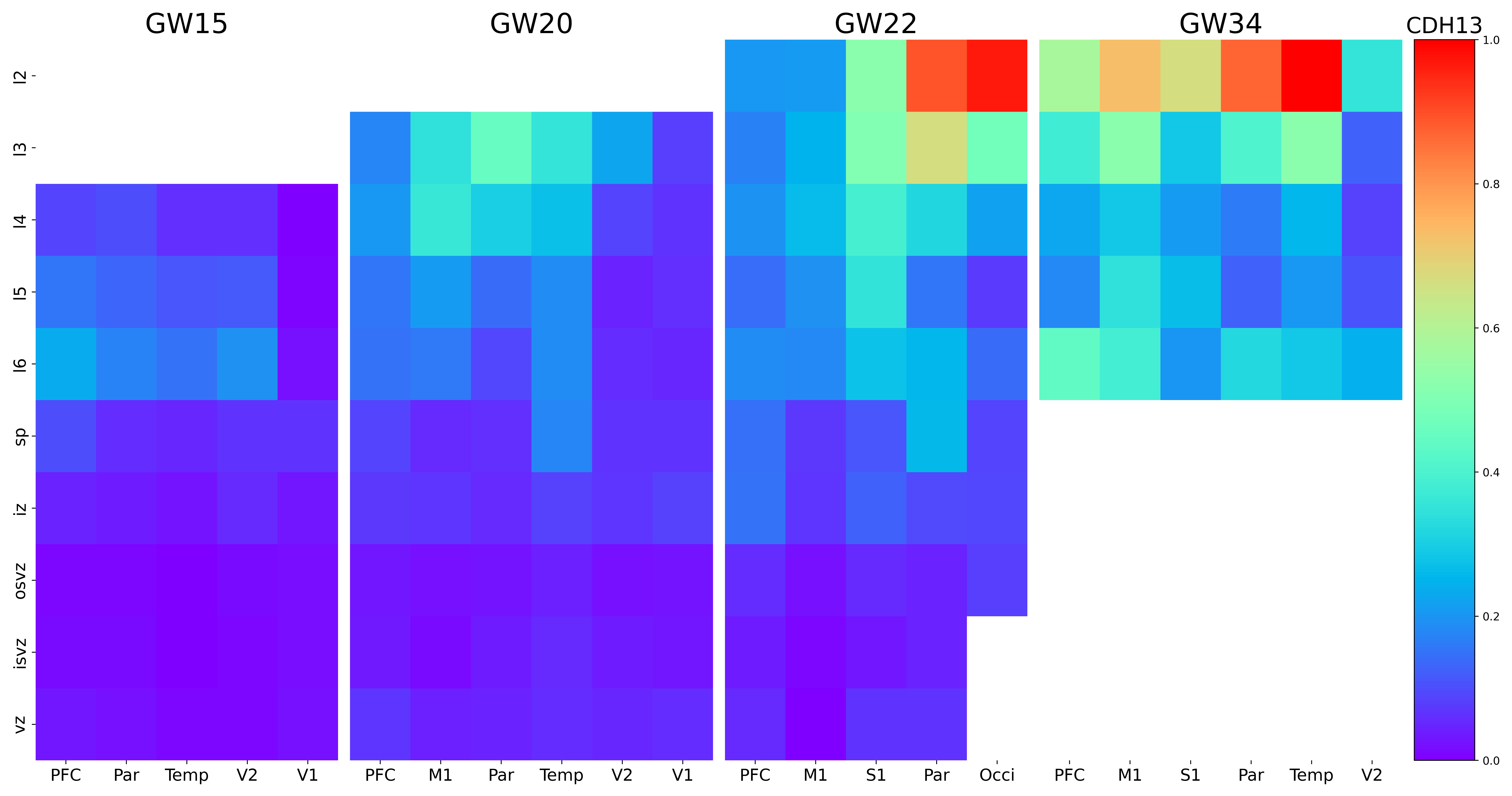

Supplement: Supplementary file 4 — Source Data Fig. 3: Expression pattern heatmap for all 300 genes in the MERFISH. [file 41586_2025_9010_MOESM4_ESM.zip › CDH13.png]

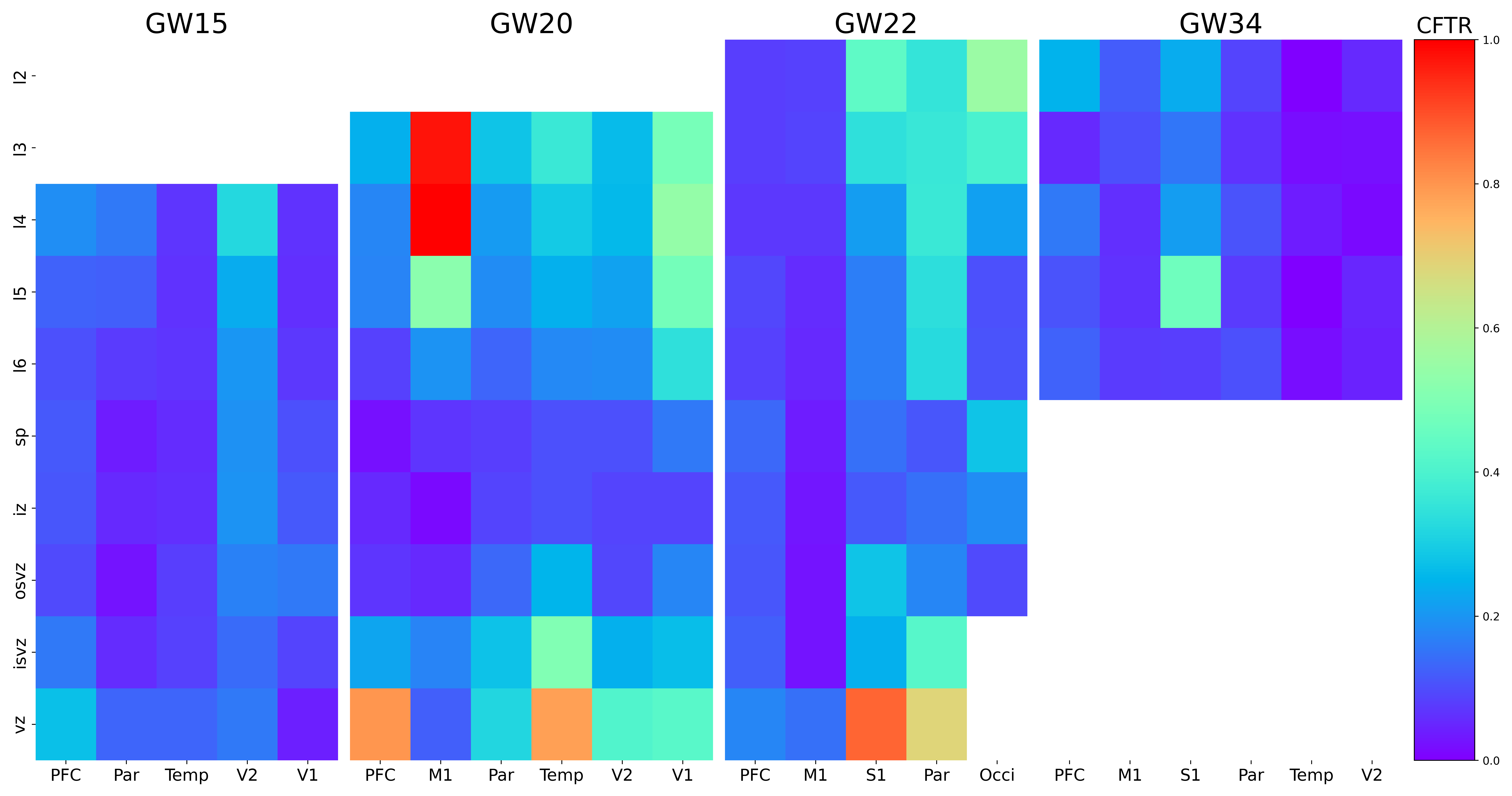

Supplement: Supplementary file 4 — Source Data Fig. 3: Expression pattern heatmap for all 300 genes in the MERFISH. [file 41586_2025_9010_MOESM4_ESM.zip › CFTR.png]

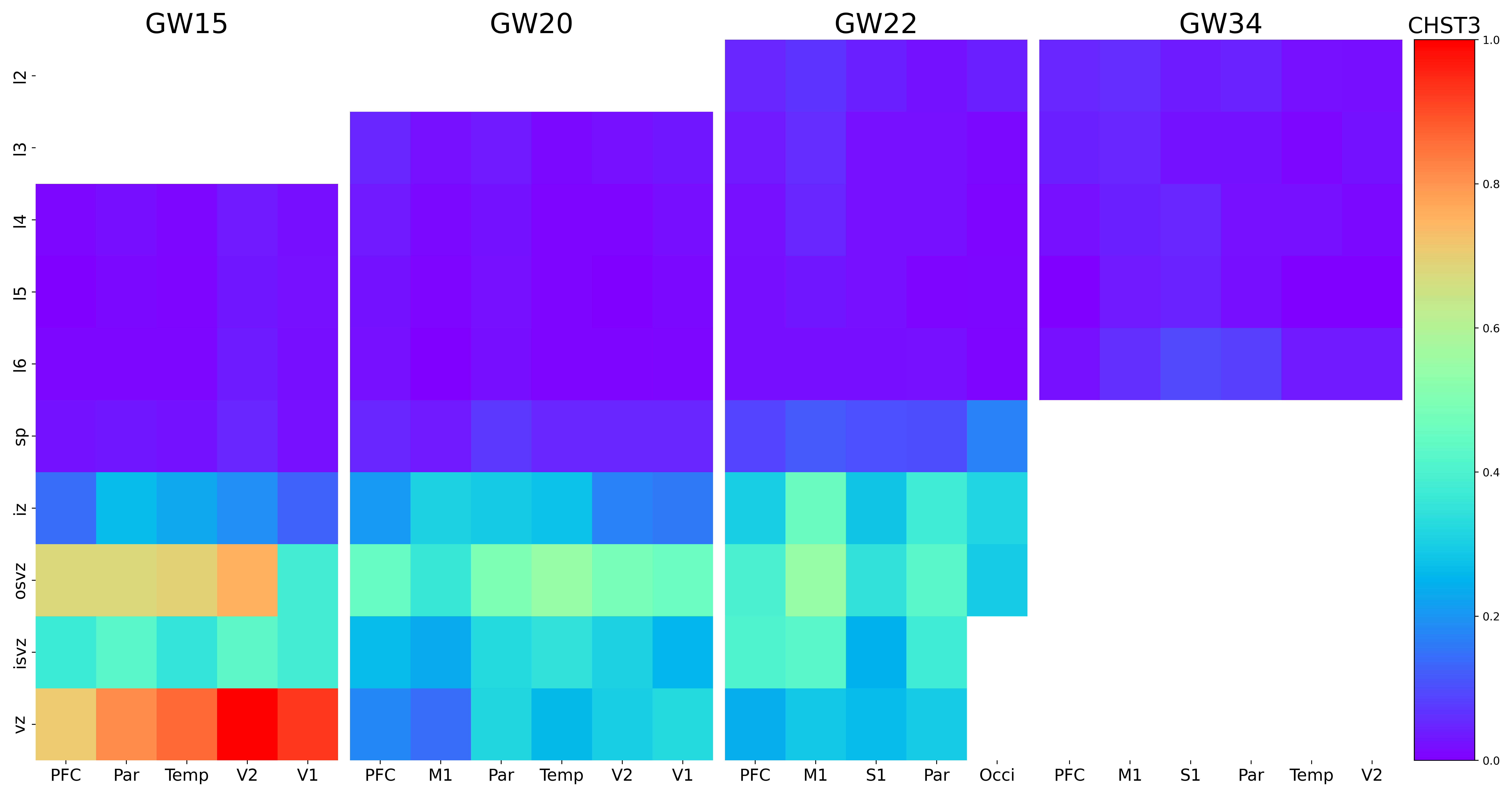

Supplement: Supplementary file 4 — Source Data Fig. 3: Expression pattern heatmap for all 300 genes in the MERFISH. [file 41586_2025_9010_MOESM4_ESM.zip › CHST3.png]

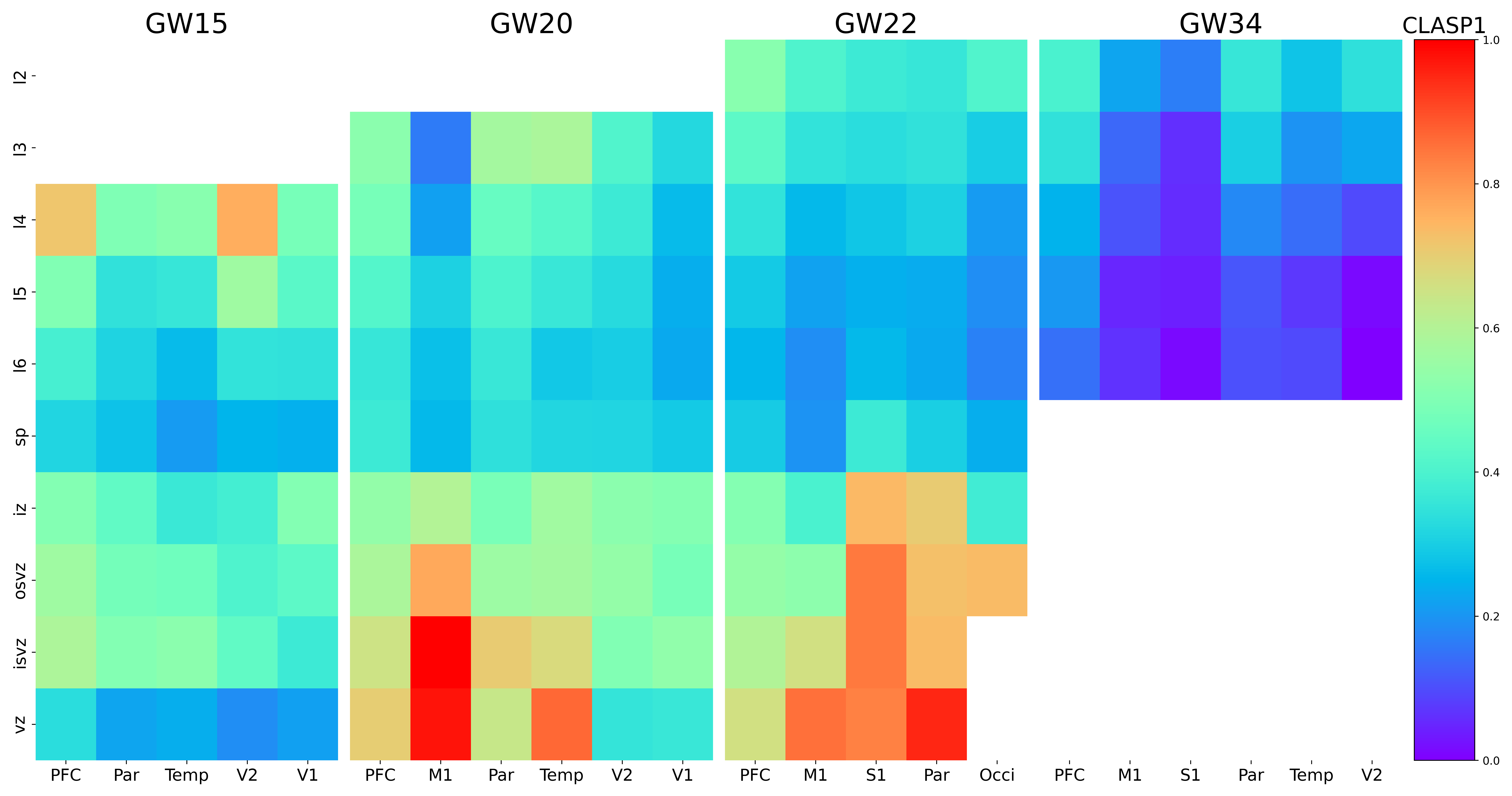

Supplement: Supplementary file 4 — Source Data Fig. 3: Expression pattern heatmap for all 300 genes in the MERFISH. [file 41586_2025_9010_MOESM4_ESM.zip › CLASP1.png]

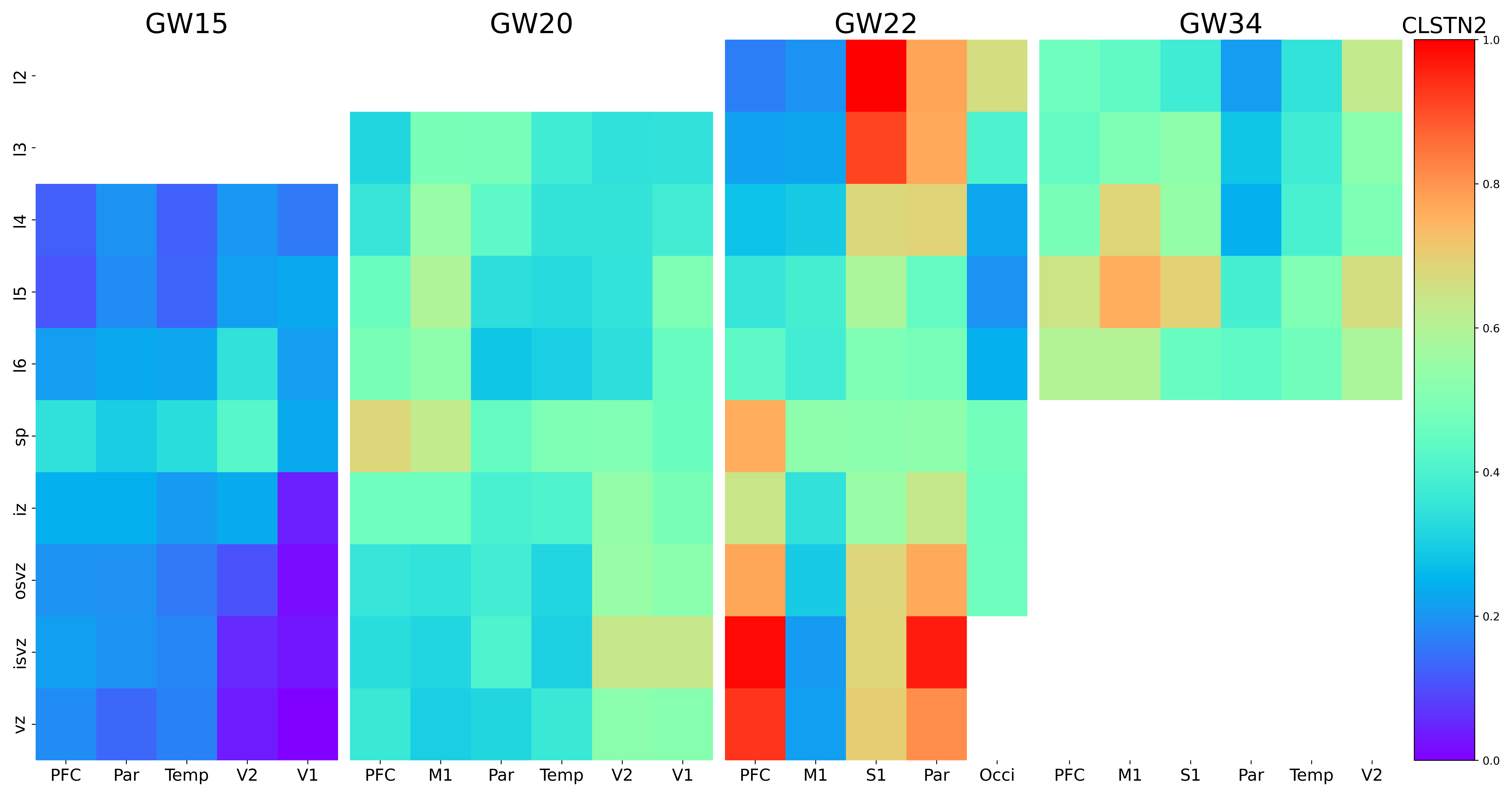

Supplement: Supplementary file 4 — Source Data Fig. 3: Expression pattern heatmap for all 300 genes in the MERFISH. [file 41586_2025_9010_MOESM4_ESM.zip › CLSTN2.png]

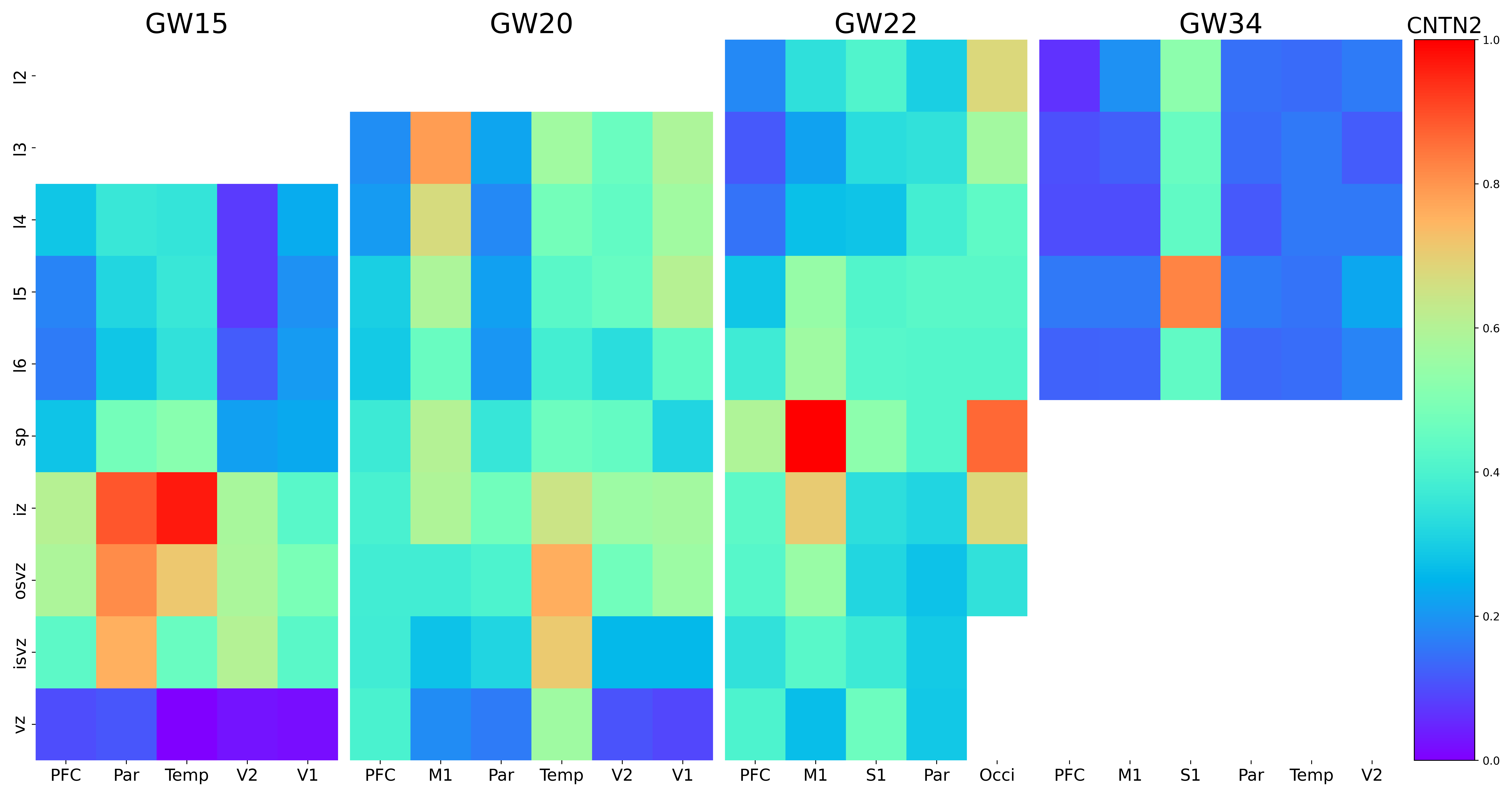

Supplement: Supplementary file 4 — Source Data Fig. 3: Expression pattern heatmap for all 300 genes in the MERFISH. [file 41586_2025_9010_MOESM4_ESM.zip › CNTN2.png]

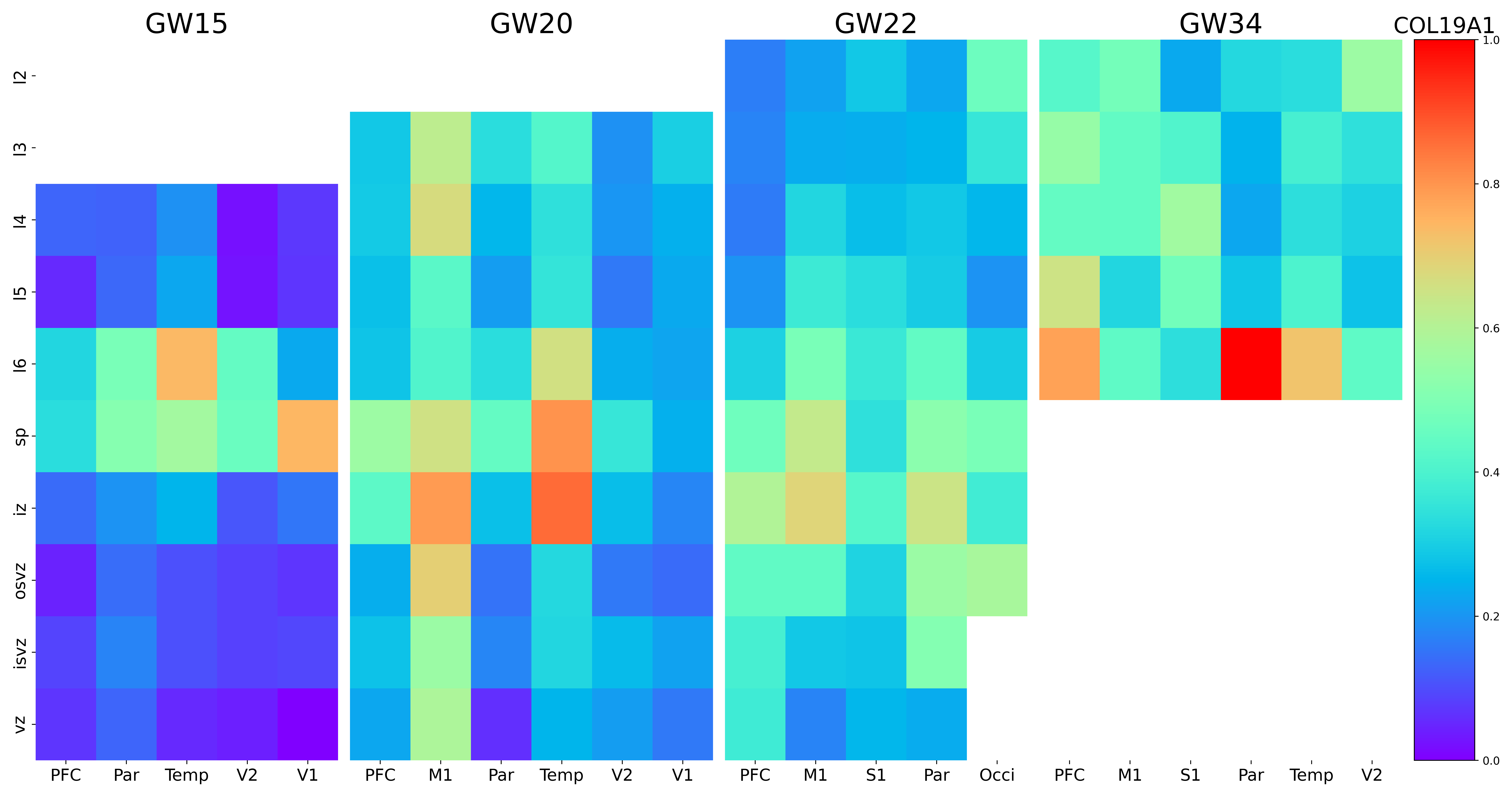

Supplement: Supplementary file 4 — Source Data Fig. 3: Expression pattern heatmap for all 300 genes in the MERFISH. [file 41586_2025_9010_MOESM4_ESM.zip › COL19A1.png]

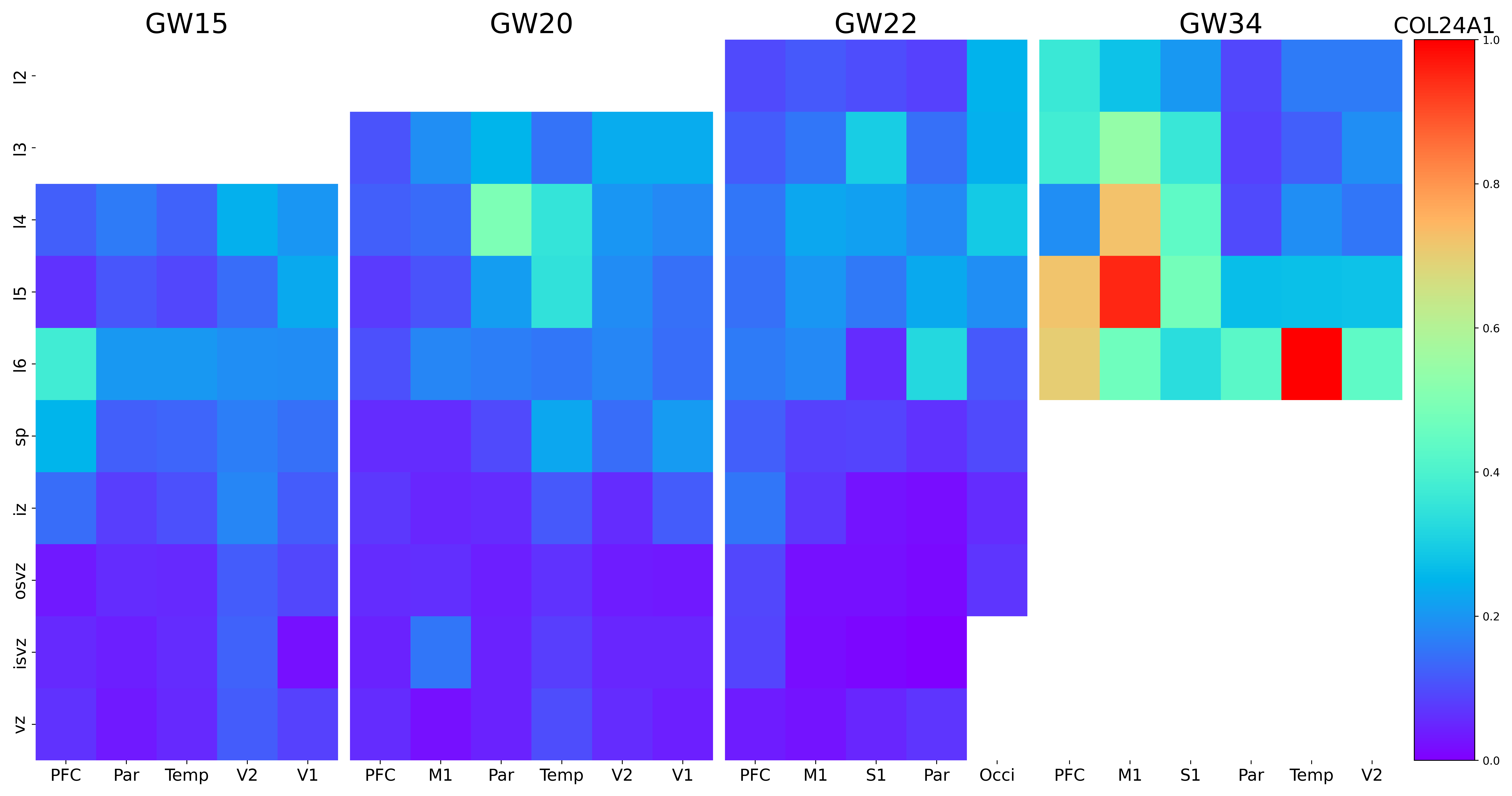

Supplement: Supplementary file 4 — Source Data Fig. 3: Expression pattern heatmap for all 300 genes in the MERFISH. [file 41586_2025_9010_MOESM4_ESM.zip › COL24A1.png]

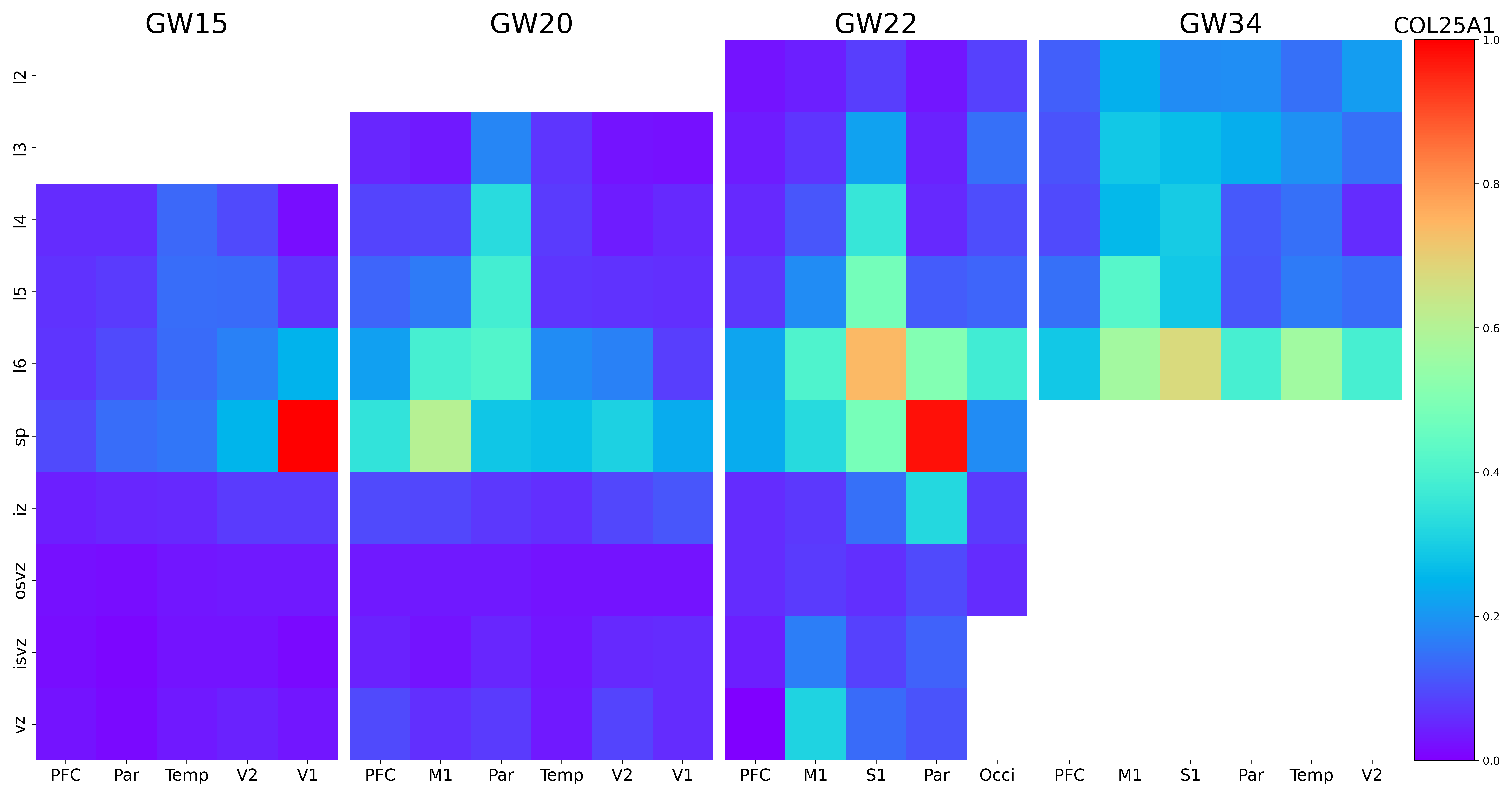

Supplement: Supplementary file 4 — Source Data Fig. 3: Expression pattern heatmap for all 300 genes in the MERFISH. [file 41586_2025_9010_MOESM4_ESM.zip › COL25A1.png]

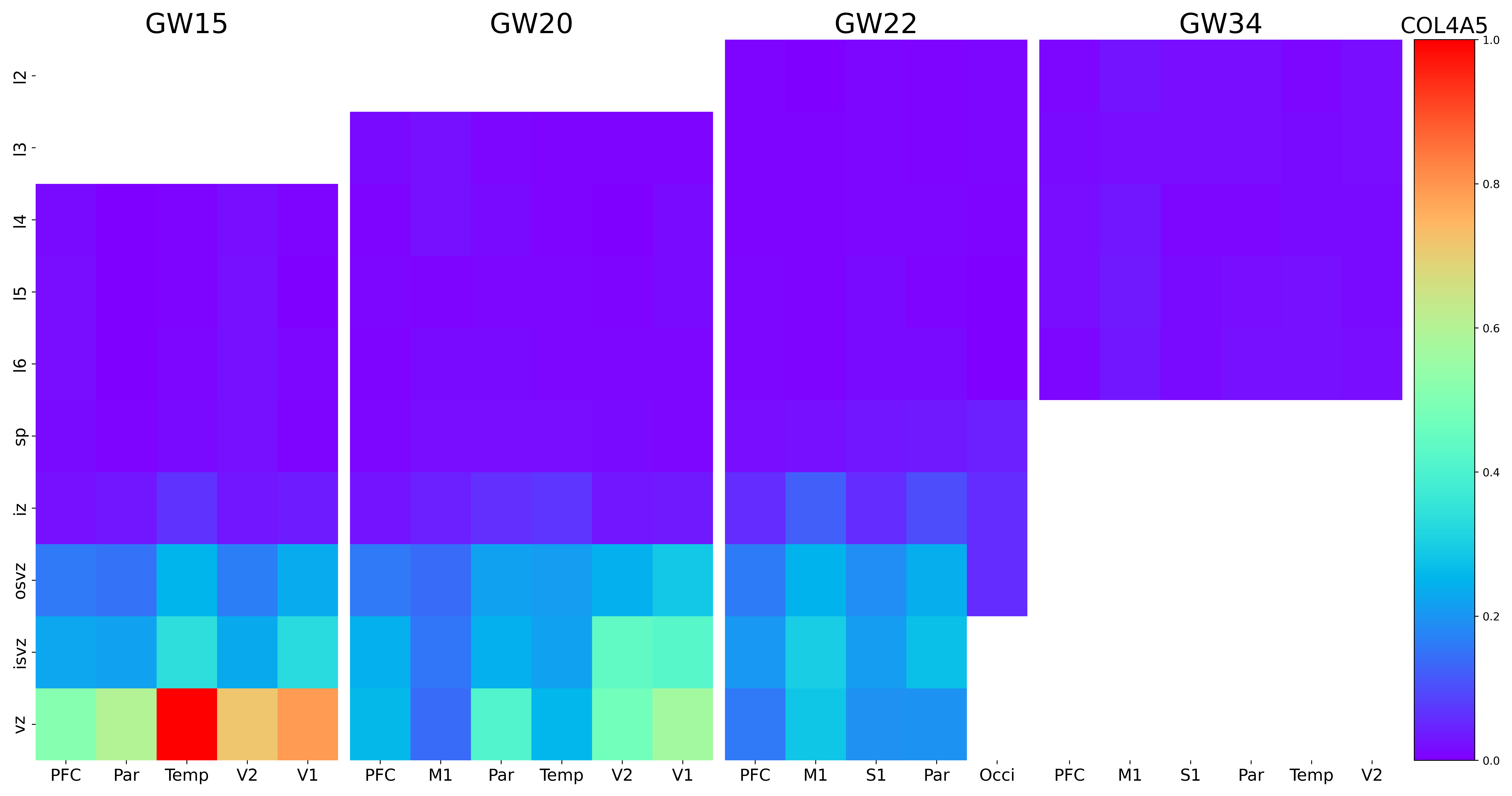

Supplement: Supplementary file 4 — Source Data Fig. 3: Expression pattern heatmap for all 300 genes in the MERFISH. [file 41586_2025_9010_MOESM4_ESM.zip › COL4A5.png]

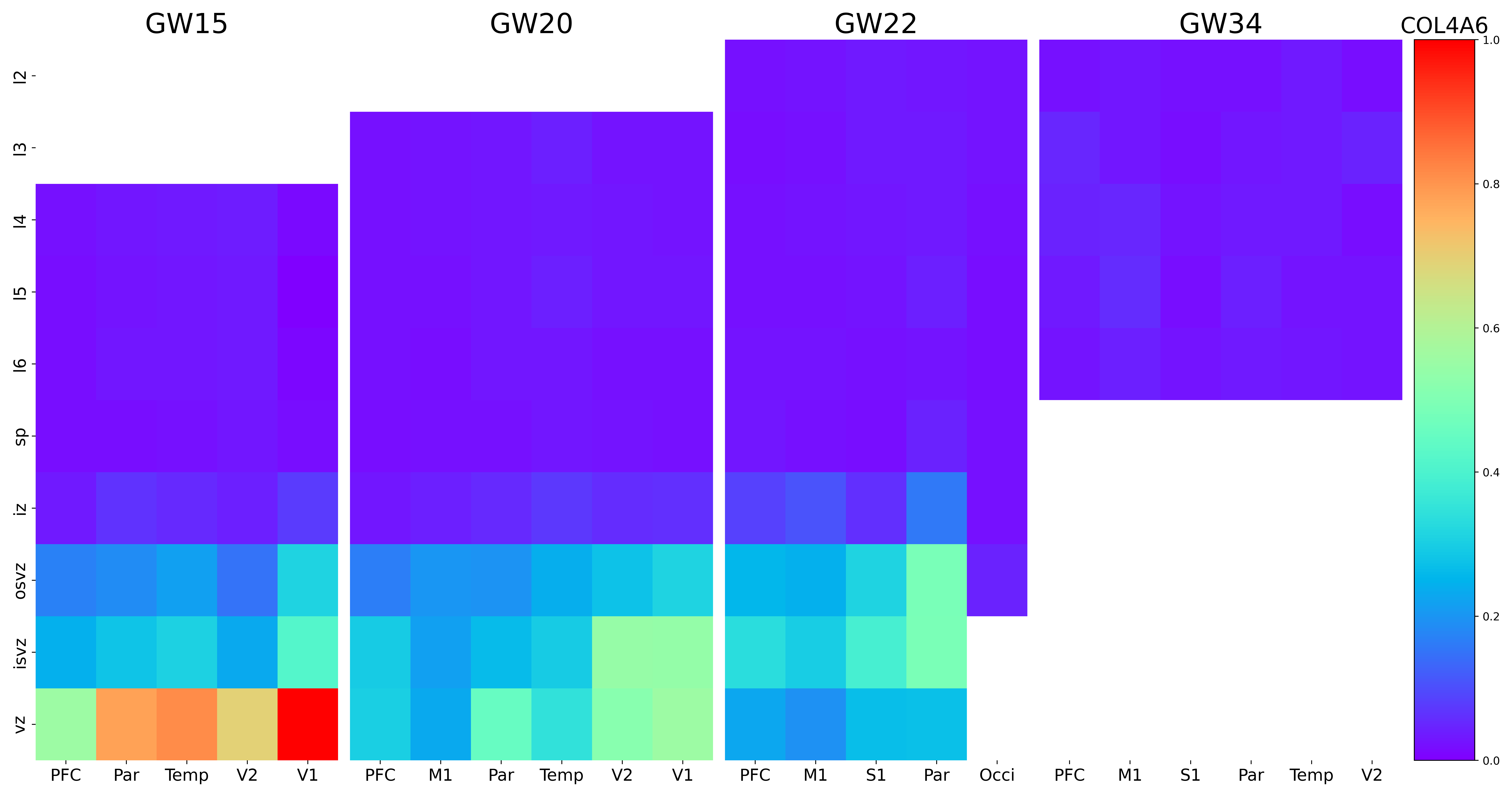

Supplement: Supplementary file 4 — Source Data Fig. 3: Expression pattern heatmap for all 300 genes in the MERFISH. [file 41586_2025_9010_MOESM4_ESM.zip › COL4A6.png]

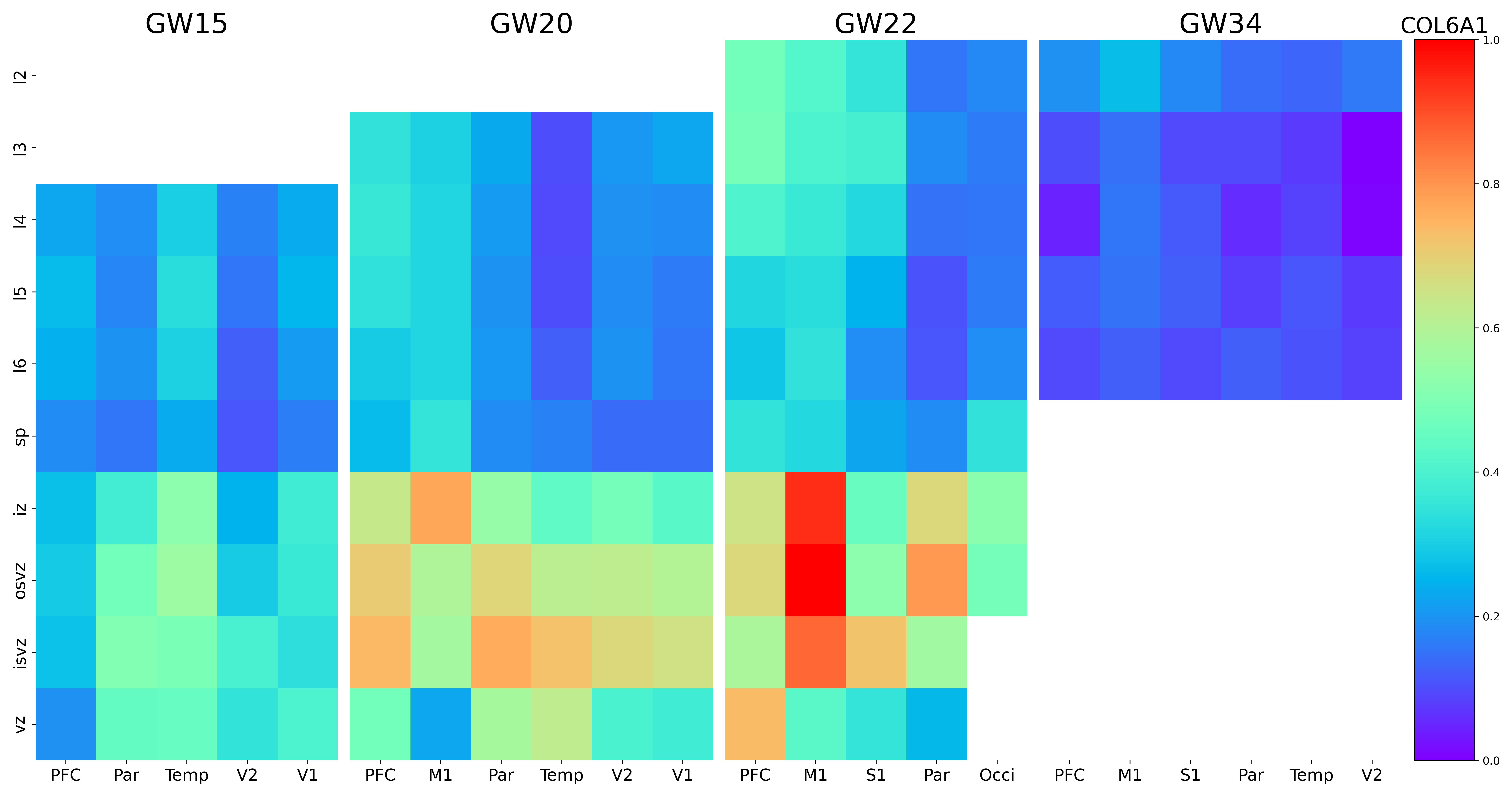

Supplement: Supplementary file 4 — Source Data Fig. 3: Expression pattern heatmap for all 300 genes in the MERFISH. [file 41586_2025_9010_MOESM4_ESM.zip › COL6A1.png]

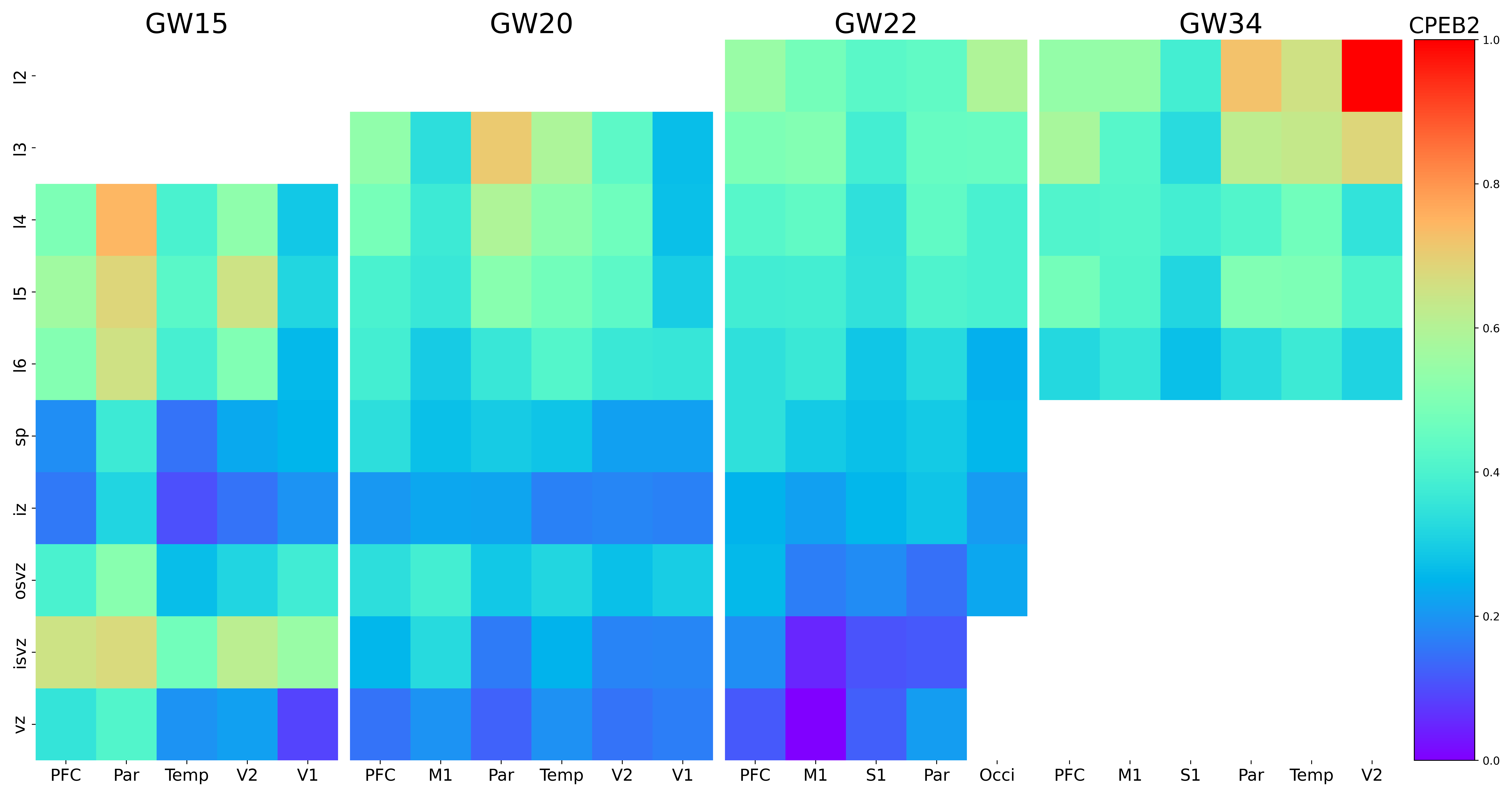

Supplement: Supplementary file 4 — Source Data Fig. 3: Expression pattern heatmap for all 300 genes in the MERFISH. [file 41586_2025_9010_MOESM4_ESM.zip › CPEB2.png]

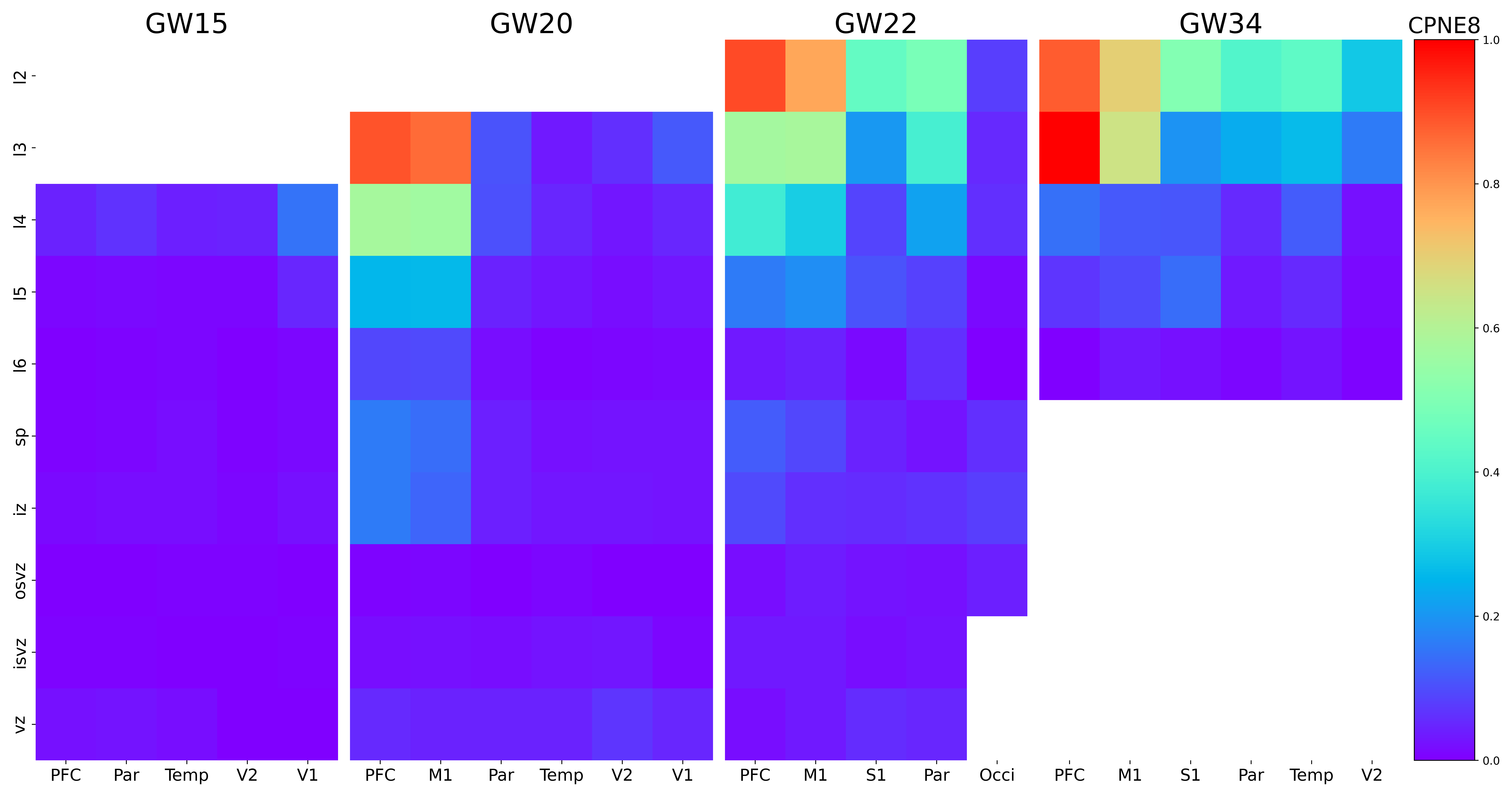

Supplement: Supplementary file 4 — Source Data Fig. 3: Expression pattern heatmap for all 300 genes in the MERFISH. [file 41586_2025_9010_MOESM4_ESM.zip › CPNE8.png]

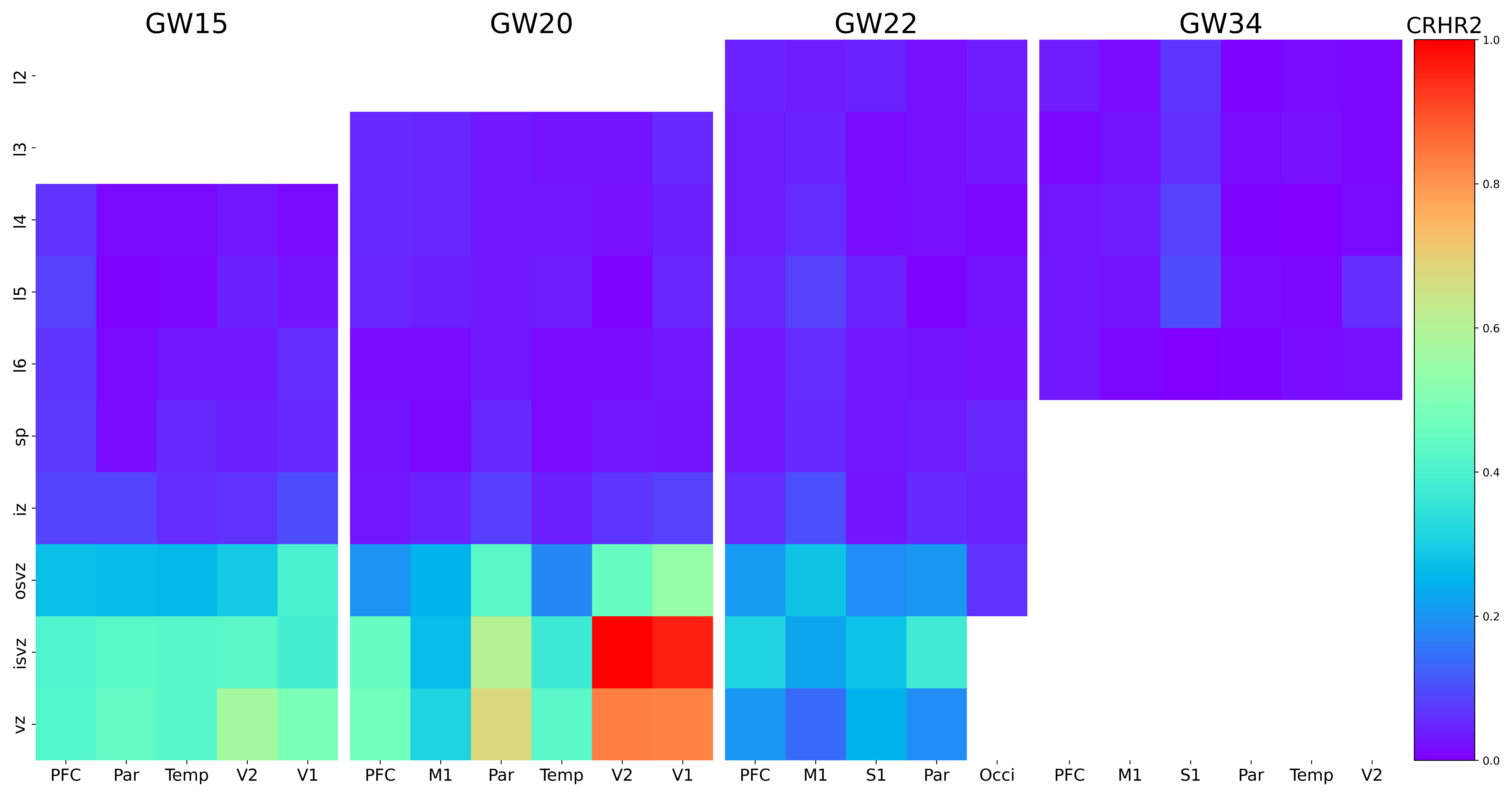

Supplement: Supplementary file 4 — Source Data Fig. 3: Expression pattern heatmap for all 300 genes in the MERFISH. [file 41586_2025_9010_MOESM4_ESM.zip › CRHR2.png]

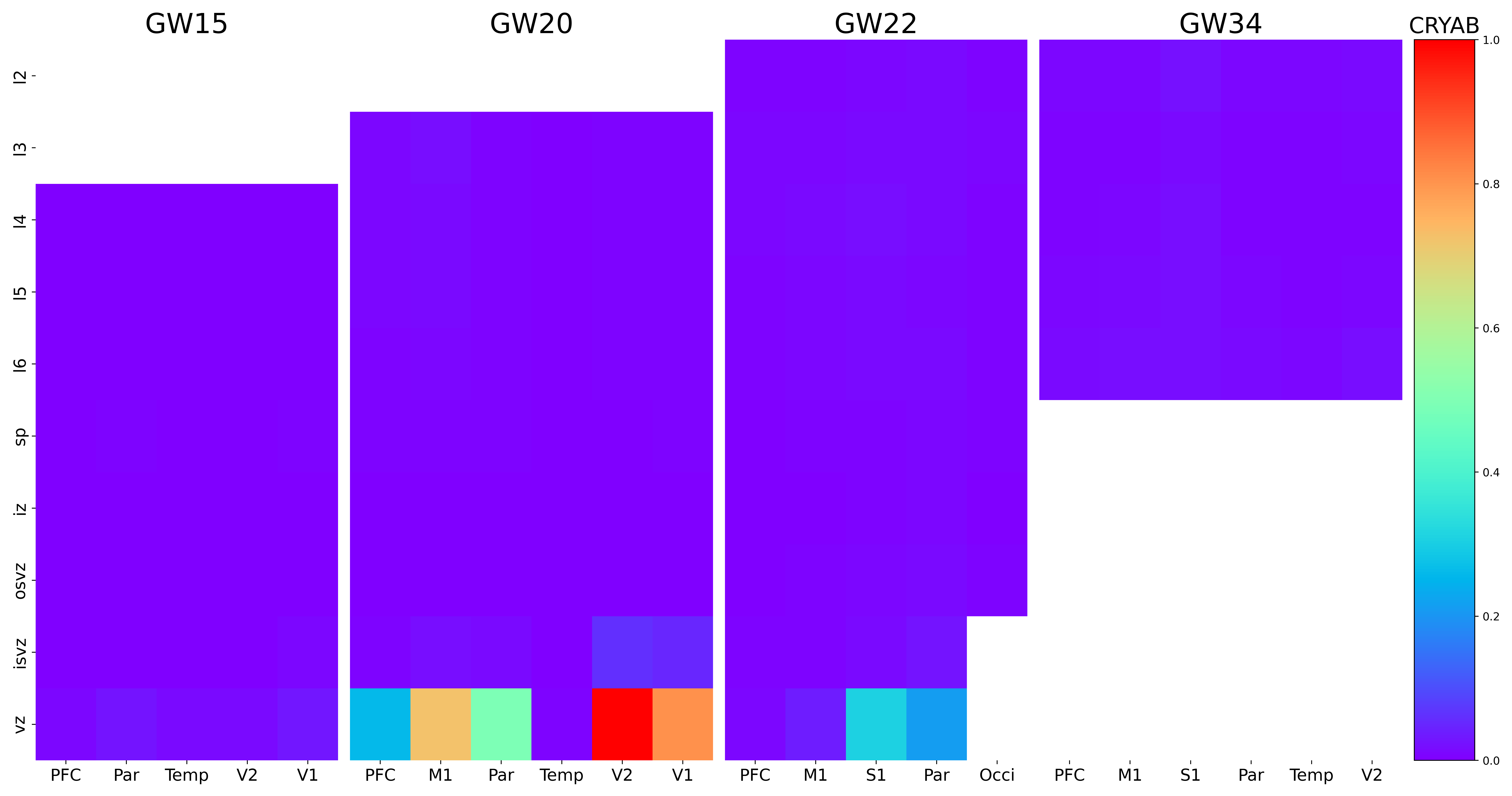

Supplement: Supplementary file 4 — Source Data Fig. 3: Expression pattern heatmap for all 300 genes in the MERFISH. [file 41586_2025_9010_MOESM4_ESM.zip › CRYAB.png]

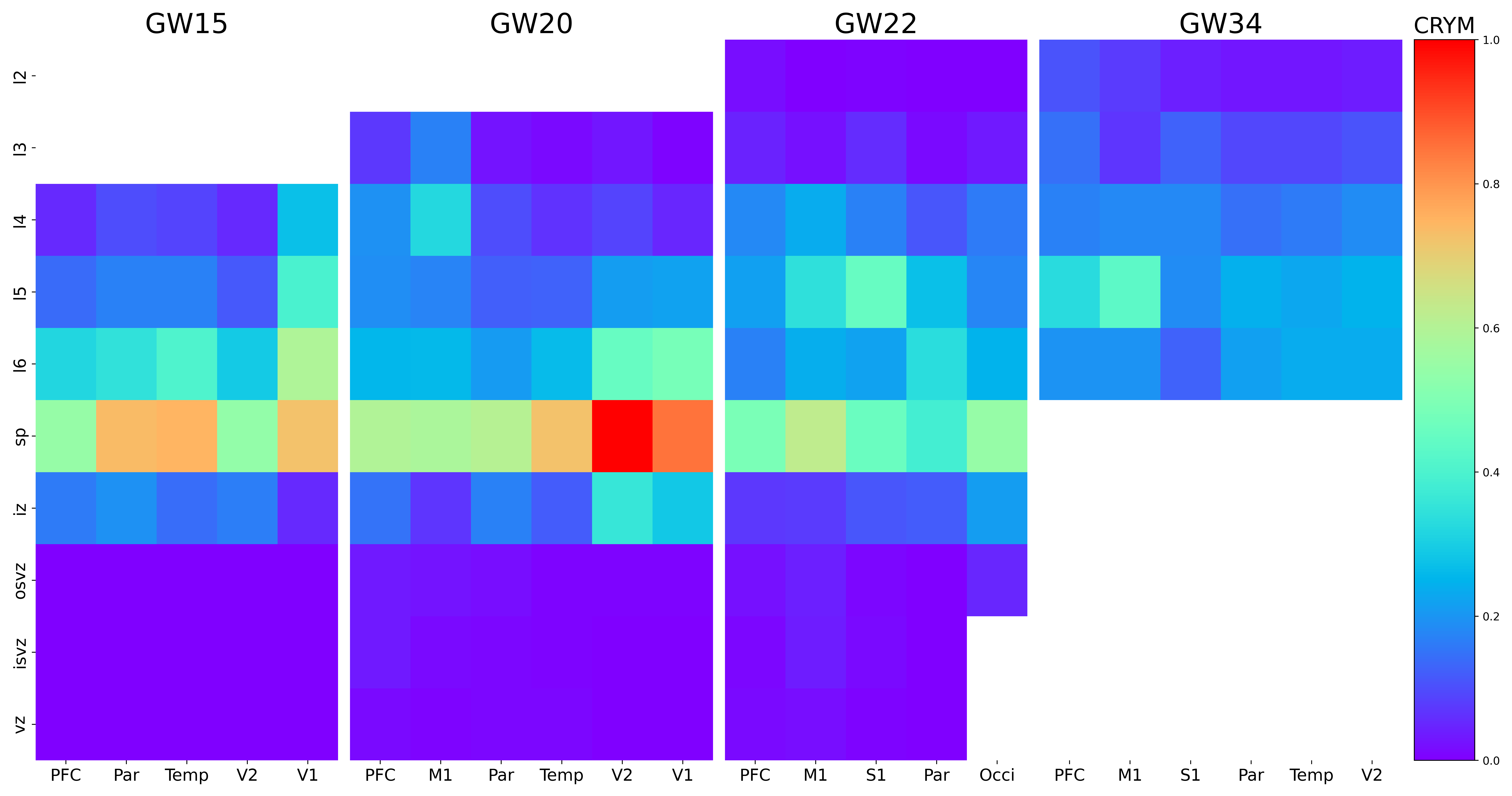

Supplement: Supplementary file 4 — Source Data Fig. 3: Expression pattern heatmap for all 300 genes in the MERFISH. [file 41586_2025_9010_MOESM4_ESM.zip › CRYM.png]

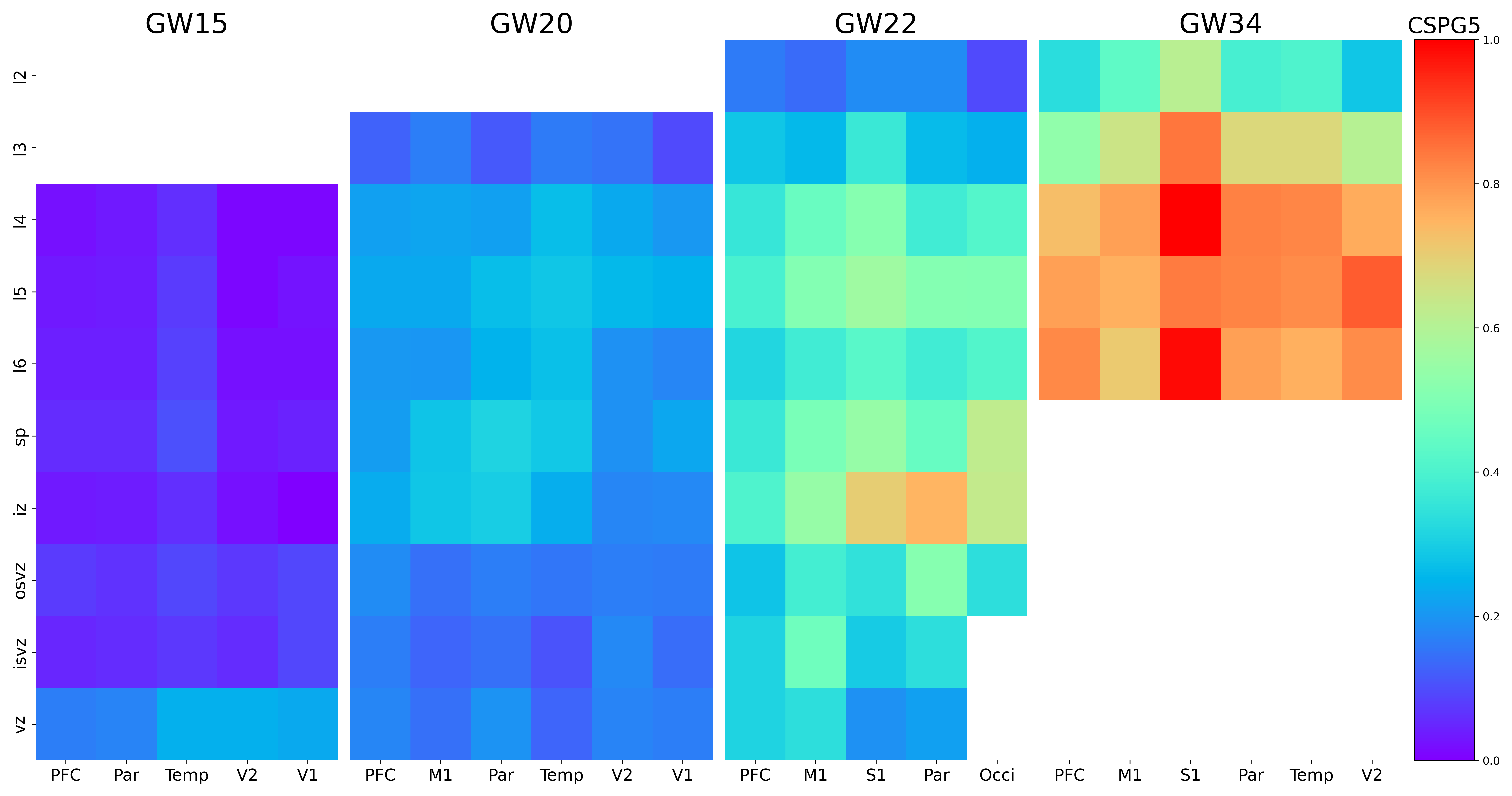

Supplement: Supplementary file 4 — Source Data Fig. 3: Expression pattern heatmap for all 300 genes in the MERFISH. [file 41586_2025_9010_MOESM4_ESM.zip › CSPG5.png]

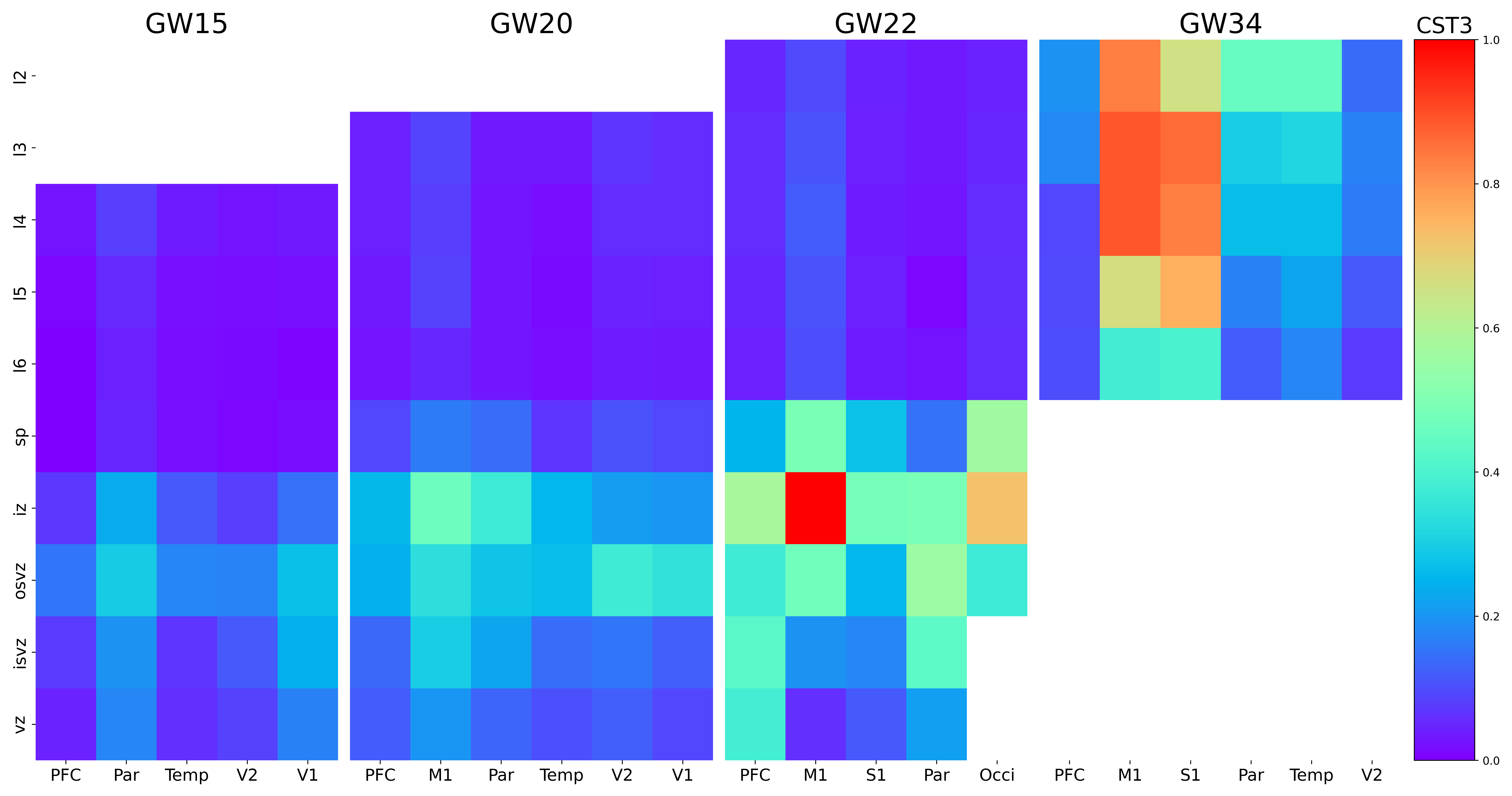

Supplement: Supplementary file 4 — Source Data Fig. 3: Expression pattern heatmap for all 300 genes in the MERFISH. [file 41586_2025_9010_MOESM4_ESM.zip › CST3.png]

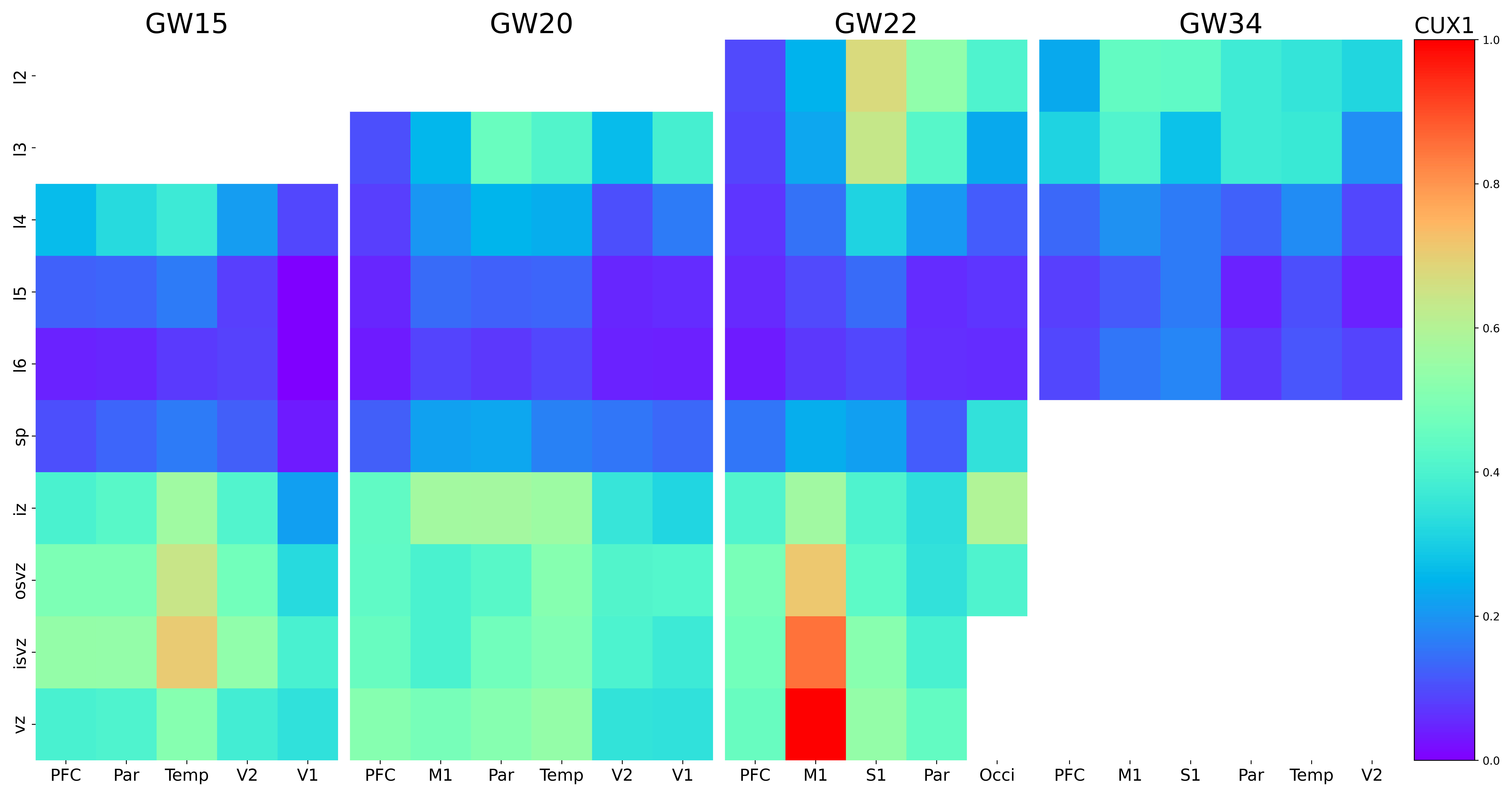

Supplement: Supplementary file 4 — Source Data Fig. 3: Expression pattern heatmap for all 300 genes in the MERFISH. [file 41586_2025_9010_MOESM4_ESM.zip › CUX1.png]

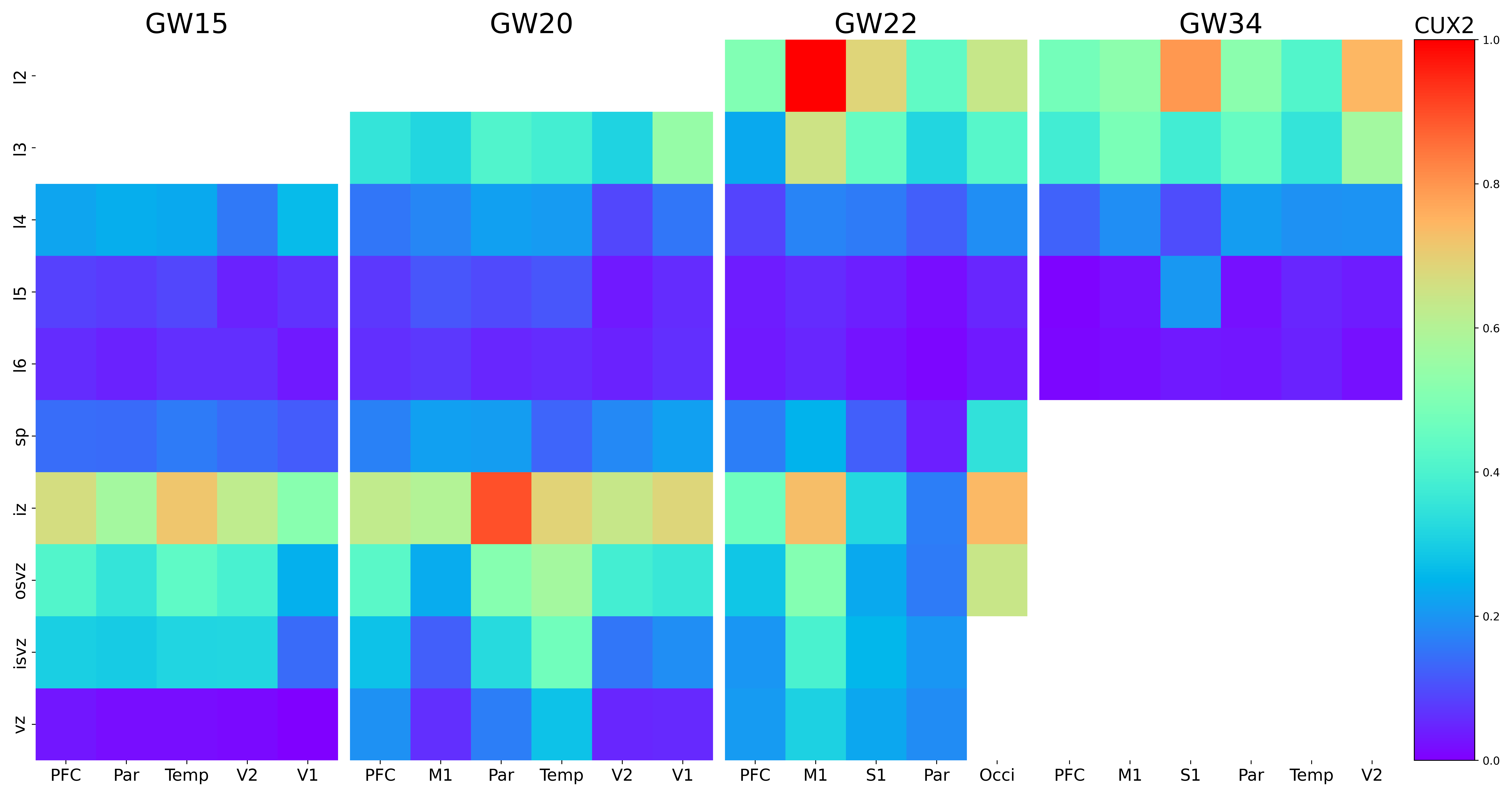

Supplement: Supplementary file 4 — Source Data Fig. 3: Expression pattern heatmap for all 300 genes in the MERFISH. [file 41586_2025_9010_MOESM4_ESM.zip › CUX2.png]

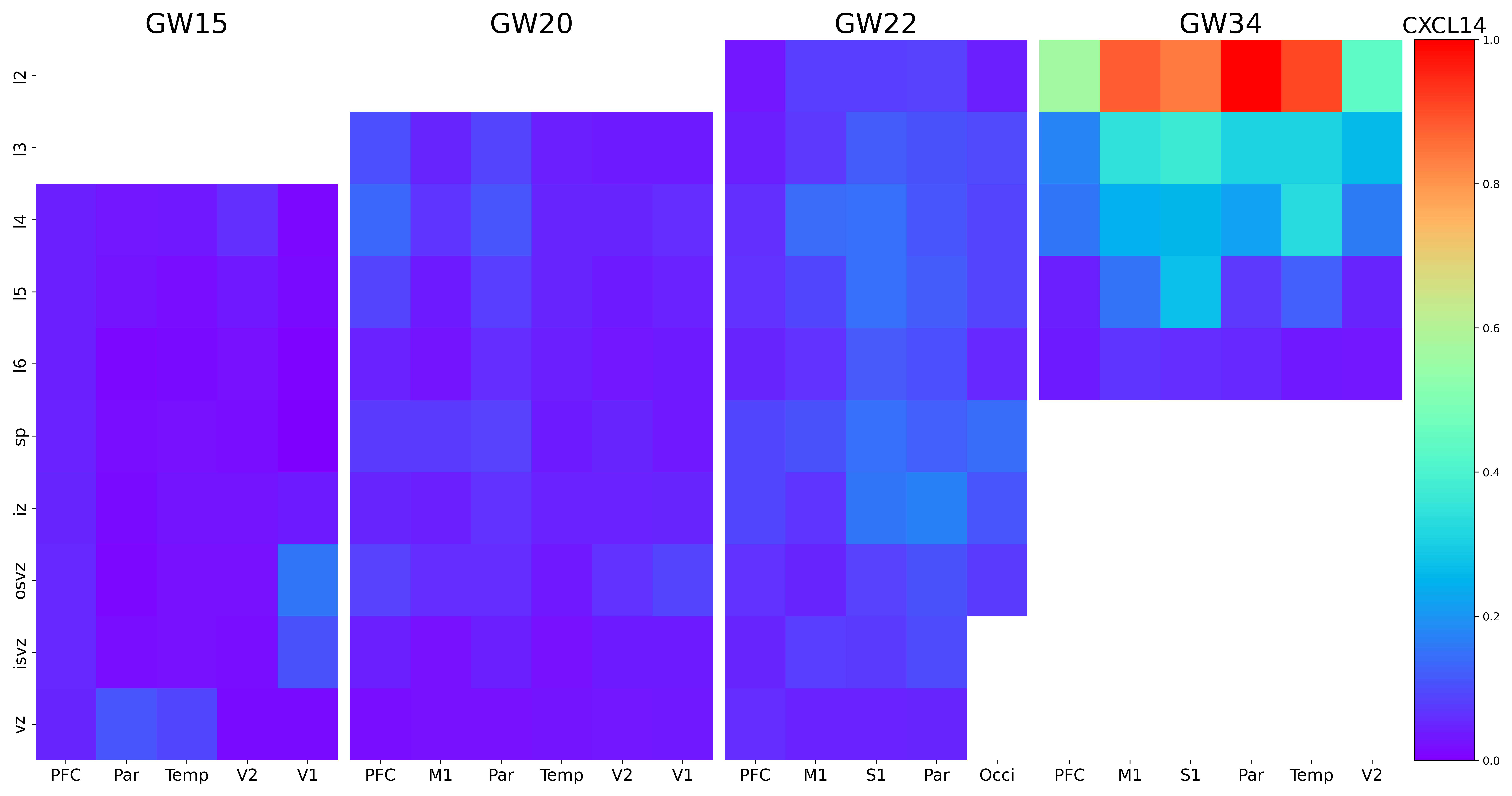

Supplement: Supplementary file 4 — Source Data Fig. 3: Expression pattern heatmap for all 300 genes in the MERFISH. [file 41586_2025_9010_MOESM4_ESM.zip › CXCL14.png]

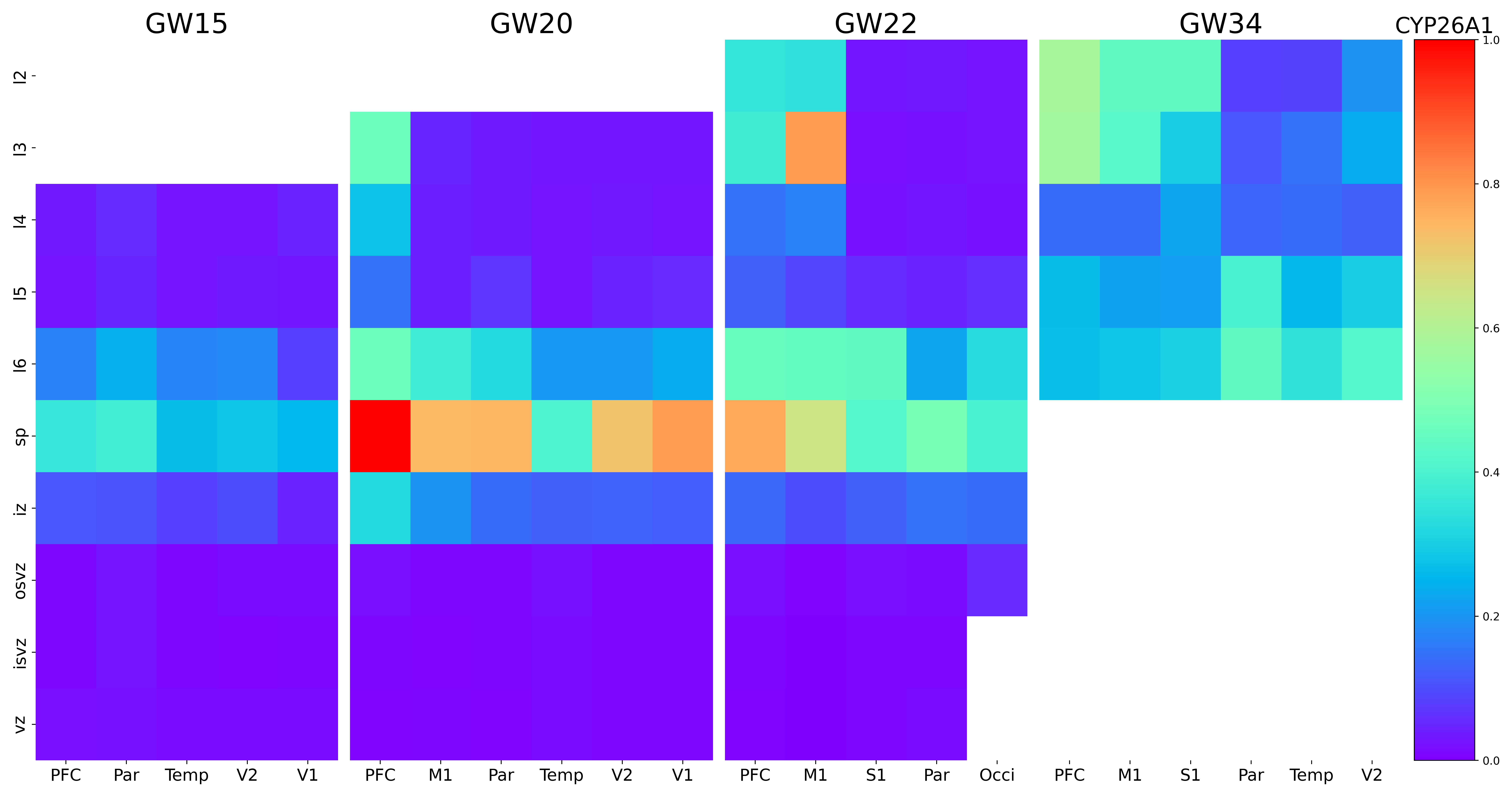

Supplement: Supplementary file 4 — Source Data Fig. 3: Expression pattern heatmap for all 300 genes in the MERFISH. [file 41586_2025_9010_MOESM4_ESM.zip › CYP26A1.png]

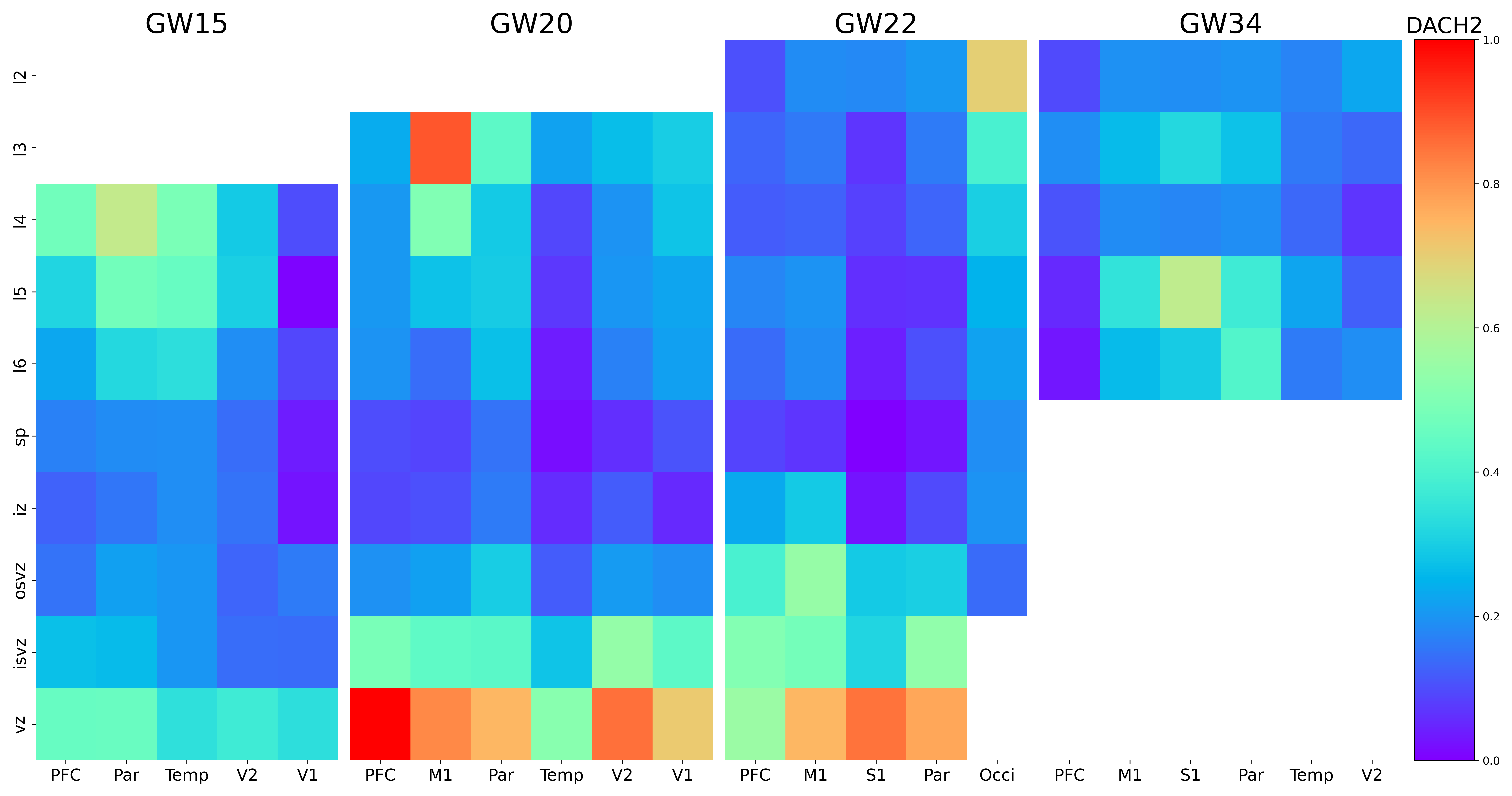

Supplement: Supplementary file 4 — Source Data Fig. 3: Expression pattern heatmap for all 300 genes in the MERFISH. [file 41586_2025_9010_MOESM4_ESM.zip › DACH2.png]

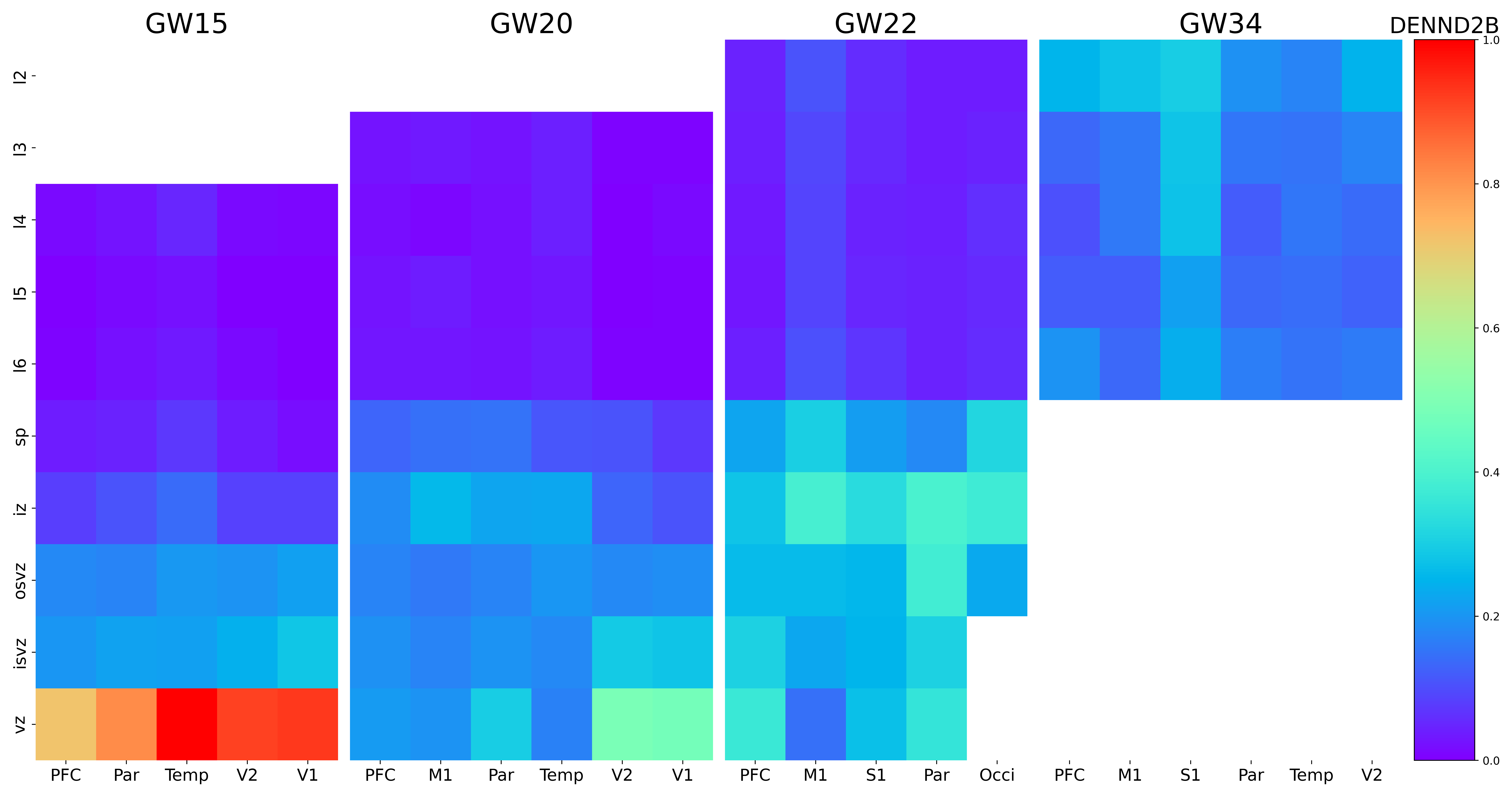

Supplement: Supplementary file 4 — Source Data Fig. 3: Expression pattern heatmap for all 300 genes in the MERFISH. [file 41586_2025_9010_MOESM4_ESM.zip › DENND2B.png]

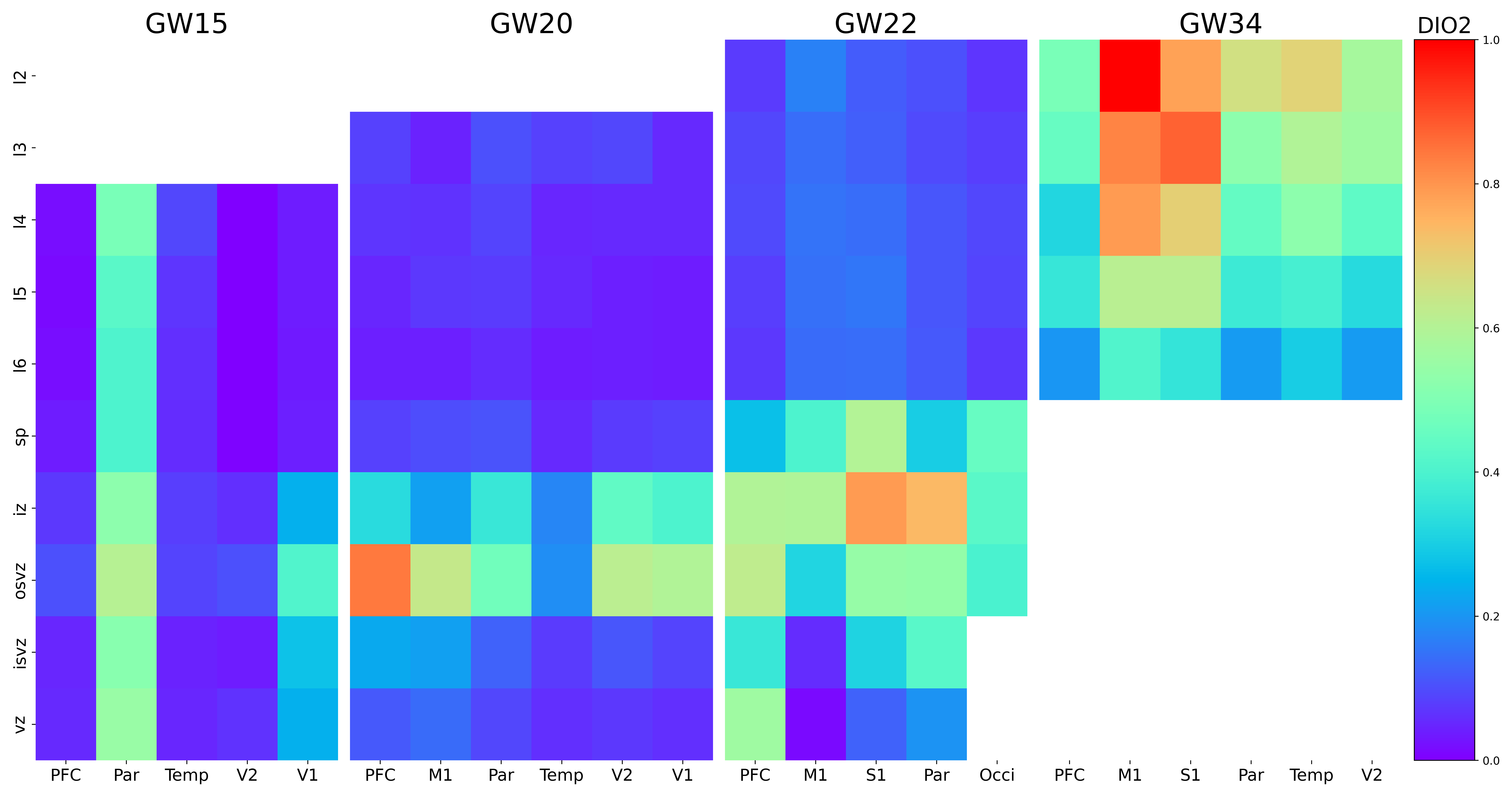

Supplement: Supplementary file 4 — Source Data Fig. 3: Expression pattern heatmap for all 300 genes in the MERFISH. [file 41586_2025_9010_MOESM4_ESM.zip › DIO2.png]

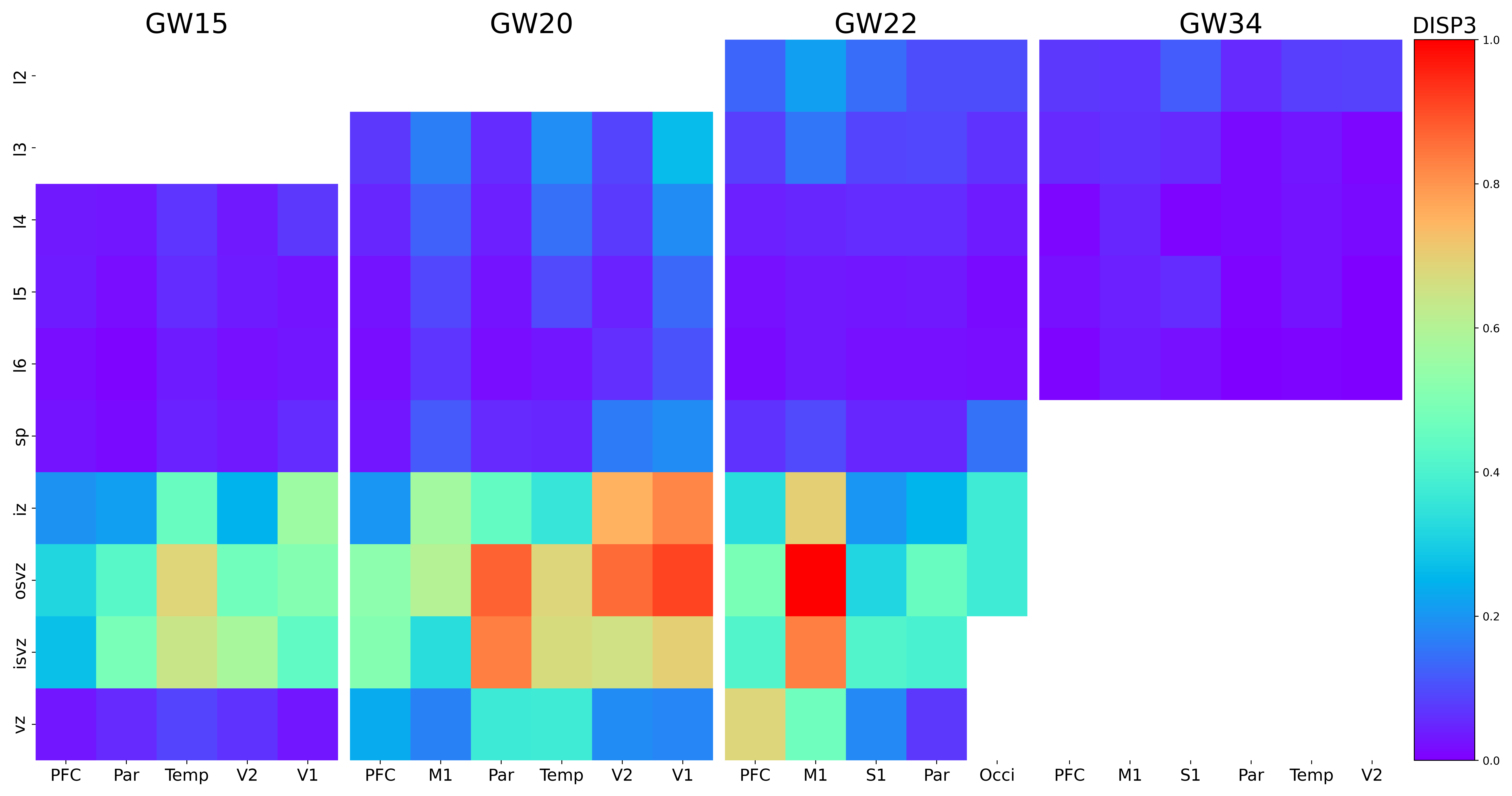

Supplement: Supplementary file 4 — Source Data Fig. 3: Expression pattern heatmap for all 300 genes in the MERFISH. [file 41586_2025_9010_MOESM4_ESM.zip › DISP3.png]

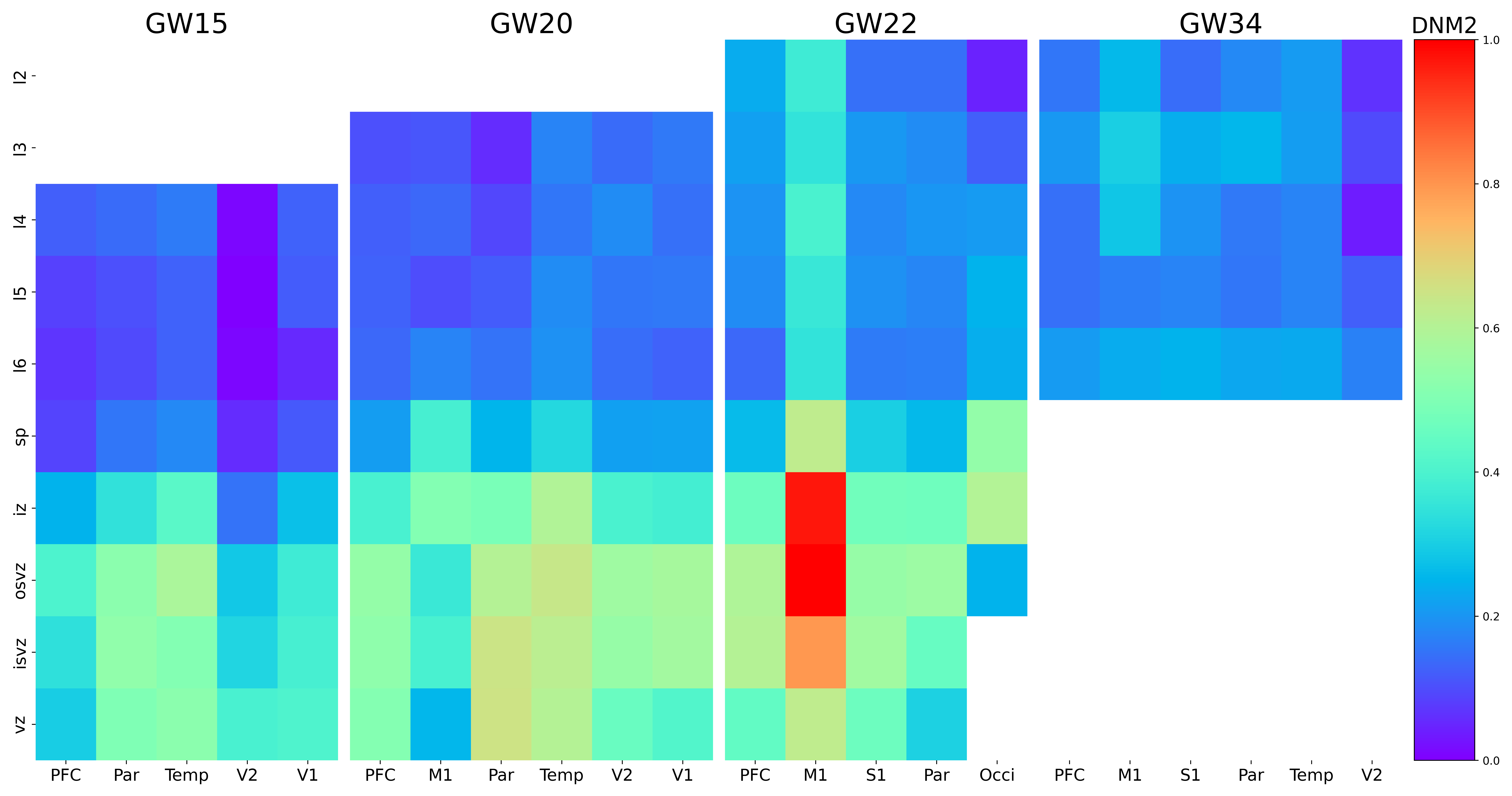

Supplement: Supplementary file 4 — Source Data Fig. 3: Expression pattern heatmap for all 300 genes in the MERFISH. [file 41586_2025_9010_MOESM4_ESM.zip › DNM2.png]

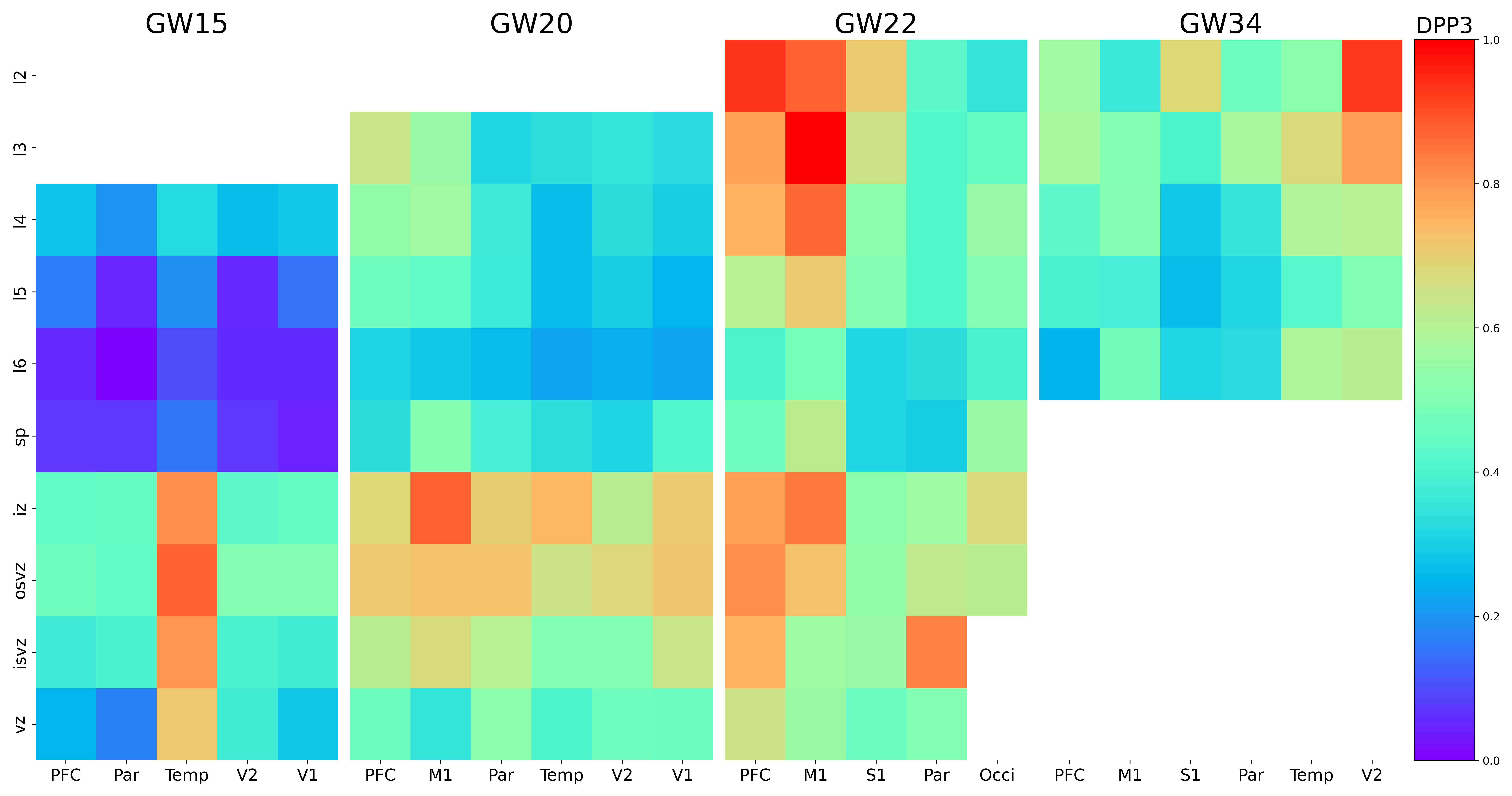

Supplement: Supplementary file 4 — Source Data Fig. 3: Expression pattern heatmap for all 300 genes in the MERFISH. [file 41586_2025_9010_MOESM4_ESM.zip › DPP3.png]

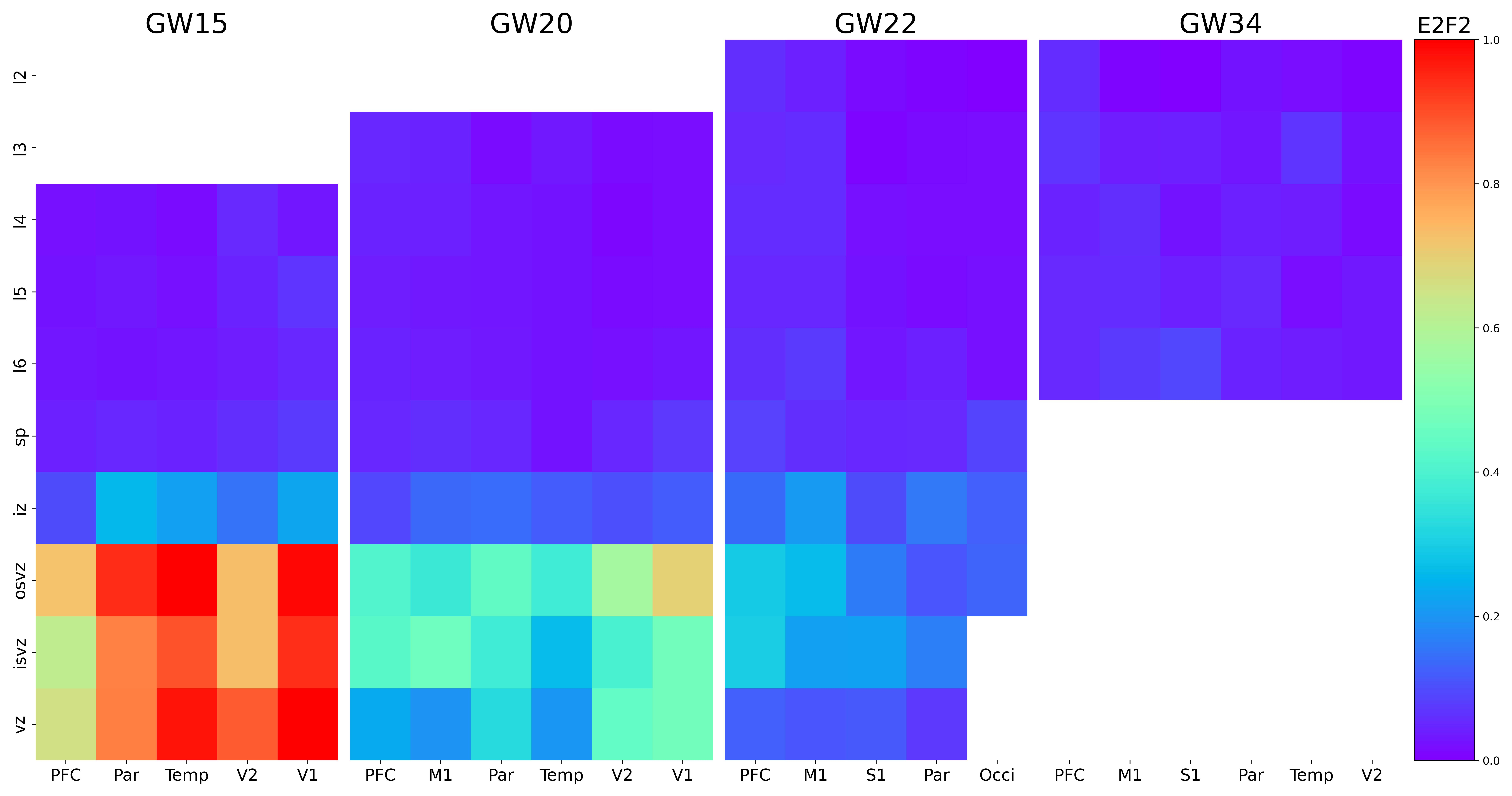

Supplement: Supplementary file 4 — Source Data Fig. 3: Expression pattern heatmap for all 300 genes in the MERFISH. [file 41586_2025_9010_MOESM4_ESM.zip › E2F2.png]

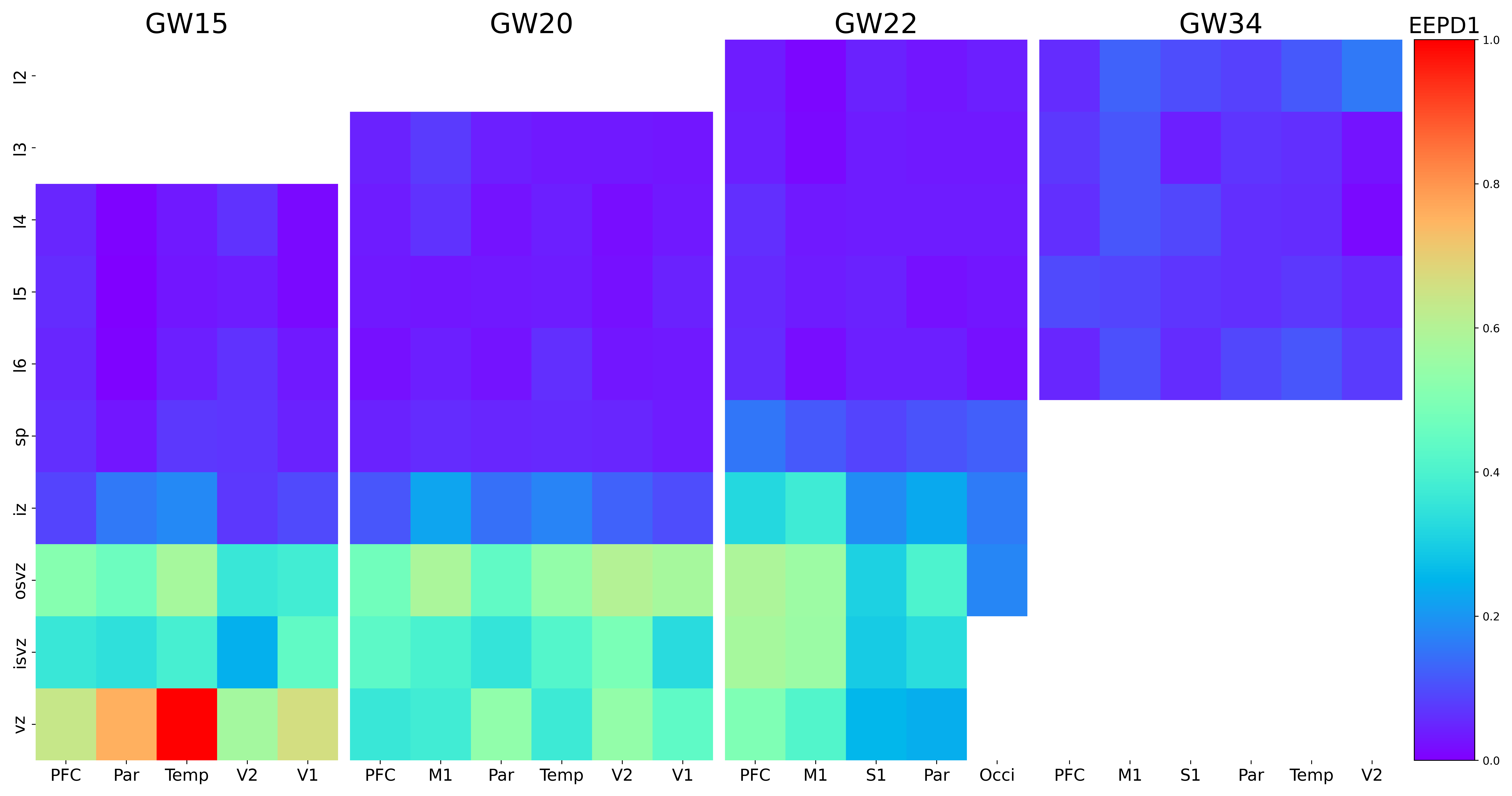

Supplement: Supplementary file 4 — Source Data Fig. 3: Expression pattern heatmap for all 300 genes in the MERFISH. [file 41586_2025_9010_MOESM4_ESM.zip › EEPD1.png]

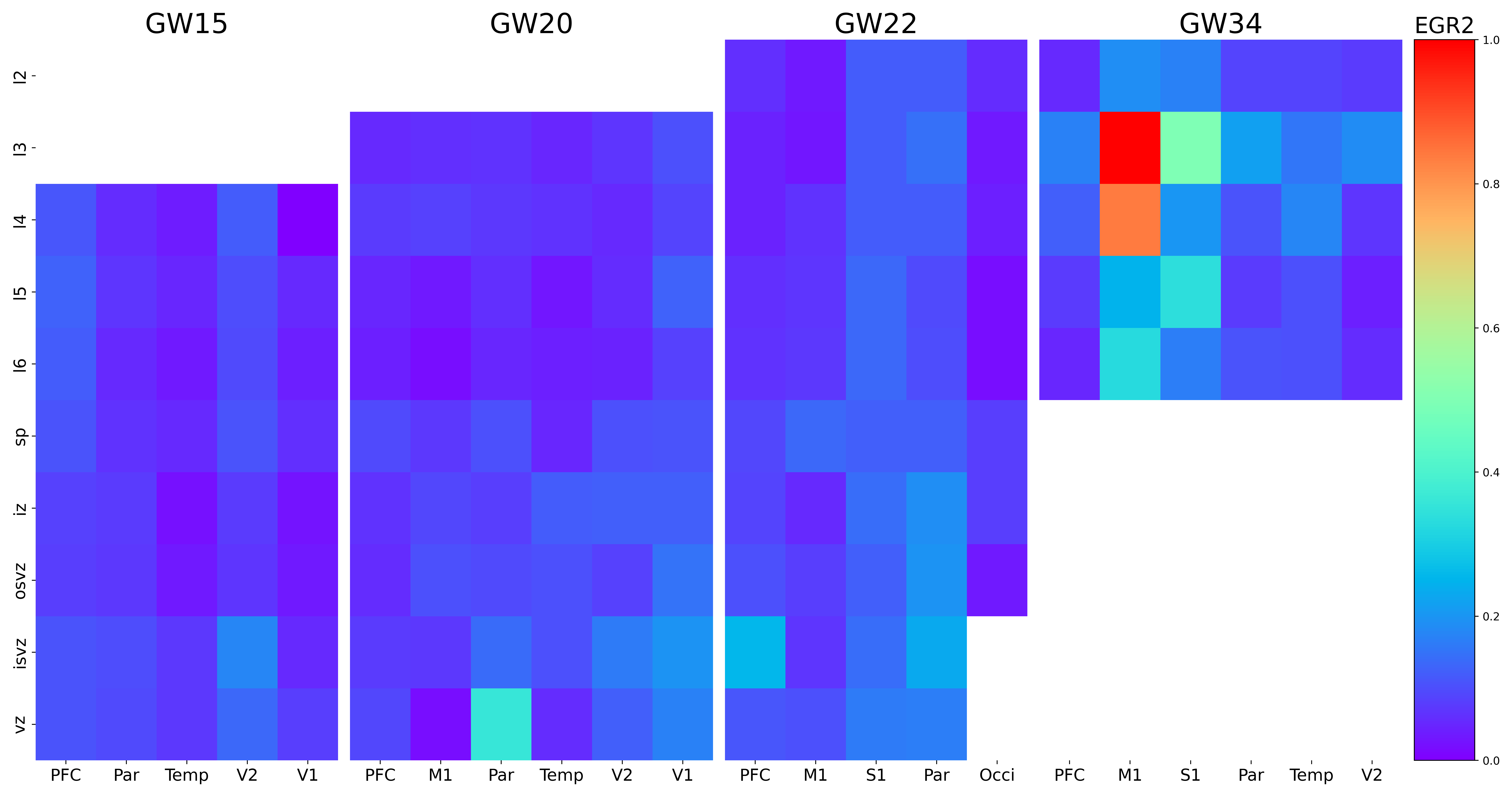

Supplement: Supplementary file 4 — Source Data Fig. 3: Expression pattern heatmap for all 300 genes in the MERFISH. [file 41586_2025_9010_MOESM4_ESM.zip › EGR2.png]

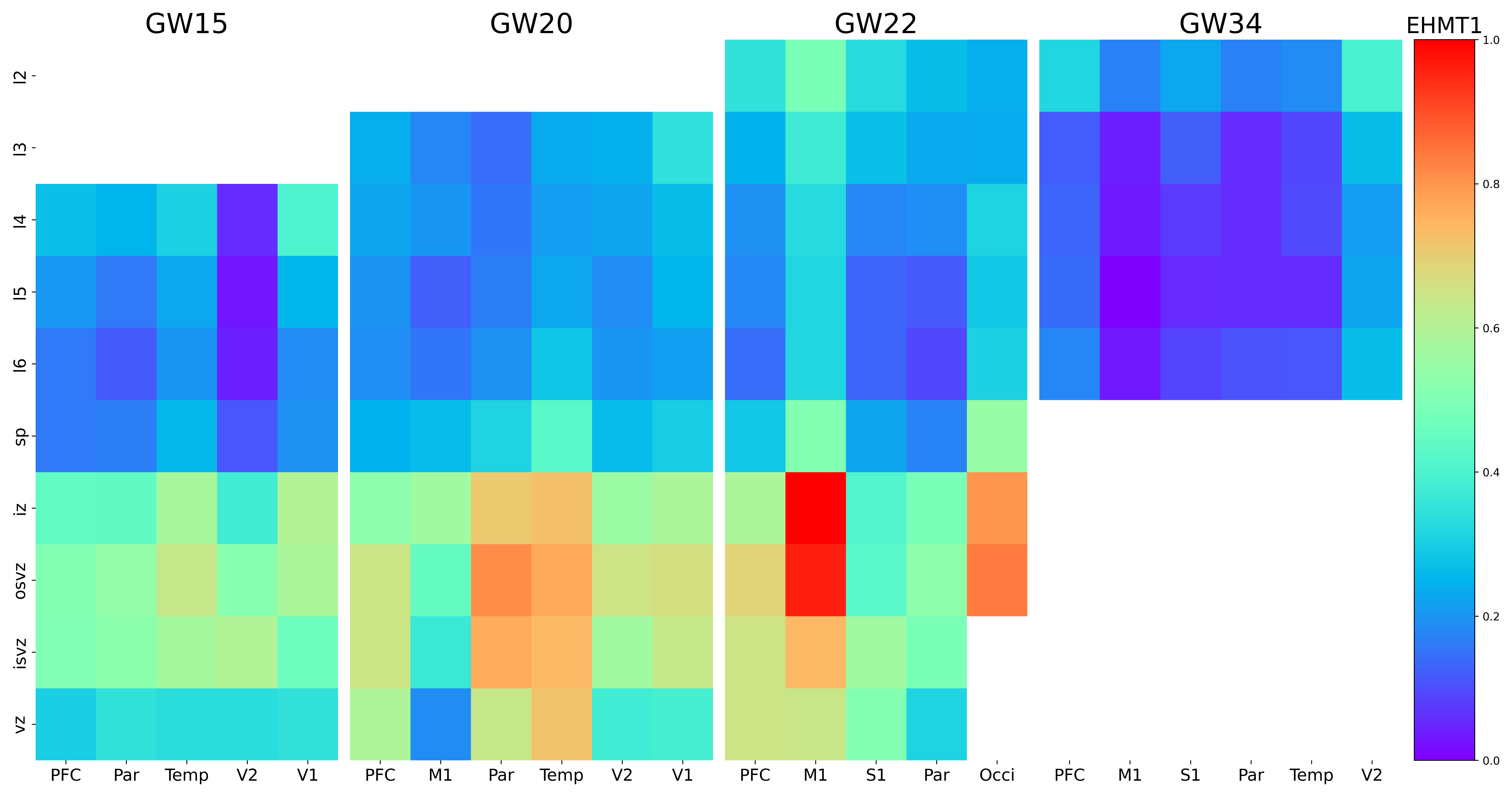

Supplement: Supplementary file 4 — Source Data Fig. 3: Expression pattern heatmap for all 300 genes in the MERFISH. [file 41586_2025_9010_MOESM4_ESM.zip › EHMT1.png]

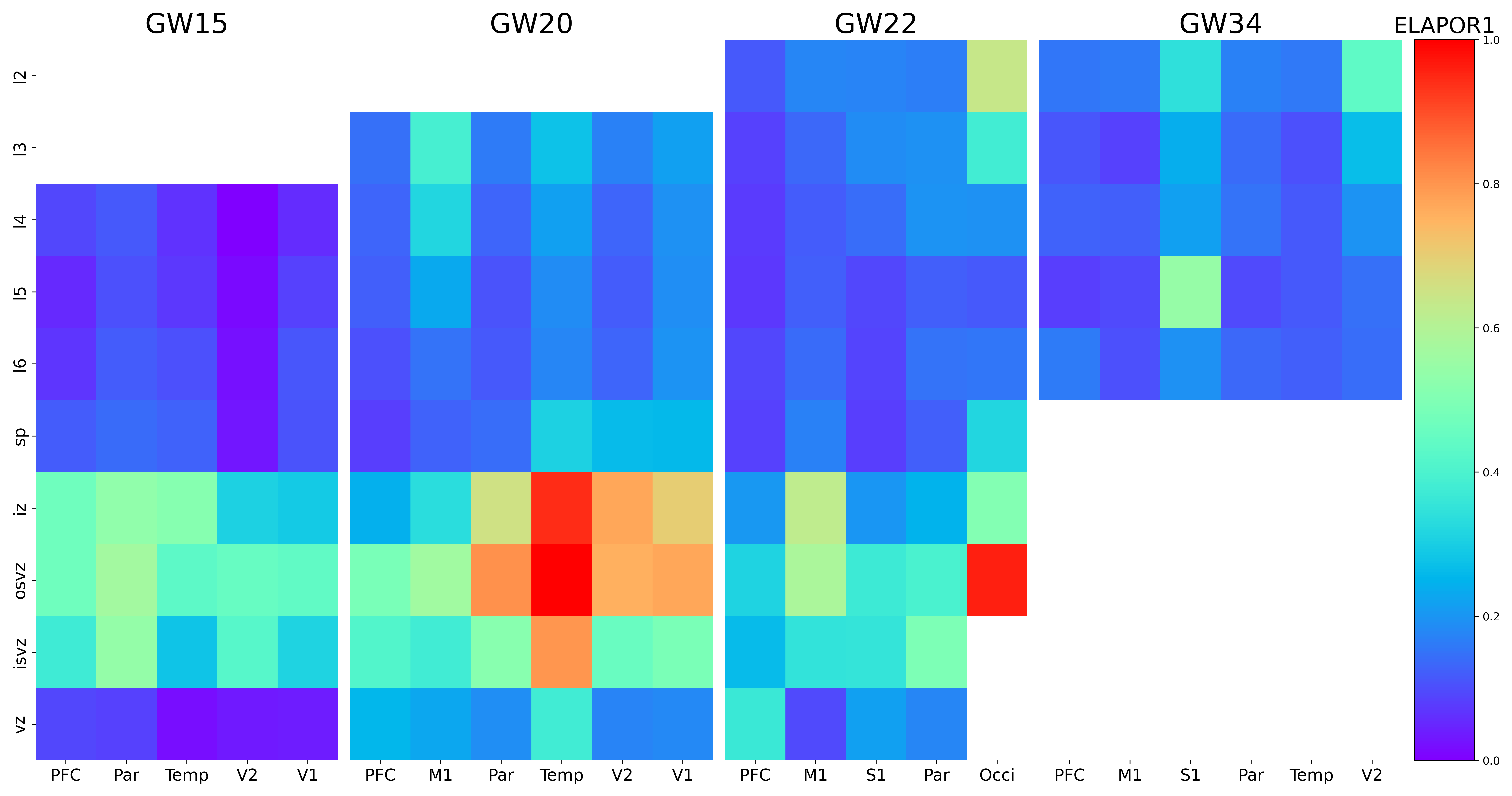

Supplement: Supplementary file 4 — Source Data Fig. 3: Expression pattern heatmap for all 300 genes in the MERFISH. [file 41586_2025_9010_MOESM4_ESM.zip › ELAPOR1.png]

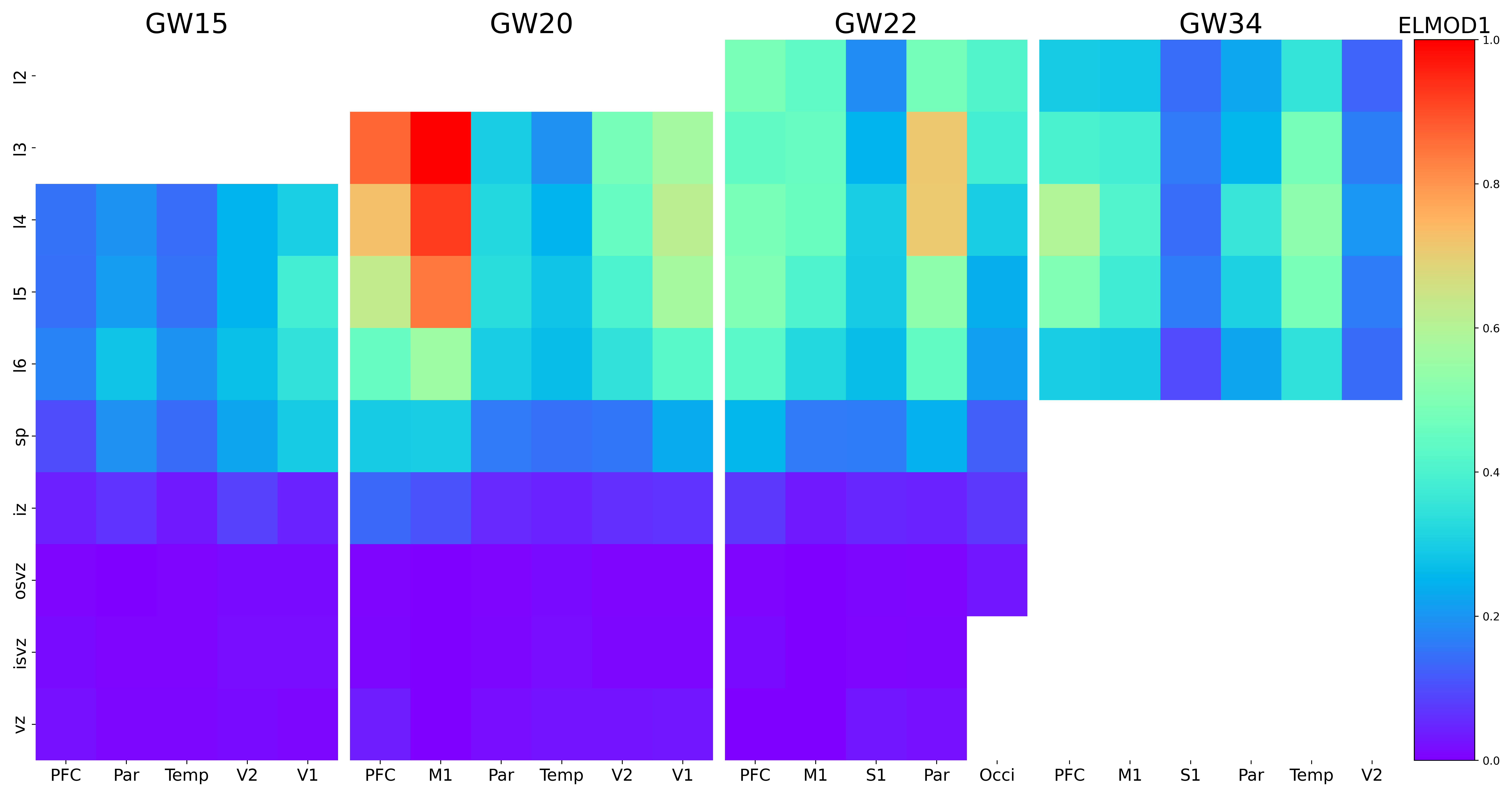

Supplement: Supplementary file 4 — Source Data Fig. 3: Expression pattern heatmap for all 300 genes in the MERFISH. [file 41586_2025_9010_MOESM4_ESM.zip › ELMOD1.png]

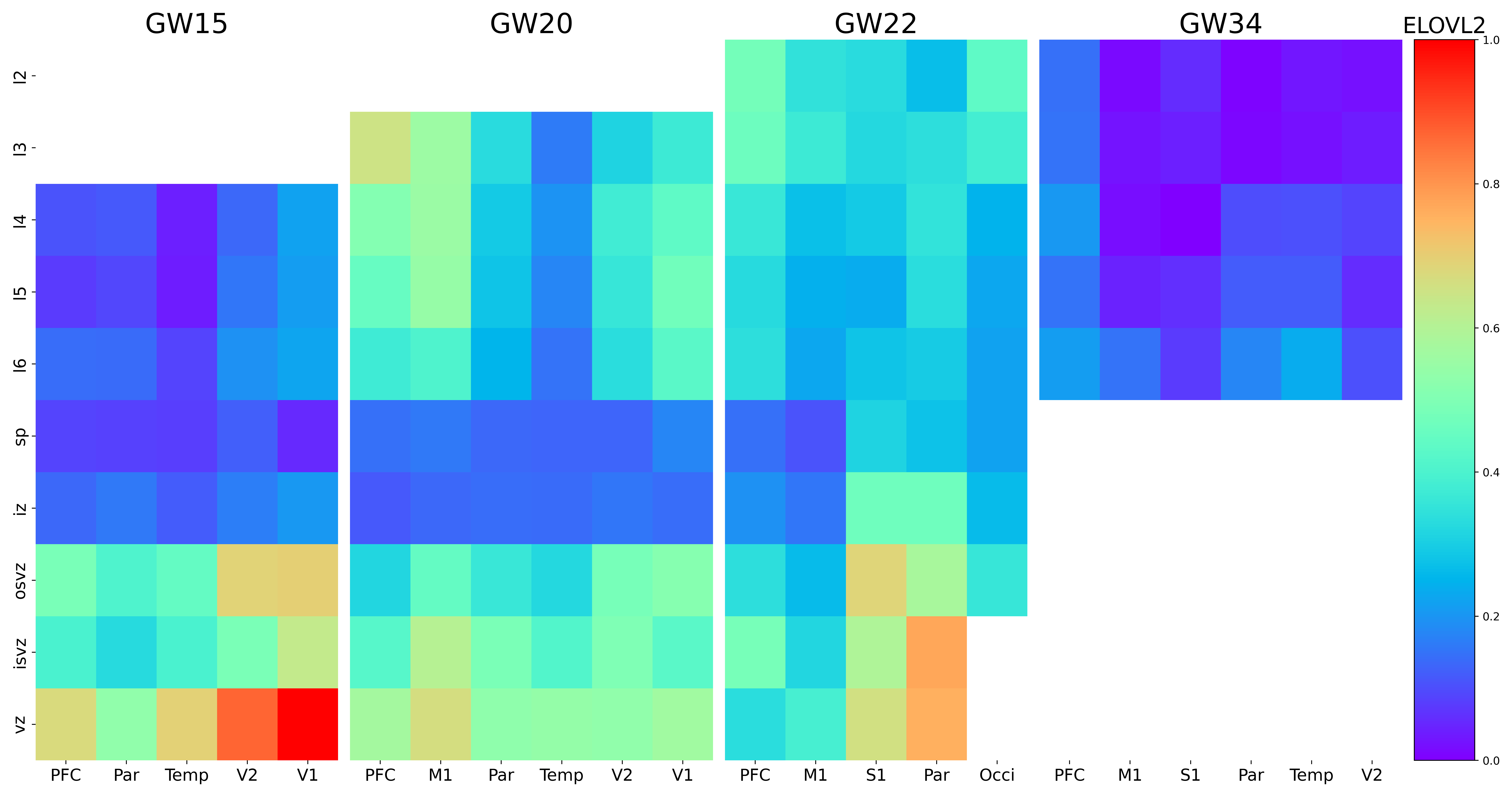

Supplement: Supplementary file 4 — Source Data Fig. 3: Expression pattern heatmap for all 300 genes in the MERFISH. [file 41586_2025_9010_MOESM4_ESM.zip › ELOVL2.png]

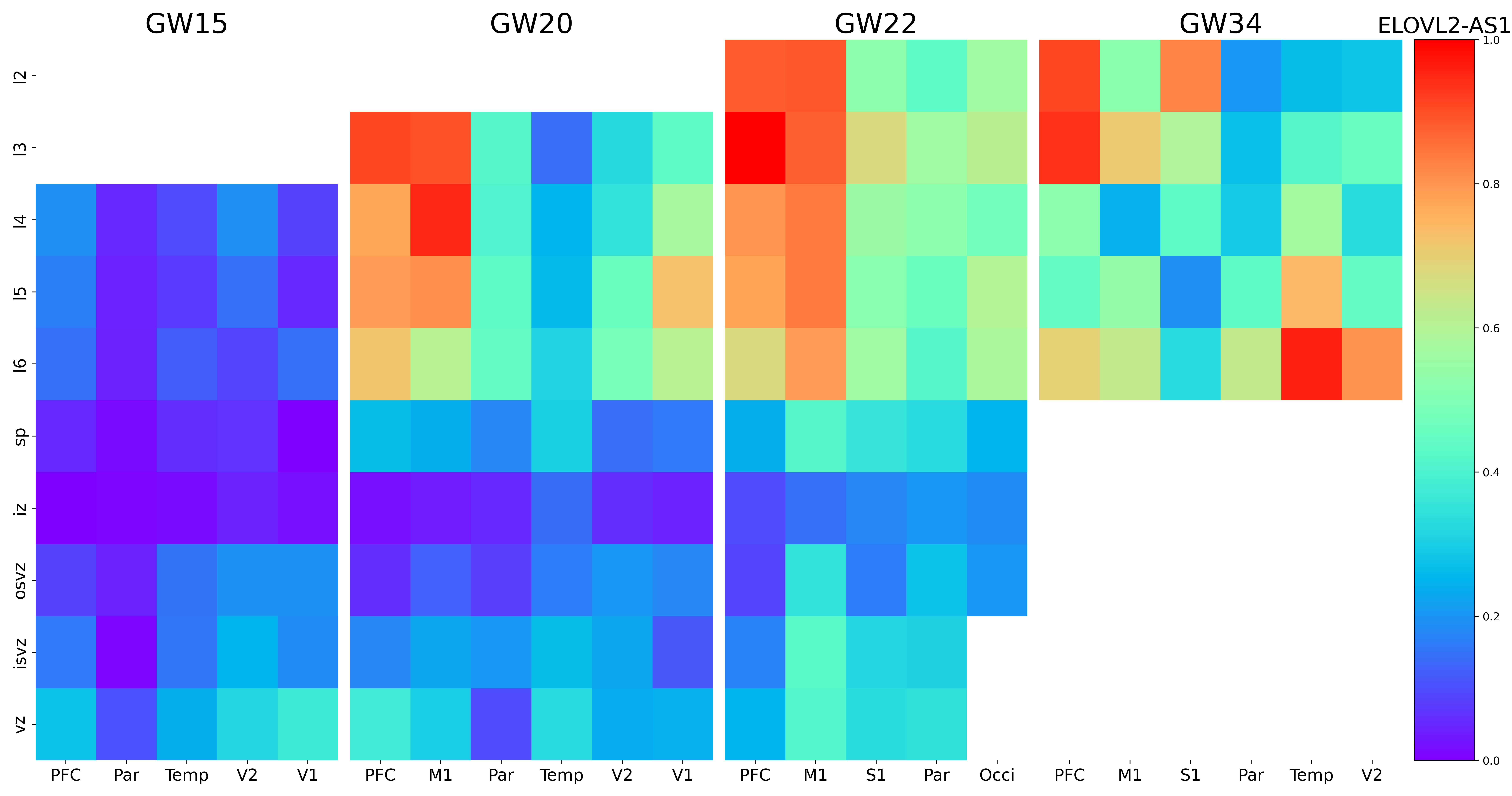

Supplement: Supplementary file 4 — Source Data Fig. 3: Expression pattern heatmap for all 300 genes in the MERFISH. [file 41586_2025_9010_MOESM4_ESM.zip › ELOVL2-AS1.png]

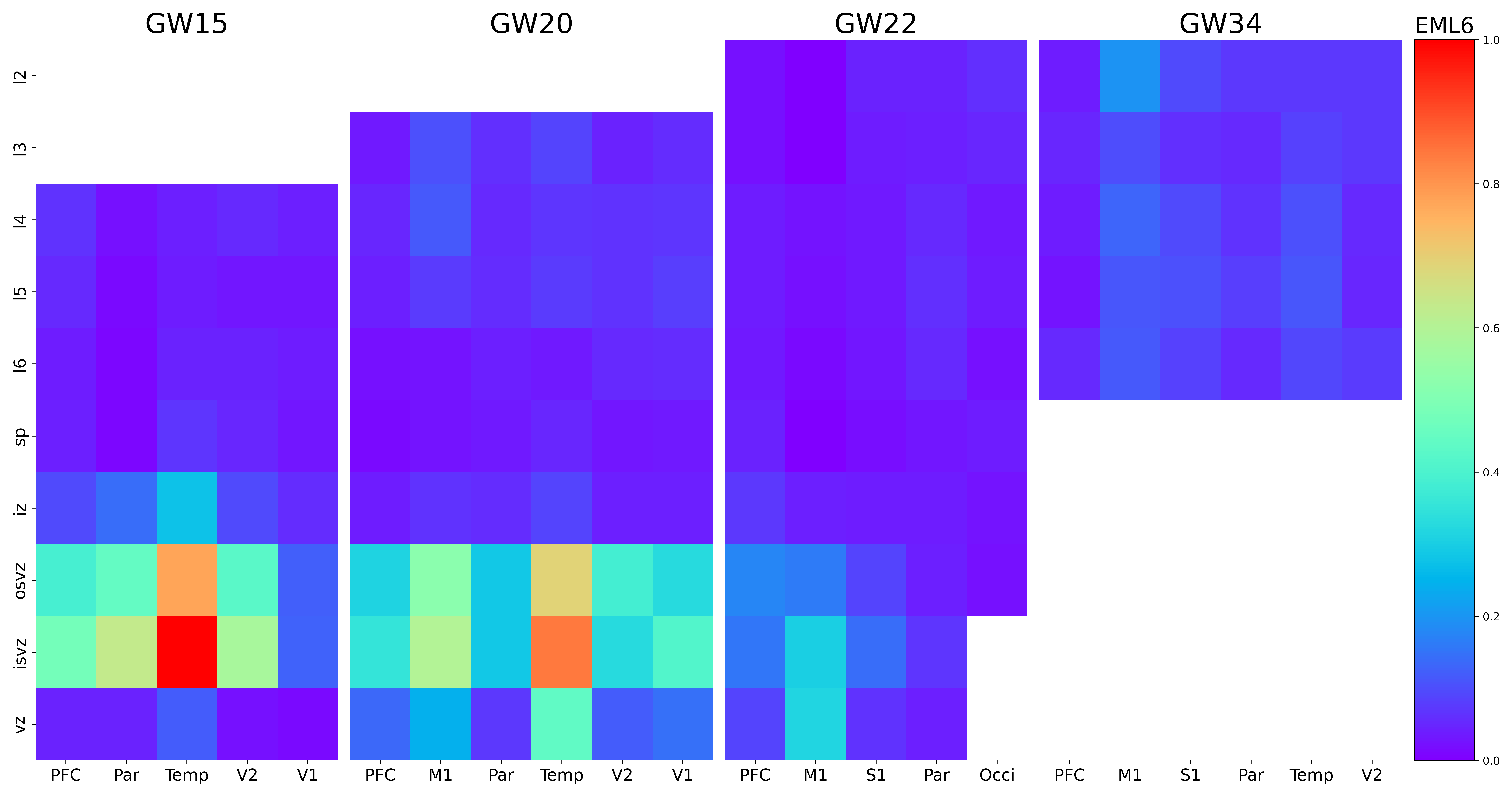

Supplement: Supplementary file 4 — Source Data Fig. 3: Expression pattern heatmap for all 300 genes in the MERFISH. [file 41586_2025_9010_MOESM4_ESM.zip › EML6.png]

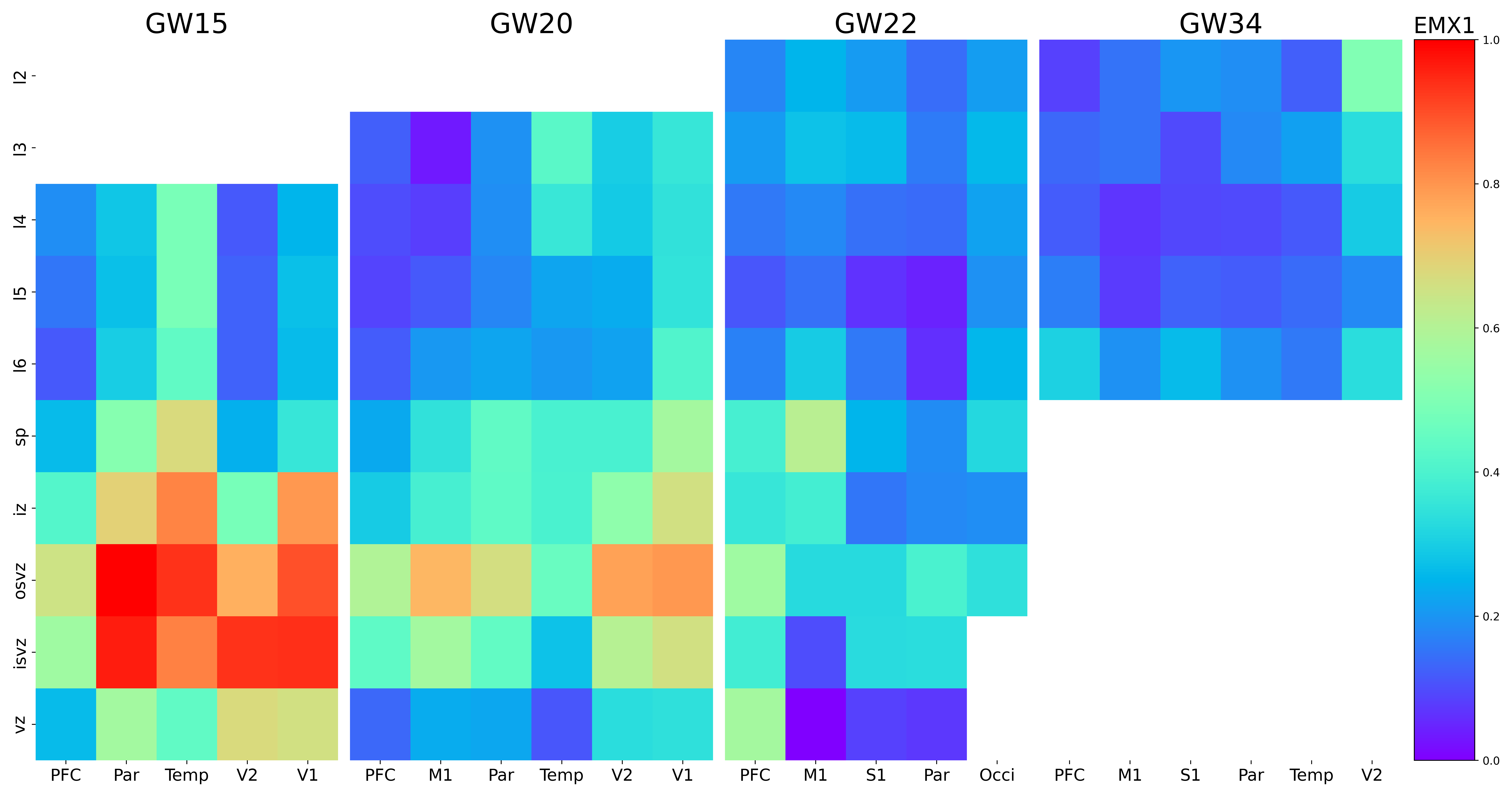

Supplement: Supplementary file 4 — Source Data Fig. 3: Expression pattern heatmap for all 300 genes in the MERFISH. [file 41586_2025_9010_MOESM4_ESM.zip › EMX1.png]

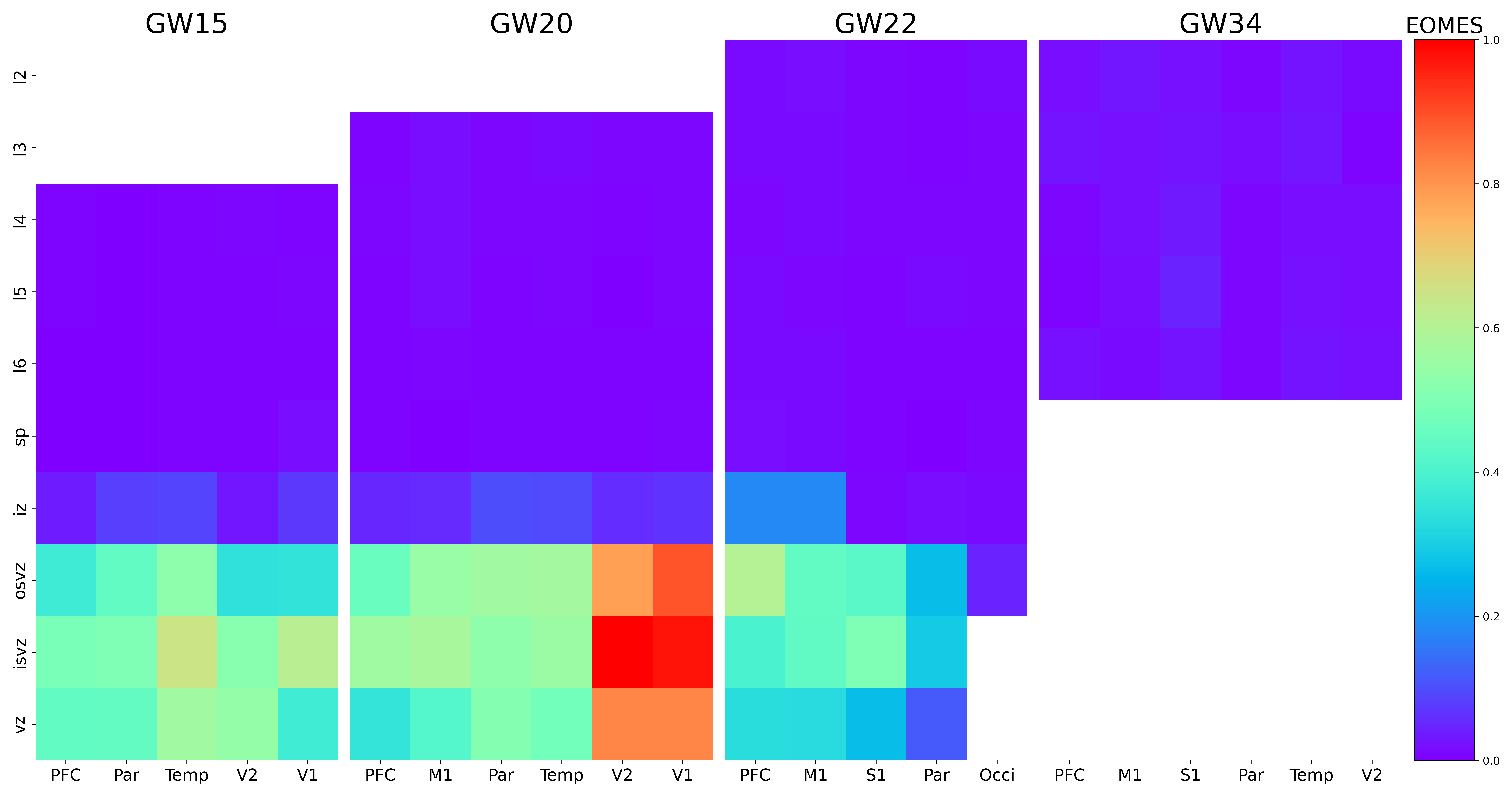

Supplement: Supplementary file 4 — Source Data Fig. 3: Expression pattern heatmap for all 300 genes in the MERFISH. [file 41586_2025_9010_MOESM4_ESM.zip › EOMES.png]

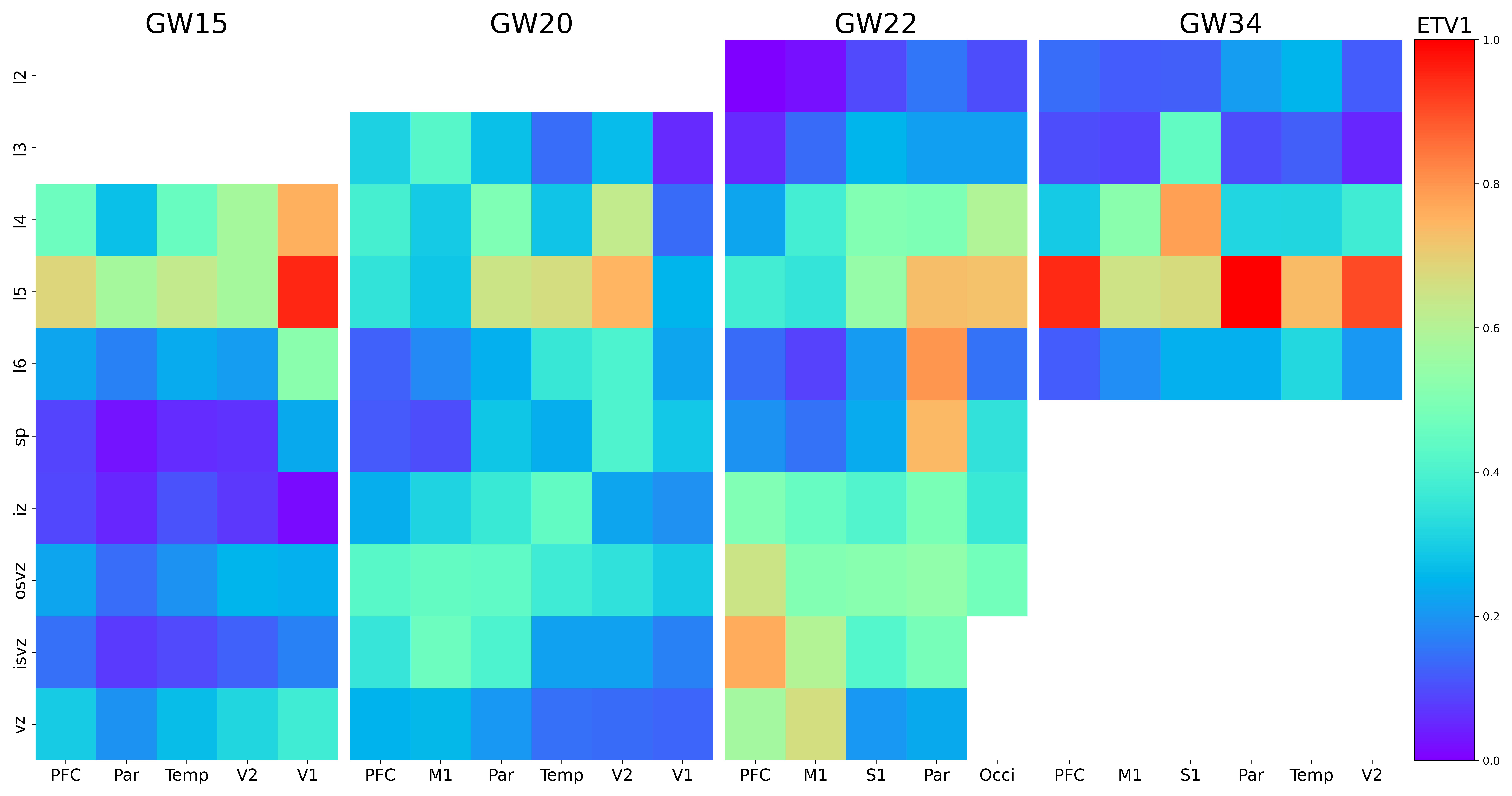

Supplement: Supplementary file 4 — Source Data Fig. 3: Expression pattern heatmap for all 300 genes in the MERFISH. [file 41586_2025_9010_MOESM4_ESM.zip › ETV1.png]

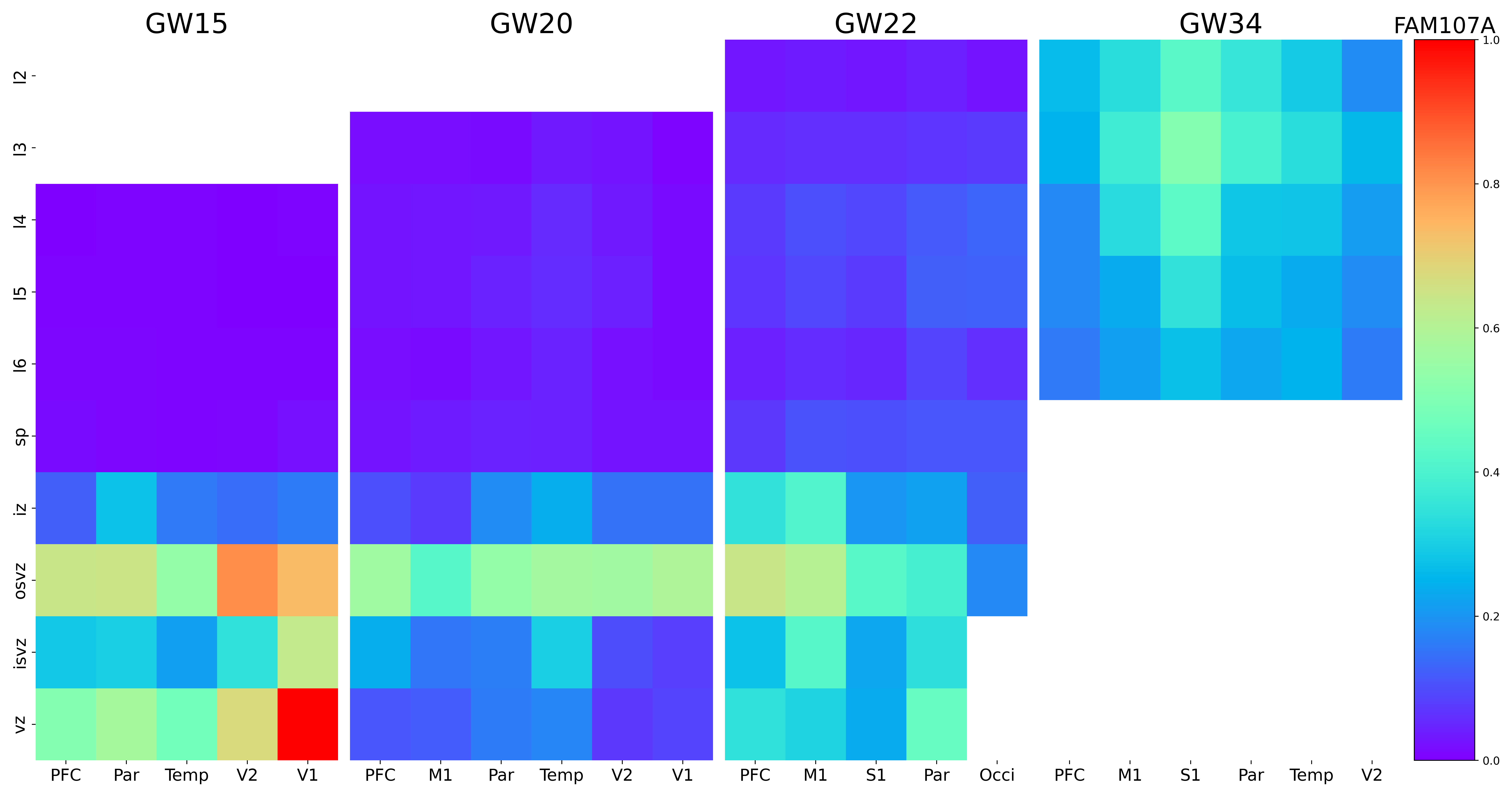

Supplement: Supplementary file 4 — Source Data Fig. 3: Expression pattern heatmap for all 300 genes in the MERFISH. [file 41586_2025_9010_MOESM4_ESM.zip › FAM107A.png]

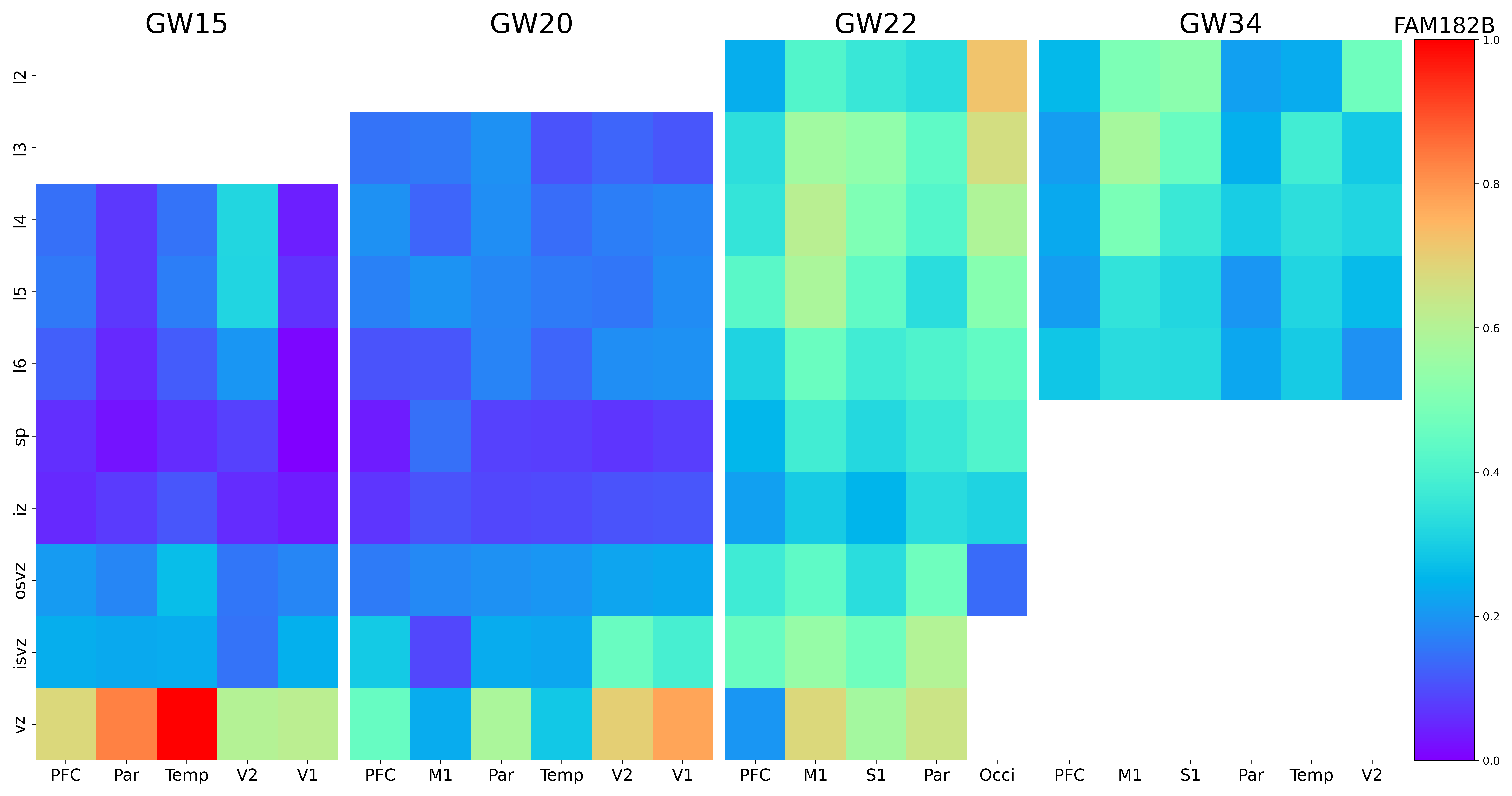

Supplement: Supplementary file 4 — Source Data Fig. 3: Expression pattern heatmap for all 300 genes in the MERFISH. [file 41586_2025_9010_MOESM4_ESM.zip › FAM182B.png]

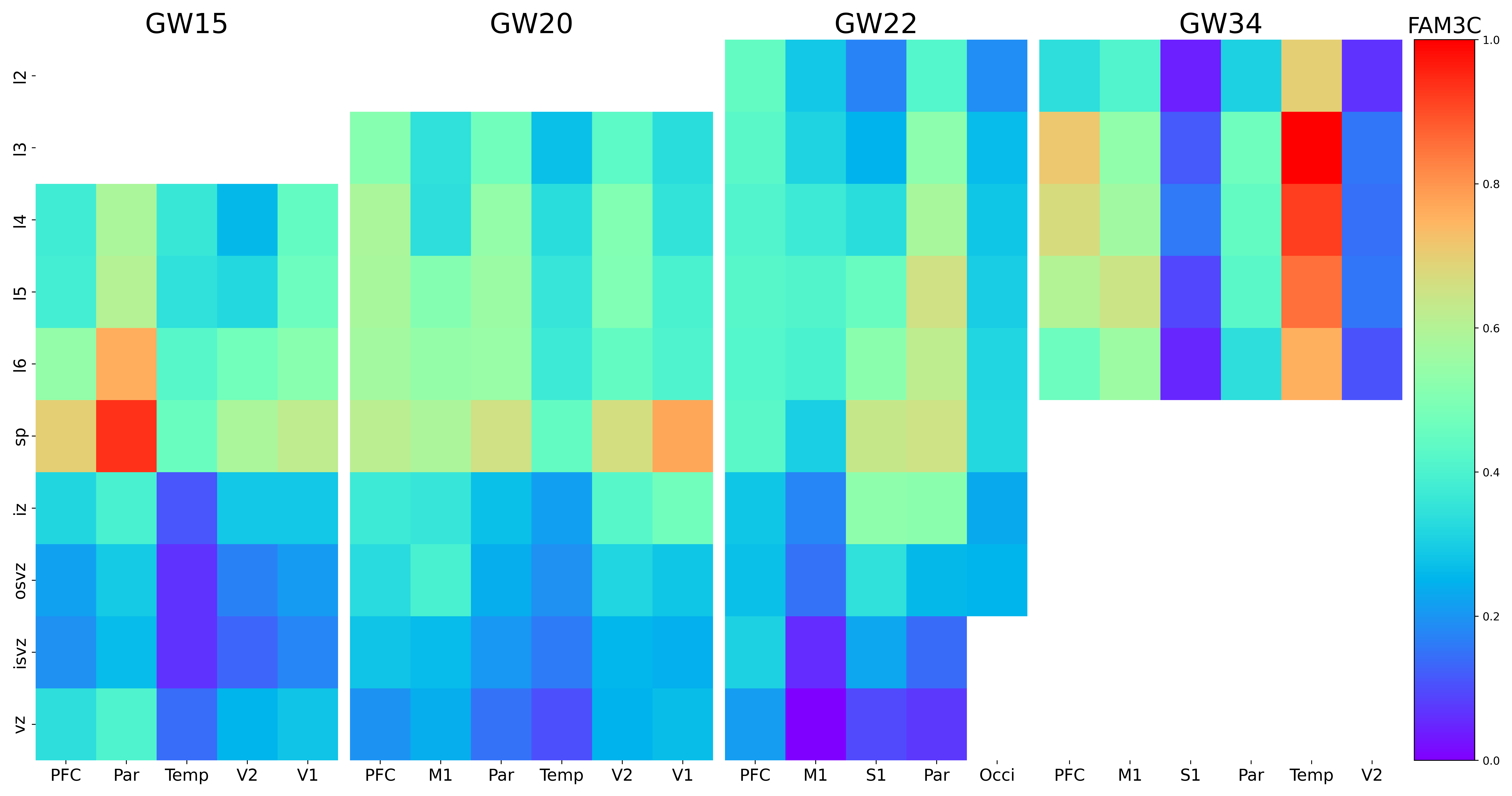

Supplement: Supplementary file 4 — Source Data Fig. 3: Expression pattern heatmap for all 300 genes in the MERFISH. [file 41586_2025_9010_MOESM4_ESM.zip › FAM3C.png]

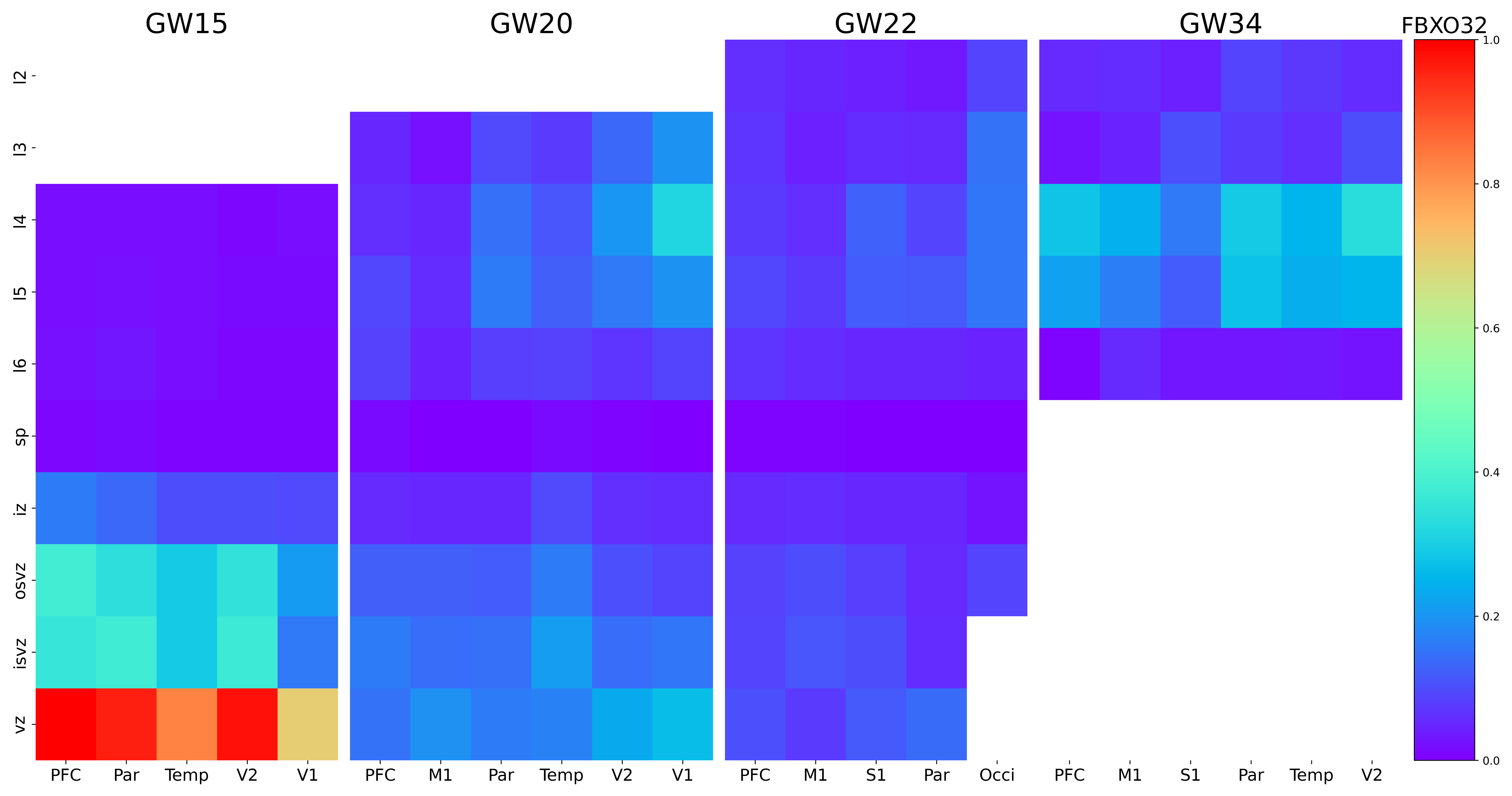

Supplement: Supplementary file 4 — Source Data Fig. 3: Expression pattern heatmap for all 300 genes in the MERFISH. [file 41586_2025_9010_MOESM4_ESM.zip › FBXO32.png]

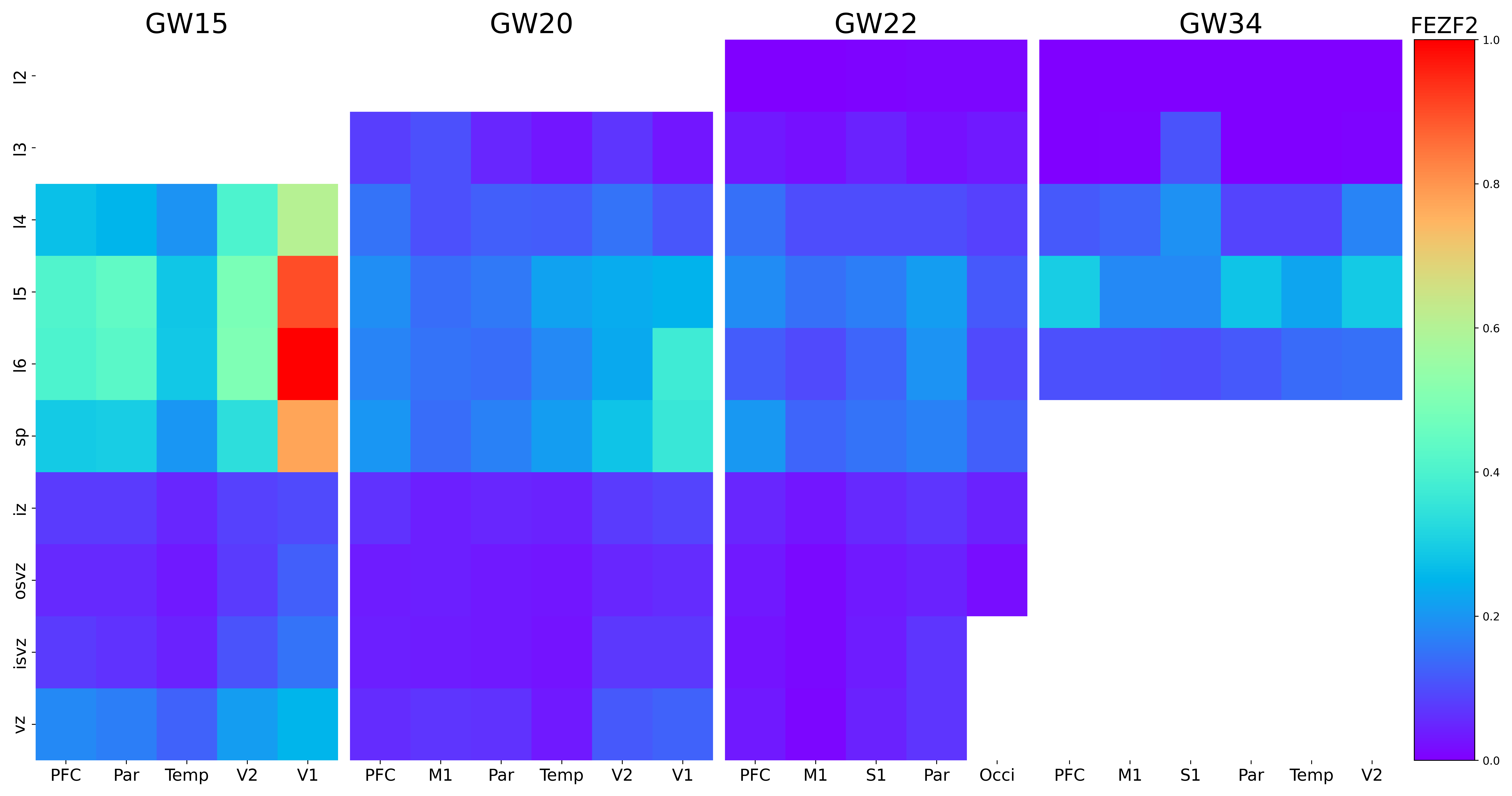

Supplement: Supplementary file 4 — Source Data Fig. 3: Expression pattern heatmap for all 300 genes in the MERFISH. [file 41586_2025_9010_MOESM4_ESM.zip › FEZF2.png]

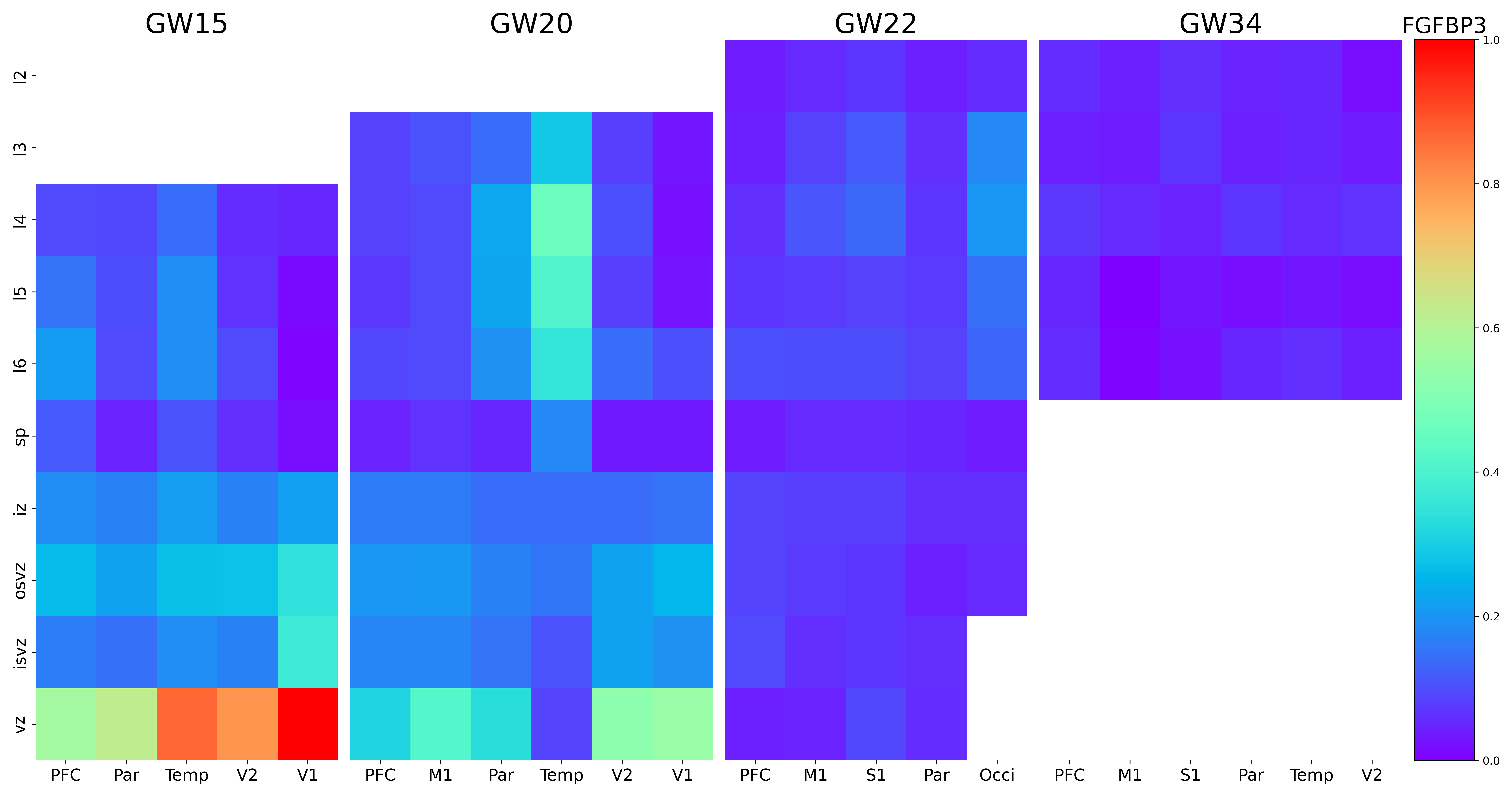

Supplement: Supplementary file 4 — Source Data Fig. 3: Expression pattern heatmap for all 300 genes in the MERFISH. [file 41586_2025_9010_MOESM4_ESM.zip › FGFBP3.png]

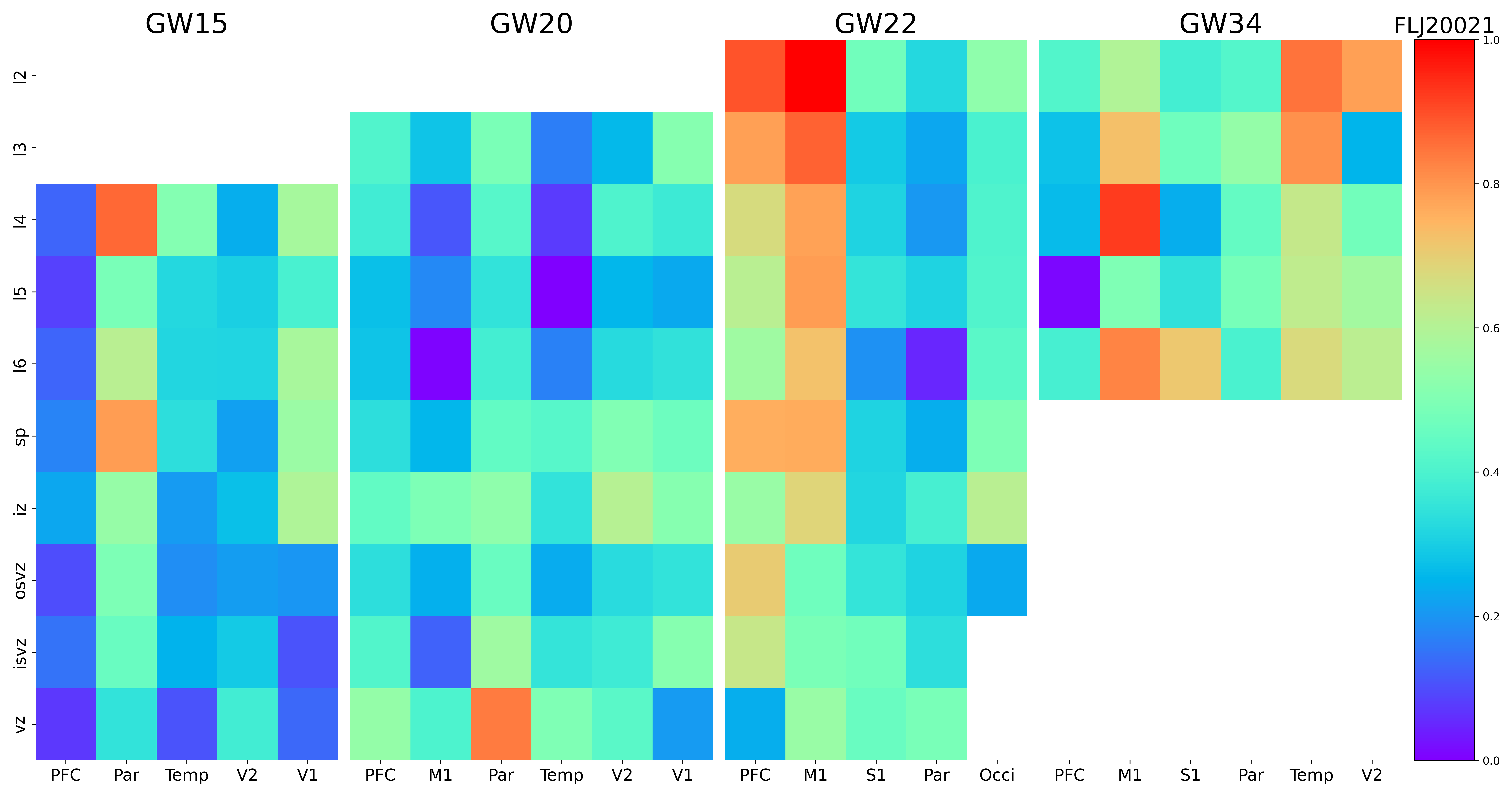

Supplement: Supplementary file 4 — Source Data Fig. 3: Expression pattern heatmap for all 300 genes in the MERFISH. [file 41586_2025_9010_MOESM4_ESM.zip › FLJ20021.png]

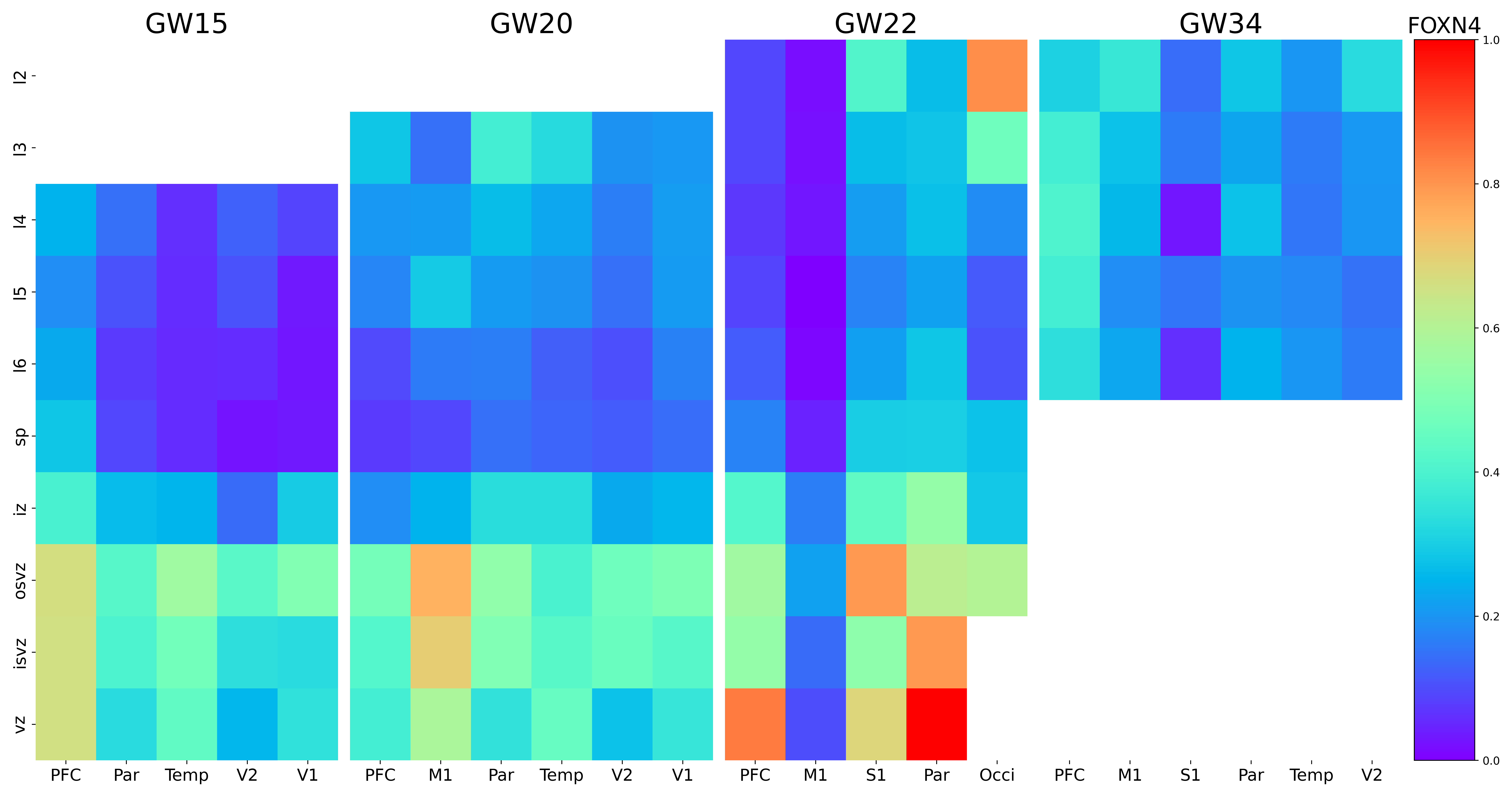

Supplement: Supplementary file 4 — Source Data Fig. 3: Expression pattern heatmap for all 300 genes in the MERFISH. [file 41586_2025_9010_MOESM4_ESM.zip › FOXN4.png]
